# Supplementary material for: Photoredox cooperative N-heterocyclic carbene/palladium-catalysed alkylacylation of alkenes
Source: Nat Commun. 2022 Sep 30;13:5754. doi: 10.1038/s41467-022-33444-0 (PMC9525644; doi:10.1038/s41467-022-33444-0)
Supplement: Supplementary file 1 — Supplementary Information [file 41467_2022_33444_MOESM1_ESM.pdf]

Supplementary Information

Photoredox Cooperative N-Heterocyclic Carbene/Palladium-Catalysed Alkylacylation of Alkenes

You-Feng Han,[1,2]† Ying Huang,[1,2]† Hao Liu,[1,2] Zhong-Hua Gao,[1,2] Chun-Lin Zhang,[1]* Song Ye[1,2]*

[1] Beijing National Laboratory for Molecular Sciences, CAS Key Laboratory of Molecular Recognition and Function, CAS Research/Education Center for Excellence in Molecular Sciences, Institute of Chemistry, Chinese Academy of Sciences, Beijing 100190, China

[2] University of Chinese Academy of Sciences, Beijing 100049, China.

†These authors contributed equally: Y.-F. H. and Y. H.

* = corresponding author. E-mail: zhangchunlin@iccas.ac.cn; songye@iccas.ac.cn

Table of Contents

1. Supplementary Notes. S2

2. Supplementary Methods. S3

2.1 Preparation of starting materials: tertiary alkyl bromides 3k-l.....S3

2.2 Alkylacylation of alkenes (Fig. 2).....S3

2.3 Cascade alkylacylation/cyclopropanation (Fig. 3 and Fig. 4).....S37

2.4 Gram-scale reaction and further transformations (Fig. 5).....S43

2.5 Control experiments S46

2.6 UV-Visible absorption analysis S50

2.7 Light on/off experiments S51

3. Supplementary Figures S52

3.1 NMR Spectra S52

4. Supplementary References.....S217

1. Supplementary Notes.

Unless otherwise indicated, all reactions were carried out under an N_2 atmosphere in oven-dried glassware with magnetic stirring. Anhydrous THF , Tol and 1,4-dioxane were distilled from sodium and benzophenone. Styrenes **1**, aldehydes **2** were purchased from Innochem and Energy Chemical. 2-Bromo-2-methylbutane and (2-bromo-2-methylpropyl)benzene **3k-l** were synthesized according to literature,[1] and other alkyl halides **3** were purchased from Innochem and Energy Chemical. Column chromatograph was performed on silica gel 200~300 mesh. All ^{1}H, ^{13}C NMR spectra were recorded on a Bruker AV 300, 400 and 500 spectrometer. Chemical shifts were reported in parts per million (ppm, δ), and the residual solvent peak was used as internal reference. ^{1}HNMR Spectroscopy splitting patterns were designated as singlet (s), doublet (d) and triplet (t). Splitting patterns that could not be interpreted or easily visualized were designated as multiplet (m). Coupling constants were reported in Herz (Hz). High-resolution mass spectra (HRMS) were obtained with the mass analyzer of an orbitrap. Infrared spectra were recorded on a JASCO FT/IR-480 spectrophotometer and reported as wave number (cm^{-1}). UV/vis absorption spectra were recorded on a Jasco V-650 spectrophotometer, equipped with a temperature control unit at 25 ℃, and the samples were measured in Hellma fluorescence QS quartz cuvettes (chamber volume = 3.0 mL) fitted with a PTFE stopper.

2. Supplementary Methods.

2.1 Preparation of starting materials: tertiary alkyl bromides 3k-l

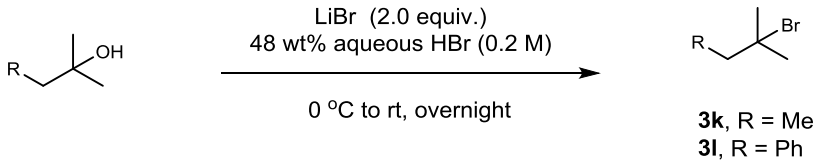

The corresponding tertiary alcohol precursor (10 mmol, 1.0 equiv.) was added LiBr (1.80 g, 20 mmol, 2.0 equiv.) in 48 wt% aqueous HBr (0.2 M, 20 mL) at 0 °C. The reaction mixture was allowed to warm to room temperature and stirred for overnight. The reaction mixture was diluted with ethyl acetate, washed with water and saturated $NaHCO_3$. The organic layer was collected, washed with brine, dried over $MgSO_4$, and concentrated. The residue was purified by column chromatography to afford the desired tertiary bromides **3k-l**, and the experimental data are in agreement with the previous reports.

2.2 Alkylacylation of alkenes (Fig. 2)

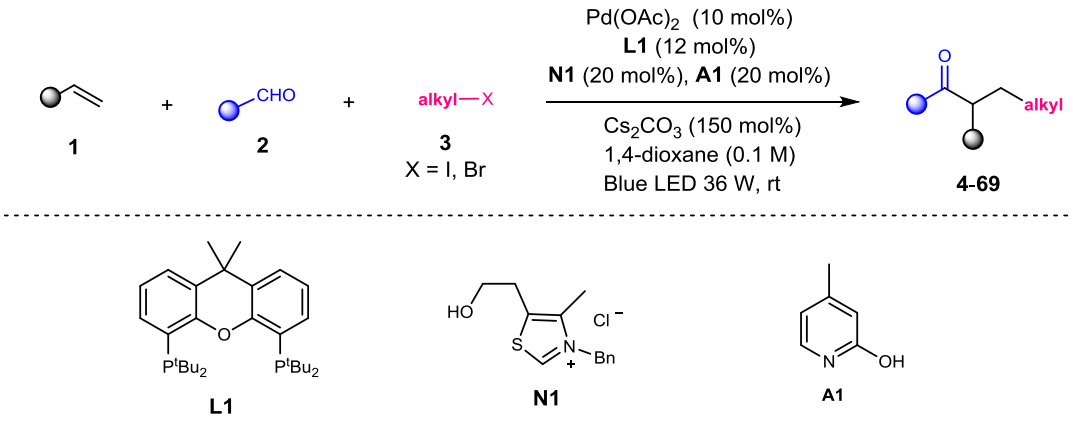

Typical procedure (Standard conditions). A 4 mL vial equipped with a stir bar was

charged with preNHC **N1** (10.8 mg, 0.04 mmol), $Pd(OAc)_2$ (4.5 mg, 0.02 mmol), ligand **L1** (11.9 mg, 0.024 mmol) and 1.0 mL of 1,4-dioxane. After stirring for 30 min in glove box, to the solution was added Cs_2CO_3 (97.8 mg, 0.3 mmol), additive **A1** (4.4 mg, 0.04 mmol), alkenes **1** (0.2 mmol), aldehydes **2** (0.4 mmol), alkyl halides **3** (0.3 mmol), and 1.0 mL of 1,4-dioxane. The reaction mixtrue was removed from the glove box and stirred under 36W blue LED lights at room temperature until the complete consumption of **1** (generally 48 hours) by TLC analysis. The reaction mixture was filtered through a small pad of silica and eluted with EtOAc. The solution was concentrated under reduced pressure, and purified by column chromatography on silica gel to afford the desired ketones.

For the reaction with secondary and tertiary haloalkanes and benzylic bromide, two two equivalent alkenes were used.

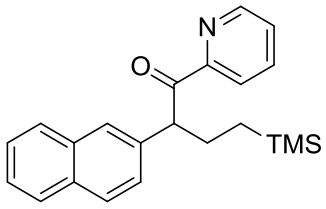

11122A

2-(naphthalen-2-yl)-1-(pyridin-2-yl)-4-(trimethylsilyl)butan-1-one (**4**)

Yield 54.0 mg, 78%. Light yellow oil, R_f = 0.44 (petroleum ether/ethyl acetate, 10:1).

^{1}H NMR (300 MHz, $CDCl_3$) δ 8.68 – 8.67 (m, 1H), 8.02 (d, *J* = 7.8 Hz, 1H), 7.86 – 7.68 (m, 5H), 7.57 (dd, *J* = 8.5, 1.8 Hz, 1H), 7.48 – 7.32 (m, 3H), 5.50 (t, *J* = 7.4 Hz, 1H), 2.31 – 2.18 (m, 1H), 2.04 – 1.91 (m, 1H), 0.62 – 0.41 (m, 2H), -0.02 (s, 9H).

^{13}C NMR (75 MHz, $CDCl_3$) δ 201.9, 153.4, 149.0, 137.0, 136.9, 133.6, 132.6, 128.2, 127.9, 127.7, 127.4, 127.0, 126.0, 125.7, 122.8, 54.2, 27.7, 15.0, -1.7.

IR (KBr) ν 2950, 1693, 1247, 859, 834, 743 696.

HRMS (ESI) *m/z*: Calc. For $C_{22}H_{26}ONSi$ ($[M+H]^+$) 348.1778, Found 348.1777.

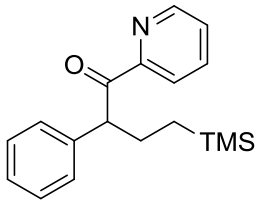

11122B

2-phenyl-1-(pyridin-2-yl)-4-(trimethylsilyl)butan-1-one (**5**)

Yield 50.5 mg, 85%. Light yellow oil, R_f = 0.48 (petroleum ether/ethyl acetate, 10:1).

^{1}H NMR (300 MHz, $CDCl_3$) δ 8.70 – 8.69 (m, 1H), 8.03 (d, *J* = 7.8 Hz, 1H), 7.78 (td, *J* = 7.7, 1.8 Hz, 1H), 7.44 – 7.40 (m, 3H), 7.32 – 7.27 (m, 2H), 7.23 – 7.20 (m, 1H), 5.37 (t, *J* = 7.5 Hz, 1H), 2.26 – 2.14 (m, 1H), 1.96 – 1.83 (m, 1H), 0.60 – 0.41 (m, 2H), -0.00 (s, 9H).

^{13}C NMR (75 MHz, $CDCl_3$) δ 202.0, 153.4, 149.0, 139.5, 136.9, 129.1, 128.6, 127.0, 126.8, 122.8, 54.0, 27.8, 14.9, -1.7.

IR (KBr) ν 2951, 1694, 1247, 860, 836, 756, 744, 701.

HRMS (ESI) *m/z*: Calc. For $C_{18}H_{24}ONSi$ ($[M+H]^+$) 298.1621, Found 298.1621.

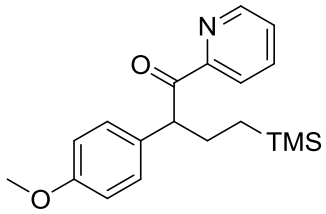

11122C

2-(4-methoxyphenyl)-1-(pyridin-2-yl)-4-(trimethylsilyl)butan-1-one (**6**)

Yield 46.7 mg, 71%. Light yellow oil, R_f = 0.31 (petroleum ether/ethyl acetate, 10:1).

^{1}H NMR (300 MHz, $CDCl_3$) δ 8.71 – 8.69 (m, 1H), 8.03 (d, *J* = 7.9 Hz, 1H), 7.79 (td, *J* = 7.7, 1.8 Hz, 1H), 7.44 – 7.40 (m, 1H), 7.34 (d, *J* = 8.7 Hz, 2H), 6.83 (d, *J* = 8.7 Hz, 2H), 5.31 (t, *J* = 7.5 Hz, 1H), 3.77 (s, 3H), 2.23 – 2.10 (m, 1H), 1.92 – 1.79 (m, 1H), 0.58 – 0.41 (m, 2H), -0.00 (s, 9H).

^{13}C NMR (75 MHz, $CDCl_3$) δ 202.1, 158.5, 153.5, 148.9, 136.9, 131.5, 130.1, 126.9, 122.8, 114.0, 55.3, 53.0, 27.6, 14.9, -1.7.

IR (KBr) ν 2951, 1693, 1501, 1249, 1177, 1036, 856, 836, 747.

HRMS (ESI) *m/z*: Calc. For $C_{19}H_{26}O_2NSi$ ($[M+H]^+$) 328.1727, Found 328.1726.

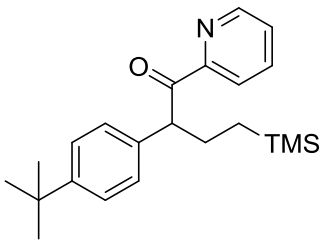

11122D

2-(4-(tert-butyl)phenyl)-1-(pyridin-2-yl)-4-(trimethylsilyl)butan-1-one (**7**)

Yield 39.5 mg, 56%. Light yellow viscous oil, R_f = 0.47 (petroleum ether/ethyl acetate, 10:1).

^{1}H NMR (300 MHz, $CDCl_3$) δ 8.72 (d, *J* = 4.8 Hz, 1H), 8.04 (d, *J* = 7.9 Hz, 1H), 7.79 (td, *J* = 7.7, 1.8 Hz, 1H), 7.45 – 7.41 (m, 1H), 7.36 – 7.28 (m, 4H), 5.39 (t, *J* = 7.5 Hz, 1H), 2.28 – 2.15 (m, 1H), 1.93 – 1.81 (m, 1H), 1.29 (s, 9H), 0.56 – 0.48 (m, 2H), 0.00 (s, 9H).

^{13}C NMR (75 MHz, $CDCl_3$) δ 202.2, 153.6, 149.5, 149.0, 136.9, 136.4, 128.6, 127.0, 125.5, 122.8, 53.3, 34.5, 31.5, 27.9, 15.1, -1.7.

IR (KBr) ν 2955, 1694, 1247, 857, 835.

HRMS (ESI) *m/z*: Calc. For $C_{21}H_{32}ONSi$ ($[M+H]^+$) 354.2248, Found 354.2247.

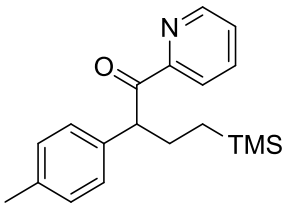

11132E

1-(pyridin-2-yl)-2-(p-tolyl)-4-(trimethylsilyl)butan-1-one (**8**)

Yield 37.1 mg, 60%. Light yellow oil, R_f = 0.44 (petroleum ether/ethyl acetate, 10:1).

^{1}H NMR (500 MHz, $CDCl_3$) δ 8.70 (d, *J* = 4.8 Hz, 1H), 8.03 (d, *J* = 7.9 Hz, 1H), 7.80 – 7.77 (m, 1H), 7.42 (t, *J* = 5.9 Hz, 1H), 7.32 – 7.29 (m, 2H), 7.10 (d, *J* = 7.8 Hz, 2H), 5.33 (t, *J* = 7.4 Hz, 1H), 2.30 (s, 3H), 2.22 – 2.14 (m, 1H), 1.91 – 1.85 (m, 1H), 0.57 – 0.45 (m, 2H), 0.00 (s, 9H).

^{13}C NMR (126 MHz, $CDCl_3$) δ 202.1, 153.5, 149.0, 136.9, 136.4, 129.3, 129.0, 126.9, 122.8, 53.6, 27.7, 21.2, 14.9, -1.7.

IR (KBr) ν 2951, 1694, 1247, 857, 837, 802, 746.

HRMS (ESI) *m/z*: Calc. For $C_{19}H_{26}NOSi$ ($[M+H]^+$) 312.1778, Found 312.1776.

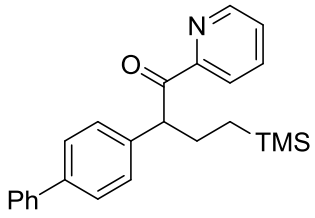

11132H

2-([1,1'-biphenyl]-4-yl)-1-(pyridin-2-yl)-4-(trimethylsilyl)butan-1-one (**9**)

Yield 58.9 mg, 79%. Light yellow oil, R_f = 0.38 (petroleum ether/ethyl acetate, 10:1).

^{1}H NMR (500 MHz, $CDCl_3$) δ 8.74 – 8.70 (m, 1H), 8.08 – 8.03 (m, 1H), 7.83 – 7.77 (m, 1H), 7.59 – 7.47 (m, 6H), 7.43 – 7.39 (m, 3H), 7.35 – 7.27 (m, 1H), 5.44 – 5.40 (m, 1H), 2.25 – 2.18 (m, 1H), 1.95 – 1.89 (m, 1H), 0.59 – 0.50 (m, 2H), 0.00 (s, 9H).

^{13}C NMR (126 MHz, $CDCl_3$) δ 201.9, 153.4, 149.0, 141.0, 139.7, 138.6, 137.0, 129.5, 128.8, 127.3, 127.2, 127.1, 127.0, 122.8, 53.6, 27.8, 15.0, -1.7.

IR (KBr) ν 2951, 1694, 1485, 1247, 857, 836, 762, 697.

HRMS (ESI) *m/z*: Calc. For $C_{24}H_{28}NOSi$ ($[M+H]^+$) 374.1935, Found 374.1932.

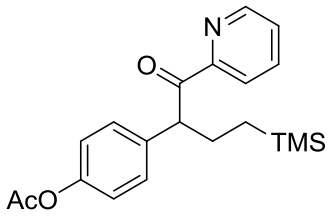

11134K

4-(1-oxo-1-(pyridin-2-yl)-4-(trimethylsilyl)butan-2-yl)phenyl acetate (10)

Yield 41.1 mg, 58%. Light yellow oil, R_f = 0.33 (petroleum ether/ethyl acetate, 5:1).

^{1}H NMR (400 MHz, $CDCl_3$) δ 8.69 (dd, *J* = 4.8, 1.8 Hz, 1H), 8.03 (d, *J* = 7.8 Hz, 1H), 7.79 (td, *J* = 7.7, 1.8 Hz, 1H), 7.45 – 7.41 (m, 3H), 7.01 – 6.99 (m, 2H), 5.39 (t, *J* = 7.5 Hz, 1H), 2.28 (s, 3H), 2.23 – 2.13 (m, 1H), 1.91 – 1.81 (m, 1H), 0.55 – 0.43 (m, 2H), -0.01 (s, 9H).

^{13}C NMR (126 MHz, $CDCl_3$) δ 201.8, 169.6, 153.2, 149.5, 149.0, 137.0, 130.1, 127.1, 122.8, 121.5, 53.2, 27.8, 21.3, 14.9, -1.7.

IR (KBr) ν 2951, 1764, 1694, 1504, 1369, 1167, 858, 837.

HRMS (ESI) *m/z*: Calc. For $C_{20}H_{26}NO_3Si$ ($[M+H]^+$) 356.1677, Found 356.1675.

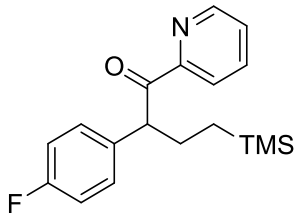

11132B

2-(4-fluorophenyl)-1-(pyridin-2-yl)-4-(trimethylsilyl)butan-1-one (**11**)

Yield 47.7 mg, 76%. Light yellow oil, R_f = 0.49 (petroleum ether/ethyl acetate, 10:1).

^{1}H NMR (500 MHz, $CDCl_3$) δ 8.70 – 8.69 (m, 1H), 8.03 (d, *J* = 7.9 Hz, 1H), 7.81 – 7.79 (m, 1H), 7.45 – 7.44 (m, 1H), 7.40 – 7.37 (m, 2H), 6.98 (t, *J* = 8.5 Hz, 2H), 5.35 (t, *J* = 7.5 Hz, 1H), 2.20 – 2.13 (m, 1H), 1.89 – 1.81 (m, 1H), 0.55 – 0.41 (m, 2H), 0.00 (s, 9H).

^{13}C NMR (126 MHz, $CDCl_3$) δ 201.9, 161.9 (d, J_{CF} = 245 Hz), 153.2, 149.0, 137.0, 135.1 (d, J_{CF} = 3 Hz), 130.6 (d, J_{CF} = 8 Hz), 127.1, 122.8, 115.4 (d, J_{CF} = 21 Hz), 53.0, 27.8, 14.8, -1.7.

IR (KBr) ν 2952, 1695, 1507, 1247, 1222, 857, 836, 747.

HRMS (ESI) *m/z*: Calc. For $C_{18}H_{23}NOFSi$ ($[M+H]^+$) 316.1528, Found 316.1526.

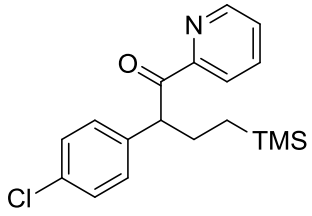

11134J

2-(4-chlorophenyl)-1-(pyridin-2-yl)-4-(trimethylsilyl)butan-1-one (12)

Yield 53.6 mg, 81%. Light yellow oil, R_f = 0.42 (petroleum ether/ethyl acetate, 10:1).

^{1}H NMR (400 MHz, $CDCl_3$) δ 8.69 (d, *J* = 4.7 Hz, 1H), 8.03 (d, *J* = 7.8 Hz, 1H), 7.81 (t, *J* = 7.7 Hz, 1H), 7.46 – 7.43 (m, 1H), 7.36 (d, *J* = 8.1 Hz, 2H), 7.26 (d, *J* = 8.1 Hz, 2H), 5.34 (t, *J* = 7.5 Hz, 1H), 2.21 – 2.11 (m, 1H), 1.90 – 1.81 (m, 1H), 0.56 – 0.40 (m, 2H), 0.00 (s, 9H).

^{13}C NMR (101 MHz, $CDCl_3$) δ 201.6, 153.1, 149.0, 138.0, 137.0, 132.7, 130.5, 128.7, 127.2, 122.8, 53.3, 27.7, 14.8, -1.7.

IR (KBr) ν 1695, 1490, 1247, 1092, 1024, 856, 836, 744.

HRMS (ESI) *m/z*: Calc. For $C_{18}H_{23}NOSiCl$ ($[M+H]^+$) 332.1232, Found 332.1228.

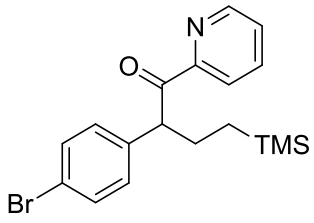

11132C

2-(4-bromophenyl)-1-(pyridin-2-yl)-4-(trimethylsilyl)butan-1-one (**13**)

Yield 65.3 mg, 87%. Light yellow oil, R_f = 0.38 (petroleum ether/ethyl acetate, 10:1).

^{1}H NMR (500 MHz, $CDCl_3$) δ 8.69 (d, *J* = 4.9 Hz, 1H), 8.03 (d, *J* = 8.0 Hz, 1H), 7.82 – 7.79 (m, 1H), 7.46 – 7.41 (m, 3H), 7.30 (d, *J* = 8.6 Hz, 2H), 5.33 (t, *J* = 7.5 Hz, 1H), 2.20 – 2.12 (m, 1H), 1.89 – 1.81 (m, 1H), 0.55 – 0.41 (m, 2H), 0.00 (s, 9H).

^{13}C NMR (126 MHz, $CDCl_3$) δ 201.5, 153.1, 149.0, 138.5, 137.0, 131.7, 130.9, 127.2, 122.8, 120.8, 53.4, 27.6, 14.8, -1.7.

IR (KBr) ν 2951, 1695, 1486, 1247, 1011, 857, 836, 744, 695.

HRMS (ESI) *m/z*: Calc. For $C_{18}H_{23}NOSiBr$ ($[M+H]^+$) 376.0727, Found 376.0723.

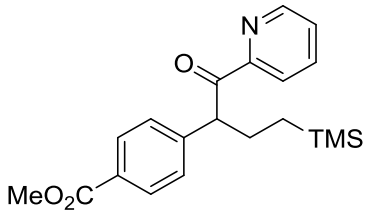

11134C

methyl 4-(1-oxo-1-(pyridin-2-yl)-4-(trimethylsilyl)butan-2-yl)benzoate (14)

Yield 62.5 mg, 88%. Light yellow oil , R_f = 0.22 (petroleum ether/ethyl acetate, 10:1).

^{1}H NMR (500 MHz, $CDCl_3$) δ 8.70 – 8.69 (m, 1H), 8.04 (d, *J* = 7.9 Hz, 1H), 7.97 (d, *J* = 8.2 Hz, 2H), 7.80 (td, *J* = 7.7, 1.7 Hz, 1H), 7.50 (d, *J* = 8.0 Hz, 2H), 7.45 – 7.43 (m, 1H), 5.43 (t, *J* = 7.4 Hz, 1H), 3.90 (s, 3H), 2.24 – 2.16 (m, 1H), 1.94 – 1.86 (m, 1H), 0.57 – 0.51 (m, 1H), 0.48 – 0.34 (m, 1H), 0.00 (s, 9H).

^{13}C NMR (126 MHz, $CDCl_3$) δ 201.3, 167.1, 153.1, 149.0, 144.9, 137.0, 129.9, 129.2, 128.7, 127.2, 122.8, 54.1, 52.1, 27.7, 14.8, -1.7.

IR (KBr) ν 2951, 1695, 1486, 1247, 1011, 857, 836, 744, 695.

HRMS (ESI) *m/z*: Calc. For $C_{20}H_{26}NO_3Si$ ($[M+H]^+$) 356.1676, Found 356.1682.

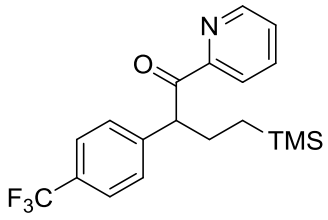

11122E

1-(pyridin-2-yl)-2-(4-(trifluoromethyl)phenyl)-4-(trimethylsilyl)butan-1-one (**15**)

Yield 53.5 mg, 73%. Light yellow oil, R_f = 0.51 (petroleum ether/ethyl acetate, 10:1).

^{1}H NMR (300 MHz, $CDCl_3$) δ 8.70 (d, *J* = 4.7 Hz, 1H), 8.05 (d, *J* = 7.8 Hz, 1H), 7.81 (td, *J* = 7.7, 1.7 Hz, 1H), 7.55 (brs, 4H), 7.47 – 7.43 (m, 1H), 5.45 (t, *J* = 7.5 Hz, 1H), 2.27 – 2.14 (m, 1H), 1.95 – 1.82 (m, 1H), 0.59 – 0.38 (m, 2H), 0.00 (s, 9H).

^{13}C NMR (126 MHz, $CDCl_3$) δ 201.3, 153.0, 149.0, 143.6, 137.1, 129.5, 129.2, 127.3, 125.5 (d, J_{CF} = 4 Hz), 124.3 (q, J_{CF} = 272 Hz), 122.8, 53.8, 27.8, 14.9, -1.7.

IR (KBr) ν 2953, 1697, 1325, 1248, 1165, 1125, 1068, 858, 837, 697.

HRMS (ESI) *m/z*: Calc. For $C_{19}H_{23}F_3ONSi$ ($[M+H]^+$) 366.1496, Found 366.1494.

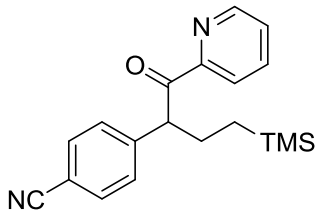

11140C

4-(1-oxo-1-(pyridin-2-yl)-4-(trimethylsilyl)butan-2-yl)benzonitrile (16)

Yield 49.9 mg, 77%. Light yellow oil , R_f = 0.18 (petroleum ether/ethyl acetate, 10:1).

^{1}H NMR (400 MHz, $CDCl_3$) δ 8.70 (d, *J* = 4.7 Hz, 1H), 8.05 (d, *J* = 7.8 Hz, 1H), 7.83 (t, *J* = 7.7 Hz, 1H), 7.57 (q, *J* = 8.2 Hz, 4H), 7.49 – 7.46 (m, 1H), 5.44 (t, *J* = 7.4 Hz, 1H), 2.24 – 2.15 (m, 1H), 1.92 – 1.82 (m, 1H), 0.52 (td, *J* = 13.6, 4.6 Hz, 1H), 0.40 (td, *J* = 13.9, 13.5, 4.4 Hz, 1H), 0.00 (s, 9H).

^{13}C NMR (101 MHz, $CDCl_3$) δ 200.9, 152.8, 149.1, 145.1, 137.1, 132.4, 129.9, 127.5, 122.8, 119.1, 110.7, 54.1, 27.8, 14.9, -1.7.

IR (KBr) ν 2952, 2228, 1696, 1247, 858, 837, 748, 562.

HRMS (ESI) *m/z*: Calc. For $C_{19}H_{23}N_2OSi$ ($[M+H]^+$) 323.1574, Found 323.1575.

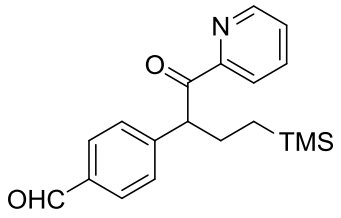

11134L

4-(1-oxo-1-(pyridin-2-yl)-4-(trimethylsilyl)butan-2-yl)benzaldehyde (17)

Yield 43.5 mg, 67%. Light yellow oil, R_f = 0.38 (petroleum ether/ethyl acetate, 5:1).

^{1}H NMR (400 MHz, CDCl3) δ 9.97 (s, 1H), 8.70 (d, *J* = 4.8 Hz, 1H), 8.05 (d, *J* = 7.9 Hz, 1H), 7.83 – 7.81 (m, 3H), 7.61 – 7.59 (m, 2H), 7.47 – 7.43 (m, 1H), 5.47 (t, *J* = 7.5 Hz, 1H), 2.25 – 2.17 (m, 1H), 1.95 – 1.87 (m, 1H), 0.58 – 0.40 (m, 2H), 0.00 (s, 9H).

^{13}C NMR (101 MHz, $CDCl_3$) δ 201.1, 192.1, 152.9, 149.1, 146.8, 137.1, 135.2, 130.0, 129.8, 127.3, 122.8, 54.3, 27.8, 14.9, -1.7.

IR (KBr) ν 1699, 1603, 1247, 1212, 1168, 857, 838, 694.

HRMS (ESI) *m/z*: Calc. For $C_{19}H_{24}NO_2Si$ ($[M+H]^+$) 326.1571, Found 326.1570.

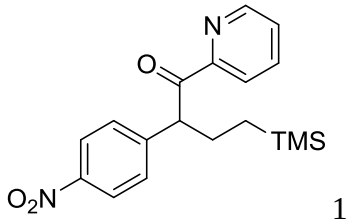

11122F

2-(4-nitrophenyl)-1-(pyridin-2-yl)-4-(trimethylsilyl)butan-1-one (**18**)

Yield 38.5 mg, 56%. Light yellow oil, R_f = 0.29 (petroleum ether/ethyl acetate, 10:1).

^{1}H NMR (300 MHz, $CDCl_3$) δ 8.70 – 8.69 (m, 1H), 8.16 (d, *J* = 8.8 Hz, 2H), 8.05 (d, *J* = 7.9 Hz, 1H), 7.83 (td, *J* = 7.7, 1.8 Hz, 1H), 7.60 (d, *J* = 8.8 Hz, 2H), 7.49 – 7.45 (m, 1H), 5.50 (t, *J* = 7.4 Hz, 1H), 2.28 – 2.15 (m, 1H), 1.96 – 1.82 (m, 1H), 0.59 – 0.35 (m, 2H), 0.00 (s, 9H).

^{13}C NMR (75 MHz, $CDCl_3$) δ 200.7, 152.7, 149.1, 147.3, 147.0, 137.2, 130.0, 127.5, 123.8, 122.8, 53.9, 27.9, 14.9, -1.7.

IR (KBr) ν 2952, 1696, 1502, 1346, 1248, 857, 837, 744, 707.

HRMS (ESI) *m/z*: Calc. For $C_{18}H_{23}O_3N_2Si$ ($[M+H]^+$) 343.1473, Found 343.1470.

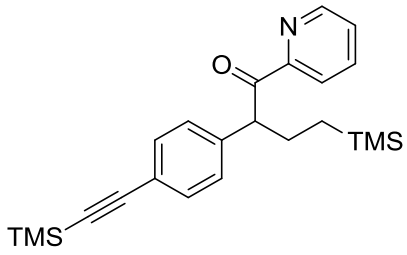

11140E

1-(pyridin-2-yl)-4-(trimethylsilyl)-2-(4-((trimethylsilyl)ethynyl)phenyl)butan-1-one (19)

Yield 53.9 mg, 68%. Light yellow oil , R_f = 0.38 (petroleum ether/ethyl acetate, 10:1).

^{1}H NMR (400 MHz, $CDCl_3$) δ 8.68 (d, *J* = 4.8 Hz, 1H), 8.02 (d, *J* = 7.9 Hz, 1H), 7.82 – 7.78 (m, 1H), 7.45 – 7.34 (m, 5H), 5.33 (t, *J* = 7.5 Hz, 1H), 2.21 – 2.12 (m, 1H), 1.92 – 1.82 (m, 1H), 0.56 – 0.38 (m, 2H), 0.25 (s, 9H), 0.00 (s, 9H).

^{13}C NMR (101 MHz, $CDCl_3$) δ 201.5, 153.2, 149.0, 140.1, 136.9, 132.2, 129.1, 127.1, 122.7, 121.5, 105.2, 94.2, 54.0, 27.5, 14.8, 0.1, -1.7.

IR (KBr) ν 2954, 2157, 1696, 1248, 862, 841, 759.

HRMS (ESI) *m/z*: Calc. For $C_{23}H_{32}NOSi_2$ ($[M+H]^+$) 394.2017, Found 394.2015.

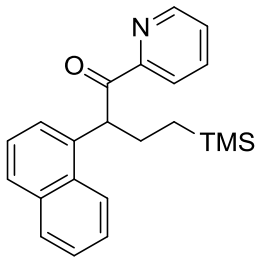

11134F

2-(naphthalen-1-yl)-1-(pyridin-2-yl)-4-(trimethylsilyl)butan-1-one (20)

Yield 42.5 mg, 61%. Light yellow oil , R_f = 0.49 (petroleum ether/ethyl acetate, 10:1).

^{1}H NMR (500 MHz, $CDCl_3$) δ 8.59 (s, 2H), 8.05 – 8.03 (m, 1H), 7.86 – 7.84 (m, 1H), 7.76 – 7.72 (m, 2H), 7.61 – 7.59 (m, 1H), 7.52 – 7.48 (m, 2H), 7.44 – 7.41 (m, 1H), 7.36 – 7.34 (m, 1H), 6.24 – 6.23 (m, 1H), 2.37 – 2.30 (m, 1H), 2.02 – 1.96 (m, 1H), 0.70 – 0.65 (m, 1H), 0.58 – 0.53 (m, 1H), 0.00 (s, 9H).

^{13}C NMR (126 MHz, $CDCl_3$) δ 202.4, 153.6, 149.0, 136.8, 136.3, 134.2, 132.3, 128.9, 127.4, 126.7, 126.1, 125.6, 125.5, 125.4, 124.4, 122.6, 49.0, 28.3, 15.4, -1.7.

IR (KBr) ν 2951, 1694, 1247, 859, 839, 795, 777.

HRMS (ESI) *m/z*: Calc. For $C_{22}H_{26}NOSi$ ($[M+H]^+$) 348.1778, Found 348.1776.

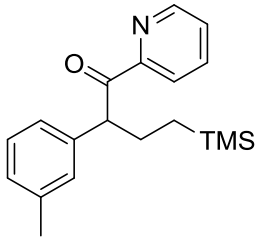

11134E

1-(pyridin-2-yl)-2-(m-tolyl)-4-(trimethylsilyl)butan-1-one (21)

Yield 26.7 mg, 43%. Light yellow oil , R_f = 0.54 (petroleum ether/ethyl acetate, 10:1).

^{1}H NMR (500 MHz, $CDCl_3$) δ 8.70 (d, *J* = 4.9 Hz, 1H), 8.03 (d, *J* = 7.9 Hz, 1H), 7.80 – 7.77 (m, 1H), 7.42 – 7.41 (m, 1H), 7.29 – 7,16 (m, 3H), 7.01 (d, *J* = 7.7 Hz, 1H), 5.36 – 5.32 (m, 1H), 2.33 (s, 3H), 2.27 – 2.16 (m, 1H), 1.90 – 1.84 (m, 1H), 0.57 – 0.44 (m, 2H), 0.00 (s, 9H).

^{13}C NMR (126 MHz, $CDCl_3$) δ 202.1, 153.5, 149.0, 139.4, 138.1, 136.9, 129.6, 128.4, 127.6, 127.0, 126.3, 122.8, 53.9, 27.9, 21.6, 15.0, -1.7.

IR (KBr) ν 2951, 1694, 1247, 861, 834, 777, 705.

HRMS (ESI) *m/z*: Calc. For $C_{19}H_{26}NOSi$ ($[M+H]^+$) 312.1778, Found 312.1780.

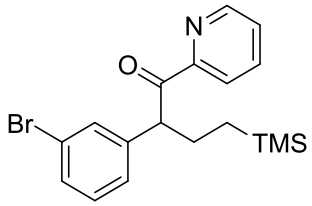

11132A

2-(3-bromophenyl)-1-(pyridin-2-yl)-4-(trimethylsilyl)butan-1-one (**22**)

Yield 50.7 mg, 67%. Light yellow oil , R_f = 0.43 (petroleum ether/ethyl acetate, 10:1).

^{1}H NMR (500 MHz, $CDCl_3$) δ 8.71 – 8.70 (m, 1H), 8.04 (d, *J* = 7.9 Hz, 1H), 7.81 (t, *J* = 7.8 Hz, 1H), 7.58 (brs, 1H), 7.46 – 7.43 (m, 1H), 7.36 – 7.32 (m, 2H), 7.16 (t, *J* = 7.8 Hz, 1H), 5.34 (t, *J* = 7.5 Hz, 1H), 2.19 – 2.14 (m, 1H), 1.88 – 1.84 (m, 1H), 0.55 – 0.41 (m, 2H), 0.00 (s, 9H).

^{13}C NMR (126 MHz, $CDCl_3$) δ 201.4, 153.0, 149.1, 141.9, 137.0, 132.1, 130.1, 130.0, 127.7, 127.2, 122.8, 122.6, 53.5, 27.8, 14.9, -1.7.

IR (KBr) ν 2951, 1695, 1566, 1247, 859, 837, 780, 689.

HRMS (ESI) *m/z*: Calc. For $C_{18}H_{23}ONBrSi$ ($[M+H]^+$) 376.0727, Found 376.0722.

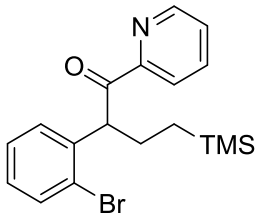

11134A

2-(2-bromophenyl)-1-(pyridin-2-yl)-4-(trimethylsilyl)butan-1-one (**23**)

Yield 52.2 mg, 69%. Light yellow oil , R_f = 0.40 (petroleum ether/ethyl acetate, 10:1).

^{1}H NMR ((500 MHz, $CDCl_3$) δ 8.68 (d, *J* = 4.8 Hz, 1H), 8.03 (d, *J* = 7.8 Hz, 1H), 7.79 (t, *J* = 7.7 Hz, 1H), 7.58 (d, *J* = 8.0 Hz, 1H), 7.42 – 7.40 (dd, *J* = 7.6, 4.6 Hz, 1H), 7.28 – 7.26 (m, 1H), 7.24 – 7.21 (m, 1H), 7.06 (t, *J* = 7.7 Hz, 1H), 5.77 (t, *J* = 7.2 Hz, 1H), 2.19 – 2.11 (m, 1H), 1.86 – 1.78 (m, 1H), 0.67 (td, *J* = 13.7, 4.5 Hz, 1H), 0.52 (td, *J* = 13.6, 4.5 Hz, 1H), 0.00 (s, 9H).

^{13}C NMR (126 MHz, $CDCl_3$) δ 201.7, 153.3, 149.2, 139.4, 136.8, 133.2, 129.1, 128.2, 127.6, 127.0, 126.0, 122.5, 53.4, 27.6, 14.9, -1.7.

IR (KBr) ν 2951, 1696, 1469, 1247, 1022, 858, 836, 749.

HRMS (ESI) *m/z*: Calc. For $C_{18}H_{23}NOSiBr$ ($[M+H]^+$) 376.0727, Found 376.0722.

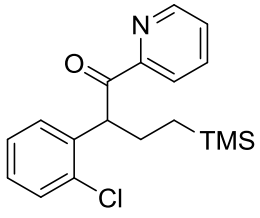

11132D

2-(2-chlorophenyl)-1-(pyridin-2-yl)-4-(trimethylsilyl)butan-1-one (**24**)

Yield 60.6 mg, 91%. Light yellow oil , R_f = 0.54 (petroleum ether/ethyl acetate, 10:1).

^{1}H NMR (500 MHz, $CDCl_3$) δ 8.68 (d, *J* = 4.7 Hz, 1H), 8.03 (d, *J* = 7.8 Hz, 1H), 7.79 (t, *J* = 7.7 Hz, 1H), 7.42 – 7.38 (m, 2H), 7.31 – 7.29 (m, 1H), 7.21 – 7.13 (m, 2H), 5.80 (t, *J* = 7.1 Hz, 1H), 2.20 – 2.13 (m, 1H), 1.87 – 1.80 (m, 1H), 0.68 – 0.62 (m, 1H), 0.55 – 0.49 (m, 1H), 0.00 (s, 9H).

^{13}C NMR (126 MHz, $CDCl_3$) δ 201.8, 153.3, 149.2, 137.7, 136.8, 135.0, 129.8, 129.1, 127.9, 127.0, 126.9, 122.5, 50.7, 27.4, 14.8, -1.7.

IR (KBr) ν 2951, 1696, 1473, 1435, 1247, 1036, 858, 837, 751.

HRMS (ESI) *m/z*: Calc. For $C_{18}H_{23}NOSiCl$ ($[M+H]^+$) 332.1232, Found 332.1229.

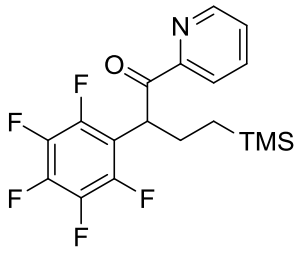

11134G

2-(perfluorophenyl)-1-(pyridin-2-yl)-4-(trimethylsilyl)butan-1-one (25)

Yield 59.7 mg, 77%. Light yellow oil , R_f = 0.42 (petroleum ether/ethyl acetate, 10:1).

^{1}H NMR (500 MHz, $CDCl_3$) δ 8.57 – 8.56 (m, 1H), 8.04 (d, *J* = 7.8 Hz, 1H), 7.82 (td, *J* = 7.7, 1.7 Hz, 1H), 7.44 – 7.41 (m, 1H), 5.25 – 5.22 (m, 1H), 2.31 – 2.24 (m, 1H), 1.92 – 1.84 (m, 1H), 0.59 (td, *J* = 13.6, 4.2 Hz, 1H), 0.38 (td, *J* = 13.7, 4.5 Hz, 1H), -0.00 (s, 9H).

^{13}C NMR (101 MHz, $CDCl_3$) δ ^{13}C NMR (126 MHz, $CDCl_3$) δ 197.7, 152.4, 148.9, 145.5(m), 139.8(m), 137.6(m), 137.1, 127.3, 122.8, 111.4(m), 46.4, 24.2, 14.7, -1.8.

IR (KBr) ν 2953, 1708, 1521, 1501, 1249, 1029, 990, 863, 836.

HRMS (ESI) *m/z*: Calc. For $C_{18}H_{19}NOF_5Si$ ($[M+H]^+$) 388.1151, Found 388.1146.

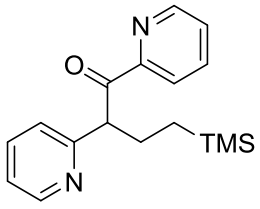

11122G

1,2-di(pyridin-2-yl)-4-(trimethylsilyl)butan-1-one (**26**)

Yield 38.5 mg, 65%. Light yellow oil, R_f = 0.15 (petroleum ether/ethyl acetate, 10:1).

1H NMR (400 MHz, $CDCl_3$) δ 8.66 (dd, J = 3.8, 1.0 Hz, 1H), 8.62 – 8.45 (m, 1H), 8.08 (dd, J = 7.9, 1.2 Hz, 1H), 7.80 (td, J = 7.7, 1.8 Hz, 1H), 7.71 – 7.57 (m, 1H), 7.55 – 7.36 (m, 2H), 7.10 (ddd, J = 7.8, 5.0, 1.3 Hz, 1H), 5.50 (t, J = 7.3 Hz, 1H), 2.26 (tdd, J = 13.4, 7.2, 4.5 Hz, 1H), 1.99 (tdd, J = 13.4, 7.4, 4.5 Hz, 1H), 0.62 (dd, J = 14.0, 4.4 Hz, 1H), 0.46 (ddd, J = 14.4, 12.9, 4.4 Hz, 1H), 0.00 (s, 9H).

^{13}C NMR (101 MHz, $CDCl_3$) δ 200.8, 159.7, 153.4, 149.5, 149.0, 136.8, 136.4, 126.8, 123.9, 122.7, 121.5, 56.9, 26.9, 14.9, -1.8.

IR (KBr) ν 2951, 1699, 1587, 1433, 1247, 858, 837, 746.

HRMS (ESI) *m/z*: Calc. For $C_{17}H_{23}ON_2Si$ ($[M+H]^+$) 299.1574, Found 299.1571.

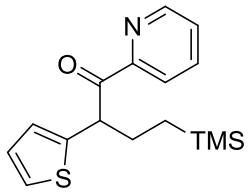

11134I

1-(pyridin-2-yl)-2-(thiophen-2-yl)-4-(trimethylsilyl)butan-1-one (27)

Yield 51.8 mg, 85%. Light yellow oil , R_f = 0.31 (petroleum ether/ethyl acetate, 10:1).

^{1}H NMR (500 MHz, $CDCl_3$) δ 8.73 (d, *J* = 4.5 Hz, 1H), 8.08 (d, *J* = 7.8 Hz, 1H), 7.83 (t, *J* = 7.7 Hz, 1H), 7.49 – 7.46 (m, 1H), 7.20 – 7.19 (m, 1H), 6.99 (s, 1H), 6.94 – 6.92 (m, 1H), 5.72 – 5.69 (m, 1H), 2.22 – 2.15 (m, 1H), 1.97 – 1.89 (m, 1H), 0.56 (t, *J* = 8.4 Hz, 2H), 0.00 (s, 9H).

^{13}C NMR (126 MHz, $CDCl_3$) δ 200.7, 152.9, 149.0, 142.1, 137.1, 127.3, 126.6, 126.1, 124.8, 123.0, 48.5, 29.1, 14.8, -1.7.

IR (KBr) ν 2951, 1697, 1247, 865, 835, 745, 697.

HRMS (ESI) *m/z*: Calc. For $C_{16}H_{22}NOSiS$ ($[M+H]^+$) 304.1186, Found 304.1186.

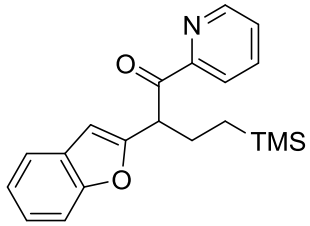

11140B

2-(benzofuran-2-yl)-1-(pyridin-2-yl)-4-(trimethylsilyl)butan-1-one (28)

Yield 56.5 mg, 84%. Light yellow oil, R_f = 0.21 (petroleum ether/ethyl acetate, 10:1).

^{1}H NMR (400 MHz, $CDCl_3$) δ 8.72 (d, *J* = 4.8 Hz, 1H), 8.09 (d, *J* = 7.8 Hz, 1H), 7.83 (td, *J* = 7.7, 1.8 Hz, 1H), 7.49 – 7.43 (m, 3H), 7.22 – 7.14 (m, 2H), 6.61 (s, 1H), 5.64 (t, *J* = 7.3 Hz, 1H), 2.34 – 2.04 (m, 2H), 0.61 – 0.57 (m, 2H), 0.00 (s, 9H).

^{13}C NMR (101 MHz, $CDCl_3$) δ 199.2, 156.2, 155.0, 152.9, 149.1, 137.1, 128.8, 127.4, 123.6, 122.8, 122.6, 120.7, 111.3, 104.6, 48.1, 25.7, 14.7, -1.7.

IR (KBr) ν 2927, 1693, 766, 743, 700.

HRMS (ESI) *m/z*: Calc. For $C_{20}H_{24}NO_2Si$ ($[M+H]^+$) 338.1571, Found 338.1567.

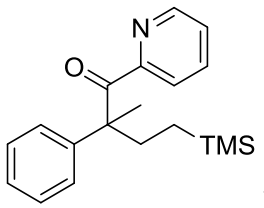

11134B

2-methyl-2-phenyl-1-(pyridin-2-yl)-4-(trimethylsilyl)butan-1-one (**29**)

Yield 44.3 mg, 71%. Light yellow oil, R_f = 0.45 (petroleum ether/ethyl acetate, 10:1).

^{1}H NMR (500 MHz, $CDCl_3$) δ 8.44 – 8.43 (m, 1H), 7.89 (d, *J* = 7.9 Hz, 1H), 7.75 (t, *J* = 7.8 Hz, 1H), 7.34 – 7.33 (m, 4H), 7.29 – 7.27 (m, 1H), 7.25 – 7.23 (m, 1H), 2.70 (td, *J* = 13.6, 4.0 Hz, 1H), 2.08 (td, *J* = 13.7, 4.2 Hz, 1H), 1.76 (s, 3H), 0.44 (td, *J* = 13.8, 4.2 Hz, 1H), 0.29 (td, *J* = 13.9, 4.0 Hz, 1H), 0.00 (s, 9H).

^{13}C NMR 204.0, 154.2, 148.2, 145.2, 136.3, 128.3, 126.5, 126.0, 125.7, 123.7, 55.8, 33.0, 23.8, 10.8, -1.8.

IR (KBr) ν 2951, 1686, 1246, 973, 836, 758, 744, 699.

HRMS (ESI) *m/z*: Calc. For $C_{19}H_{26}NOSi$ ($[M+H]^+$) 312.1778, Found 312.1777.

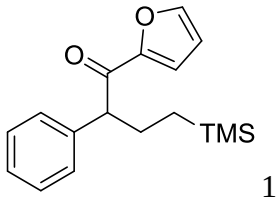

11148B

1-(furan-2-yl)-2-phenyl-4-(trimethylsilyl)butan-1-one (30)

Yield 29.6 mg, 52%. Colorless oil , R_f = 0.32 (petroleum ether/ethyl acetate, 20:1).

^{1}H NMR (400 MHz, $CDCl_3$) δ 7.57 (s, 1H), 7.38 – 7.20 (m, 6H), 6.50 (s, 1H), 4.33 (t, *J* = 7.3 Hz, 1H), 2.25 – 2.15 (m, 1H), 1.88 – 1.79 (m, 1H), 0.58 – 0.39 (m, 2H), 0.00 (s, 9H).

^{13}C NMR (101 MHz, $CDCl_3$) δ 189.6, 152.9, 146.5, 139.4, 128.8, 128.5, 127.2, 117.8, 112.3, 57.3, 27.9, 15.0, -1.7

IR (KBr) ν 2951, 1673, 1565, 1466, 1248, 1030, 859, 836, 759, 698.

HRMS (ESI) *m/z*: Calc. For $C_{17}H_{23}O_2Si$ ($[M+H]^+$) 287.1462, Found 287.1462.

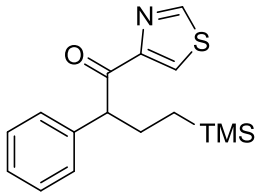

11148J

2-phenyl-1-(thiazol-4-yl)-4-(trimethylsilyl)butan-1-one (31)

Yield 38.9 mg, 64%. Colorless Oil, R_f = 0.50 (petroleum ether/ethyl acetate, 20:1).

^{1}H NMR (400 MHz, $CDCl_3$) δ 8.80 (d, *J* = 2.1 Hz, 1H), 8.21 (d, *J* = 2.1 Hz, 1H), 7.42 – 7.41 (m, 2H), 7.33 – 7.29 (m, 2H), 7.24 – 7.23 (m, 1H), 4.95 (t, *J* = 7.4 Hz, 1H), 2.27 – 2.18 (m, 1H), 1.92 – 1.82 (m, 1H), 0.59 – 0.43 (m, 2H), 0.00 (s, 9H).

^{13}C NMR (101 MHz, $CDCl_3$) δ 195.4, 156.0, 152.6, 139.2, 129.0, 128.7, 127.0, 125.9, 57.9, 27.8, 14.9, -1.7.

IR (KBr) ν 2950, 1685, 1476, 1418, 1247, 858, 836, 740, 697.

HRMS (ESI) *m/z*: Calc. For $C_{16}H_{22}NOSiS$ ($[M+H]^+$) 304.1186, Found 304.1184.

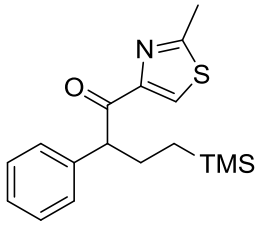

11148G

1-(2-methylthiazol-4-yl)-2-phenyl-4-(trimethylsilyl)butan-1-one (32)

Yield 42.2 mg, 66%. Colorless oil, R_f = 0.49 (petroleum ether/ethyl acetate, 10:1).

^{1}H NMR (400 MHz, $CDCl_3$) δ 7.99 (s, 1H), 7.42 (d, *J* = 7.6 Hz, 2H), 7.33 – 7.29 (m, 2H), 7.25 – 7.23 (m, 1H), 4.86 (t, *J* = 7.4 Hz, 1H), 2.76 (s, 3H), 2.26 – 2.16 (m, 1H), 1.90 – 1.80 (m, 1H), 0.59 – 0.41 (m, 2H), 0.00 (s, 9H).

^{13}C NMR (101 MHz, $CDCl_3$) δ 195.3, 165.8, 154.9, 139.4, 129.0, 128.6, 127.0, 126.1, 57.7, 27.8, 19.5, 14.9, -1.7.

IR (KBr) ν 2951, 1684, 1476, 1247, 1155, 856, 836, 740, 697.

HRMS (ESI) *m/z*: Calc. For $C_{17}H_{24}NOSiS$ ($[M+H]^+$) 318.1342, Found 318.1342.

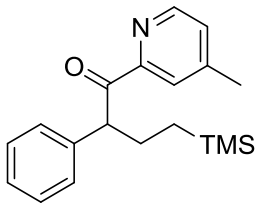

11148F

1-(4-methylpyridin-2-yl)-2-phenyl-4-(trimethylsilyl)butan-1-one (33)

Yield 37.4 mg, 60%. Colorless oil , R_f = 0.27 (petroleum ether/ethyl acetate, 20:1).

^{1}H NMR (400 MHz, $CDCl_3$) δ 8.55 (d, *J* = 4.9 Hz, 1H), 7.85 (s, 1H), 7.42 (d, *J* = 7.7 Hz, 2H), 7.30 – 7.18 (m, 4H), 5.37 (t, *J* = 7.5 Hz, 1H), 2.39 (s, 3H), 2.24 – 2.15 (m, 1H), 1.93 – 1.84 (m, 1H), 0.58 – 0.43 (m, 2H), 0.00 (s, 9H).

^{13}C NMR (101 MHz, $CDCl_3$) δ 202.3, 153.4, 148.8, 148.2, 139.6, 129.1, 128.5, 127.9, 126.8, 123.6, 54.0, 27.7, 21.1, 14.9, -1.7.

IR (KBr) ν 2951, 1693, 1599, 1247, 1169, 1034, 859, 834, 748, 702.

HRMS (ESI) *m/z*: Calc. For $C_{19}H_{26}NOSi$ ($[M+H]^+$) 312.1778, Found 312.1777.

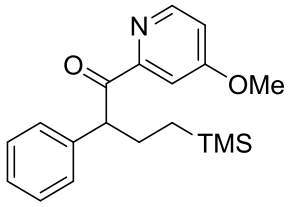

11148I

1-(4-methoxypyridin-2-yl)-2-phenyl-4-(trimethylsilyl)butan-1-one (34)

Yield 39.7 mg, 61%. Colorless oil , R_f = 0.44 (petroleum ether/ethyl acetate, 10:1).

^{1}H NMR (400 MHz, $CDCl_3$) δ 8.50 (d, *J* = 5.7 Hz, 1H), 7.56 (d, *J* = 2.5 Hz, 1H), 7.42 (d, *J* = 7.6 Hz, 2H), 7.29 (t, *J* = 7.5 Hz, 2H), 7.22 – 7.20 (m, 1H), 6.94 – 6.93 (m, 1H), 5.37 (t, *J* = 7.5 Hz, 1H), 3.88 (s, 3H), 2.24 – 2.14 (m, 1H), 1.93 – 1.83 (m, 1H), 0.57 – 0.44 (m, 2H), 0.00 (s, 9H).

^{13}C NMR (101 MHz, $CDCl_3$) δ 202.0, 166.6, 155.4, 150.1, 139.5, 129.1, 128.6, 126.8, 113.9, 107.8, 55.5, 54.0, 27.8, 14.9, -1.7.

IR (KBr) ν 2950, 1694, 1591, 1474, 1304, 1249, 1034, 860, 836, 747, 699.

HRMS (ESI) *m/z*: Calc. For $C_{19}H_{26}NO_2Si$ ($[M+H]^+$) 328.1727, Found 328.1726.

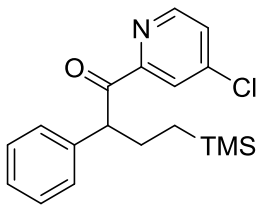

11148H

1-(4-chloropyridin-2-yl)-2-phenyl-4-(trimethylsilyl)butan-1-one (35)

Yield 26.9 mg, 41%. Colorless oil , R_f = 0.50 (petroleum ether/ethyl acetate, 20:1).

^{1}H NMR (400 MHz, $CDCl_3$) δ 8.58 (d, *J* = 5.2 Hz, 1H), 8.01 (m, 1H), 7.43 – 7.38 (m, 3H), 7.31 – 7.27 (m, 2H), 7.22 – 7.19 (m, 1H), 5.29 (t, *J* = 7.4 Hz, 1H), 2.23 – 2.14 (m, 1H), 1.93 – 1.83 (m, 1H), 0.57 – 0.41 (m, 2H), 0.00 (s, 9H).

^{13}C NMR (101 MHz, $CDCl_3$) δ 200.7, 154.6, 149.9, 145.5, 139.0, 129.1, 128.7, 127.1, 127.0, 123.2, 54.3, 27.7, 14.9, -1.7.

IR (KBr) ν 2951, 1696, 1569, 1247, 1216, 837, 727, 697.

HRMS (ESI) *m/z*: Calc. For $C_{18}H_{23}NOSiCl$ ($[M+H]^+$) 332.1232, Found 332.1231.

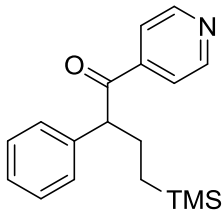

11178A

2-phenyl-1-(pyridin-4-yl)-4-(trimethylsilyl)butan-1-one (36)

Yield 47.2 mg, 79%. Colorless oil, R_f = 0.18 (petroleum ether/ethyl acetate, 5:1).

^{1}H NMR (400 MHz, $CDCl_3$) δ 8.74 – 8.73 (m, 2H), 7.71 – 7.70 (m, 2H), 7.34 – 7.32 (m, 2H), 7.29 – 7.26 (m, 3H), 4.41 (t, *J* = 7.1 Hz, 1H), 2.24 – 2.14 (m, 1H), 1.87 – 1.79 (m, 1H), 0.53 (td, *J* = 13.6, 4.4 Hz, 1H), 0.42 (td, *J* = 13.6, 4.5 Hz, 1H), 0.00 (s, 9H).

^{13}C NMR (101 MHz, $CDCl_3$) δ 199.9, 150.9, 143.2, 138.6, 129.3, 128.4, 127.5, 121.7, 57.9, 28.4, 14.9, -1.7.

IR (KBr) ν 2951, 1692, 1407, 1246, 1219, 860, 836, 700.

HRMS (APCI) *m/z*: Calc. For $C_{18}H_{22}NOSi$ ($[M-H]^-$) 296.1476, Found 296.1477.

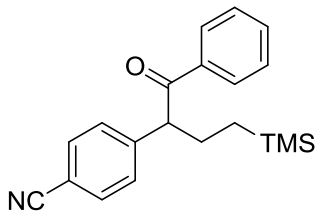

11157C

4-(1-oxo-1-phenyl-4-(trimethylsilyl)butan-2-yl)benzonitrile (37)

Yield 35.6 mg, 55%. Light yellow oil , R_f = 0.20 (petroleum ether/ethyl acetate, 20:1).

^{1}H NMR (500 MHz, $CDCl_3$) δ 7.96 (d, *J* = 7.8 Hz, 2H), 7.62 (d, *J* = 8.2 Hz, 2H), 7.58 – 7.55 (m, 1H), 7.48 – 7.44 (m, 4H), 4.59 (t, *J* = 7.2 Hz, 1H), 2.24 – 2.17 (m, 1H),

1.86 – 1.79 (m, 1H), 0.56 – 0.50 (m, 1H), 0.41 – 0.36 (m, 1H), 0.00 (d, *J* = 2.1 Hz, 9H).

^{13}C NMR (126 MHz, $CDCl_3$) δ 199.4, 145.1, 136.8, 133.5, 132.7, 129.3, 128.9, 128.7, 118.8, 111.1, 56.9, 29.0, 15.1, -1.8.

IR (KBr) ν 2951, 2228, 1681, 1247, 855, 836, 692.

HRMS (EI) *m/z*: Calc. For $C_{20}H_{23}NOSi$ ($[M]^+$) 321.1543, Found 321.1546.

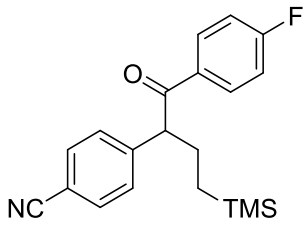

11168A

4-(1-(4-fluorophenyl)-1-oxo-4-(trimethylsilyl)butan-2-yl)benzonitrile (38)

Yield 35.0 mg, 52%. Colorless oil, R_f = 0.24 (petroleum ether/ethyl acetate, 20:1).

^{1}H NMR (500 MHz, $CDCl_3$) δ 8.00 – 7.97 (m, 2H), 7.63 (d, *J* = 8.3 Hz, 2H), 7.45 (d, *J* = 8.3 Hz, 2H), 7.12 (t, *J* = 8.6 Hz, 2H), 4.53 (t, *J* = 7.2 Hz, 1H), 2.23 – 2.16 (m, 1H), 1.85 – 1.78 (m, 1H), 0.55 – 0.49 (m, 1H), 0.41 – 0.35 (m, 1H), 0.00 (s, 9H).

^{13}C NMR (126 MHz, $CDCl_3$) δ 197.8, 165.9 (d, J_{CF} = 256 Hz), 144.9, 133.2 (d, J_{CF} = 3 Hz), 132.8, 131.3 (d, J_{CF} = 9 Hz), 129.2, 118.8, 116.0 (d, J_{CF} = 22 Hz), 111.3, 56.9, 29.0, 15.1, -1.8.

IR (KBr) ν 2951, 2228, 1682, 1597, 1504, 1246, 1156, 836.

HRMS (APCI) *m/z*: Calc. For $C_{20}H_{21}NOFSi$ ($[M-H]^-$) 338.1382, Found 338.1382.

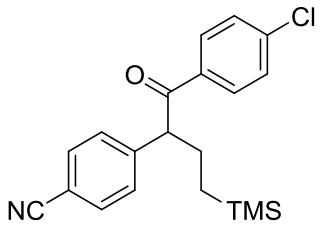

11172C

4-(1-(4-chlorophenyl)-1-oxo-4-(trimethylsilyl)butan-2-yl)benzonitrile (39)

Yield 49.3 mg, 69%. Colorless oil, R_f = 0.23 (petroleum ether/ethyl acetate, 20:1).

^{1}H NMR (500 MHz, $CDCl_3$) δ 7.90 – 7.88 (m, 2H), 7.64 – 7.62 (m, 2H), 7.45 – 7.42 (m, 4H), 4.53 – 4.51 (m, 1H), 2.23 – 2.15 (m, 1H), 1.85 – 1.78 (m, 1H), 0.55 – 0.49 (m, 1H), 0.41 – 0.34 (m, 1H), 0.00 (s, 9H).

^{13}C NMR (126 MHz, $CDCl_3$) δ 198.2, 144.8, 140.0, 135.1, 132.8, 130.1, 129.22, 129.20, 118.7, 111.3, 57.0, 28.9, 15.1, -1.8.

IR (KBr) ν 2952, 2228, 1683, 1588, 1247, 1093, 837.

HRMS (APCI) *m/z*: Calc. For $C_{20}H_{21}NOClSi$ ($[M-H]^-$) 354.1086, Found 354.1087.

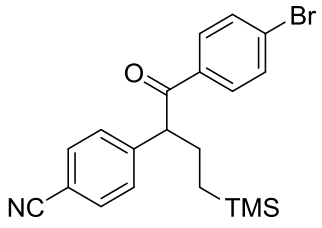

11168G

4-(1-(4-bromophenyl)-1-oxo-4-(trimethylsilyl)butan-2-yl)benzonitrile (40)

Yield 51.8 mg, 65%. Colorless oil, R_f = 0.26 (petroleum ether/ethyl acetate, 20:1).

^{1}H NMR (500 MHz, $CDCl_3$) δ 7.81 (dd, *J* = 8.4, 3.2 Hz, 2H), 7.64 – 7.58 (M, 4H), 7.44 (dd, *J* = 8.3, 3.3 Hz, 2H), 4.53 – 4.50 (m, 1H), 2.22 – 2.15 (m, 1H), 1.85 – 1.77 (m, 1H), 0.52 (td, *J* = 13.7, 4.1 Hz, 1H), 0.37 (td, *J* = 13.6, 4.1 Hz, 1H), 0.00 (m, 9H).

^{13}C NMR (126 MHz, $CDCl_3$) δ 198.4, 144.8, 135.5, 132.8, 132.2, 130.1, 129.2, 128.8, 118.7, 111.3, 56.9, 28.9, 15.1, -1.8.

IR (KBr) ν 2951, 2228, 1682, 1584, 1247, 1070, 836.

HRMS (APCI) *m/z*: Calc. For $C_{20}H_{21}NOBrSi$ ($[M-H]^-$) 398.0581, Found 398.0585.

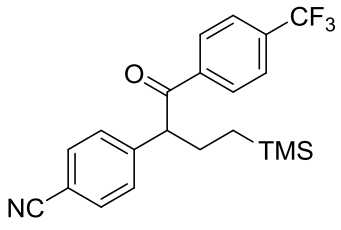

11157A

4-(1-oxo-1-(3-(trifluoromethyl)phenyl)-4-(trimethylsilyl)butan-2-yl)benzonitrile (41)

Yield 65.8 mg, 84%. Light yellow oil, R_f = 0.28 (petroleum ether/ethyl acetate, 20:1).

^{1}H NMR (500 MHz, $CDCl_3$) δ 8.04 (d, *J* = 8.1 Hz, 2H), 7.71 (d, *J* = 8.1 Hz, 2H), 7.64 (d, *J* = 8.0 Hz, 2H), 7.44 (d, *J* = 8.0 Hz, 2H), 4.56 (t, *J* = 7.1 Hz, 1H), 2.24 – 2.17 (m, 1H), 1.87 – 1.80 (m, 1H), 0.52 (td, *J* = 13.7, 4.3 Hz, 1H), 0.38 (td, *J* = 13.6, 4.3 Hz, 1H), 0.00 (s, 9H).

^{13}C NMR (126 MHz, $CDCl_3$) δ 198.5, 144.4, 139.4, 134.7 (qf, J_{CF} = 33 Hz), 132.9, 129.2, 129.0, 126.0 (q, J_{CF} J = 4 Hz), 123.4 (q, J_{CF} = 273 Hz), 118.6, 111.5, 57.4, 28.9, 15.1, -1.8.

IR (KBr) ν 2229, 1689, 1325, 1170, 1132, 1067, 837.

HRMS (ESI) *m/z*: Calc. For $C_{21}H_{21}NF_3OSi$ ($[M-H]^-$) 388.1350, Found 388.1358.

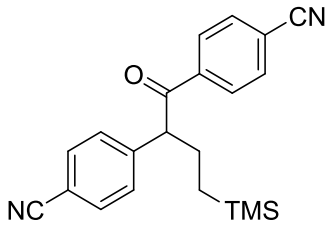

11172D

4,4'-(1-oxo-4-(trimethylsilyl)butane-1,2-diyl)dibenzonitrile (42)

Yield 66.6 mg, 96%. Colorless oil, R_f = 0.19 (petroleum ether/ethyl acetate, 5:1).

^{1}H NMR (500 MHz, $CDCl_3$) δ 8.08 – 8.01 (m, 2H), 7.83 – 7.74 (m, 2H), 7.65 – 7.64 (m, 2H), 7.44 – 7.42 (m, 2H), 4.54 – 4.51 (m, 1H), 2.23 – 2.17 (m, 1H), 1.86 – 1.80 (m, 1H), 0.52 (td, J = 13.7, 3.6 Hz, 1H), 0.38 (td, J = 13.7, 3.1 Hz, 1H), 0.00 (s, 9H).

^{13}C NMR (126 MHz, $CDCl_3$) δ 198.2, 144.1, 139.7, 132.9, 132.7, 129.2, 129.0, 118.6, 117.8, 116.7, 111.6, 57.4, 28.9, 15.0, -1.8.

IR (KBr) ν 2229, 1688, 1404, 1246, 1216, 836.

HRMS (APCI) *m/z*: Calc. For $C_{21}H_{21}N_2OSi$ ($[M-H]^-$) 345.1429, Found 345.1431.

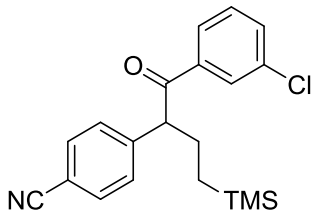

11168B

4-(1-(3-chlorophenyl)-1-oxo-4-(trimethylsilyl)butan-2-yl)benzonitrile (43)

Yield 53.6 mg, 75%. Light yellow oil, R_f = 0.21 (petroleum ether/ethyl acetate, 20:1).

^{1}H NMR (500 MHz, $CDCl_3$) δ 7.92 (s, 1H), 7.81 (dt, J = 7.8 Hz, 1H), 7.64 (d, J = 8.1 Hz, 2H), 7.54 – 7.52 (m, 1H), 7.45 (d, J = 8.1 Hz, 2H), 7.39 (t, J = 7.9 Hz, 1H), 4.52 (t, J = 7.1 Hz, 1H), 2.22 – 2.15 (m, 1H), 1.85 – 1.77 (m, 1H), 0.52 (td, J = 13.7, 4.3 Hz, 1H), 0.38 (td, J = 13.6, 4.2 Hz, 1H), 0.00 (s, 9H).

^{13}C NMR (126 MHz, $CDCl_3$) δ 198.2, 144.6, 138.3, 135.3, 133.4, 132.9, 130.2, 129.2, 128.8, 126.7, 118.7, 111.4, 57.1, 29.0, 15.1, -1.8.

IR (KBr) ν 2951, 2228, 1685, 1247, 1214, 856, 837, 752.

HRMS (APCI) *m/z*: Calc. For $C_{20}H_{21}NOClSi$ ($[M-H]^-$) 354.1086, Found 354.1089.

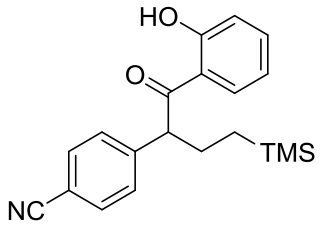

11157B

4-(1-(2-hydroxyphenyl)-1-oxo-4-(trimethylsilyl)butan-2-yl)benzonitrile (44)

Yield 35.7 mg, 53%. Colorless oil, R_f = 0.20 (petroleum ether/ethyl acetate, 20:1).

^{1}H NMR (400 MHz, $CDCl_3$) δ 12.33 (s, 1H), 7.78 (d, *J* = 8.1 Hz, 1H), 7.64 (d, *J* = 7.9 Hz, 2H), 7.48 – 7.43 (m, 3H), 7.00 (d, *J* = 8.4 Hz, 1H), 6.93 – 6.86 (m, 1H), 4.61 (t, *J* = 7.2 Hz, 1H), 2.23 – 2.16 (m, 1H), 1.88 – 1.78 (m, 1H), 0.53 (td, *J* = 13.6, 4.3 Hz, 1H), 0.39 (td, *J* = 13.5, 4.3 Hz, 1H), -0.02 (s, 9H).

^{13}C NMR (101 MHz, $CDCl_3$) δ 205.3, 163.4, 144.7, 136.9, 132.8, 130.0, 129.6, 129.1, 119.2, 119.1, 118.7, 111.5, 56.1, 28.8, 15.1, -1.8.

IR (KBr) ν 2951, 2228, 1636, 1605, 1446, 1247, 859, 837, 755.

HRMS (ESI) *m/z*: Calc. For $C_{20}H_{22}NO_2Si$ ($[M-H]^-$) 336.1425, Found 336.1433.

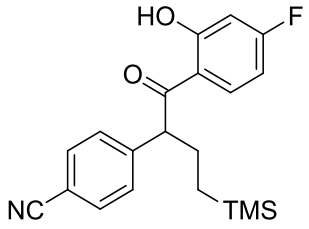

11172A

4-(1-(4-fluoro-2-hydroxyphenyl)-1-oxo-4-(trimethylsilyl)butan-2-yl)benzonitrile (45)

Yield 50.1 mg, 70%. Colorless oil, R_f = 0.17 (petroleum ether/ethyl acetate, 20:1).

^{1}H NMR (500 MHz, $CDCl_3$) δ 12.66 (s, 1H), 7.79 (dd, *J* = 9.0, 6.2 Hz, 1H), 7.65 (d, *J* = 8.2 Hz, 2H), 7.46 (d, *J* = 8.0 Hz, 2H), 6.68 – 6.66 (m, 1H), 6.59 (td, *J* = 8.5, 2.6 Hz, 1H), 4.52 (t, *J* = 7.1 Hz, 1H), 2.23 – 2.15 (m, 1H), 1.86 – 1.79 (m, 1H), 0.51 (td, *J* = 13.7, 4.3 Hz, 1H), 0.38 (td, *J* = 13.6, 4.3 Hz, 1H), 0.00 (s, 9H).

^{13}C NMR (126 MHz, $CDCl_3$) δ 204.2, 167.6 (d, J_{CF} = 258 Hz), 166.1 (d, J_{CF} = 14 Hz), 144.5, 132.9, 132.4 (d, J_{CF} J = 12 Hz), 129.1, 118.6, 116.4, 111.6, 107.6 (d, J_{CF} = 23 Hz), 105.6 (d, J_{CF} = 24 Hz), 56.3, 28.8, 15.1, -1.8.

IR (KBr) ν 2952, 2228, 1638, 1603, 1503, 1247, 1120, 855, 837.

HRMS (APCI) *m/z*: Calc. For $C_{20}H_{21}NO_2FSi$ ([M-H]$^-$) 354.1331, Found 354.1332.

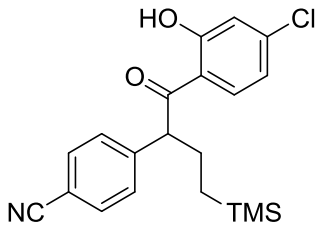

11172B

4-(1-(4-chloro-2-hydroxyphenyl)-1-oxo-4-(trimethylsilyl)butan-2-yl)benzonitrile (46)

Yield 53.3 mg, 72%. Colorless oil, R_f = 0.17 (petroleum ether/ethyl acetate, 20:1).

^{1}H NMR (500 MHz, $CDCl_3$) δ 12.45 (d, *J* = 4.2 Hz, 1H), 7.69 (dd, *J* = 8.7, 4.1 Hz, 1H), 7.66 – 7.63 (m, 2H), 7.46 – 7.43 (m, 2H), 7.01 – 7.00 (m, 1H), 6.86 – 6.84 (m, 1H), 4.54 – 4.51 (m, 1H), 2.22 – 2.15 (m, 1H), 1.85 – 1.78 (m, 1H), 0.53 – 0.48 (m, 1H), 0.41 – 0.37 (m, 1H), 0.00 (s, 9H).

^{13}C NMR (126 MHz, $CDCl_3$) δ 204.6, 164.1, 144.4, 142.8, 132.9, 131.0, 129.1, 119.9, 119.1, 118.6, 117.7, 111.6, 56.4, 28.8, 15.1, -1.8.

IR (KBr) ν 2952, 2229, 1637, 1605. 1487, 1409, 1247, 1203, 858, 837.

HRMS (APCI) *m/z*: Calc. For $C_{20}H_{21}NO_2ClSi$ ([M-H]$^-$) 370.1036, Found 370.1038.

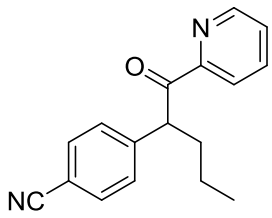

11154A

4-(1-oxo-1-(pyridin-2-yl)pentan-2-yl)benzonitrile (47)

Yield 30.5 mg, 58%. Light yellow oil, R_f = 0.17 (petroleum ether/ethyl acetate, 10:1).

^{1}H NMR (500 MHz, $CDCl_3$) δ 8.66 (d, *J* = 4.8 Hz, 1H), 8.01 (d, *J* = 7.9 Hz, 1H), 7.80 (t, *J* = 7.7 Hz, 1H), 7.56 – 7.43 (m, 5H), 5.49 (t, *J* = 7.6 Hz, 1H), 2.19 – 2.12 (m, 1H), 1.88 – 1.81 (m, 1H), 1.32 – 1.26 (m, 2H), 0.92 (t, *J* = 7.3 Hz, 3H).

^{13}C NMR (126 MHz, $CDCl_3$) δ 200.6, 152.7, 149.1, 145.3, 137.1, 132.3, 129.9, 127.5, 122.9, 119.1, 110.7, 50.6, 35.2, 20.9, 14.1.

IR (KBr) ν 2958, 2227, 1696, 1330, 995, 747, 563.

HRMS (ESI) *m/z*: Calc. For $C_{17}H_{17}N_2O$ ($[M+H]^+$) 265.1335, Found 265.1336.

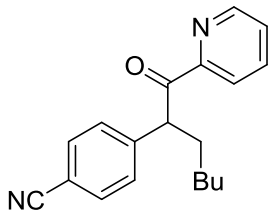

11154B

4-(1-oxo-1-(pyridin-2-yl)heptan-2-yl)benzonitrile (48)

Yield 34.3 mg, 59%. Light yellow oil, R_f = 0.22 (petroleum ether/ethyl acetate, 10:1).

^{1}H NMR (500 MHz, $CDCl_3$) δ 8.67 – 8.66 (m, 1H), 8.01 (d, *J* = 7.9 Hz, 1H), 7.80 (td, *J* = 7.7, 1.8 Hz, 1H), 7.56 – 7.51 (m, 4H), 7.45 – 7.43 (m, 1H), 5.46 (t, *J* = 7.5 Hz, 1H), 2.17 – 2.14 (m, 1H), 1.89 – 1.84 (m, 1H), 1.28 – 1.26 (m, 6H), 0.85 – 0.83 (m, 3H).

^{13}C NMR (126 MHz, $CDCl_3$) δ 200.7, 152.7, 149.1, 145.3, 137.1, 132.3, 129.9, 127.5, 122.9, 119.0, 110.7, 50.9, 33.0, 31.8, 27.4, 22.5, 14.1.

IR (KBr) ν 2955, 2227, 1697, 994, 750, 564.

HRMS (ESI) *m/z*: Calc. For $C_{19}H_{21}N_2O$ ($[M+H]^+$) 293.1648, Found 293.1651.

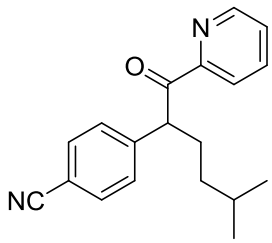

11150C

4-(5-methyl-1-oxo-1-(pyridin-2-yl)hexan-2-yl)benzonitrile (49)

Yield 40.5 mg, 69%. Light yellow oil, R_f = 0.30 (petroleum ether/ethyl acetate, 10:1).

^{1}H NMR (400 MHz, $CDCl_3$) δ 8.66 (d, *J* = 4.8 Hz, 1H), 8.02 (d, *J* = 7.9 Hz, 1H), 7.80 (td, *J* = 7.7, 1.7 Hz, 1H), 7.57 – 7.51 (m, 4H), 7.45 – 7.42 (m, 1H), 5.43 (t, *J* = 7.5 Hz,

1H), 2.20 – 2.14 (m, 1H), 1.90 – 1.82 (m, 1H), 1.61 – 1.52 (m, 1H), 1.22 – 1.15 (m, 1H), 1.12 – 1.04 (m, 1H), 0.85 (t, *J* = 7.0 Hz, 6H).

^{13}C NMR (101 MHz, $CDCl_3$) δ 200.7, 152.7, 149.1, 145.3, 137.1, 132.4, 129.9, 127.5, 122.9, 119.0, 110.7, 51.1, 36.8, 31.0, 28.1, 22.7, 22.5.

IR (KBr) ν 2932, 2850, 2227, 1696, 1447, 994, 750.

HRMS (ESI) *m/z*: Calc. For $C_{19}H_{21}N_2O$ ($[M+H]^+$) 293.1648, Found 293.1650.

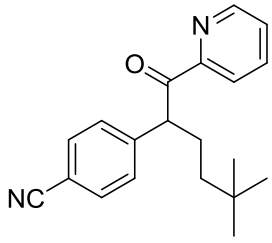

11154E

4-(5,5-dimethyl-1-oxo-1-(pyridin-2-yl)hexan-2-yl)benzonitrile (50)

Yield 50.6 mg, 81%. Light yellow oil, R_f = 0.18 (petroleum ether/ethyl acetate, 10:1).

^{1}H NMR (500 MHz, $CDCl_3$) δ 8.67 – 8.66 (m, 1H), 8.02 (d, *J* = 7.8 Hz, 1H), 7.81 – 7.78 (m, 1H), 7.57 – 7.52 (m, 4H), 7.45 – 7.43 (m, 1H), 5.39 (td, *J* = 7.5, 2.6 Hz, 1H), 2.19 – 2.12 (m, 1H), 1.87 – 1.81 (m, 1H), 1.24 – 1.17 (m, 1H), 1.09 – 1.03 (m, 1H), 0.85 (s, 9H).

^{13}C NMR (126 MHz, $CDCl_3$) δ 200.7, 152.6, 149.1, 145.3, 137.1, 132.4, 129.9, 127.5, 122.9, 119.0, 110.7, 51.5, 41.9, 30.4, 29.3, 28.3.

IR (KBr) ν 2955, 2227, 1697, 994, 750, 564.

HRMS (ESI) *m/z*: Calc. For $C_{20}H_{23}N_2O$ ($[M+H]^+$) 307.1805, Found 307.1804.

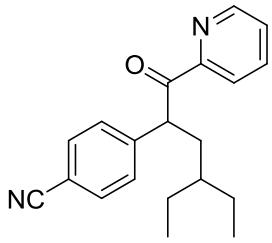

11158D

4-(4-ethyl-1-oxo-1-(pyridin-2-yl)hexan-2-yl)benzonitrile (51)

Yield 40.1 mg, 65%. Light yellow oil, R_f = 0.23 (petroleum ether/ethyl acetate, 10:1).

^{1}H NMR (400 MHz, $CDCl_3$) δ 8.67 (d, *J* = 4.7 Hz, 1H), 8.01 (d, *J* = 7.9 Hz, 1H), 7.80 (t, *J* = 7.7 Hz, 1H), 7.57 – 7.52 (m, 4H), 7.46 – 7.43 (m, 1H), 5.62 (t, *J* = 7.6 Hz, 1H),

2.15 – 2.08 (m, 1H), 1.84 – 1.77 (m, 1H), 1.41 – 1.26 (m, 4H), 1.11 – 1.07 (m, 1H), 0.85 (t, *J* = 7.6 Hz, 3H), 0.78 (t, *J* = 7.5 Hz, 3H).

^{13}C NMR (101 MHz, $CDCl_3$) δ 200.7, 152.6, 149.1, 145.5, 137.1, 132.3, 130.0, 127.4, 122.9, 119.1, 110.6, 48.4, 38.2, 36.4, 25.5, 25.1, 10.7, 10.4.

IR (KBr) ν 2920, 2227, 1697, 1460, 994, 749.

HRMS (ESI) *m/z*: Calc. For $C_{20}H_{23}N_2O$ ($[M+H]^+$) 307.1805, Found 307.1804.

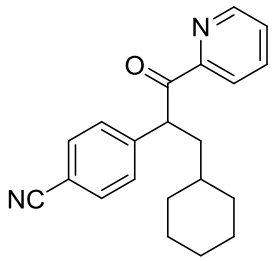

11150B

4-(3-cyclohexyl-1-oxo-1-(pyridin-2-yl)propan-2-yl)benzonitrile (52)

Yield 41.4 mg, 65%. Light yellow oil, R_f = 0.26 (petroleum ether/ethyl acetate, 10:1).

^{1}H NMR (400 MHz, $CDCl_3$) δ 8.67 (d, *J* = 4.8 Hz, 1H), 8.01 (d, *J* = 7.8 Hz, 1H), 7.79 (t, *J* = 7.7 Hz, 1H), 7.56 – 7.51 (m, 4H), 7.45 – 7.42 (m, 1H), 5.64 (t, *J* = 7.5 Hz, 1H), 2.13 – 2.06 (m, 1H), 1.81 – 1.92 (m, 6H), 1.15 – 1.10 (m, 4H), 0.97 – 0.89 (m, 2H).

^{13}C NMR (101 MHz, $CDCl_3$) δ 200.6, 152.6, 149.1, 145.6, 137.1, 132.3, 129.9, 127.4, 122.9, 119.1, 110.6, 48.0, 40.6, 35.7, 33.7, 33.2, 26.6, 26.24, 26.21.

IR (KBr) ν 2955, 2869, 2228, 1697.

HRMS (ESI) *m/z*: Calc. For $C_{21}H_{23}N_2O$ ($[M+H]^+$) 319.1805, Found 319.1806.

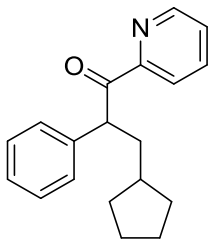

11136H

3-cyclopentyl-2-phenyl-1-(pyridin-2-yl)propan-1-one (53)

Yield 42.6 mg, 76%. Light yellow oil, R_f = 0.33 (petroleum ether/ethyl acetate, 10:1).

^{1}H NMR (500 MHz, $CDCl_3$) δ 8.67 – 8.66 (m, 1H), 7.99 (d, *J* = 7.9 Hz, 1H), 7.75 (td, *J* = 7.7, 1.7 Hz, 1H), 7.41 – 7.37 (m, 3H), 7.26 – 7.23 (m, 2H), 7.15 (t, *J* = 7.4 Hz, 1H), 5.48 (t, *J* = 7.6 Hz, 1H), 2.20 – 2.14 (m, 1H), 1.97 – 1.91 (m, 1H), 1.86 – 1.77 (m,

1H), 1.71 – 1.64 (m, 2H), 1.62 – 1.53 (m, 2H), 1.48 – 1.41 (m, 2H), 1.20 – 1.13 (m, 2H).

^{13}C NMR (126 MHz, $CDCl_3$) δ 201.8, 153.3, 149.0, 139.8, 136.9, 129.1, 128.5, 127.0, 126.8, 122.8, 49.8, 39.5, 38.3, 33.1, 32.7, 25.2.

IR (KBr) ν 2947, 2865, 1694, 859, 836, 701.

HRMS (ESI) *m/z*: Calc. For $C_{19}H_{22}NO$ ($[M+H]^+$) 280.1696, Found 280.1695.

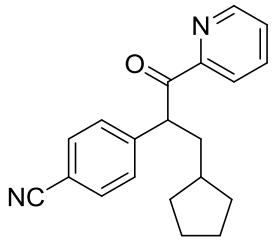

11193B

4-(3-cyclopentyl-1-oxo-1-(pyridin-2-yl)propan-2-yl)benzonitrile (54)

Yield 50.0 mg, 82%. Light yellow oil, R_f = 0.11 (petroleum ether/ethyl acetate, 10:1).

^{1}H NMR (500 MHz, $CDCl_3$) δ 8.67 (d, *J* = 4.5 Hz, 1H), 8.01 (dd, *J* = 8.0, 2.4 Hz, 1H), 7.81 – 7.78 (m, 1H), 7.58 – 7.53 (m, 4H), 7.45 – 7.43 (m, 1H), 5.57 – 5.54 (m, 1H), 2.18 – 2.14 (m, 1H), 1.95 – 1.89 (m, 1H), 1.78 – 1.75 (m, 1H), 1.68 – 1.58 (m, 4H), 1.45 – 1.43 (m, 2H), 1.20 – 1.09 (m, 2H).

^{13}C NMR (126 MHz, $CDCl_3$) δ 200.7, 152.6, 149.1, 145.4, 137.1, 132.3, 129.9, 127.4, 122.9, 119.1, 110.7, 50.0, 39.3, 38.2, 33.1, 32.6, 25.2, 25.2.

IR (KBr) ν 2948, 2865, 2228, 1696, 1605, 1314.

HRMS (APCI) *m/z*: Calc. For $C_{20}H_{21}N_2O$ ($[M+H]^+$) 305.1648, Found 305.1641

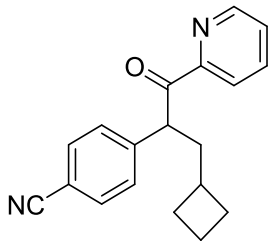

11154K

4-(3-cyclobutyl-1-oxo-1-(pyridin-2-yl)propan-2-yl)benzonitrile (55)

Yield 33.2 mg, 57%. Light yellow oil, R_f = 0.20 (petroleum ether/ethyl acetate, 10:1).

^{1}H NMR (500 MHz, $CDCl_3$) δ 8.66 (d, *J* = 4.8 Hz, 1H), 8.01 (d, *J* = 7.8 Hz, 1H), 7.79 (t, *J* = 7.6 Hz, 1H), 7.55 – 7.50 (m, 4H), 7.45 – 7.42 (m, 1H), 5.41 (t, *J* = 7.5 Hz, 1H),

2.25 – 2.21 (m, 1H), 2.17 – 2.14 (m, 1H), 2.00 – 1.96 (m, 2H), 1.89 – 1.86 (m, 1H), 1.78 – 1.67 (m, 3H), 1.59 – 1.55 (m, 1H).

^{13}C NMR (126 MHz, $CDCl_3$) δ 200.5, 152.6, 149.1, 145.3, 137.1, 132.3, 129.9, 127.4, 122.9, 119.1, 110.7, 49.0, 40.2, 34.4, 28.6, 28.3, 18.5.

IR (KBr) ν 2930, 2227, 1696, 1330.

HRMS (ESI) *m/z*: Calc. For $C_{19}H_{19}N_2O$ ($[M+H]^+$) 291.1492, Found 291.1492.

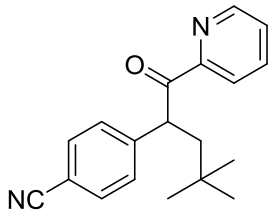

11154L

4-(4,4-dimethyl-1-oxo-1-(pyridin-2-yl)pentan-2-yl)benzonitrile (56)

Yield 40.3 mg, 69%. Light yellow oil, R_f = 0.23 (petroleum ether/ethyl acetate, 10:1).

^{1}H NMR (500 MHz, $CDCl_3$) δ 8.71 – 8.70 (m, 1H), 8.01 (d, *J* = 7.8 Hz, 1H), 7.79 (td, *J* = 7.7, 1.8 Hz, 1H), 7.55 – 7.51 (m, 4H), 7.46 – 7.44 (m, 1H), 5.73 (dd, *J* = 8.9, 3.7 Hz, 1H), 2.55 (dd, *J* = 13.9, 8.9 Hz, 1H), 1.63 (dd, *J* = 13.9, 3.8 Hz, 1H), 0.88 (s, 9H).

^{13}C NMR (126 MHz, $CDCl_3$) δ 200.6, 152.4, 149.1, 146.8, 137.1, 132.4, 129.8, 127.5, 123.1, 119.0, 110.5, 46.9, 46.8, 31.6, 29.9.

IR (KBr) ν 2954, 2228, 1698, 563.

HRMS (ESI) *m/z*: Calc. For $C_{19}H_{21}N_2O$ ($[M+H]^+$) 293.1647, Found 293.1648.

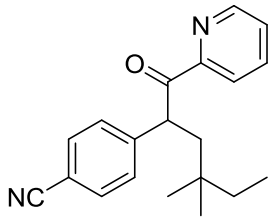

11166A

4-(4,4-dimethyl-1-oxo-1-(pyridin-2-yl)hexan-2-yl)benzonitrile (57)

Yield 45.6 mg, 74%. Light yellow oil, R_f = 0.23 (petroleum ether/ethyl acetate, 10:1).

^{1}H NMR (500 MHz, $CDCl_3$) δ 8.70 (d, *J* = 4.8 Hz, 1H), 8.01 (d, *J* = 7.8 Hz, 1H), 7.79 (t, *J* = 7.7 Hz, 1H), 7.55 – 7.51 (m, 4H), 7.46 – 7.43 (m, 1H), 5.71 (dd, *J* = 8.8, 3.6 Hz,

1H), 2.53 (dd, *J* = 14.0, 8.8 Hz, 1H), 1.62 (dd, *J* = 14.0, 3.6 Hz, 1H), 1.25 (q, *J* = 7.4 Hz, 2H), 0.81 – 0.77 (m, 9H).

^{13}C NMR (126 MHz, $CDCl_3$) δ 200.6, 152.4, 149.1, 146.9, 137.1, 132.4, 129.8, 127.4, 123.1, 119.0, 110.5, 46.3, 44.6, 34.7, 34.1, 27.1, 27.0, 8.5.

IR (KBr) ν 2961, 2227, 1697, 1463, 994, 564.

HRMS (ESI) *m/z*: Calc. For $C_{20}H_{23}N_2O$ ($[M+H]^+$) 307.1805, Found 307.1805.

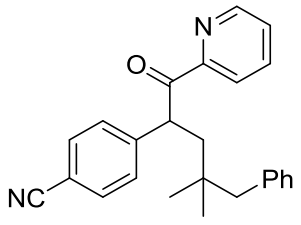

11158G

4-(4,4-dimethyl-1-oxo-5-phenyl-1-(pyridin-2-yl)pentan-2-yl)benzonitrile (58)

Yield 58.4 mg, 79%. Light yellow oil, R_f = 0.18 (petroleum ether/ethyl acetate, 10:1).

^{1}H NMR (400 MHz, $CDCl_3$) δ 8.72 (d, *J* = 4.7 Hz, 1H), 8.00 (d, *J* = 7.8 Hz, 1H), 7.79 (t, *J* = 7.7 Hz, 1H), 7.56 – 7.51 (m, 4H), 7.47 – 7.44 (m, 1H), 7.26 – 7.17 (m, 3H), 7.08 (d, *J* = 7.1 Hz, 2H), 5.84 (dd, *J* = 9.2, 3.3 Hz, 1H), 2.63 (dd, *J* = 13.9, 9.0 Hz, 1H), 2.55 (s, 2H), 1.68 (dd, *J* = 13.9, 3.3 Hz, 1H), 0.83 (s, 3H), 0.81 (s, 3H).

^{13}C NMR (126 MHz, $CDCl_3$) δ 200.5, 152.3, 149.1, 146.6, 138.8, 137.2, 132.4, 130.7, 129.8, 127.9, 127.5, 126.1, 123.2, 119.0, 110.6, 48.9, 46.3, 45.3, 35.3, 27.2, 27.0.

IR (KBr) ν 2958, 2227, 1697, 994, 702, 562.

HRMS (ESI) *m/z*: Calc. For $C_{25}H_{25}N_2O$ ($[M+H]^+$) 369.1961, Found 369.1959.

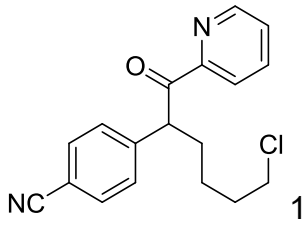

11154J

4-(6-chloro-1-oxo-1-(pyridin-2-yl)hexan-2-yl)benzonitrile (59)

Yield 43.1 mg, 69%. Light yellow oil, R_f = 0.17 (petroleum ether/ethyl acetate, 5:1).

^{1}H NMR (500 MHz, $CDCl_3$) δ 8.66 (d, *J* = 4.7 Hz, 1H), 8.02 (d, *J* = 7.9 Hz, 1H), 7.80 (t, *J* = 7.8 Hz, 1H), 7.57 – 7.51 (m, 4H), 7.46 – 7.43 (m, 1H), 5.48 (t, *J* = 7.5 Hz, 1H),

3.50 (t, *J* = 6.6 Hz, 2H), 2.22 – 2.18 (m, 1H), 1.92 – 1.77 (m, 3H), 1.46 – 1.37 (m, 2H).

^{13}C NMR (126 MHz, $CDCl_3$) δ 200.3, 152.4, 149.1, 144.8, 137.2, 132.4, 129.8, 127.6, 122.9, 118.9, 110.9, 50.7, 44.7, 32.5, 32.2, 25.0.

IR (KBr) ν 2937, 2228, 1696, 1605, 1435, 1397, 1088, 1042, 995, 749, 565.

HRMS (EI) *m/z*: Calc. For $C_{18}H_{17}N_2OCl$ ($[M]^+$) 312.1024, Found 312.1023.

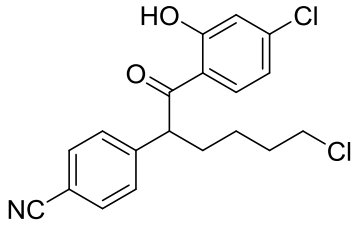

11188C

4-(6-chloro-1-(4-chloro-2-hydroxyphenyl)-1-oxohexan-2-yl)benzonitrile (60)

Yield 54.8 mg, 76%. Oil, R_f = 0.35 (petroleum ether/ethyl acetate, 5:1).

^{1}H NMR (500 MHz, $CDCl_3$) δ 12.35 (s, 1H), 7.67 – 7.62 (m, 3H), 7.43 (d, *J* = 8.0 Hz, 2H), 6.99 (d, *J* = 2.2 Hz, 1H), 6.83 (dd, *J* = 8.7, 2.1 Hz, 1H), 4.57 (t, *J* = 7.3 Hz, 1H), 3.51 (q, *J* = 7.0, 6.5 Hz, 2H), 2.24 – 2.18 (m, 1H), 1.90 – 1.75 (m, 3H), 1.50 – 1.37 (m, 2H).

^{13}C NMR (126 MHz, $CDCl_3$) δ 203.9, 164.1, 144.1, 142.9, 133.1, 130.9, 128.9, 120.0, 119.1, 118.5, 117.4, 111.8, 53.0, 44.6, 33.0, 32.4, 25.0.

IR (KBr) ν 2920, 2228, 1635, 1604, 1238.

HRMS (APCI) *m/z*: Calc. For $C_{19}H_{16}NO_2Cl_2$ ($[M-H]^-$) 360.0564, Found 360.0567.

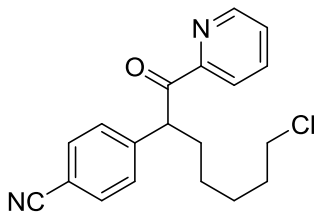

11154F

4-(7-chloro-1-oxo-1-(pyridin-2-yl)heptan-2-yl)benzonitrile (61)

Yield 36.4 mg, 56%. Light yellow oil, R_f = 0.20 (petroleum ether/ethyl acetate, 5:1).

^{1}H NMR (500 MHz, $CDCl_3$) δ 8.66 (d, *J* = 4.9 Hz, 1H), 8.01 (d, *J* = 7.8 Hz, 1H), 7.80 (td, *J* = 7.7, 1.8 Hz, 1H), 7.57 – 7.51 (m, 4H), 7.46 – 7.43 (m, 1H), 5.47 (t, *J* = 7.6 Hz,

1H), 3.49 (t, *J* = 6.6 Hz, 2H), 2.22 – 2.18 (m, 1H), 1.89 – 1.85 (m, 1H), 1.76 – 1.70 (m, 2H), 1.49 – 1.45 (m, 2H), 1.32 – 1.24 (m, 2H).

^{13}C NMR (126 MHz, $CDCl_3$) δ 200.4, 152.5, 149.1, 145.0, 137.2, 132.4, 129.8, 127.5, 122.9, 119.0, 110.8, 50.7, 45.0, 32.8, 32.4, 27.0, 26.8.

IR (KBr) ν 2920, 2870, 2195, 1712.

HRMS (ESI) *m/z*: Calc. For $C_{19}H_{20}N_2OCl$ ($[M+H]^+$) 327.1259, Found 327.1258.

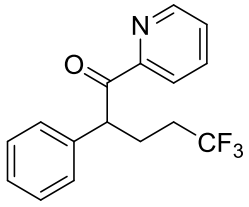

11136B

5,5,5-trifluoro-2-phenyl-1-(pyridin-2-yl)pentan-1-one (62)

Yield 47.9 mg, 82%. Light yellow oil, R_f = 0.26 (petroleum ether/ethyl acetate, 10:1).

^{1}H NMR ^{1}H NMR (500 MHz, $CDCl_3$) δ 8.65 – 8.64 (m, 1H), 7.99 (d, *J* = 7.8 Hz, 1H), 7.76 (td, *J* = 7.7, 1.8 Hz, 1H), 7.41 – 7.36 (m, 3H), 7.27 (t, *J* = 7.3 Hz, 2H), 7.19 (t, *J* = 7.3 Hz, 1H), 5.41 (t, *J* = 7.6 Hz, 1H), 2.41 – 2.34 (m, 1H), 2.23 – 1.97 (m, 3H).

^{13}C NMR (126 MHz, $CDCl_3$) δ 200.3, 152.7, 149.0, 137.9, 137.0, 129.0, 128.9, 128.3, 127.4, 127.3, 127.1 (q, J_{CF} = 276.6 Hz), 126.1, 122.9, 49.7, 31.8 (q, J_{CF} = 28.7 Hz), 24.95 (d, J_{CF} = 3.2 Hz).

IR (KBr) ν 1696, 1389, 1301, 1256, 1213, 1139, 994, 702.

HRMS (ESI) *m/z*: Calc. For $C_{16}H_{15}NOF_3$ ($[M+H]^+$) 294.1100, Found 294.1100.

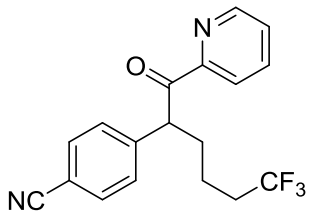

11154G

4-(6,6,6-trifluoro-1-oxo-1-(pyridin-2-yl)hexan-2-yl)benzonitrile (63)

Yield 54.6 mg, 82%. Light yellow oil, R_f = 0.17 (petroleum ether/ethyl acetate, 5:1).

^{1}H NMR (500 MHz, $CDCl_3$) δ 8.66 (d, *J* = 4.8 Hz, 1H), 8.02 (d, *J* = 7.9 Hz, 1H), 7.81 (td, *J* = 7.7, 1.8 Hz, 1H), 7.57 (d, *J* = 8.1 Hz, 2H), 7.51 (d, *J* = 8.1 Hz, 2H), 7.47 –

7.44 (m, 1H), 5.49 (t, J = 7.5 Hz, 1H), 2.28 – 2.22 (m, 1H), 2.16 – 2.07 (m, 2H), 1.96 – 1.92 (m, 1H), 1.60 – 1.48 (m, 2H).

^{13}C NMR (126 MHz, $CDCl_3$) δ 199.9, 152.3, 149.1, 144.3, 137.2, 132.6, 129.8, 127.7, 127.0 (q, J_{CF} = 275 Hz), 123.0, 118.8, 111.1, 50.5, 33.7 (q, J_{CF} = 29 Hz), 31.9, 20.3 (d, J_{CF} = 3 Hz).

IR (KBr) ν 2228, 1698, 1281, 1255, 1136, 1042, 1021, 995, 560.

HRMS (ESI) *m/z*: Calc. For $C_{18}H_{16}N_2F_3O$ ($[M+H]^+$) 333.1209, Found 333.1208.

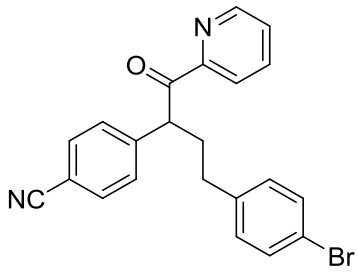

11158H

4-(4-(4-bromophenyl)-1-oxo-1-(pyridin-2-yl)butan-2-yl)benzonitrile (64)

Yield 49.7 mg, 61%. Light yellow oil, R_f = 0.19 (petroleum ether/ethyl acetate, 5:1).

^{1}H NMR (400 MHz, $CDCl_3$) δ 8.64 (d, J = 4.7 Hz, 1H), 7.99 (d, J = 7.8 Hz, 1H), 7.80 (t, J = 7.8 Hz, 1H), 7.58 – 7.50 (m, 4H), 7.45 – 7.42 (m, 1H), 7.36 (d, J = 8.0 Hz, 2H), 6.99 (d, J = 8.0 Hz, 2H), 5.49 – 5.45 (m, 1H), 2.56 – 2.50 (m, 3H), 2.19 – 2.10 (m, 1H).

^{13}C NMR (101 MHz, $CDCl_3$) δ 200.0, 152.4, 149.1, 144.7, 140.3, 137.2, 132.5, 131.6, 130.3, 129.9, 127.6, 122.9, 120.0, 118.9, 111.0, 50.3, 34.3, 33.3.

IR (KBr) ν 2923, 2227, 1696, 1487, 1404, 1073, 1010, 818.

HRMS (ESI) *m/z*: Calc. For $C_{22}H_{16}N_2OBr$ ($[M-H]^-$) 403.0452, Found 403.0458.

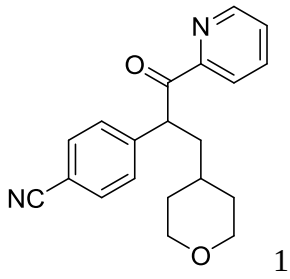

11158A

4-(1-oxo-1-(pyridin-2-yl)-3-(tetrahydro-2H-pyran-4-yl)propan-2-yl)benzonitrile (65)

Yield 44.8 mg, 70%. Light yellow oil, R_f = 0.23 (petroleum ether/ethyl acetate, 5:1).

^{1}H NMR (400 MHz, $CDCl_3$) δ 8.67 (d, *J* = 4.8 Hz, 1H), 8.01 (d, *J* = 7.9 Hz, 1H), 7.81 (t, *J* = 7.7 Hz, 1H), 7.54 (q, *J* = 8.1 Hz, 4H), 7.45 (dd, *J* = 7.5, 4.8 Hz, 1H), 5.66 (t, *J* = 7.6 Hz, 1H), 3.93 – 3.88 (m, 2H), 3.30 – 3.22 (m, 2H), 2.22 – 2.15 (m, 1H), 1.84 – 1.78 (m, 1H), 1.67 (d, *J* = 11.8 Hz, 1H), 1.59 (d, *J* = 12.6 Hz, 1H), 1.41 – 1.28 (m, 3H).

^{13}C NMR (101 MHz, $CDCl_3$) δ 200.2, 152.4, 149.1, 145.0, 137.2, 132.5, 129.8, 127.6, 123.0, 118.9, 110.9, 67.9, 67.9, 47.5, 40.0, 33.3, 33.1, 33.0.

IR (KBr) ν 2924, 2227, 1695, 1090, 994.

HRMS (ESI) *m/z*: Calc. For $C_{20}H_{21}N_2O_2$ ($[M+H]^+$) 321.1598, Found 321.1596.

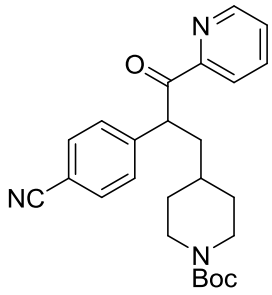

11158B

tert-butyl-4-(2-(4-cyanophenyl)-3-oxo-3-(pyridin-2-yl)propyl)piperidine-1-carboxylate (66)

Yield 81.8 mg, 97%. Light yellow oil, R_f = 0.34 (petroleum ether/ethyl acetate, 5:1).

^{1}H NMR (400 MHz, $CDCl_3$) δ 8.67 (d, *J* = 4.8 Hz, 1H), 8.01 (d, *J* = 7.9 Hz, 1H), 7.80 (t, *J* = 7.7 Hz, 1H), 7.54 (q, *J* = 8.4 Hz, 4H), 7.47 – 7.44 (m, 1H), 5.65 (t, *J* = 7.6 Hz, 1H), 4.03 (brs, 2H), 2.57 (d, *J* = 12.5 Hz, 2H), 2.21 – 2.14 (m, 1H), 1.81 – 1.72 (m, 2H), 1.65 (d, *J* = 12.7 Hz, 1H), 1.43 (s, 9H), 1.32 – 1.26 (m, 1H), 1.16 – 1.13 (m, 2H).

^{13}C NMR (101 MHz, $CDCl_3$) δ 200.2, 154.9, 152.4, 149.1, 145.0, 137.2, 132.5, 129.8, 127.6, 123.0, 118.9, 110.9, 79.4, 47.7, 39.6, 34.1, 32.4, 28.6.

IR (KBr) ν 2928, 2228, 1692, 1423, 1244, 1166.

HRMS (ESI) *m/z*: Calc. For $C_{25}H_{28}N_3O_3$ ($[M-H]^-$) 418.2136, Found 418.2146.

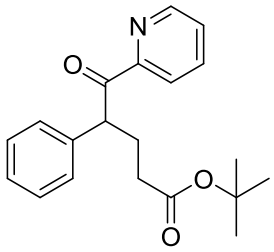

11151D

tert-butyl 5-oxo-4-phenyl-5-(pyridin-2-yl)pentanoate (67)

Yield 59.1 mg, 91%. Light yellow oil, R_f = 0.24 (petroleum ether/ethyl acetate, 10:1).

^{1}H NMR (400 MHz, $CDCl_3$) δ 8.63 (d, *J* = 5.0 Hz, 1H), 8.00 – 7.98 (m, 1H), 7.76 – 7.72 (m, 1H), 7.39 – 7.37 (m, 3H), 7.25 (t, *J* = 7.5 Hz, 2H), 7.18 – 7.15 (m, 1H), 5.43 (t, *J* = 6.9 Hz, 1H), 2.44 – 2.38 (m, 1H), 2.23 – 2.15 (m, 3H), 1.42 (s, 9H).

^{13}C NMR (101 MHz, $CDCl_3$) δ 201.0, 172.7, 153.0, 149.0, 138.7, 136.9, 129.1, 128.7, 127.1, 122.8, 80.3, 49.9, 33.7, 28.3, 28.2.

IR (KBr) ν 1695, 1366, 1148, 701.

HRMS (ESI) *m/z*: Calc. For $C_{20}H_{24}NO_3$ ($[M+H]^+$) 326.1751, Found 326.1749.

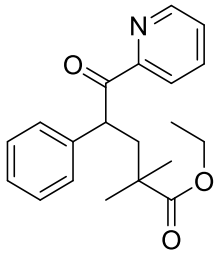

11156B

ethyl 2,2-dimethyl-5-oxo-4-phenyl-5-(pyridin-2-yl)pentanoate (68)

Yield 45.2 mg, 68%. Light yellow oil, R_f = 0.13 (petroleum ether/ethyl acetate, 10:1).

^{1}H NMR (500 MHz, $CDCl_3$) δ 8.67 (d, *J* = 4.8 Hz, 1H), 7.98 (d, *J* = 7.9 Hz, 1H), 7.75 – 7.72 (m, 1H), 7.38 (d, *J* = 8.1 Hz, 3H), 7.23 – 7.20 (m, 2H), 7.13 (t, *J* = 7.4 Hz, 1H), 5.60 (dd, *J* = 8.1, 4.6 Hz, 1H), 3.98 – 3.92 (m, 1H), 3.88 – 3.83 (m, 1H), 2.71 (dd, *J* = 14.1, 8.1 Hz, 1H), 2.12 (dd, *J* = 14.1, 4.6 Hz, 1H), 1.19 (s, 3H), 1.17 (s, 3H), 1.13 (t, *J* = 7.2 Hz, 3H).

^{13}C NMR (126 MHz, $CDCl_3$) δ 200.8, 177.5, 152.8, 149.0, 140.0, 136.8, 129.2, 128.6, 127.0, 126.8, 122.9, 60.4, 47.1, 43.3, 42.4, 26.4, 25.2, 14.1.

IR (KBr) ν 1696, 1193, 1136, 701.

HRMS (EI) *m/z*: Calc. For $C_{20}H_{23}NO_3$ ($[M]^+$) 325.1672, Found 325.1671.

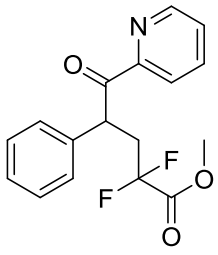

11156A

methyl 2,2-difluoro-5-oxo-4-phenyl-5-(pyridin-2-yl)pentanoate (69)

Yield 42.8 mg, 69%. Light yellow oil, R_f = 0. (petroleum ether/ethyl acetate, 10:1).

^{1}H NMR (500 MHz, $CDCl_3$) δ 8.67 – 8.66 (m, 1H), 8.00 (d, *J* = 7.8 Hz, 1H), 7.75 (td, *J* = 7.7, 1.8 Hz, 1H), 7.41 – 7.38 (m, 3H), 7.26 – 7.23 (m, 2H), 7.18 – 7.15 (m, 1H), 5.81 – 5.78 (m, 1H), 3.66 (s, 3H), 3.28 – 3.17 (m, 1H), 2.68 – 2.57 (m, 1H).

^{13}C NMR (126 MHz, $CDCl_3$) δ 198.7, 164.4 (t, J_{CF}= 33 Hz), 152.1, 149.1, 137.5, 136.9, 129.1, 128.8, 127.5, 127.3, 123.1, 115.6 (t, J_{CF} = 251 Hz), 53.3, 44.2 (t, J_{CF} = 4 Hz), 37.5 (t, J_{CF} = 24 Hz).

IR (KBr) ν 1769, 1698, 1241, 1092.

HRMS (ESI) *m/z*: Calc. For $C_{17}H_{16}NF_2O_3$ ($[M+H]^+$) 320.1093, Found 320.1090.

2.3 **Cascade alkylacylation/cyclopropanation** (Fig. 3 and Fig. 4)

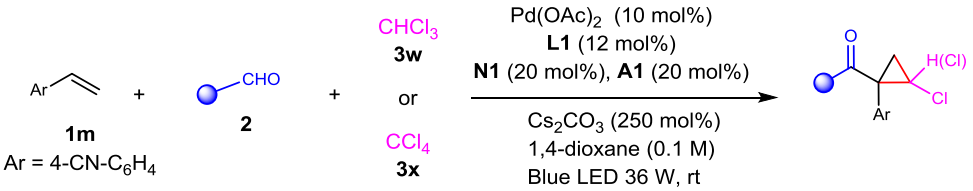

A 4 mL vial equipped with a stir bar was charged with preNHC **N1** (10.8 mg, 0.04 mmol), $Pd(OAc)_2$ (4.5 mg, 0.02 mmol), ligand **L1** (11.9 mg, 0.024 mmol) and 1.0 mL of 1,4-dioxane. After stirring for 30 min in glove box, to the solution was added Cs_2CO_3 (97.8 mg, 0.3 mmol), additive **A1** (4.4 mg, 0.04 mmol), styrene **1m** (25.8 mg, 0.2 mmol), aldehydes **2** (0.4 mmol), $CHCl_3$ **3w** (238.8 mg, 2.0 mmol) or CCl_4 **3x** (153.8 mg, 1.0 mmol), and 1.0 mL of 1,4-dioxane. The reaction mixtrue was removed from the glove box and stirred under 36W blue LED lights at room

temperature until the complete consumption of **1m** (generally 48 hours) by TLC analysis. The reaction mixture was filtered through a small pad of silica and eluted with EtOAc. The solution was concentrated under reduced pressure, and purified by column chromatography on silica gel to afford the desired cyclopropanes **70-79**.

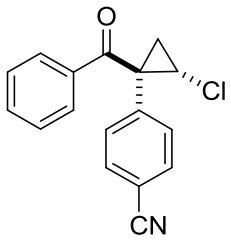

11199D

4-(1-benzoyl-2-chlorocyclopropyl)benzonitrile (70)

Yield 34.4 mg, 61%, > 20:1 dr. Light yellow oil, R_f = 0.12 (petroleum ether/ethyl acetate, 10:1).

^{1}H NMR (500 MHz, $CDCl_3$) δ 7.67 (d, *J* = 7.4 Hz, 2H), 7.61 (d, *J* = 8.2 Hz, 2H), 7.46 – 7.42 (m, 3H), 7.31 (t, *J* = 7.8 Hz, 2H), 4.18 (dd, *J* = 7.7, 5.0 Hz, 1H), 2.07 – 1.99 (m, 2H).

^{13}C NMR (126 MHz, $CDCl_3$) δ 196.7, 140.7, 135.8, 133.2, 132.5, 131.0, 129.4, 128.6, 118.5, 111.9, 41.6, 38.3, 23.5.

IR (KBr) ν 2229, 1674, 1598, 1448, 1318, 1255, 653.

HRMS (APCI) *m/z*: Calc. For $C_{17}H_{13}NO_3Cl$ ($[M+H]^+$) 282.0680, Found 282.0674.

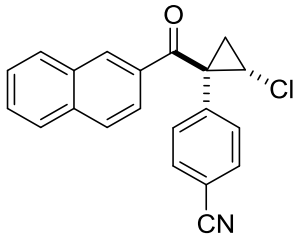

11193C

4-(1-(2-naphthoyl)-2-chlorocyclopropyl)benzonitrile (71)

Yield 34.1 mg, 52%, 11:1 dr. Colorless oil, R_f = 0.14 (petroleum ether/ethyl acetate, 10:1).

^{1}H NMR (500 MHz, $CDCl_3$) δ 8.20 (s, 1H), 7.79 (dd, *J* = 8.2, 4.2 Hz, 2H), 7.74 – 7.73 (m, 2H), 7.61 – 7.55 (m, 3H), 7.52 – 7.48 (m, 3H), 4.26 – 4.24 (m, 1H), 2.14 – 2.07 (m, 2H).

^{13}C NMR (126 MHz, $CDCl_3$) δ 196.5, 140.9, 135.4, 133.0, 132.5, 132.2, 131.4, 130.9, 129.6, 129.0, 128.5, 127.9, 127.2, 124.7, 118.5, 111.9, 41.7, 38.4, 23.6.

IR (KBr) ν 2920, 2229, 1670, 1403, 1276, 1196.

HRMS (APCI) *m/z*: Calc. For $C_{21}H_{15}NO_3Cl$ ($[M+H]^+$) 332.0837, Found 332.0828.

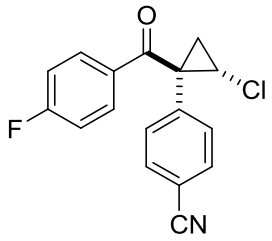

11200A

4-(2-chloro-1-(4-fluorobenzoyl)cyclopropyl)benzonitrile (72)

Yield 31.4 mg, 52%, 13:1 dr. Light yellow oil, R_f = 0.17 (petroleum ether/ethyl acetate, 10:1).

^{1}H NMR (500 MHz, $CDCl_3$) δ 7.74 – 7.62 (m, 4H), 7.46 – 7.41 (m, 2H), 7.01 – 6.97 (m, 2H), 4.19 – 4.17 (m, 1H), 2.12 – 1.97 (m, 2H).

^{13}C NMR (126 MHz, $CDCl_3$) δ 194.9, 165.5 (d, J_{CF} = 258 Hz), 140.5, 132.6, 132.1 (d, J_{CF} = 9 Hz), 130.8, 118.4, 115.8 (d, J_{CF} = 22 Hz), 112.1, 41.4, 38.2, 23.3.

IR (KBr) ν 2229, 1726, 1675, 1600, 1506, 1257, 1236, 1155, 848.

HRMS (EI) *m/z*: Calc. For $C_{17}H_{11}NOFCl$ ($[M]^+$) 299.0508, Found 299.0508.

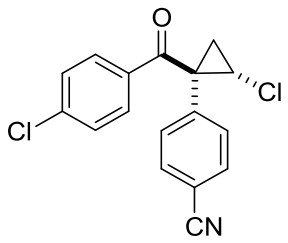

11199F

4-(-2-chloro-1-(4-chlorobenzoyl)cyclopropyl)benzonitrile (73)

Yield 62.4 mg, 67%, > 20:1 dr. Light yellow oil, R_f = 0.19 (petroleum ether/ethyl acetate, 10:1).

^{1}H NMR (500 MHz, $CDCl_3$) δ 7.63 – 7.60 (td, *J* = 8.2, 2.3 Hz, 4H), 7.41 – 7.40 (m, 2H), 7.29 – 7.26 (m, 2H), 4.19 – 4.17 (m, 1H), 2.07 – 1.98 (m, 2H).

^{13}C NMR (126 MHz, $CDCl_3$) δ 195.5, 140.4, 139.7, 134.1, 132.6, 130.9, 130.8, 128.9, 118.4, 112.1, 41.5, 38.3, 23.6.

IR (KBr) ν 2920, 2850, 2229, 1726, 1675, 1589, 1257, 1092.

HRMS (APCI) *m/z*: Calc. For $C_{17}H_{12}NOCl$ ($[M+H]^+$) 316.0291, Found 316.0282

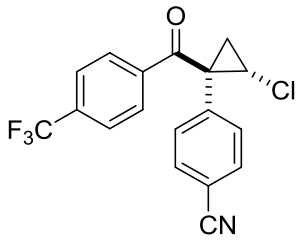

11199E

4-(2-chloro-1-(4-(trifluoromethyl)benzoyl)cyclopropyl)benzonitrile (74)

Yield 49.7 mg, 71%, > 20:1 dr. Light yellow oil, R_f = 0.13 (petroleum ether/ethyl acetate, 10:1).

^{1}H NMR (500 MHz, $CDCl_3$) δ 7.74 – 7.73 (m, 3H), 7.64 – 7.56 (m, 3H), 7.41 – 7.40 (m, 2H), 4.22 – 4.20 (m, 1H), 2.09 – 2.05 (m, 2H).

^{13}C NMR (126 MHz,) δ 196.2, 140.0, 139.0, 132.7, 131.1, 129.5, 129.5, 125.6, 123.3 (q, J_{CF} = 233 Hz),118.3, 112.3, 41.7, 38.6, 24.2.

IR (KBr) ν 2230, 1683, 1326, 1169, 1131, 1066.

HRMS (APCI) *m/z*: Calc. For $C_{17}H_{10}NOF_3Cl$ ($[M-H]^-$) 348.0409, Found 348.0399.

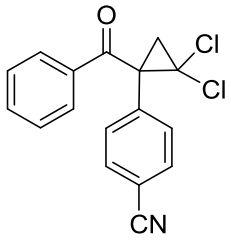

11199H

4-(1-benzoyl-2,2-dichlorocyclopropyl)benzonitrile (75)

Yield 47.2 mg, 75%. Light yellow oil, R_f = 0.19 (petroleum ether/ethyl acetate, 10:1).

^{1}H NMR (500 MHz, $CDCl_3$) δ 7.96 (d, *J* = 7.3 Hz, 2H), 7.73 (d, *J* = 8.2 Hz, 2H), 7.62 (d, *J* = 8.2 Hz, 2H), 7.57 (t, *J* = 7.3 Hz, 1H), 7.48 (t, *J* = 7.6 Hz, 2H), 2.67 (d, *J* = 7.7 Hz, 1H), 2.25 (d, *J* = 7.7 Hz, 1H).

^{13}C NMR (126 MHz, $CDCl_3$) δ 192.1, 139.9, 134.5, 133.9, 132.5, 131.3, 129.8, 128.8, 118.3, 112.5, 60.8, 48.4, 32.0.

IR (KBr) ν 2230, 1681, 1323, 1259, 1066.

HRMS (APCI) *m/z*: Calc. For $C_{17}H_{12}NOCl_2$ ($[M+H]^+$) 316.0291, Found 316.0284.

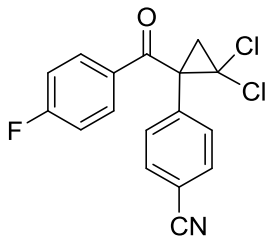

11200E

4-(2,2-dichloro-1-(4-fluorobenzoyl)cyclopropyl)benzonitrile (76)

Yield 34.6 mg, 52%. Light yellow oil, R_f = 0. 21(petroleum ether/ethyl acetate, 10:1).

^{1}H NMR (500 MHz, $CDCl_3$) δ 8.03 – 8.00 (m, 2H), 7.71 (d, *J* = 8.7 Hz, 2H), 7.63 (d, *J* = 8.4 Hz, 2H), 7.16 (t, *J* = 8.7 Hz, 2H), 2.65 (dd, *J* = 7.6, 1.4 Hz, 1H), 2.24 (d, *J* = 7.6 Hz, 1H).

^{13}C NMR (126 MHz, $CDCl_3$) δ 190.5, 166.1 (d, J_{CF} = 257 Hz), 139.7, 132.6, 132.5, 131.2, 130.8 (d, J_{CF} = 3 Hz), 118.2, 116.2 (d, J_{CF} = 22 Hz), 112.6, 60.7, 48.3, 32.1.

IR (KBr) ν 2220, 1681, 1598, 1260, 1238, 1154.

HRMS (EI) *m/z*: Calc. For $C_{17}H_{10}NOFCl_2$ ($[M]^+$) 333.0118, Found 333.0118.

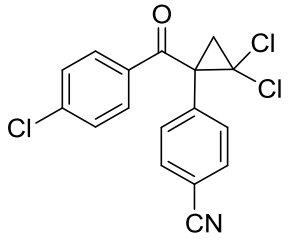

11200F

4-(2,2-dichloro-1-(4-chlorobenzoyl)cyclopropyl)benzonitrile (77)

Yield 54.1mg, 77%. Light yellow oil, R_f = 0. 21(petroleum ether/ethyl acetate, 10:1).

^{1}H NMR (500 MHz, $CDCl_3$) δ 7.91 (d, *J* = 8.8 Hz, 2H), 7.70 (d, *J* = 7.7 Hz, 2H), 7.63 (d, *J* = 8.6 Hz, 2H), 7.45 (d, *J* = 8.7 Hz, 2H), 2.66 (d, *J* = 7.8 Hz, 1H), 2.23 (d, *J* = 7.7 Hz, 1H).

^{13}C NMR (126 MHz, $CDCl_3$) δ 190.9, 140.6, 139.6, 132.8, 132.6, 131.24, 131.16, 129.3, 118.2, 112.7, 60.7, 48.3, 32.1.

IR (KBr) ν 2921, 2360, 1728, 1260, 1066.

HRMS (EI) *m/z*: Calc. For $C_{17}H_{10}NOCl_3$ ($[M]^+$) 348.9822, Found 348.9822.

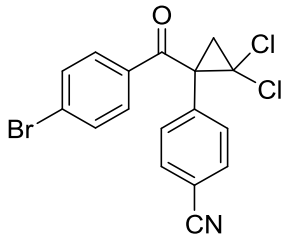

11200G

4-(1-(4-bromobenzoyl)-2,2-dichlorocyclopropyl)benzonitrile (78)

Yield 46.7 mg, 59%. Light yellow oil, R_f = 0.18 (petroleum ether/ethyl acetate, 10:1).

^{1}H NMR (500 MHz, $CDCl_3$) δ 7.83 (d, *J* = 8.2 Hz, 2H), 7.70 (d, *J* = 8.2 Hz, 2H), 7.63 (d, *J* = 8.3 Hz, 4H), 2.66 (d, *J* = 7.7 Hz, 1H), 2.22 (d, *J* = 7.7 Hz, 1H).

^{13}C NMR (126 MHz, $CDCl_3$) δ 191.1, 139.5, 133.2, 132.6, 132.3, 131.2, 131.2, 129.3, 118.2, 112.7, 60.7, 48.3, 32.0.

IR (KBr) ν 2360, 2340, 1682, 1196, 1133.

HRMS (APCI) *m/z*: Calc. For $C_{17}H_9NO_3Cl_2Br$ ($[M-H]^-$) 391.9250, Found 391.9245.

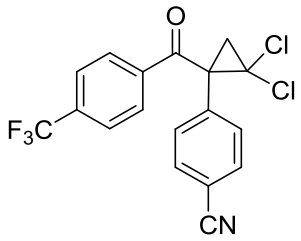

11200H

4-(2,2-dichloro-1-(4-(trifluoromethyl)benzoyl)cyclopropyl)benzonitrile (79)

Yield 53.4 mg, 70%. Light yellow oil, R_f = 0.16 (petroleum ether/ethyl acetate, 10:1).

^{1}H NMR (500 MHz, $CDCl_3$) δ 8.06 (d, *J* = 8.0 Hz, 2H), 7.81 – 7.72 (m, 2H), 7.70 (d, *J* = 8.4 Hz, 2H), 7.63 (d, *J* = 8.4 Hz, 2H), 2.71 (d, *J* = 7.6 Hz, 1H), 2.25 (d, *J* = 7.7 Hz, 1H).

^{13}C NMR (126 MHz, $CDCl_3$) δ 191.4, 139.2, 137.5, 135.1 (q, J_{CF} = 33 Hz), 132.7, 131.4, 130.0, 125.9 (d, J_{CF} = 4 Hz), 123.4 (q, J_{CF} = 272 Hz), 118.1, 112.9, 60.7, 48.5, 32.0.

IR (KBr) ν 2361, 1688, 1326, 1132, 1067.

HRMS (APCI) *m/z*: Calc. For $C_{18}H_9NOF_3Cl_2$ ($[M-H]^-$) 382.0019, Found 382.0022.

2.4 Gram-scale reaction and further transformations (Fig. 5**)**

Gram-scale reaction:

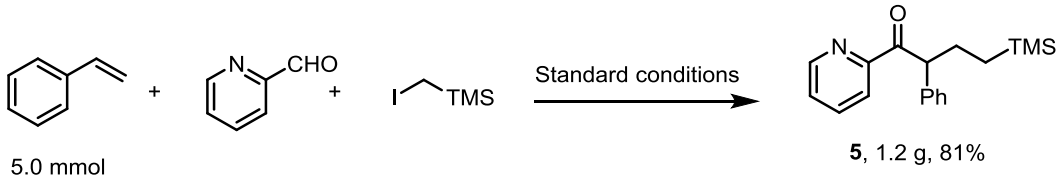

A 4 mL vial equipped with a stir bar was charged with preNHC **N1** (270 mg, 1.0 mmol), $Pd(OAc)_2$ (112.5 mg, 0.5 mmol), ligand **L1** (297.5 mg, 0.6 mmol) and 20 mL of 1,4-dioxane. After stirring for 30 min in glove box, to the solution was added Cs_2CO_3 (2.45 g, 7.5 mmol), additive **A1** (110 mg, 1.0 mmol), styrene **1b** (520 mg, 5.0 mmol), aldehyde **2a** (1.07 g, 10.0 mmol), alkyl halide **3a** (1.61 g, 7.5 mmol), and 20 mL of 1,4-dioxane. The reaction mixtrue was removed from the glove box and stirred under 36W blue LED lights at room temperature until the complete consumption of **1b** by TLC analysis. The reaction mixture was filtered through a small pad of silica and eluted with EtOAc. The solution was concentrated under reduced pressure, and purified by column chromatography on silicagal to afford the desired ketones **5** (1.21 g, 81%).

Reaction a:

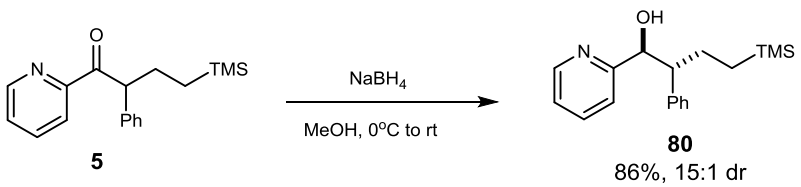

To a solution of $NaBH_4$ (9.8 mg, 0.22 mmol, 1.1 eq) in methanol (2.0 mL) in an ice/water bath was added dropwise the solution of ketone **5** (59.4 mg, 0.2 mmol) in THF (1.0 mL). The reaction mixture was allowed to warm to room temperature. After

stirring for 6 h, the reaction was quenched by addition of saturated aqueous NH_4Cl (2 mL) and extracted with ethyl acetate for three times. The combined organic layer was dried over anhydrous Na_2SO_4. After filtration, the solvent was removed under reduced pressure, and the residue was purified by column chromatography on silica gel to give alcohol **80** as a colorless oil (51.4 mg, 86% yield) with 15:1 dr.

R_f = 0.37 (petroleum ether/ethyl acetate, 2:1).

^{1}H NMR (400 MHz, $CDCl_3$) δ 8.44 (d, *J* = 5.0 Hz, 1H), 7.68 – 7.64 (m, 1H), 7.31 – 7.14 (m, 5H), 7.09 – 7.05 (m, 2H), 5.06 (t, *J* = 5.2 Hz, 1H), 3.95 (d, *J* = 6.1 Hz, 1H), 3.02 – 2.97 (m, 1H), 1.94 – 1.87 (m, 2H), 0.55 – 0.41 (m, 2H), 0.00 (s, 9H).

^{13}C NMR (101 MHz, $CDCl_3$) δ 160.9, 148.0, 140.2, 136.3, 129.3, 128.0, 126.5, 122.2, 121.1, 76.0, 56.5, 26.4, 14.8, -1.6.

IR (KBr) ν 2951, 1595, 1246, 1072, 860, 834, 702.

HRMS (APCI) *m/z*: Calc. For $C_{18}H_{26}NOSi$ ($[M+H]^+$) 300.1778, Found 300.1768.

Reaction b:

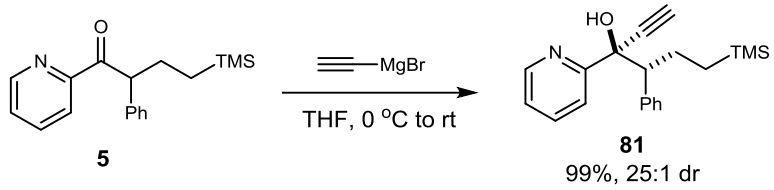

5 **81**

99%, 25:1 dr

To a solution of ketone **5** (59.4 mg, 0.2 mmol) in THF (1.5 mL) at 0 ℃ under N_2 atmosphere was added a solution of ethynyl magnesium bromide (0.5 M in THF, 0.56 mL, 1.4eq) dropwise. The reaction mixture was allowed to warm to room temperature and stirred for 4 hours. After completion, the reaction was quenched by addition of saturated aqueous NH_4Cl (2.0 mL). The mixture was diluted with water (5.0 mL) and was extracted with ethyl acetate for three times. The combined organic layer was washed with brine and was dried over anhydrous Na_2SO_4. After filtration, the solvent was removed under reduced pressure, and the residue was purified by column

chromatography on silica gel to give alcohol **81** as a colorless oil (63.8 mg, 99% yield) with 25:1 dr.

R_f = 0.18 (petroleum ether/ethyl acetate, 10:1).

^{1}H NMR (400 MHz, $CDCl_3$) δ 8.25 (d, *J* = 4.9 Hz, 1H), 7.71 (t, *J* = 7.6 Hz, 1H), 7.65 – 7.63 (m, 1H), 7.14 – 7.10 (m, 4H), 7.01 – 7.00 (m, 2H), 5.51 (s, 1H), 3.07 (dd, *J* = 11.7, 3.0 Hz, 1H), 2.68 (s, 1H), 2.47 – 2.38 (m, 1H), 2.17 – 2.06 (m, 1H), 0.33 – 0.29 (m, 2H), 0.00 (s, 9H).

^{13}C NMR (101 MHz, $CDCl_3$) δ 159.7, 146.7, 138.4, 136.8, 130.0, 127.6, 126.6, 122.6, 121.3, 86.4, 74.6, 73.4, 61.1, 24.4, 14.8, -1.6.

IR (KBr) ν 3304, 2850, 1434, 1247, 836.

HRMS (APCI) *m/z*: Calc. For $C_{20}H_{26}NOSi$ ($[M+H]^+$) 324.1778, Found 324.1769

Reaction c:

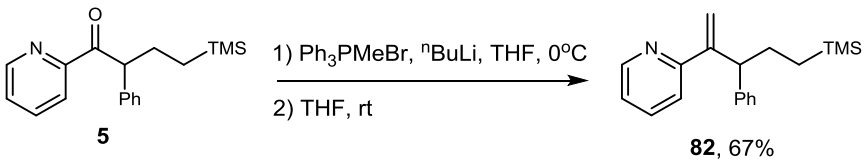

To a flask charged with methyltriphenylphosphonium bromide (143 mg, 0.4 mmol) in 4.0 mL of anhydrous THF at 0 ℃ was added *n*-butyllithium (0.25 mL, 1.6 M in hexanes, 0.4 mmol). The reaction was allowed to warm to room temperature spontaneously and then stirred for 1 h. Ketone **5** (59.4 mg, 0.2 mmol) in anhydrous THF (1.0 mL) was added dropwise at room temperature. After stirring for 9 h, the reaction mixture was quenched with saturated aqueous NH_4Cl, and then extracted with ethyl acetate for three times. The combined organic layer was washed with brine and dried over anhydrous Na_2SO_4. After filtration, the solvent was removed under reduced pressure, and the residue was purified by column chromatography on silica gel to afford olefin **82** as a colorless oil (39.5 mg, 67% yield).

R_f = 0.34 (petroleum ether/ethyl acetate, 20:1).

^{1}H NMR (400 MHz, $CDCl_3$) δ 8.59 (d, *J* = 4.8 Hz, 1H), 7.57 (t, *J* = 7.7 Hz, 1H), 7.35 (d, *J* = 7.9 Hz, 1H), 7.32 – 7.26 (m, 4H), 7.20 – 7.14 (m, 1H), 7.13 – 7.11 (m, 1H), 5.88 (s, 1H), 5.38 (s, 1H), 4.23 (t, *J* = 7.4 Hz, 1H), 2.02 – 1.95 (m, 1H), 1.89 – 1.82 (m, 1H), 0.69 – 0.62 (m, 1H), 0.54 – 0.46 (m, 1H), 0.00 (s, 9H).

^{13}C NMR (101 MHz, $CDCl_3$) δ 159.5, 151.5, 148.9, 143.9, 136.3, 128.5, 128.3, 126.1, 122.1, 121.3, 115.0, 51.1, 29.6, 15.1, -1.6.

IR (KBr) ν 2952, 2922, 1584, 1466, 1247, 862, 836.

HRMS (APCI) *m/z*: Calc. For $C_{19}H_{26}NSi$ ($[M+H]^+$) 296.1829, Found 296.1821.

2.5 Control experiments

(a) Radical trapping experiment

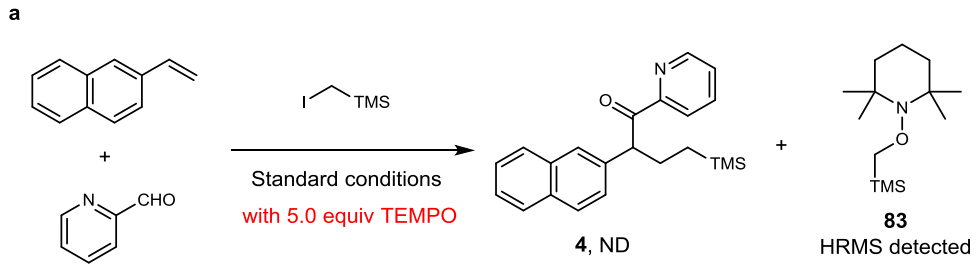

A 4 mL vial equipped with a stir bar was charged with preNHC **N1** (10.8 mg, 0.04 mmol), $Pd(OAc)_2$ (4.5 mg, 0.02 mmol), ligand **L1** (11.9 mg, 0.024 mmol) and 1.0 mL of 1,4-dioxane. After stirring for 30 min in glove box, to the solution was added Cs_2CO_3 (97.8 mg, 0.3 mmol), additive **A1** (4.4 mg, 0.04 mmol), alkenes **1** (0.2 mmol), aldehydes **2** (0.4 mmol), alkyl halides **3** (0.3 mmol), TEMPO (156.3 mg, 1.0 mmol) and 1.0 mL of 1,4-dioxane. The reaction mixtrue was removed from the glove box and stirred under 36W blue LED lights at room temperature for 48 hours. A portion of the reaction mixture was collected and annalyis by HRMS without desired ketone **4** but with the adduct **83** trapped by TEMPO detected, **HRMS** (APCI) *m/z*: Calc. For

$C_{13}H_{30}NOSi$ ([M+H]$^+$) 244.2091, Found 244.2087.

(b) Radical clock experiment with (bromomethyl)cyclopropane

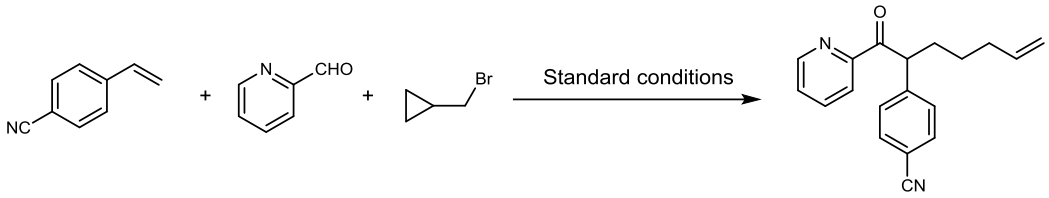

84, 47%

A 4 mL vial equipped with a stir bar was charged with preNHC **N1** (10.8 mg, 0.04 mmol), $Pd(OAc)_2$ (4.5 mg, 0.02 mmol), ligand **L1** (11.9 mg, 0.024 mmol) and 1.0 mL of 1,4-dioxane. After stirring for 30 min in glove box, to the solution was added Cs_2CO_3 (97.8 mg, 0.3 mmol), additive **A1** (4.4 mg, 0.04 mmol), strene **1m** (25.8 mg, 0.2 mmol), aldehyde **2a** (42.8mg, 0.4 mmol), alkyl halide **3y** (40.5 mg, 0.3 mmol), and 1.0 mL of 1,4-dioxane. The reaction mixtrue was removed from the glove box and stirred under 36W blue LED lights at room temperature for 48 hours. The reaction mixture was filtered through a small pad of silica and eluted with EtOAc. The solution was concentrated under reduced pressure, and purified by column chromatography on silica gel to afford the desired ketone **84** as a light yellow oil (27.2 mg, 47%).

R_f = 0.35 (petroleum ether/ethyl acetate, 10:1).

^{1}H NMR (500 MHz, CDCl3) δ 8.66 (s, 1H), 8.02 – 8.01 (m, 1H), 7.81 – 7.78 (m, 1H), 7.56 – 7.51 (m, 4H), 7.45 – 7.43 (m, 1H), 5.76 – 5.71 (m, 1H), 5.49 – 5.46 (m, 1H), 4.99 – 4.92 (m, 2H), 2.19 – 2.07 (m, 3H), 1.88 – 1.86 (m, 1H), 1.40 – 1.31 (m, 2H).

^{13}C NMR (126 MHz, $CDCl_3$) δ 200.5, 152.6, 149.1, 145.1, 138.3, 137.2, 132.4, 129.9, 127.5, 122.9, 119.0, 115.0, 110.8, 50.7, 33.7, 32.5, 27.0.

IR (KBr) ν 2925, 2228, 1697, 1435, 1087, 1042, 994.

HRMS (ESI) *m/z*: Calc. For $C_{19}H_{19}N_2O$ ($[M+H]^+$) 291.1492, Found 291.1493.

(c) Radical probe experiment with 6-bromohex-1-ene

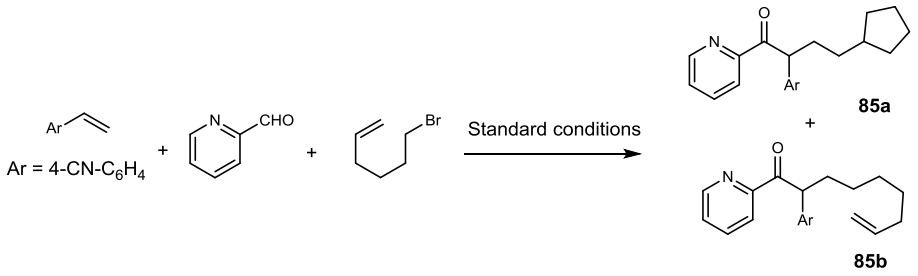

85a/85b = 5:1, total yield 42%

A 4 mL vial equipped with a stir bar was charged with preNHC **N1** (10.8 mg, 0.04 mmol), $Pd(OAc)_2$ (4.5 mg, 0.02 mmol), ligand **L1** (11.9 mg, 0.024 mmol) and 1.0 mL of 1,4-dioxane. After stirring for 30 min in glove box, to the solution was added Cs_2CO_3 (97.8 mg, 0.3 mmol), additive **A1** (4.4 mg, 0.04 mmol), strene **1m** (25.8 mg, 0.2 mmol), aldehyde **2a** (42.8mg, 0.4 mmol), alkyl halide **3z** (48.9 mg, 0.3 mmol), and 1.0 mL of 1,4-dioxane. The reaction mixtrue was removed from the glove box and stirred under 36W blue LED lights at room temperature for 48 hours. The reaction mixture was filtered through a small pad of silica and eluted with EtOAc. The solution was concentrated under reduced pressure, and purified by column chromatography on silica gel to afford the mixtrue of **85a** and **85b** as a light yellow oil (**85a**/**85b =** 5.3:1, total 26.7 mg, 42%).

R_f = 0.19 (petroleum ether/ethyl acetate, 10:1).

85a, **^{1}H NMR** (400 MHz, $CDCl_3$) δ 8.66 (d, *J* = 4.8 Hz, 1H), 8.02 (d, *J* = 7.8 Hz, 1H), 7.80 (t, *J* = 7.7 Hz, 1H), 7.57 – 7.51 (m, 4H), 7.46 – 7.43 (m, 1H), 5.45 (t, *J* = 7.3 Hz, 1H), 2.20 – 2.15 (m, 1H), 1.88 – 1.74 (m, 4H), 1.55 – 1.48 (m, 3H), 1.34 – 1.19 (m, 3H), 1.105 – 1.00 (m, 2H).

^{13}C NMR (101 MHz, $CDCl_3$) δ 200.5, 152.6, 148.9, 145.2, 137.0, 132.2, 129.8, 127.3, 122.8, 118.9, 110.6, 50.8, 38.7, 33.1, 27.7, 25.4, 22.6, 22.5.

IR (KBr) ν 2941, 2227, 1696, 994, 562.

HRMS (APCI) *m/z*: Calc. For $C_{21}H_{21}N_2O$ ([M-H]$^-$) 317.1659, Found 317.1660.

(d) Radical clock experiment with (1-(2-phenylcyclopropyl)vinyl)benzene

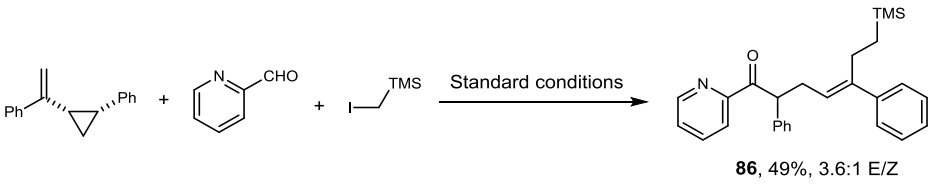

86, 49%, 3.6:1 E/Z

A 4 mL vial equipped with a stir bar was charged with preNHC **N1** (10.8 mg, 0.04 mmol), $Pd(OAc)_2$ (4.5 mg, 0.02 mmol), ligand **L1** (11.9 mg, 0.024 mmol) and 1.0 mL of 1,4-dioxane. After stirring for 30 min in glove box, to the solution was added Cs_2CO_3 (97.8 mg, 0.3 mmol), additive **A1** (4.4 mg, 0.04 mmol), strene **1aa** (44.0 mg, 0.2 mmol), aldehyde **2a** (42.8mg, 0.4 mmol), alkyl halide **3a** (42.8 mg, 0.3 mmol), and 1.0 mL of 1,4-dioxane. The reaction mixtrue was removed from the glove box and stirred under 36W blue LED lights at room temperature for 48 hours. The reaction mixture was filtered through a small pad of silica and eluted with EtOAc. The solution was concentrated under reduced pressure, and purified by column chromatography on silica gel to afford the desired ketone **86** as a light yellow oil (40.9 mg, 49%).

R_f = 0.50 (petroleum ether/ethyl acetate, 20:1).

Major: ^{1}H NMR (500 MHz, $CDCl_3$) δ 8.73 – 8.56 (m, 1H), 8.12 – 8.01 (m, 1H), 7.79 – 7.76 (m, 1H), 7.45 – 7.44 (m, , 1H), 7.42 – 7.39 (m, , 1H), 7.31 – 7.06 (m, 9H), 5.54 (q, J = 7.2 Hz, 2H), 3.05 (dt, J = 14.4, 7.2 Hz, 1H), 2.80 (dt, J = 14.9, 7.8 Hz, 1H), 2.43 (td, J = 11.4, 5.3 Hz, 2H), 0.60 – 0.45 (m, 1H), 0.45 – 0.28 (m, 1H), -0.00 (s, 9H).

^{13}C NMR (126 MHz, $CDCl_3$) δ 201.2, 153.1, 149.0, 144.6, 143.0, 139.1, 136.9, 129.1, 128.7, 128.2, 127.1, 127.0, 126.6, 126.6, 124.4, 122.8, 51.2, 32.3, 23.8, 16.1, -1.7.

IR (KBr) ν 2951, 1695, 1582, 1435, 1246, 860, 835.

HRMS (ESI) *m/z*: Calc. For $C_{27}H_{32}NOSi$ ($[M+H]^+$) 414.2248, Found 414.2248.

2.6 UV-Visible absorption analysis

UV/Vis absorption spectra were recorded on a Jasco V-650 spectrophotometer, equipped with a temperature control unit at 25 ℃. The samples were measured in Hellma fluorescence QS quartz cuvettes (chamber volume = 3.0 mL) fitted with a PTFE stopper. The UV-visible absorption of the substrates $Pd(OAc)_2$ and **L1** (10^{-4}M), strene (**1m**, 10^{-3}M), benzaldehyde (**2i**, $2x10^{-3}$M), trimethyl(iodomethyl)silane (**3a**, $1.5 x10^{-3}$M), **N1** ($2x10^{-4}$M), [**L1** (10^{-4}M) and **3a** ($1.5x10^{-3}$M)], **N1** and **2i** ($2x10^{-3}$M), [$Pd(OAc)_2$, **L1** (10^{-4}M), and **N1** ($2x10^{-4}$M)], and standard reaction mixture were determined in 1,4-dioxane.

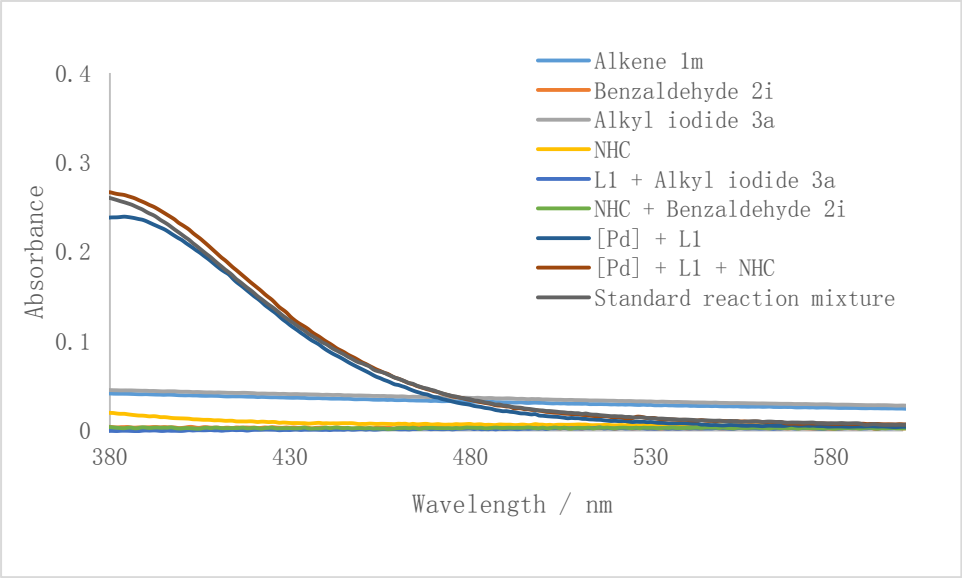

Supplementary Figure 1. UV-Visible absorption spectra.

2.7 Light on/off experiments

A 4 mL vials equipped with a stir bar separately were charged with preNHC **N1** (10.8 mg, 0.04 mmol), $Pd(OAc)_2$ (4.5 mg, 0.02 mmol), ligand **L1** (11.9 mg, 0.024 mmol) and 1.0 mL of 1,4-dioxane. After stirring for 30 min in glove box, to the solution was added Cs_2CO_3 (97.8 mg, 0.3 mmol), additive **A1** (4.4 mg, 0.04 mmol), styrene **1a** (30.8 mg, 0.2 mmol), aldehyde **2a** (42.8mg, 0.4 mmol), alkyl halide **3a** (42.8 mg, 0.3 mmol), dodecane (internal standard, 25.8mg, 0.15 mmol) and 1.0 mL of 1,4-dioxane. The reaction mixtrue was removed from the glove box and stirred under 36W blue LED lights at room temperature for 10 min, followed by 10 min in the dark, which was continued for a total reaction time of 80 min. After each sequence, an aliquot was taken and the reaction was analyzed via GC-MS analysis of the crude reaction mixture.

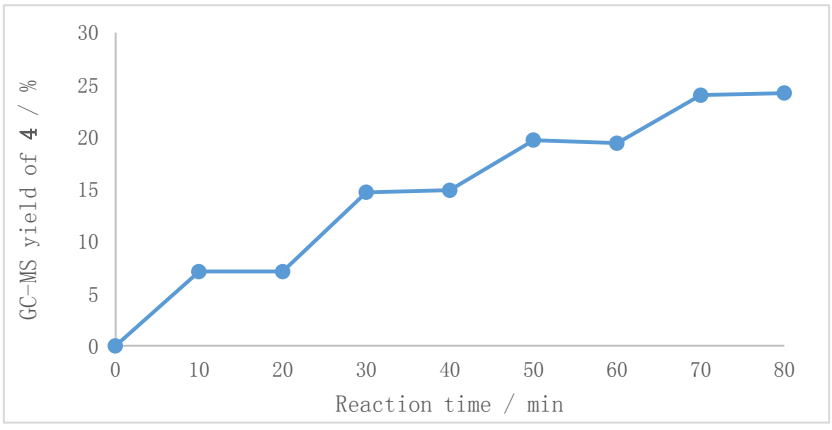

Supplementary Figure 2. Light on/off experiments

3. Supplementary Figures
3.1 NMR Spectra

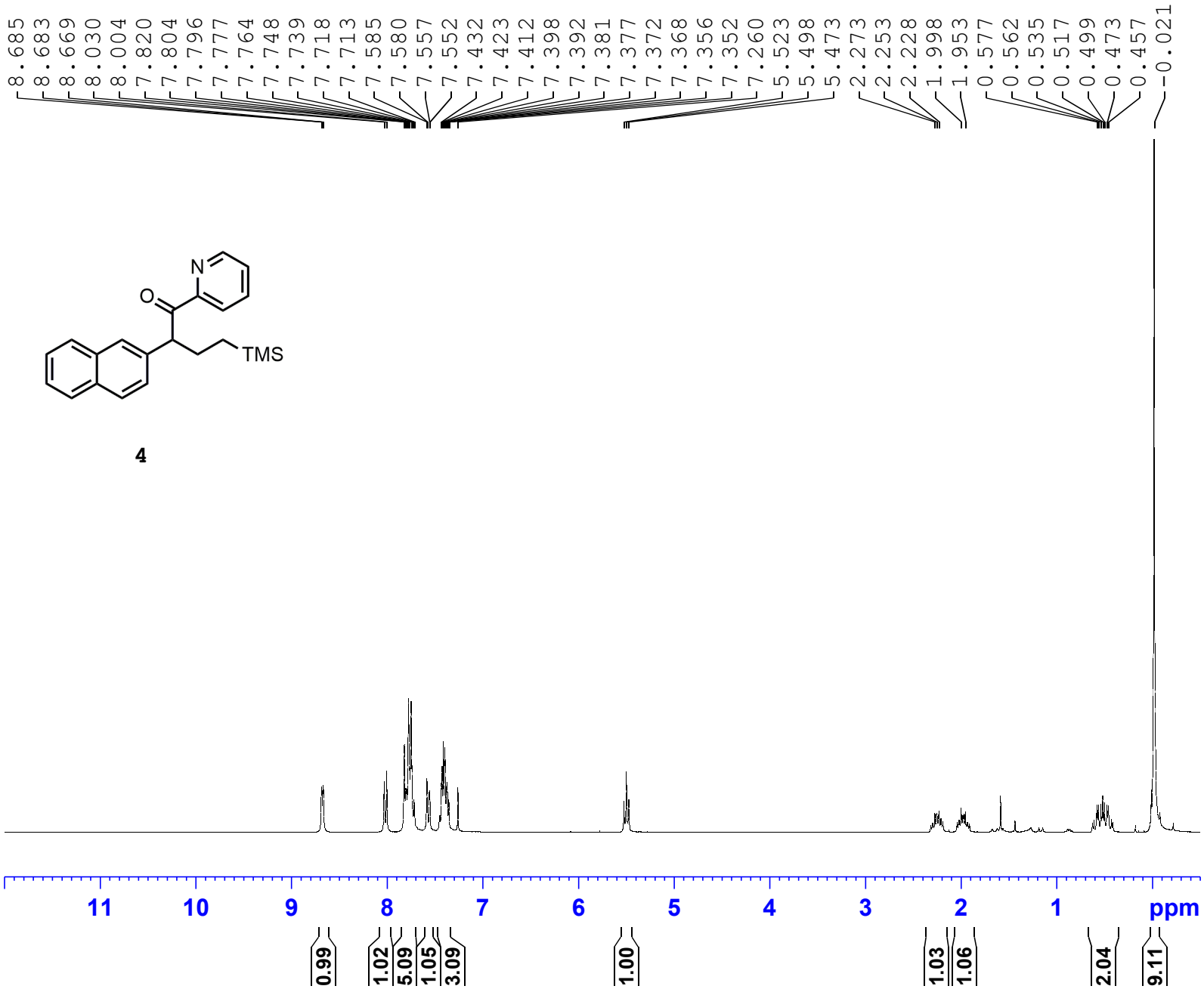


```
Current Data Parameters
NAME                  11122A
EXPNO                      1
PROCNO                     1

F2 - Acquisition Parameters
Date_              20220130
Time                  19.18
INSTRUM               spect
PROBHD   5 mm DUL 13C-1
PULPROG                zg30
TD                    65536
SOLVENT               CDCl3
NS                       12
DS                        2
SWH               6009.615 Hz
FIDRES           0.091699 Hz
AQ               5.4525952 sec
RG                   209.09
DW                   83.200 usec
DE                     6.50 usec
TE                    297.2 K
D1             1.00000000 sec
D11       0 sec
TD0                       1

======== CHANNEL f1 ========
SFO1          300.1318534 MHz
NUC1                     1H
P1                    8.00 usec
PLW1          18.00000000 W

======== CHANNEL f2 ========
SFO2          300.1318534 MHz
NUC2                   off
CPDPRG[2
PCPD2      0 usec
PLW2       0 W
PLW12      0 W
PLW13      0 W

F2 - Processing parameters
SI                    65536
SF            300.1300072 MHz
WDW                      EM
SSB        0
LB                    0.30 Hz
GB         0
PC                    1.00
```

Supplementary Figure 3. H-NMR of compound **4**, recorded at 300MHz and 25 ^{O}C in $CDCl_3$

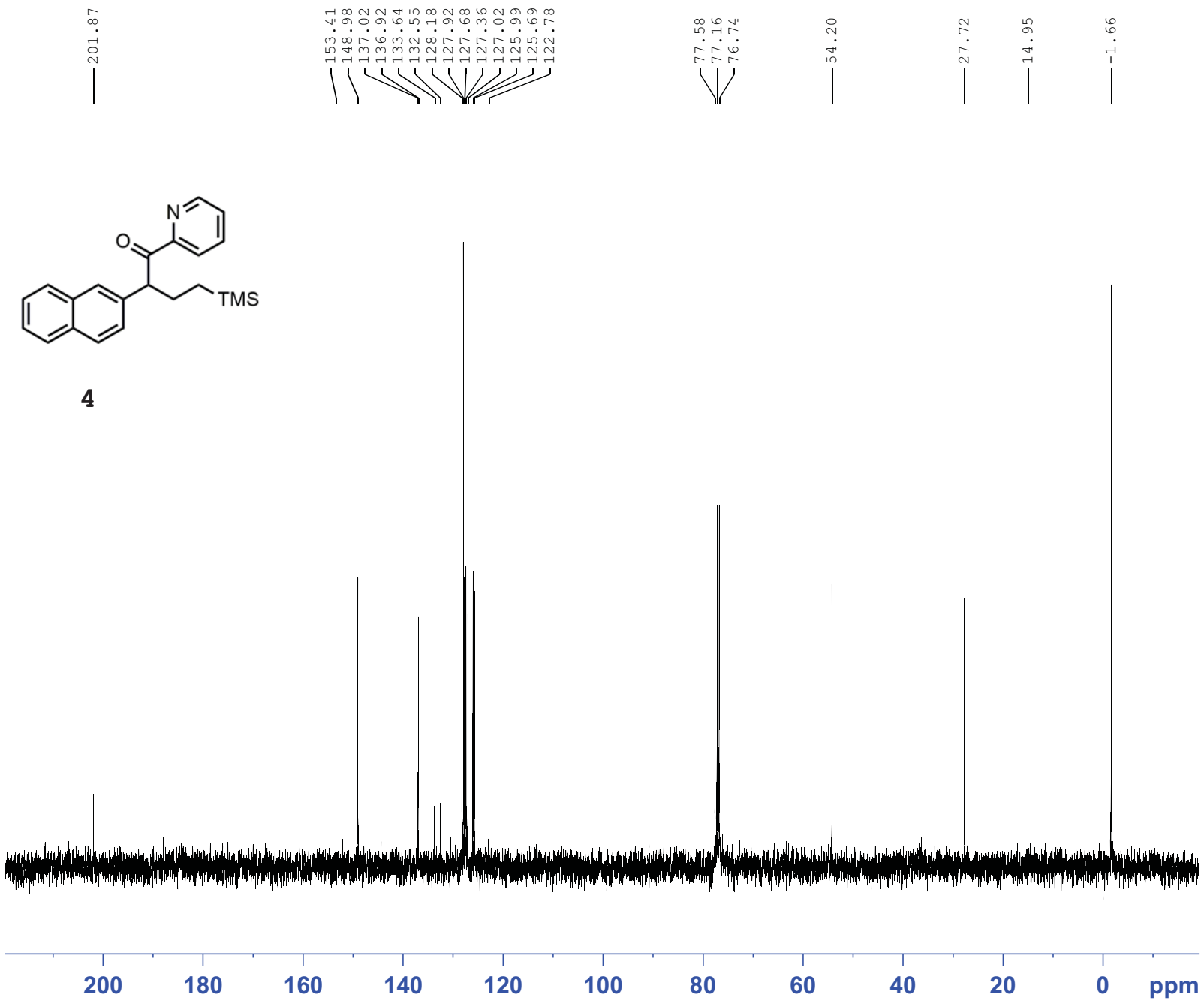


```
Current Data Parameters
NAME                 11122A
EXPNO                     2
PROCNO                    1

F2 - Acquisition Parameters
Date_              20220130
Time                 19.21
INSTRUM              spect
PROBHD    5 mm DUL 13C-1
PULPROG             zgpg30
TD                   65536
SOLVENT              CDCl3
NS                     100
DS                       4
SWH              18028.846 Hz
FIDRES            0.275098 Hz
AQ                1.8175317 sec
RG                   209.09
DW                  27.733 usec
DE                    6.50 usec
TE                   297.5 K
D1             2.00000000 sec
D11            0.03000000 sec
TD0                      1

======== CHANNEL f1 ========
SFO1            75.4752949 MHz
NUC1                   13C
P1                  11.00 usec
PLW1         195.00000000 W

======== CHANNEL f2 ========
SFO2           300.1312005 MHz
NUC2                   1H
CPDPRG[2           waltz16
PCPD2                90.00 usec
PLW2           14.00000000 W
PLW12           0.17284000 W
PLW13           0.14000000 W

F2 - Processing parameters
SI                    32768
SF              75.4677388 MHz
WDW                      EM
SSB       0
LB                    1.00 Hz
GB        0
PC                    1.40
```

Supplementary Figure 4. ^{13}C-NMR of compound **4**, recorded at 75 MHz and 25 °C in $CDCl_3$.

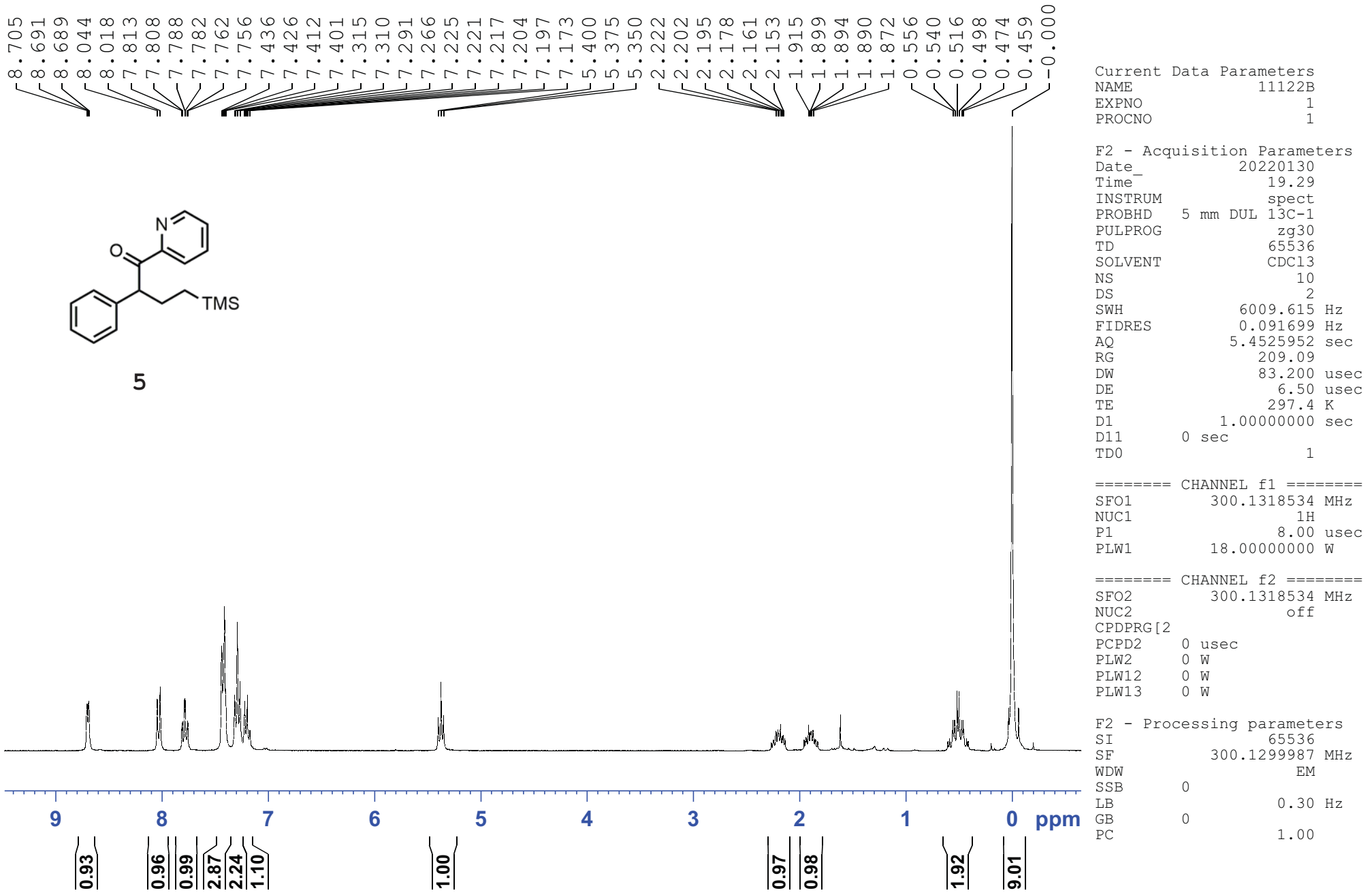


```
Current Data Parameters
NAME                  11122B
EXPNO                      1
PROCNO                     1

F2 - Acquisition Parameters
Date_              20220130
Time                  19.29
INSTRUM               spect
PROBHD    5 mm DUL 13C-1
PULPROG                zg30
TD                    65536
SOLVENT               CDCl3
NS                       10
DS                        2
SWH               6009.615 Hz
FIDRES           0.091699 Hz
AQ               5.4525952 sec
RG                   209.09
DW                   83.200 usec
DE                     6.50 usec
TE                    297.4 K
D1             1.00000000 sec
D11       0 sec
TD0                       1

======== CHANNEL f1 ========
SFO1          300.1318534 MHz
NUC1                     1H
P1                    8.00 usec
PLW1          18.00000000 W

======== CHANNEL f2 ========
SFO2          300.1318534 MHz
NUC2                   off
CPDPRG[2
PCPD2      0 usec
PLW2       0 W
PLW12      0 W
PLW13      0 W

F2 - Processing parameters
SI                    65536
SF          300.1299987 MHz
WDW                      EM
SSB        0
LB                    0.30 Hz
GB         0
PC                    1.00
```

Supplementary Figure 5. ^{1}H-NMR of compound **5**, recorded at 300 MHz and 25 °C in $CDCl_3$.

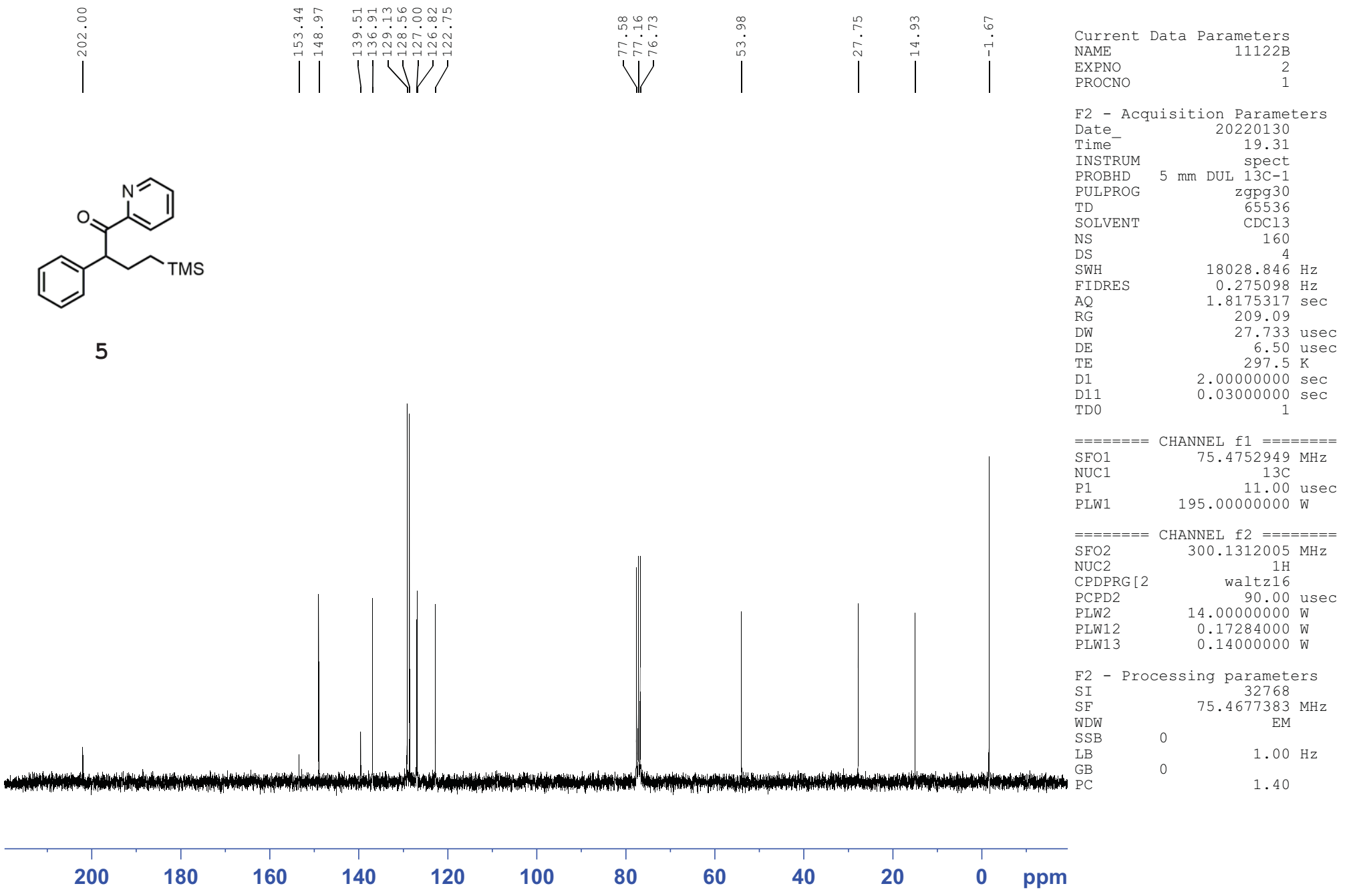

Supplementary Figure 6. ^{13}C-NMR of compound **5**, recorded at 75 MHz and 25 °C in $CDCl_3$.

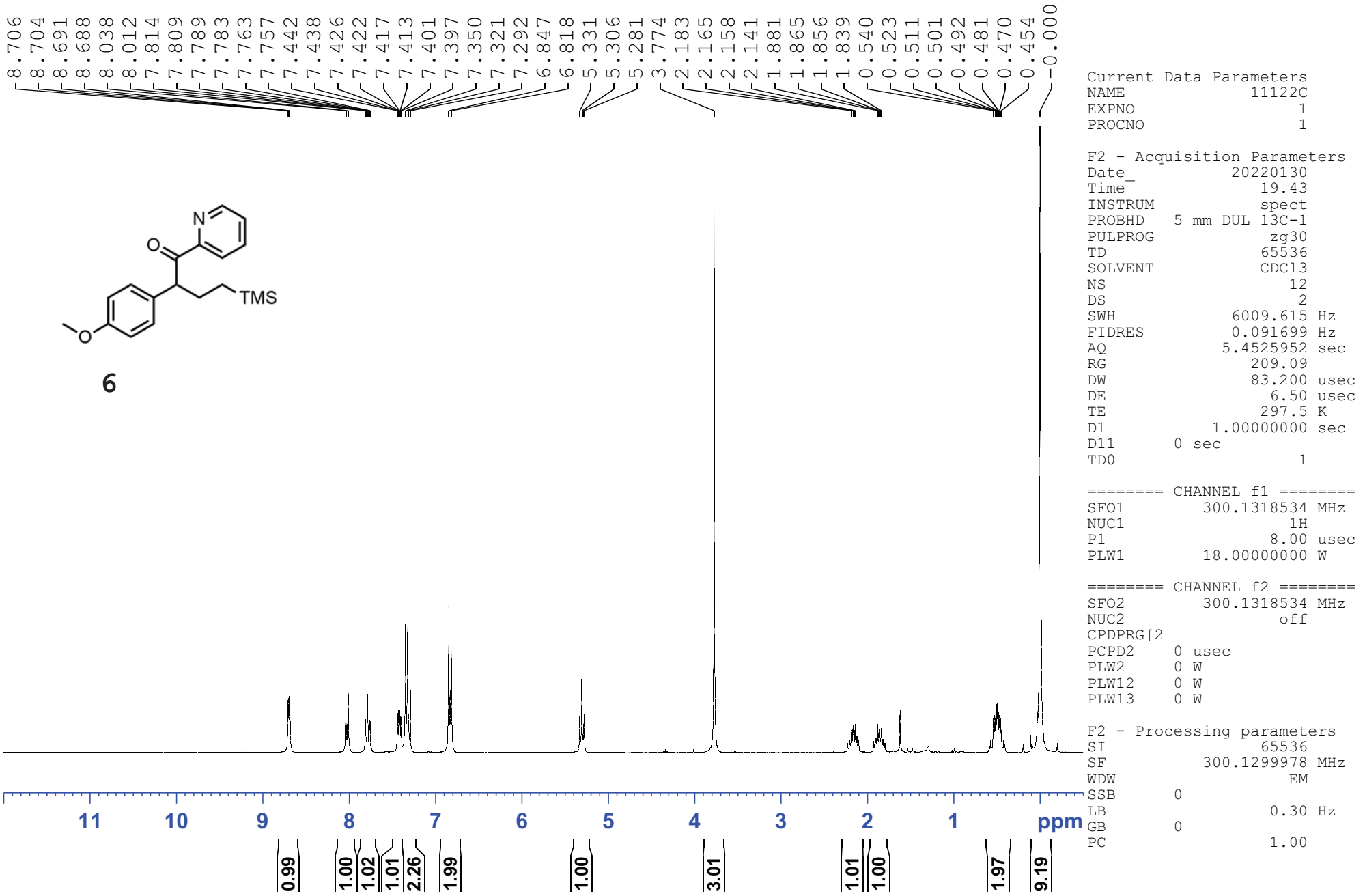

Supplementary Figure 7. ^{1}H-NMR of compound **6**, recorded at 300 MHz and 25 °C in $CDCl_3$.

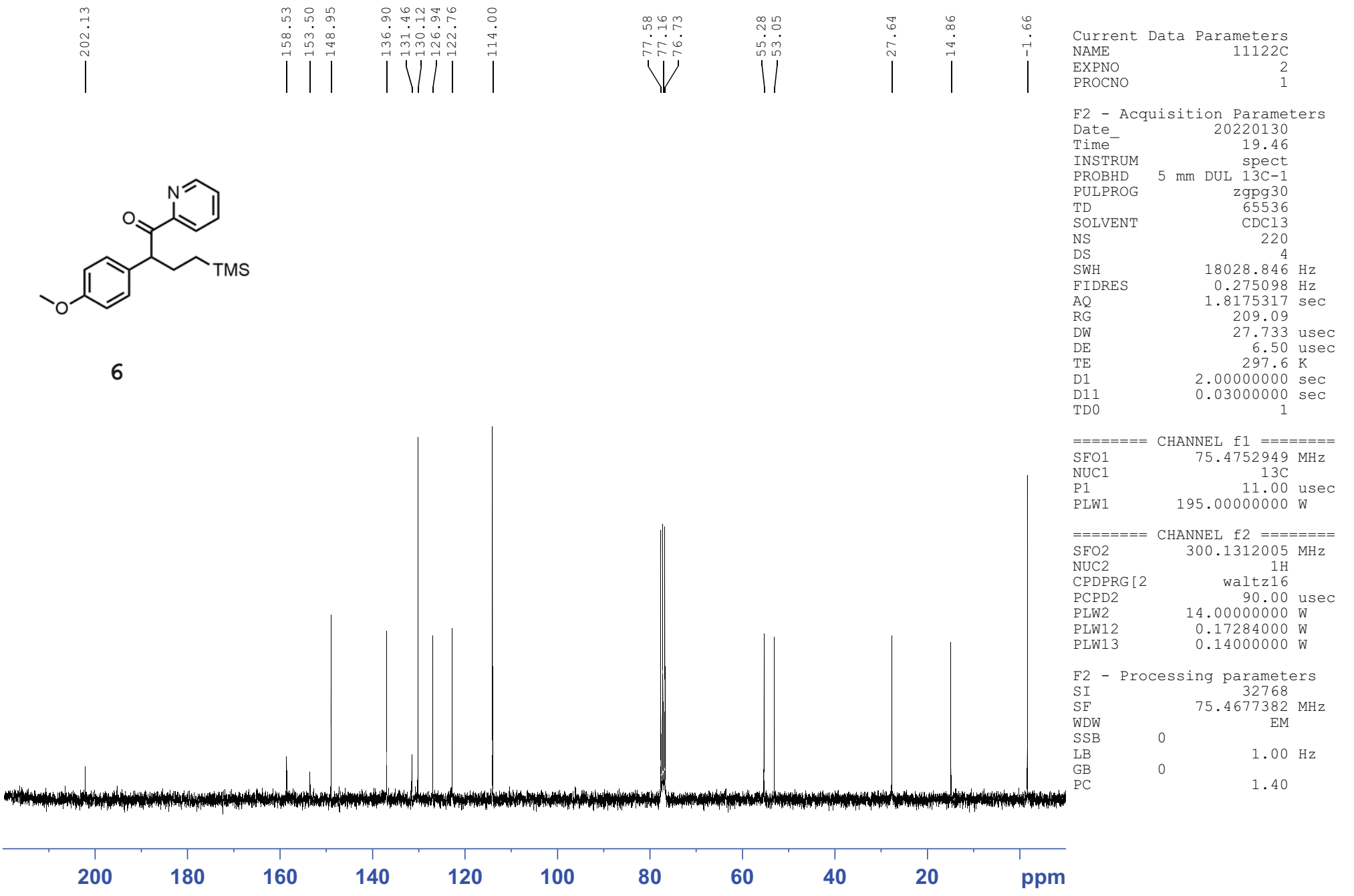


```
Current Data Parameters
NAME                 11122C
EXPNO                     2
PROCNO                    1

F2 - Acquisition Parameters
Date_              20220130
Time                  19.46
INSTRUM               spect
PROBHD   5 mm DUL 13C-1
PULPROG              zgpg30
TD                    65536
SOLVENT               CDCl3
NS                      220
DS                        4
SWH              18028.846 Hz
FIDRES            0.275098 Hz
AQ                1.8175317 sec
RG                   209.09
DW                   27.733 usec
DE                    6.50 usec
TE                   297.6 K
D1              2.00000000 sec
D11             0.03000000 sec
TD0                       1

======== CHANNEL f1 ========
SFO1            75.4752949 MHz
NUC1                    13C
P1                   11.00 usec
PLW1         195.00000000 W

======== CHANNEL f2 ========
SFO2           300.1312005 MHz
NUC2                    1H
CPDPRG[2           waltz16
PCPD2                90.00 usec
PLW2           14.00000000 W
PLW12           0.17284000 W
PLW13           0.14000000 W

F2 - Processing parameters
SI                    32768
SF              75.4677382 MHz
WDW                      EM
SSB         0
LB                    1.00 Hz
GB          0
PC                    1.40
```

Supplementary Figure 8. ^{13}C-NMR of compound **6**, recorded at 75 MHz and 25 °C in $CDCl_3$.

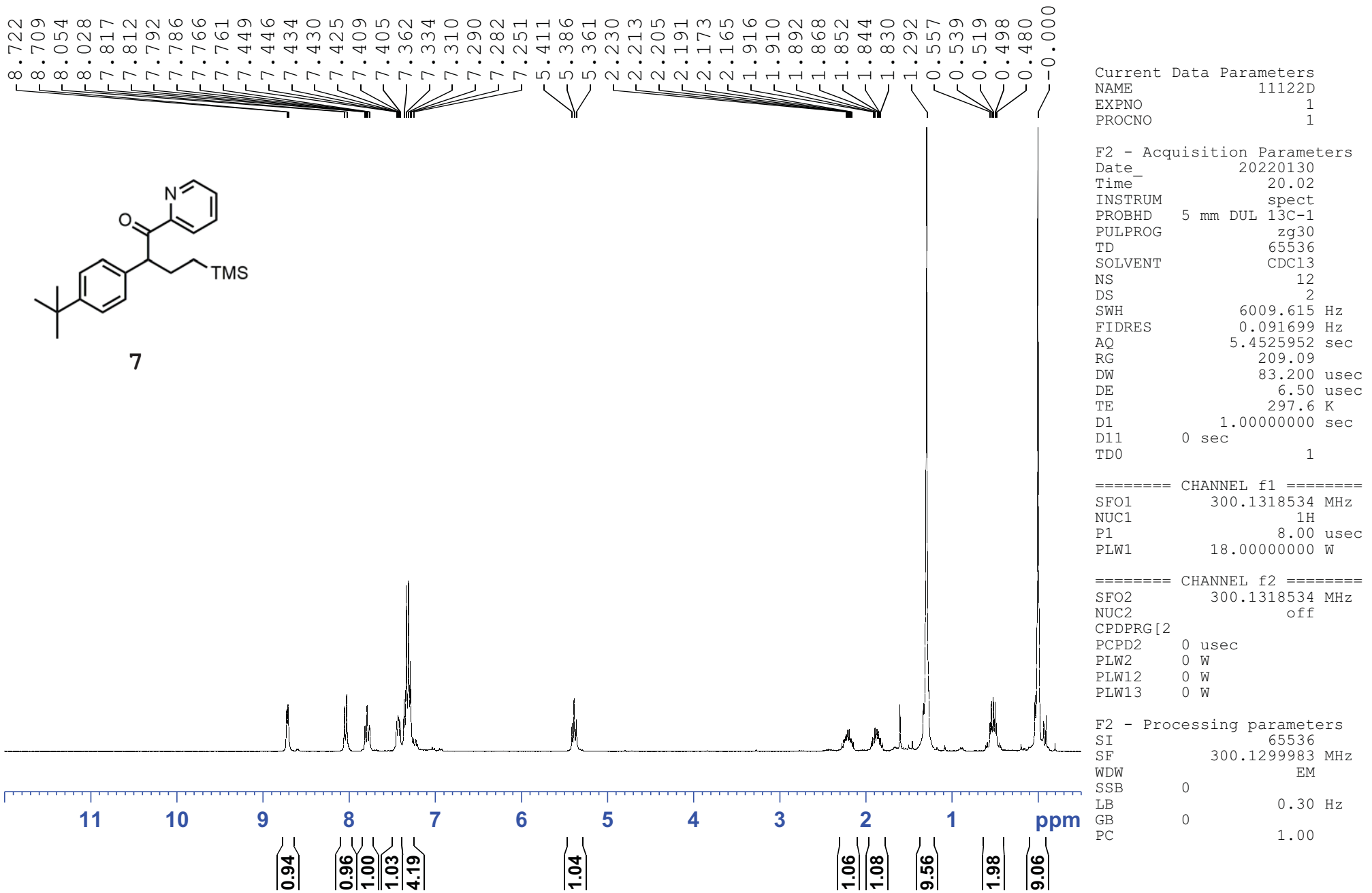


```
Current Data Parameters
NAME                  11122D
EXPNO                      1
PROCNO                     1

F2 - Acquisition Parameters
Date_              20220130
Time                 20.02
INSTRUM              spect
PROBHD    5 mm DUL 13C-1
PULPROG              zg30
TD                  65536
SOLVENT             CDCl3
NS                     12
DS                      2
SWH              6009.615 Hz
FIDRES           0.091699 Hz
AQ               5.4525952 sec
RG                  209.09
DW                  83.200 usec
DE                    6.50 usec
TE                   297.6 K
D1             1.00000000 sec
D11       0 sec
TD0                        1

======== CHANNEL f1 ========
SFO1          300.1318534 MHz
NUC1                     1H
P1                    8.00 usec
PLW1          18.00000000 W

======== CHANNEL f2 ========
SFO2          300.1318534 MHz
NUC2                   off
CPDPRG[2
PCPD2      0 usec
PLW2       0 W
PLW12      0 W
PLW13      0 W

F2 - Processing parameters
SI                    65536
SF            300.1299983 MHz
WDW                      EM
SSB        0
LB                    0.30 Hz
GB         0
PC                    1.00
```

Supplementary Figure 9. ^{1}H-NMR of compound **7**, recorded at 300 MHz and 25 °C in $CDCl_3$.

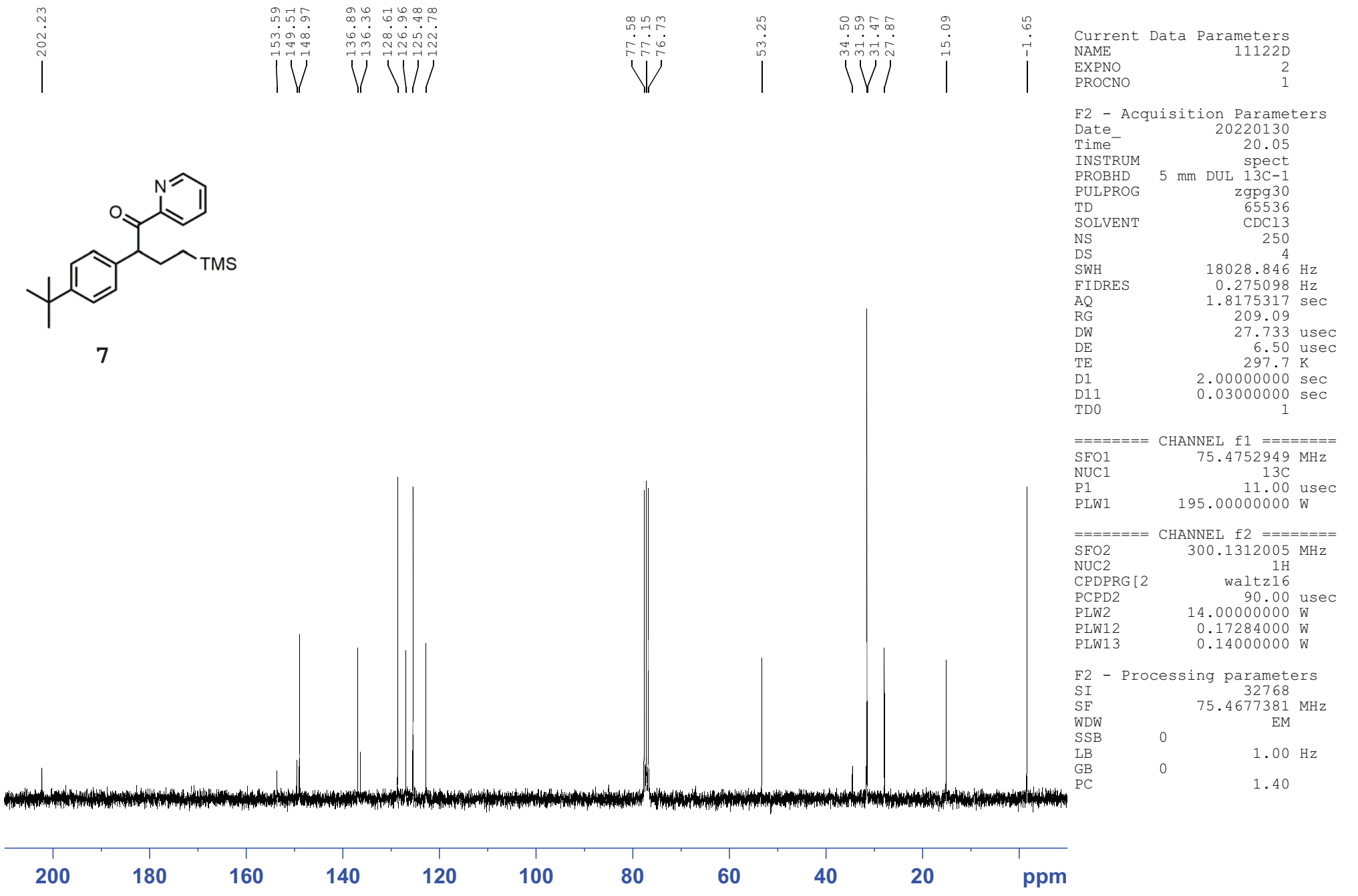


```
Current Data Parameters
NAME                 11122D
EXPNO                     2
PROCNO                    1

F2 - Acquisition Parameters
Date_              20220130
Time                 20.05
INSTRUM              spect
PROBHD    5 mm DUL 13C-1
PULPROG             zgpg30
TD                   65536
SOLVENT              CDCl3
NS                     250
DS                       4
SWH              18028.846 Hz
FIDRES            0.275098 Hz
AQ                1.8175317 sec
RG                   209.09
DW                  27.733 usec
DE                    6.50 usec
TE                   297.7 K
D1              2.00000000 sec
D11             0.03000000 sec
TD0                      1

======== CHANNEL f1 ========
SFO1            75.4752949 MHz
NUC1                   13C
P1                  11.00 usec
PLW1         195.00000000 W

======== CHANNEL f2 ========
SFO2           300.1312005 MHz
NUC2                   1H
CPDPRG[2           waltz16
PCPD2                90.00 usec
PLW2           14.00000000 W
PLW12           0.17284000 W
PLW13           0.14000000 W

F2 - Processing parameters
SI                    32768
SF              75.4677381 MHz
WDW                      EM
SSB       0
LB                    1.00 Hz
GB        0
PC                    1.40
```

Supplementary Figure 10. ^{13}C-NMR of compound **7**, recorded at 75 MHz and 25 °C in $CDCl_3$.

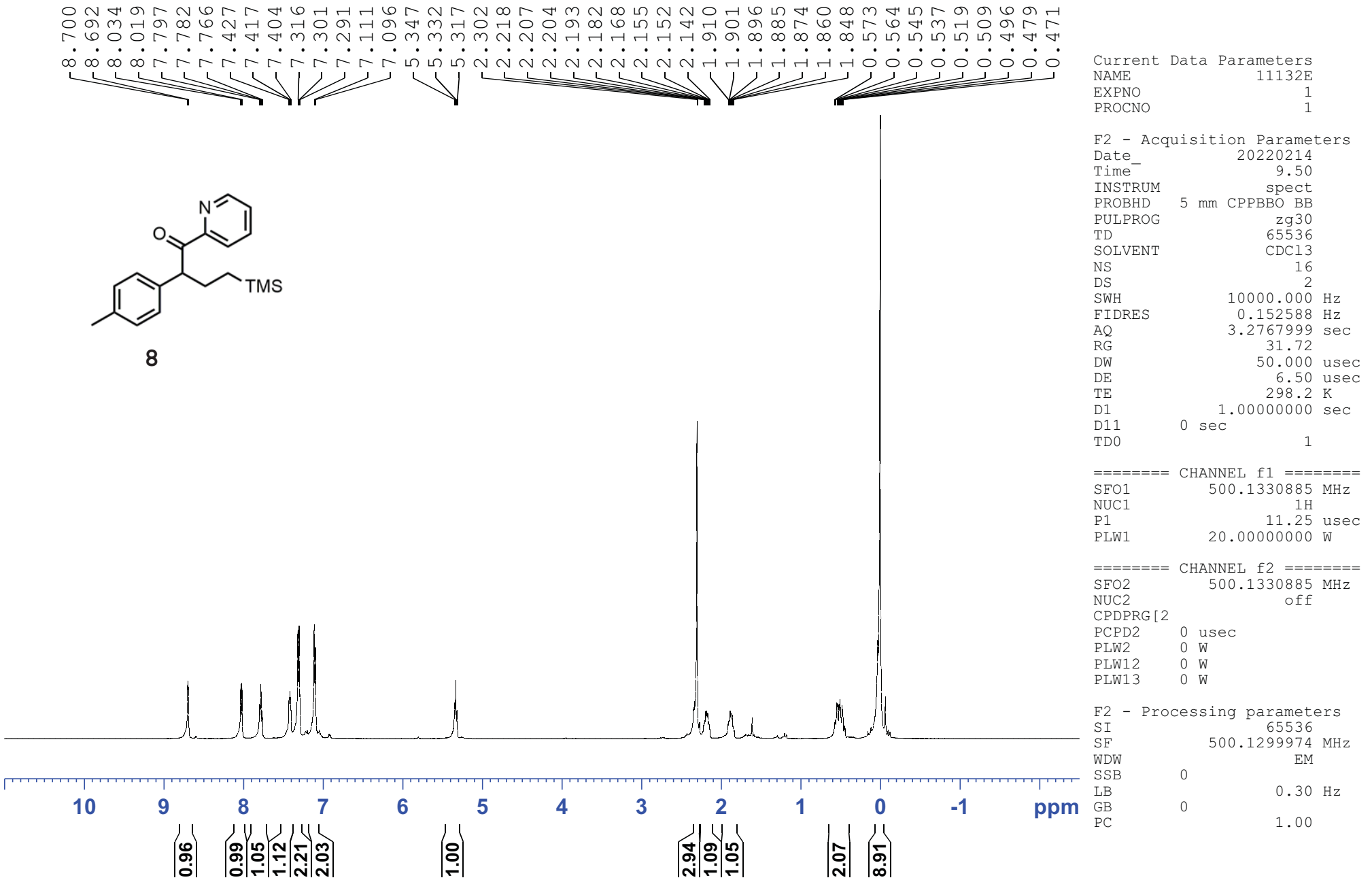


```
Current Data Parameters
NAME                  11132E
EXPNO                      1
PROCNO                     1

F2 - Acquisition Parameters
Date_              20220214
Time                   9.50
INSTRUM               spect
PROBHD    5 mm CPPBBO BB
PULPROG                zg30
TD                    65536
SOLVENT               CDCl3
NS                       16
DS                        2
SWH              10000.000 Hz
FIDRES            0.152588 Hz
AQ               3.2767999 sec
RG                    31.72
DW                  50.000 usec
DE                   6.50 usec
TE                   298.2 K
D1              1.00000000 sec
D11       0 sec
TD0                       1

======== CHANNEL f1 ========
SFO1           500.1330885 MHz
NUC1                     1H
P1                   11.25 usec
PLW1           20.00000000 W

======== CHANNEL f2 ========
SFO2           500.1330885 MHz
NUC2                    off
CPDPRG[2
PCPD2      0 usec
PLW2       0 W
PLW12      0 W
PLW13      0 W

F2 - Processing parameters
SI                    65536
SF           500.1299974 MHz
WDW                      EM
SSB        0
LB                    0.30 Hz
GB         0
PC                    1.00
```

Supplementary Figure 11. ^{1}H-NMR of compound **8**, recorded at 500 MHz and 25 °C in $CDCl_3$.

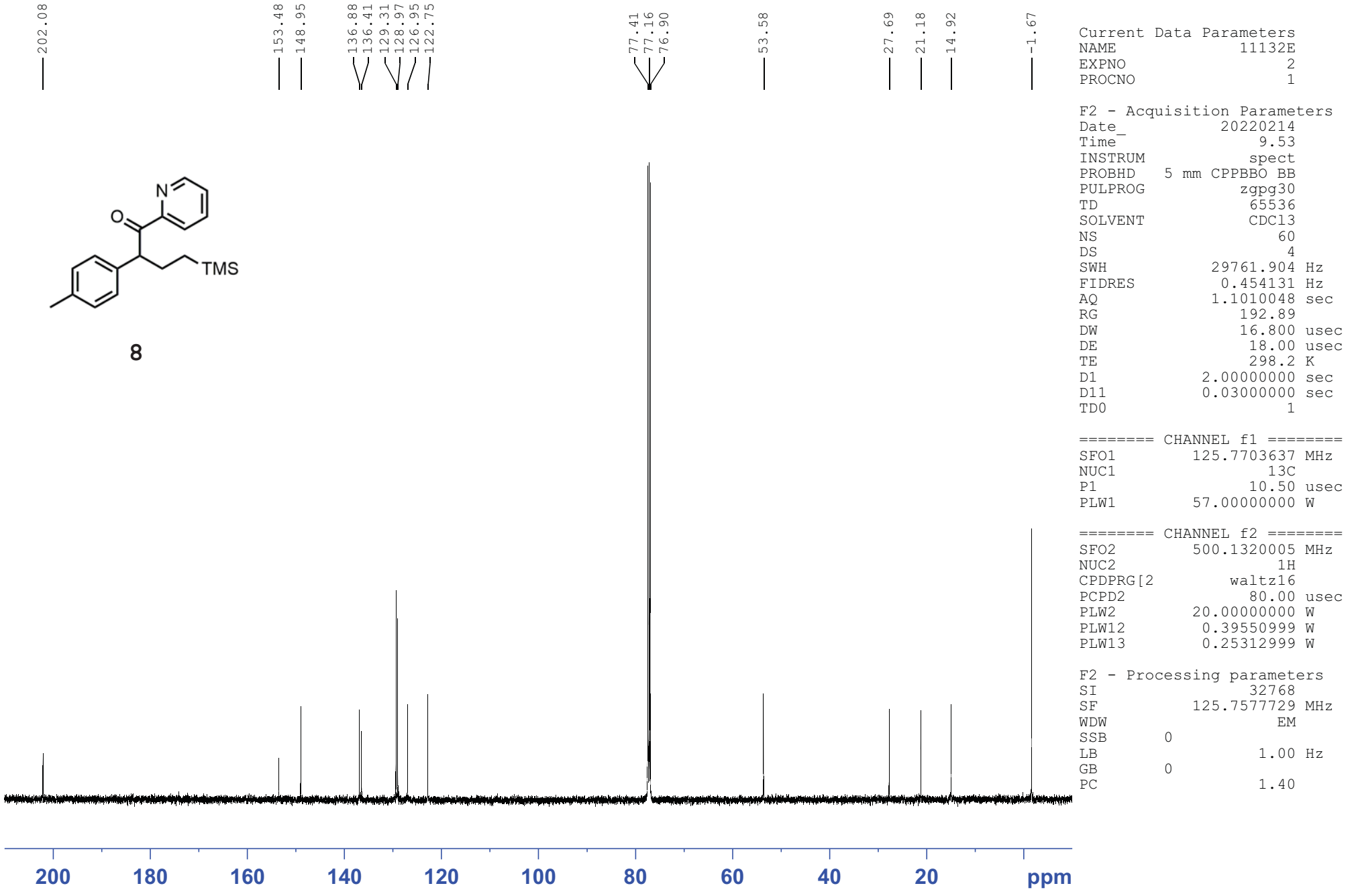


```
Current Data Parameters
NAME                  11132E
EXPNO                      2
PROCNO                     1

F2 - Acquisition Parameters
Date_              20220214
Time                   9.53
INSTRUM               spect
PROBHD   5 mm CPPBBO BB
PULPROG              zgpg30
TD                    65536
SOLVENT               CDCl3
NS                       60
DS                        4
SWH              29761.904 Hz
FIDRES            0.454131 Hz
AQ                1.1010048 sec
RG                   192.89
DW                   16.800 usec
DE                   18.00 usec
TE                   298.2 K
D1              2.00000000 sec
D11             0.03000000 sec
TD0                       1

======== CHANNEL f1 ========
SFO1           125.7703637 MHz
NUC1                    13C
P1                   10.50 usec
PLW1           57.00000000 W

======== CHANNEL f2 ========
SFO2           500.1320005 MHz
NUC2                    1H
CPDPRG[2           waltz16
PCPD2                80.00 usec
PLW2           20.00000000 W
PLW12           0.39550999 W
PLW13           0.25312999 W

F2 - Processing parameters
SI                    32768
SF             125.7577729 MHz
WDW                      EM
SSB          0
LB                    1.00 Hz
GB           0
PC                    1.40
```

Supplementary Figure 12. ^{13}C-NMR of compound **8**, recorded at 126 MHz and 25 °C in $CDCl_3$.

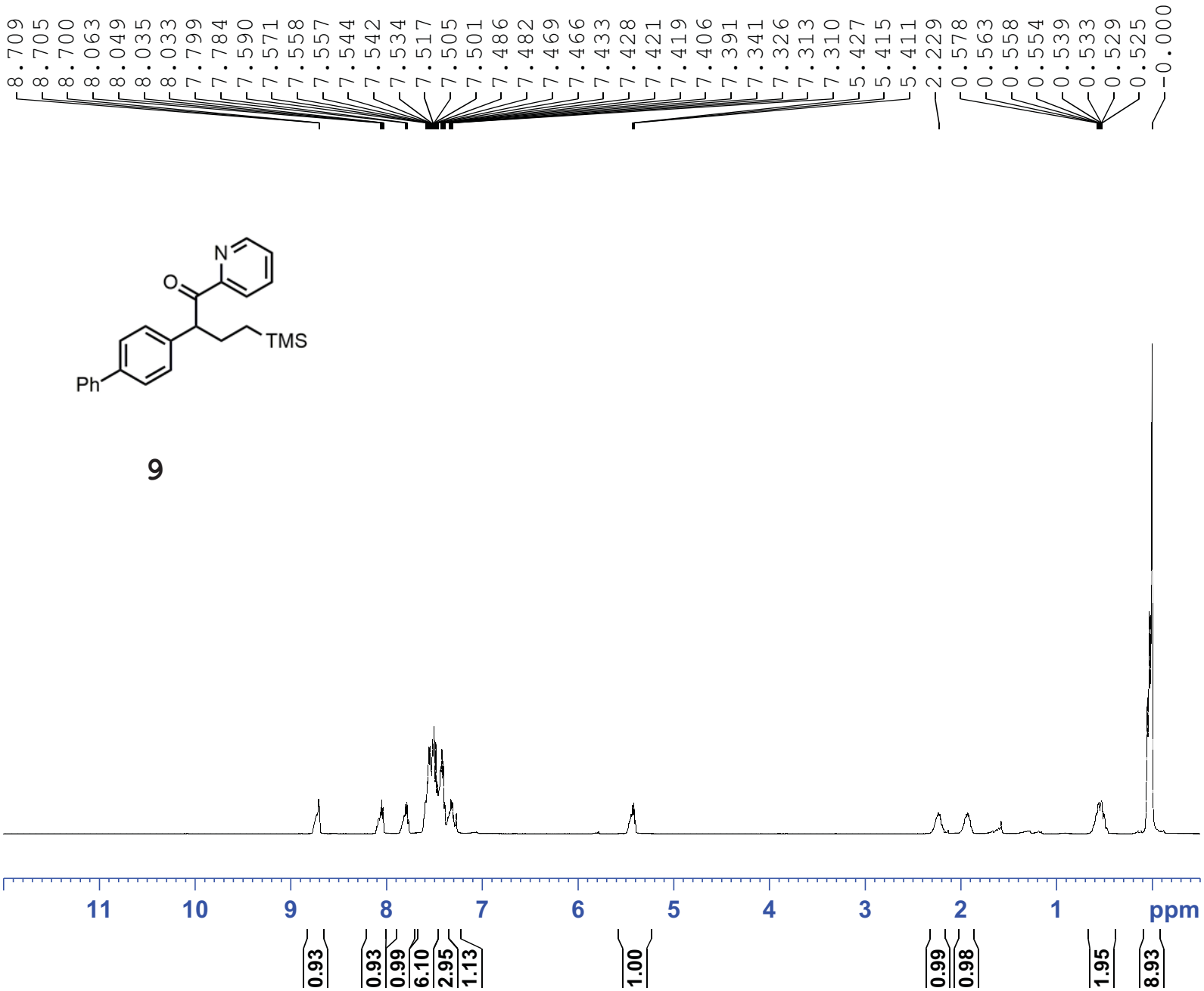


```
Current Data Parameters
NAME                  11132H
EXPNO                      1
PROCNO                     1

F2 - Acquisition Parameters
Date_              20220214
Time                  10.07
INSTRUM               spect
PROBHD    5 mm CPPBBO BB
PULPROG                zg30
TD                    65536
SOLVENT               CDCl3
NS                       16
DS                        2
SWH              10000.000 Hz
FIDRES               0.152588 Hz
AQ                3.2767999 sec
RG                    49.27
DW                   50.000 usec
DE                    6.50 usec
TE                   298.2 K
D1             1.00000000 sec
D11       0 sec
TD0                       1

======== CHANNEL f1 ========
SFO1           500.1330885 MHz
NUC1                      1H
P1                    11.25 usec
PLW1           20.00000000 W

======== CHANNEL f2 ========
SFO2           500.1330885 MHz
NUC2                    off
CPDPRG[2
PCPD2      0 usec
PLW2       0 W
PLW12      0 W
PLW13      0 W

F2 - Processing parameters
SI                    65536
SF           500.1300076 MHz
WDW                     EM
SSB        0
LB                   0.30 Hz
GB         0
PC                    1.00
```

Supplementary Figure 13. ^{1}H-NMR of compound **9**, recorded at 500 MHz and 25 °C in $CDCl_3$.

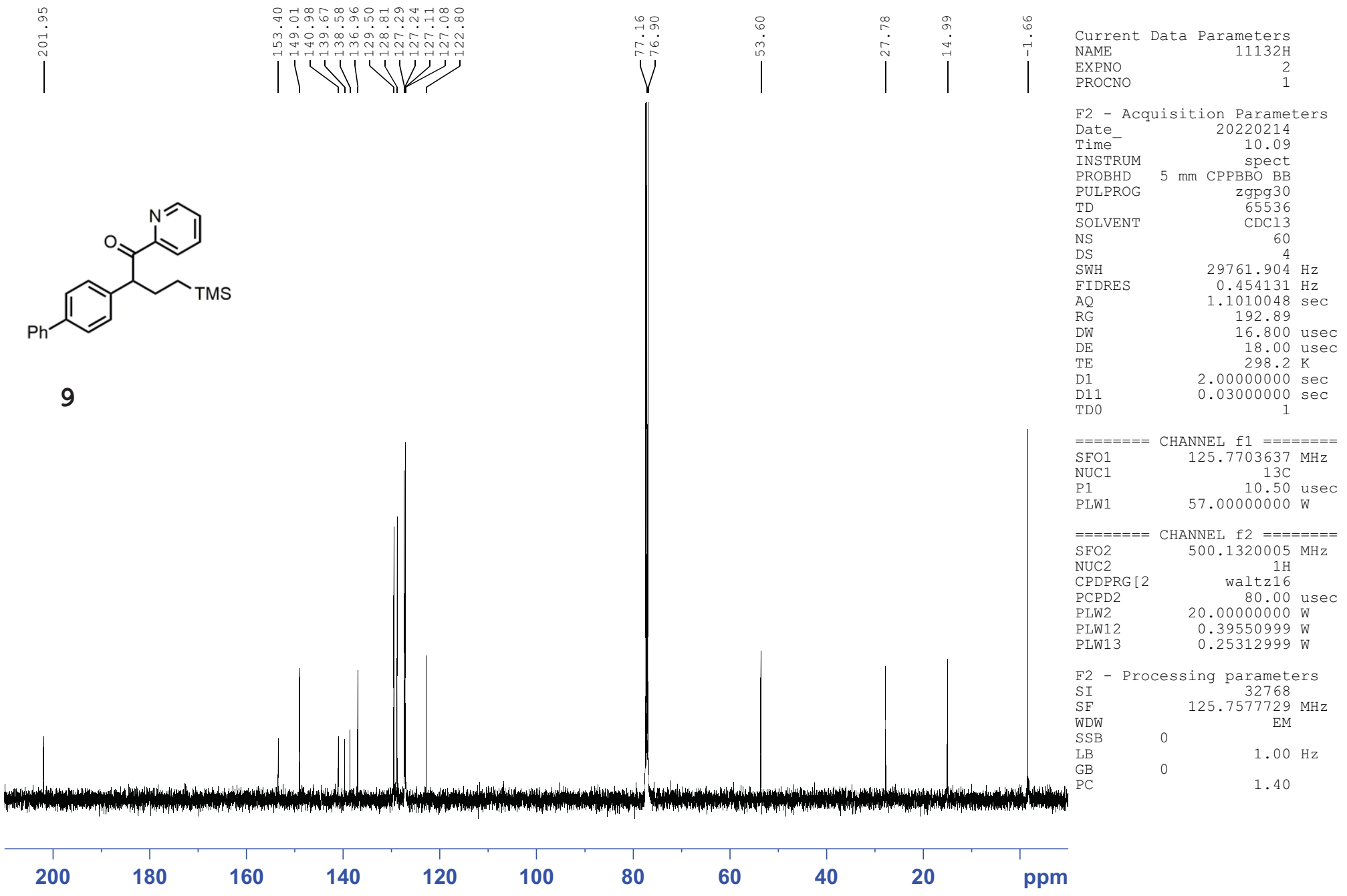


```
Current Data Parameters
NAME                 11132H
EXPNO                     2
PROCNO                    1

F2 - Acquisition Parameters
Date_              20220214
Time                 10.09
INSTRUM              spect
PROBHD   5 mm CPPBBO BB
PULPROG              zgpg30
TD                    65536
SOLVENT               CDCl3
NS                       60
DS                        4
SWH              29761.904 Hz
FIDRES            0.454131 Hz
AQ                1.1010048 sec
RG                   192.89
DW                   16.800 usec
DE                   18.00 usec
TE                   298.2 K
D1              2.00000000 sec
D11             0.03000000 sec
TD0                       1

======== CHANNEL f1 ========
SFO1           125.7703637 MHz
NUC1                    13C
P1                   10.50 usec
PLW1           57.00000000 W

======== CHANNEL f2 ========
SFO2           500.1320005 MHz
NUC2                    1H
CPDPRG[2           waltz16
PCPD2                80.00 usec
PLW2           20.00000000 W
PLW12           0.39550999 W
PLW13           0.25312999 W

F2 - Processing parameters
SI                    32768
SF             125.7577729 MHz
WDW                      EM
SSB          0
LB                    1.00 Hz
GB           0
PC                    1.40
```

Supplementary Figure 14. ^{13}C-NMR of compound **9**, recorded at 126 MHz and 25 °C in $CDCl_3$.

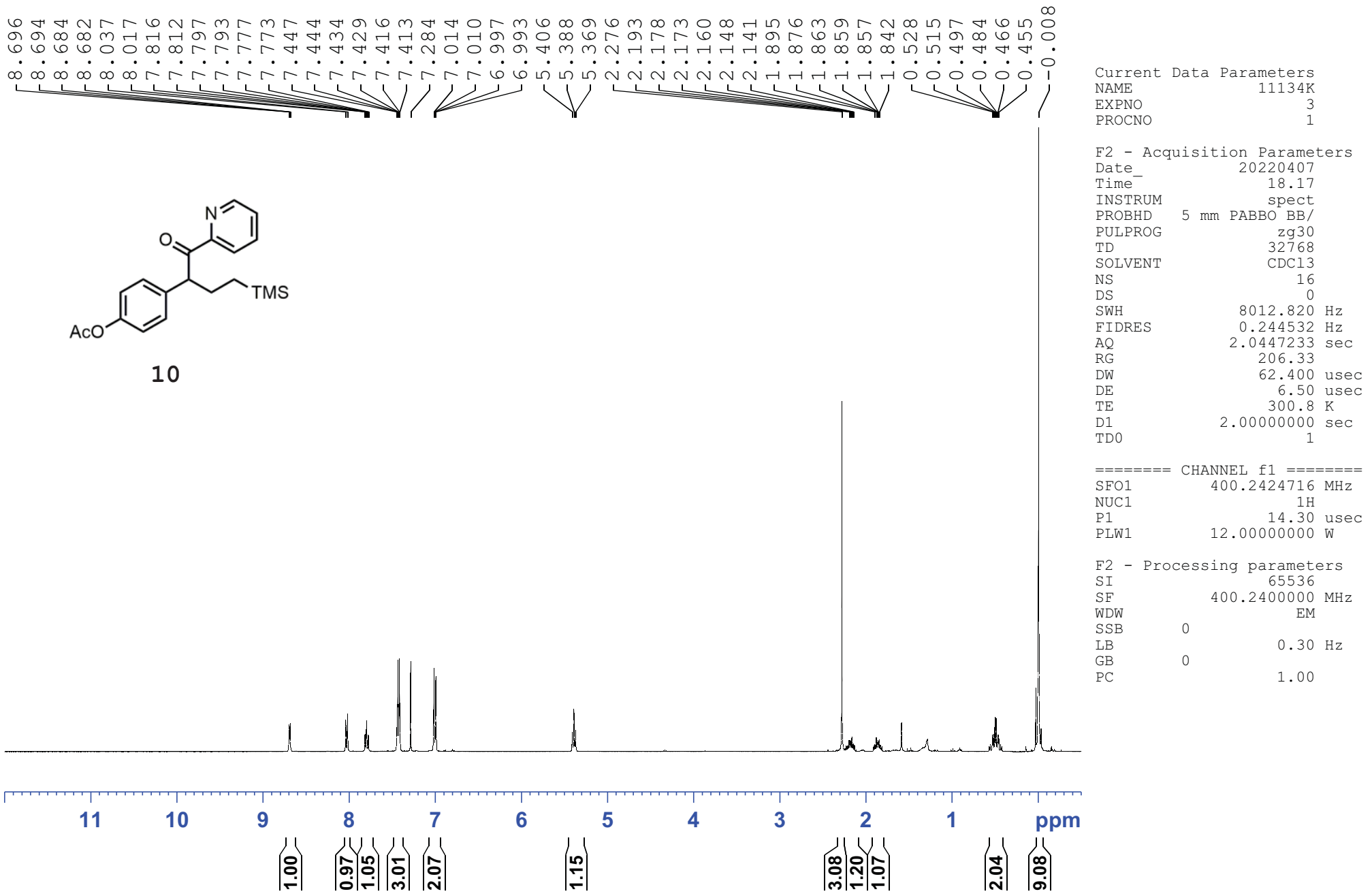


```
Current Data Parameters
NAME                  11134K
EXPNO                      3
PROCNO                     1

F2 - Acquisition Parameters
Date_              20220407
Time                  18.17
INSTRUM               spect
PROBHD    5 mm PABBO BB/
PULPROG                zg30
TD                    32768
SOLVENT               CDCl3
NS                       16
DS                        0
SWH                8012.820 Hz
FIDRES             0.244532 Hz
AQ                2.0447233 sec
RG                   206.33
DW                   62.400 usec
DE                    6.50 usec
TE                   300.8 K
D1              2.00000000 sec
TD0                       1

======== CHANNEL f1 ========
SFO1           400.2424716 MHz
NUC1                     1H
P1                    14.30 usec
PLW1           12.00000000 W

F2 - Processing parameters
SI                    65536
SF             400.2400000 MHz
WDW                      EM
SSB       0
LB                    0.30 Hz
GB        0
PC                    1.00
```

Supplementary Figure 15. ^{1}H-NMR of compound **10**, recorded at 400 MHz and 25 °C in $CDCl_3$.

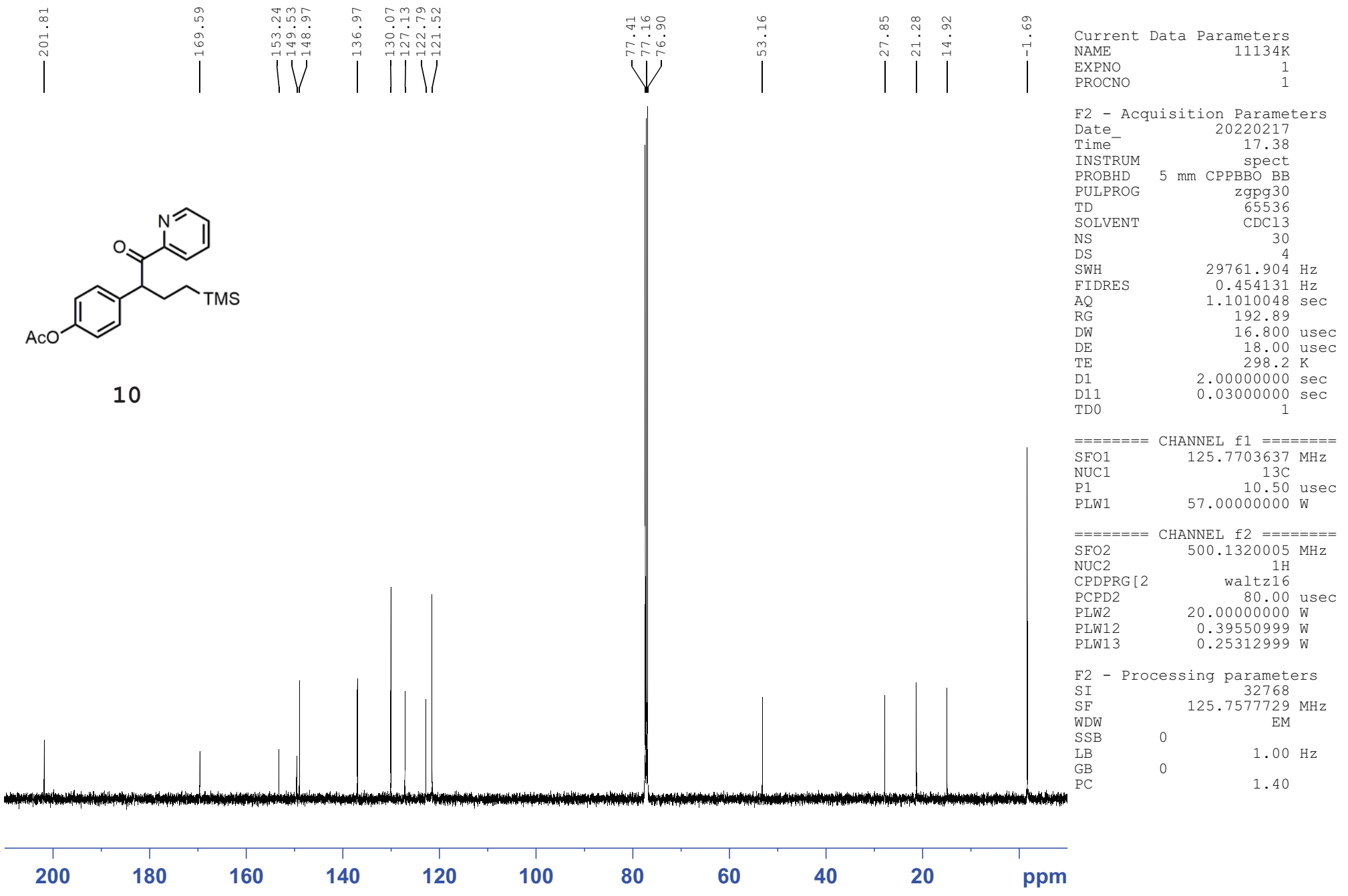

Supplementary Figure 16. ^{13}C-NMR of compound **10**, recorded at 126 MHz and 25 °C in $CDCl_3$.

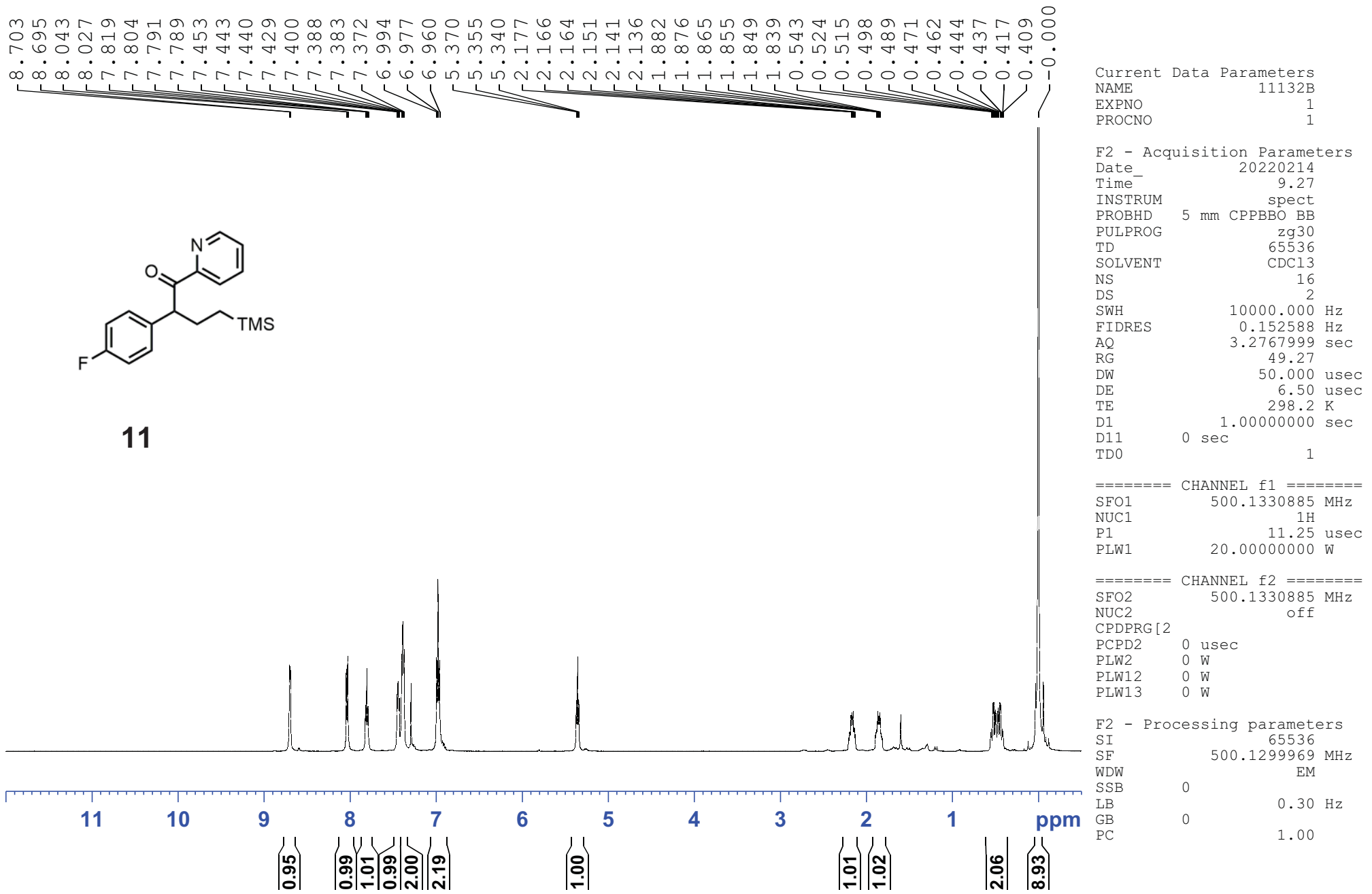


```
Current Data Parameters
NAME                  11132B
EXPNO                      1
PROCNO                     1

F2 - Acquisition Parameters
Date_              20220214
Time                   9.27
INSTRUM               spect
PROBHD   5 mm CPPBBO BB
PULPROG                zg30
TD                    65536
SOLVENT               CDCl3
NS                       16
DS                        2
SWH              10000.000 Hz
FIDRES            0.152588 Hz
AQ               3.2767999 sec
RG                    49.27
DW                   50.000 usec
DE                    6.50 usec
TE                   298.2 K
D1              1.00000000 sec
D11       0 sec
TD0                        1

======== CHANNEL f1 ========
SFO1           500.1330885 MHz
NUC1                      1H
P1                    11.25 usec
PLW1           20.00000000 W

======== CHANNEL f2 ========
SFO2           500.1330885 MHz
NUC2                     off
CPDPRG[2
PCPD2      0 usec
PLW2       0 W
PLW12      0 W
PLW13      0 W

F2 - Processing parameters
SI                    65536
SF               500.1299969 MHz
WDW                      EM
SSB        0
LB                    0.30 Hz
GB         0
PC                    1.00
```

Supplementary Figure 17. ^{1}H-NMR of compound **11**, recorded at 500 MHz and 25 °C in $CDCl_3$.

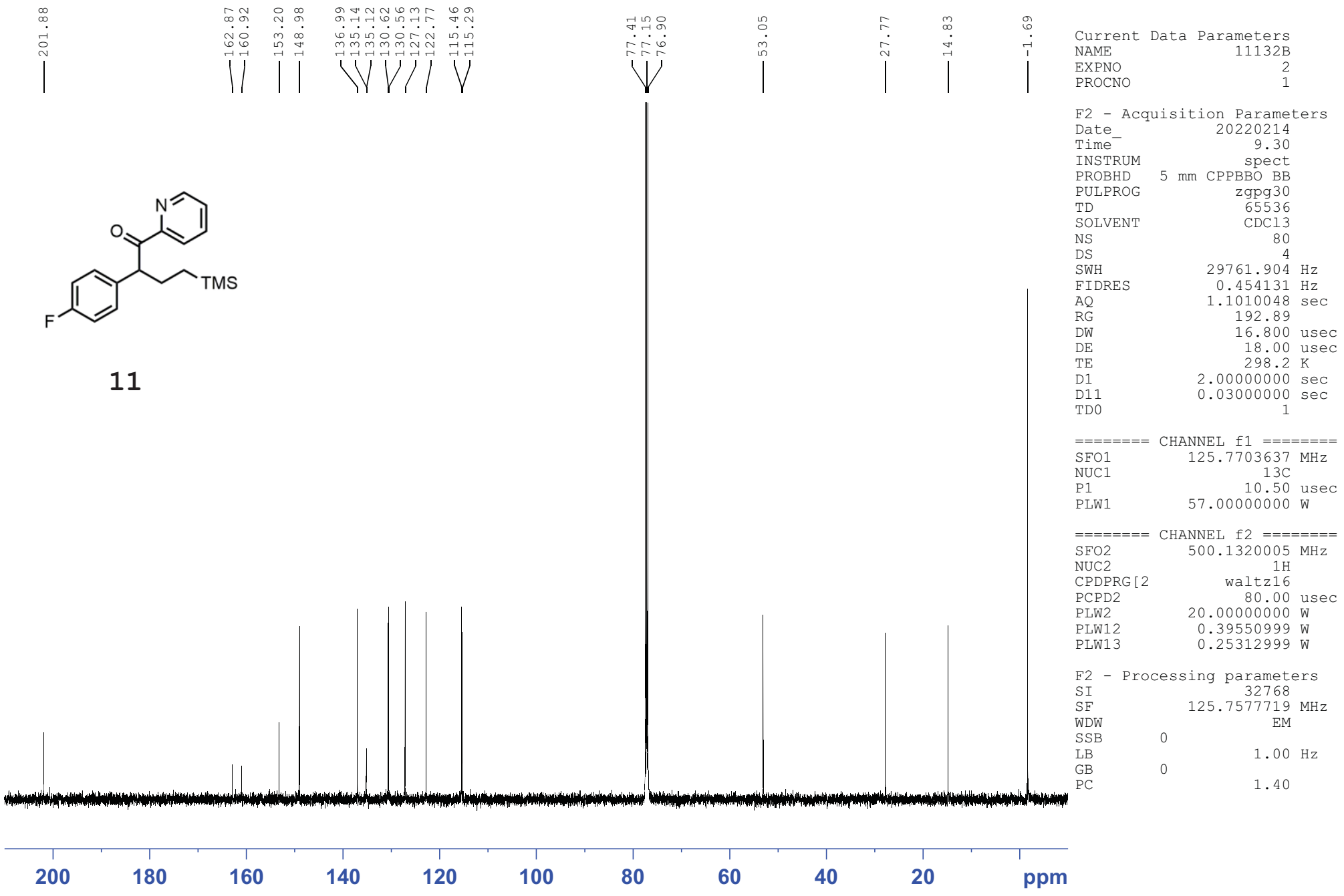

Supplementary Figure 18. ^{13}C-NMR of compound **11**, recorded at 126 MHz and 25 °C in $CDCl_3$.

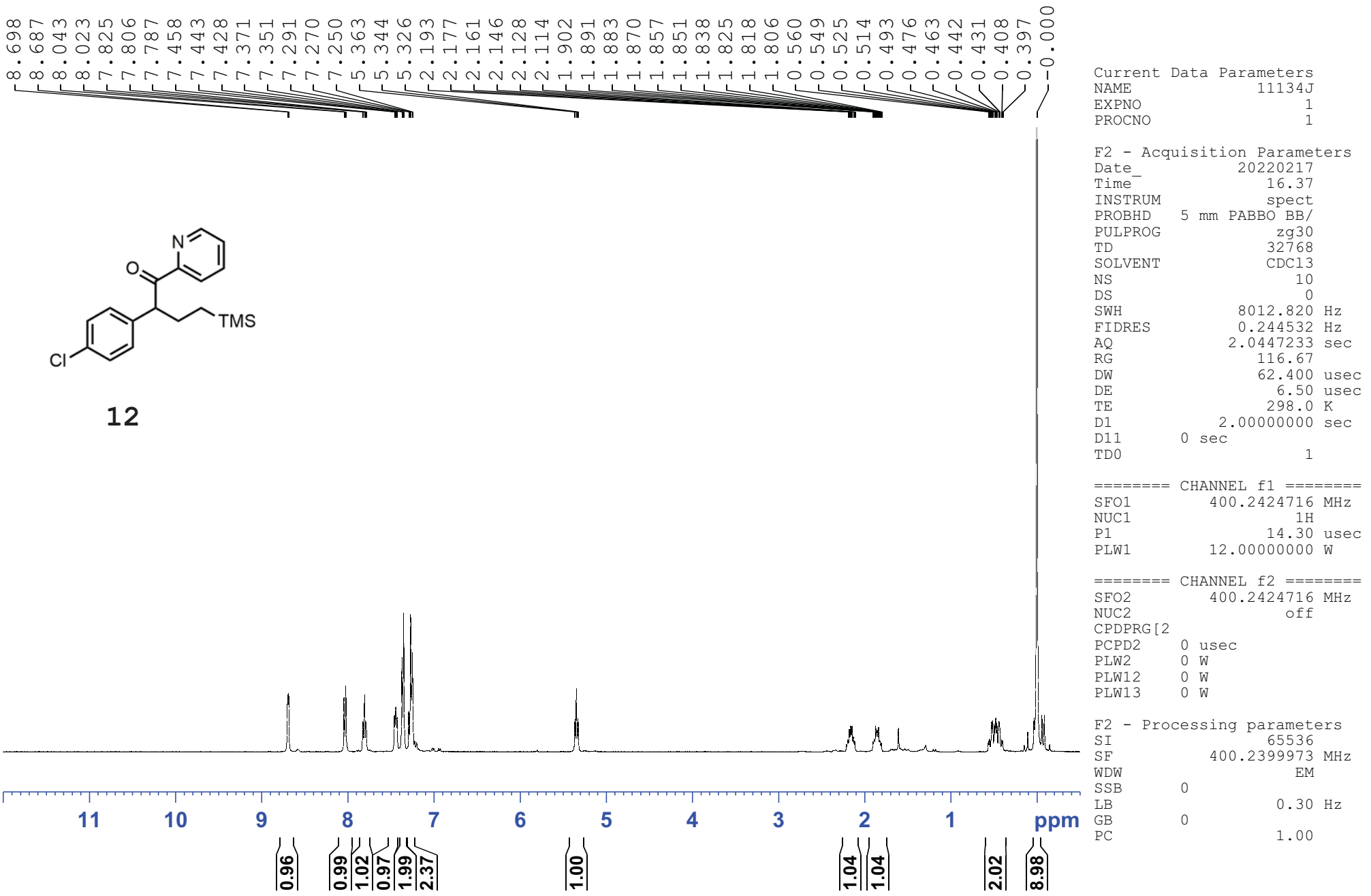


```
Current Data Parameters
NAME                  11134J
EXPNO                      1
PROCNO                     1

F2 - Acquisition Parameters
Date_              20220217
Time                  16.37
INSTRUM               spect
PROBHD    5 mm PABBO BB/
PULPROG                zg30
TD                    32768
SOLVENT               CDCl3
NS                       10
DS                        0
SWH                8012.820 Hz
FIDRES             0.244532 Hz
AQ                2.0447233 sec
RG                   116.67
DW                   62.400 usec
DE                    6.50 usec
TE                   298.0 K
D1              2.00000000 sec
D11       0 sec
TD0                       1

======== CHANNEL f1 ========
SFO1           400.2424716 MHz
NUC1                      1H
P1                    14.30 usec
PLW1           12.00000000 W

======== CHANNEL f2 ========
SFO2           400.2424716 MHz
NUC2                     off
CPDPRG[2
PCPD2      0 usec
PLW2       0 W
PLW12      0 W
PLW13      0 W

F2 - Processing parameters
SI                     65536
SF             400.2399973 MHz
WDW                       EM
SSB        0
LB                    0.30 Hz
GB         0
PC                    1.00
```

Supplementary Figure 19. ^{1}H-NMR of compound **12**, recorded at 400 MHz and 25 °C in $CDCl_3$.

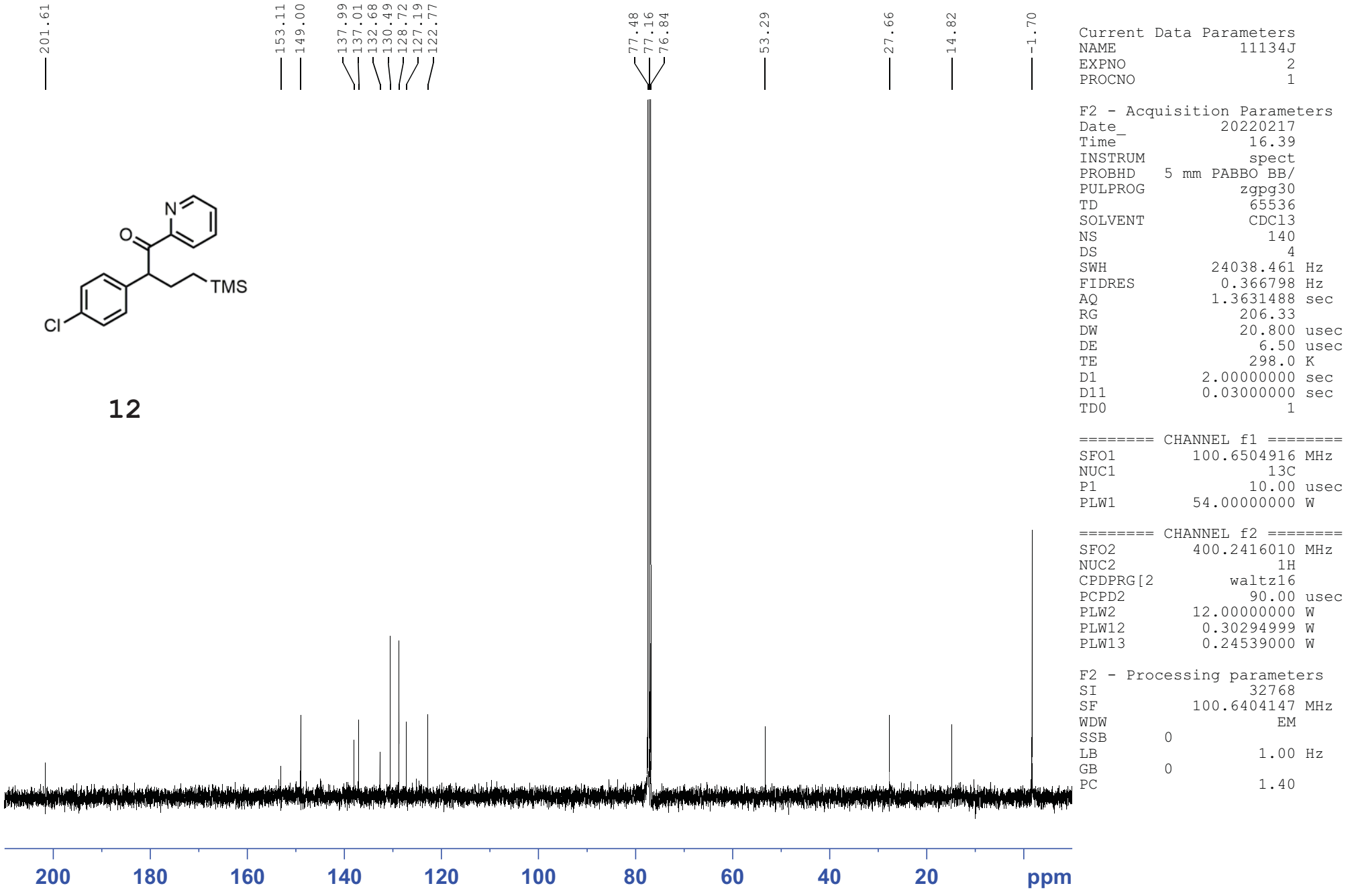

Supplementary Figure 20. ^{13}C-NMR of compound **12**, recorded at 101 MHz and 25 °C in $CDCl_3$.

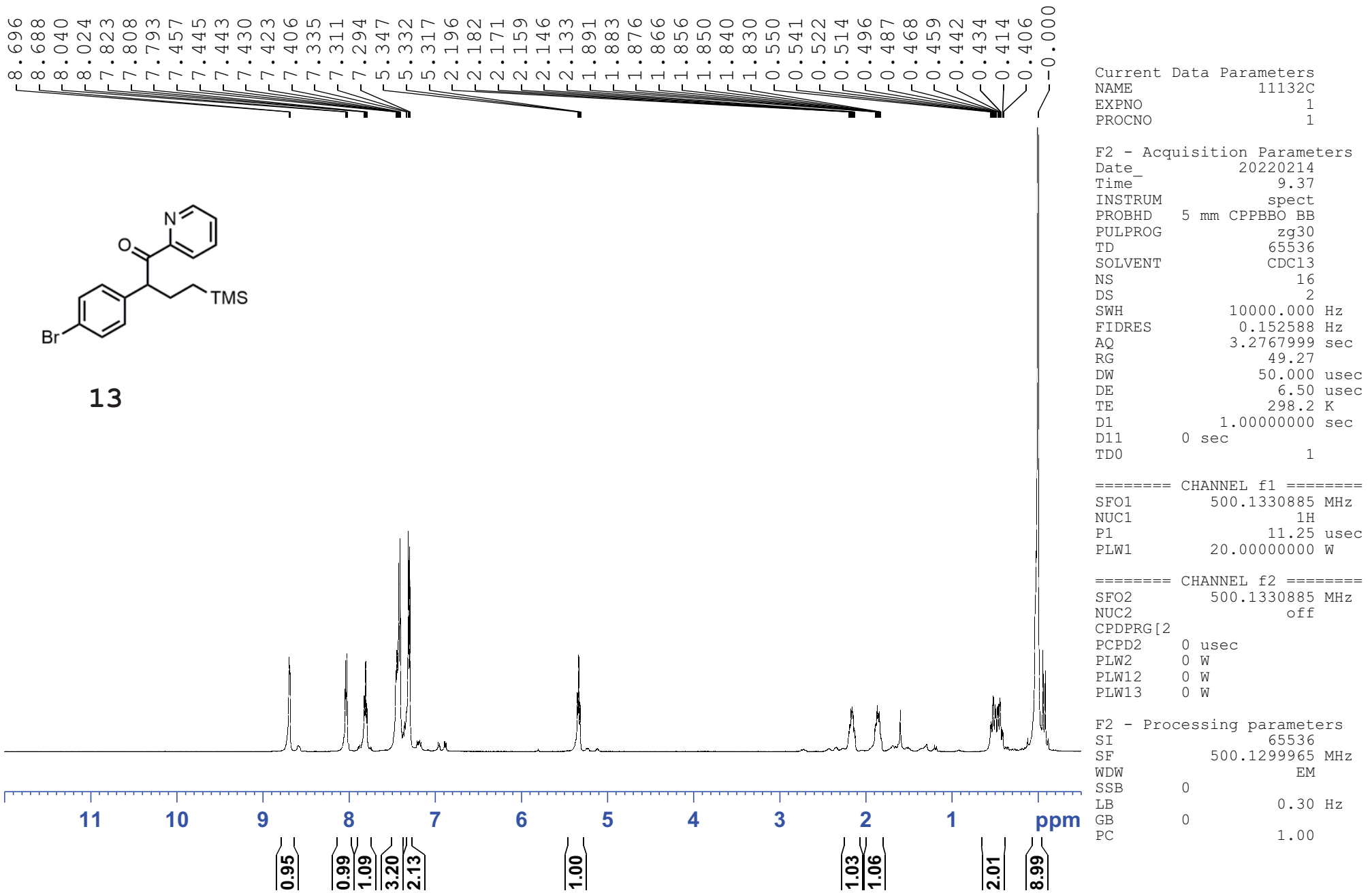


```
Current Data Parameters
NAME                  11132C
EXPNO                      1
PROCNO                     1

F2 - Acquisition Parameters
Date_              20220214
Time                   9.37
INSTRUM               spect
PROBHD    5 mm CPPBBO BB
PULPROG                zg30
TD                    65536
SOLVENT               CDCl3
NS                       16
DS                        2
SWH              10000.000 Hz
FIDRES               0.152588 Hz
AQ               3.2767999 sec
RG                    49.27
DW                   50.000 usec
DE                    6.50 usec
TE                   298.2 K
D1             1.00000000 sec
D11       0 sec
TD0                        1

======== CHANNEL f1 ========
SFO1           500.1330885 MHz
NUC1                      1H
P1                    11.25 usec
PLW1          20.00000000 W

======== CHANNEL f2 ========
SFO2           500.1330885 MHz
NUC2                     off
CPDPRG[2
PCPD2      0 usec
PLW2       0 W
PLW12      0 W
PLW13      0 W

F2 - Processing parameters
SI                        65536
SF             500.1299965 MHz
WDW                        EM
SSB        0
LB                    0.30 Hz
GB         0
PC                    1.00
```

Supplementary Figure 21. ^{1}H-NMR of compound **13**, recorded at 500 MHz and 25 °C in $CDCl_3$.

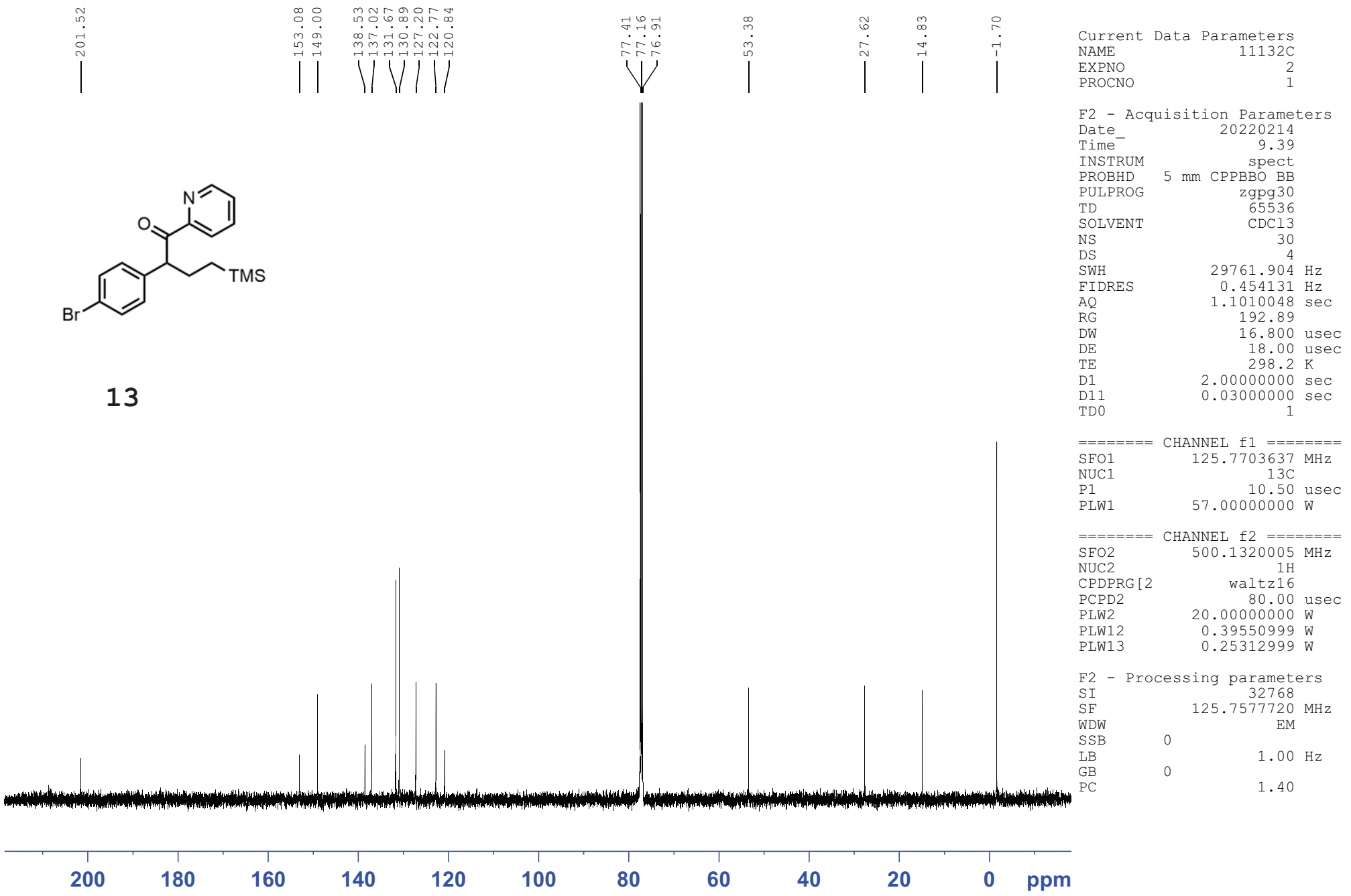


```
Current Data Parameters
NAME                 11132C
EXPNO                     2
PROCNO                    1

F2 - Acquisition Parameters
Date_              20220214
Time                   9.39
INSTRUM               spect
PROBHD   5 mm CPPBBO BB
PULPROG              zgpg30
TD                    65536
SOLVENT               CDCl3
NS                       30
DS                        4
SWH              29761.904 Hz
FIDRES            0.454131 Hz
AQ               1.1010048 sec
RG                   192.89
DW                 16.800 usec
DE                 18.00 usec
TE                  298.2 K
D1              2.00000000 sec
D11             0.03000000 sec
TD0                       1

======== CHANNEL f1 ========
SFO1           125.7703637 MHz
NUC1                    13C
P1                  10.50 usec
PLW1           57.00000000 W

======== CHANNEL f2 ========
SFO2           500.1320005 MHz
NUC2                    1H
CPDPRG[2           waltz16
PCPD2               80.00 usec
PLW2           20.00000000 W
PLW12           0.39550999 W
PLW13           0.25312999 W

F2 - Processing parameters
SI                      32768
SF             125.7577720 MHz
WDW                        EM
SSB       0
LB                   1.00 Hz
GB        0
PC                   1.40
```

Supplementary Figure 22. ^{13}C-NMR of compound **13**, recorded at 126 MHz and 25 °C in $CDCl_3$.

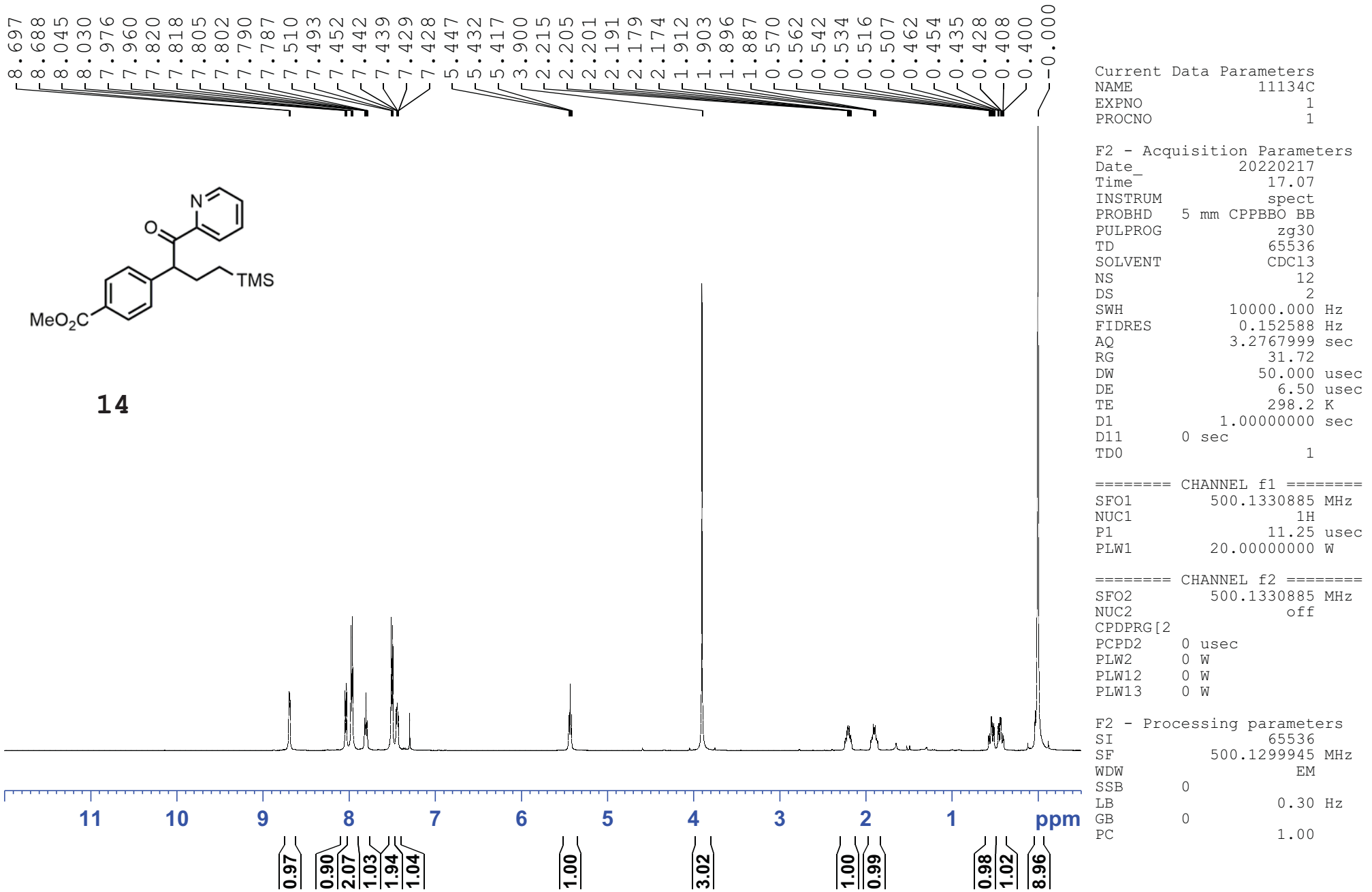


```
Current Data Parameters
NAME                  11134C
EXPNO                      1
PROCNO                     1

F2 - Acquisition Parameters
Date_              20220217
Time                 17.07
INSTRUM              spect
PROBHD    5 mm CPPBBO BB
PULPROG               zg30
TD                   65536
SOLVENT              CDCl3
NS                      12
DS                       2
SWH             10000.000 Hz
FIDRES           0.152588 Hz
AQ               3.2767999 sec
RG                   31.72
DW                  50.000 usec
DE                   6.50 usec
TE                  298.2 K
D1              1.00000000 sec
D11       0 sec
TD0                      1

======== CHANNEL f1 ========
SFO1          500.1330885 MHz
NUC1                    1H
P1                  11.25 usec
PLW1          20.00000000 W

======== CHANNEL f2 ========
SFO2          500.1330885 MHz
NUC2                   off
CPDPRG[2
PCPD2      0 usec
PLW2       0 W
PLW12      0 W
PLW13      0 W

F2 - Processing parameters
SI                   65536
SF            500.1299945 MHz
WDW                     EM
SSB        0
LB                   0.30 Hz
GB         0
PC                   1.00
```

Supplementary Figure 23. ^{1}H-NMR of compound **14**, recorded at 500 MHz and 25 °C in $CDCl_3$.

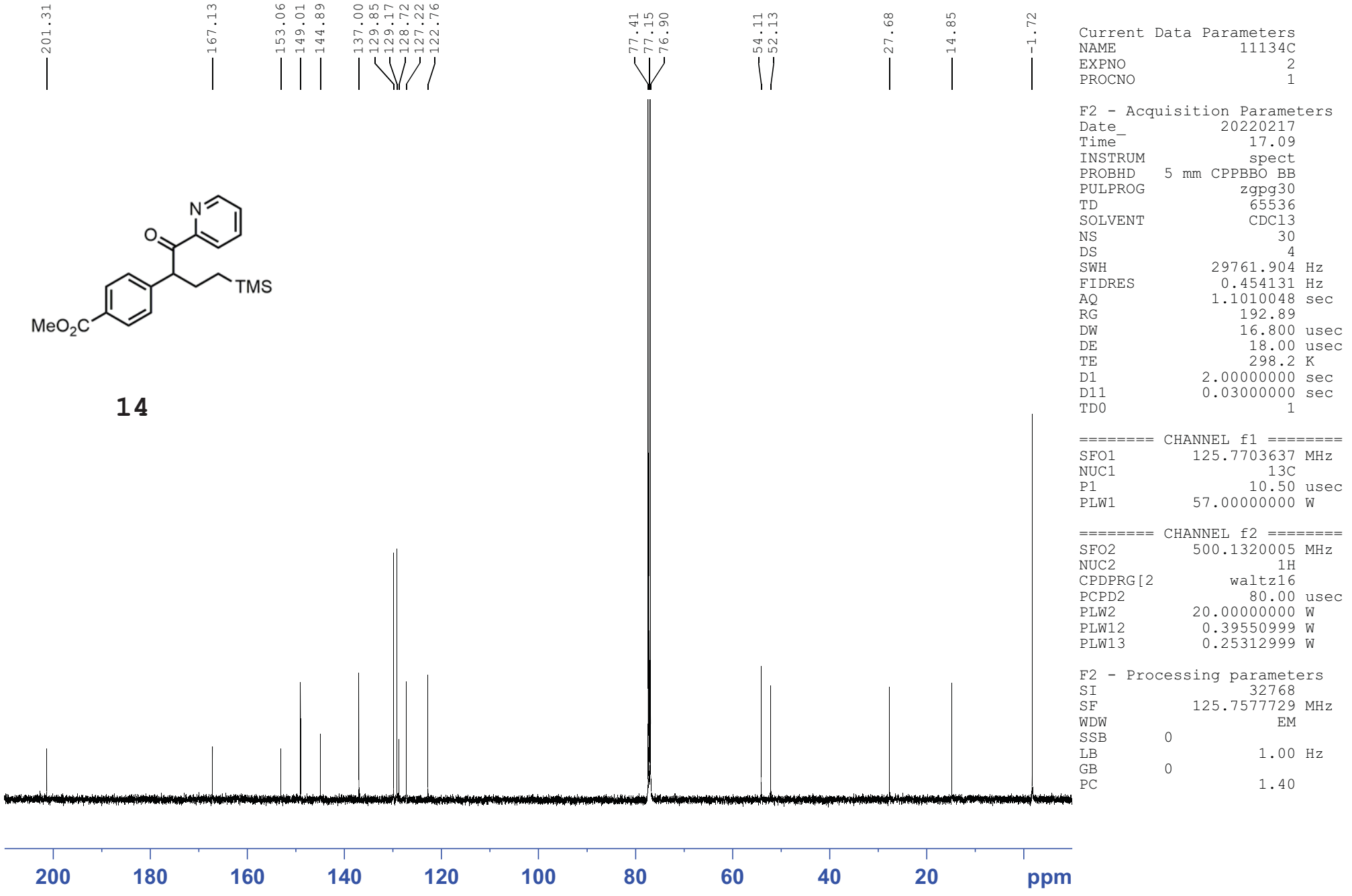


```
Current Data Parameters
NAME                  11134C
EXPNO                      2
PROCNO                     1

F2 - Acquisition Parameters
Date_              20220217
Time                  17.09
INSTRUM               spect
PROBHD   5 mm CPPBBO BB
PULPROG              zgpg30
TD                    65536
SOLVENT               CDCl3
NS                       30
DS                        4
SWH              29761.904 Hz
FIDRES            0.454131 Hz
AQ               1.1010048 sec
RG                   192.89
DW                   16.800 usec
DE                   18.00 usec
TE                   298.2 K
D1              2.00000000 sec
D11             0.03000000 sec
TD0                       1

======== CHANNEL f1 ========
SFO1           125.7703637 MHz
NUC1                    13C
P1                   10.50 usec
PLW1           57.00000000 W

======== CHANNEL f2 ========
SFO2           500.1320005 MHz
NUC2                    1H
CPDPRG[2             waltz16
PCPD2                80.00 usec
PLW2           20.00000000 W
PLW12           0.39550999 W
PLW13           0.25312999 W

F2 - Processing parameters
SI                      32768
SF             125.7577729 MHz
WDW                        EM
SSB          0
LB                    1.00 Hz
GB           0
PC                    1.40
```

Supplementary Figure 24. ^{13}C-NMR of compound **14**, recorded at 126 MHz and 25 °C in $CDCl_3$.

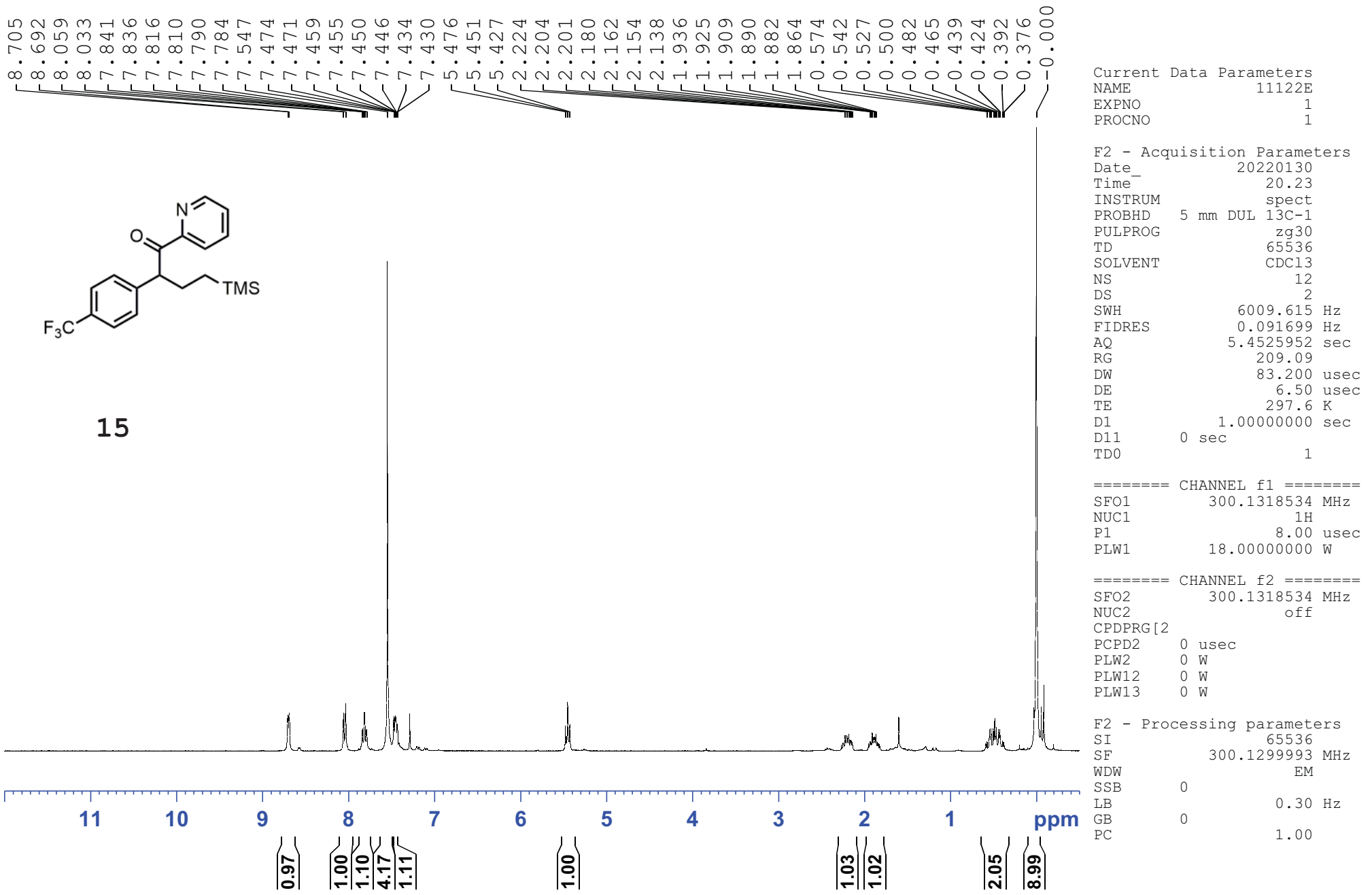


```
Current Data Parameters
NAME                  11122E
EXPNO                      1
PROCNO                     1

F2 - Acquisition Parameters
Date_              20220130
Time                  20.23
INSTRUM               spect
PROBHD    5 mm DUL 13C-1
PULPROG               zg30
TD                   65536
SOLVENT              CDCl3
NS                      12
DS                       2
SWH              6009.615 Hz
FIDRES           0.091699 Hz
AQ               5.4525952 sec
RG                  209.09
DW                  83.200 usec
DE                    6.50 usec
TE                   297.6 K
D1             1.00000000 sec
D11       0 sec
TD0                      1

======== CHANNEL f1 ========
SFO1          300.1318534 MHz
NUC1                    1H
P1                   8.00 usec
PLW1          18.00000000 W

======== CHANNEL f2 ========
SFO2          300.1318534 MHz
NUC2                   off
CPDPRG[2
PCPD2      0 usec
PLW2       0 W
PLW12      0 W
PLW13      0 W

F2 - Processing parameters
SI                   65536
SF            300.1299993 MHz
WDW                     EM
SSB        0
LB                   0.30 Hz
GB         0
PC                   1.00
```

Supplementary Figure 25. ^{1}H-NMR of compound **15**, recorded at 500 MHz and 25 °C in $CDCl_3$.

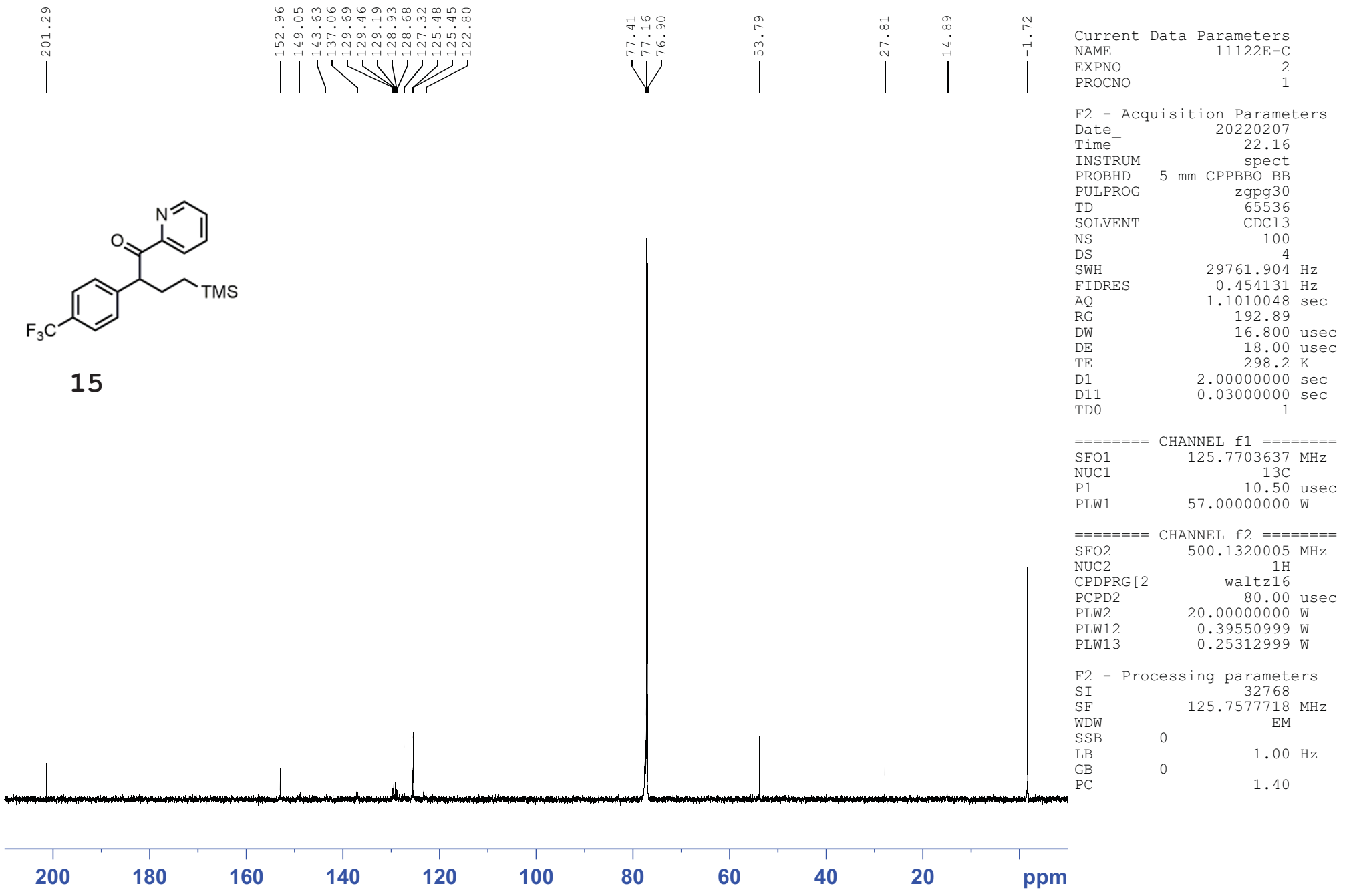


```
Current Data Parameters
NAME            11122E-C
EXPNO                  2
PROCNO                 1

F2 - Acquisition Parameters
Date_           20220207
Time              22.16
INSTRUM           spect
PROBHD   5 mm CPPBBO BB
PULPROG          zgpg30
TD                65536
SOLVENT           CDCl3
NS                  100
DS                    4
SWH           29761.904 Hz
FIDRES         0.454131 Hz
AQ             1.1010048 sec
RG                192.89
DW                16.800 usec
DE                18.00 usec
TE                298.2 K
D1           2.00000000 sec
D11          0.03000000 sec
TD0                   1

======== CHANNEL f1 ========
SFO1         125.7703637 MHz
NUC1                13C
P1                10.50 usec
PLW1         57.00000000 W

======== CHANNEL f2 ========
SFO2         500.1320005 MHz
NUC2                1H
CPDPRG[2         waltz16
PCPD2             80.00 usec
PLW2         20.00000000 W
PLW12         0.39550999 W
PLW13         0.25312999 W

F2 - Processing parameters
SI                 32768
SF           125.7577718 MHz
WDW                  EM
SSB       0
LB                1.00 Hz
GB        0
PC                1.40
```

Supplementary Figure 26. ^{13}C-NMR of compound **15**, recorded at 126 MHz and 25 °C in $CDCl_3$.

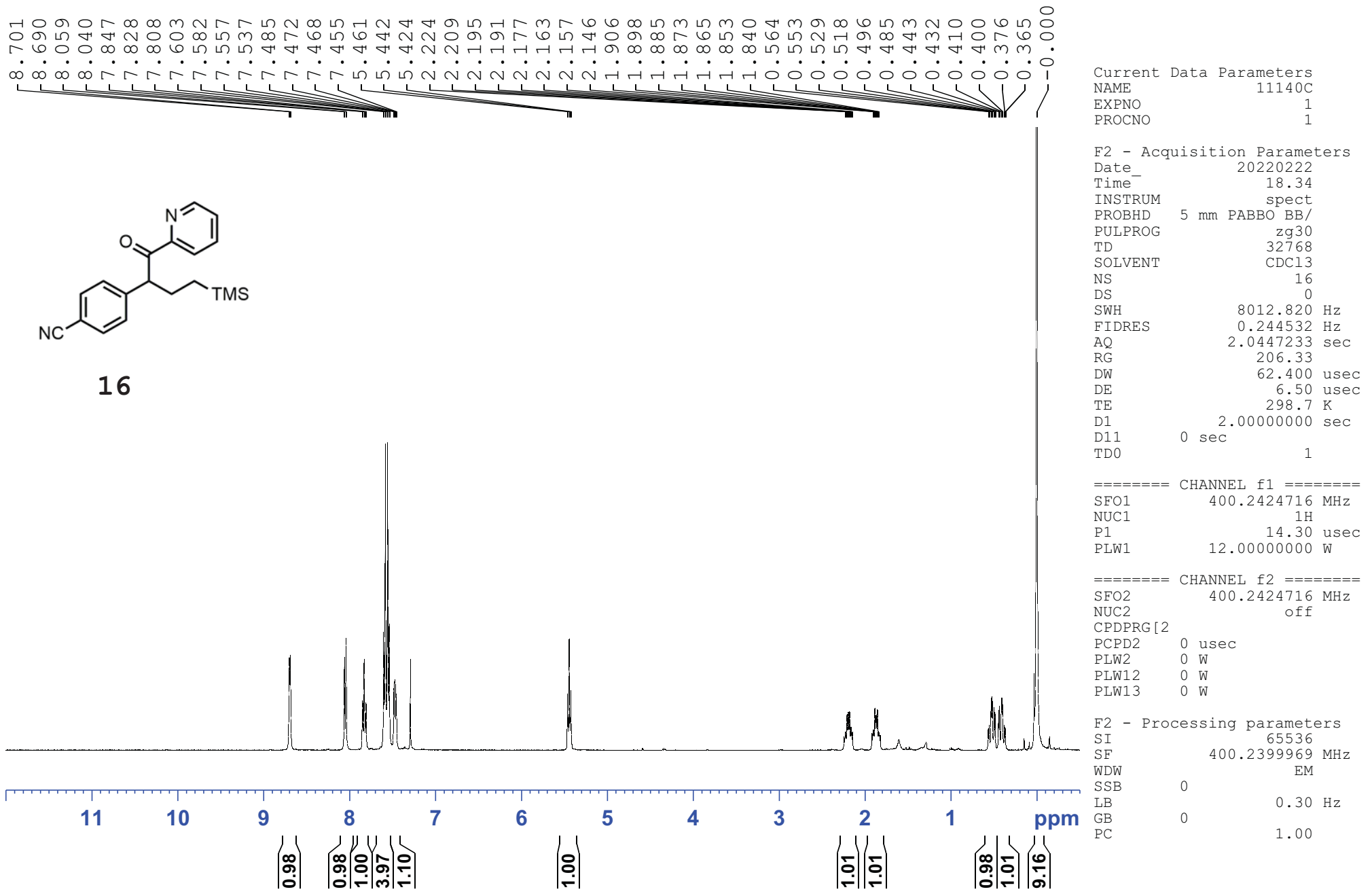

Supplementary Figure 27. ^{1}H-NMR of compound **16**, recorded at 500 MHz and 25 °C in $CDCl_3$.

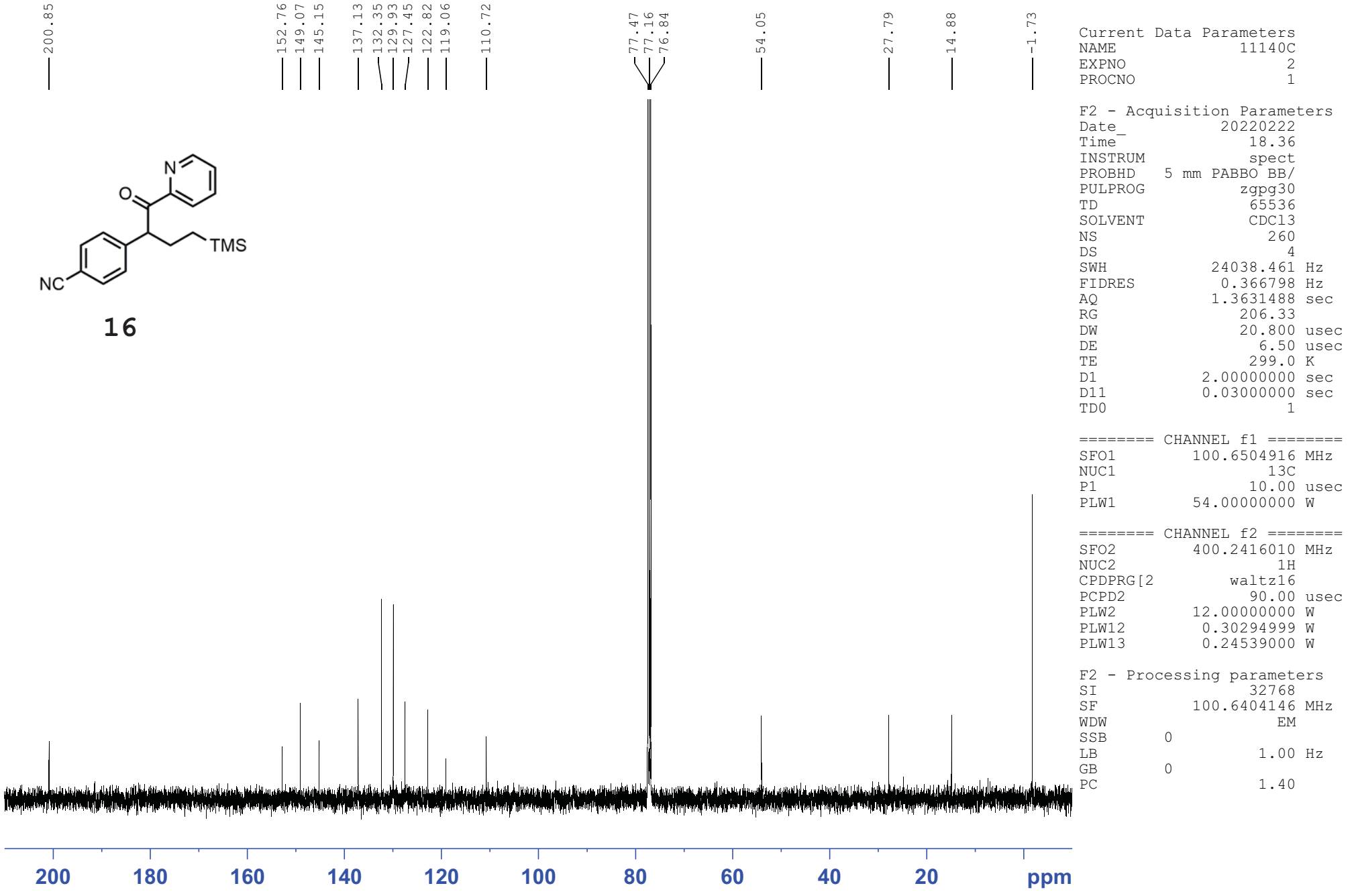


```
Current Data Parameters
NAME                 11140C
EXPNO                     2
PROCNO                    1

F2 - Acquisition Parameters
Date_              20220222
Time                  18.36
INSTRUM               spect
PROBHD   5 mm PABBO BB/
PULPROG              zgpg30
TD                    65536
SOLVENT               CDCl3
NS                      260
DS                        4
SWH              24038.461 Hz
FIDRES            0.366798 Hz
AQ                1.3631488 sec
RG                   206.33
DW                   20.800 usec
DE                    6.50 usec
TE                   299.0 K
D1             2.00000000 sec
D11            0.03000000 sec
TD0                       1

======== CHANNEL f1 ========
SFO1           100.6504916 MHz
NUC1                    13C
P1                   10.00 usec
PLW1           54.00000000 W

======== CHANNEL f2 ========
SFO2           400.2416010 MHz
NUC2                    1H
CPDPRG[2            waltz16
PCPD2                90.00 usec
PLW2           12.00000000 W
PLW12           0.30294999 W
PLW13           0.24539000 W

F2 - Processing parameters
SI                    32768
SF             100.6404146 MHz
WDW                      EM
SSB          0
LB                    1.00 Hz
GB           0
PC                    1.40
```

Supplementary Figure 28. ^{13}C-NMR of compound **16**, recorded at 101 MHz and 25 °C in $CDCl_3$.

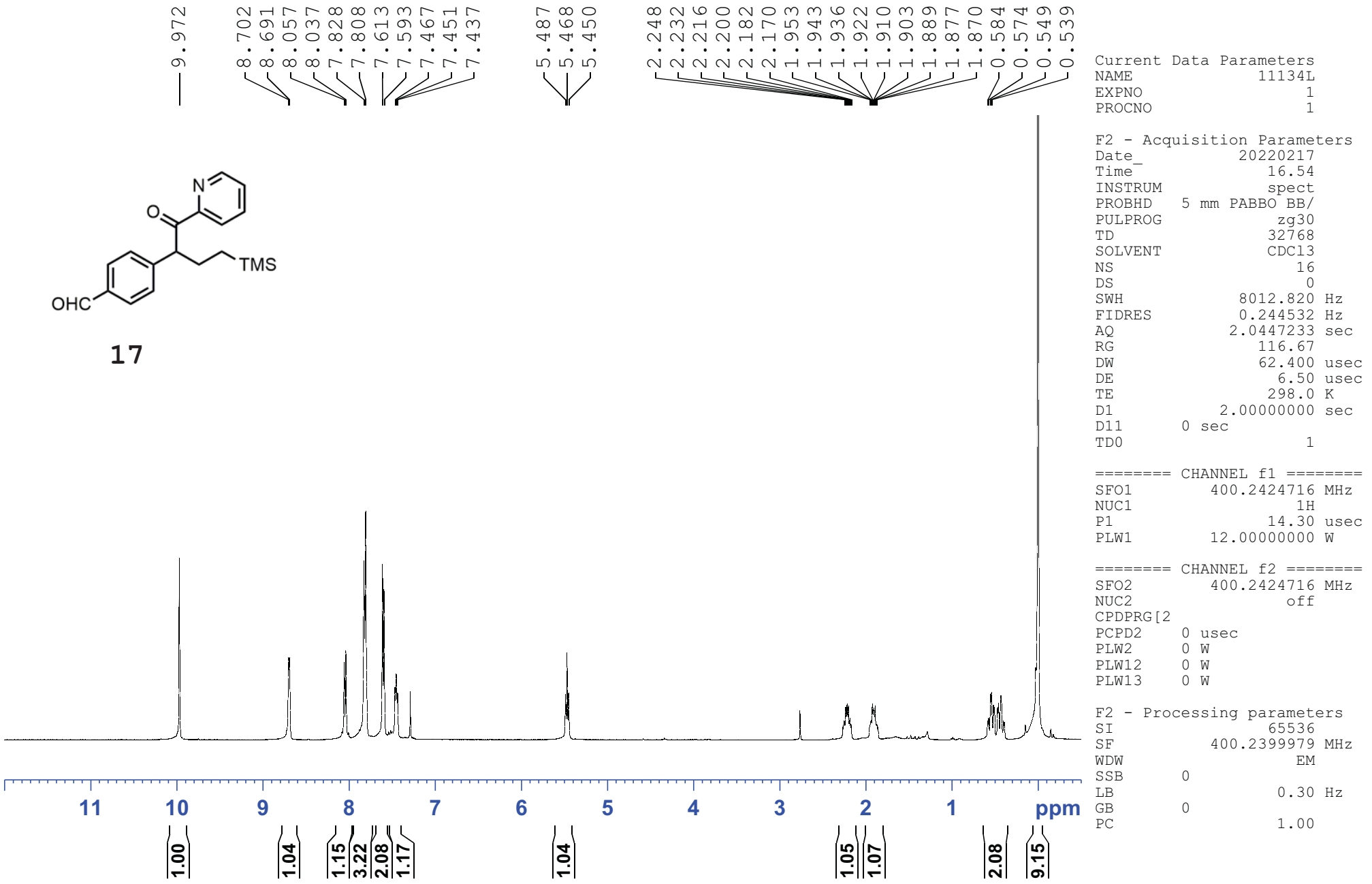


```
Current Data Parameters
NAME                  11134L
EXPNO                      1
PROCNO                     1

F2 - Acquisition Parameters
Date_              20220217
Time                 16.54
INSTRUM              spect
PROBHD    5 mm PABBO BB/
PULPROG               zg30
TD                   32768
SOLVENT              CDCl3
NS                      16
DS                       0
SWH               8012.820 Hz
FIDRES            0.244532 Hz
AQ               2.0447233 sec
RG                  116.67
DW                  62.400 usec
DE                    6.50 usec
TE                  298.0 K
D1             2.00000000 sec
D11       0 sec
TD0                      1

======== CHANNEL f1 ========
SFO1           400.2424716 MHz
NUC1                    1H
P1                   14.30 usec
PLW1           12.00000000 W

======== CHANNEL f2 ========
SFO2           400.2424716 MHz
NUC2                   off
CPDPRG[2
PCPD2      0 usec
PLW2       0 W
PLW12      0 W
PLW13      0 W

F2 - Processing parameters
SI                    65536
SF           400.2399979 MHz
WDW                     EM
SSB        0
LB                  0.30 Hz
GB         0
PC                  1.00
```

Supplementary Figure 29. ^{1}H-NMR of compound **17**, recorded at 400 MHz and 25 °C in $CDCl_3$.

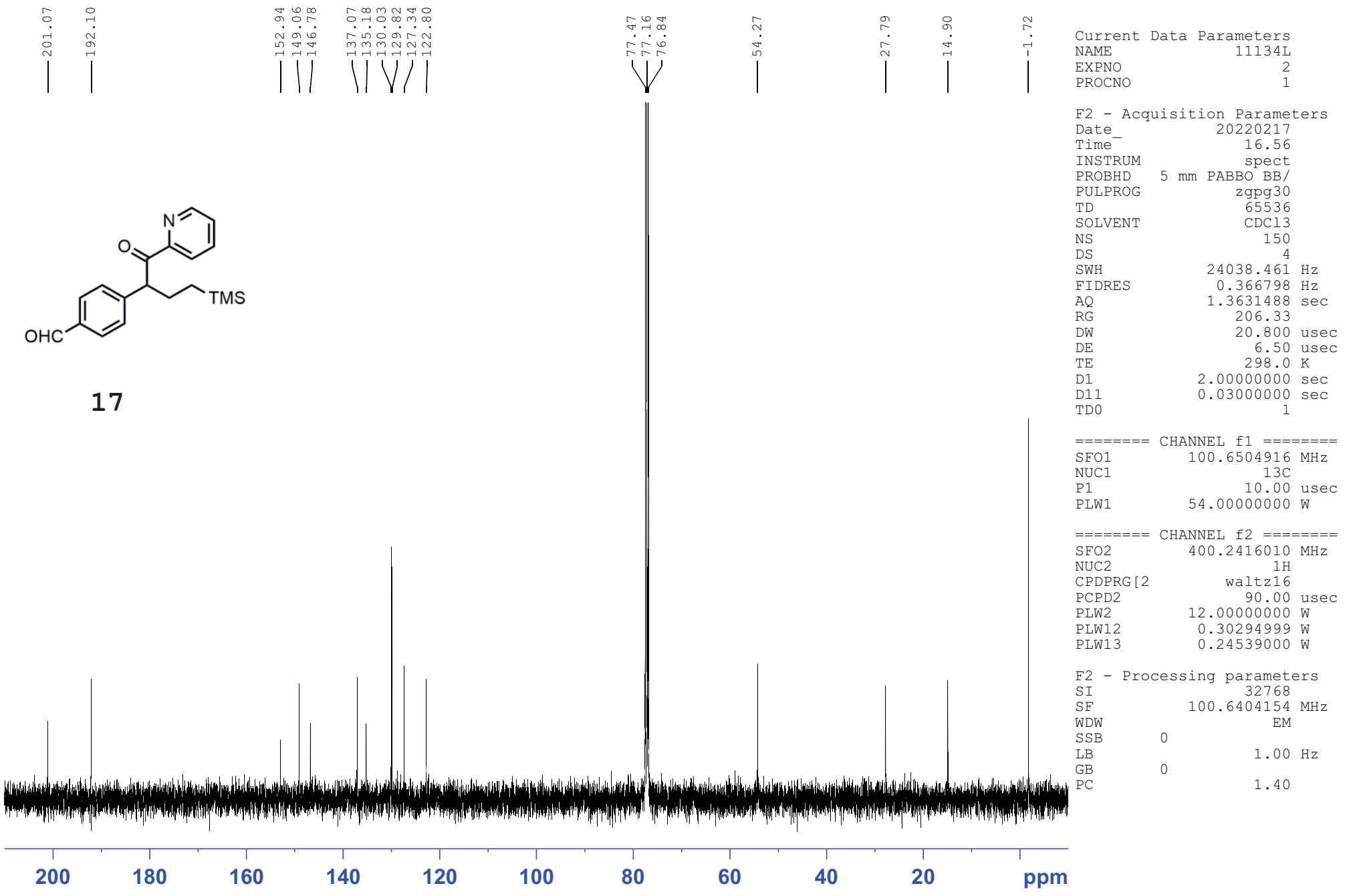


```
Current Data Parameters
NAME                 11134L
EXPNO                     2
PROCNO                    1

F2 - Acquisition Parameters
Date_              20220217
Time                 16.56
INSTRUM              spect
PROBHD   5 mm PABBO BB/
PULPROG             zgpg30
TD                   65536
SOLVENT              CDCl3
NS                     150
DS                       4
SWH             24038.461 Hz
FIDRES           0.366798 Hz
AQ               1.3631488 sec
RG                  206.33
DW                  20.800 usec
DE                   6.50 usec
TE                  298.0 K
D1             2.00000000 sec
D11            0.03000000 sec
TD0                      1

======== CHANNEL f1 ========
SFO1          100.6504916 MHz
NUC1                   13C
P1                  10.00 usec
PLW1          54.00000000 W

======== CHANNEL f2 ========
SFO2          400.2416010 MHz
NUC2                   1H
CPDPRG[2           waltz16
PCPD2               90.00 usec
PLW2          12.00000000 W
PLW12          0.30294999 W
PLW13          0.24539000 W

F2 - Processing parameters
SI                   32768
SF            100.6404154 MHz
WDW                    EM
SSB       0
LB                  1.00 Hz
GB        0
PC                  1.40
```

Supplementary Figure 30. ^{13}C-NMR of compound **17**, recorded at 101 MHz and 25 °C in $CDCl_3$.

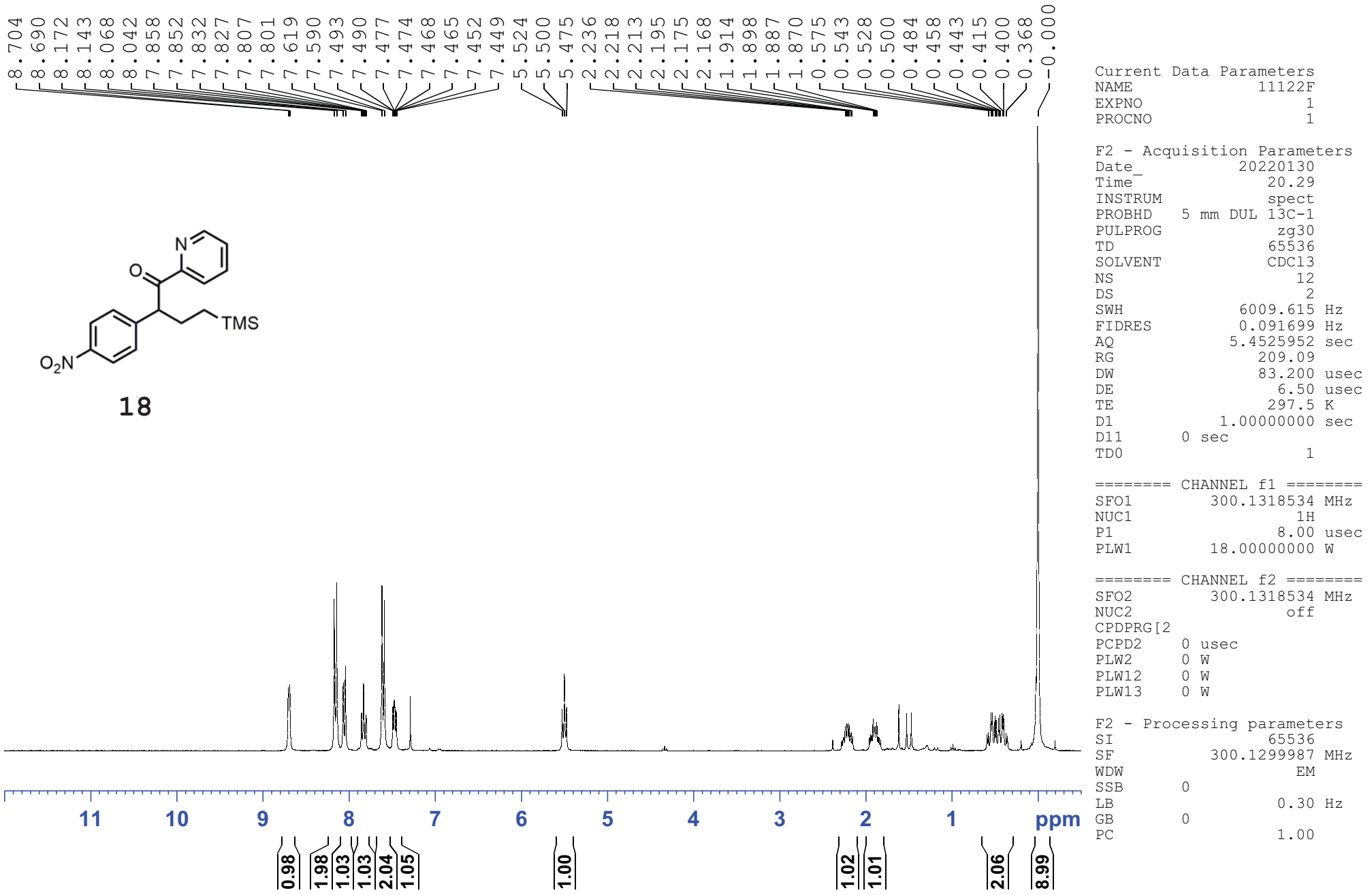


```
Current Data Parameters
NAME                  11122F
EXPNO                      1
PROCNO                     1

F2 - Acquisition Parameters
Date_              20220130
Time                  20.29
INSTRUM               spect
PROBHD    5 mm DUL 13C-1
PULPROG               zg30
TD                   65536
SOLVENT              CDCl3
NS                      12
DS                       2
SWH              6009.615 Hz
FIDRES           0.091699 Hz
AQ               5.4525952 sec
RG                  209.09
DW                  83.200 usec
DE                    6.50 usec
TE                   297.5 K
D1             1.00000000 sec
D11       0 sec
TD0                      1

======== CHANNEL f1 ========
SFO1          300.1318534 MHz
NUC1                    1H
P1                   8.00 usec
PLW1          18.00000000 W

======== CHANNEL f2 ========
SFO2          300.1318534 MHz
NUC2                   off
CPDPRG[2
PCPD2      0 usec
PLW2       0 W
PLW12      0 W
PLW13      0 W

F2 - Processing parameters
SI                   65536
SF          300.1299987 MHz
WDW                     EM
SSB        0
LB                   0.30 Hz
GB         0
PC                   1.00
```

Supplementary Figure 31. ^{1}H-NMR of compound **18**, recorded at 300 MHz and 25 °C in $CDCl_3$.

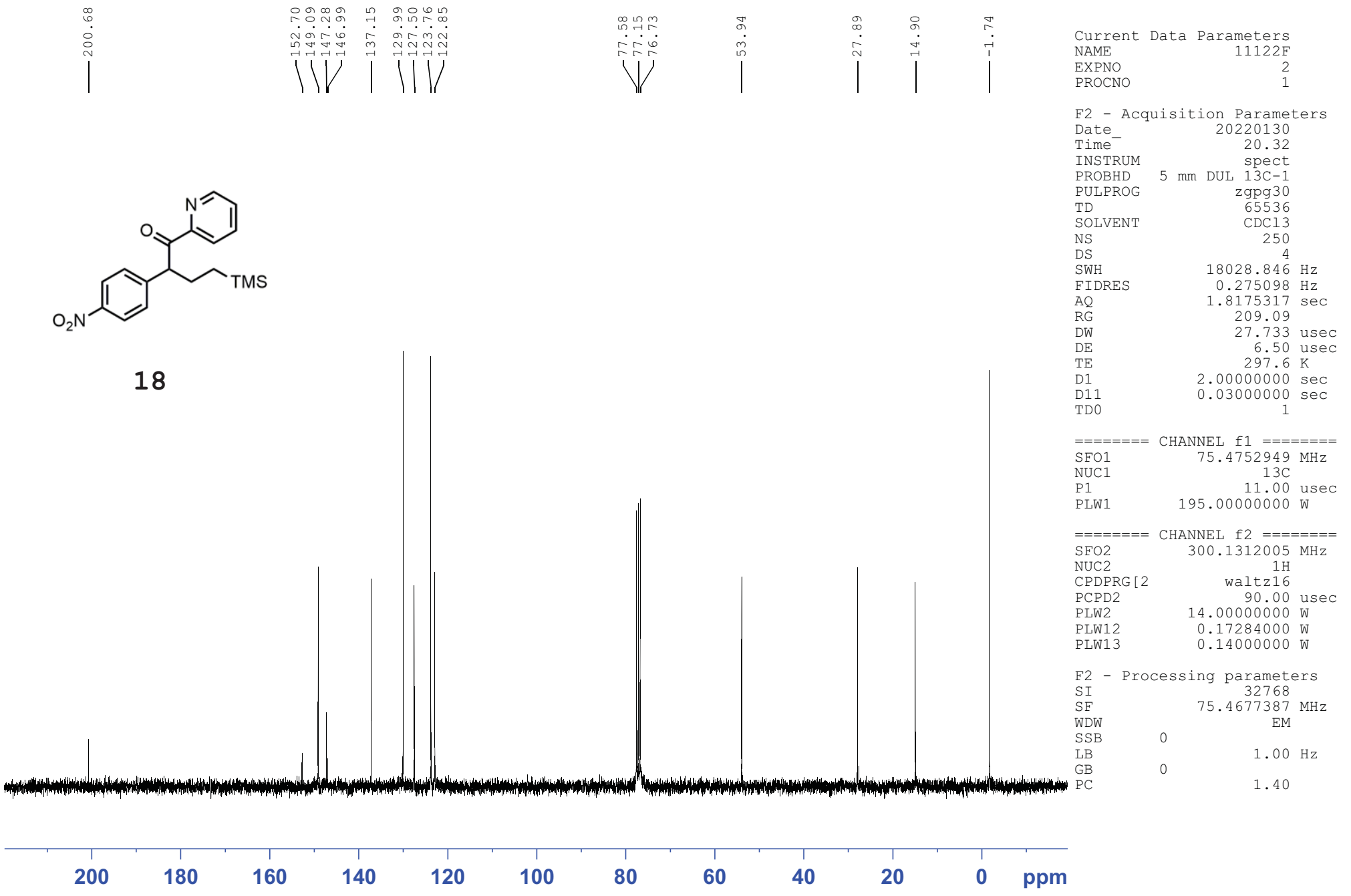


```
Current Data Parameters
NAME                 11122F
EXPNO                     2
PROCNO                    1

F2 - Acquisition Parameters
Date_              20220130
Time                 20.32
INSTRUM              spect
PROBHD   5 mm DUL 13C-1
PULPROG             zgpg30
TD                   65536
SOLVENT              CDCl3
NS                     250
DS                       4
SWH              18028.846 Hz
FIDRES           0.275098 Hz
AQ               1.8175317 sec
RG                  209.09
DW                  27.733 usec
DE                    6.50 usec
TE                   297.6 K
D1             2.00000000 sec
D11            0.03000000 sec
TD0                      1

======== CHANNEL f1 ========
SFO1           75.4752949 MHz
NUC1                   13C
P1                  11.00 usec
PLW1         195.00000000 W

======== CHANNEL f2 ========
SFO2          300.1312005 MHz
NUC2                   1H
CPDPRG[2           waltz16
PCPD2                90.00 usec
PLW2          14.00000000 W
PLW12          0.17284000 W
PLW13          0.14000000 W

F2 - Processing parameters
SI                   32768
SF             75.4677387 MHz
WDW                     EM
SSB       0
LB                   1.00 Hz
GB        0
PC                   1.40
```

Supplementary Figure 32. ^{13}C-NMR of compound **18**, recorded at 75 MHz and 25 °C in $CDCl_3$.

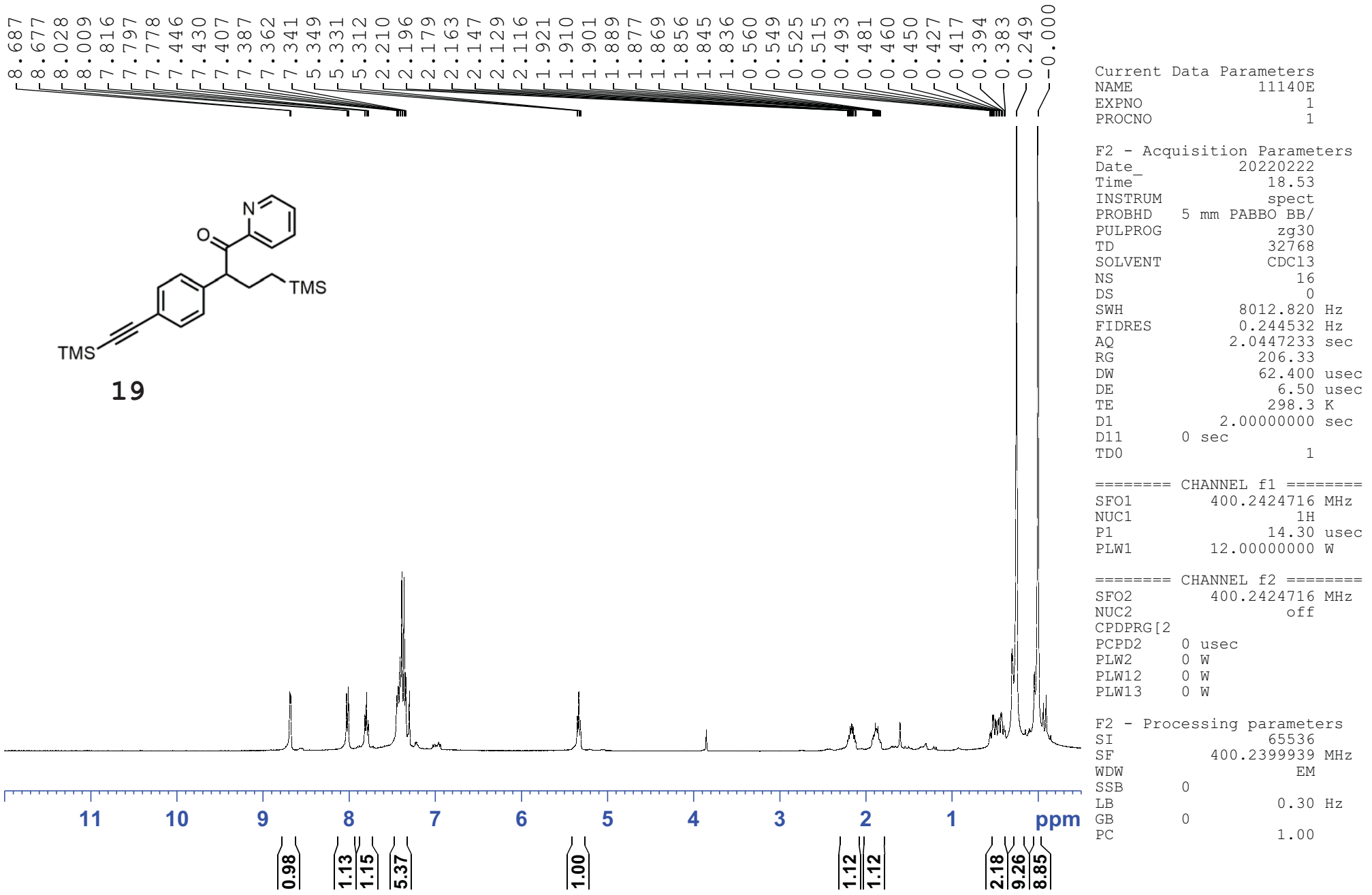


```
Current Data Parameters
NAME                  11140E
EXPNO                      1
PROCNO                     1

F2 - Acquisition Parameters
Date_              20220222
Time                  18.53
INSTRUM               spect
PROBHD    5 mm PABBO BB/
PULPROG                zg30
TD                    32768
SOLVENT               CDCl3
NS                       16
DS                        0
SWH                8012.820 Hz
FIDRES             0.244532 Hz
AQ                2.0447233 sec
RG                   206.33
DW                   62.400 usec
DE                    6.50 usec
TE                   298.3 K
D1              2.00000000 sec
D11       0 sec
TD0                       1

======== CHANNEL f1 ========
SFO1           400.2424716 MHz
NUC1                      1H
P1                    14.30 usec
PLW1           12.00000000 W

======== CHANNEL f2 ========
SFO2           400.2424716 MHz
NUC2                    off
CPDPRG[2
PCPD2      0 usec
PLW2       0 W
PLW12      0 W
PLW13      0 W

F2 - Processing parameters
SI                     65536
SF             400.2399939 MHz
WDW                       EM
SSB        0
LB                    0.30 Hz
GB         0
PC                    1.00
```

Supplementary Figure 33. ^{1}H-NMR of compound **19**, recorded at 400 MHz and 25 °C in $CDCl_3$.

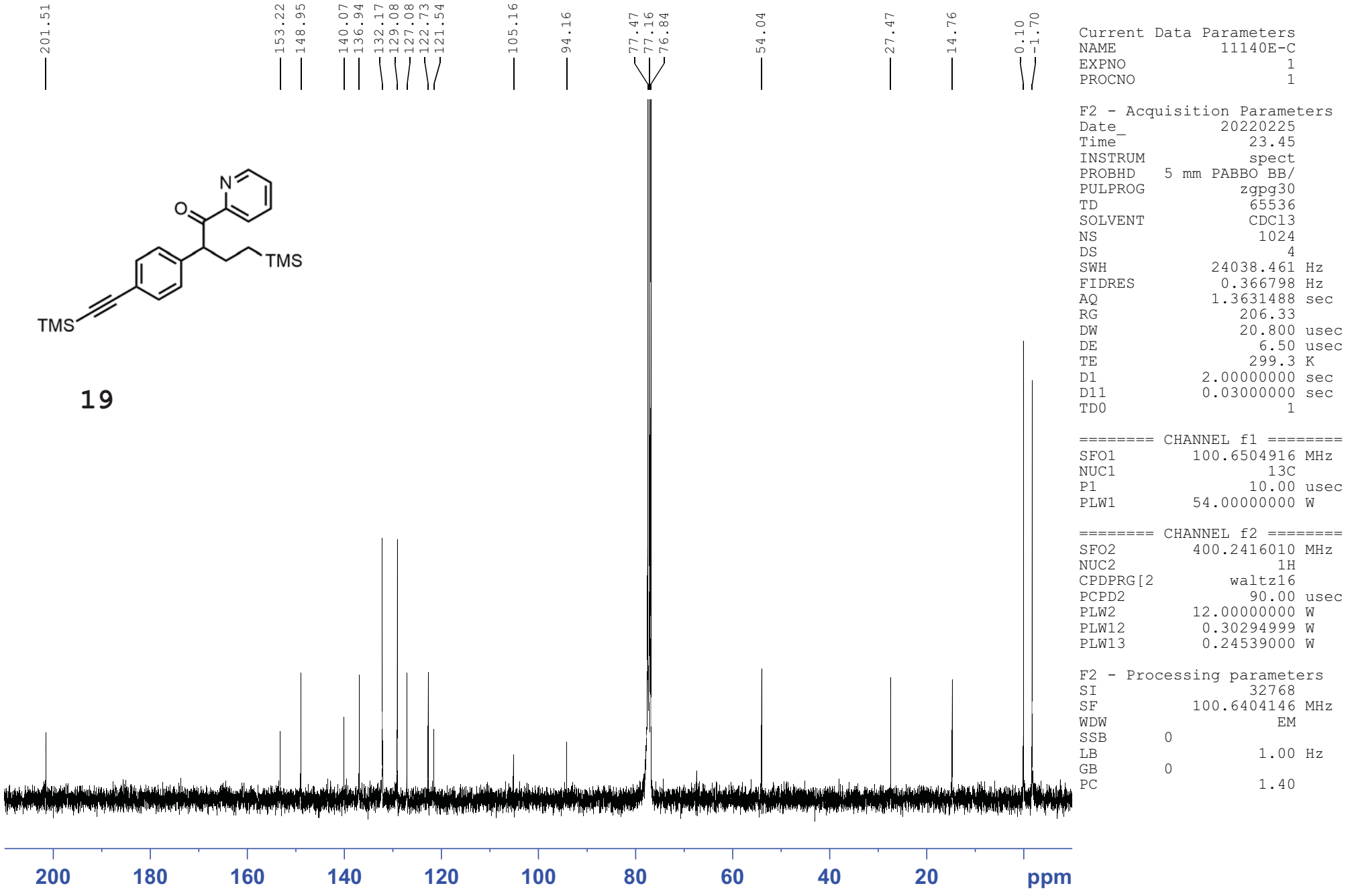

Supplementary Figure 34. ^{13}C-NMR of compound **19**, recorded at 101 MHz and 25 °C in $CDCl_3$.

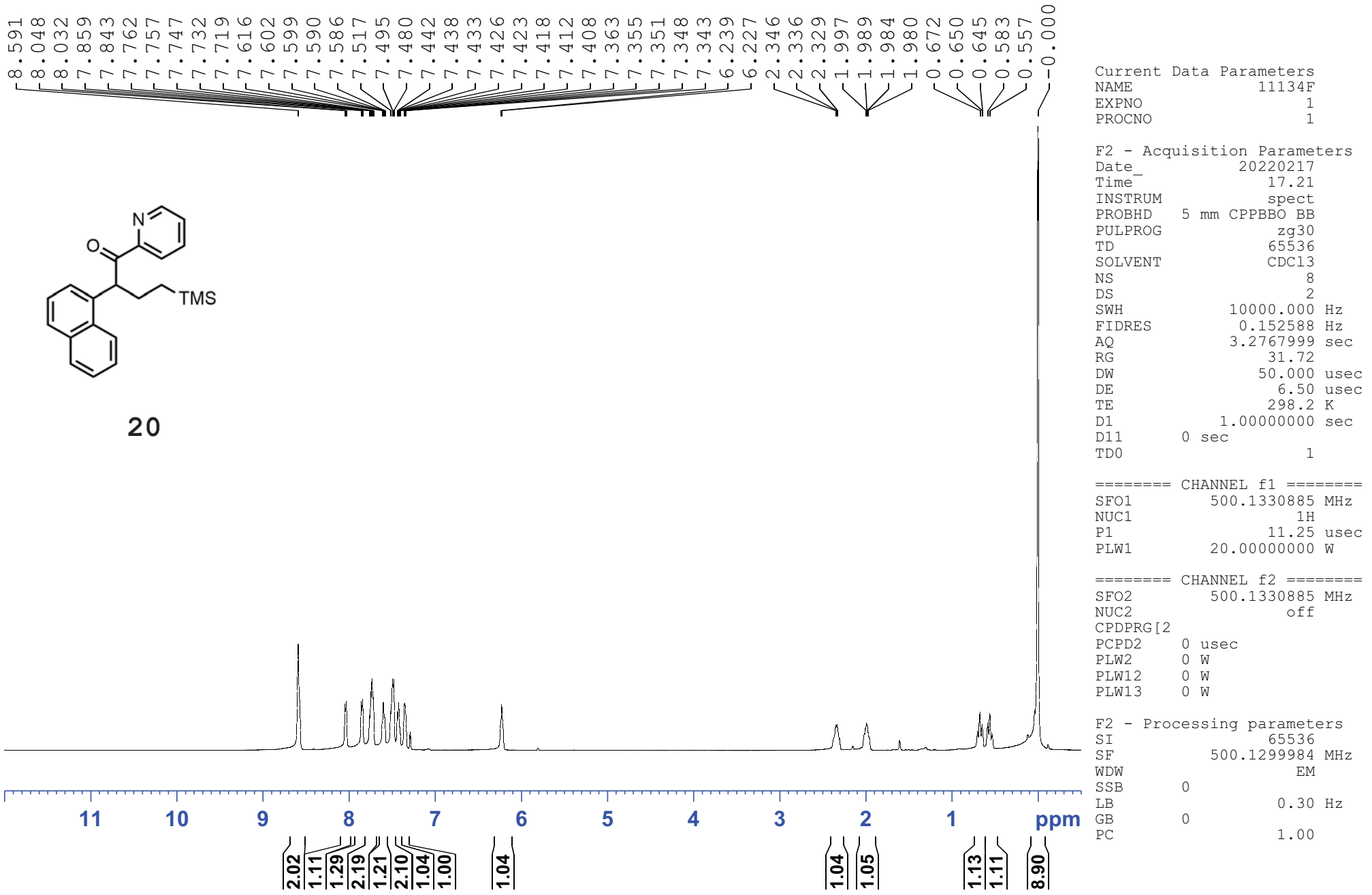


```
Current Data Parameters
NAME                  11134F
EXPNO                      1
PROCNO                     1

F2 - Acquisition Parameters
Date_              20220217
Time                  17.21
INSTRUM               spect
PROBHD    5 mm CPPBBO BB
PULPROG                zg30
TD                    65536
SOLVENT               CDCl3
NS                        8
DS                        2
SWH              10000.000 Hz
FIDRES            0.152588 Hz
AQ               3.2767999 sec
RG                    31.72
DW                  50.000 usec
DE                   6.50 usec
TE                   298.2 K
D1             1.00000000 sec
D11       0 sec
TD0                       1

======== CHANNEL f1 ========
SFO1           500.1330885 MHz
NUC1                     1H
P1                   11.25 usec
PLW1          20.00000000 W

======== CHANNEL f2 ========
SFO2           500.1330885 MHz
NUC2                   off
CPDPRG[2
PCPD2      0 usec
PLW2       0 W
PLW12      0 W
PLW13      0 W

F2 - Processing parameters
SI                    65536
SF           500.1299984 MHz
WDW                      EM
SSB        0
LB                   0.30 Hz
GB         0
PC                    1.00
```

Supplementary Figure 35. ^{1}H-NMR of compound **20**, recorded at 500 MHz and 25 °C in $CDCl_3$.

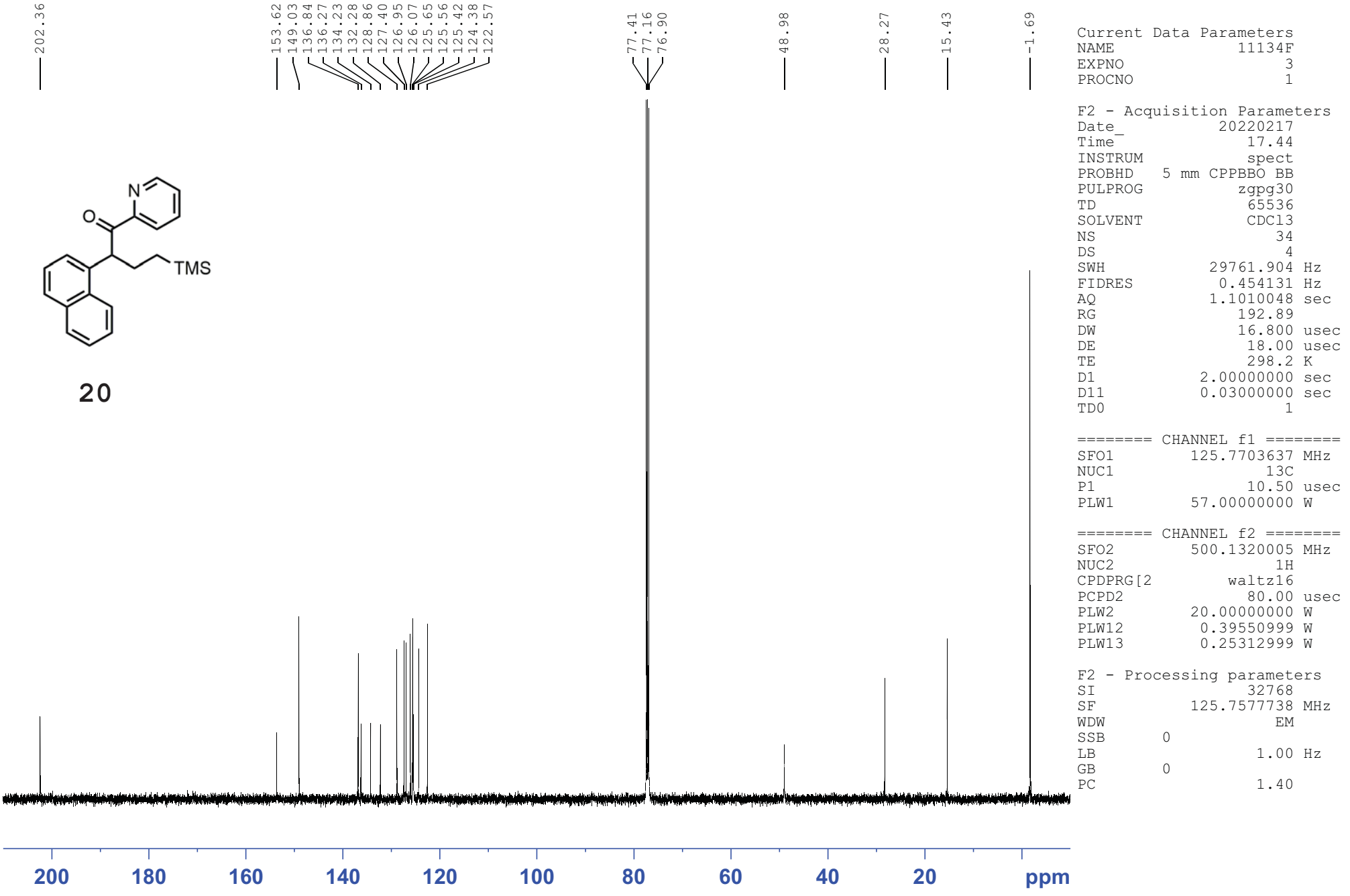

Supplementary Figure 36. ^{13}C-NMR of compound **20**, recorded at 126 MHz and 25 °C in $CDCl_3$.

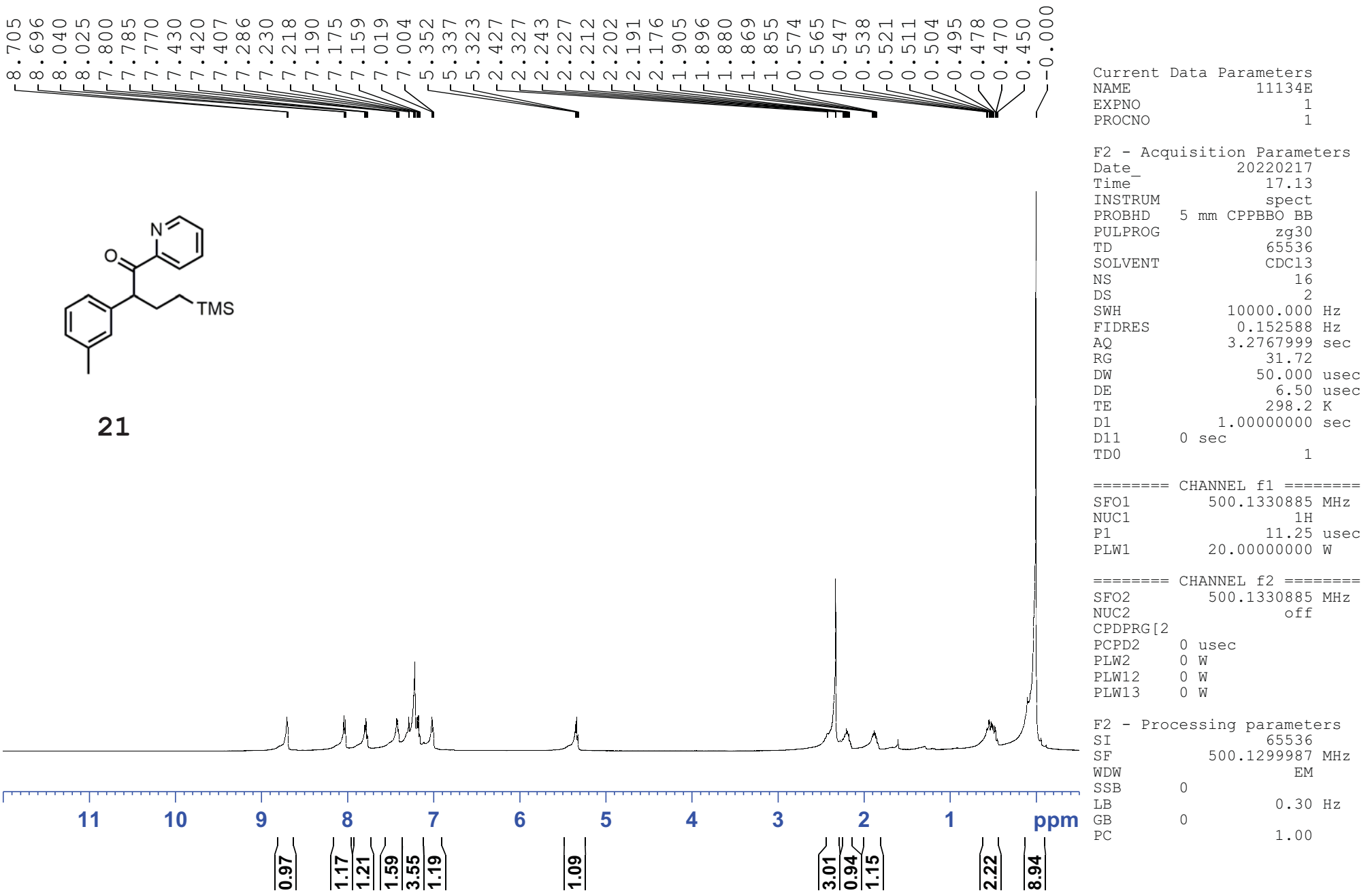


```
Current Data Parameters
NAME                  11134E
EXPNO                      1
PROCNO                     1

F2 - Acquisition Parameters
Date_              20220217
Time                  17.13
INSTRUM               spect
PROBHD    5 mm CPPBBO BB
PULPROG                zg30
TD                    65536
SOLVENT               CDCl3
NS                       16
DS                        2
SWH              10000.000 Hz
FIDRES            0.152588 Hz
AQ               3.2767999 sec
RG                    31.72
DW                  50.000 usec
DE                   6.50 usec
TE                   298.2 K
D1             1.00000000 sec
D11       0 sec
TD0                       1

======== CHANNEL f1 ========
SFO1           500.1330885 MHz
NUC1                     1H
P1                   11.25 usec
PLW1          20.00000000 W

======== CHANNEL f2 ========
SFO2           500.1330885 MHz
NUC2                   off
CPDPRG[2
PCPD2      0 usec
PLW2       0 W
PLW12      0 W
PLW13      0 W

F2 - Processing parameters
SI                    65536
SF           500.1299987 MHz
WDW                     EM
SSB        0
LB                  0.30 Hz
GB         0
PC                  1.00
```

Supplementary Figure 37. ^{1}H-NMR of compound **21**, recorded at 500 MHz and 25 °C in $CDCl_3$.

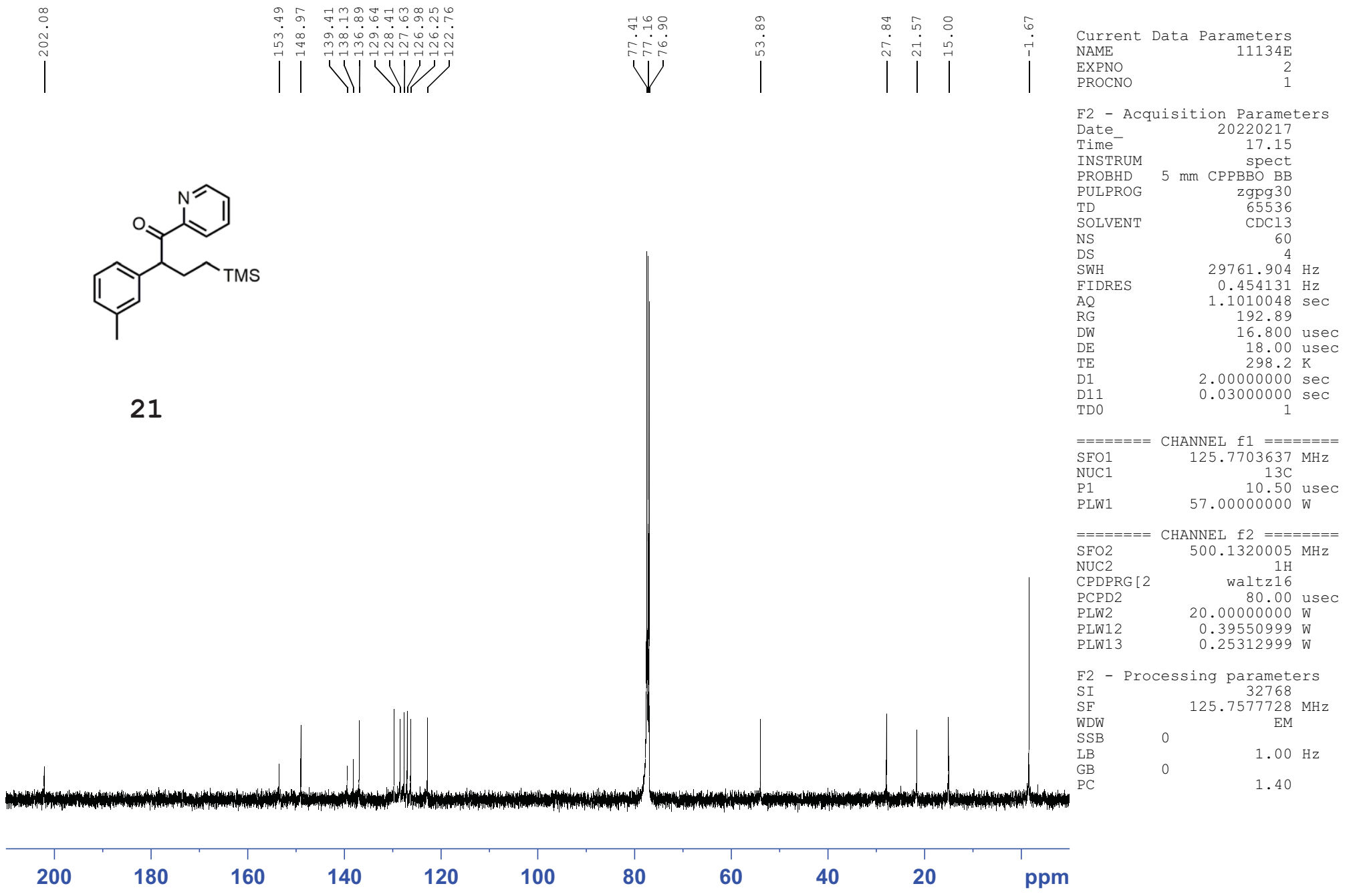


```
Current Data Parameters
NAME                 11134E
EXPNO                     2
PROCNO                    1

F2 - Acquisition Parameters
Date_              20220217
Time                 17.15
INSTRUM              spect
PROBHD   5 mm CPPBBO BB
PULPROG             zgpg30
TD                   65536
SOLVENT              CDCl3
NS                      60
DS                       4
SWH              29761.904 Hz
FIDRES           0.454131 Hz
AQ               1.1010048 sec
RG                  192.89
DW                  16.800 usec
DE                  18.00 usec
TE                  298.2 K
D1             2.00000000 sec
D11            0.03000000 sec
TD0                      1

======== CHANNEL f1 ========
SFO1           125.7703637 MHz
NUC1                   13C
P1                  10.50 usec
PLW1           57.00000000 W

======== CHANNEL f2 ========
SFO2           500.1320005 MHz
NUC2                   1H
CPDPRG[2           waltz16
PCPD2               80.00 usec
PLW2           20.00000000 W
PLW12           0.39550999 W
PLW13           0.25312999 W

F2 - Processing parameters
SI                    32768
SF             125.7577728 MHz
WDW                      EM
SSB          0
LB                   1.00 Hz
GB           0
PC                   1.40
```

Supplementary Figure 38. ^{13}C-NMR of compound **21**, recorded at 126 MHz and 25 °C in $CDCl_3$.

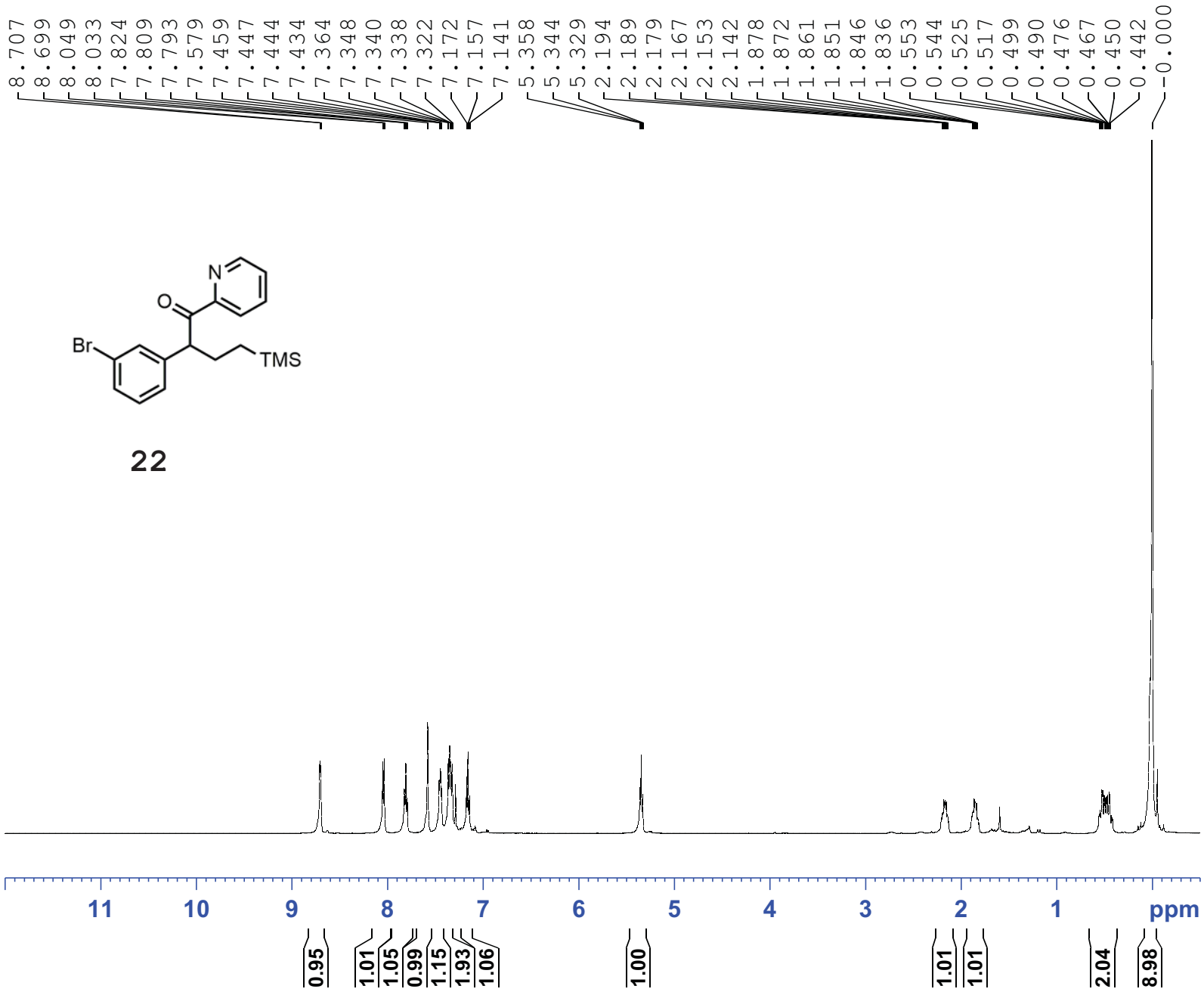


```
Current Data Parameters
NAME                  11132A
EXPNO                      1
PROCNO                     1

F2 - Acquisition Parameters
Date_              20220214
Time                   9.20
INSTRUM               spect
PROBHD   5 mm CPPBBO BB
PULPROG                zg30
TD                    65536
SOLVENT               CDCl3
NS                       16
DS                        2
SWH              10000.000 Hz
FIDRES               0.152588 Hz
AQ               3.2767999 sec
RG                    31.72
DW                   50.000 usec
DE                    6.50 usec
TE                   298.2 K
D1             1.00000000 sec
D11       0 sec
TD0                        1

======== CHANNEL f1 ========
SFO1          500.1330885 MHz
NUC1                      1H
P1                    11.25 usec
PLW1          20.00000000 W

======== CHANNEL f2 ========
SFO2          500.1330885 MHz
NUC2                     off
CPDPRG[2
PCPD2      0 usec
PLW2       0 W
PLW12      0 W
PLW13      0 W

F2 - Processing parameters
SI                    65536
SF            500.1299996 MHz
WDW                      EM
SSB        0
LB                    0.30 Hz
GB         0
PC                    1.00
```

Supplementary Figure 39. ^{1}H-NMR of compound **22**, recorded at 500 MHz and 25 °C in $CDCl_3$.

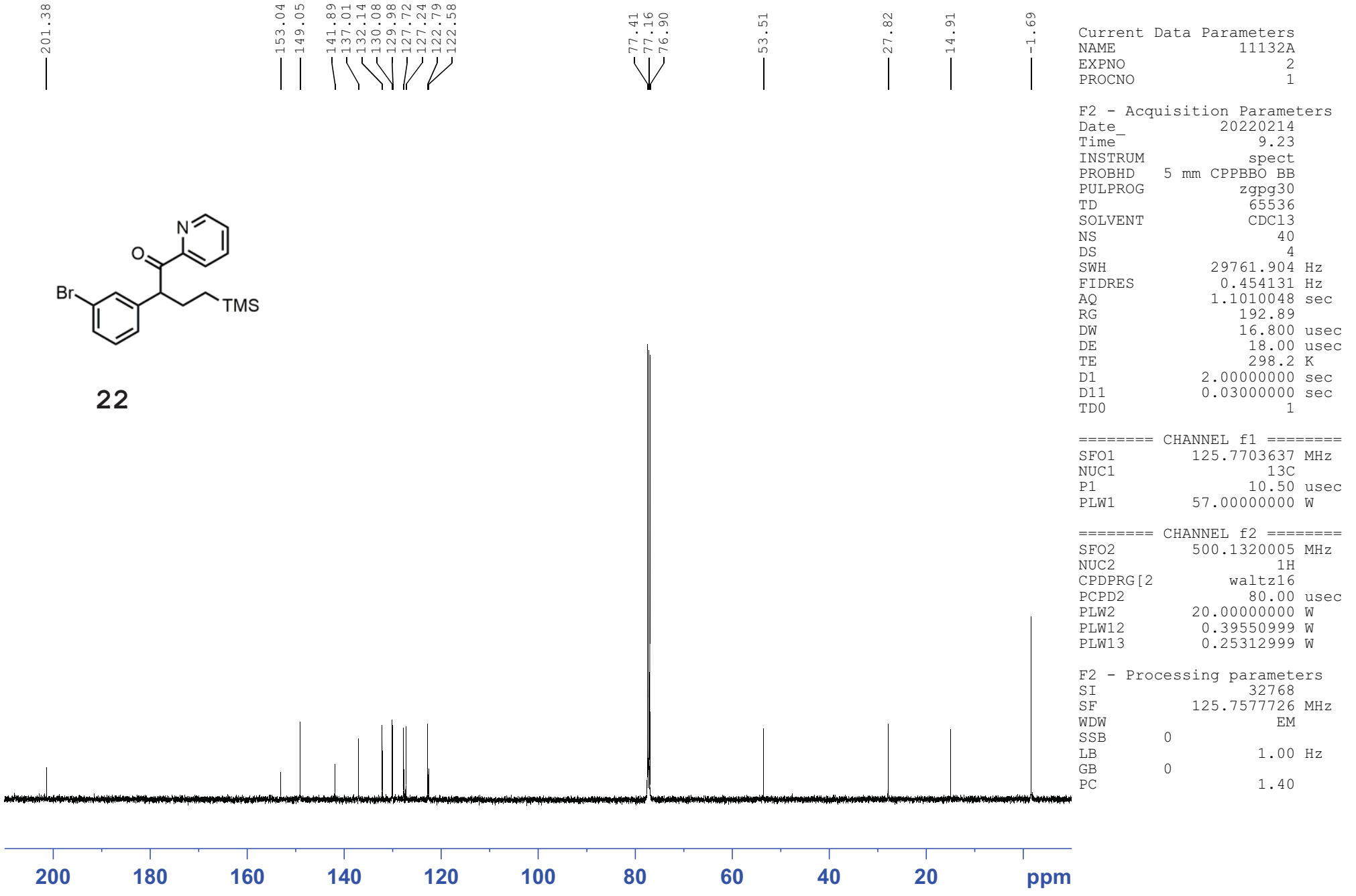


```
Current Data Parameters
NAME                 11132A
EXPNO                     2
PROCNO                    1

F2 - Acquisition Parameters
Date_              20220214
Time                   9.23
INSTRUM               spect
PROBHD   5 mm CPPBBO BB
PULPROG              zgpg30
TD                    65536
SOLVENT               CDCl3
NS                       40
DS                        4
SWH              29761.904 Hz
FIDRES            0.454131 Hz
AQ                1.1010048 sec
RG                   192.89
DW                  16.800 usec
DE                  18.00 usec
TE                  298.2 K
D1              2.00000000 sec
D11             0.03000000 sec
TD0                       1

======== CHANNEL f1 ========
SFO1           125.7703637 MHz
NUC1                    13C
P1                  10.50 usec
PLW1           57.00000000 W

======== CHANNEL f2 ========
SFO2           500.1320005 MHz
NUC2                    1H
CPDPRG[2           waltz16
PCPD2               80.00 usec
PLW2           20.00000000 W
PLW12           0.39550999 W
PLW13           0.25312999 W

F2 - Processing parameters
SI                    32768
SF             125.7577726 MHz
WDW                      EM
SSB          0
LB                   1.00 Hz
GB           0
PC                   1.40
```

Supplementary Figure 40. ^{13}C-NMR of compound **22**, recorded at 126 MHz and 25 °C in $CDCl_3$.

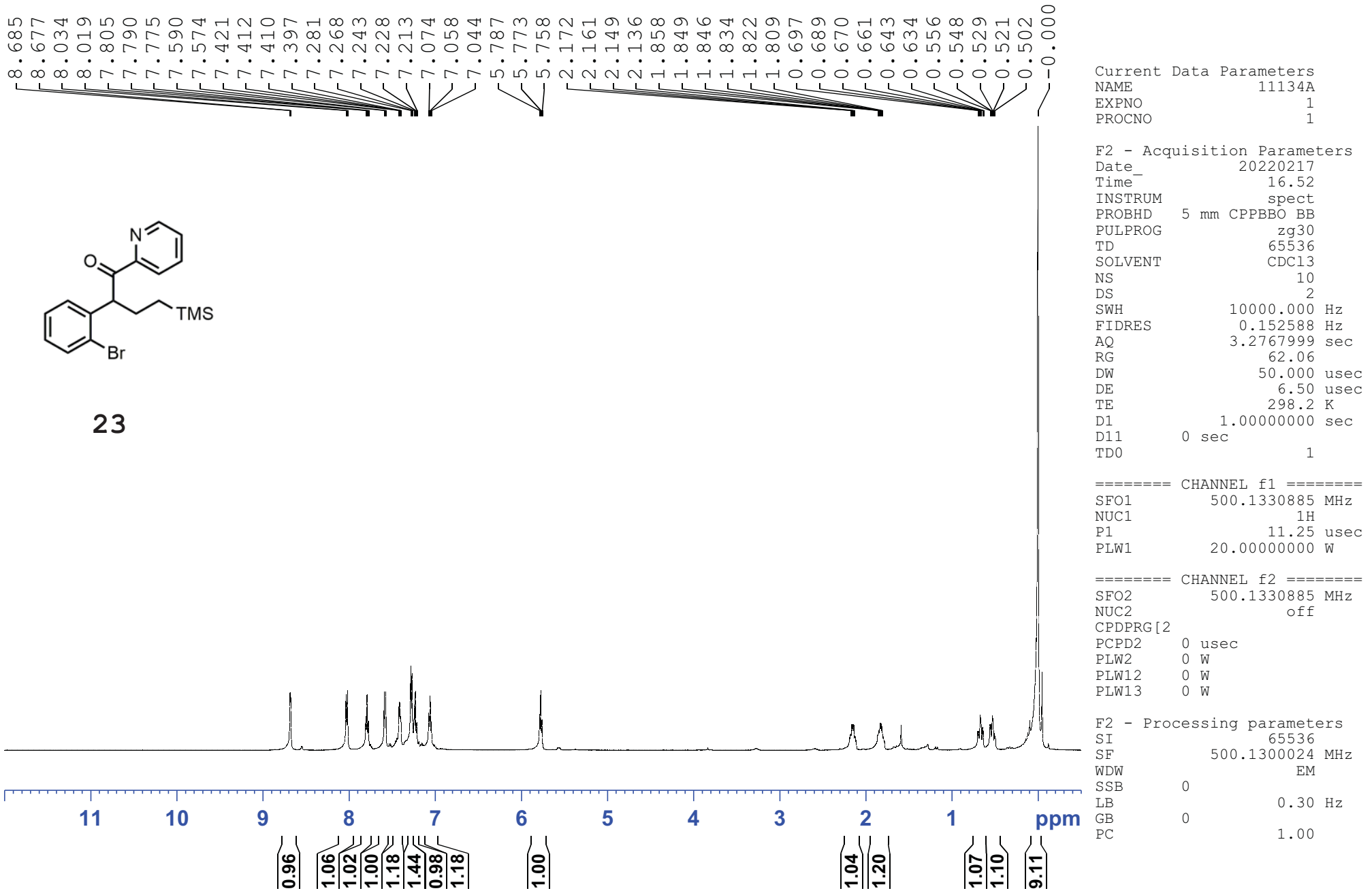


```
Current Data Parameters
NAME                  11134A
EXPNO                      1
PROCNO                     1

F2 - Acquisition Parameters
Date_              20220217
Time                  16.52
INSTRUM               spect
PROBHD    5 mm CPPBBO BB
PULPROG                zg30
TD                    65536
SOLVENT               CDCl3
NS                       10
DS                        2
SWH              10000.000 Hz
FIDRES            0.152588 Hz
AQ               3.2767999 sec
RG                    62.06
DW                  50.000 usec
DE                   6.50 usec
TE                   298.2 K
D1             1.00000000 sec
D11       0 sec
TD0                       1

======== CHANNEL f1 ========
SFO1           500.1330885 MHz
NUC1                     1H
P1                   11.25 usec
PLW1          20.00000000 W

======== CHANNEL f2 ========
SFO2           500.1330885 MHz
NUC2                   off
CPDPRG[2
PCPD2      0 usec
PLW2       0 W
PLW12      0 W
PLW13      0 W

F2 - Processing parameters
SI                    65536
SF               500.1300024 MHz
WDW                      EM
SSB        0
LB                    0.30 Hz
GB         0
PC                    1.00
```

Supplementary Figure 41. ^{1}H-NMR of compound **23**, recorded at 500 MHz and 25 °C in $CDCl_3$.

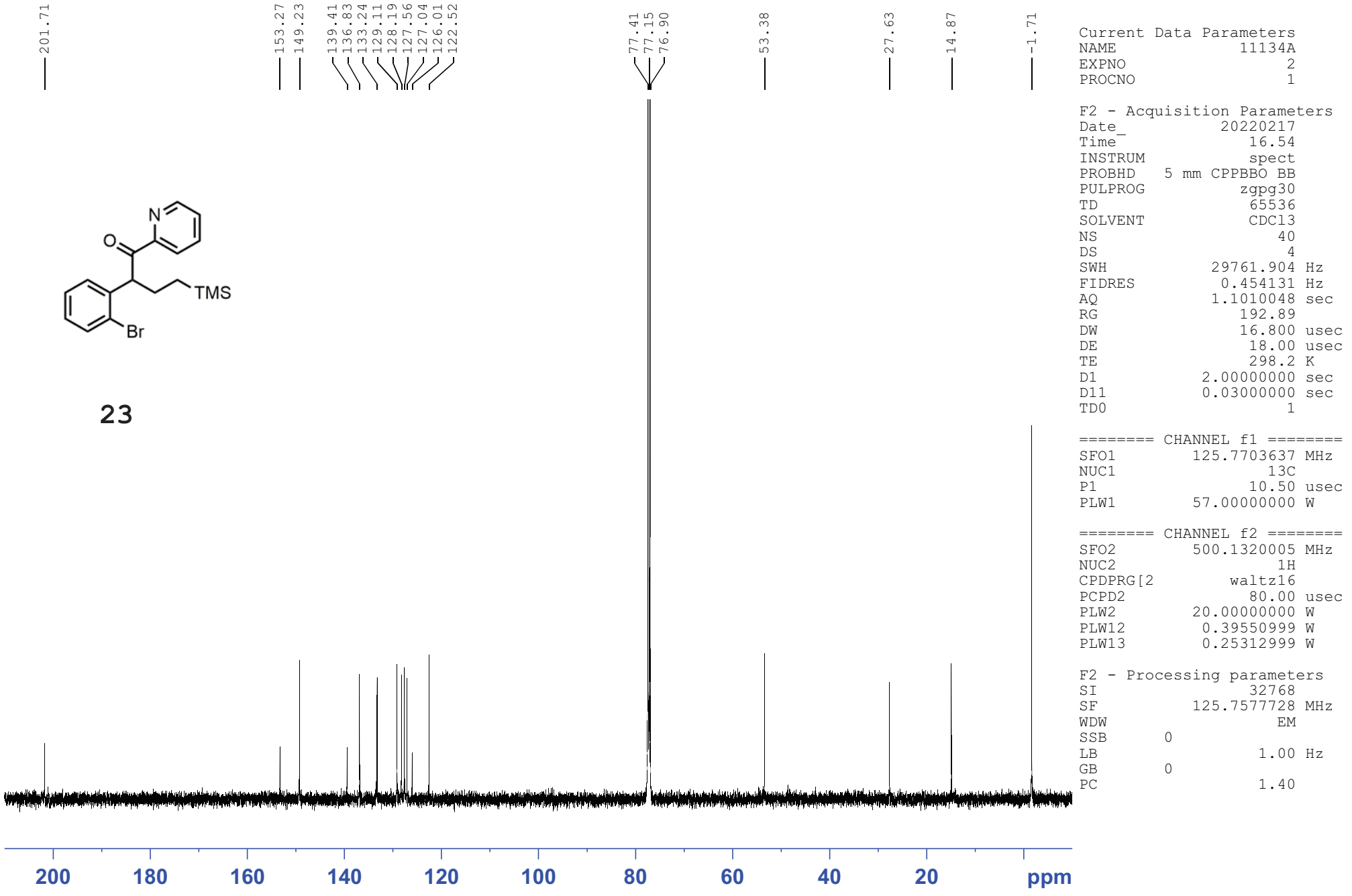

Supplementary Figure 42. ^{13}C-NMR of compound **23**, recorded at 126 MHz and 25 °C in $CDCl_3$.

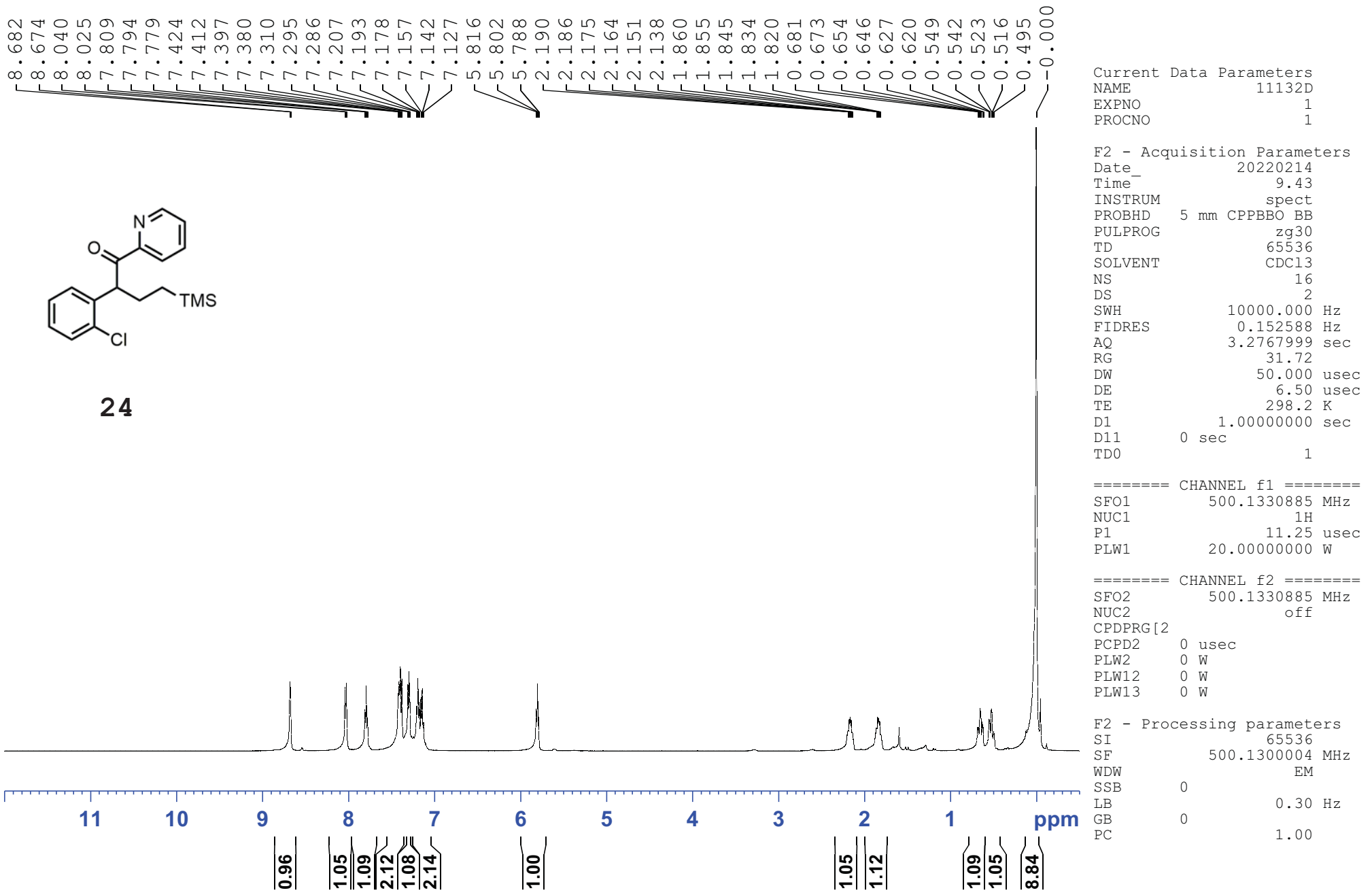


```
Current Data Parameters
NAME                  11132D
EXPNO                      1
PROCNO                     1

F2 - Acquisition Parameters
Date_              20220214
Time                   9.43
INSTRUM               spect
PROBHD   5 mm CPPBBO BB
PULPROG                zg30
TD                    65536
SOLVENT               CDCl3
NS                       16
DS                        2
SWH              10000.000 Hz
FIDRES            0.152588 Hz
AQ               3.2767999 sec
RG                    31.72
DW                  50.000 usec
DE                   6.50 usec
TE                   298.2 K
D1              1.00000000 sec
D11       0 sec
TD0                       1

======== CHANNEL f1 ========
SFO1           500.1330885 MHz
NUC1                     1H
P1                   11.25 usec
PLW1           20.00000000 W

======== CHANNEL f2 ========
SFO2           500.1330885 MHz
NUC2                    off
CPDPRG[2
PCPD2      0 usec
PLW2       0 W
PLW12      0 W
PLW13      0 W

F2 - Processing parameters
SI                    65536
SF           500.1300004 MHz
WDW                      EM
SSB        0
LB                    0.30 Hz
GB         0
PC                    1.00
```

Supplementary Figure 43. ^{1}H-NMR of compound **24**, recorded at 500 MHz and 25 °C in $CDCl_3$.

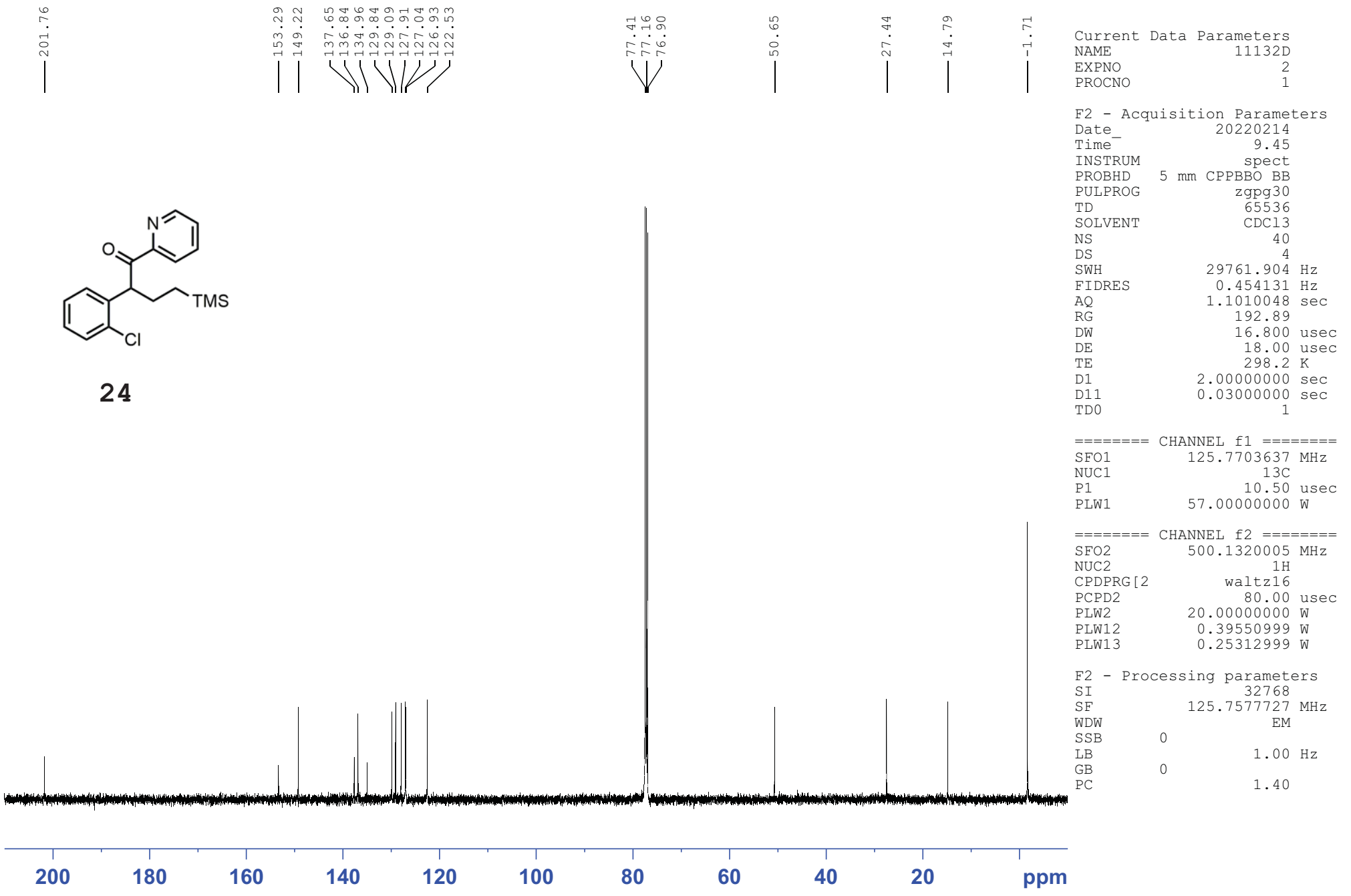


```
Current Data Parameters
NAME                 11132D
EXPNO                     2
PROCNO                    1

F2 - Acquisition Parameters
Date_              20220214
Time                  9.45
INSTRUM              spect
PROBHD   5 mm CPPBBO BB
PULPROG             zgpg30
TD                   65536
SOLVENT              CDCl3
NS                      40
DS                       4
SWH              29761.904 Hz
FIDRES            0.454131 Hz
AQ                1.1010048 sec
RG                   192.89
DW                   16.800 usec
DE                   18.00 usec
TE                   298.2 K
D1             2.00000000 sec
D11            0.03000000 sec
TD0                       1

======== CHANNEL f1 ========
SFO1           125.7703637 MHz
NUC1                    13C
P1                   10.50 usec
PLW1           57.00000000 W

======== CHANNEL f2 ========
SFO2           500.1320005 MHz
NUC2                    1H
CPDPRG[2            waltz16
PCPD2                80.00 usec
PLW2           20.00000000 W
PLW12           0.39550999 W
PLW13           0.25312999 W

F2 - Processing parameters
SI                      32768
SF             125.7577727 MHz
WDW                        EM
SSB          0
LB                    1.00 Hz
GB           0
PC                    1.40
```

Supplementary Figure 44. ^{13}C-NMR of compound **24**, recorded at 126 MHz and 25 °C in $CDCl_3$.

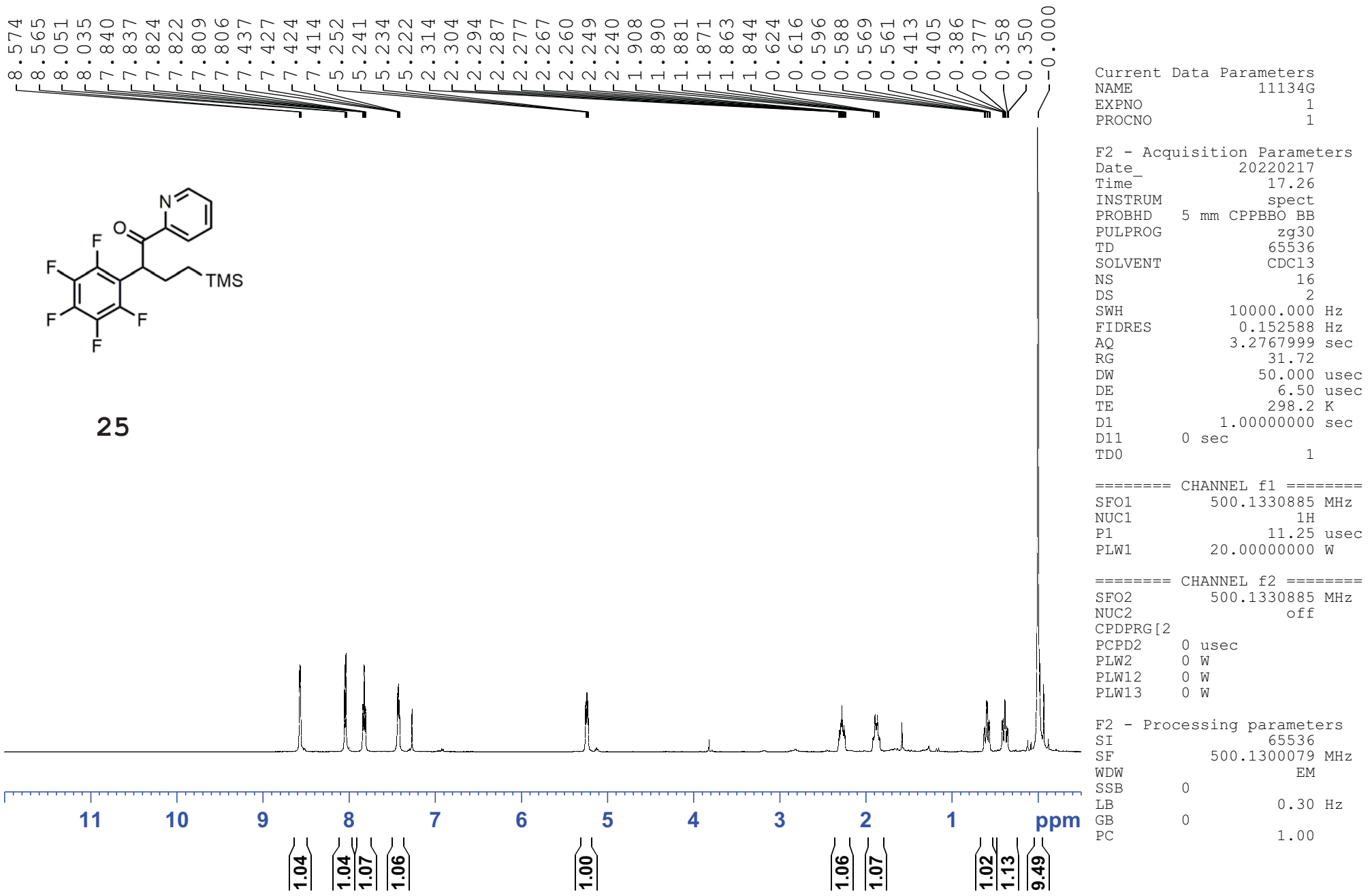


```
Current Data Parameters
NAME                  11134G
EXPNO                      1
PROCNO                     1

F2 - Acquisition Parameters
Date_              20220217
Time                  17.26
INSTRUM               spect
PROBHD    5 mm CPPBBO BB
PULPROG                zg30
TD                    65536
SOLVENT               CDCl3
NS                       16
DS                        2
SWH              10000.000 Hz
FIDRES            0.152588 Hz
AQ                3.2767999 sec
RG                    31.72
DW                   50.000 usec
DE                    6.50 usec
TE                   298.2 K
D1              1.00000000 sec
D11       0 sec
TD0                       1

======== CHANNEL f1 ========
SFO1           500.1330885 MHz
NUC1                      1H
P1                   11.25 usec
PLW1           20.00000000 W

======== CHANNEL f2 ========
SFO2           500.1330885 MHz
NUC2                     off
CPDPRG[2
PCPD2      0 usec
PLW2       0 W
PLW12      0 W
PLW13      0 W

F2 - Processing parameters
SI                    65536
SF           500.1300079 MHz
WDW                      EM
SSB        0
LB                    0.30 Hz
GB         0
PC                    1.00
```

Supplementary Figure 45. ^{1}H-NMR of compound **25**, recorded at 500 MHz and 25 °C in $CDCl_3$.

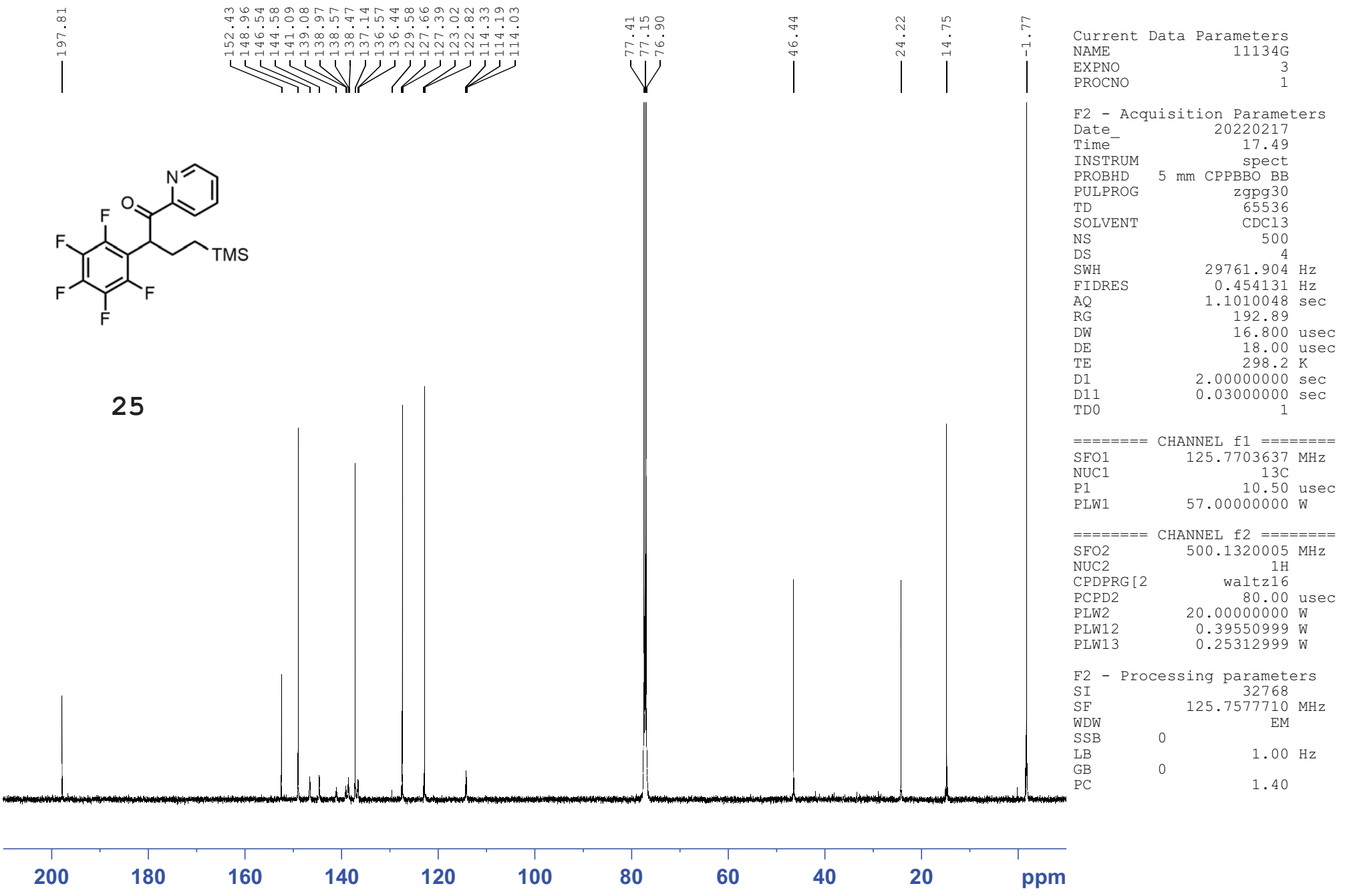

Supplementary Figure 46. ^{13}C-NMR of compound **25**, recorded at 126 MHz and 25 °C in $CDCl_3$.

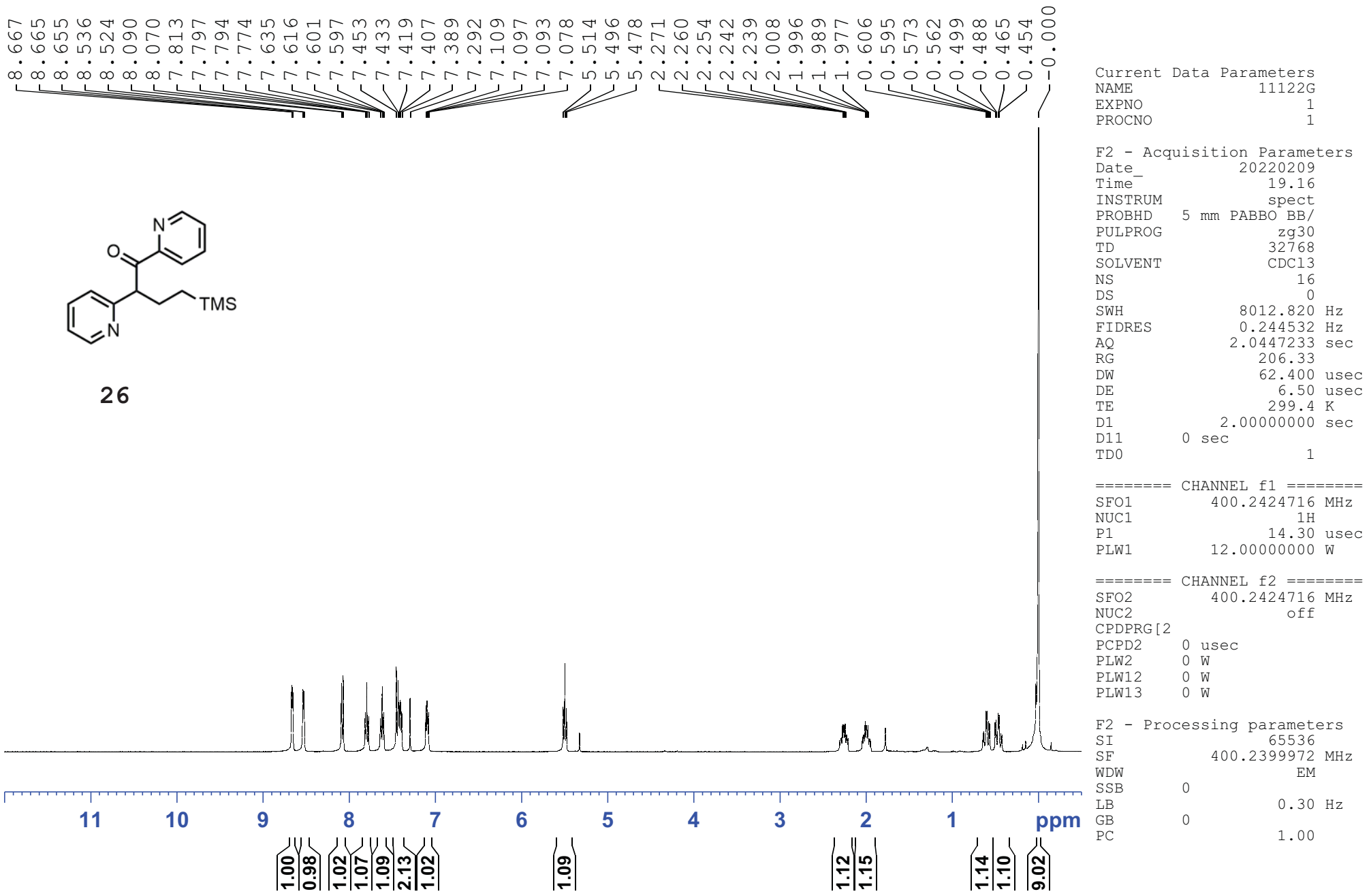


```
Current Data Parameters
NAME                  11122G
EXPNO                      1
PROCNO                     1

F2 - Acquisition Parameters
Date_              20220209
Time                  19.16
INSTRUM               spect
PROBHD    5 mm PABBO BB/
PULPROG                zg30
TD                    32768
SOLVENT               CDCl3
NS                       16
DS                        0
SWH                8012.820 Hz
FIDRES             0.244532 Hz
AQ                2.0447233 sec
RG                   206.33
DW                   62.400 usec
DE                    6.50 usec
TE                   299.4 K
D1              2.00000000 sec
D11       0 sec
TD0                       1

======== CHANNEL f1 ========
SFO1           400.2424716 MHz
NUC1                      1H
P1                    14.30 usec
PLW1           12.00000000 W

======== CHANNEL f2 ========
SFO2           400.2424716 MHz
NUC2                    off
CPDPRG[2
PCPD2      0 usec
PLW2       0 W
PLW12      0 W
PLW13      0 W

F2 - Processing parameters
SI                     65536
SF             400.2399972 MHz
WDW                       EM
SSB        0
LB                    0.30 Hz
GB         0
PC                    1.00
```

Supplementary Figure 47. ^{1}H-NMR of compound **26**, recorded at 400 MHz and 25 °C in $CDCl_3$.

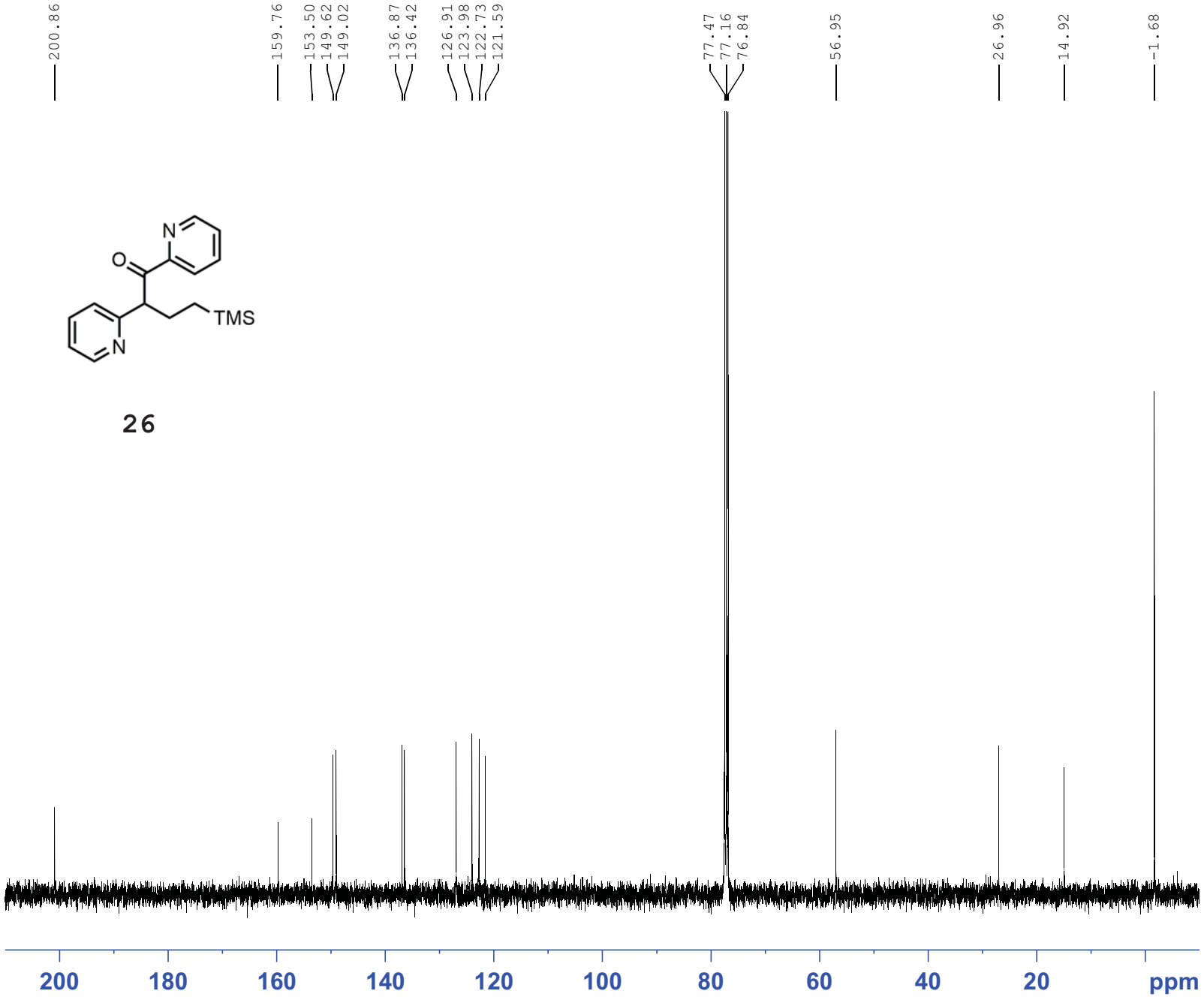


```
Current Data Parameters
NAME                 11122G
EXPNO                     2
PROCNO                    1

F2 - Acquisition Parameters
Date_              20220209
Time                 19.40
INSTRUM              spect
PROBHD   5 mm PABBO BB/
PULPROG             zgpg30
TD                   65536
SOLVENT              CDCl3
NS                     400
DS                       4
SWH             24038.461 Hz
FIDRES           0.366798 Hz
AQ               1.3631488 sec
RG                  206.33
DW                  20.800 usec
DE                   6.50 usec
TE                  300.2 K
D1             2.00000000 sec
D11            0.03000000 sec
TD0                      1

======== CHANNEL f1 ========
SFO1           100.6504916 MHz
NUC1                   13C
P1                  10.00 usec
PLW1           54.00000000 W

======== CHANNEL f2 ========
SFO2           400.2416010 MHz
NUC2                   1H
CPDPRG[2           waltz16
PCPD2               90.00 usec
PLW2           12.00000000 W
PLW12           0.30294999 W
PLW13           0.24539000 W

F2 - Processing parameters
SI                     32768
SF             100.6404146 MHz
WDW                     EM
SSB       0
LB                   1.00 Hz
GB        0
PC                   1.40
```

Supplementary Figure 48. ^{13}C-NMR of compound **26**, recorded at 101 MHz and 25 °C in $CDCl_3$.

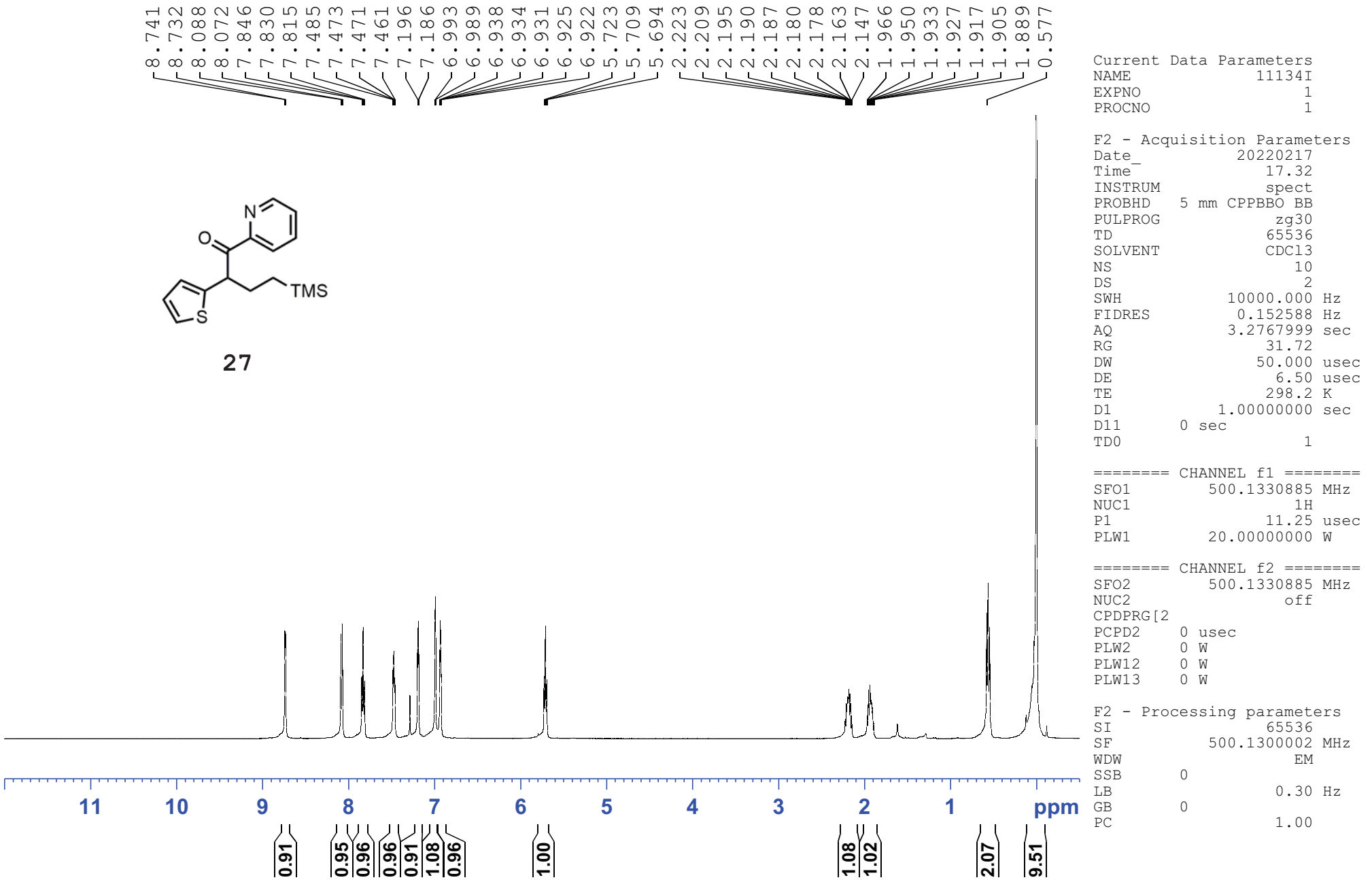

Supplementary Figure 49. ^{1}H-NMR of compound **27**, recorded at 500 MHz and 25 °C in $CDCl_3$.

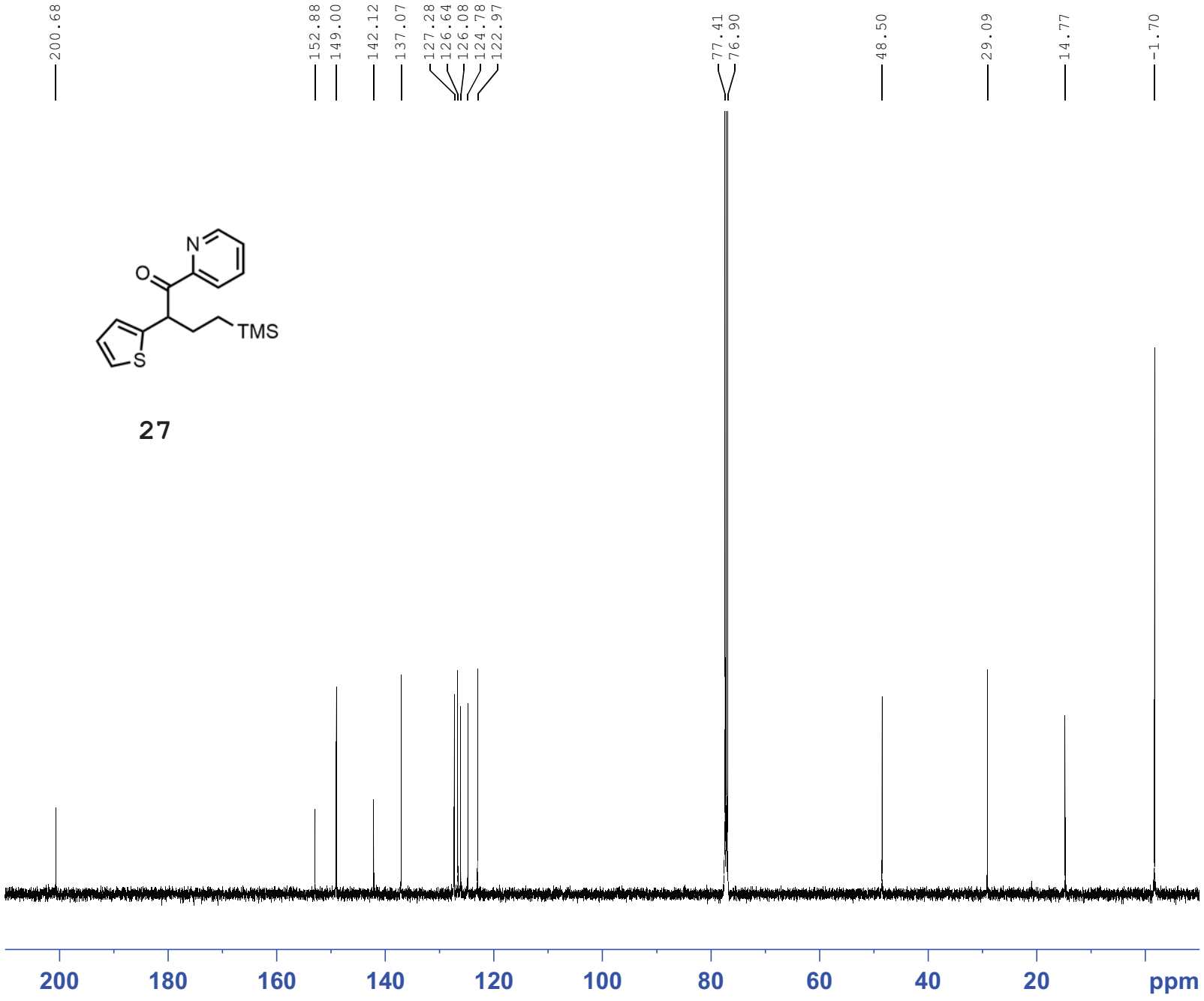


```
Current Data Parameters
NAME                 11134I
EXPNO                     2
PROCNO                    1

F2 - Acquisition Parameters
Date_              20220217
Time                 17.34
INSTRUM              spect
PROBHD   5 mm CPPBBO BB
PULPROG             zgpg30
TD                   65536
SOLVENT              CDCl3
NS                      30
DS                       4
SWH              29761.904 Hz
FIDRES            0.454131 Hz
AQ                1.1010048 sec
RG                   192.89
DW                  16.800 usec
DE                  18.00 usec
TE                  298.2 K
D1              2.00000000 sec
D11             0.03000000 sec
TD0                      1

======== CHANNEL f1 ========
SFO1           125.7703637 MHz
NUC1                   13C
P1                  10.50 usec
PLW1           57.00000000 W

======== CHANNEL f2 ========
SFO2           500.1320005 MHz
NUC2                   1H
CPDPRG[2           waltz16
PCPD2               80.00 usec
PLW2           20.00000000 W
PLW12           0.39550999 W
PLW13           0.25312999 W

F2 - Processing parameters
SI                    32768
SF             125.7577730 MHz
WDW                      EM
SSB       0
LB                   1.00 Hz
GB        0
PC                   1.40
```

Supplementary Figure 50. ^{13}C-NMR of compound **27**, recorded at 126 MHz and 25 °C in $CDCl_3$.

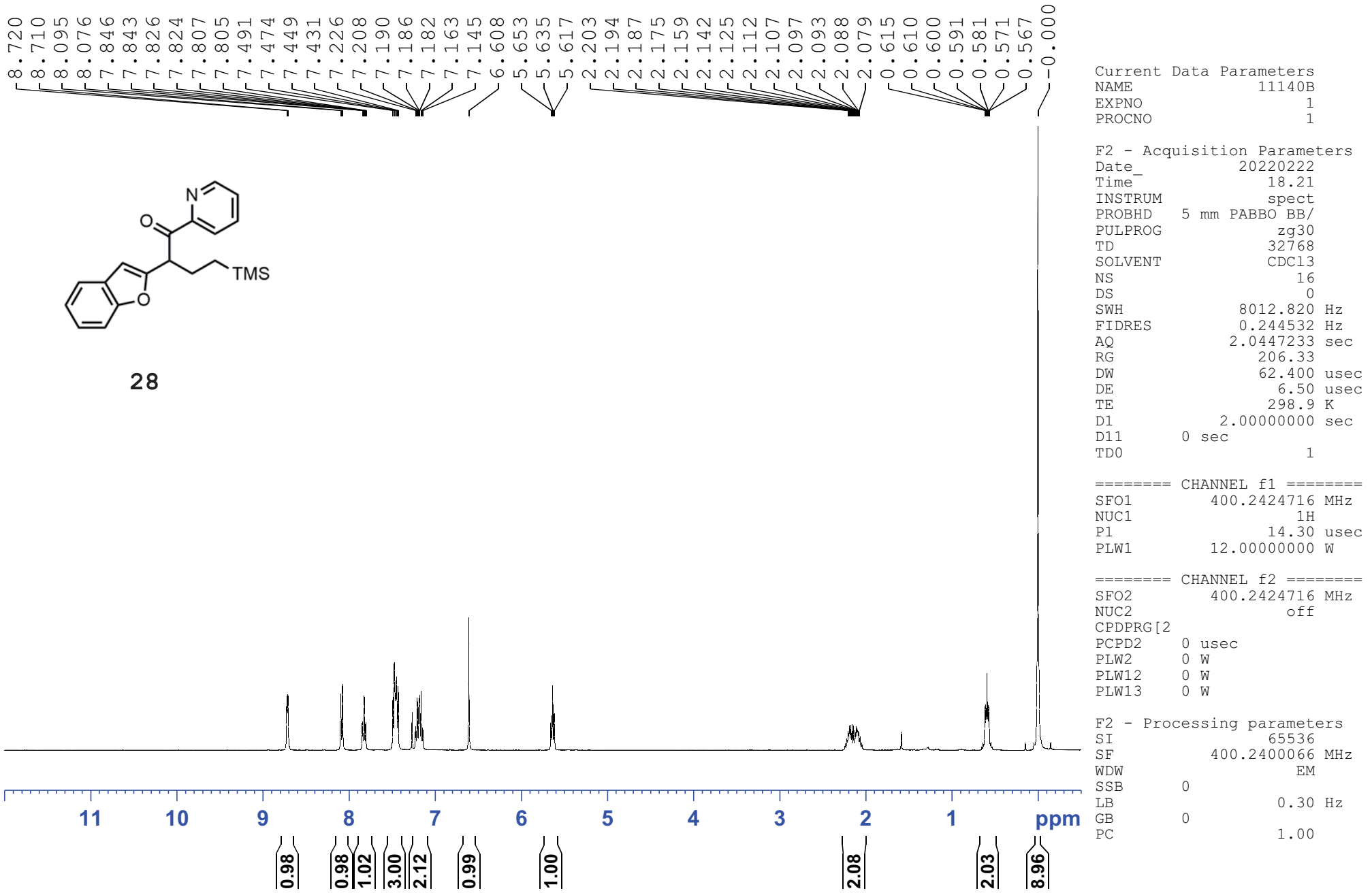


```
Current Data Parameters
NAME                  11140B
EXPNO                      1
PROCNO                     1

F2 - Acquisition Parameters
Date_              20220222
Time                  18.21
INSTRUM               spect
PROBHD    5 mm PABBO BB/
PULPROG                zg30
TD                    32768
SOLVENT               CDCl3
NS                       16
DS                        0
SWH               8012.820 Hz
FIDRES             0.244532 Hz
AQ                2.0447233 sec
RG                   206.33
DW                   62.400 usec
DE                    6.50 usec
TE                   298.9 K
D1             2.00000000 sec
D11       0 sec
TD0                       1

======== CHANNEL f1 ========
SFO1           400.2424716 MHz
NUC1                      1H
P1                   14.30 usec
PLW1          12.00000000 W

======== CHANNEL f2 ========
SFO2           400.2424716 MHz
NUC2                    off
CPDPRG[2
PCPD2      0 usec
PLW2       0 W
PLW12      0 W
PLW13      0 W

F2 - Processing parameters
SI                     65536
SF             400.2400066 MHz
WDW                       EM
SSB        0
LB                   0.30 Hz
GB         0
PC                   1.00
```

Supplementary Figure 51. ^{1}H-NMR of compound **28**, recorded at 400 MHz and 25 °C in $CDCl_3$.

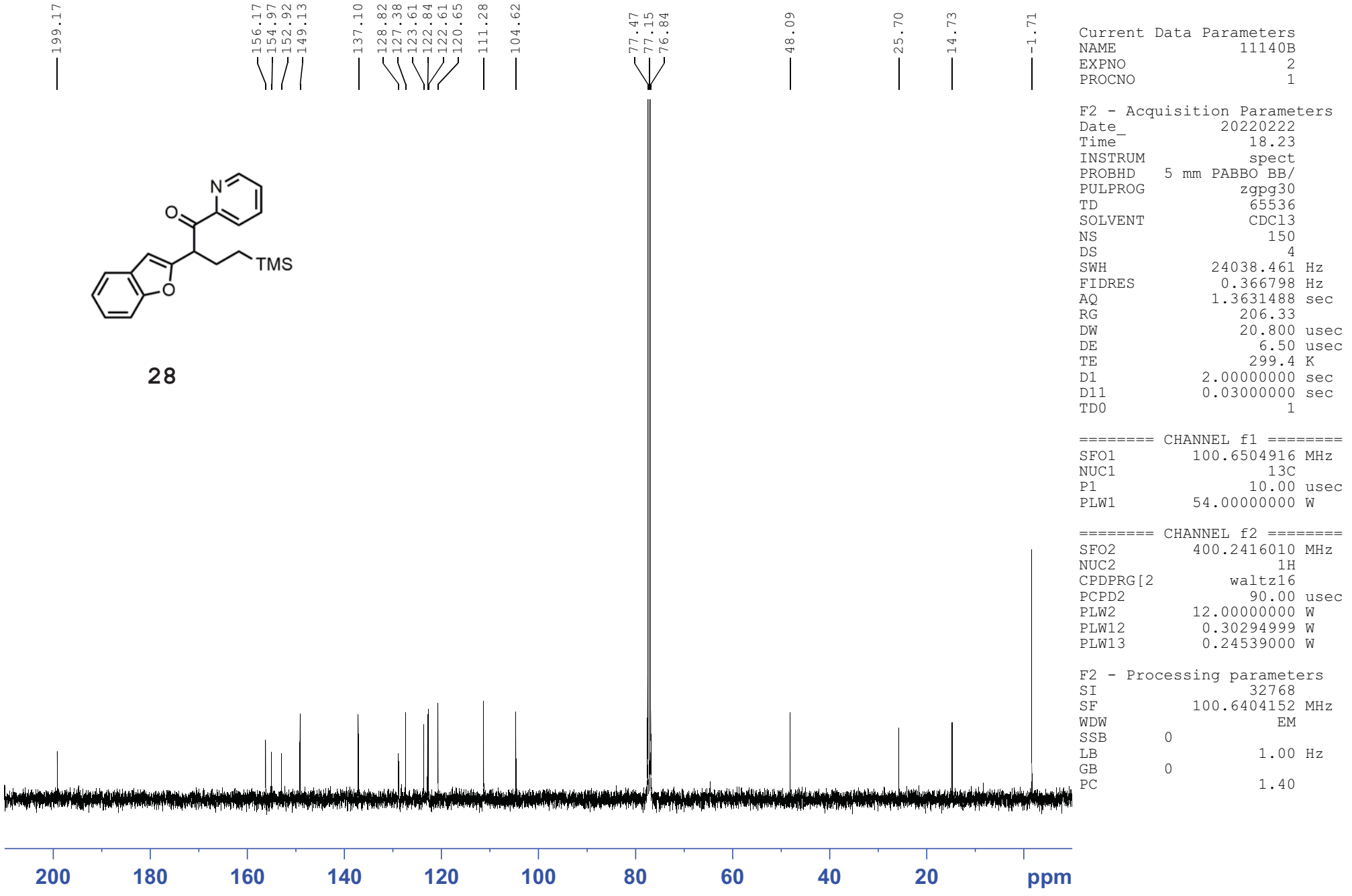


```
Current Data Parameters
NAME                 11140B
EXPNO                     2
PROCNO                    1

F2 - Acquisition Parameters
Date_              20220222
Time                 18.23
INSTRUM              spect
PROBHD   5 mm PABBO BB/
PULPROG             zgpg30
TD                   65536
SOLVENT              CDCl3
NS                     150
DS                       4
SWH             24038.461 Hz
FIDRES           0.366798 Hz
AQ               1.3631488 sec
RG                  206.33
DW                  20.800 usec
DE                   6.50 usec
TE                  299.4 K
D1              2.00000000 sec
D11             0.03000000 sec
TD0                      1

======== CHANNEL f1 ========
SFO1           100.6504916 MHz
NUC1                   13C
P1                  10.00 usec
PLW1           54.00000000 W

======== CHANNEL f2 ========
SFO2           400.2416010 MHz
NUC2                   1H
CPDPRG[2           waltz16
PCPD2               90.00 usec
PLW2           12.00000000 W
PLW12           0.30294999 W
PLW13           0.24539000 W

F2 - Processing parameters
SI                   32768
SF             100.6404152 MHz
WDW                    EM
SSB          0
LB                   1.00 Hz
GB           0
PC                   1.40
```

Supplementary Figure 52. ^{13}C-NMR of compound **28**, recorded at 101 MHz and 25 °C in $CDCl_3$.

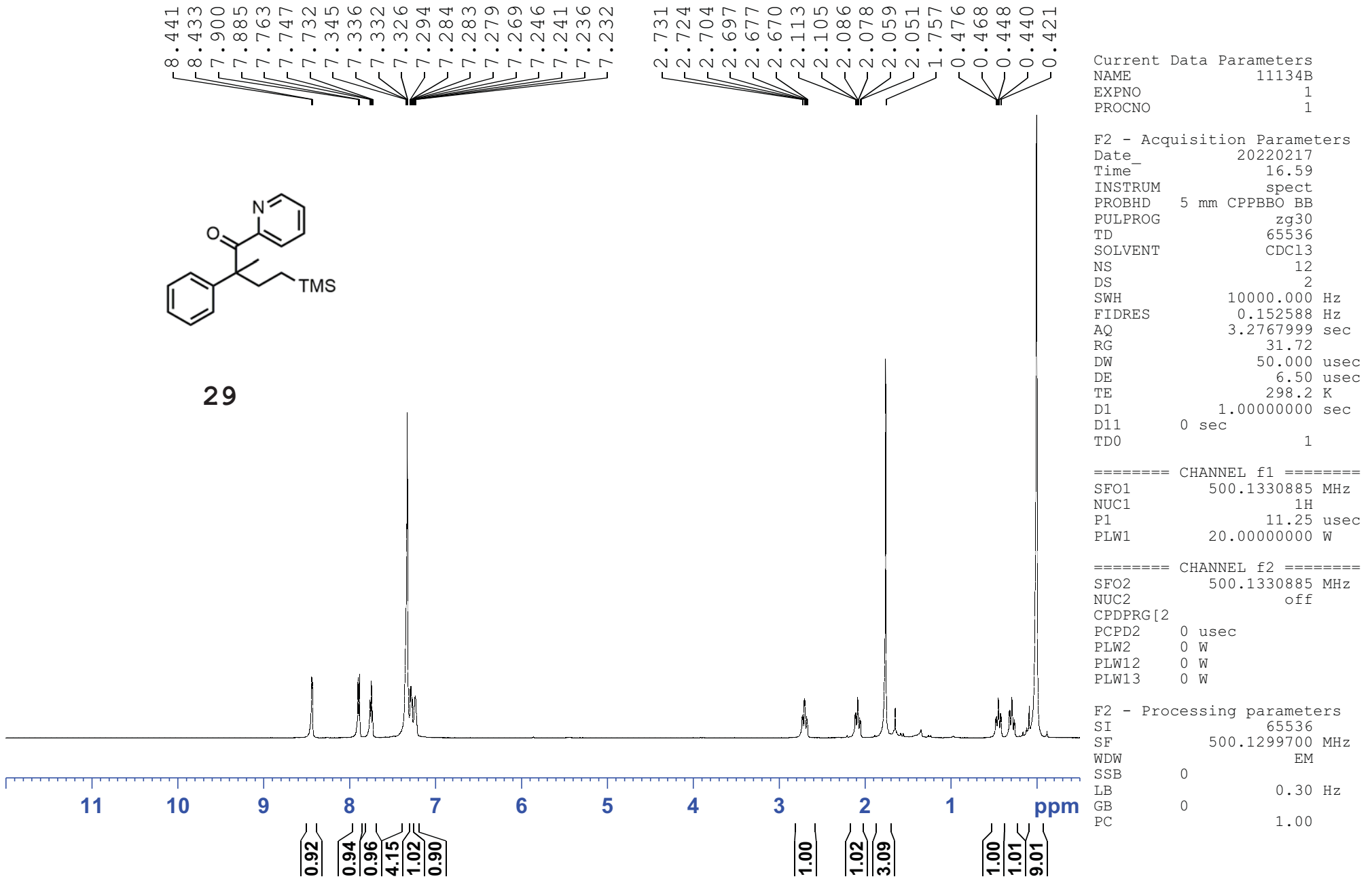


```
Current Data Parameters
NAME                  11134B
EXPNO                      1
PROCNO                     1

F2 - Acquisition Parameters
Date_              20220217
Time                  16.59
INSTRUM               spect
PROBHD   5 mm CPPBBO BB
PULPROG                zg30
TD                    65536
SOLVENT               CDCl3
NS                       12
DS                        2
SWH              10000.000 Hz
FIDRES            0.152588 Hz
AQ               3.2767999 sec
RG                    31.72
DW                  50.000 usec
DE                   6.50 usec
TE                   298.2 K
D1             1.00000000 sec
D11       0 sec
TD0                       1

======== CHANNEL f1 ========
SFO1          500.1330885 MHz
NUC1                     1H
P1                   11.25 usec
PLW1          20.00000000 W

======== CHANNEL f2 ========
SFO2          500.1330885 MHz
NUC2                   off
CPDPRG[2
PCPD2      0 usec
PLW2       0 W
PLW12      0 W
PLW13      0 W

F2 - Processing parameters
SI                    65536
SF          500.1299700 MHz
WDW                      EM
SSB        0
LB                    0.30 Hz
GB         0
PC                    1.00
```

Supplementary Figure 53. ^{1}H-NMR of compound **29**, recorded at 500 MHz and 25 °C in $CDCl_3$.

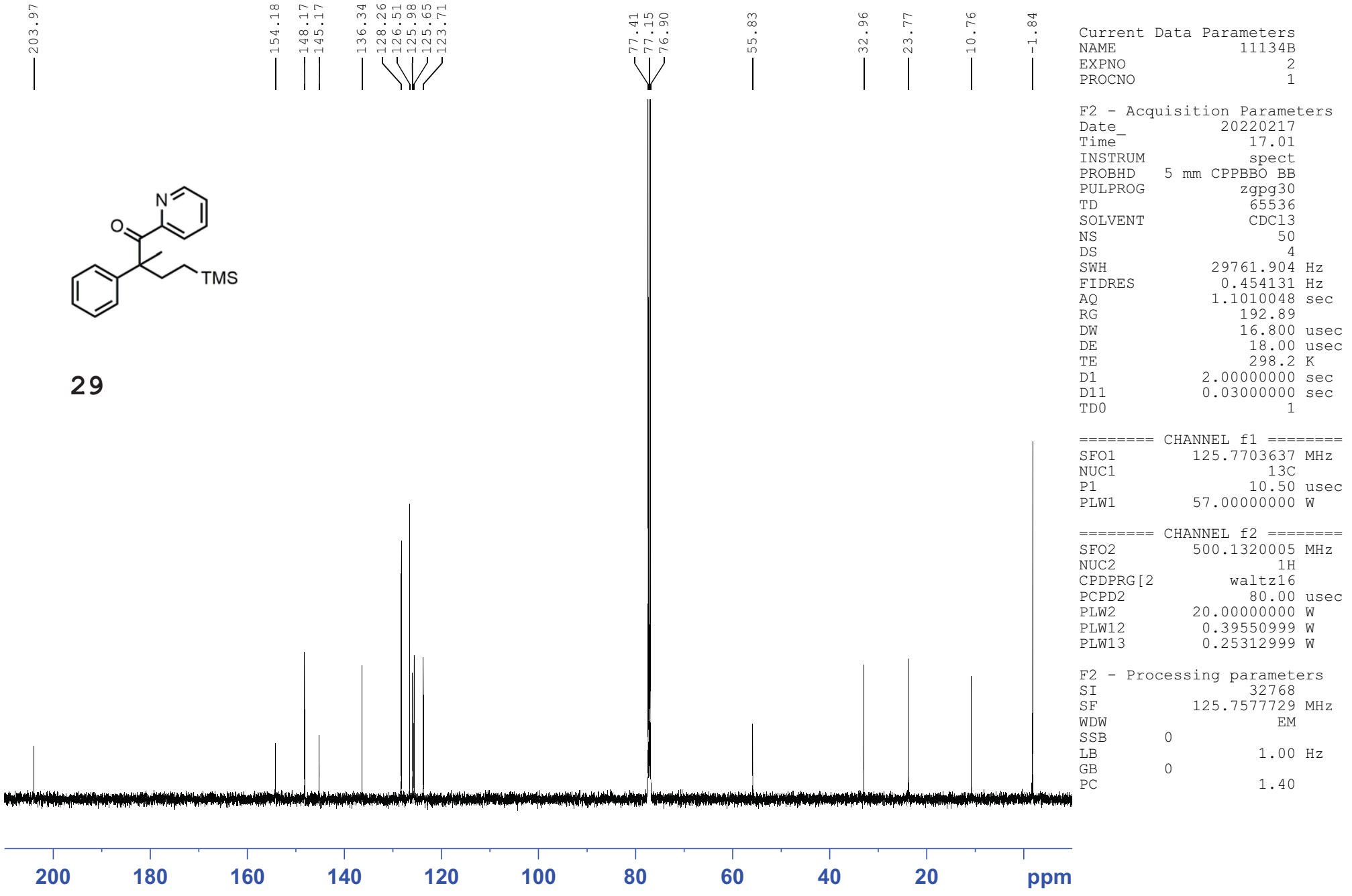


```
Current Data Parameters
NAME                 11134B
EXPNO                     2
PROCNO                    1

F2 - Acquisition Parameters
Date_              20220217
Time                 17.01
INSTRUM              spect
PROBHD   5 mm CPPBBO BB
PULPROG              zgpg30
TD                    65536
SOLVENT               CDCl3
NS                       50
DS                        4
SWH              29761.904 Hz
FIDRES            0.454131 Hz
AQ               1.1010048 sec
RG                   192.89
DW                   16.800 usec
DE                   18.00 usec
TE                   298.2 K
D1             2.00000000 sec
D11            0.03000000 sec
TD0                       1

======== CHANNEL f1 ========
SFO1           125.7703637 MHz
NUC1                    13C
P1                   10.50 usec
PLW1           57.00000000 W

======== CHANNEL f2 ========
SFO2           500.1320005 MHz
NUC2                    1H
CPDPRG[2           waltz16
PCPD2                80.00 usec
PLW2           20.00000000 W
PLW12           0.39550999 W
PLW13           0.25312999 W

F2 - Processing parameters
SI                    32768
SF             125.7577729 MHz
WDW                      EM
SSB          0
LB                    1.00 Hz
GB           0
PC                    1.40
```

Supplementary Figure 54. ^{13}C-NMR of compound **29**, recorded at 126 MHz and 25 °C in $CDCl_3$.

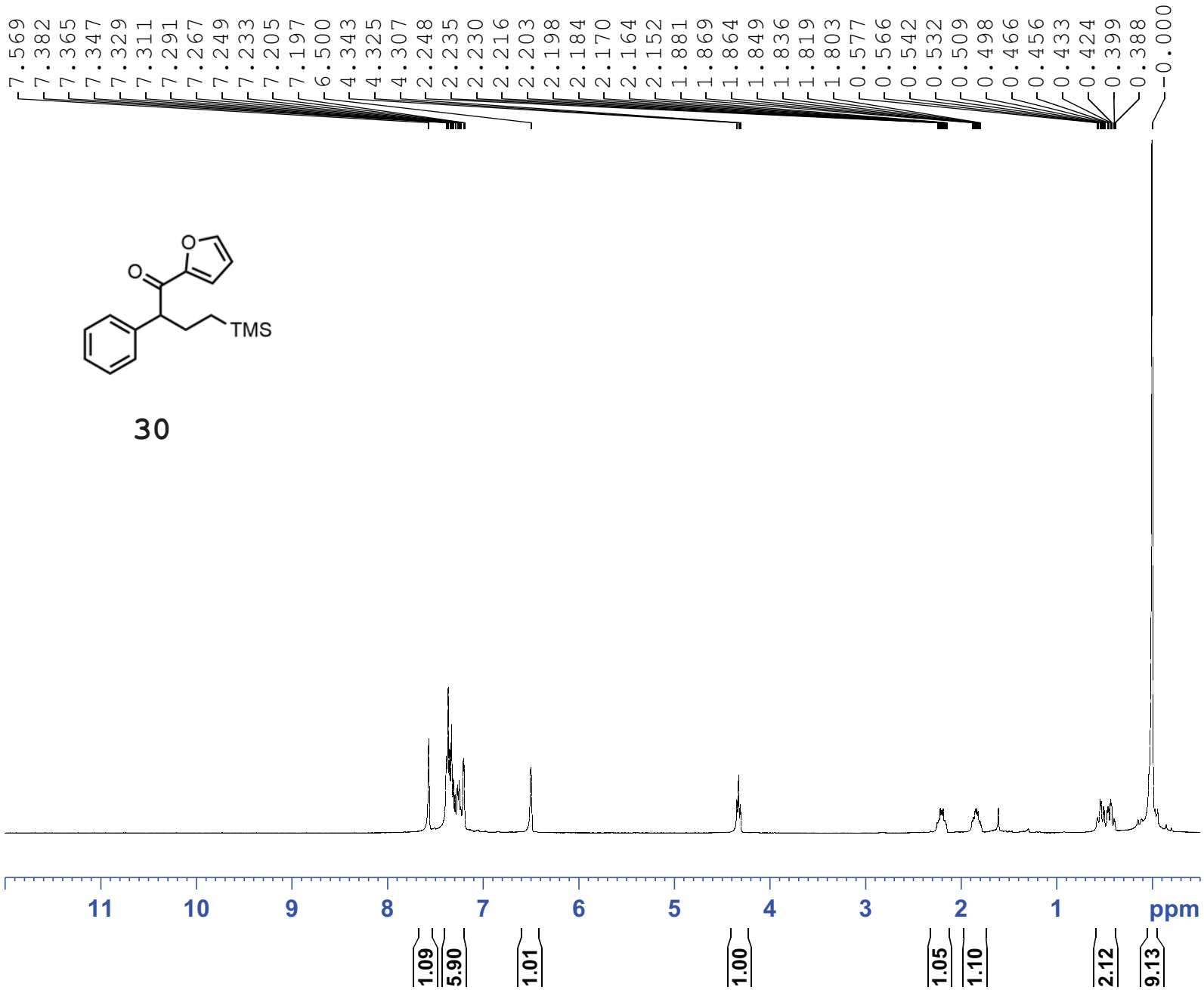


```
Current Data Parameters
NAME                  11148B
EXPNO                      1
PROCNO                     1

F2 - Acquisition Parameters
Date_              20220226
Time                   3.02
INSTRUM               spect
PROBHD    5 mm PABBO BB/
PULPROG                zg30
TD                    32768
SOLVENT               CDCl3
NS                       16
DS                        0
SWH                8012.820 Hz
FIDRES             0.244532 Hz
AQ                2.0447233 sec
RG                   162.77
DW                   62.400 usec
DE                    6.50 usec
TE                   298.5 K
D1              2.00000000 sec
D11       0 sec
TD0                        1

======== CHANNEL f1 ========
SFO1           400.2424716 MHz
NUC1                      1H
P1                    14.30 usec
PLW1           12.00000000 W

======== CHANNEL f2 ========
SFO2           400.2424716 MHz
NUC2                     off
CPDPRG[2
PCPD2      0 usec
PLW2       0 W
PLW12      0 W
PLW13      0 W

F2 - Processing parameters
SI                     65536
SF             400.2399970 MHz
WDW                       EM
SSB        0
LB                    0.30 Hz
GB         0
PC                    1.00
```

Supplementary Figure 55. ^{1}H-NMR of compound **30**, recorded at 400 MHz and 25 °C in $CDCl_3$.

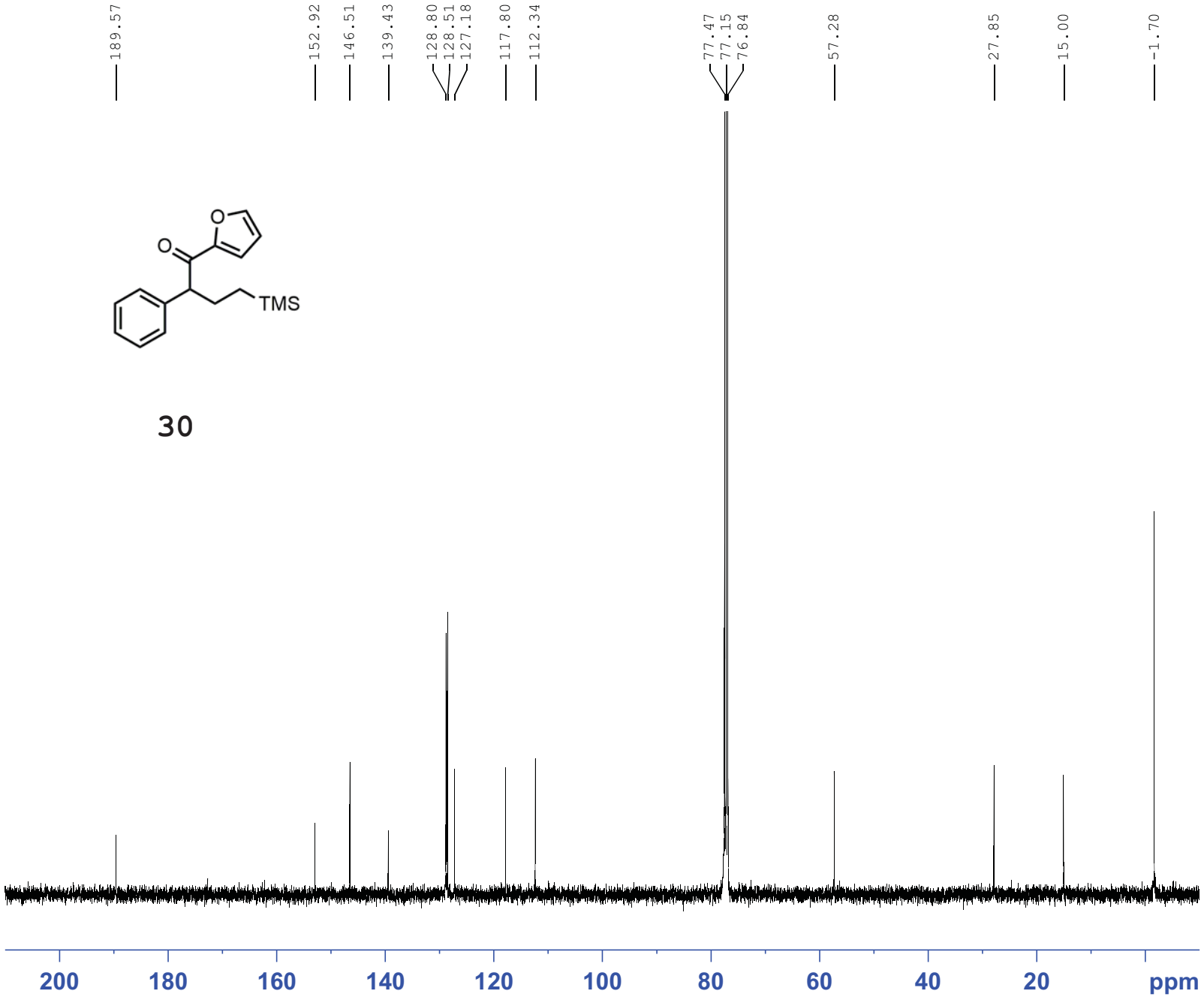

Supplementary Figure 56. ^{13}C-NMR of compound **30**, recorded at 101 MHz and 25 °C in $CDCl_3$.

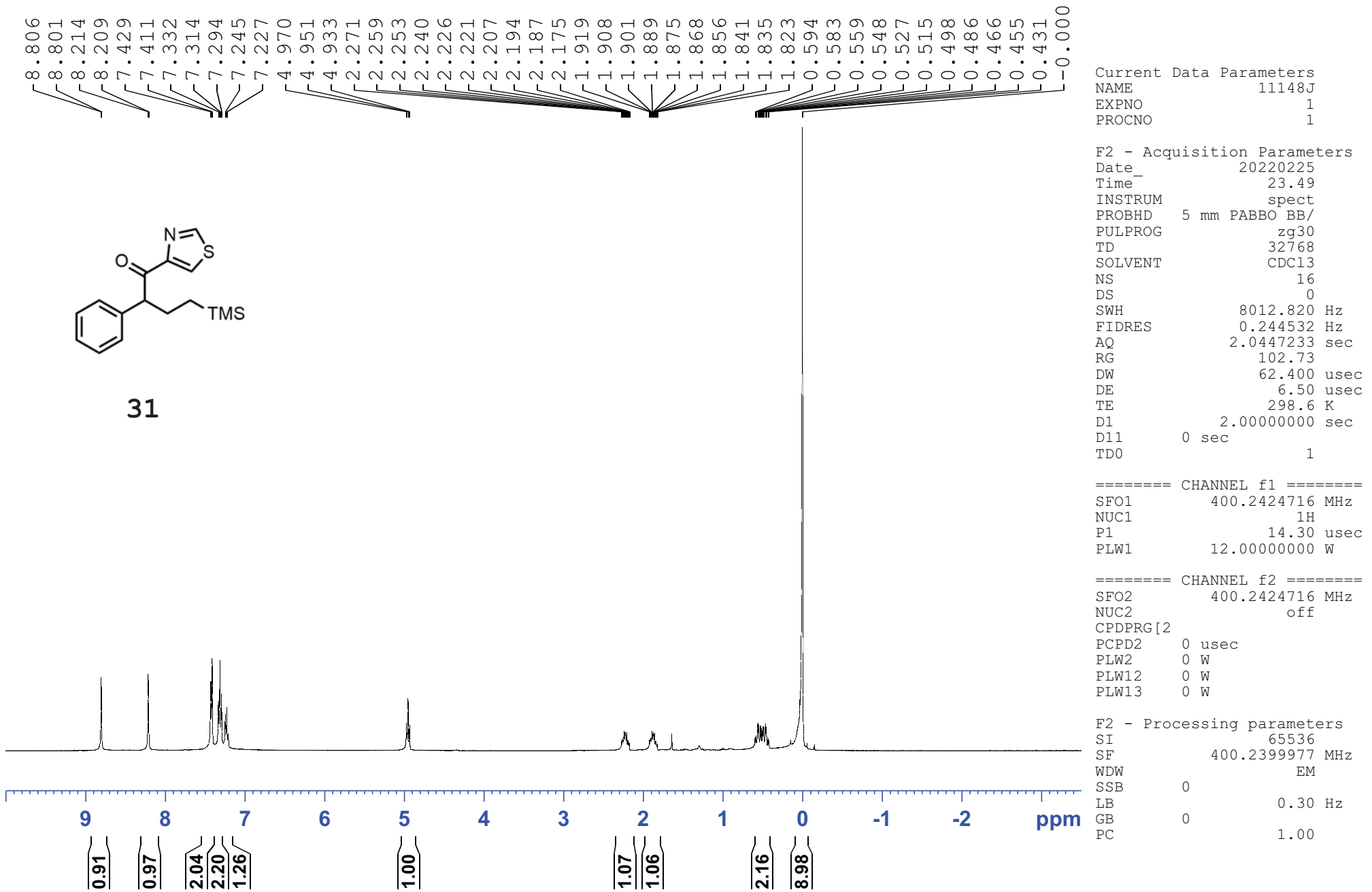


```
Current Data Parameters
NAME                  11148J
EXPNO                      1
PROCNO                     1

F2 - Acquisition Parameters
Date_              20220225
Time                  23.49
INSTRUM               spect
PROBHD    5 mm PABBO BB/
PULPROG                zg30
TD                    32768
SOLVENT               CDCl3
NS                       16
DS                        0
SWH               8012.820 Hz
FIDRES             0.244532 Hz
AQ                2.0447233 sec
RG                   102.73
DW                   62.400 usec
DE                    6.50 usec
TE                   298.6 K
D1              2.00000000 sec
D11       0 sec
TD0                       1

======== CHANNEL f1 ========
SFO1           400.2424716 MHz
NUC1                      1H
P1                   14.30 usec
PLW1           12.00000000 W

======== CHANNEL f2 ========
SFO2           400.2424716 MHz
NUC2                     off
CPDPRG[2
PCPD2      0 usec
PLW2       0 W
PLW12      0 W
PLW13      0 W

F2 - Processing parameters
SI                    65536
SF           400.2399977 MHz
WDW                      EM
SSB        0
LB                   0.30 Hz
GB         0
PC                   1.00
```

Supplementary Figure 57. ^{1}H-NMR of compound **31**, recorded at 400 MHz and 25 °C in $CDCl_3$.

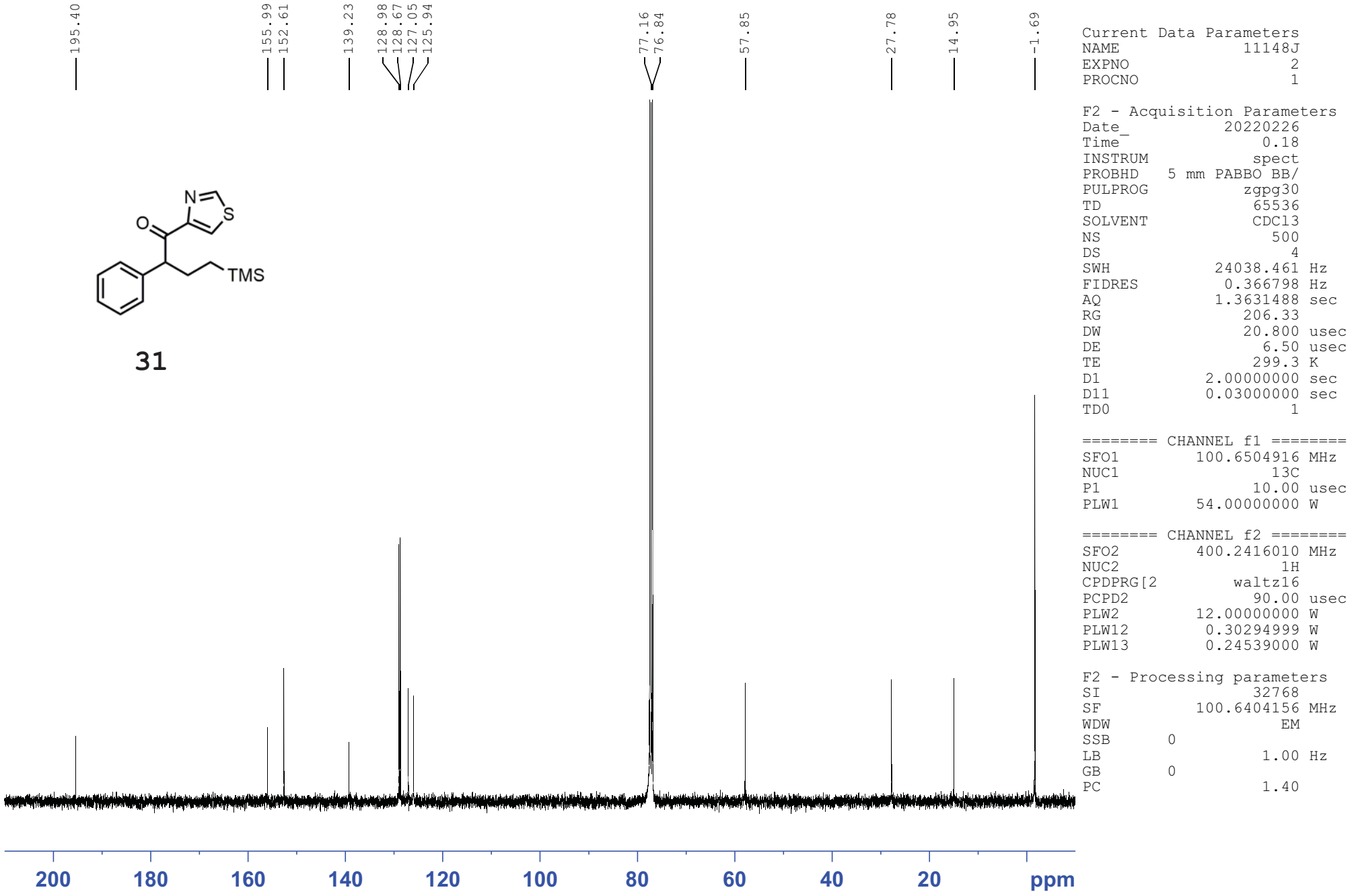

Supplementary Figure 58. ^{13}C-NMR of compound **31**, recorded at 101 MHz and 25 °C in $CDCl_3$.

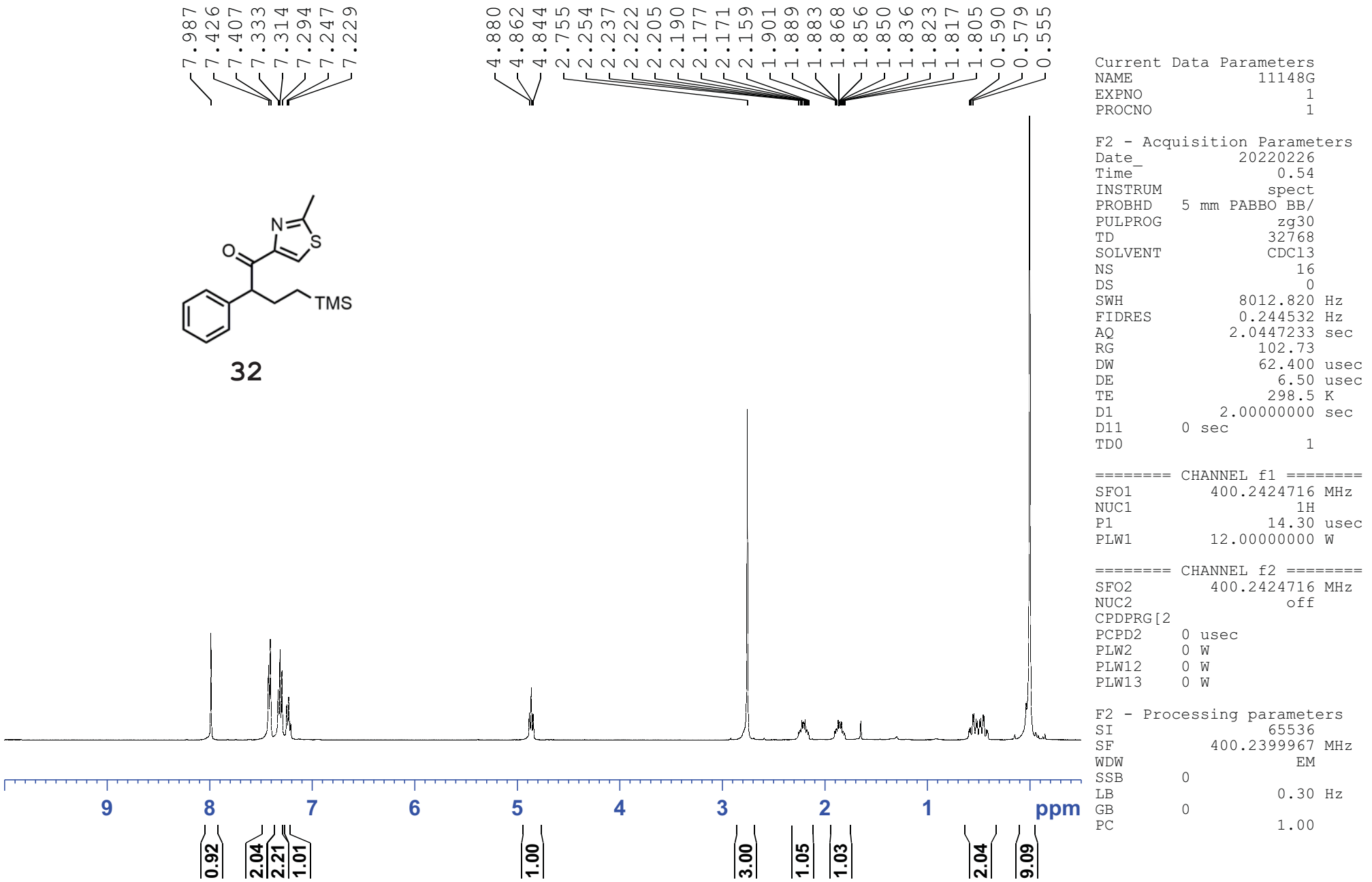


```
Current Data Parameters
NAME                  11148G
EXPNO                      1
PROCNO                     1

F2 - Acquisition Parameters
Date_              20220226
Time                   0.54
INSTRUM               spect
PROBHD    5 mm PABBO BB/
PULPROG                zg30
TD                    32768
SOLVENT               CDCl3
NS                       16
DS                        0
SWH               8012.820 Hz
FIDRES            0.244532 Hz
AQ               2.0447233 sec
RG                   102.73
DW                   62.400 usec
DE                    6.50 usec
TE                   298.5 K
D1             2.00000000 sec
D11       0 sec
TD0                        1

======== CHANNEL f1 ========
SFO1           400.2424716 MHz
NUC1                      1H
P1                   14.30 usec
PLW1           12.00000000 W

======== CHANNEL f2 ========
SFO2           400.2424716 MHz
NUC2                     off
CPDPRG[2
PCPD2      0 usec
PLW2       0 W
PLW12      0 W
PLW13      0 W

F2 - Processing parameters
SI                    65536
SF           400.2399967 MHz
WDW                      EM
SSB        0
LB                   0.30 Hz
GB         0
PC                   1.00
```

Supplementary Figure 59. ^{1}H-NMR of compound **32**, recorded at 400 MHz and 25 °C in $CDCl_3$.

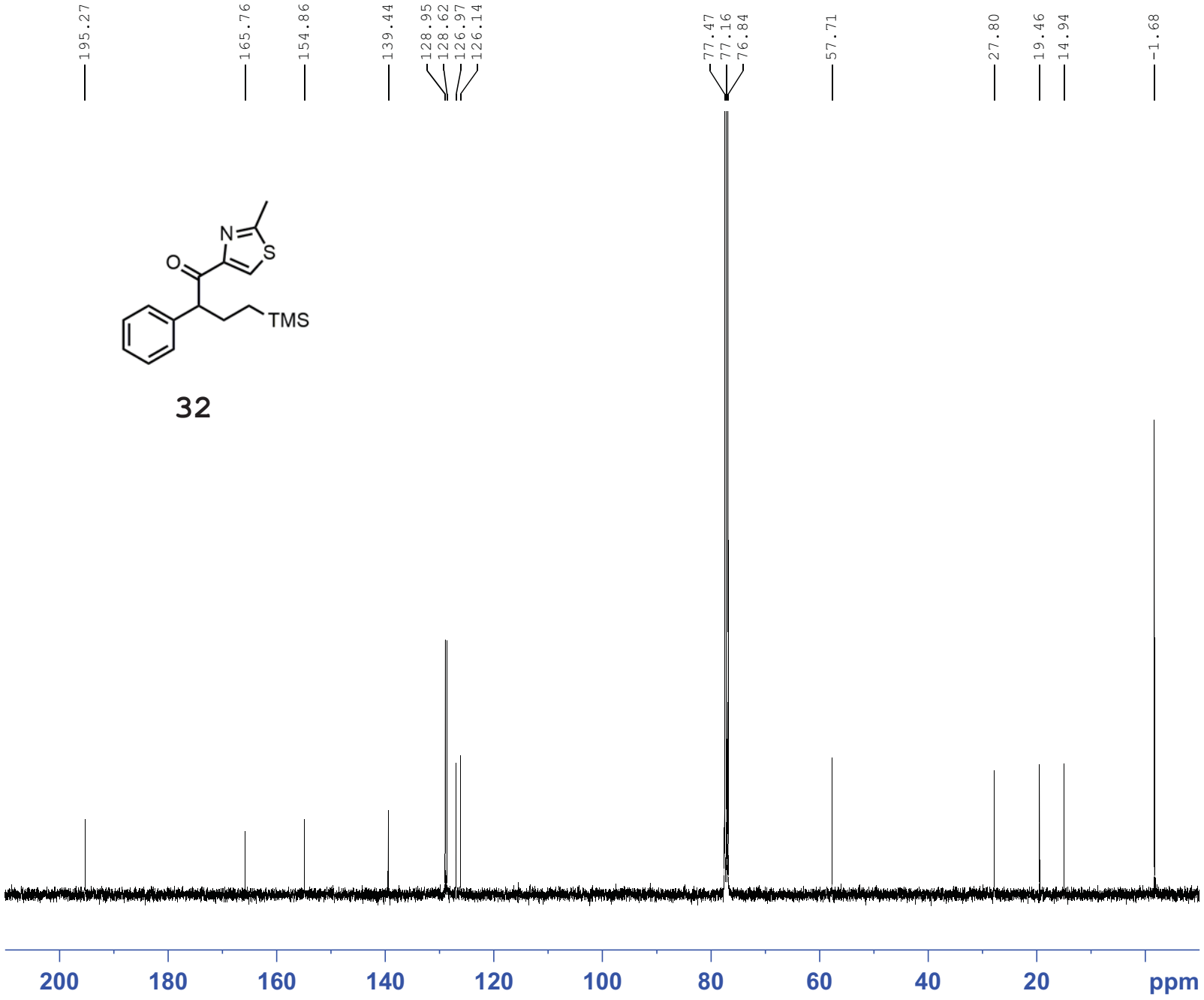


```
Current Data Parameters
NAME                 11148G
EXPNO                     2
PROCNO                    1

F2 - Acquisition Parameters
Date_              20220226
Time                  1.23
INSTRUM              spect
PROBHD   5 mm PABBO BB/
PULPROG             zgpg30
TD                   65536
SOLVENT              CDCl3
NS                     500
DS                       4
SWH              24038.461 Hz
FIDRES            0.366798 Hz
AQ                1.3631488 sec
RG                   206.33
DW                  20.800 usec
DE                   6.50 usec
TE                  299.3 K
D1             2.00000000 sec
D11            0.03000000 sec
TD0                       1

======== CHANNEL f1 ========
SFO1          100.6504916 MHz
NUC1                    13C
P1                   10.00 usec
PLW1          54.00000000 W

======== CHANNEL f2 ========
SFO2          400.2416010 MHz
NUC2                    1H
CPDPRG[2           waltz16
PCPD2                90.00 usec
PLW2          12.00000000 W
PLW12          0.30294999 W
PLW13          0.24539000 W

F2 - Processing parameters
SI                   32768
SF            100.6404154 MHz
WDW                     EM
SSB       0
LB                    1.00 Hz
GB        0
PC                    1.40
```

Supplementary Figure 60. ^{13}C-NMR of compound **32**, recorded at 101 MHz and 25 °C in $CDCl_3$.

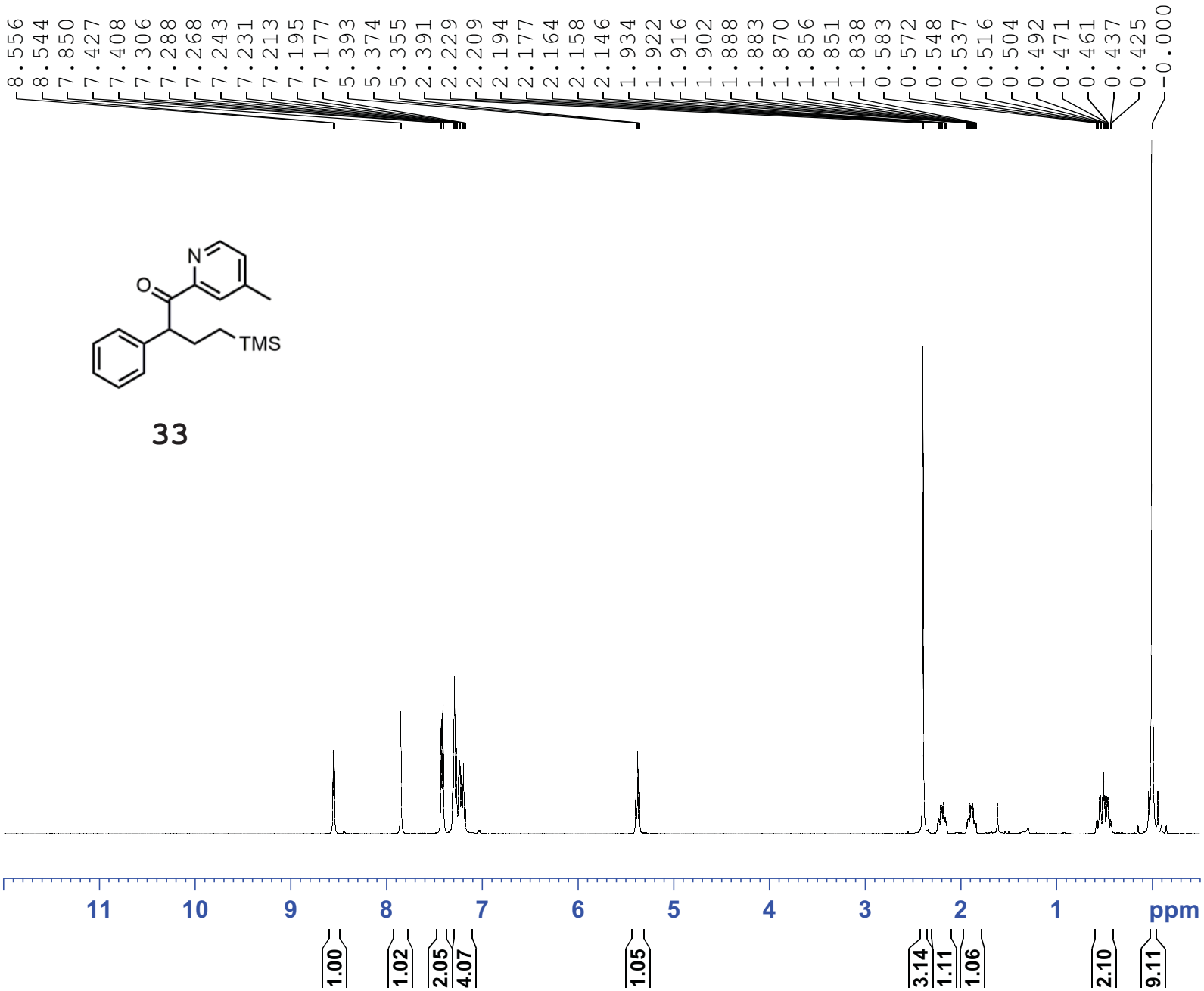


```
Current Data Parameters
NAME                  11148F
EXPNO                      1
PROCNO                     1

F2 - Acquisition Parameters
Date_              20220226
Time                   1.27
INSTRUM               spect
PROBHD    5 mm PABBO BB/
PULPROG                zg30
TD                    32768
SOLVENT               CDCl3
NS                       16
DS                        0
SWH                8012.820 Hz
FIDRES             0.244532 Hz
AQ                2.0447233 sec
RG                   206.33
DW                   62.400 usec
DE                    6.50 usec
TE                   298.5 K
D1              2.00000000 sec
D11       0 sec
TD0                       1

======== CHANNEL f1 ========
SFO1           400.2424716 MHz
NUC1                     1H
P1                   14.30 usec
PLW1           12.00000000 W

======== CHANNEL f2 ========
SFO2           400.2424716 MHz
NUC2                    off
CPDPRG[2
PCPD2      0 usec
PLW2       0 W
PLW12      0 W
PLW13      0 W

F2 - Processing parameters
SI                     65536
SF             400.2399968 MHz
WDW                       EM
SSB        0
LB                    0.30 Hz
GB         0
PC                    1.00
```

Supplementary Figure 61. ^{1}H-NMR of compound **33**, recorded at 400 MHz and 25 °C in $CDCl_3$.

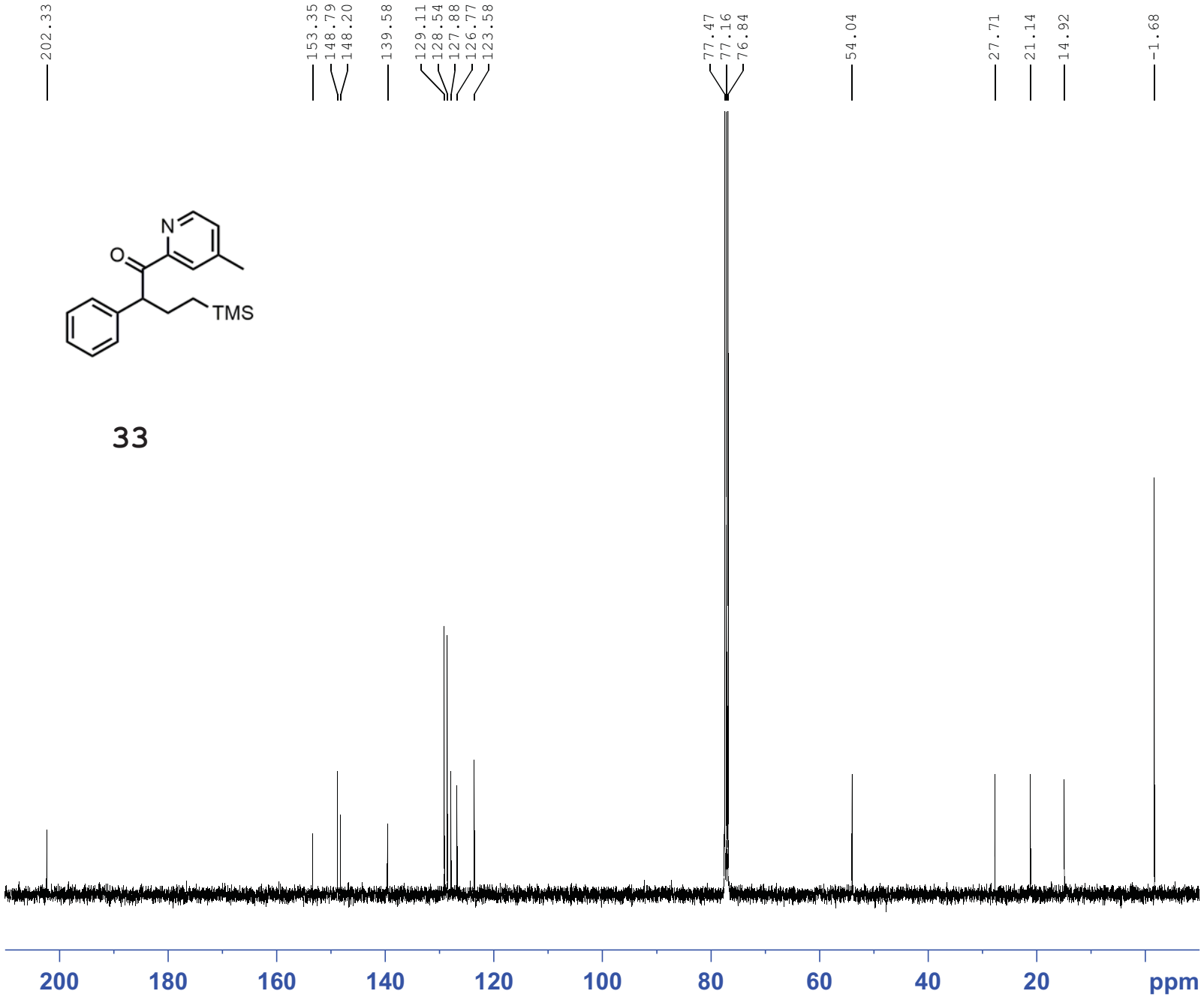


```
Current Data Parameters
NAME                  11148F
EXPNO                      2
PROCNO                     1

F2 - Acquisition Parameters
Date_              20220226
Time                   1.56
INSTRUM               spect
PROBHD   5 mm PABBO BB/
PULPROG              zgpg30
TD                    65536
SOLVENT               CDCl3
NS                      500
DS                        4
SWH              24038.461 Hz
FIDRES            0.366798 Hz
AQ               1.3631488 sec
RG                   206.33
DW                   20.800 usec
DE                    6.50 usec
TE                   299.3 K
D1              2.00000000 sec
D11             0.03000000 sec
TD0                       1

======== CHANNEL f1 ========
SFO1           100.6504916 MHz
NUC1                    13C
P1                   10.00 usec
PLW1           54.00000000 W

======== CHANNEL f2 ========
SFO2           400.2416010 MHz
NUC2                    1H
CPDPRG[2           waltz16
PCPD2                90.00 usec
PLW2           12.00000000 W
PLW12           0.30294999 W
PLW13           0.24539000 W

F2 - Processing parameters
SI                      32768
SF             100.6404147 MHz
WDW                        EM
SSB          0
LB                    1.00 Hz
GB           0
PC                    1.40
```

Supplementary Figure 62. ^{13}C-NMR of compound **33**, recorded at 101 MHz and 25 °C in $CDCl_3$.

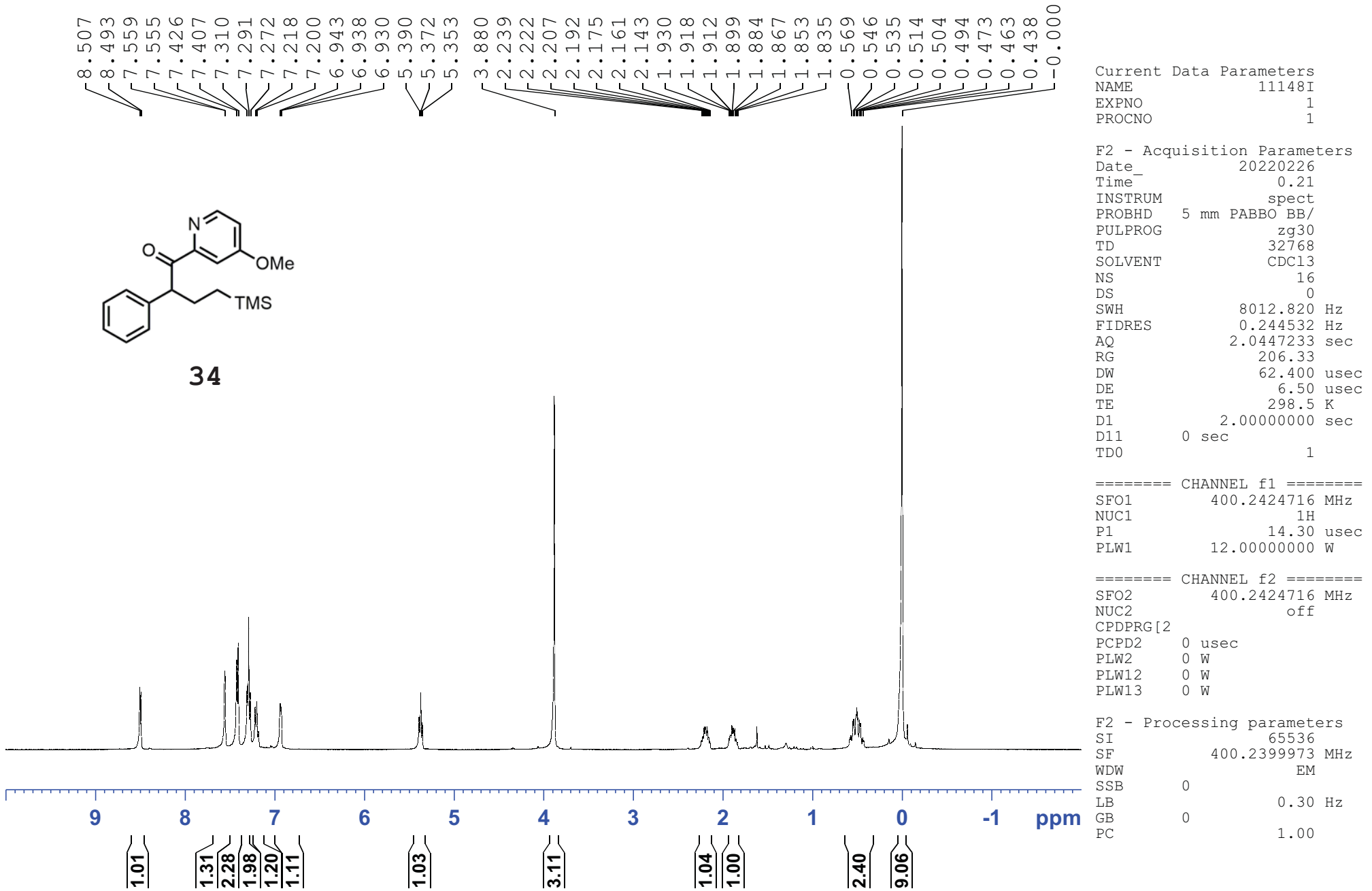


```
Current Data Parameters
NAME                  11148I
EXPNO                      1
PROCNO                     1

F2 - Acquisition Parameters
Date_              20220226
Time                   0.21
INSTRUM               spect
PROBHD    5 mm PABBO BB/
PULPROG                zg30
TD                    32768
SOLVENT               CDCl3
NS                       16
DS                        0
SWH                8012.820 Hz
FIDRES             0.244532 Hz
AQ                2.0447233 sec
RG                   206.33
DW                   62.400 usec
DE                    6.50 usec
TE                   298.5 K
D1              2.00000000 sec
D11       0 sec
TD0                        1

======== CHANNEL f1 ========
SFO1           400.2424716 MHz
NUC1                      1H
P1                    14.30 usec
PLW1           12.00000000 W

======== CHANNEL f2 ========
SFO2           400.2424716 MHz
NUC2                     off
CPDPRG[2
PCPD2      0 usec
PLW2       0 W
PLW12      0 W
PLW13      0 W

F2 - Processing parameters
SI                     65536
SF             400.2399973 MHz
WDW                       EM
SSB        0
LB                    0.30 Hz
GB         0
PC                    1.00
```

Supplementary Figure 63. ^{1}H-NMR of compound **34**, recorded at 400 MHz and 25 °C in $CDCl_3$.

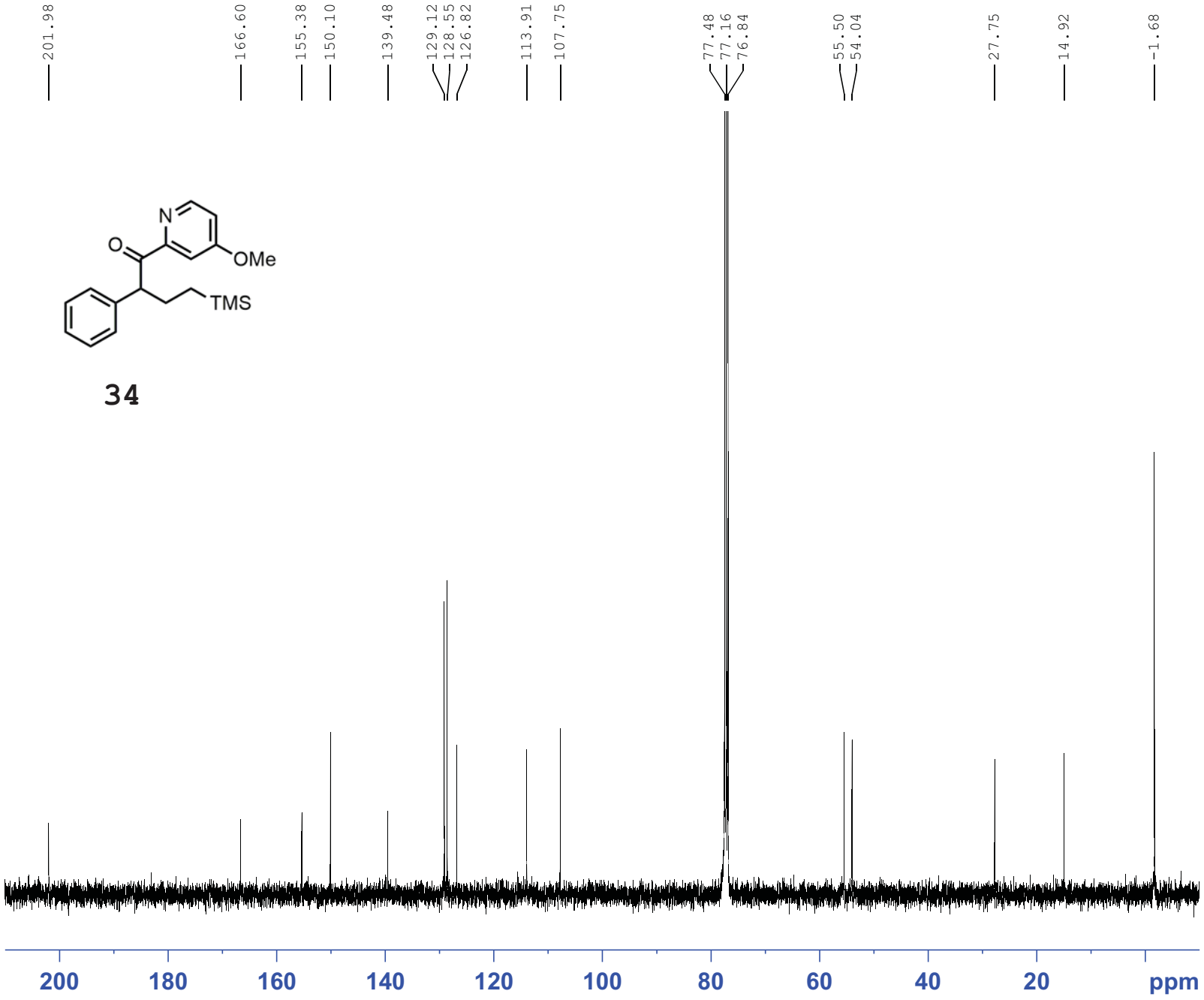


```
Current Data Parameters
NAME                  11148I
EXPNO                      2
PROCNO                     1

F2 - Acquisition Parameters
Date_              20220226
Time                   0.50
INSTRUM               spect
PROBHD   5 mm PABBO BB/
PULPROG              zgpg30
TD                    65536
SOLVENT               CDCl3
NS                      500
DS                        4
SWH             24038.461 Hz
FIDRES           0.366798 Hz
AQ               1.3631488 sec
RG                  206.33
DW                  20.800 usec
DE                   6.50 usec
TE                  299.3 K
D1             2.00000000 sec
D11            0.03000000 sec
TD0                       1

======== CHANNEL f1 ========
SFO1           100.6504916 MHz
NUC1                    13C
P1                   10.00 usec
PLW1           54.00000000 W

======== CHANNEL f2 ========
SFO2           400.2416010 MHz
NUC2                    1H
CPDPRG[2           waltz16
PCPD2                90.00 usec
PLW2           12.00000000 W
PLW12           0.30294999 W
PLW13           0.24539000 W

F2 - Processing parameters
SI                     32768
SF             100.6404146 MHz
WDW                       EM
SSB          0
LB                    1.00 Hz
GB           0
PC                    1.40
```

Supplementary Figure 64. ^{13}C-NMR of compound **34**, recorded at 101 MHz and 25 °C in $CDCl_3$.

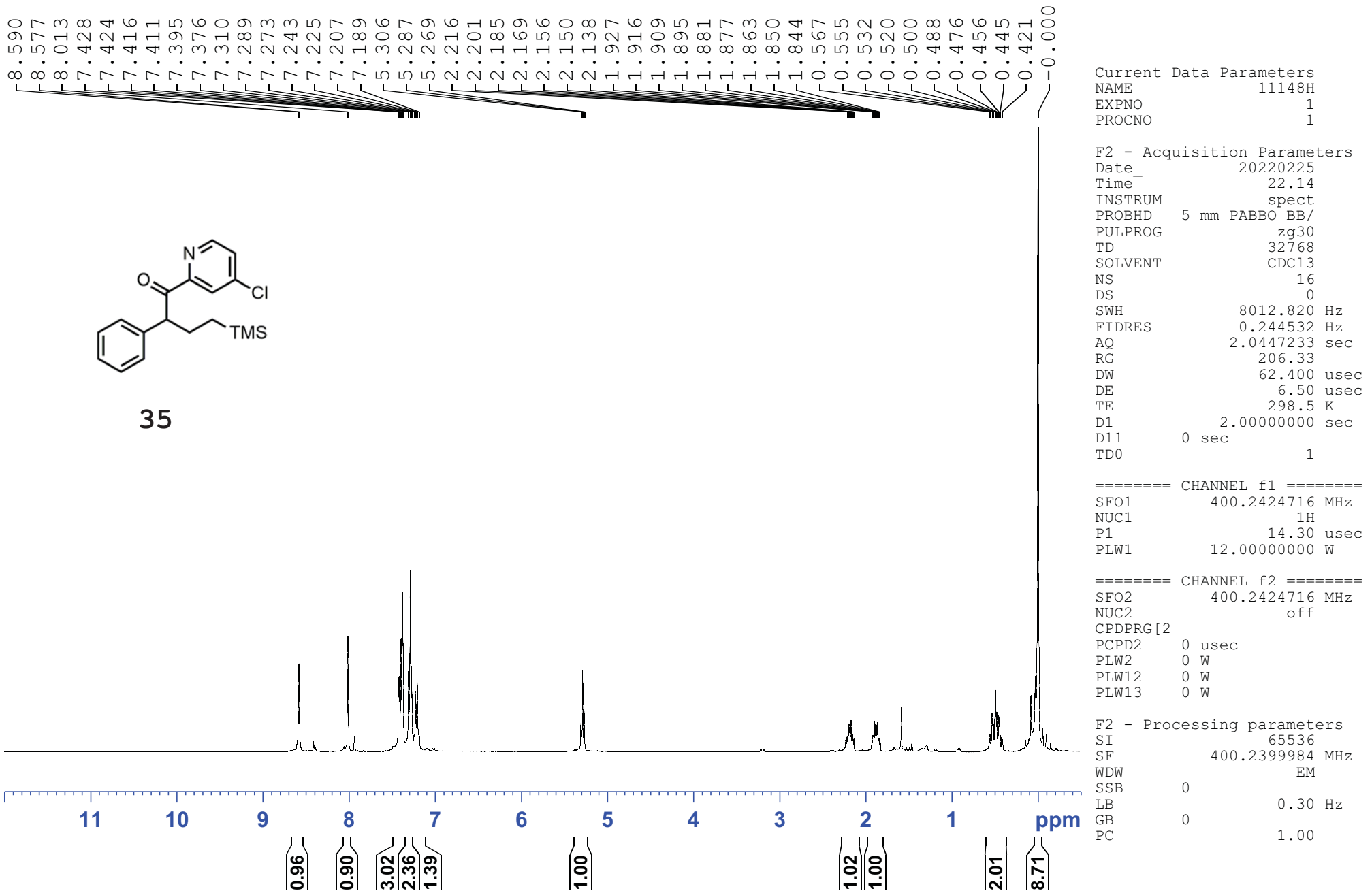


```
Current Data Parameters
NAME                  11148H
EXPNO                      1
PROCNO                     1

F2 - Acquisition Parameters
Date_              20220225
Time                  22.14
INSTRUM               spect
PROBHD    5 mm PABBO BB/
PULPROG                zg30
TD                    32768
SOLVENT               CDCl3
NS                       16
DS                        0
SWH                8012.820 Hz
FIDRES             0.244532 Hz
AQ                2.0447233 sec
RG                   206.33
DW                   62.400 usec
DE                    6.50 usec
TE                   298.5 K
D1              2.00000000 sec
D11       0 sec
TD0                       1

======== CHANNEL f1 ========
SFO1           400.2424716 MHz
NUC1                      1H
P1                    14.30 usec
PLW1           12.00000000 W

======== CHANNEL f2 ========
SFO2           400.2424716 MHz
NUC2                    off
CPDPRG[2
PCPD2      0 usec
PLW2       0 W
PLW12      0 W
PLW13      0 W

F2 - Processing parameters
SI                     65536
SF             400.2399984 MHz
WDW                       EM
SSB        0
LB                    0.30 Hz
GB         0
PC                    1.00
```

Supplementary Figure 65. ^{1}H-NMR of compound **35**, recorded at 400 MHz and 25 °C in $CDCl_3$.

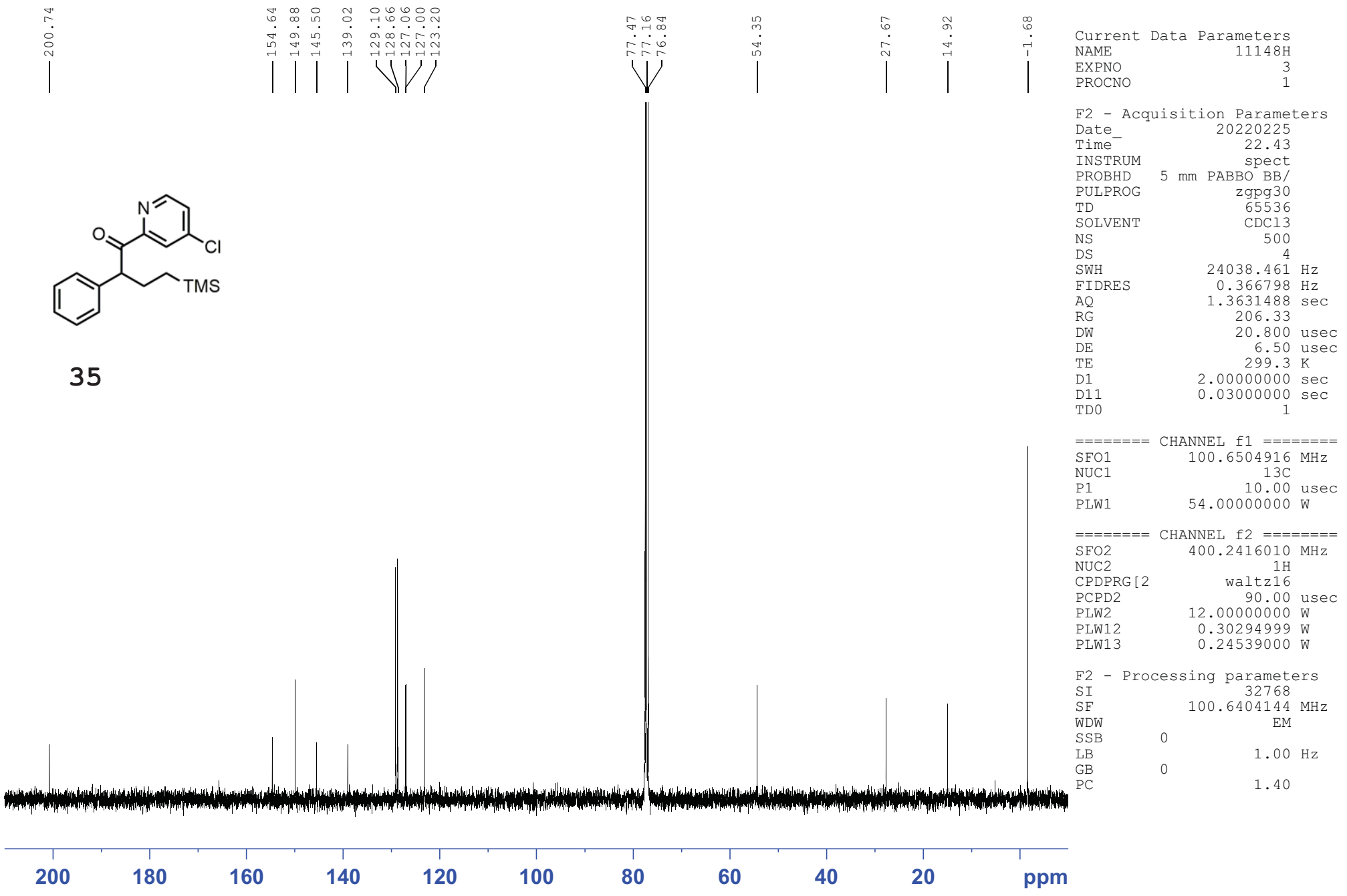

Supplementary Figure 66. ^{13}C-NMR of compound **35**, recorded at 101 MHz and 25 °C in $CDCl_3$.

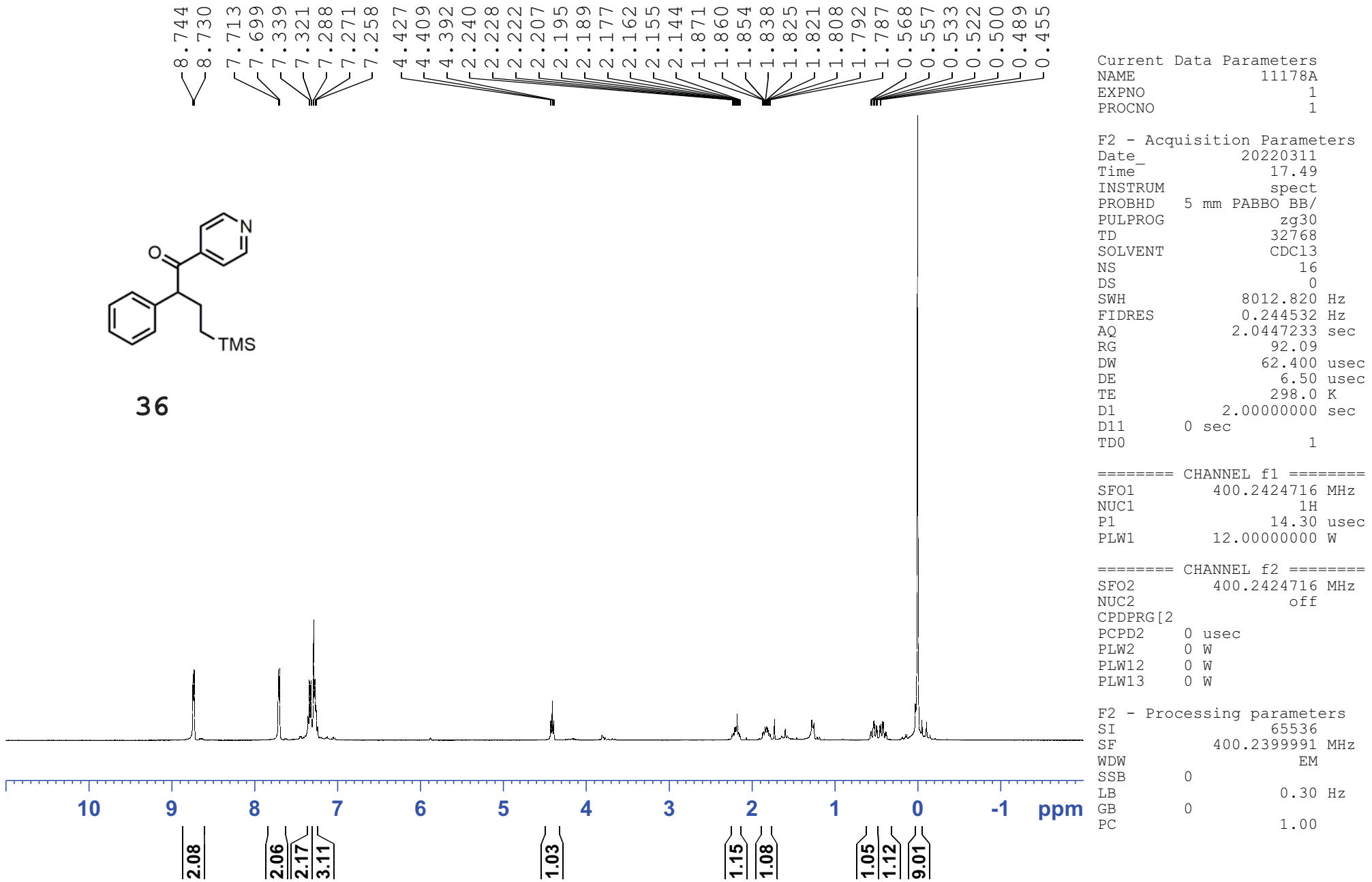


```
Current Data Parameters
NAME                  11178A
EXPNO                      1
PROCNO                     1

F2 - Acquisition Parameters
Date_              20220311
Time                  17.49
INSTRUM               spect
PROBHD    5 mm PABBO BB/
PULPROG                zg30
TD                    32768
SOLVENT               CDCl3
NS                       16
DS                        0
SWH               8012.820 Hz
FIDRES             0.244532 Hz
AQ                2.0447233 sec
RG                    92.09
DW                  62.400 usec
DE                   6.50 usec
TE                  298.0 K
D1              2.00000000 sec
D11       0 sec
TD0                       1

======== CHANNEL f1 ========
SFO1           400.2424716 MHz
NUC1                      1H
P1                    14.30 usec
PLW1           12.00000000 W

======== CHANNEL f2 ========
SFO2           400.2424716 MHz
NUC2                     off
CPDPRG[2
PCPD2      0 usec
PLW2       0 W
PLW12      0 W
PLW13      0 W

F2 - Processing parameters
SI                     65536
SF             400.2399991 MHz
WDW                       EM
SSB        0
LB                    0.30 Hz
GB         0
PC                    1.00
```

Supplementary Figure 67. ^{1}H-NMR of compound **36**, recorded at 400 MHz and 25 °C in $CDCl_3$.

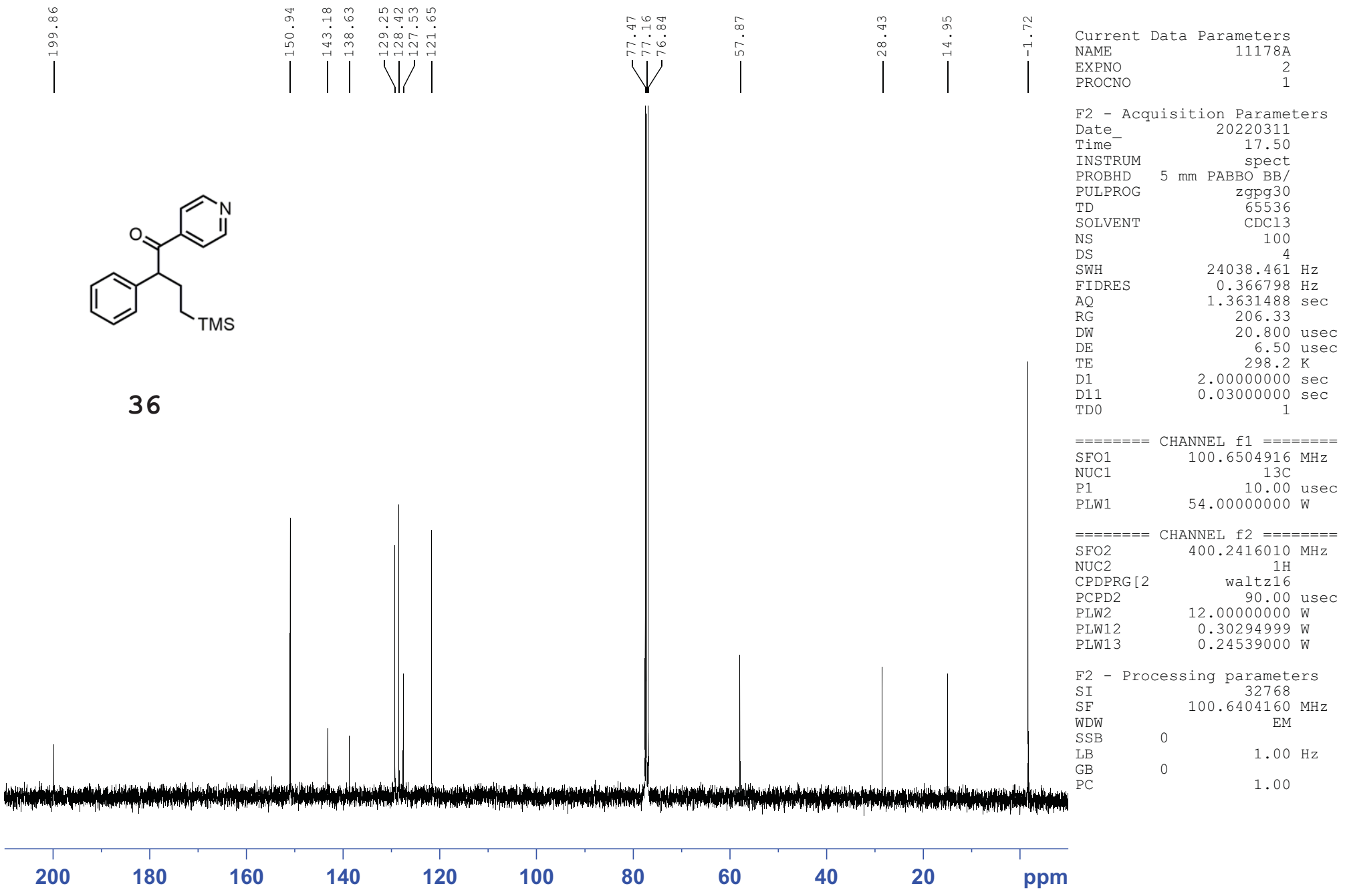

Supplementary Figure 68. ^{13}C-NMR of compound **36**, recorded at 101 MHz and 25 °C in $CDCl_3$.

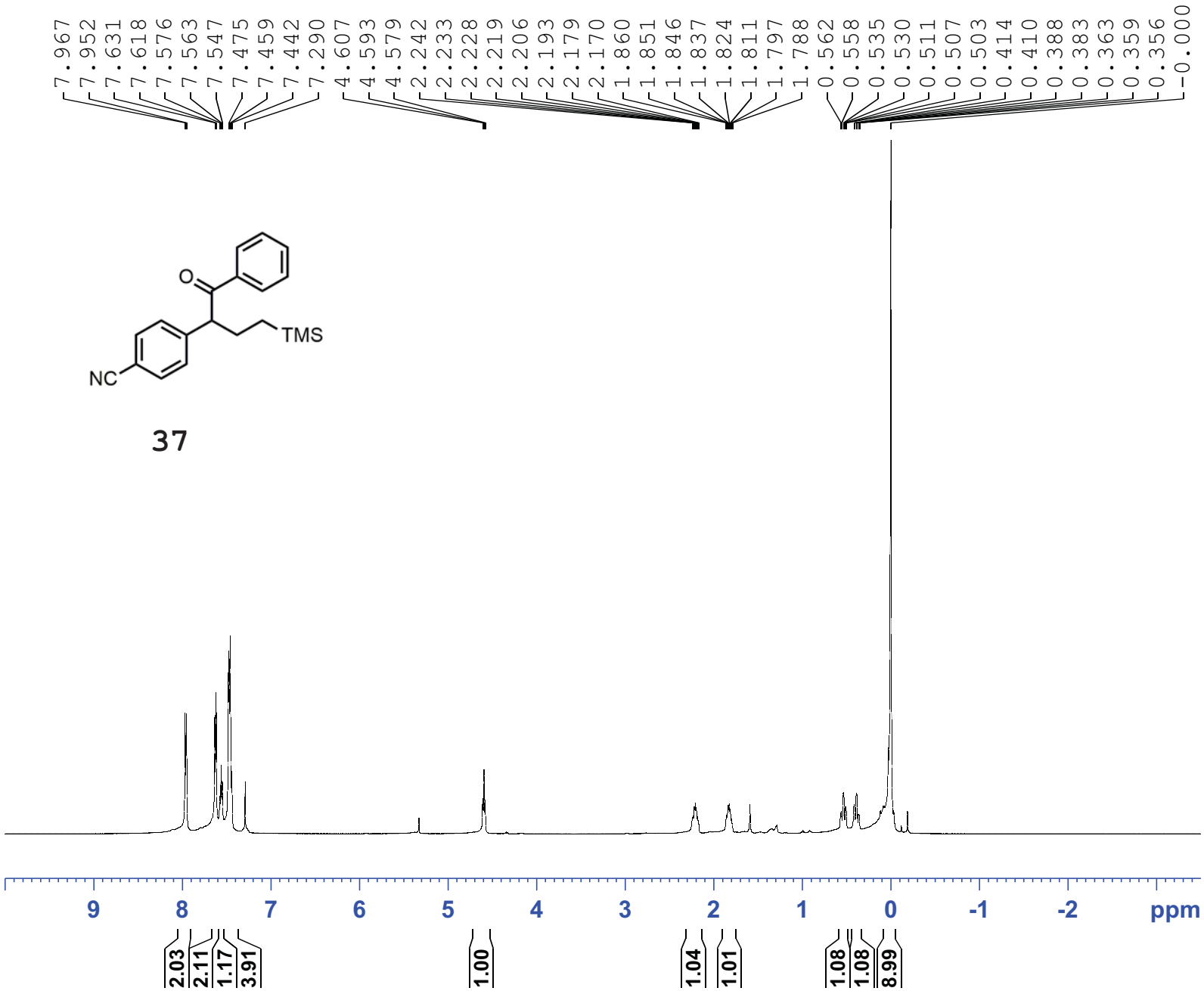


```
Current Data Parameters
NAME                  11157C
EXPNO                      1
PROCNO                     1

F2 - Acquisition Parameters
Date_              20220304
Time                  15.44
INSTRUM               spect
PROBHD   5 mm CPPBBO BB
PULPROG                zg30
TD                    65536
SOLVENT               CDCl3
NS                       16
DS                        2
SWH              10000.000 Hz
FIDRES            0.152588 Hz
AQ               3.2767999 sec
RG                    55.37
DW                  50.000 usec
DE                   6.50 usec
TE                   298.2 K
D1              1.00000000 sec
D11       0 sec
TD0                       1

======== CHANNEL f1 ========
SFO1           500.1330885 MHz
NUC1                   1H
P1                  11.25 usec
PLW1           20.00000000 W

======== CHANNEL f2 ========
SFO2           500.1330885 MHz
NUC2                  off
CPDPRG[2
PCPD2      0 usec
PLW2       0 W
PLW12      0 W
PLW13      0 W

F2 - Processing parameters
SI                    65536
SF           500.1299984 MHz
WDW                     EM
SSB        0
LB                  0.30 Hz
GB         0
PC                  1.00
```

Supplementary Figure 69. ^{1}H-NMR of compound **37**, recorded at 500 MHz and 25 °C in $CDCl_3$.

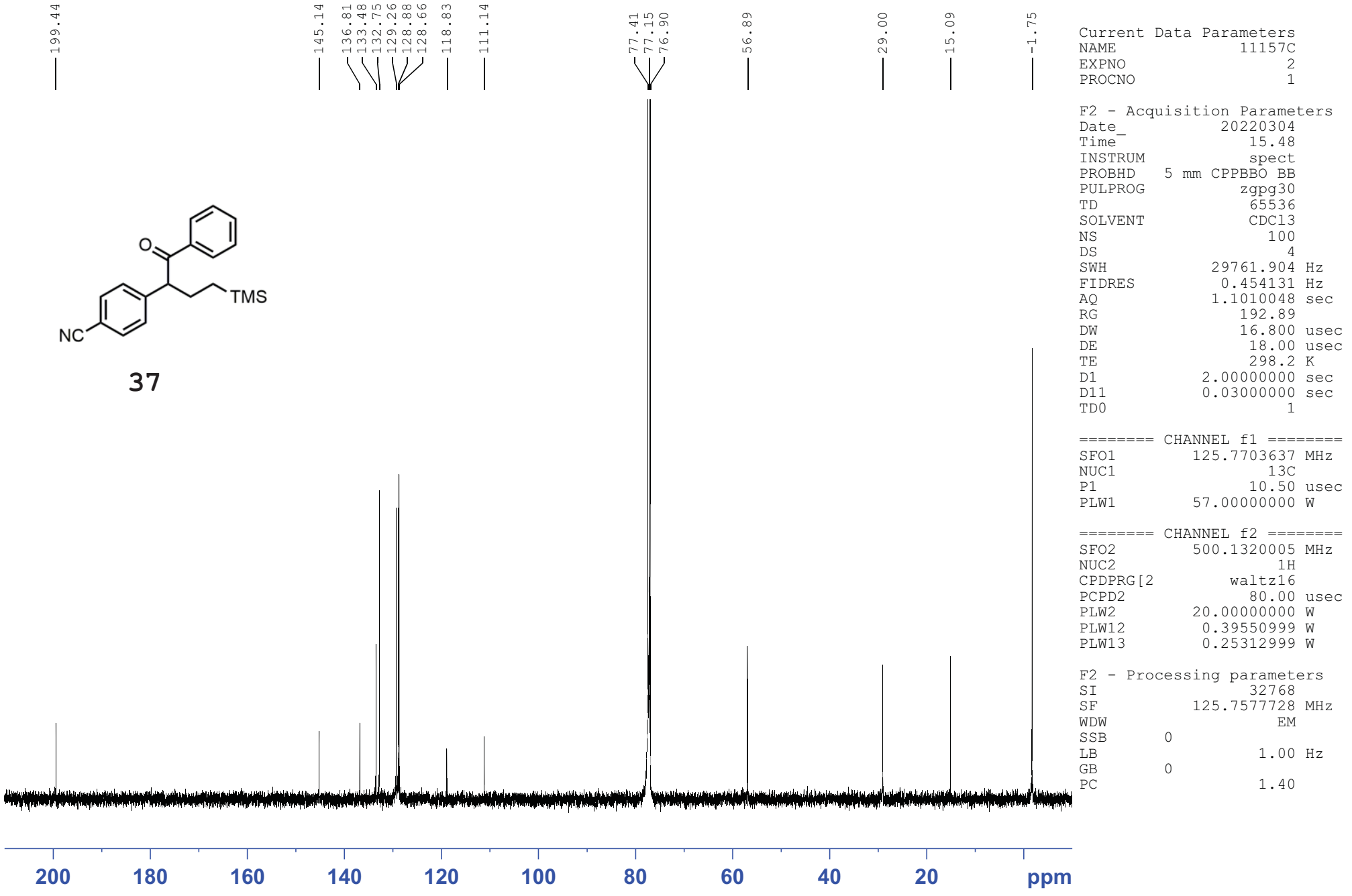

Supplementary Figure 70. ^{13}C-NMR of compound **37**, recorded at 126 MHz and 25 °C in $CDCl_3$.

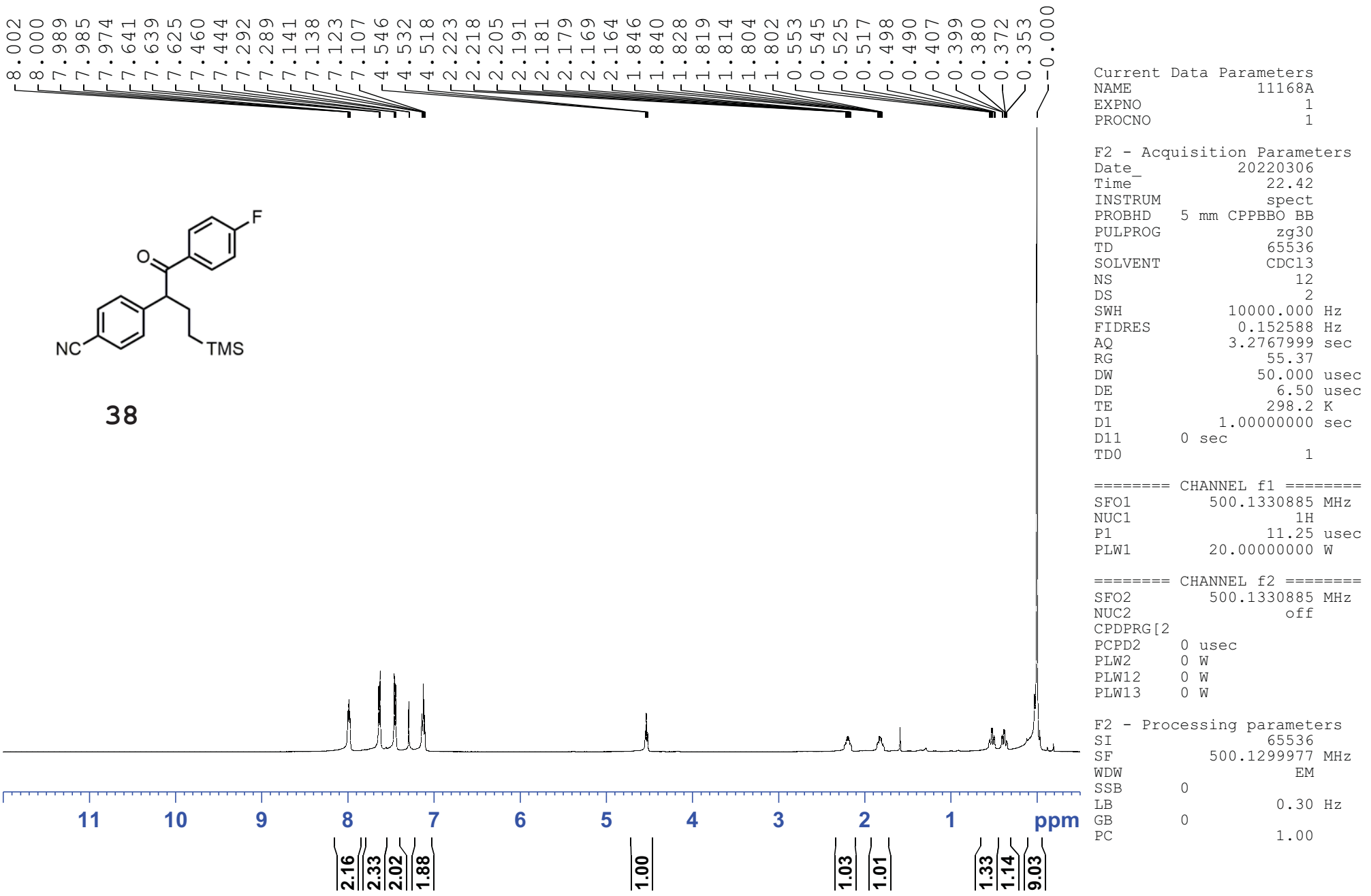


```
Current Data Parameters
NAME                  11168A
EXPNO                      1
PROCNO                     1

F2 - Acquisition Parameters
Date_              20220306
Time                  22.42
INSTRUM               spect
PROBHD    5 mm CPPBBO BB
PULPROG                zg30
TD                    65536
SOLVENT               CDCl3
NS                       12
DS                        2
SWH              10000.000 Hz
FIDRES               0.152588 Hz
AQ                3.2767999 sec
RG                    55.37
DW                   50.000 usec
DE                    6.50 usec
TE                   298.2 K
D1             1.00000000 sec
D11        0 sec
TD0                       1

======== CHANNEL f1 ========
SFO1           500.1330885 MHz
NUC1                      1H
P1                    11.25 usec
PLW1           20.00000000 W

======== CHANNEL f2 ========
SFO2           500.1330885 MHz
NUC2                    off
CPDPRG[2
PCPD2      0 usec
PLW2       0 W
PLW12      0 W
PLW13      0 W

F2 - Processing parameters
SI                        65536
SF             500.1299977 MHz
WDW                       EM
SSB        0
LB                    0.30 Hz
GB         0
PC                    1.00
```

Supplementary Figure 71. ^{1}H-NMR of compound **38**, recorded at 500 MHz and 25 °C in $CDCl_3$.

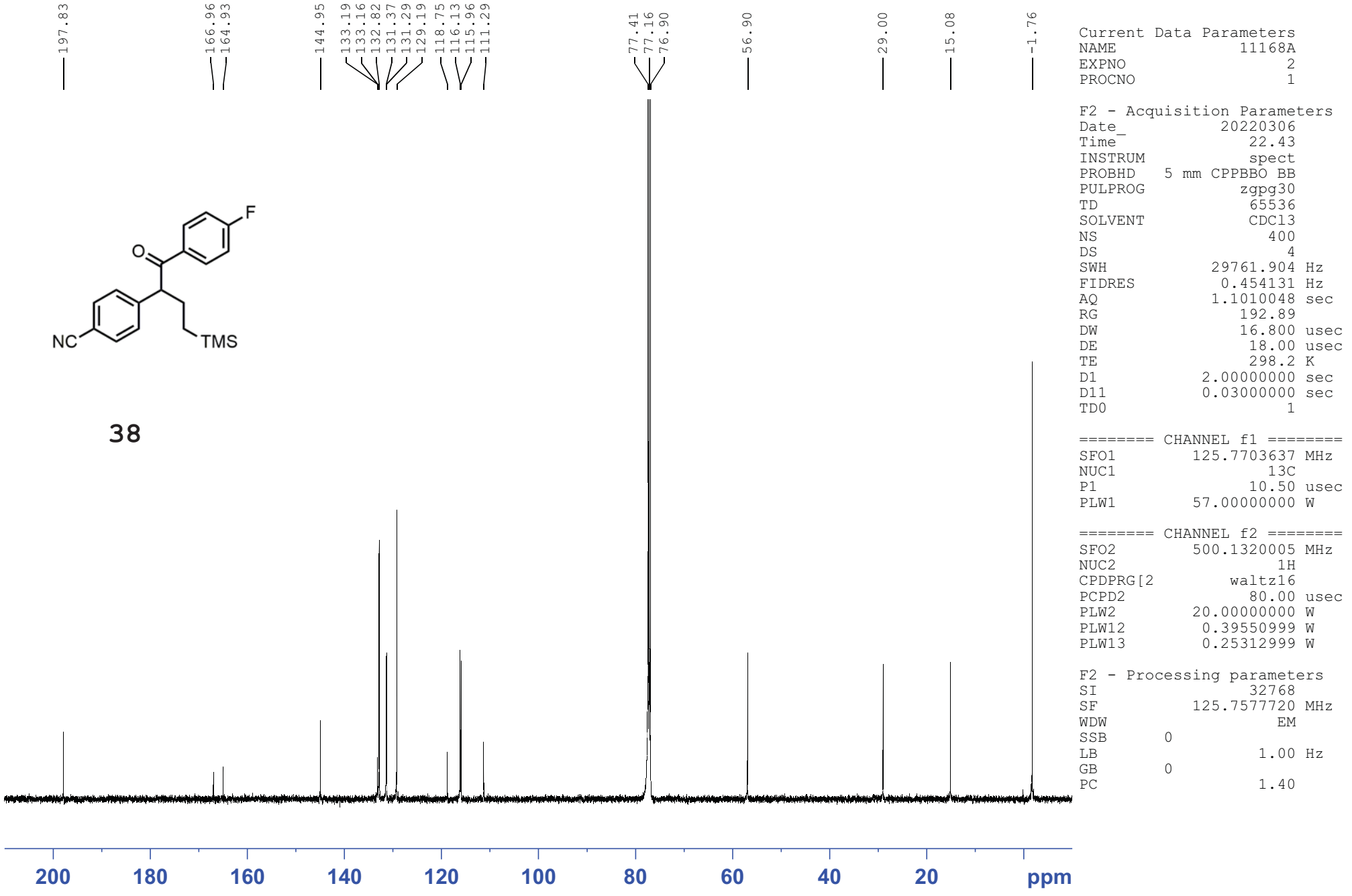

Supplementary Figure 72. ^{13}C-NMR of compound **39**, recorded at 126 MHz and 25 °C in $CDCl_3$.

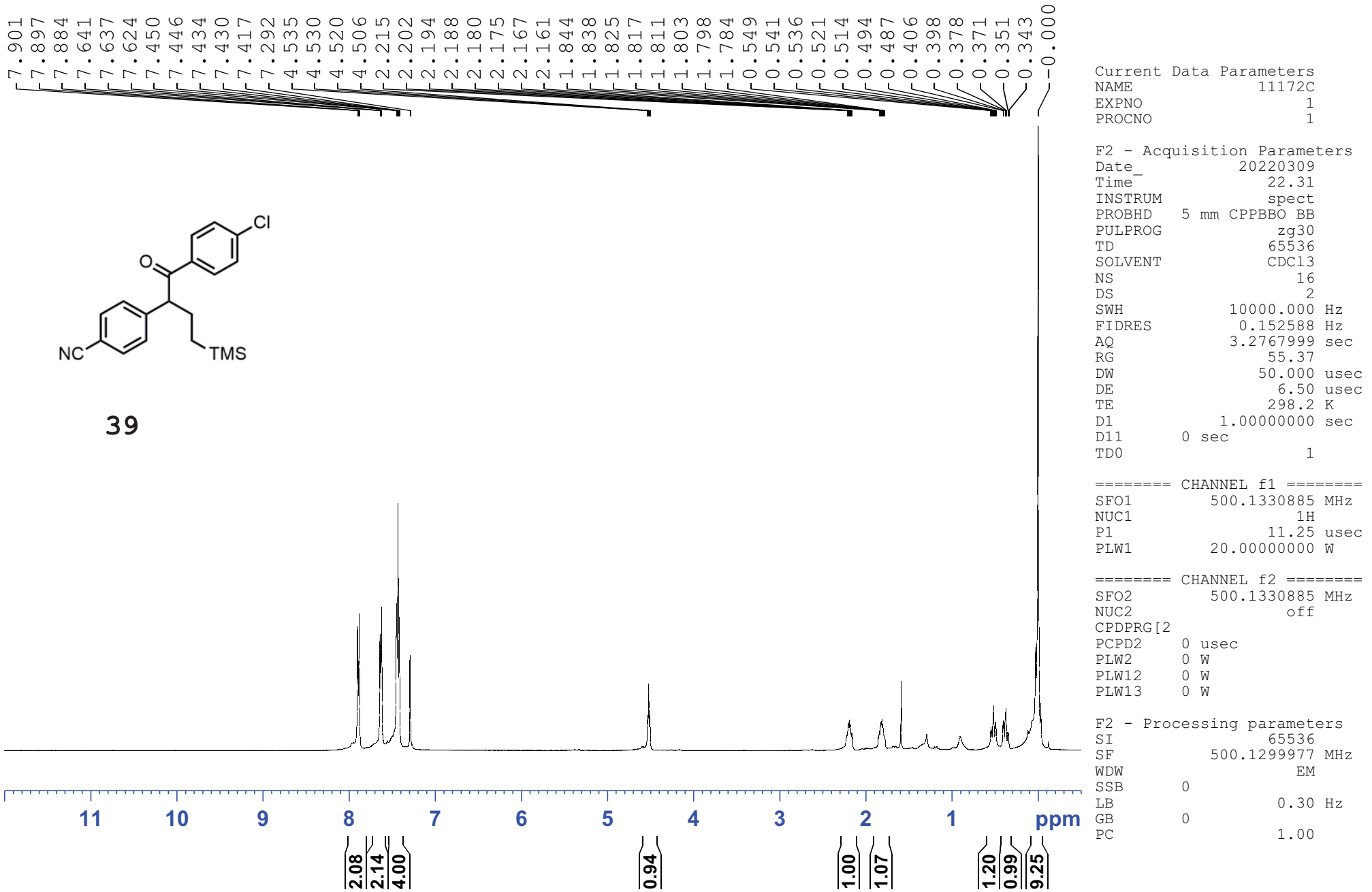


```
Current Data Parameters
NAME                  11172C
EXPNO                      1
PROCNO                     1

F2 - Acquisition Parameters
Date_              20220309
Time                 22.31
INSTRUM              spect
PROBHD    5 mm CPPBBO BB
PULPROG               zg30
TD                   65536
SOLVENT              CDCl3
NS                      16
DS                       2
SWH             10000.000 Hz
FIDRES              0.152588 Hz
AQ                3.2767999 sec
RG                    55.37
DW                  50.000 usec
DE                   6.50 usec
TE                  298.2 K
D1              1.00000000 sec
D11       0 sec
TD0                        1

======== CHANNEL f1 ========
SFO1           500.1330885 MHz
NUC1                    1H
P1                  11.25 usec
PLW1           20.00000000 W

======== CHANNEL f2 ========
SFO2           500.1330885 MHz
NUC2                   off
CPDPRG[2
PCPD2      0 usec
PLW2       0 W
PLW12      0 W
PLW13      0 W

F2 - Processing parameters
SI                     65536
SF             500.1299977 MHz
WDW                      EM
SSB        0
LB                   0.30 Hz
GB         0
PC                   1.00
```

Supplementary Figure 73. ^{1}H-NMR of compound **39**, recorded at 500 MHz and 25 °C in $CDCl_3$.

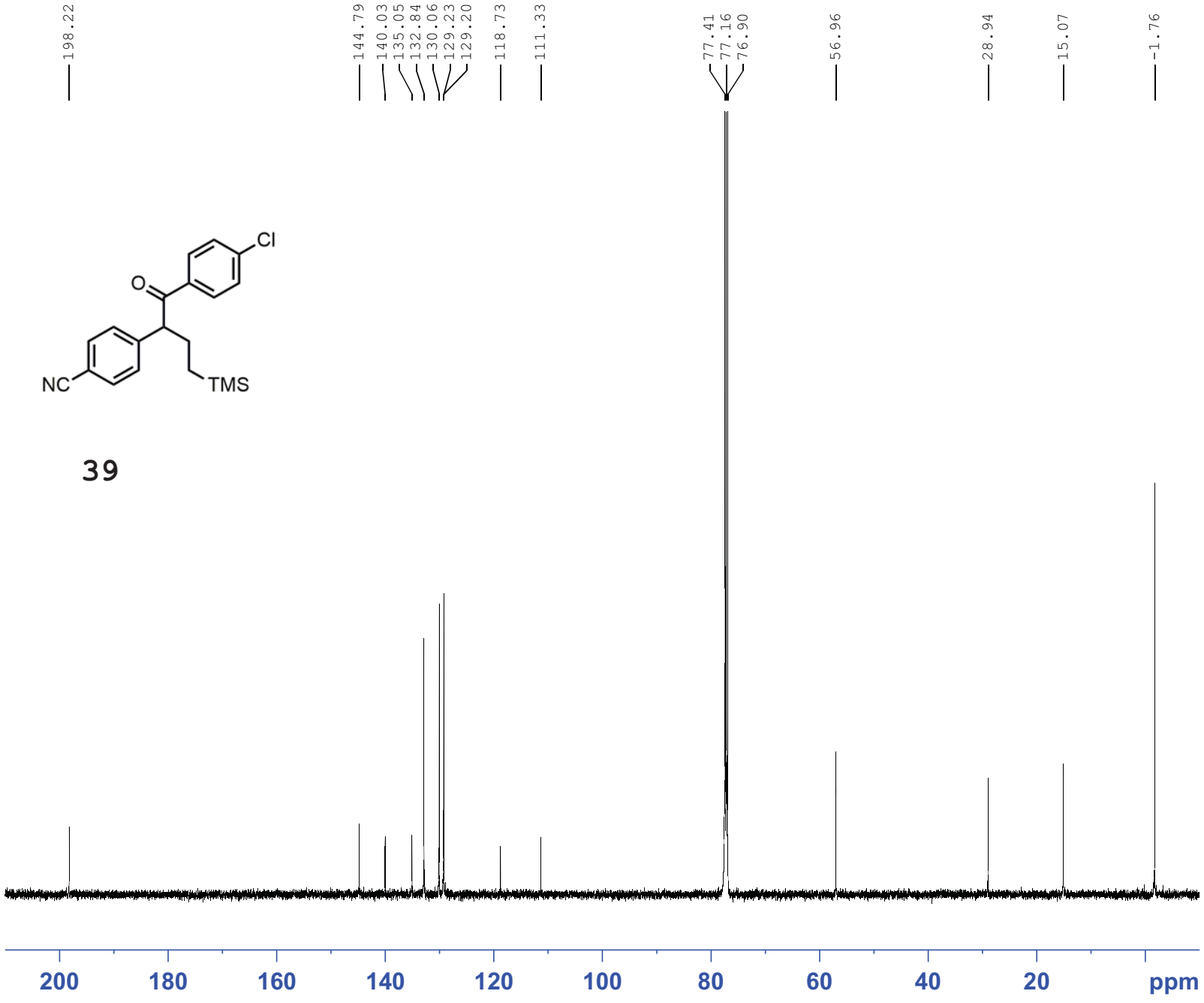


```
Current Data Parameters
NAME                 11172C
EXPNO                     2
PROCNO                    1

F2 - Acquisition Parameters
Date_              20220309
Time                 22.42
INSTRUM              spect
PROBHD   5 mm CPPBBO BB
PULPROG              zgpg30
TD                    65536
SOLVENT               CDCl3
NS                      200
DS                        4
SWH              29761.904 Hz
FIDRES            0.454131 Hz
AQ                1.1010048 sec
RG                   192.89
DW                   16.800 usec
DE                   18.00 usec
TE                   298.2 K
D1              2.00000000 sec
D11             0.03000000 sec
TD0                       1

======== CHANNEL f1 ========
SFO1           125.7703637 MHz
NUC1                    13C
P1                   10.50 usec
PLW1           57.00000000 W

======== CHANNEL f2 ========
SFO2           500.1320005 MHz
NUC2                    1H
CPDPRG[2            waltz16
PCPD2                80.00 usec
PLW2           20.00000000 W
PLW12           0.39550999 W
PLW13           0.25312999 W

F2 - Processing parameters
SI                    32768
SF             125.7577721 MHz
WDW                      EM
SSB          0
LB                    1.00 Hz
GB           0
PC                    1.40
```

Supplementary Figure 74. ^{13}C-NMR of compound **39**, recorded at 126 MHz and 25 °C in $CDCl_3$.

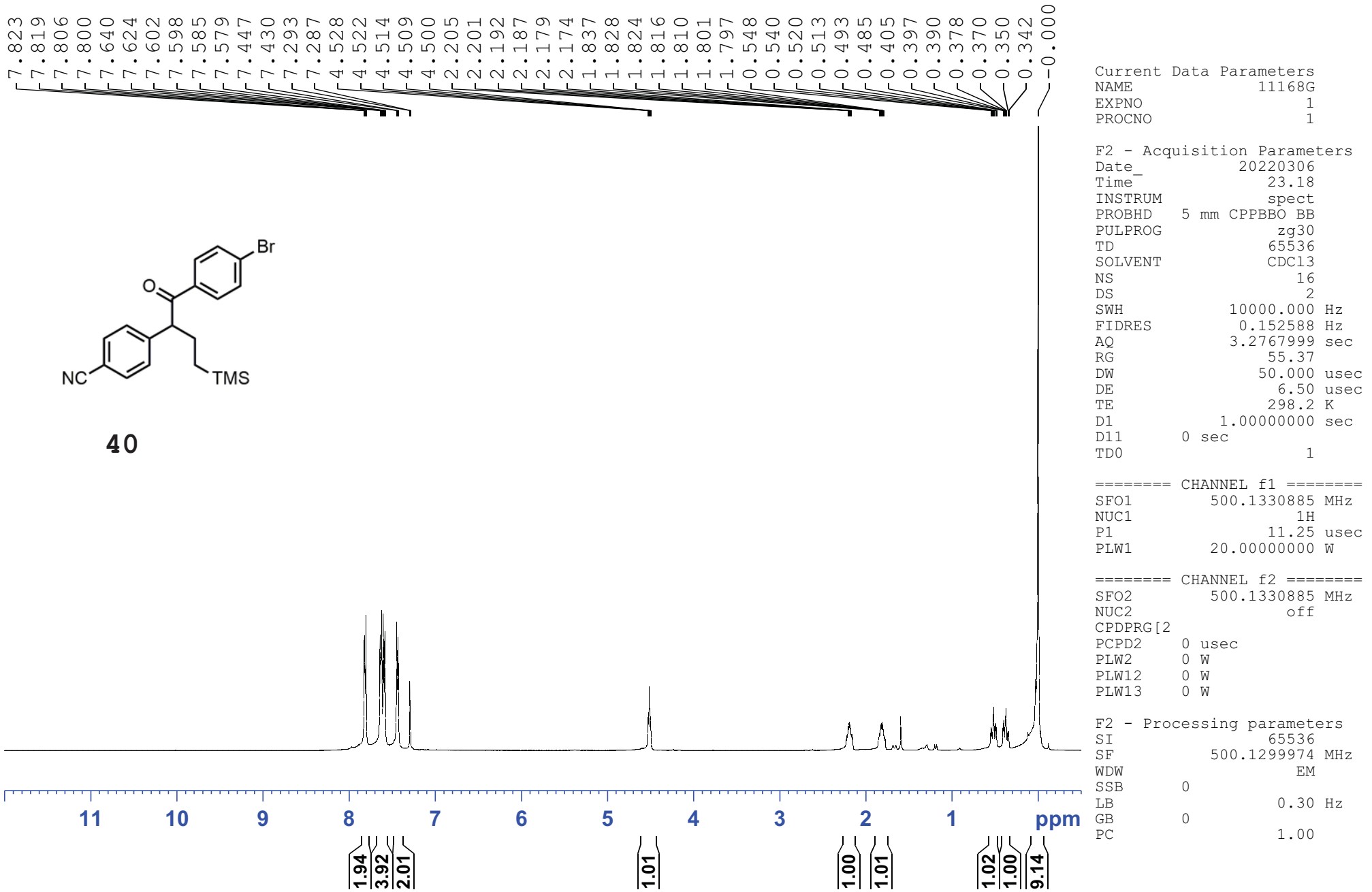


```
Current Data Parameters
NAME                  11168G
EXPNO                      1
PROCNO                     1

F2 - Acquisition Parameters
Date_              20220306
Time                  23.18
INSTRUM               spect
PROBHD    5 mm CPPBBO BB
PULPROG                zg30
TD                    65536
SOLVENT               CDCl3
NS                       16
DS                        2
SWH              10000.000 Hz
FIDRES            0.152588 Hz
AQ               3.2767999 sec
RG                    55.37
DW                   50.000 usec
DE                    6.50 usec
TE                   298.2 K
D1             1.00000000 sec
D11       0 sec
TD0                       1

======== CHANNEL f1 ========
SFO1           500.1330885 MHz
NUC1                     1H
P1                   11.25 usec
PLW1          20.00000000 W

======== CHANNEL f2 ========
SFO2           500.1330885 MHz
NUC2                   off
CPDPRG[2
PCPD2      0 usec
PLW2       0 W
PLW12      0 W
PLW13      0 W

F2 - Processing parameters
SI                    65536
SF           500.1299974 MHz
WDW                      EM
SSB        0
LB                  0.30 Hz
GB         0
PC                  1.00
```

Supplementary Figure 75. ^{1}H-NMR of compound **40**, recorded at 400 MHz and 25 °C in $CDCl_3$.

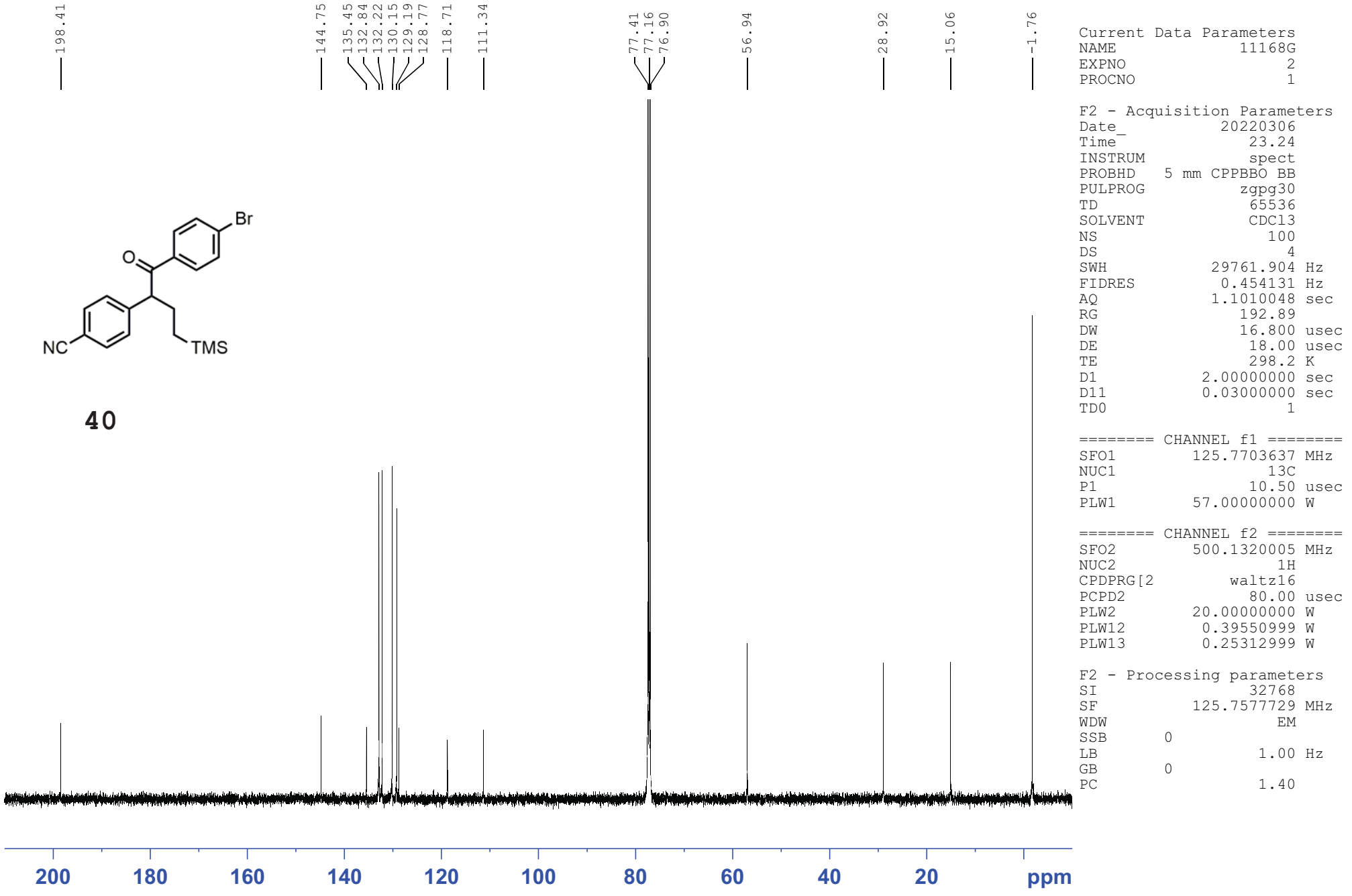

Supplementary Figure 76. ^{13}C-NMR of compound **40**, recorded at 126 MHz and 25 °C in $CDCl_3$.

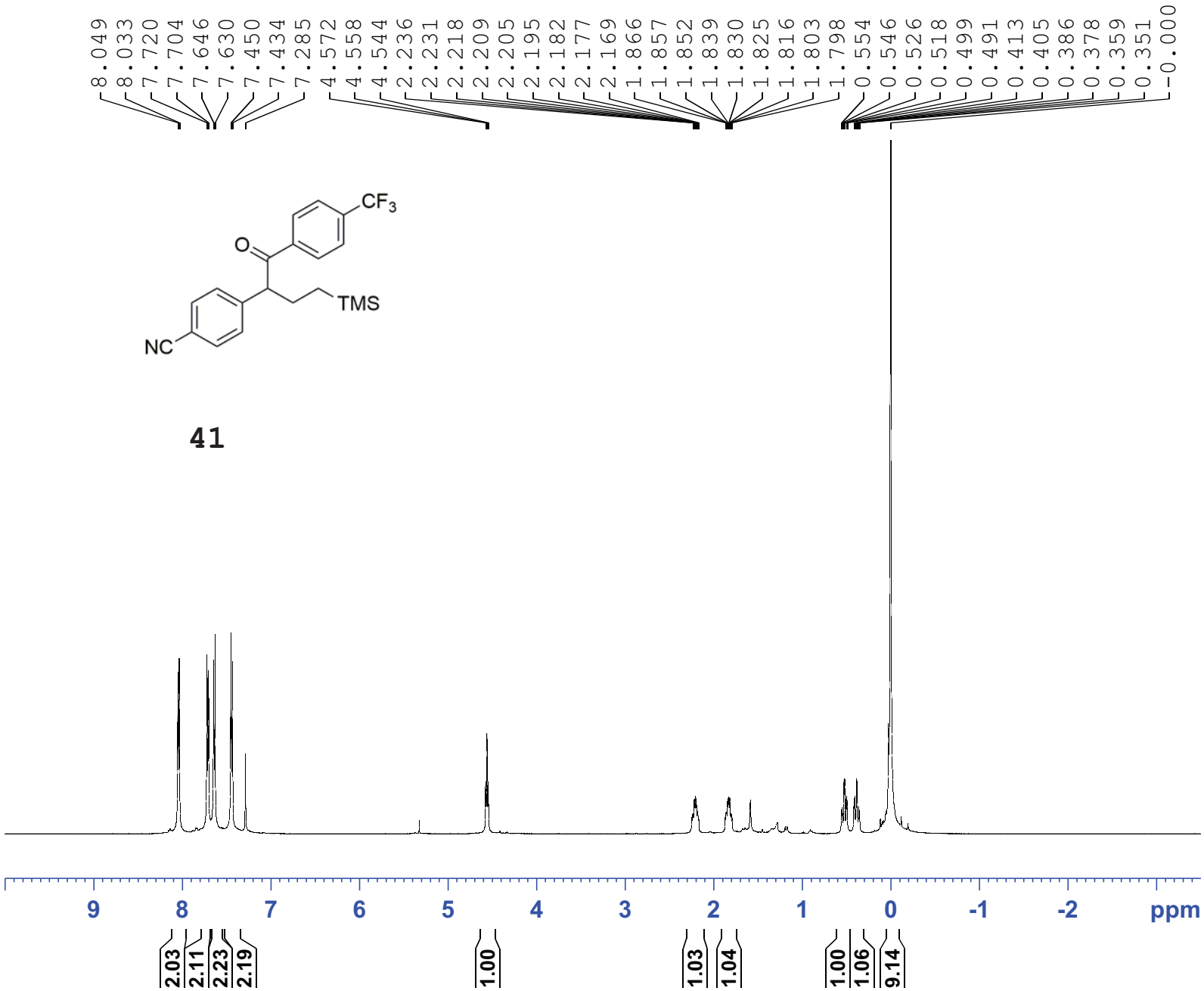


```
Current Data Parameters
NAME                  11157A
EXPNO                      1
PROCNO                     1

F2 - Acquisition Parameters
Date_              20220304
Time                  15.15
INSTRUM               spect
PROBHD    5 mm CPPBBO BB
PULPROG                zg30
TD                    65536
SOLVENT               CDCl3
NS                       16
DS                        2
SWH              10000.000 Hz
FIDRES               0.152588 Hz
AQ                3.2767999 sec
RG                    55.37
DW                  50.000 usec
DE                   6.50 usec
TE                   298.2 K
D1              1.00000000 sec
D11       0 sec
TD0                       1

======== CHANNEL f1 ========
SFO1           500.1330885 MHz
NUC1                      1H
P1                    11.25 usec
PLW1           20.00000000 W

======== CHANNEL f2 ========
SFO2           500.1330885 MHz
NUC2                    off
CPDPRG[2
PCPD2      0 usec
PLW2       0 W
PLW12      0 W
PLW13      0 W

F2 - Processing parameters
SI                    65536
SF           500.1299998 MHz
WDW                     EM
SSB        0
LB                   0.30 Hz
GB         0
PC                    1.00
```

Supplementary Figure 77. ^{1}H-NMR of compound **41**, recorded at 500 MHz and 25 °C in $CDCl_3$.

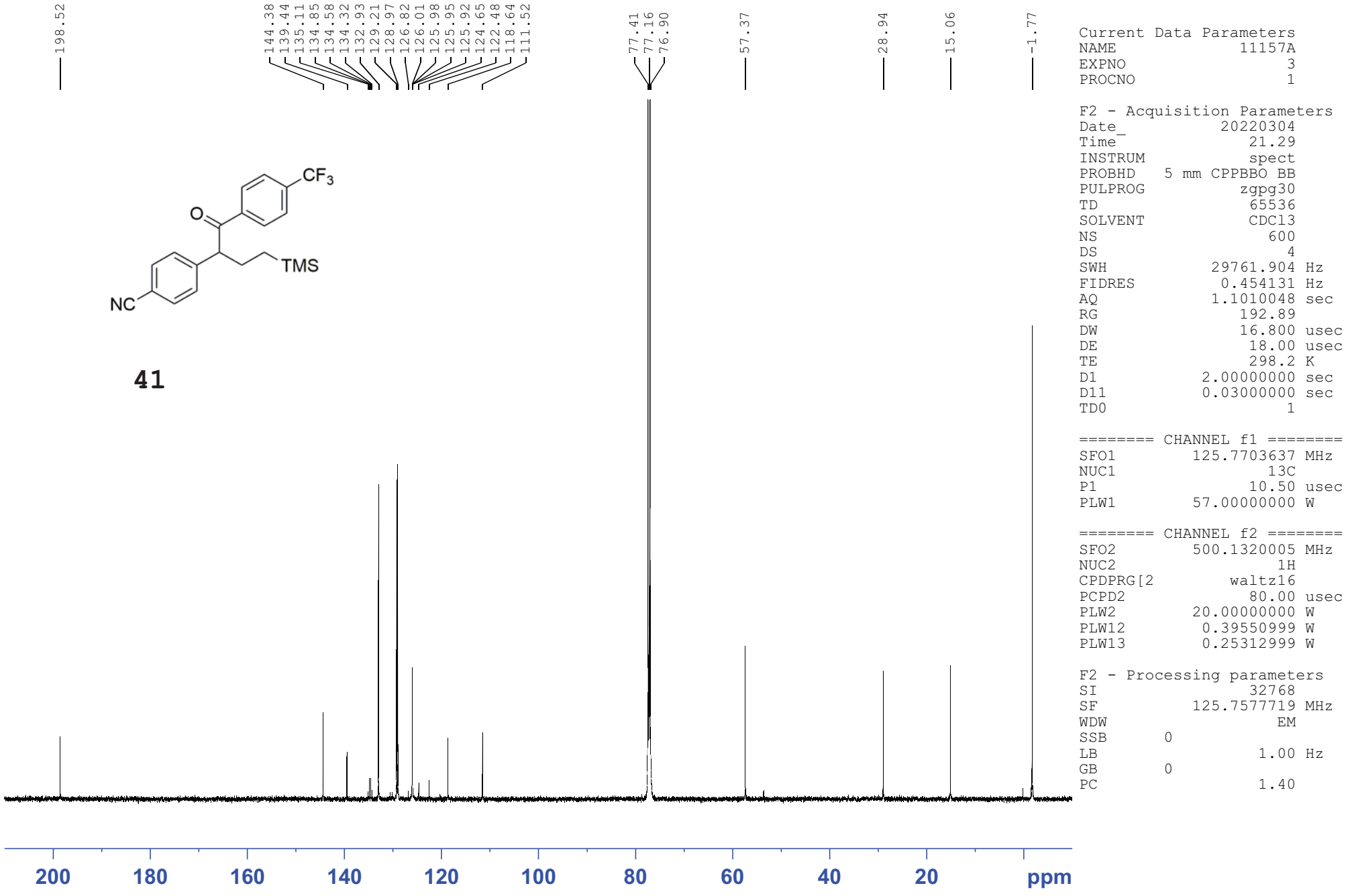

Supplementary Figure 78. ^{13}C-NMR of compound **41**, recorded at 126 MHz and 25 °C in $CDCl_3$.

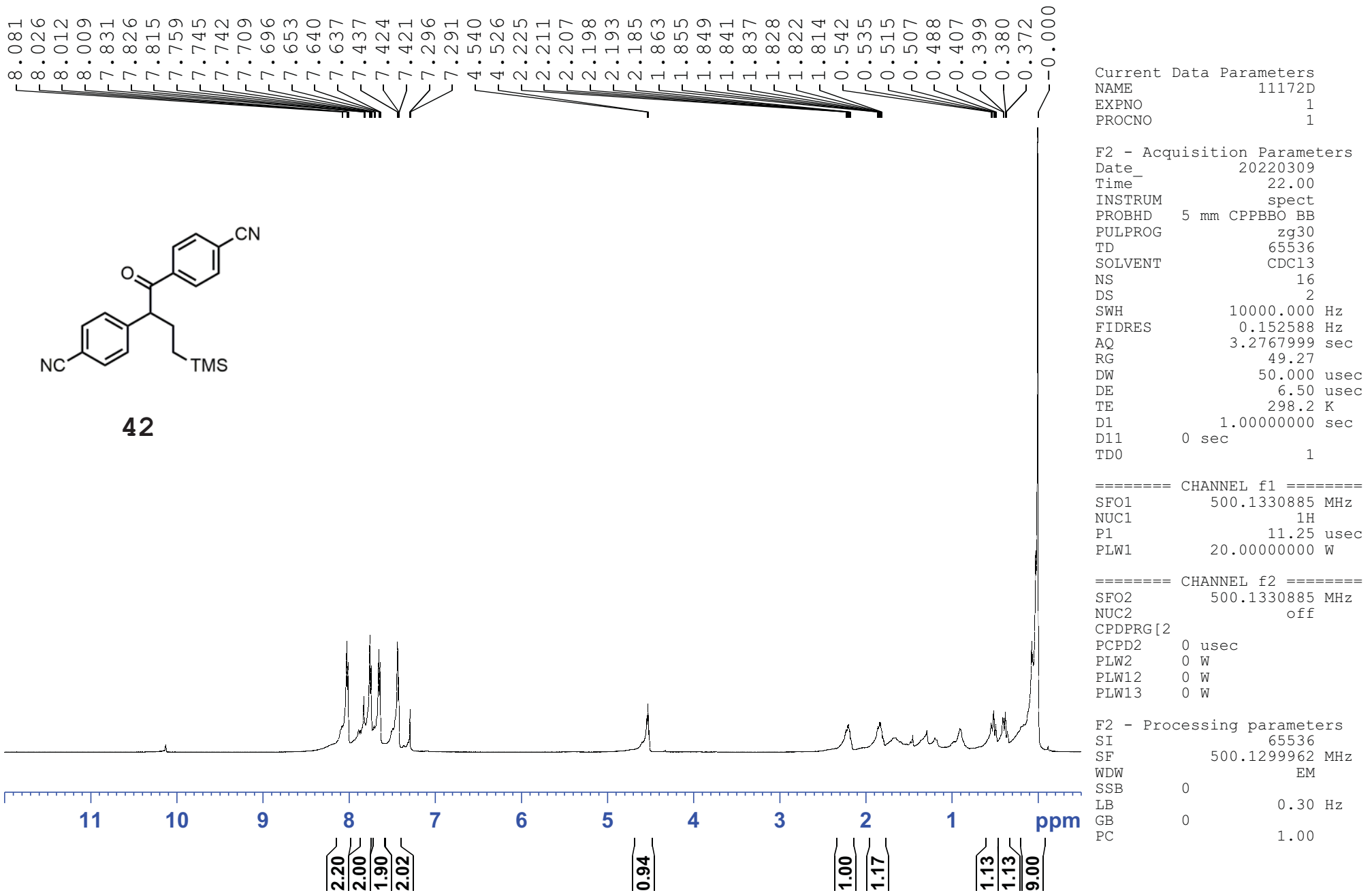


```
Current Data Parameters
NAME                  11172D
EXPNO                      1
PROCNO                     1

F2 - Acquisition Parameters
Date_              20220309
Time                 22.00
INSTRUM              spect
PROBHD    5 mm CPPBBO BB
PULPROG               zg30
TD                   65536
SOLVENT              CDCl3
NS                      16
DS                       2
SWH             10000.000 Hz
FIDRES              0.152588 Hz
AQ                3.2767999 sec
RG                   49.27
DW                  50.000 usec
DE                   6.50 usec
TE                  298.2 K
D1              1.00000000 sec
D11       0 sec
TD0                        1

======== CHANNEL f1 ========
SFO1           500.1330885 MHz
NUC1                    1H
P1                  11.25 usec
PLW1           20.00000000 W

======== CHANNEL f2 ========
SFO2           500.1330885 MHz
NUC2                   off
CPDPRG[2
PCPD2      0 usec
PLW2       0 W
PLW12      0 W
PLW13      0 W

F2 - Processing parameters
SI                   65536
SF             500.1299962 MHz
WDW                     EM
SSB        0
LB                  0.30 Hz
GB         0
PC                  1.00
```

Supplementary Figure 79. ^{1}H-NMR of compound **42**, recorded at 500 MHz and 25 °C in $CDCl_3$.

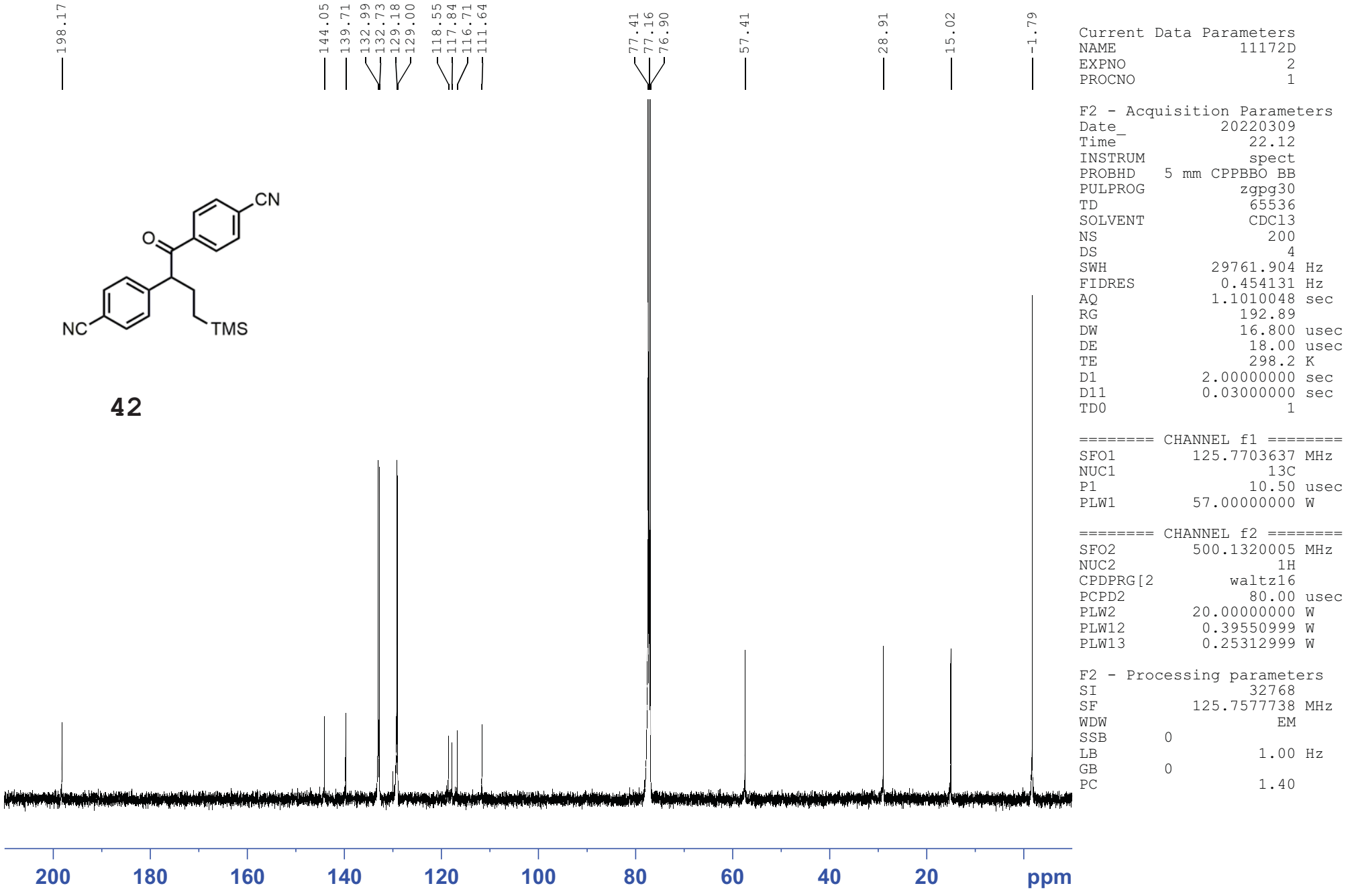

Supplementary Figure 80. ^{13}C-NMR of compound **42**, recorded at 126 MHz and 25 °C in $CDCl_3$.

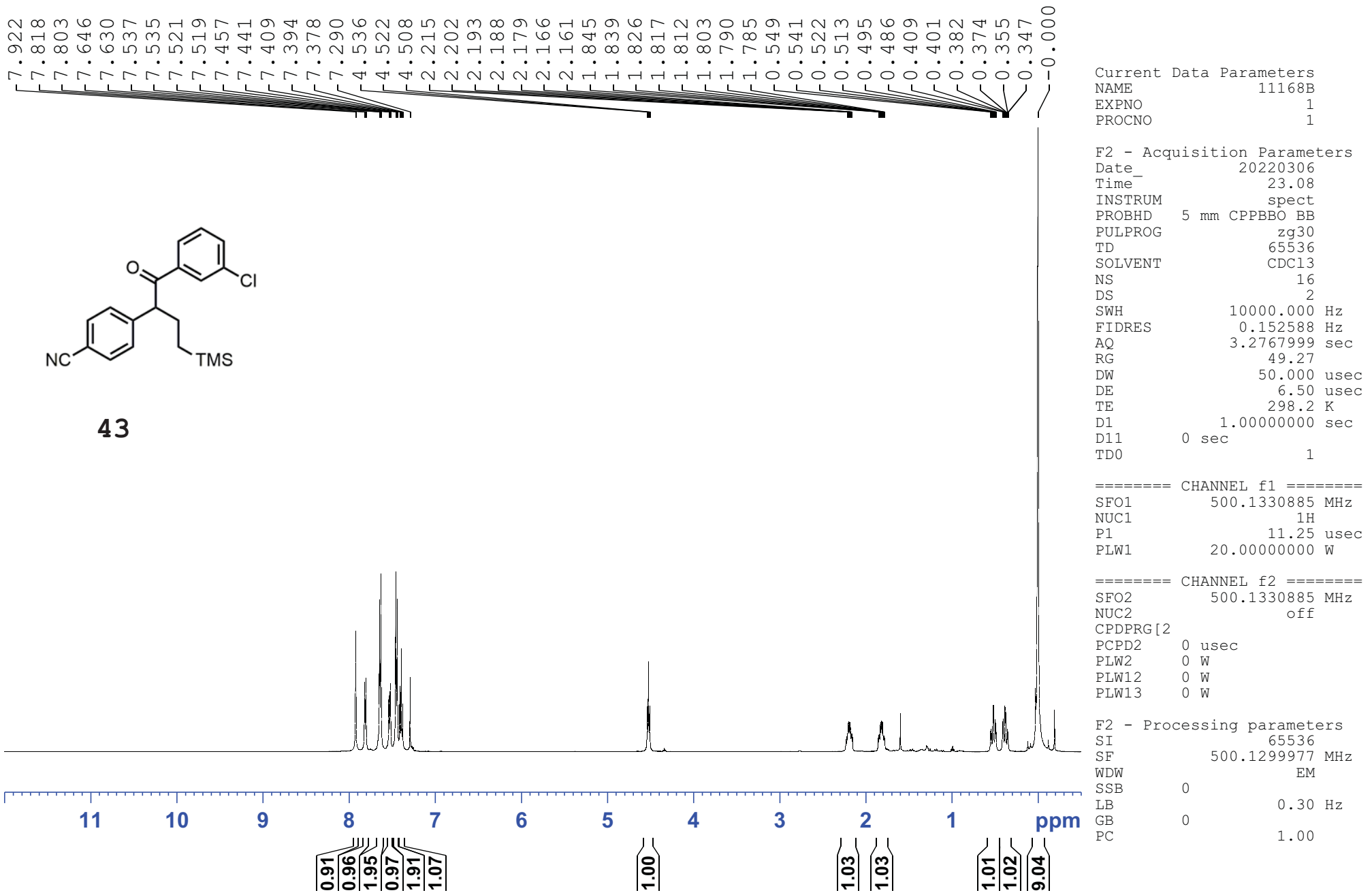


```
Current Data Parameters
NAME                  11168B
EXPNO                      1
PROCNO                     1

F2 - Acquisition Parameters
Date_              20220306
Time                 23.08
INSTRUM              spect
PROBHD    5 mm CPPBBO BB
PULPROG               zg30
TD                   65536
SOLVENT              CDCl3
NS                      16
DS                       2
SWH             10000.000 Hz
FIDRES              0.152588 Hz
AQ                3.2767999 sec
RG                   49.27
DW                  50.000 usec
DE                   6.50 usec
TE                  298.2 K
D1              1.00000000 sec
D11       0 sec
TD0                      1

======== CHANNEL f1 ========
SFO1           500.1330885 MHz
NUC1                    1H
P1                   11.25 usec
PLW1           20.00000000 W

======== CHANNEL f2 ========
SFO2           500.1330885 MHz
NUC2                   off
CPDPRG[2
PCPD2      0 usec
PLW2       0 W
PLW12      0 W
PLW13      0 W

F2 - Processing parameters
SI                   65536
SF             500.1299977 MHz
WDW                     EM
SSB        0
LB                  0.30 Hz
GB         0
PC                  1.00
```

Supplementary Figure 81. ^{1}H-NMR of compound **43**, recorded at 500 MHz and 25 °C in $CDCl_3$.

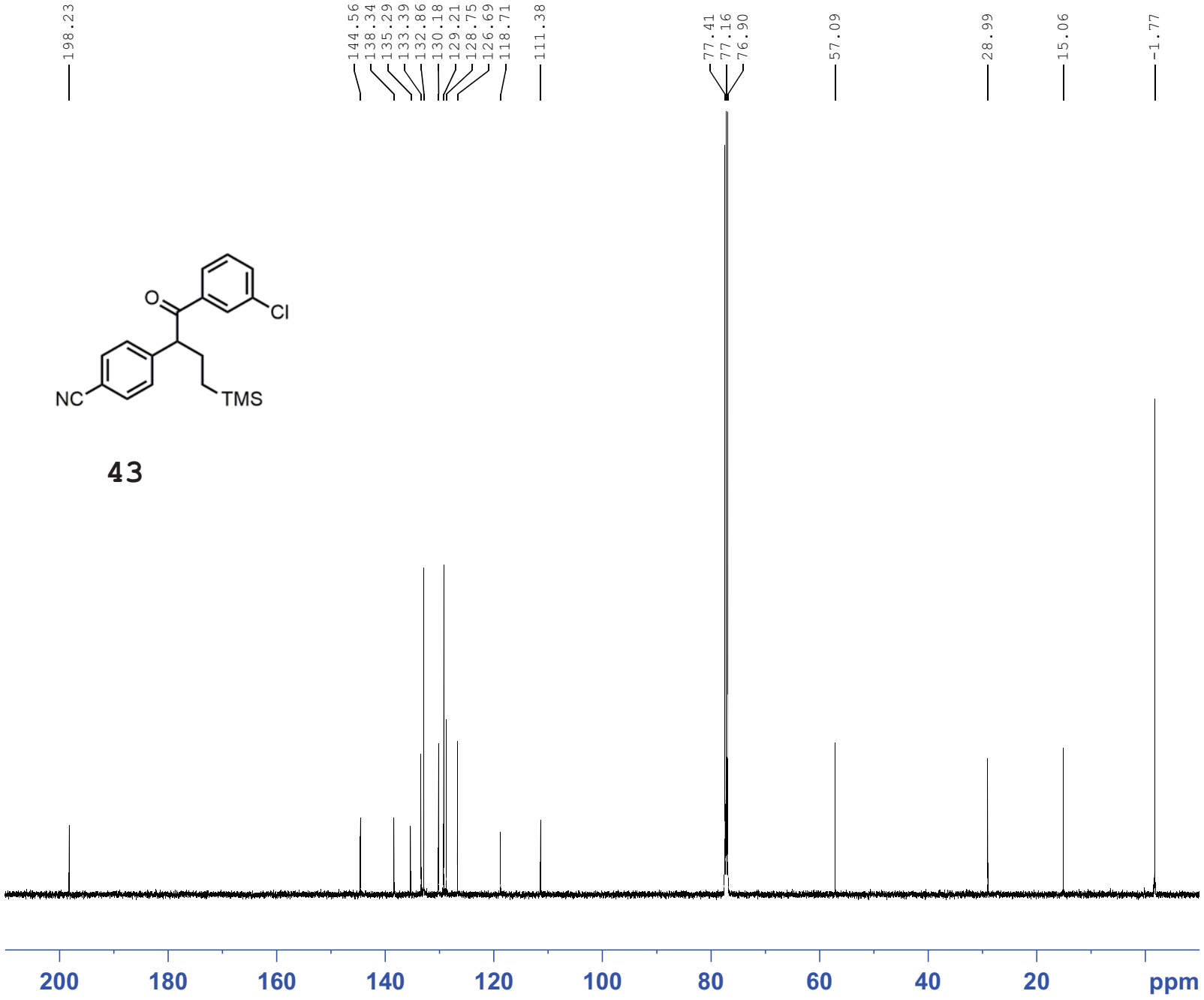


```
Current Data Parameters
NAME                 11168B
EXPNO                     2
PROCNO                    1

F2 - Acquisition Parameters
Date_              20220306
Time                 23.14
INSTRUM              spect
PROBHD   5 mm CPPBBO BB
PULPROG             zgpg30
TD                   65536
SOLVENT              CDCl3
NS                     100
DS                       4
SWH              29761.904 Hz
FIDRES            0.454131 Hz
AQ                1.1010048 sec
RG                   192.89
DW                   16.800 usec
DE                   18.00 usec
TE                   298.2 K
D1              2.00000000 sec
D11             0.03000000 sec
TD0                      1

======== CHANNEL f1 ========
SFO1           125.7703637 MHz
NUC1                   13C
P1                   10.50 usec
PLW1           57.00000000 W

======== CHANNEL f2 ========
SFO2           500.1320005 MHz
NUC2                   1H
CPDPRG[2           waltz16
PCPD2                80.00 usec
PLW2           20.00000000 W
PLW12           0.39550999 W
PLW13           0.25312999 W

F2 - Processing parameters
SI                     32768
SF             125.7577728 MHz
WDW                       EM
SSB          0
LB                    1.00 Hz
GB           0
PC                    1.40
```

Supplementary Figure 82. ^{13}C-NMR of compound **43**, recorded at 126 MHz and 25 °C in $CDCl_3$.

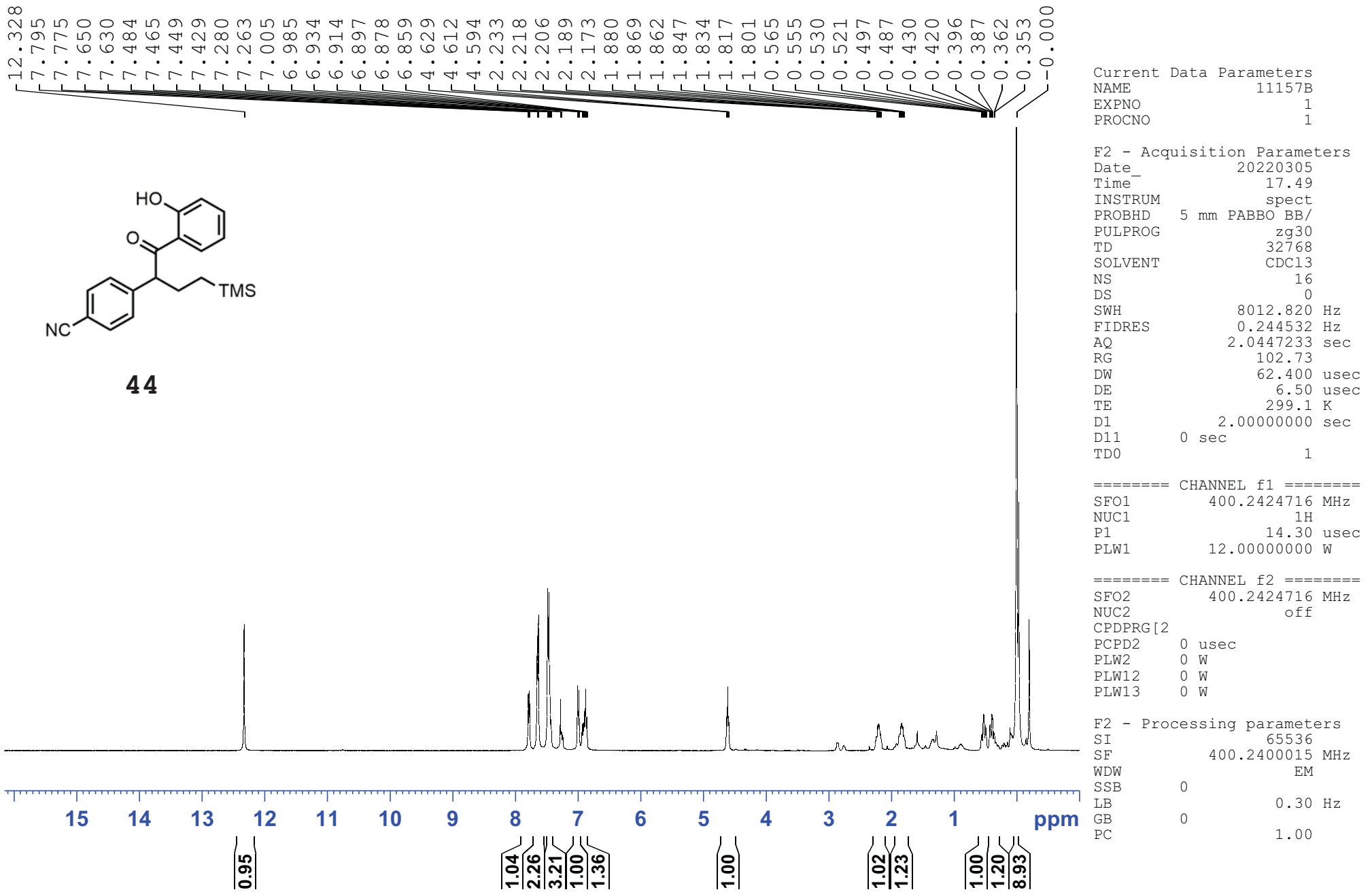


```
Current Data Parameters
NAME                  11157B
EXPNO                      1
PROCNO                     1

F2 - Acquisition Parameters
Date_              20220305
Time                  17.49
INSTRUM               spect
PROBHD    5 mm PABBO BB/
PULPROG                zg30
TD                    32768
SOLVENT               CDCl3
NS                       16
DS                        0
SWH               8012.820 Hz
FIDRES            0.244532 Hz
AQ                2.0447233 sec
RG                   102.73
DW                   62.400 usec
DE                    6.50 usec
TE                   299.1 K
D1              2.00000000 sec
D11       0 sec
TD0                       1

======== CHANNEL f1 ========
SFO1           400.2424716 MHz
NUC1                     1H
P1                   14.30 usec
PLW1           12.00000000 W

======== CHANNEL f2 ========
SFO2           400.2424716 MHz
NUC2                   off
CPDPRG[2
PCPD2      0 usec
PLW2       0 W
PLW12      0 W
PLW13      0 W

F2 - Processing parameters
SI                    65536
SF             400.2400015 MHz
WDW                      EM
SSB        0
LB                   0.30 Hz
GB         0
PC                   1.00
```

Supplementary Figure 83. ^{1}H-NMR of compound **44**, recorded at 400 MHz and 25 °C in $CDCl_3$.

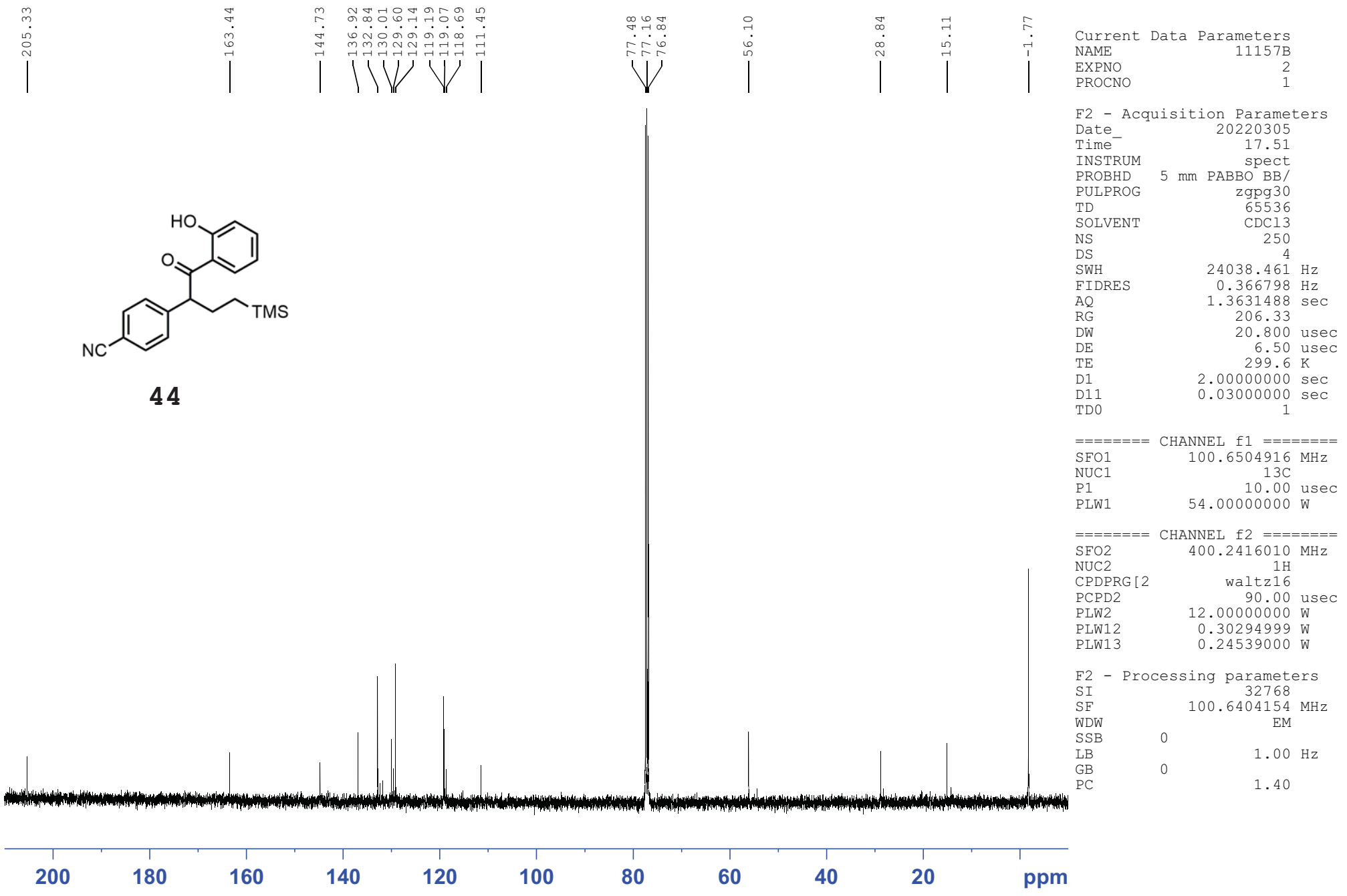


```
Current Data Parameters
NAME                 11157B
EXPNO                     2
PROCNO                    1

F2 - Acquisition Parameters
Date_              20220305
Time                 17.51
INSTRUM              spect
PROBHD   5 mm PABBO BB/
PULPROG             zgpg30
TD                   65536
SOLVENT              CDCl3
NS                     250
DS                       4
SWH             24038.461 Hz
FIDRES           0.366798 Hz
AQ               1.3631488 sec
RG                  206.33
DW                  20.800 usec
DE                   6.50 usec
TE                  299.6 K
D1             2.00000000 sec
D11            0.03000000 sec
TD0                      1

======== CHANNEL f1 ========
SFO1           100.6504916 MHz
NUC1                   13C
P1                  10.00 usec
PLW1           54.00000000 W

======== CHANNEL f2 ========
SFO2           400.2416010 MHz
NUC2                   1H
CPDPRG[2           waltz16
PCPD2               90.00 usec
PLW2           12.00000000 W
PLW12           0.30294999 W
PLW13           0.24539000 W

F2 - Processing parameters
SI                   32768
SF             100.6404154 MHz
WDW                     EM
SSB       0
LB                   1.00 Hz
GB        0
PC                   1.40
```

Supplementary Figure 84. ^{13}C-NMR of compound **44**, recorded at 101 MHz and 25 °C in $CDCl_3$.

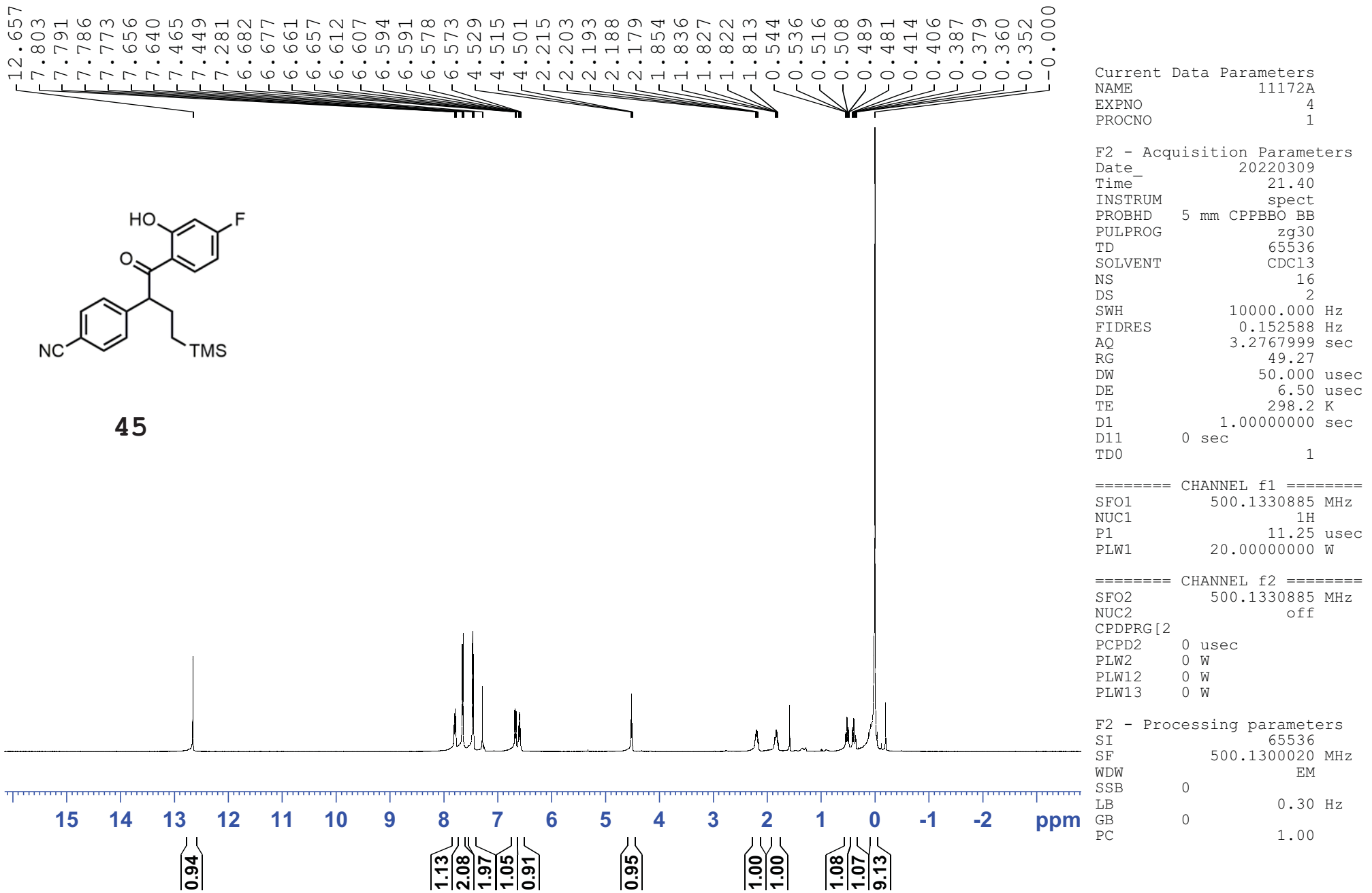


```
Current Data Parameters
NAME                  11172A
EXPNO                      4
PROCNO                     1

F2 - Acquisition Parameters
Date_              20220309
Time                 21.40
INSTRUM              spect
PROBHD    5 mm CPPBBO BB
PULPROG               zg30
TD                   65536
SOLVENT              CDCl3
NS                      16
DS                       2
SWH             10000.000 Hz
FIDRES           0.152588 Hz
AQ               3.2767999 sec
RG                   49.27
DW                  50.000 usec
DE                   6.50 usec
TE                  298.2 K
D1              1.00000000 sec
D11       0 sec
TD0                      1

======== CHANNEL f1 ========
SFO1          500.1330885 MHz
NUC1                    1H
P1                  11.25 usec
PLW1          20.00000000 W

======== CHANNEL f2 ========
SFO2          500.1330885 MHz
NUC2                   off
CPDPRG[2
PCPD2      0 usec
PLW2       0 W
PLW12      0 W
PLW13      0 W

F2 - Processing parameters
SI                   65536
SF            500.1300020 MHz
WDW                     EM
SSB        0
LB                   0.30 Hz
GB         0
PC                   1.00
```

Supplementary Figure 85. ^{1}H-NMR of compound **45**, recorded at 500 MHz and 25 °C in $CDCl_3$.

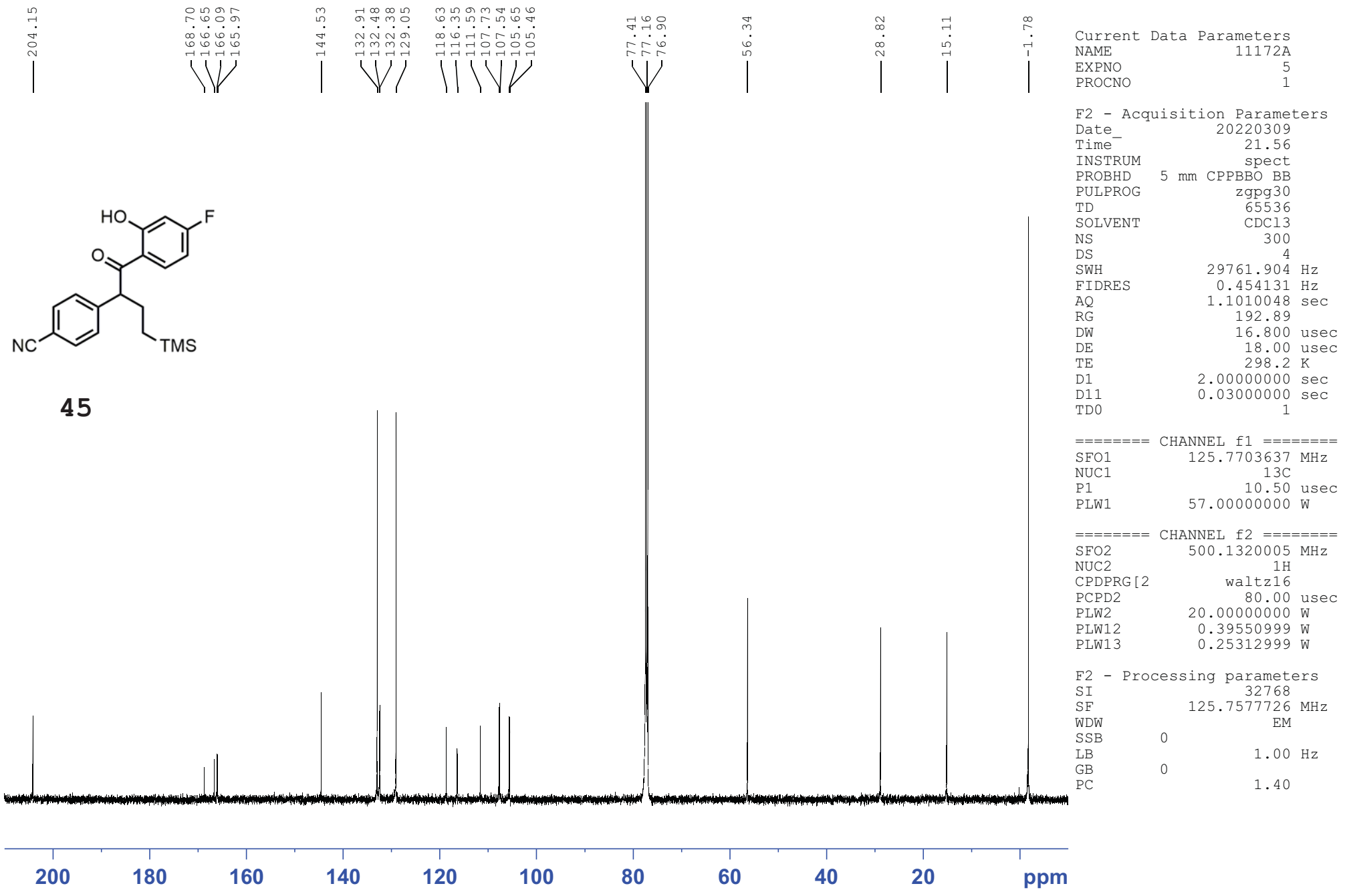

Supplementary Figure 86. ^{13}C-NMR of compound **45**, recorded at 126 MHz and 25 °C in $CDCl_3$.

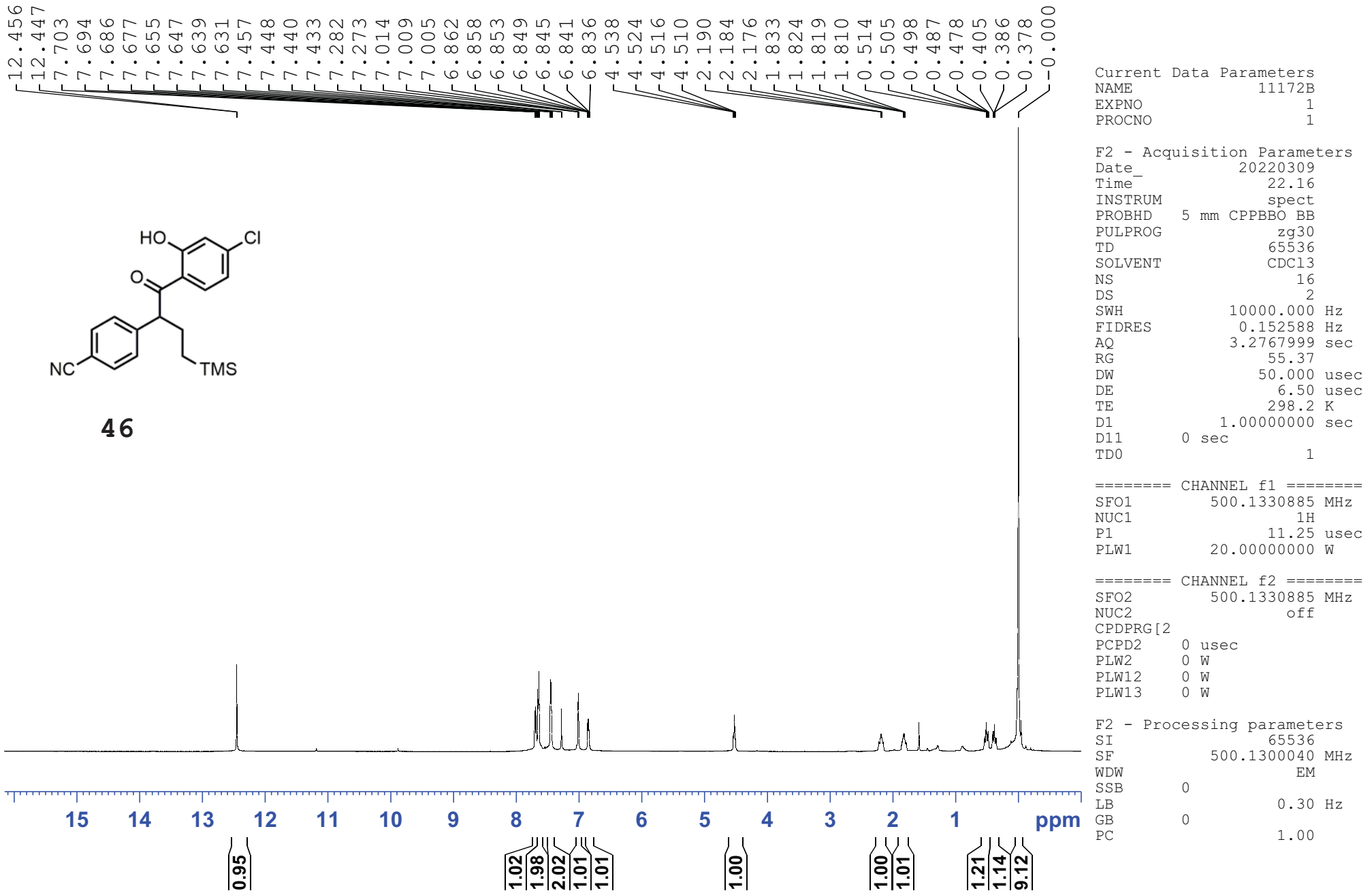


```
Current Data Parameters
NAME                  11172B
EXPNO                      1
PROCNO                     1

F2 - Acquisition Parameters
Date_              20220309
Time                  22.16
INSTRUM               spect
PROBHD    5 mm CPPBBO BB
PULPROG                zg30
TD                    65536
SOLVENT               CDCl3
NS                       16
DS                        2
SWH              10000.000 Hz
FIDRES            0.152588 Hz
AQ               3.2767999 sec
RG                    55.37
DW                  50.000 usec
DE                   6.50 usec
TE                  298.2 K
D1             1.00000000 sec
D11       0 sec
TD0                       1

======== CHANNEL f1 ========
SFO1           500.1330885 MHz
NUC1                      1H
P1                   11.25 usec
PLW1          20.00000000 W

======== CHANNEL f2 ========
SFO2           500.1330885 MHz
NUC2                    off
CPDPRG[2
PCPD2      0 usec
PLW2       0 W
PLW12      0 W
PLW13      0 W

F2 - Processing parameters
SI                    65536
SF           500.1300040 MHz
WDW                      EM
SSB        0
LB                   0.30 Hz
GB         0
PC                   1.00
```

Supplementary Figure 87. ^{1}H-NMR of compound **46**, recorded at 500 MHz and 25 °C in $CDCl_3$.

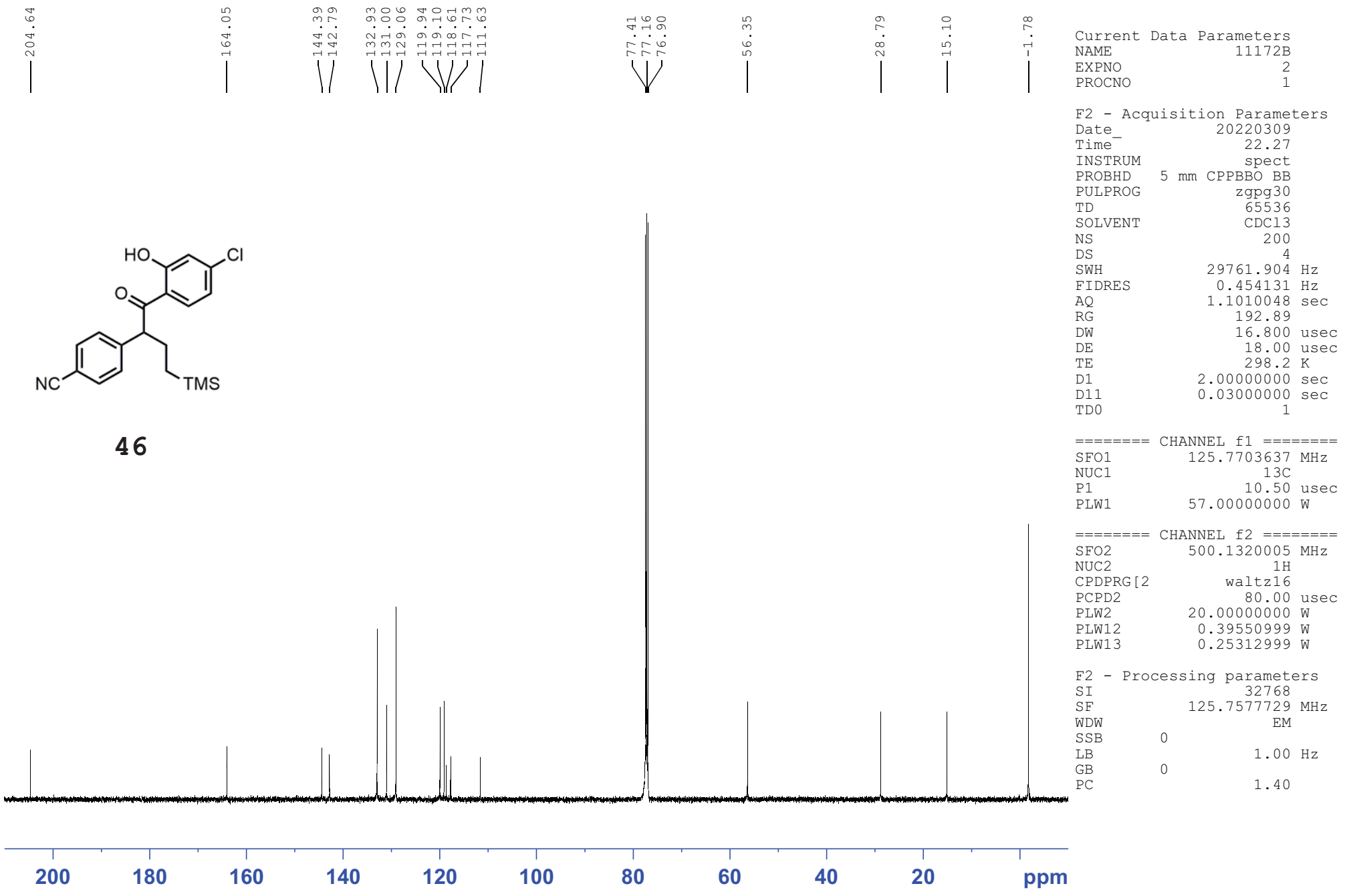


```
Current Data Parameters
NAME                  11172B
EXPNO                      2
PROCNO                     1

F2 - Acquisition Parameters
Date_              20220309
Time                  22.27
INSTRUM               spect
PROBHD    5 mm CPPBBO BB
PULPROG              zgpg30
TD                    65536
SOLVENT               CDCl3
NS                      200
DS                        4
SWH              29761.904 Hz
FIDRES            0.454131 Hz
AQ                1.1010048 sec
RG                   192.89
DW                   16.800 usec
DE                   18.00 usec
TE                   298.2 K
D1              2.00000000 sec
D11             0.03000000 sec
TD0                       1

======== CHANNEL f1 ========
SFO1           125.7703637 MHz
NUC1                    13C
P1                   10.50 usec
PLW1           57.00000000 W

======== CHANNEL f2 ========
SFO2           500.1320005 MHz
NUC2                    1H
CPDPRG[2            waltz16
PCPD2                80.00 usec
PLW2           20.00000000 W
PLW12           0.39550999 W
PLW13           0.25312999 W

F2 - Processing parameters
SI                    32768
SF             125.7577729 MHz
WDW                      EM
SSB          0
LB                    1.00 Hz
GB           0
PC                    1.40
```

Supplementary Figure 88. ^{13}C-NMR of compound **46**, recorded at 126 MHz and 25 °C in $CDCl_3$.

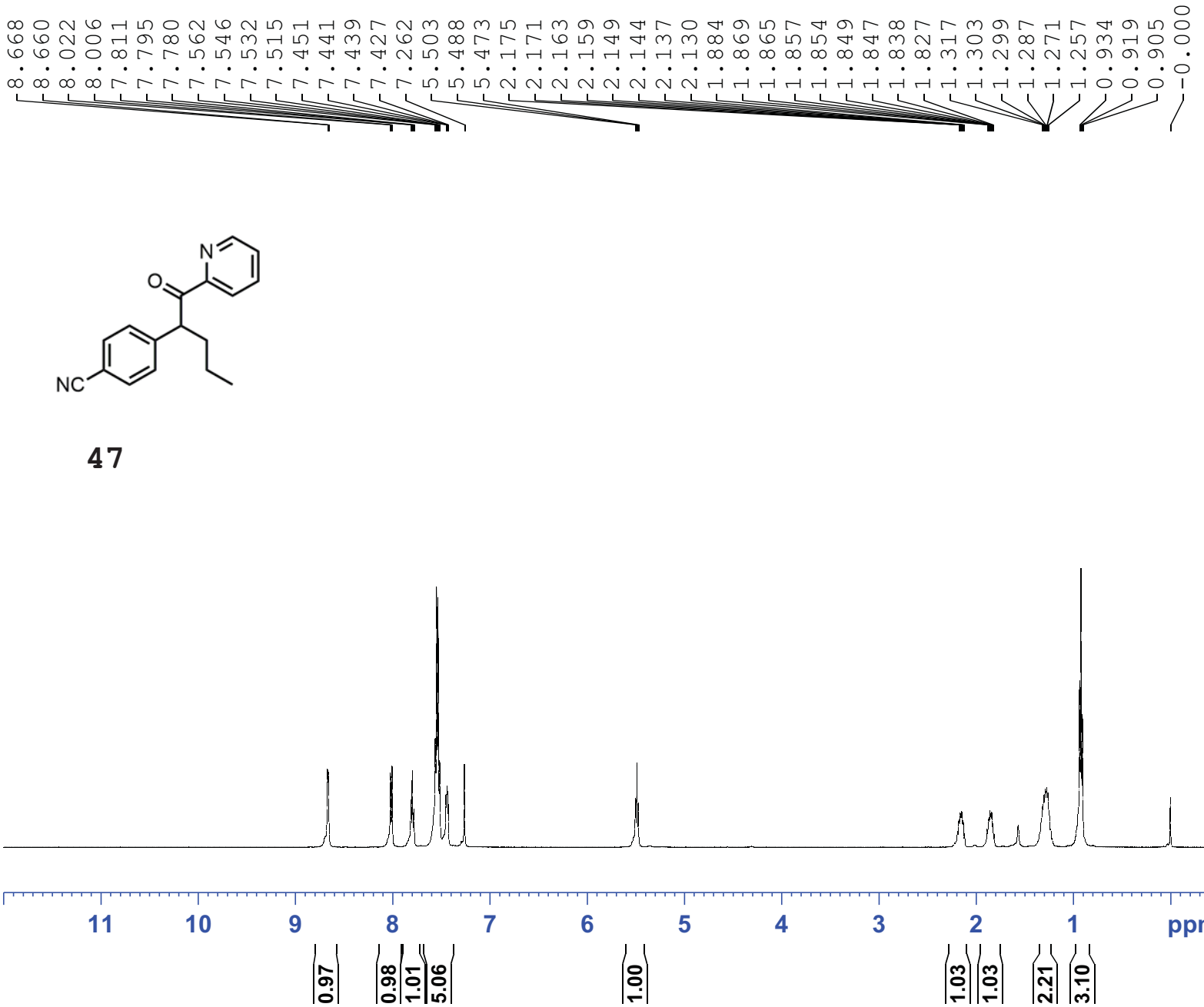


```
Current Data Parameters
NAME                  11154A
EXPNO                      1
PROCNO                     1

F2 - Acquisition Parameters
Date_              20220301
Time                   19.27
INSTRUM                spect
PROBHD    5 mm CPPBBO BB
PULPROG                zg30
TD                    65536
SOLVENT               CDCl3
NS                       16
DS                        2
SWH              10000.000 Hz
FIDRES               0.152588 Hz
AQ               3.2767999 sec
RG                    55.37
DW                  50.000 usec
DE                   6.50 usec
TE                   298.2 K
D1             1.00000000 sec
D11       0 sec
TD0                       1

======== CHANNEL f1 ========
SFO1           500.1330885 MHz
NUC1                      1H
P1                   11.25 usec
PLW1           20.00000000 W

======== CHANNEL f2 ========
SFO2           500.1330885 MHz
NUC2                     off
CPDPRG[2
PCPD2      0 usec
PLW2       0 W
PLW12      0 W
PLW13      0 W

F2 - Processing parameters
SI                    65536
SF               500.1300122 MHz
WDW                      EM
SSB        0
LB                    0.30 Hz
GB         0
PC                    1.00
```

Supplementary Figure 89. ^{1}H-NMR of compound **47**, recorded at 500 MHz and 25 °C in $CDCl_3$.

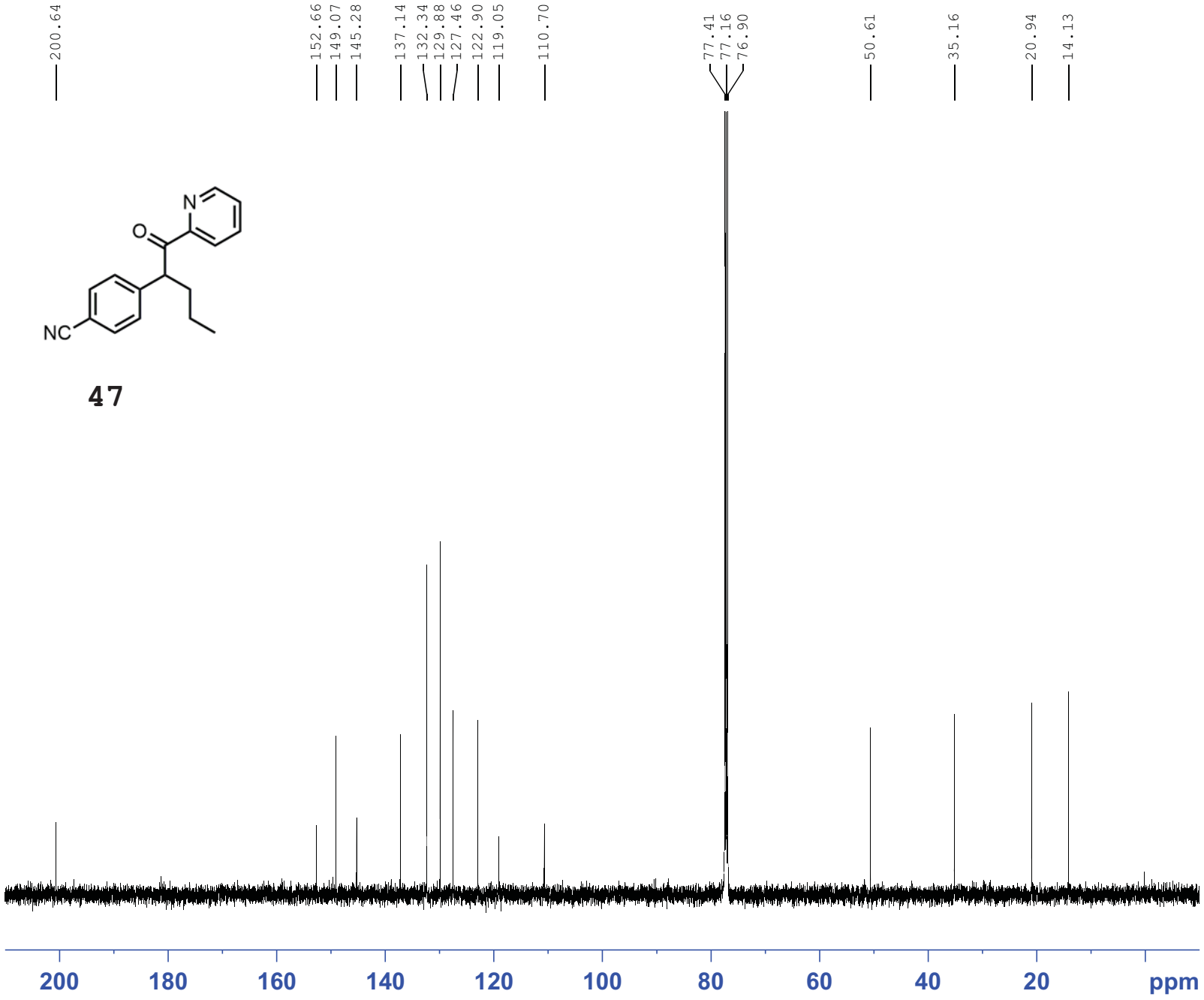


```
Current Data Parameters
NAME                 11154A
EXPNO                     2
PROCNO                    1

F2 - Acquisition Parameters
Date_              20220301
Time                 19.29
INSTRUM              spect
PROBHD   5 mm CPPBBO BB
PULPROG             zgpg30
TD                   65536
SOLVENT              CDCl3
NS                      76
DS                       4
SWH              29761.904 Hz
FIDRES            0.454131 Hz
AQ                1.1010048 sec
RG                  192.89
DW                  16.800 usec
DE                  18.00 usec
TE                  298.2 K
D1              2.00000000 sec
D11             0.03000000 sec
TD0                      1

======== CHANNEL f1 ========
SFO1           125.7703637 MHz
NUC1                   13C
P1                  10.50 usec
PLW1           57.00000000 W

======== CHANNEL f2 ========
SFO2           500.1320005 MHz
NUC2                   1H
CPDPRG[2           waltz16
PCPD2               80.00 usec
PLW2           20.00000000 W
PLW12           0.39550999 W
PLW13           0.25312999 W

F2 - Processing parameters
SI                    32768
SF             125.7577720 MHz
WDW                      EM
SSB          0
LB                    1.00 Hz
GB           0
PC                    1.40
```

Supplementary Figure 90. ^{13}C-NMR of compound **47**, recorded at 126 MHz and 25 °C in $CDCl_3$.

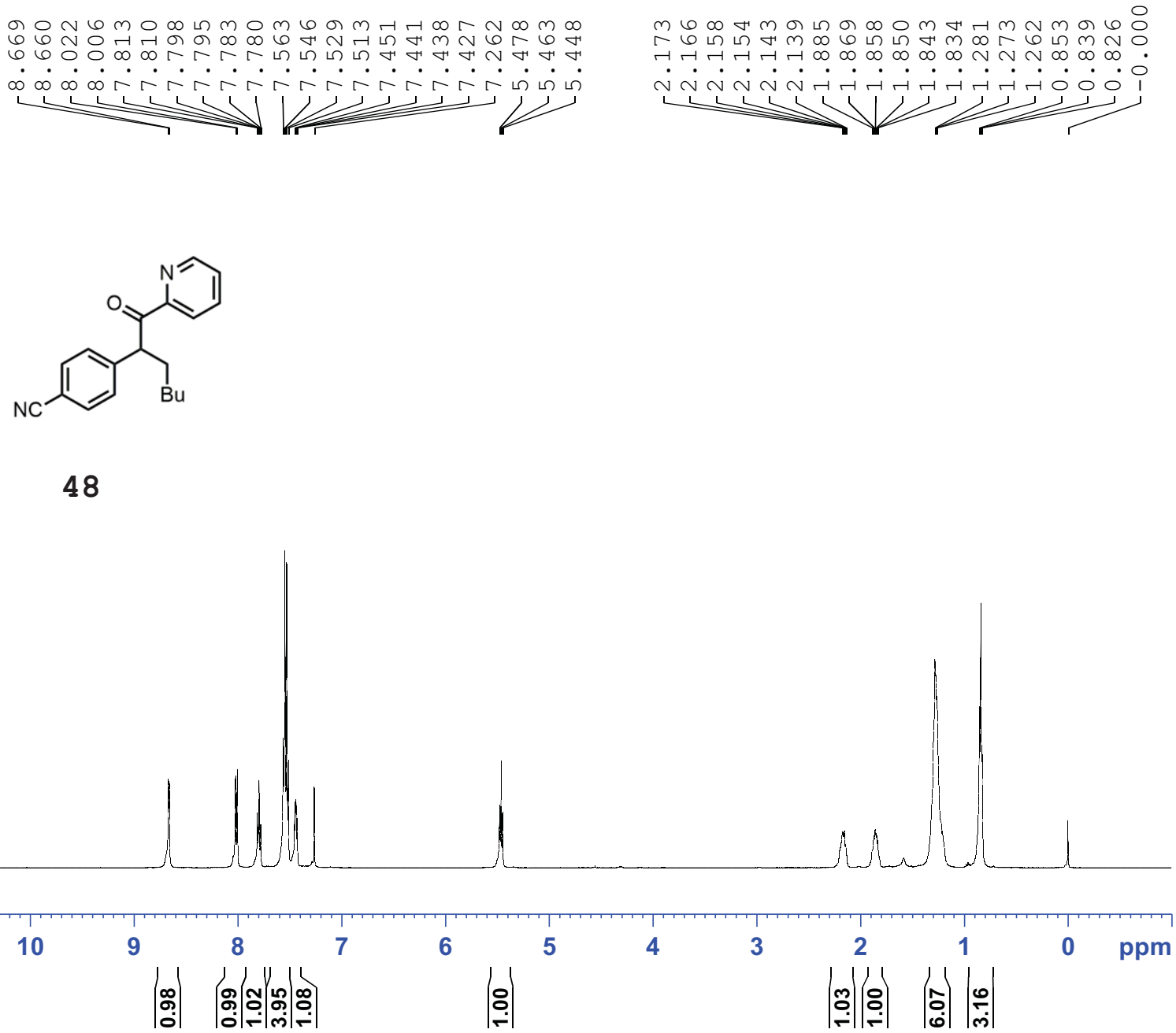


```
Current Data Parameters
NAME                  11154B
EXPNO                      1
PROCNO                     1

F2 - Acquisition Parameters
Date_              20220301
Time                  19.36
INSTRUM               spect
PROBHD    5 mm CPPBBO BB
PULPROG                zg30
TD                    65536
SOLVENT               CDCl3
NS                       16
DS                        2
SWH              10000.000 Hz
FIDRES               0.152588 Hz
AQ               3.2767999 sec
RG                    55.37
DW                   50.000 usec
DE                    6.50 usec
TE                   298.2 K
D1             1.00000000 sec
D11       0 sec
TD0                       1

======== CHANNEL f1 ========
SFO1           500.1330885 MHz
NUC1                      1H
P1                    11.25 usec
PLW1           20.00000000 W

======== CHANNEL f2 ========
SFO2           500.1330885 MHz
NUC2                    off
CPDPRG[2
PCPD2      0 usec
PLW2       0 W
PLW12      0 W
PLW13      0 W

F2 - Processing parameters
SI                    65536
SF           500.1300115 MHz
WDW                     EM
SSB        0
LB                    0.30 Hz
GB         0
PC                    1.00
```

Supplementary Figure 91. ^{1}H-NMR of compound **48**, recorded at 500 MHz and 25 °C in $CDCl_3$.

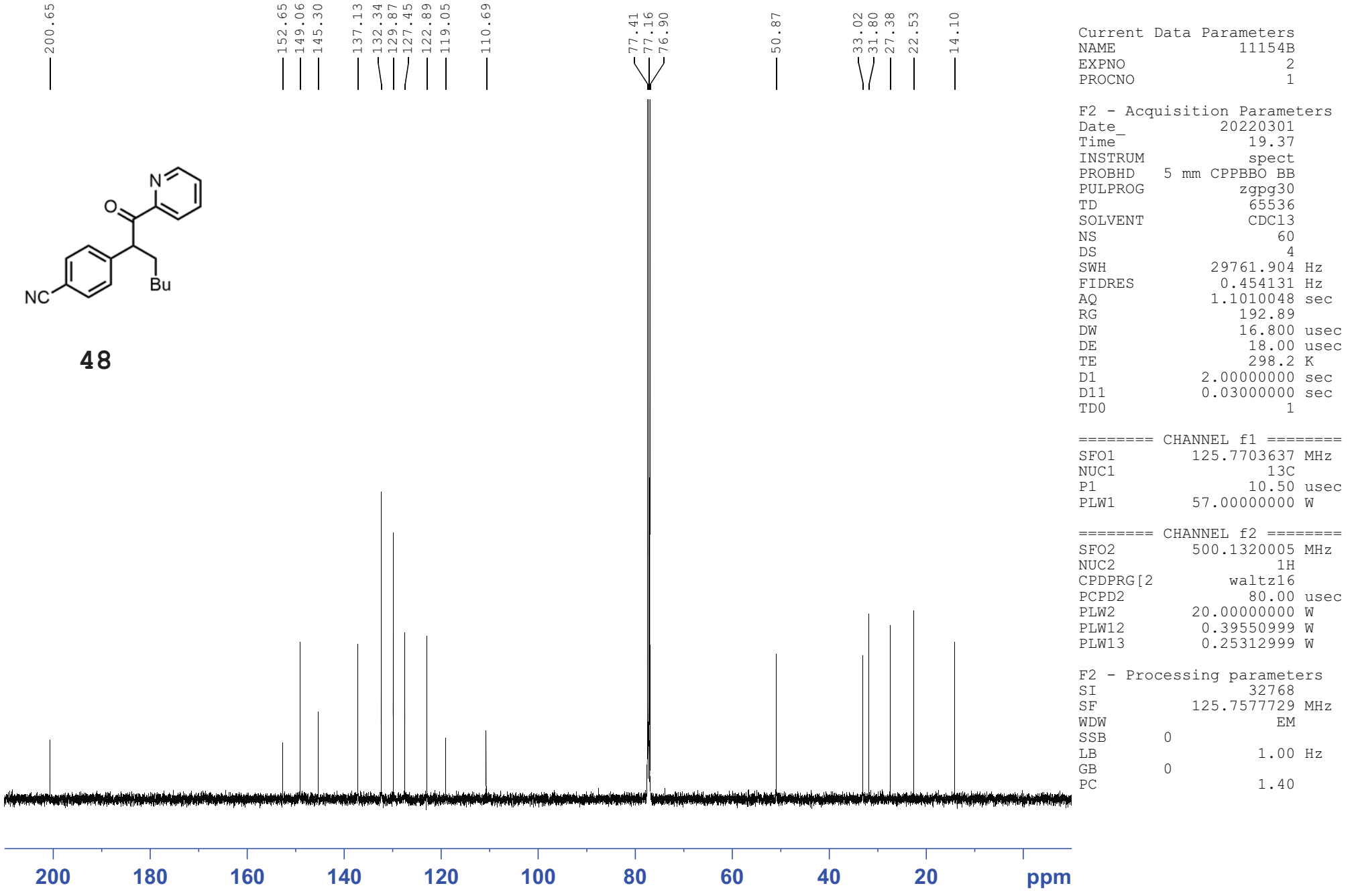


```
Current Data Parameters
NAME                 11154B
EXPNO                     2
PROCNO                    1

F2 - Acquisition Parameters
Date_              20220301
Time                 19.37
INSTRUM              spect
PROBHD   5 mm CPPBBO BB
PULPROG              zgpg30
TD                    65536
SOLVENT               CDCl3
NS                       60
DS                        4
SWH              29761.904 Hz
FIDRES            0.454131 Hz
AQ                1.1010048 sec
RG                   192.89
DW                   16.800 usec
DE                   18.00 usec
TE                   298.2 K
D1              2.00000000 sec
D11             0.03000000 sec
TD0                       1

======== CHANNEL f1 ========
SFO1           125.7703637 MHz
NUC1                    13C
P1                   10.50 usec
PLW1           57.00000000 W

======== CHANNEL f2 ========
SFO2           500.1320005 MHz
NUC2                    1H
CPDPRG[2            waltz16
PCPD2                80.00 usec
PLW2           20.00000000 W
PLW12           0.39550999 W
PLW13           0.25312999 W

F2 - Processing parameters
SI                    32768
SF             125.7577729 MHz
WDW                      EM
SSB          0
LB                    1.00 Hz
GB           0
PC                    1.40
```

Supplementary Figure 92. ^{13}C-NMR of compound **48**, recorded at 126 MHz and 25 °C in $CDCl_3$.

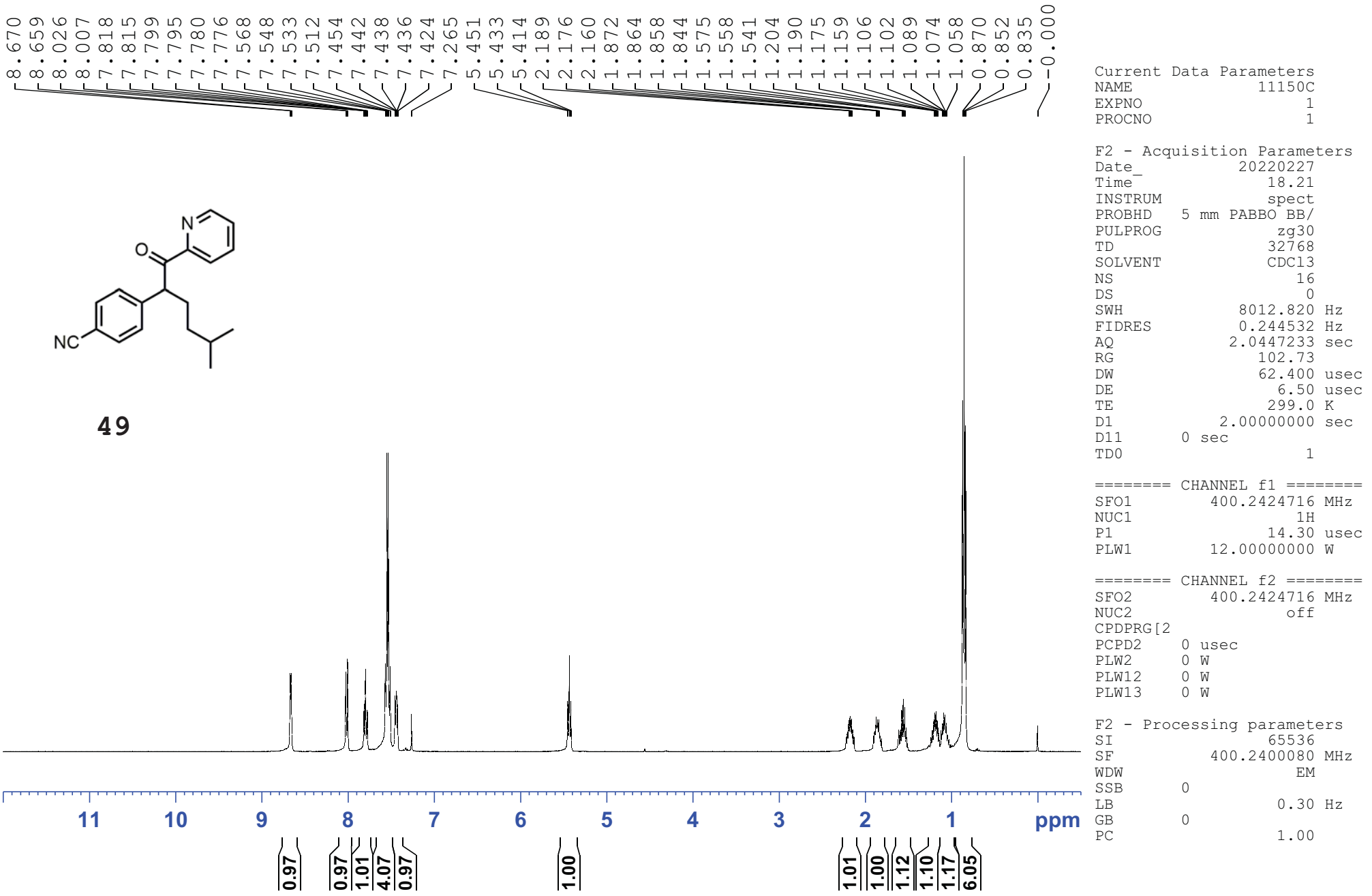


```
Current Data Parameters
NAME                  11150C
EXPNO                      1
PROCNO                     1

F2 - Acquisition Parameters
Date_              20220227
Time                 18.21
INSTRUM              spect
PROBHD    5 mm PABBO BB/
PULPROG               zg30
TD                   32768
SOLVENT              CDCl3
NS                      16
DS                       0
SWH               8012.820 Hz
FIDRES             0.244532 Hz
AQ                2.0447233 sec
RG                   102.73
DW                   62.400 usec
DE                    6.50 usec
TE                   299.0 K
D1              2.00000000 sec
D11       0 sec
TD0                      1

======== CHANNEL f1 ========
SFO1           400.2424716 MHz
NUC1                    1H
P1                   14.30 usec
PLW1           12.00000000 W

======== CHANNEL f2 ========
SFO2           400.2424716 MHz
NUC2                   off
CPDPRG[2
PCPD2      0 usec
PLW2       0 W
PLW12      0 W
PLW13      0 W

F2 - Processing parameters
SI                    65536
SF           400.2400080 MHz
WDW                     EM
SSB        0
LB                   0.30 Hz
GB         0
PC                   1.00
```

Supplementary Figure 93. ^{1}H-NMR of compound **49**, recorded at 400 MHz and 25 °C in $CDCl_3$.

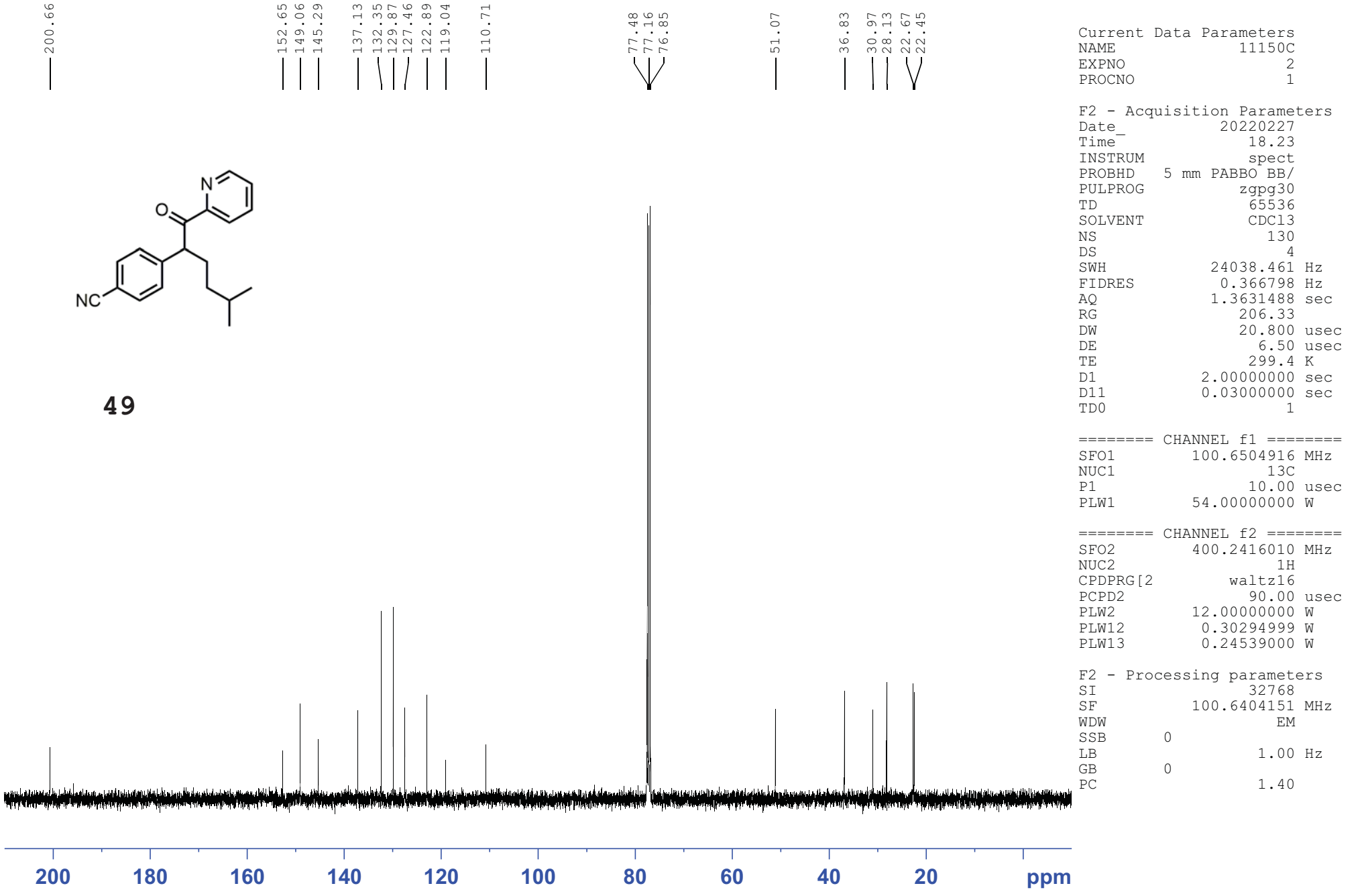


```
Current Data Parameters
NAME                 11150C
EXPNO                     2
PROCNO                    1

F2 - Acquisition Parameters
Date_              20220227
Time                 18.23
INSTRUM              spect
PROBHD    5 mm PABBO BB/
PULPROG             zgpg30
TD                   65536
SOLVENT              CDCl3
NS                     130
DS                       4
SWH             24038.461 Hz
FIDRES           0.366798 Hz
AQ               1.3631488 sec
RG                  206.33
DW                  20.800 usec
DE                   6.50 usec
TE                  299.4 K
D1             2.00000000 sec
D11            0.03000000 sec
TD0                      1

======== CHANNEL f1 ========
SFO1           100.6504916 MHz
NUC1                   13C
P1                  10.00 usec
PLW1           54.00000000 W

======== CHANNEL f2 ========
SFO2           400.2416010 MHz
NUC2                   1H
CPDPRG[2           waltz16
PCPD2               90.00 usec
PLW2           12.00000000 W
PLW12           0.30294999 W
PLW13           0.24539000 W

F2 - Processing parameters
SI                    32768
SF             100.6404151 MHz
WDW                      EM
SSB          0
LB                    1.00 Hz
GB           0
PC                    1.40
```

Supplementary Figure 94. ^{13}C-NMR of compound **49**, recorded at 101 MHz and 25 °C in $CDCl_3$.

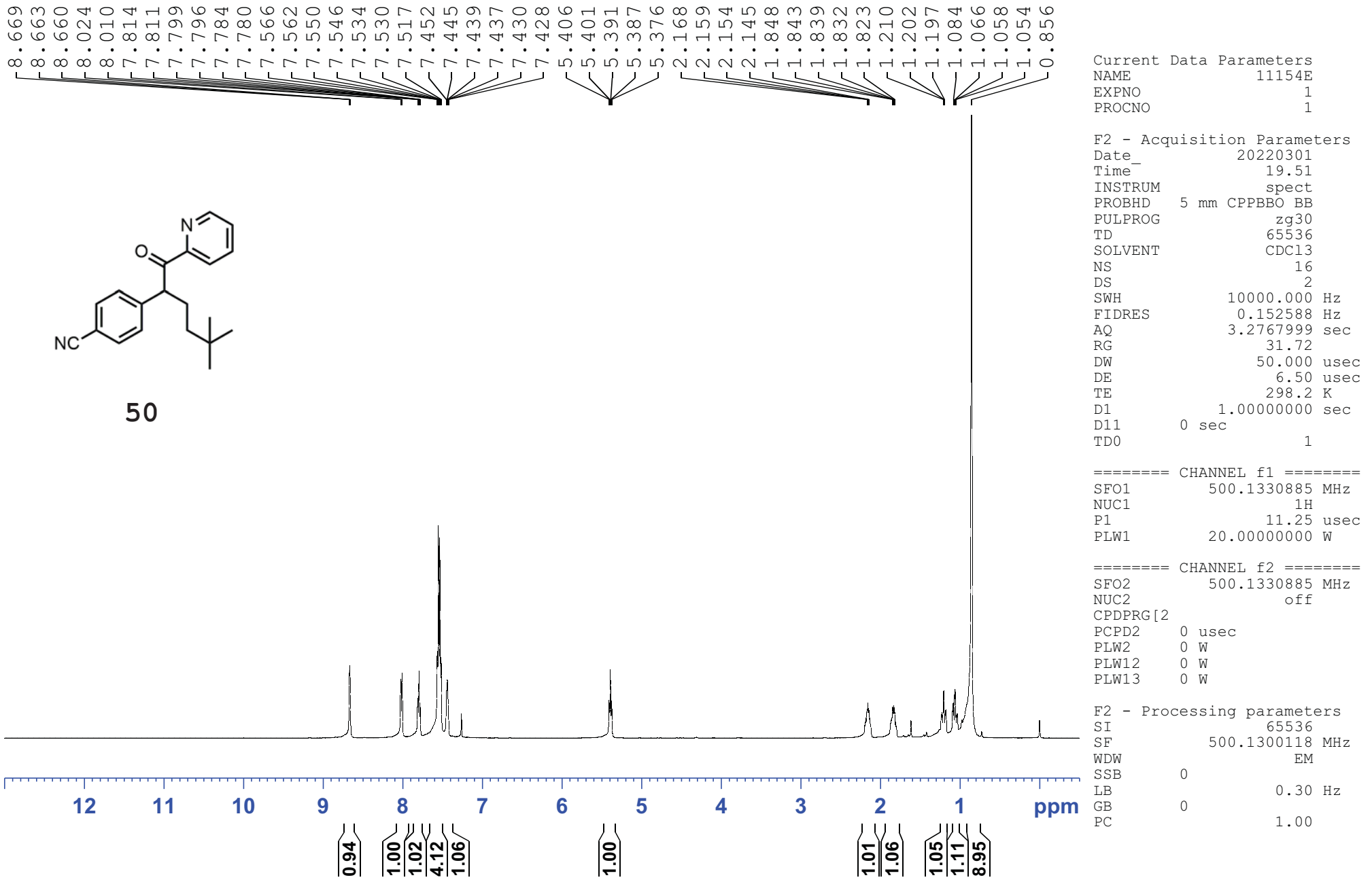


```
Current Data Parameters
NAME                  11154E
EXPNO                      1
PROCNO                     1

F2 - Acquisition Parameters
Date_              20220301
Time                  19.51
INSTRUM               spect
PROBHD   5 mm CPPBBO BB
PULPROG                zg30
TD                    65536
SOLVENT               CDCl3
NS                       16
DS                        2
SWH              10000.000 Hz
FIDRES            0.152588 Hz
AQ               3.2767999 sec
RG                    31.72
DW                  50.000 usec
DE                   6.50 usec
TE                   298.2 K
D1             1.00000000 sec
D11       0 sec
TD0                       1

======== CHANNEL f1 ========
SFO1          500.1330885 MHz
NUC1                     1H
P1                   11.25 usec
PLW1          20.00000000 W

======== CHANNEL f2 ========
SFO2          500.1330885 MHz
NUC2                   off
CPDPRG[2
PCPD2      0 usec
PLW2       0 W
PLW12      0 W
PLW13      0 W

F2 - Processing parameters
SI                    65536
SF          500.1300118 MHz
WDW                      EM
SSB        0
LB                  0.30 Hz
GB         0
PC                  1.00
```

Supplementary Figure 95. ^{1}H-NMR of compound **50**, recorded at 500 MHz and 25 °C in $CDCl_3$.

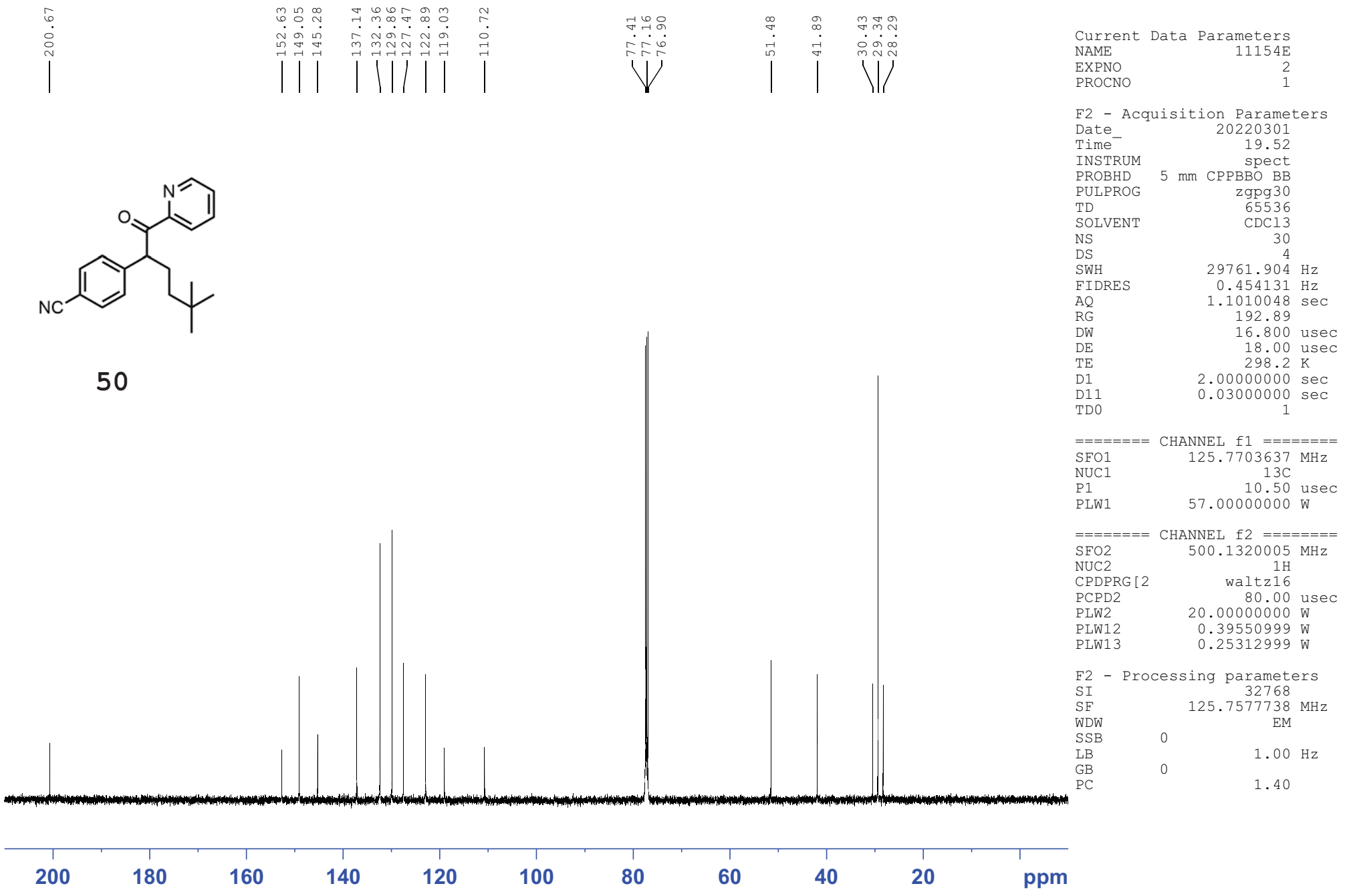


```
Current Data Parameters
NAME                 11154E
EXPNO                     2
PROCNO                    1

F2 - Acquisition Parameters
Date_              20220301
Time                 19.52
INSTRUM              spect
PROBHD   5 mm CPPBBO BB
PULPROG             zgpg30
TD                   65536
SOLVENT              CDCl3
NS                      30
DS                       4
SWH              29761.904 Hz
FIDRES            0.454131 Hz
AQ                1.1010048 sec
RG                  192.89
DW                  16.800 usec
DE                  18.00 usec
TE                  298.2 K
D1              2.00000000 sec
D11             0.03000000 sec
TD0                      1

======== CHANNEL f1 ========
SFO1           125.7703637 MHz
NUC1                   13C
P1                  10.50 usec
PLW1           57.00000000 W

======== CHANNEL f2 ========
SFO2           500.1320005 MHz
NUC2                   1H
CPDPRG[2           waltz16
PCPD2               80.00 usec
PLW2           20.00000000 W
PLW12           0.39550999 W
PLW13           0.25312999 W

F2 - Processing parameters
SI                    32768
SF             125.7577738 MHz
WDW                      EM
SSB       0
LB                  1.00 Hz
GB        0
PC                  1.40
```

Supplementary Figure 96. ^{13}C-NMR of compound **50**, recorded at 126 MHz and 25 °C in $CDCl_3$.

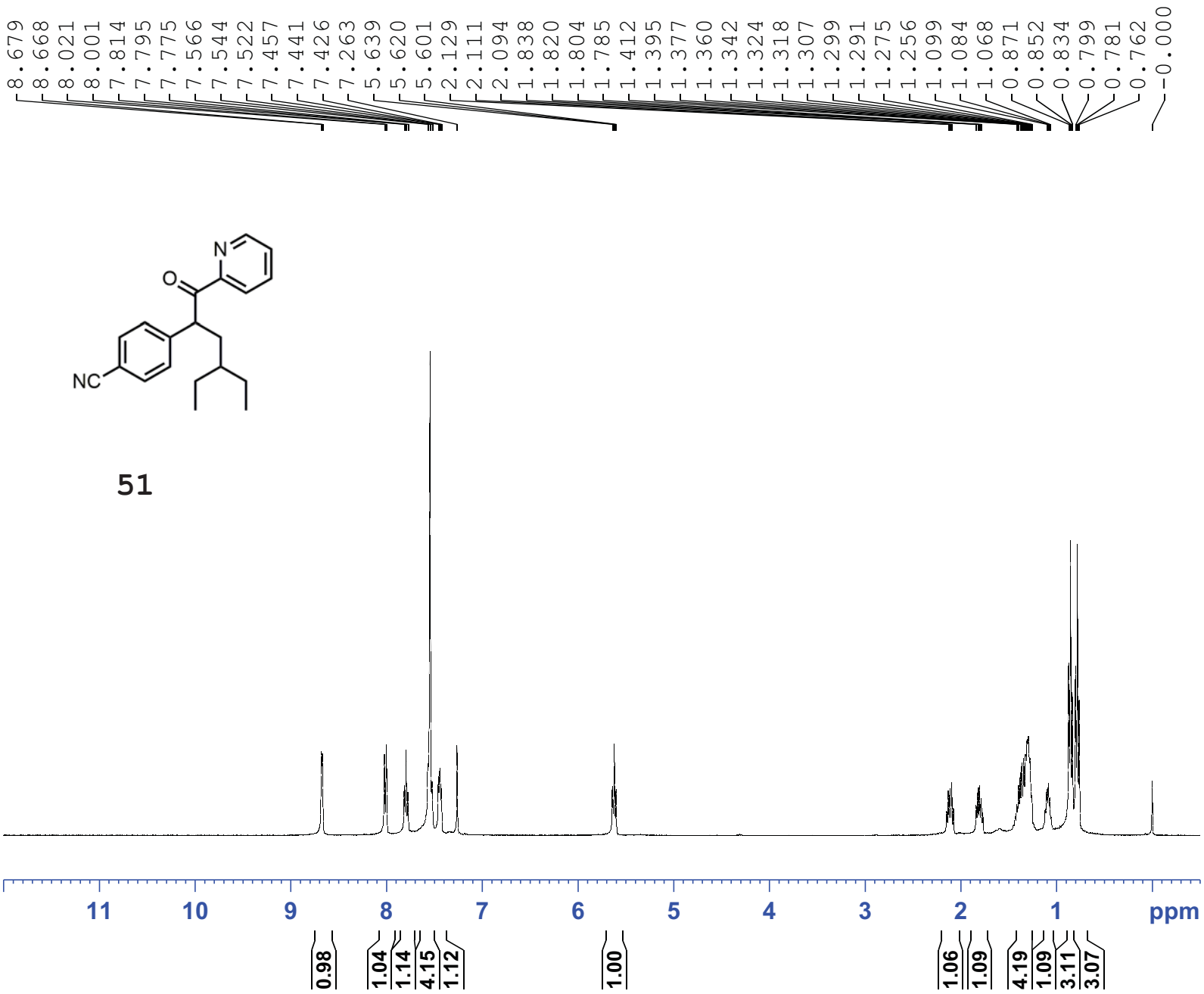


```
Current Data Parameters
NAME                  11158D
EXPNO                      1
PROCNO                     1

F2 - Acquisition Parameters
Date_              20220304
Time                  15.08
INSTRUM               spect
PROBHD    5 mm PABBO BB/
PULPROG                zg30
TD                    32768
SOLVENT               CDCl3
NS                       10
DS                        0
SWH               8012.820 Hz
FIDRES            0.244532 Hz
AQ               2.0447233 sec
RG                   206.33
DW                   62.400 usec
DE                    6.50 usec
TE                   298.1 K
D1             2.00000000 sec
D11       0 sec
TD0                       1

======== CHANNEL f1 ========
SFO1          400.2424716 MHz
NUC1                     1H
P1                   14.30 usec
PLW1          12.00000000 W

======== CHANNEL f2 ========
SFO2          400.2424716 MHz
NUC2                   off
CPDPRG[2
PCPD2      0 usec
PLW2       0 W
PLW12      0 W
PLW13      0 W

F2 - Processing parameters
SI                    65536
SF            400.2400092 MHz
WDW                     EM
SSB        0
LB                   0.30 Hz
GB         0
PC                   1.00
```

Supplementary Figure 97. ^{1}H-NMR of compound **51**, recorded at 400 MHz and 25 °C in $CDCl_3$.

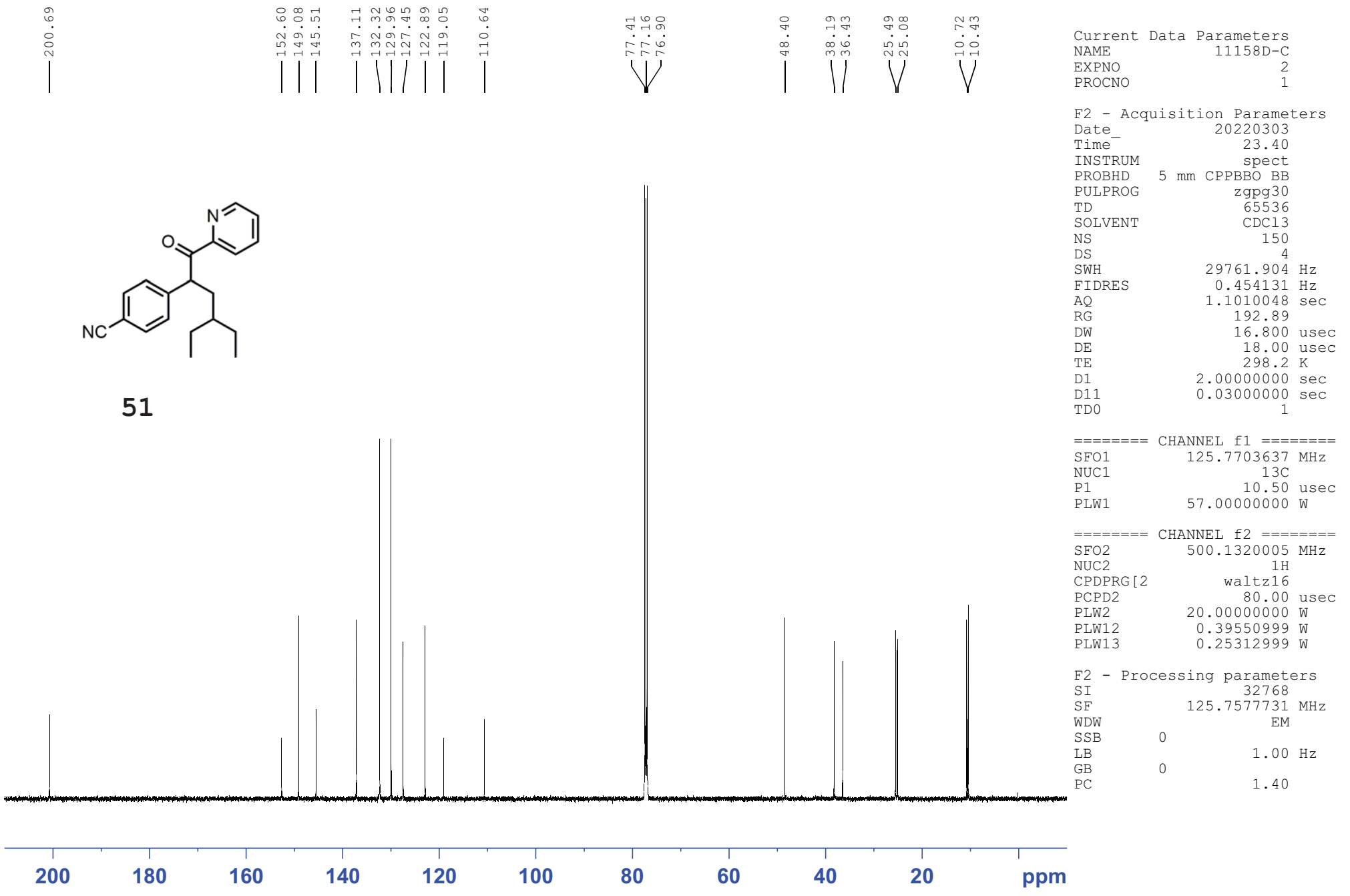


```
Current Data Parameters
NAME            11158D-C
EXPNO                  2
PROCNO                 1

F2 - Acquisition Parameters
Date_           20220303
Time              23.40
INSTRUM           spect
PROBHD   5 mm CPPBBO BB
PULPROG          zgpg30
TD                65536
SOLVENT           CDCl3
NS                  150
DS                    4
SWH           29761.904 Hz
FIDRES         0.454131 Hz
AQ             1.1010048 sec
RG                192.89
DW                16.800 usec
DE                18.00 usec
TE                298.2 K
D1           2.00000000 sec
D11          0.03000000 sec
TD0                   1

======== CHANNEL f1 ========
SFO1         125.7703637 MHz
NUC1                 13C
P1                10.50 usec
PLW1         57.00000000 W

======== CHANNEL f2 ========
SFO2         500.1320005 MHz
NUC2                 1H
CPDPRG[2          waltz16
PCPD2             80.00 usec
PLW2         20.00000000 W
PLW12         0.39550999 W
PLW13         0.25312999 W

F2 - Processing parameters
SI                 32768
SF           125.7577731 MHz
WDW                  EM
SSB       0
LB                1.00 Hz
GB        0
PC                1.40
```

Supplementary Figure 98. ^{13}C-NMR of compound **51**, recorded at 126 MHz and 25 °C in $CDCl_3$.

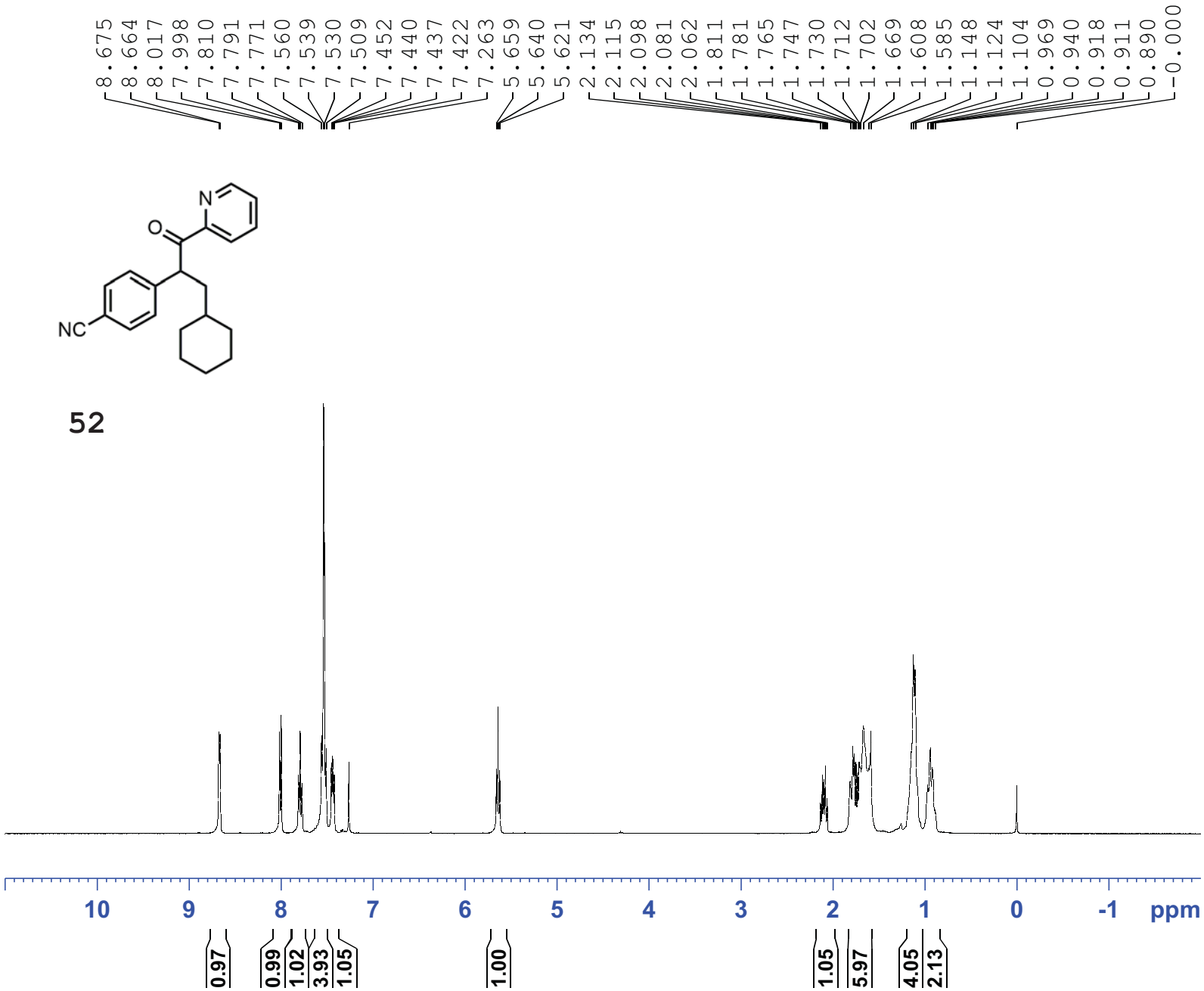


```
Current Data Parameters
NAME                  11150B
EXPNO                      3
PROCNO                     1

F2 - Acquisition Parameters
Date_              20220227
Time                  18.44
INSTRUM               spect
PROBHD    5 mm PABBO BB/
PULPROG                zg30
TD                    32768
SOLVENT               CDCl3
NS                       16
DS                        0
SWH               8012.820 Hz
FIDRES            0.244532 Hz
AQ               2.0447233 sec
RG                   206.33
DW                   62.400 usec
DE                    6.50 usec
TE                   299.1 K
D1             2.00000000 sec
D11       0 sec
TD0                       1

======== CHANNEL f1 ========
SFO1           400.2424716 MHz
NUC1                      1H
P1                   14.30 usec
PLW1           12.00000000 W

======== CHANNEL f2 ========
SFO2           400.2424716 MHz
NUC2                    off
CPDPRG[2
PCPD2      0 usec
PLW2       0 W
PLW12      0 W
PLW13      0 W

F2 - Processing parameters
SI                    65536
SF           400.2400089 MHz
WDW                      EM
SSB        0
LB                    0.30 Hz
GB         0
PC                    1.00
```

Supplementary Figure 99. ^{1}H-NMR of compound **52**, recorded at 400 MHz and 25 °C in $CDCl_3$.

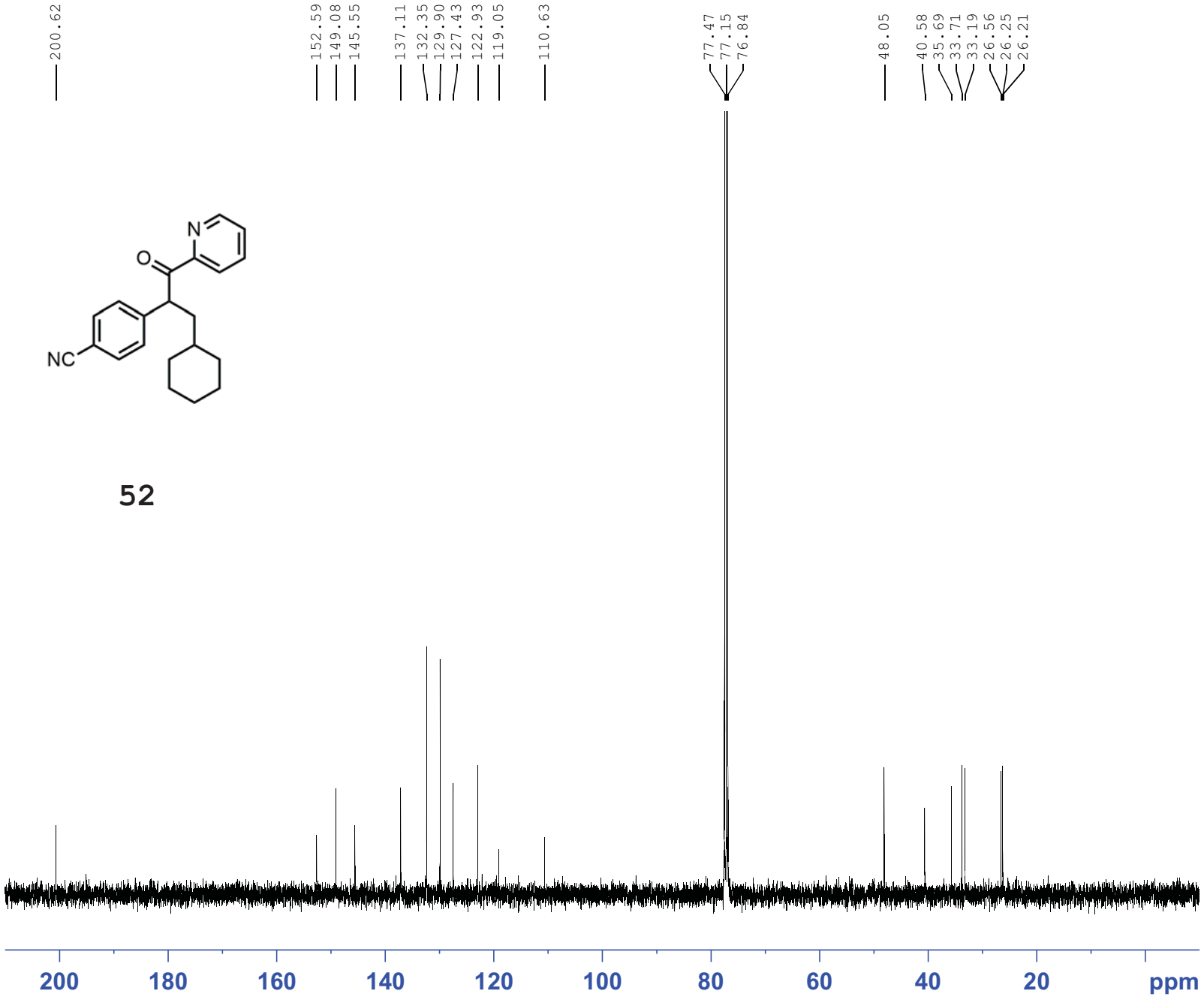


```
Current Data Parameters
NAME                 11150B
EXPNO                     4
PROCNO                    1

F2 - Acquisition Parameters
Date_              20220227
Time                 18.46
INSTRUM              spect
PROBHD    5 mm PABBO BB/
PULPROG             zgpg30
TD                   65536
SOLVENT              CDCl3
NS                     210
DS                       4
SWH             24038.461 Hz
FIDRES           0.366798 Hz
AQ               1.3631488 sec
RG                  206.33
DW                  20.800 usec
DE                   6.50 usec
TE                  299.3 K
D1              2.00000000 sec
D11             0.03000000 sec
TD0                      1

======== CHANNEL f1 ========
SFO1           100.6504916 MHz
NUC1                   13C
P1                  10.00 usec
PLW1           54.00000000 W

======== CHANNEL f2 ========
SFO2           400.2416010 MHz
NUC2                   1H
CPDPRG[2           waltz16
PCPD2               90.00 usec
PLW2           12.00000000 W
PLW12           0.30294999 W
PLW13           0.24539000 W

F2 - Processing parameters
SI                    32768
SF             100.6404153 MHz
WDW                      EM
SSB          0
LB                    1.00 Hz
GB           0
PC                    1.40
```

Supplementary Figure 100. ^{13}C-NMR of compound **52**, recorded at 101 MHz and 25 °C in $CDCl_3$.

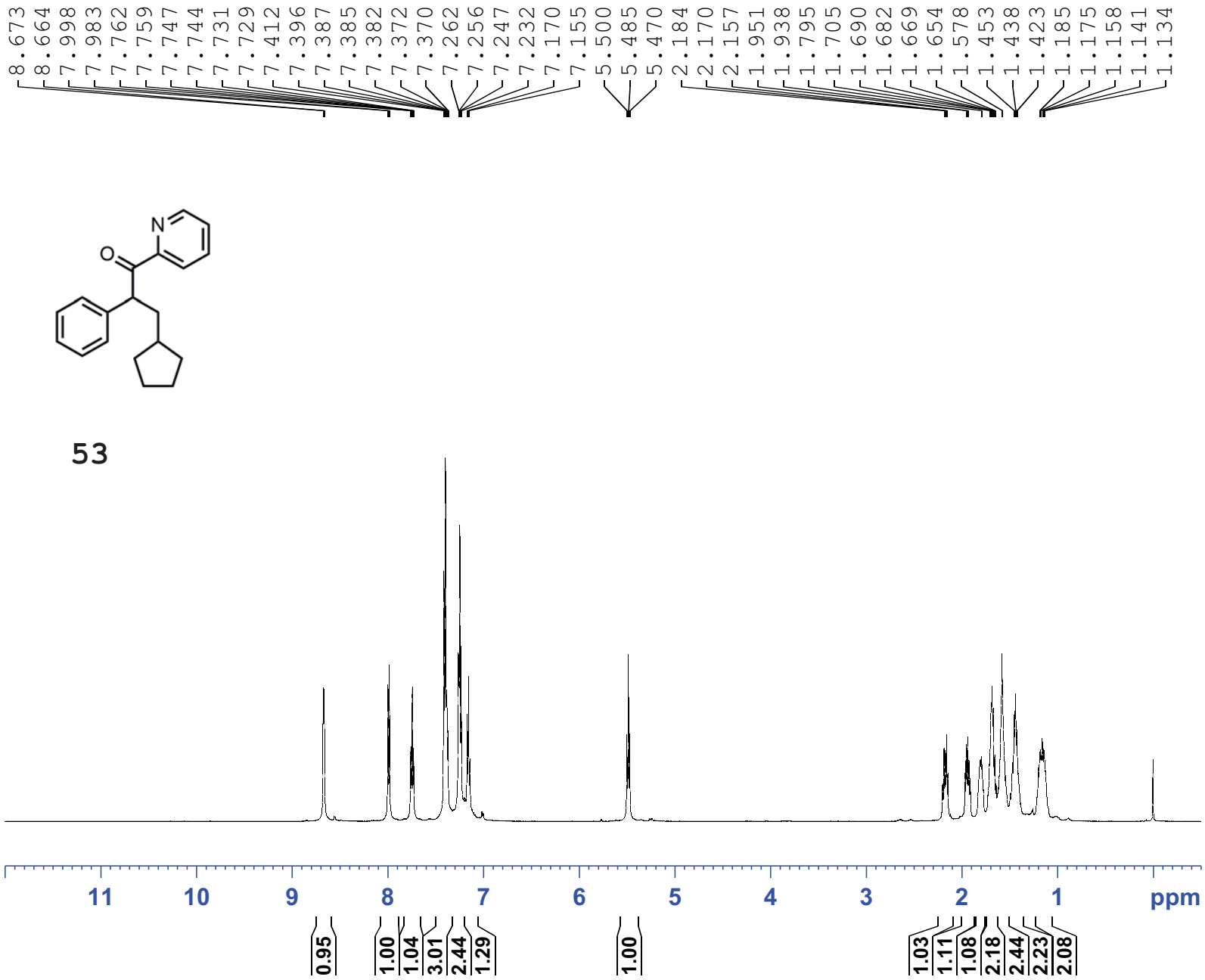


```
Current Data Parameters
NAME                  11136H
EXPNO                      1
PROCNO                     1

F2 - Acquisition Parameters
Date_              20220220
Time                  17.52
INSTRUM               spect
PROBHD    5 mm CPPBBO BB
PULPROG                zg30
TD                    65536
SOLVENT               CDCl3
NS                       16
DS                        2
SWH              10000.000 Hz
FIDRES            0.152588 Hz
AQ               3.2767999 sec
RG                    31.72
DW                  50.000 usec
DE                   6.50 usec
TE                  298.2 K
D1              1.00000000 sec
D11       0 sec
TD0                       1

======== CHANNEL f1 ========
SFO1           500.1330885 MHz
NUC1                   1H
P1                  11.25 usec
PLW1           20.00000000 W

======== CHANNEL f2 ========
SFO2           500.1330885 MHz
NUC2                  off
CPDPRG[2
PCPD2      0 usec
PLW2       0 W
PLW12      0 W
PLW13      0 W

F2 - Processing parameters
SI                    65536
SF             500.1300145 MHz
WDW                     EM
SSB        0
LB                  0.30 Hz
GB         0
PC                  1.00
```

Supplementary Figure 101. ^{1}H-NMR of compound **53**, recorded at 500 MHz and 25 °C in $CDCl_3$.

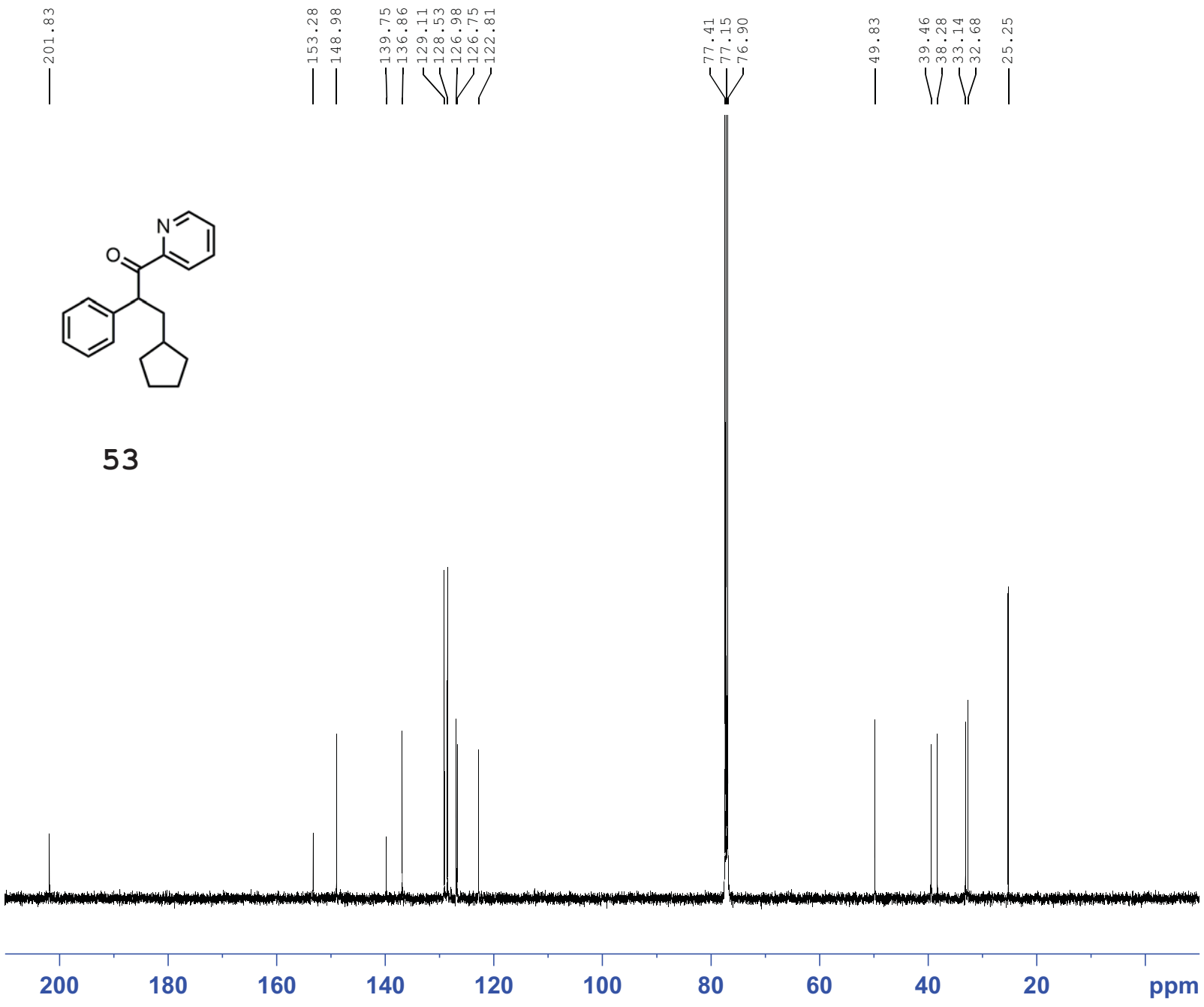


```
Current Data Parameters
NAME                  11136H
EXPNO                      2
PROCNO                     1

F2 - Acquisition Parameters
Date_              20220220
Time                  17.55
INSTRUM               spect
PROBHD   5 mm CPPBBO BB
PULPROG              zgpg30
TD                    65536
SOLVENT               CDCl3
NS                       44
DS                        4
SWH              29761.904 Hz
FIDRES            0.454131 Hz
AQ                1.1010048 sec
RG                   192.89
DW                   16.800 usec
DE                   18.00 usec
TE                   298.2 K
D1              2.00000000 sec
D11             0.03000000 sec
TD0                       1

======== CHANNEL f1 ========
SFO1           125.7703637 MHz
NUC1                    13C
P1                   10.50 usec
PLW1           57.00000000 W

======== CHANNEL f2 ========
SFO2           500.1320005 MHz
NUC2                    1H
CPDPRG[2            waltz16
PCPD2                80.00 usec
PLW2           20.00000000 W
PLW12           0.39550999 W
PLW13           0.25312999 W

F2 - Processing parameters
SI                    32768
SF             125.7577729 MHz
WDW                      EM
SSB          0
LB                    1.00 Hz
GB           0
PC                    1.40
```

Supplementary Figure 102. ^{13}C-NMR of compound **53**, recorded at 126 MHz and 25 °C in $CDCl_3$.

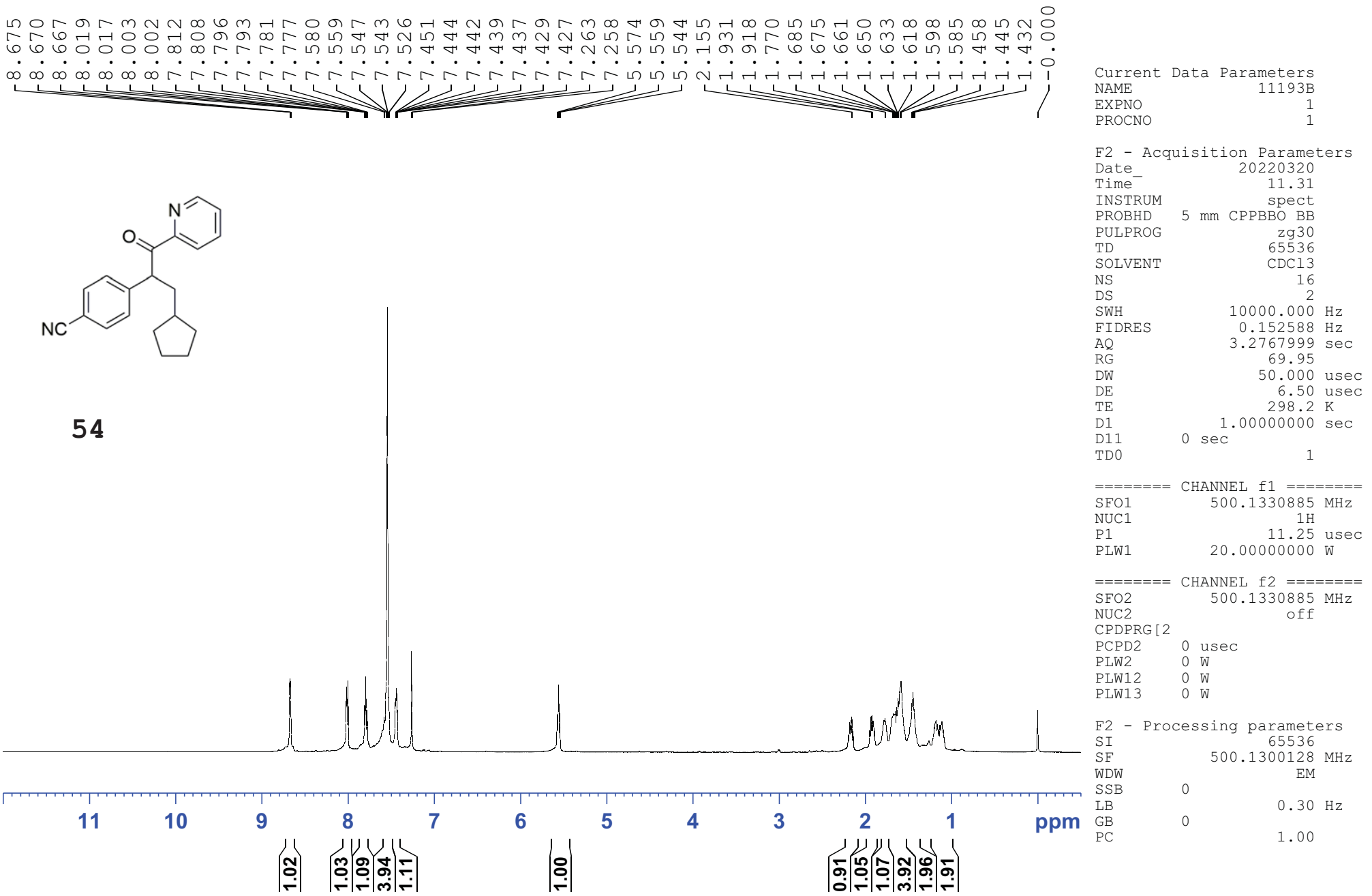


```
Current Data Parameters
NAME                  11193B
EXPNO                      1
PROCNO                     1

F2 - Acquisition Parameters
Date_              20220320
Time                  11.31
INSTRUM               spect
PROBHD   5 mm CPPBBO BB
PULPROG                zg30
TD                    65536
SOLVENT               CDCl3
NS                       16
DS                        2
SWH              10000.000 Hz
FIDRES            0.152588 Hz
AQ               3.2767999 sec
RG                    69.95
DW                  50.000 usec
DE                   6.50 usec
TE                  298.2 K
D1              1.00000000 sec
D11       0 sec
TD0                       1

======== CHANNEL f1 ========
SFO1           500.1330885 MHz
NUC1                      1H
P1                    11.25 usec
PLW1           20.00000000 W

======== CHANNEL f2 ========
SFO2           500.1330885 MHz
NUC2                    off
CPDPRG[2
PCPD2      0 usec
PLW2       0 W
PLW12      0 W
PLW13      0 W

F2 - Processing parameters
SI                    65536
SF           500.1300128 MHz
WDW                     EM
SSB        0
LB                  0.30 Hz
GB         0
PC                  1.00
```

Supplementary Figure 103. ^{1}H-NMR of compound **54**, recorded at 500 MHz and 25 °C in $CDCl_3$.

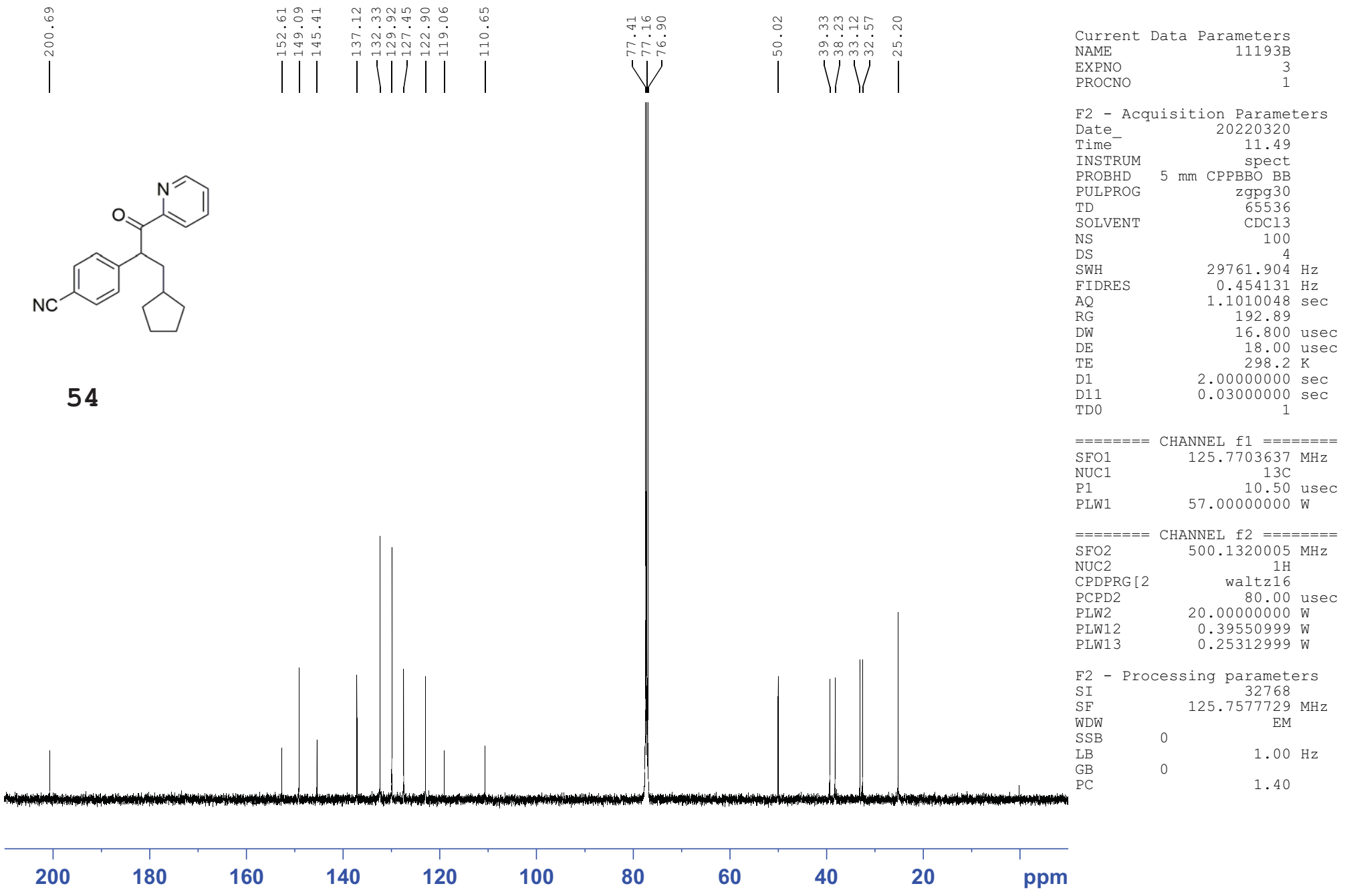


```
Current Data Parameters
NAME                 11193B
EXPNO                     3
PROCNO                    1

F2 - Acquisition Parameters
Date_              20220320
Time                 11.49
INSTRUM              spect
PROBHD   5 mm CPPBBO BB
PULPROG             zgpg30
TD                   65536
SOLVENT              CDCl3
NS                     100
DS                       4
SWH              29761.904 Hz
FIDRES            0.454131 Hz
AQ                1.1010048 sec
RG                   192.89
DW                  16.800 usec
DE                  18.00 usec
TE                  298.2 K
D1              2.00000000 sec
D11             0.03000000 sec
TD0                      1

======== CHANNEL f1 ========
SFO1           125.7703637 MHz
NUC1                    13C
P1                   10.50 usec
PLW1           57.00000000 W

======== CHANNEL f2 ========
SFO2           500.1320005 MHz
NUC2                    1H
CPDPRG[2           waltz16
PCPD2                80.00 usec
PLW2           20.00000000 W
PLW12           0.39550999 W
PLW13           0.25312999 W

F2 - Processing parameters
SI                   32768
SF             125.7577729 MHz
WDW                     EM
SSB       0
LB                    1.00 Hz
GB        0
PC                    1.40
```

Supplementary Figure 104. ^{13}C-NMR of compound **54**, recorded at 126 MHz and 25 °C in $CDCl_3$.

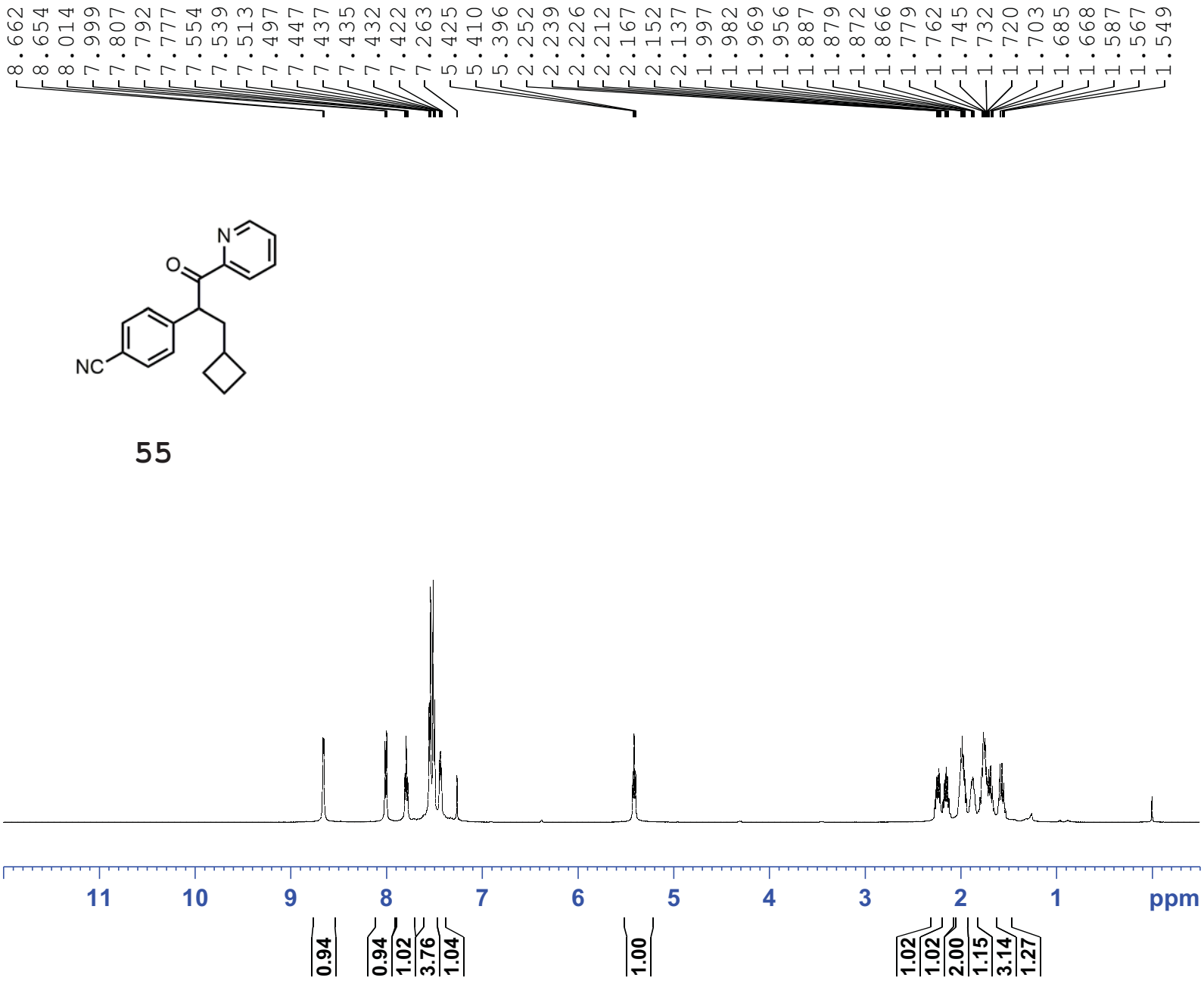


```
Current Data Parameters
NAME                  H-154K
EXPNO                      1
PROCNO                     1

F2 - Acquisition Parameters
Date_              20220301
Time                  20.23
INSTRUM               spect
PROBHD    5 mm CPPBBO BB
PULPROG                zg30
TD                    65536
SOLVENT               CDCl3
NS                       16
DS                        2
SWH              10000.000 Hz
FIDRES            0.152588 Hz
AQ               3.2767999 sec
RG                    49.27
DW                  50.000 usec
DE                   6.50 usec
TE                   298.2 K
D1             1.00000000 sec
D11       0 sec
TD0                       1

======== CHANNEL f1 ========
SFO1           500.1330885 MHz
NUC1                     1H
P1                   11.25 usec
PLW1           20.00000000 W

======== CHANNEL f2 ========
SFO2           500.1330885 MHz
NUC2                    off
CPDPRG[2
PCPD2      0 usec
PLW2       0 W
PLW12      0 W
PLW13      0 W

F2 - Processing parameters
SI                    65536
SF           500.1300114 MHz
WDW                      EM
SSB        0
LB                    0.30 Hz
GB         0
PC                    1.00
```

Supplementary Figure 105. ^{1}H-NMR of compound **55**, recorded at 500 MHz and 25 °C in $CDCl_3$.

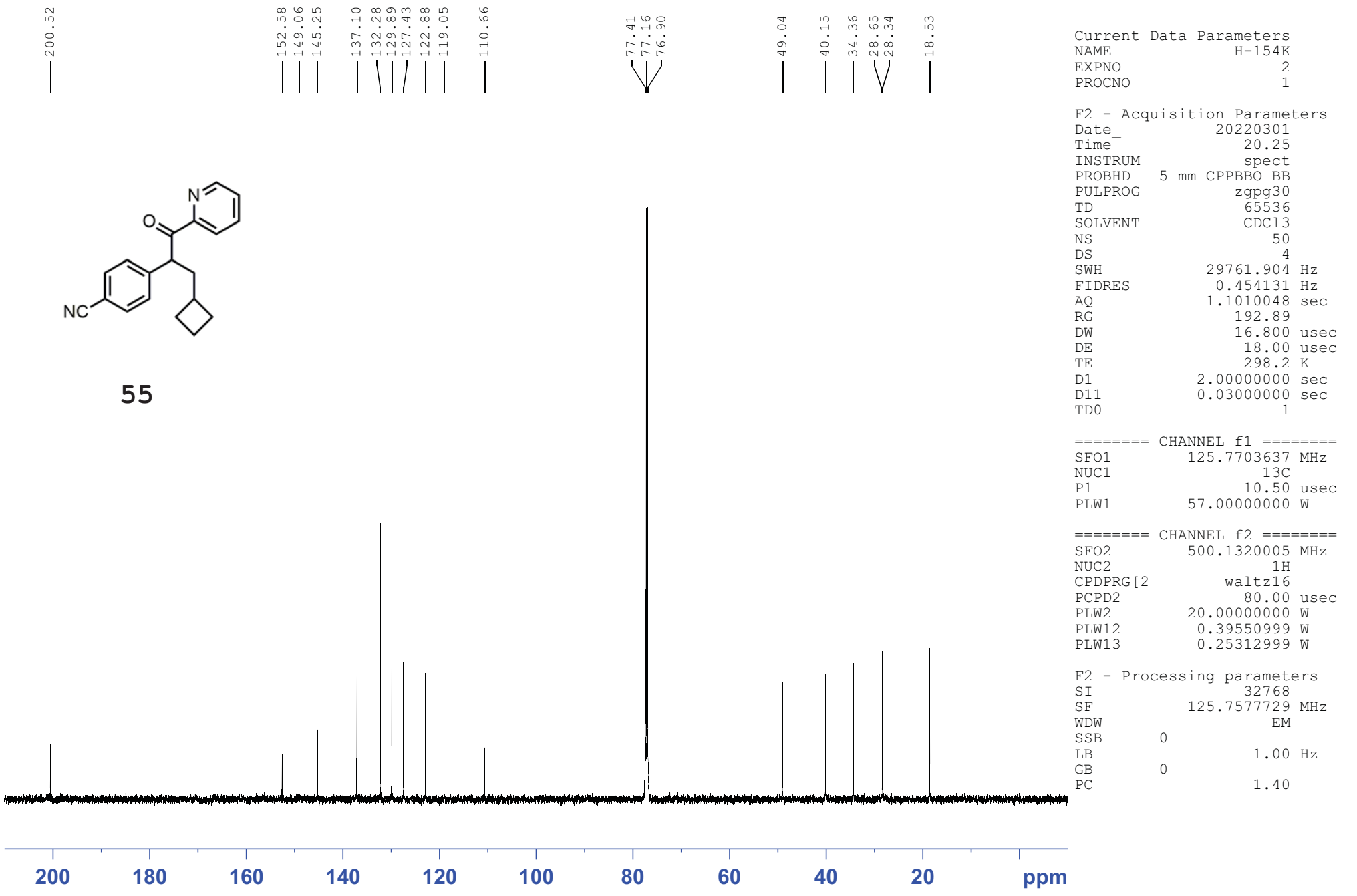


```
Current Data Parameters
NAME                 H-154K
EXPNO                     2
PROCNO                    1

F2 - Acquisition Parameters
Date_              20220301
Time                 20.25
INSTRUM              spect
PROBHD   5 mm CPPBBO BB
PULPROG             zgpg30
TD                   65536
SOLVENT              CDCl3
NS                      50
DS                       4
SWH              29761.904 Hz
FIDRES            0.454131 Hz
AQ                1.1010048 sec
RG                   192.89
DW                  16.800 usec
DE                  18.00 usec
TE                  298.2 K
D1              2.00000000 sec
D11             0.03000000 sec
TD0                       1

======== CHANNEL f1 ========
SFO1           125.7703637 MHz
NUC1                   13C
P1                  10.50 usec
PLW1           57.00000000 W

======== CHANNEL f2 ========
SFO2           500.1320005 MHz
NUC2                   1H
CPDPRG[2           waltz16
PCPD2               80.00 usec
PLW2           20.00000000 W
PLW12           0.39550999 W
PLW13           0.25312999 W

F2 - Processing parameters
SI                    32768
SF             125.7577729 MHz
WDW                      EM
SSB       0
LB                   1.00 Hz
GB        0
PC                   1.40
```

Supplementary Figure 106. ^{13}C-NMR of compound **55**, recorded at 126 MHz and 25 °C in $CDCl_3$.

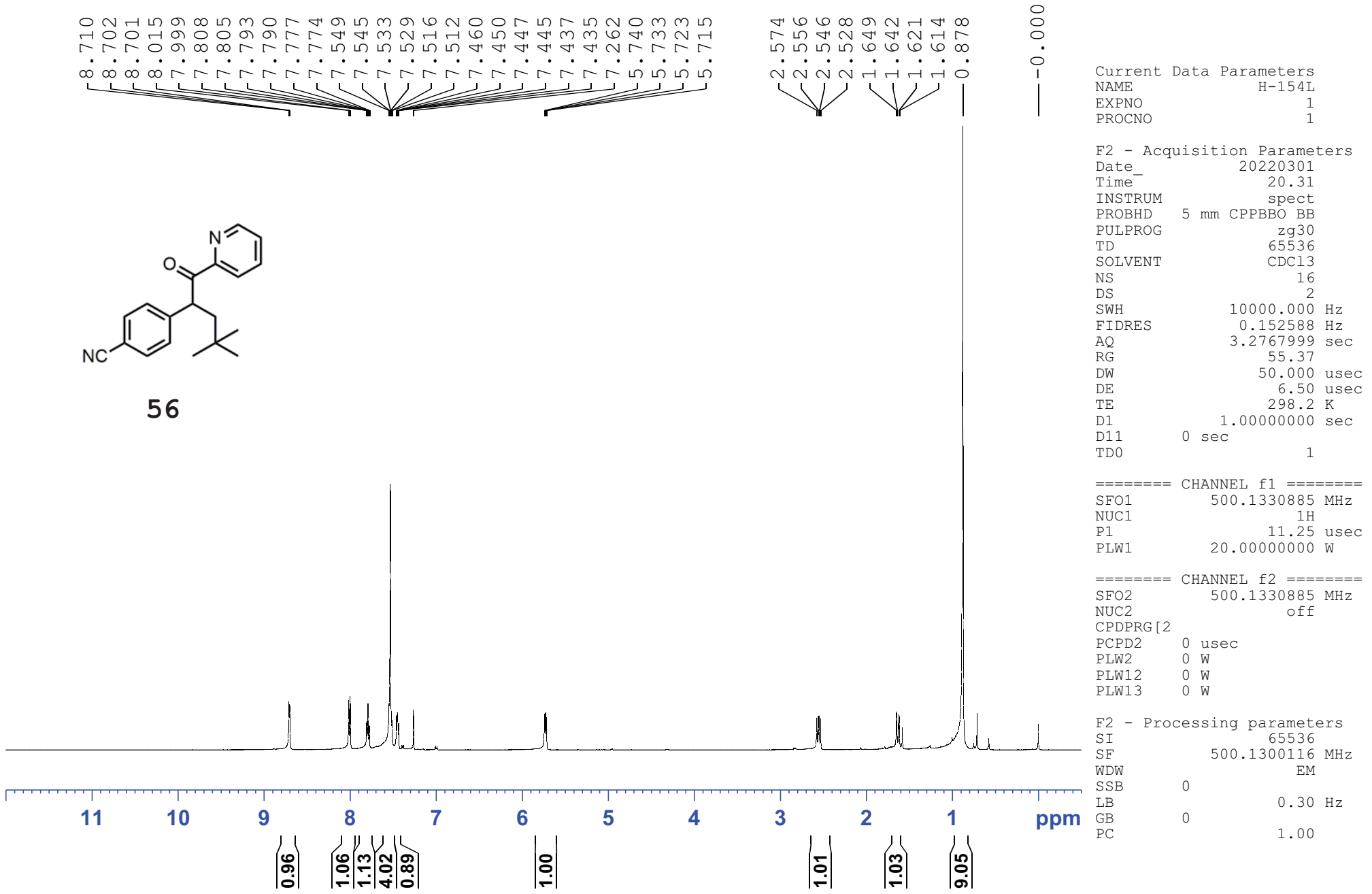


```
Current Data Parameters
NAME                  H-154L
EXPNO                      1
PROCNO                     1

F2 - Acquisition Parameters
Date_              20220301
Time                  20.31
INSTRUM               spect
PROBHD    5 mm CPPBBO BB
PULPROG                zg30
TD                    65536
SOLVENT               CDCl3
NS                       16
DS                        2
SWH              10000.000 Hz
FIDRES               0.152588 Hz
AQ               3.2767999 sec
RG                    55.37
DW                   50.000 usec
DE                    6.50 usec
TE                   298.2 K
D1             1.00000000 sec
D11       0 sec
TD0                       1

======== CHANNEL f1 ========
SFO1          500.1330885 MHz
NUC1                     1H
P1                   11.25 usec
PLW1          20.00000000 W

======== CHANNEL f2 ========
SFO2          500.1330885 MHz
NUC2                   off
CPDPRG[2
PCPD2      0 usec
PLW2       0 W
PLW12      0 W
PLW13      0 W

F2 - Processing parameters
SI                    65536
SF          500.1300116 MHz
WDW                      EM
SSB        0
LB                   0.30 Hz
GB         0
PC                   1.00
```

Supplementary Figure 107. ^{1}H-NMR of compound **56**, recorded at 500 MHz and 25 °C in $CDCl_3$.

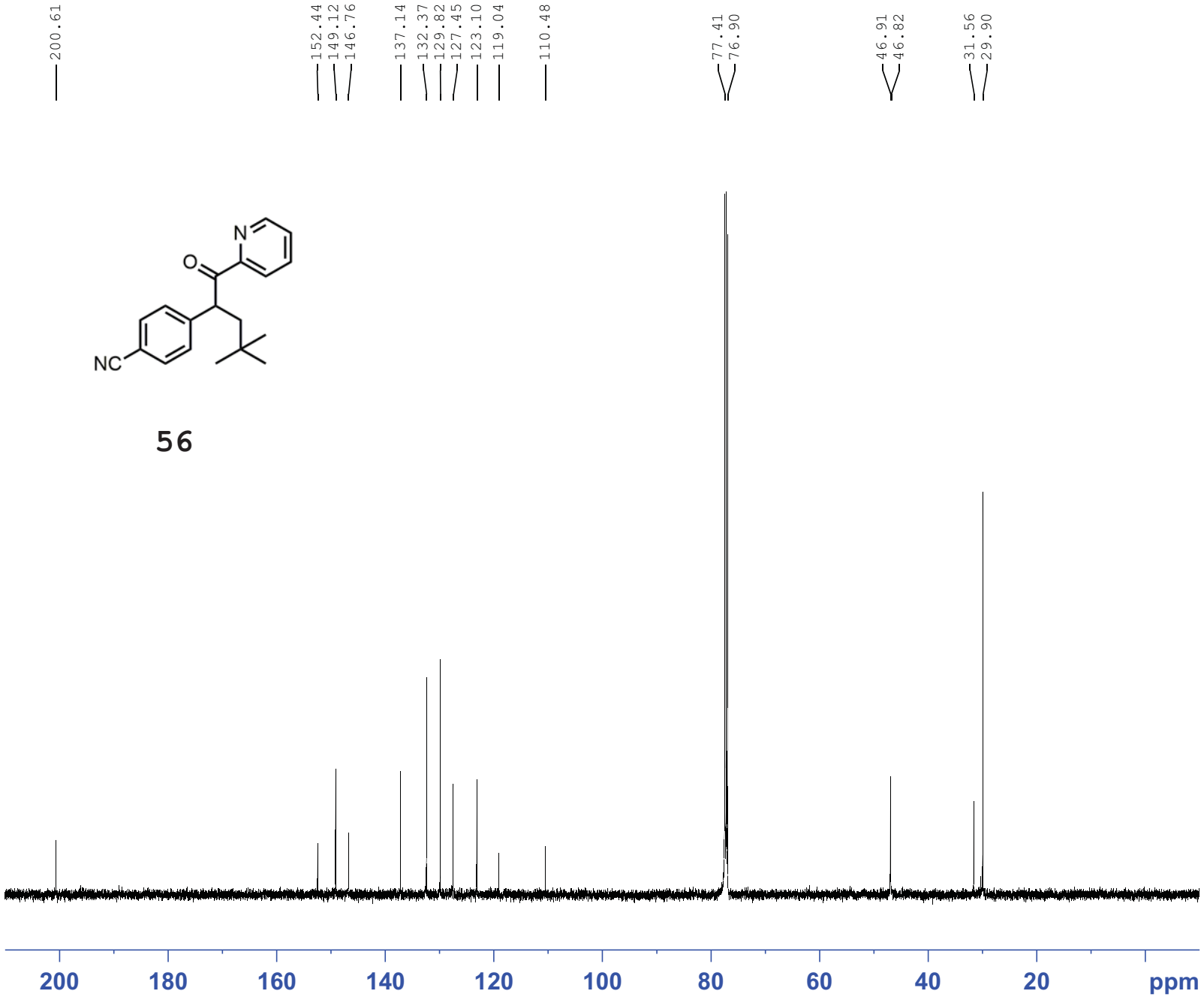


```
Current Data Parameters
NAME                 H-154L
EXPNO                     2
PROCNO                    1

F2 - Acquisition Parameters
Date_              20220301
Time                 20.33
INSTRUM              spect
PROBHD   5 mm CPPBBO BB
PULPROG             zgpg30
TD                   65536
SOLVENT              CDCl3
NS                      50
DS                       4
SWH              29761.904 Hz
FIDRES            0.454131 Hz
AQ                1.1010048 sec
RG                   192.89
DW                  16.800 usec
DE                  18.00 usec
TE                  298.2 K
D1              2.00000000 sec
D11             0.03000000 sec
TD0                       1

======== CHANNEL f1 ========
SFO1           125.7703637 MHz
NUC1                    13C
P1                   10.50 usec
PLW1           57.00000000 W

======== CHANNEL f2 ========
SFO2           500.1320005 MHz
NUC2                    1H
CPDPRG[2           waltz16
PCPD2                80.00 usec
PLW2           20.00000000 W
PLW12           0.39550999 W
PLW13           0.25312999 W

F2 - Processing parameters
SI                    32768
SF             125.7577729 MHz
WDW                      EM
SSB       0
LB                    1.00 Hz
GB        0
PC                    1.40
```

Supplementary Figure 108. ^{13}C-NMR of compound **56**, recorded at 126 MHz and 25 °C in $CDCl_3$.

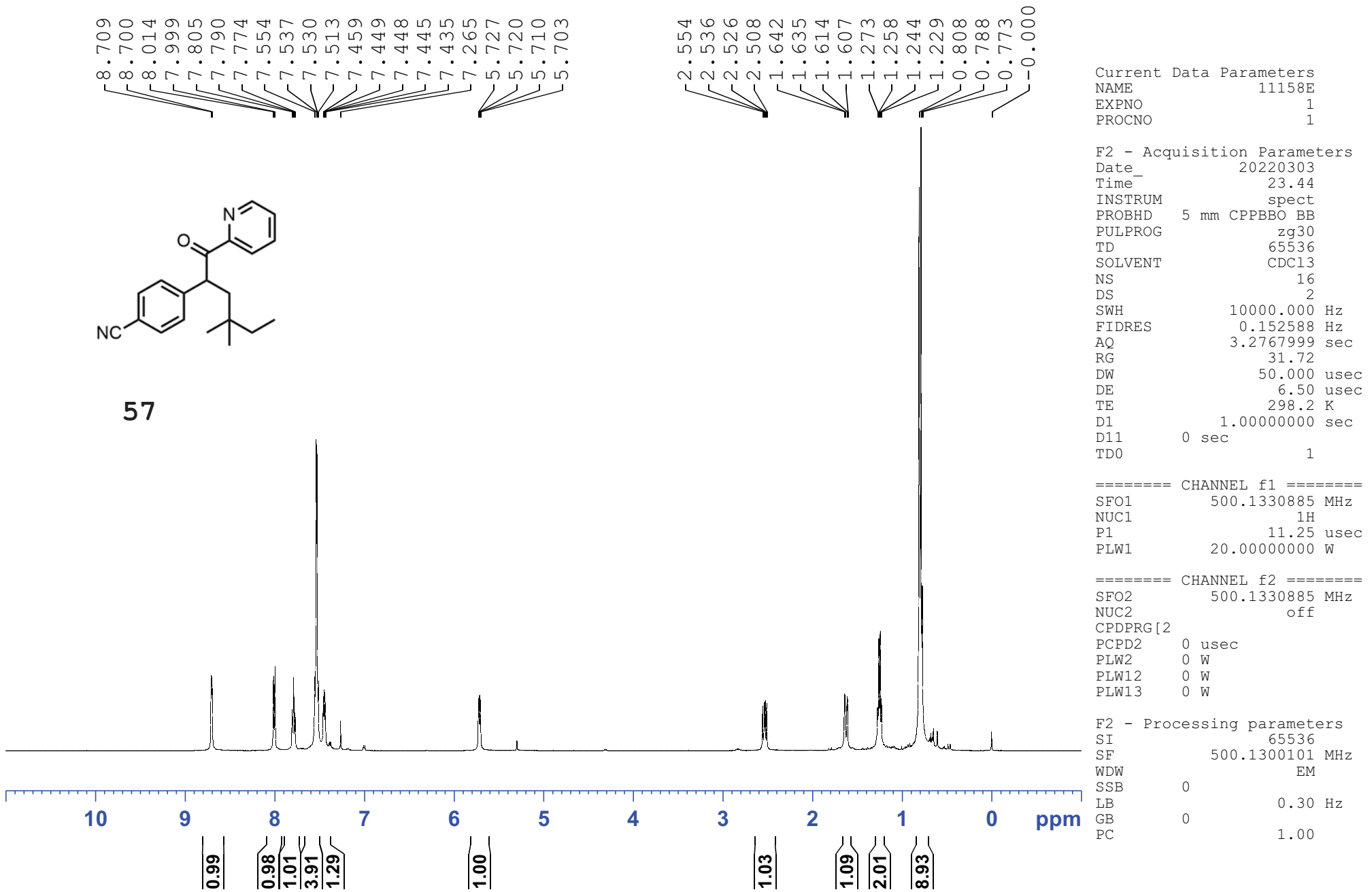


```
Current Data Parameters
NAME                  11158E
EXPNO                      1
PROCNO                     1

F2 - Acquisition Parameters
Date_              20220303
Time                  23.44
INSTRUM               spect
PROBHD   5 mm CPPBBO BB
PULPROG                zg30
TD                    65536
SOLVENT               CDCl3
NS                       16
DS                        2
SWH              10000.000 Hz
FIDRES            0.152588 Hz
AQ               3.2767999 sec
RG                    31.72
DW                  50.000 usec
DE                   6.50 usec
TE                   298.2 K
D1             1.00000000 sec
D11       0 sec
TD0                       1

======== CHANNEL f1 ========
SFO1           500.1330885 MHz
NUC1                     1H
P1                   11.25 usec
PLW1           20.00000000 W

======== CHANNEL f2 ========
SFO2           500.1330885 MHz
NUC2                   off
CPDPRG[2
PCPD2      0 usec
PLW2       0 W
PLW12      0 W
PLW13      0 W

F2 - Processing parameters
SI                    65536
SF           500.1300101 MHz
WDW                      EM
SSB        0
LB                   0.30 Hz
GB         0
PC                    1.00
```

Supplementary Figure 109. ^{1}H-NMR of compound **57**, recorded at 500 MHz and 25 °C in $CDCl_3$.

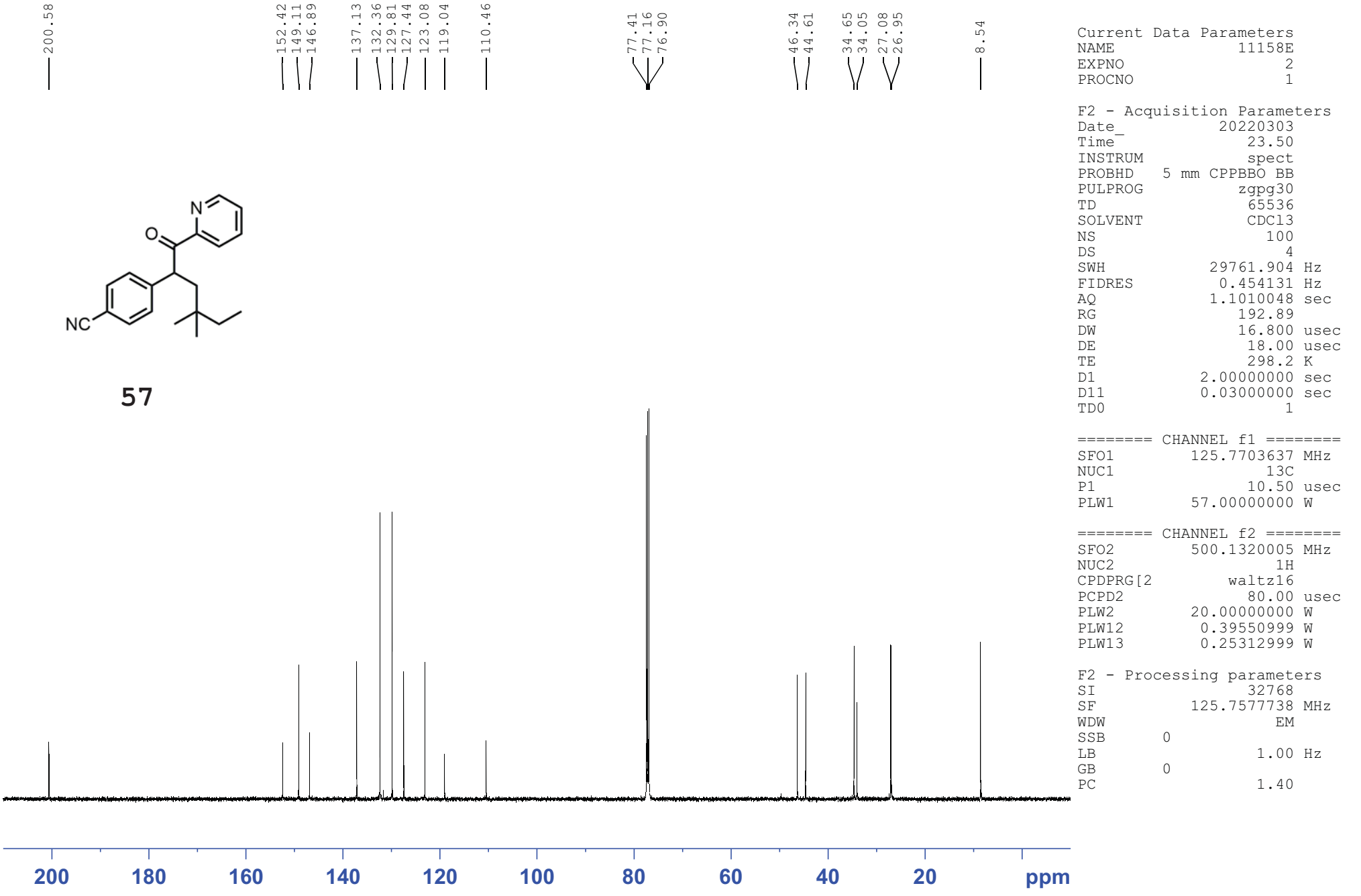


```
Current Data Parameters
NAME                 11158E
EXPNO                     2
PROCNO                    1

F2 - Acquisition Parameters
Date_              20220303
Time                 23.50
INSTRUM              spect
PROBHD   5 mm CPPBBO BB
PULPROG             zgpg30
TD                   65536
SOLVENT              CDCl3
NS                     100
DS                       4
SWH              29761.904 Hz
FIDRES            0.454131 Hz
AQ                1.1010048 sec
RG                   192.89
DW                  16.800 usec
DE                  18.00 usec
TE                  298.2 K
D1              2.00000000 sec
D11             0.03000000 sec
TD0                      1

======== CHANNEL f1 ========
SFO1           125.7703637 MHz
NUC1                   13C
P1                  10.50 usec
PLW1           57.00000000 W

======== CHANNEL f2 ========
SFO2           500.1320005 MHz
NUC2                   1H
CPDPRG[2           waltz16
PCPD2               80.00 usec
PLW2           20.00000000 W
PLW12           0.39550999 W
PLW13           0.25312999 W

F2 - Processing parameters
SI                    32768
SF             125.7577738 MHz
WDW                      EM
SSB       0
LB                   1.00 Hz
GB        0
PC                   1.40
```

Supplementary Figure 110. ^{13}C-NMR of compound **57**, recorded at 126 MHz and 25 °C in $CDCl_3$.

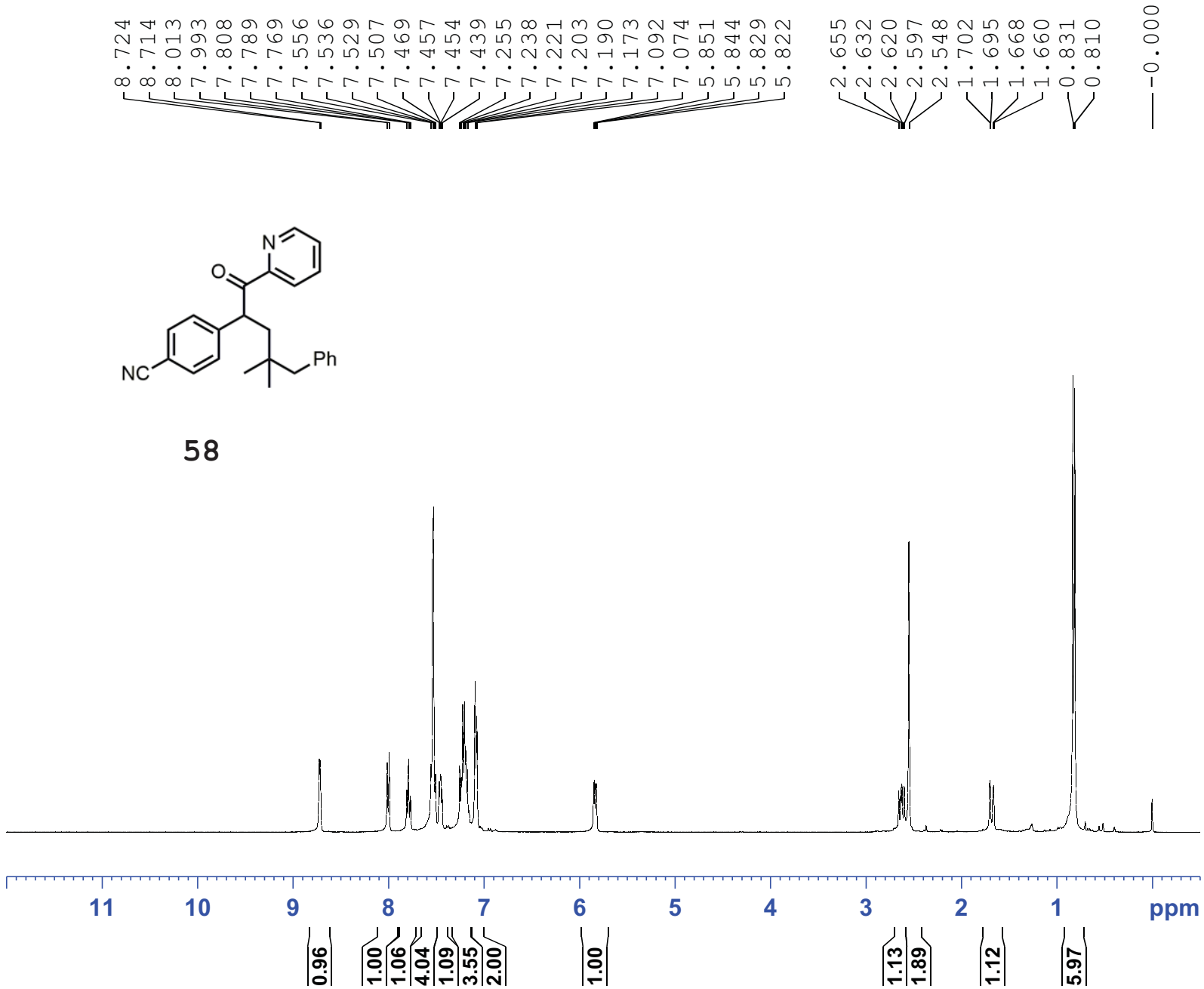


```
Current Data Parameters
NAME                  11158G
EXPNO                      1
PROCNO                     1

F2 - Acquisition Parameters
Date_              20220304
Time                  15.04
INSTRUM               spect
PROBHD    5 mm PABBO BB/
PULPROG                zg30
TD                    32768
SOLVENT               CDCl3
NS                       14
DS                        0
SWH               8012.820 Hz
FIDRES            0.244532 Hz
AQ                2.0447233 sec
RG                   140.59
DW                   62.400 usec
DE                    6.50 usec
TE                   298.1 K
D1             2.00000000 sec
D11       0 sec
TD0                       1

======== CHANNEL f1 ========
SFO1           400.2424716 MHz
NUC1                     1H
P1                   14.30 usec
PLW1           12.00000000 W

======== CHANNEL f2 ========
SFO2           400.2424716 MHz
NUC2                    off
CPDPRG[2
PCPD2      0 usec
PLW2       0 W
PLW12      0 W
PLW13      0 W

F2 - Processing parameters
SI                    65536
SF           400.2400121 MHz
WDW                      EM
SSB        0
LB                   0.30 Hz
GB         0
PC                   1.00
```

Supplementary Figure 111. ^{1}H-NMR of compound **58**, recorded at 400 MHz and 25 °C in $CDCl_3$.

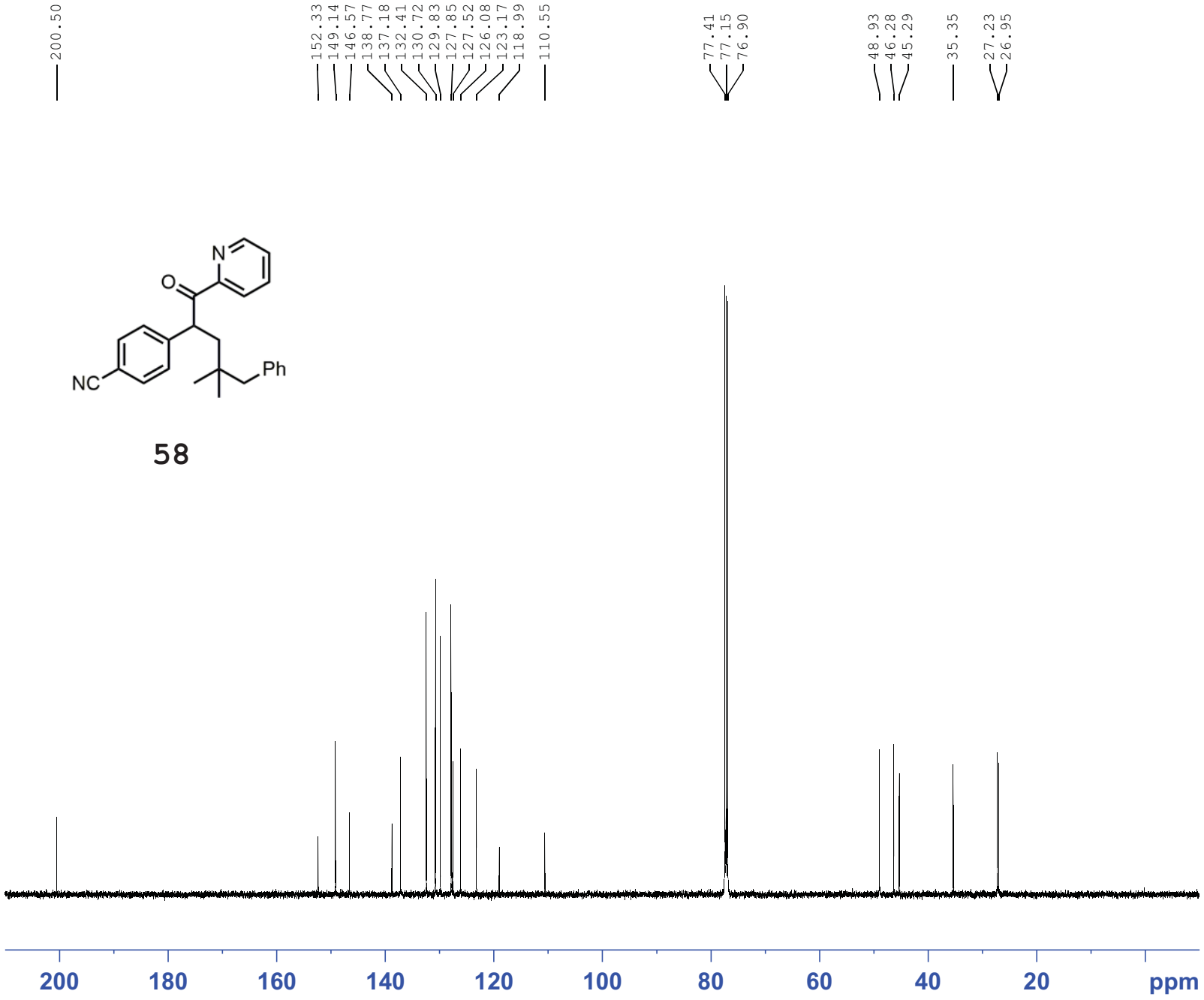


```
Current Data Parameters
NAME            11158G-C
EXPNO                  2
PROCNO                 1

F2 - Acquisition Parameters
Date_           20220303
Time              23.59
INSTRUM           spect
PROBHD   5 mm CPPBBO BB
PULPROG          zgpg30
TD                65536
SOLVENT           CDCl3
NS                   80
DS                    4
SWH           29761.904 Hz
FIDRES         0.454131 Hz
AQ             1.1010048 sec
RG               192.89
DW               16.800 usec
DE               18.00 usec
TE               298.2 K
D1          2.00000000 sec
D11         0.03000000 sec
TD0                   1

======== CHANNEL f1 ========
SFO1        125.7703637 MHz
NUC1                13C
P1                10.50 usec
PLW1        57.00000000 W

======== CHANNEL f2 ========
SFO2        500.1320005 MHz
NUC2                1H
CPDPRG[2         waltz16
PCPD2             80.00 usec
PLW2        20.00000000 W
PLW12        0.39550999 W
PLW13        0.25312999 W

F2 - Processing parameters
SI                 32768
SF          125.7577745 MHz
WDW                  EM
SSB       0
LB                1.00 Hz
GB        0
PC                1.40
```

Supplementary Figure 112. ^{13}C-NMR of compound **58**, recorded at 126 MHz and 25 °C in $CDCl_3$.

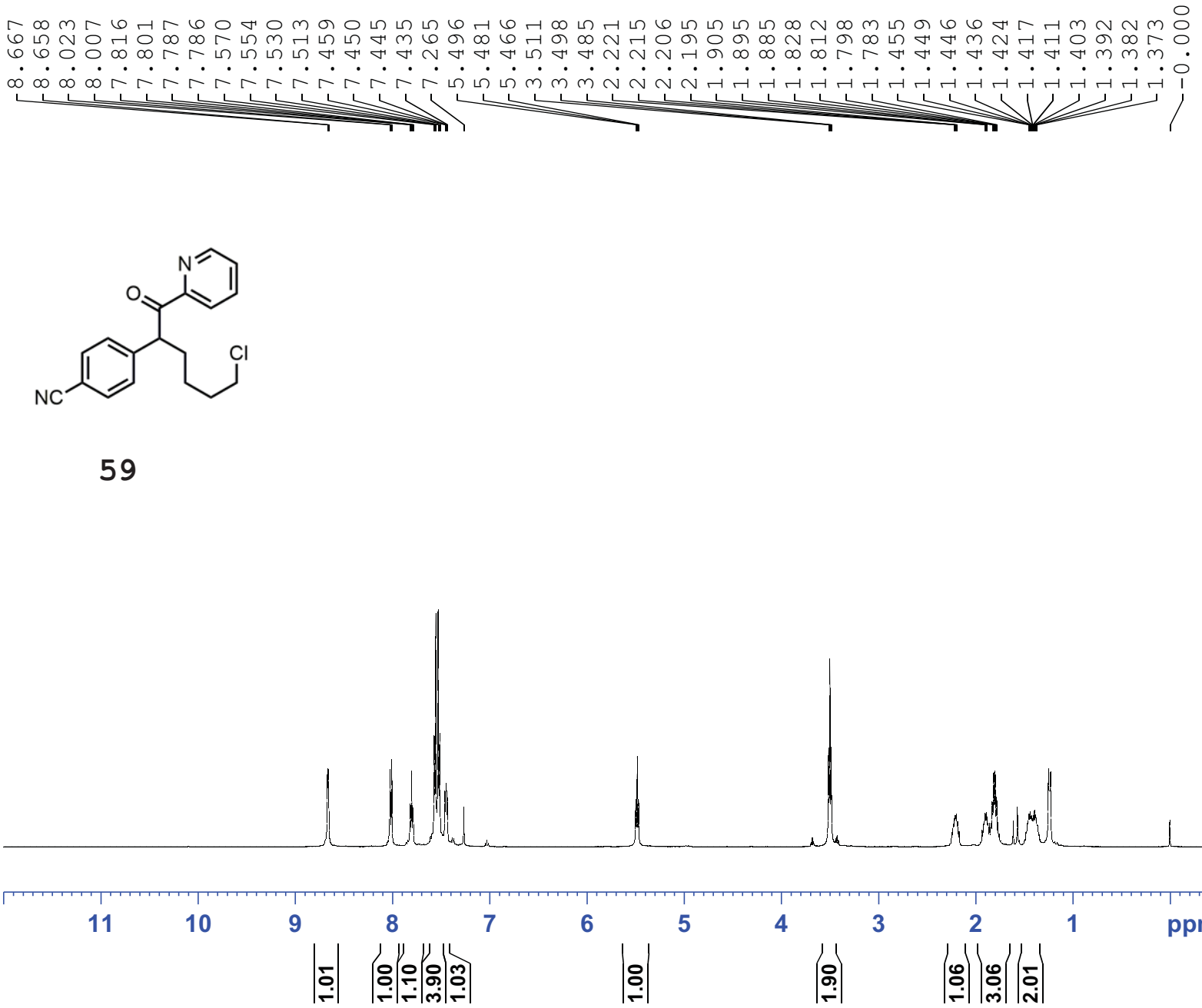


```
Current Data Parameters
NAME                  H-154J
EXPNO                      1
PROCNO                     1

F2 - Acquisition Parameters
Date_              20220301
Time                  20.09
INSTRUM               spect
PROBHD    5 mm CPPBBO BB
PULPROG                zg30
TD                    65536
SOLVENT               CDCl3
NS                       16
DS                        2
SWH              10000.000 Hz
FIDRES               0.152588 Hz
AQ                3.2767999 sec
RG                    31.72
DW                   50.000 usec
DE                    6.50 usec
TE                   298.2 K
D1              1.00000000 sec
D11       0 sec
TD0                       1

======== CHANNEL f1 ========
SFO1           500.1330885 MHz
NUC1                      1H
P1                    11.25 usec
PLW1           20.00000000 W

======== CHANNEL f2 ========
SFO2           500.1330885 MHz
NUC2                    off
CPDPRG[2
PCPD2      0 usec
PLW2       0 W
PLW12      0 W
PLW13      0 W

F2 - Processing parameters
SI                    65536
SF           500.1300099 MHz
WDW                     EM
SSB        0
LB                    0.30 Hz
GB         0
PC                    1.00
```

Supplementary Figure 113. ^{1}H-NMR of compound **59**, recorded at 500 MHz and 25 °C in $CDCl_3$.

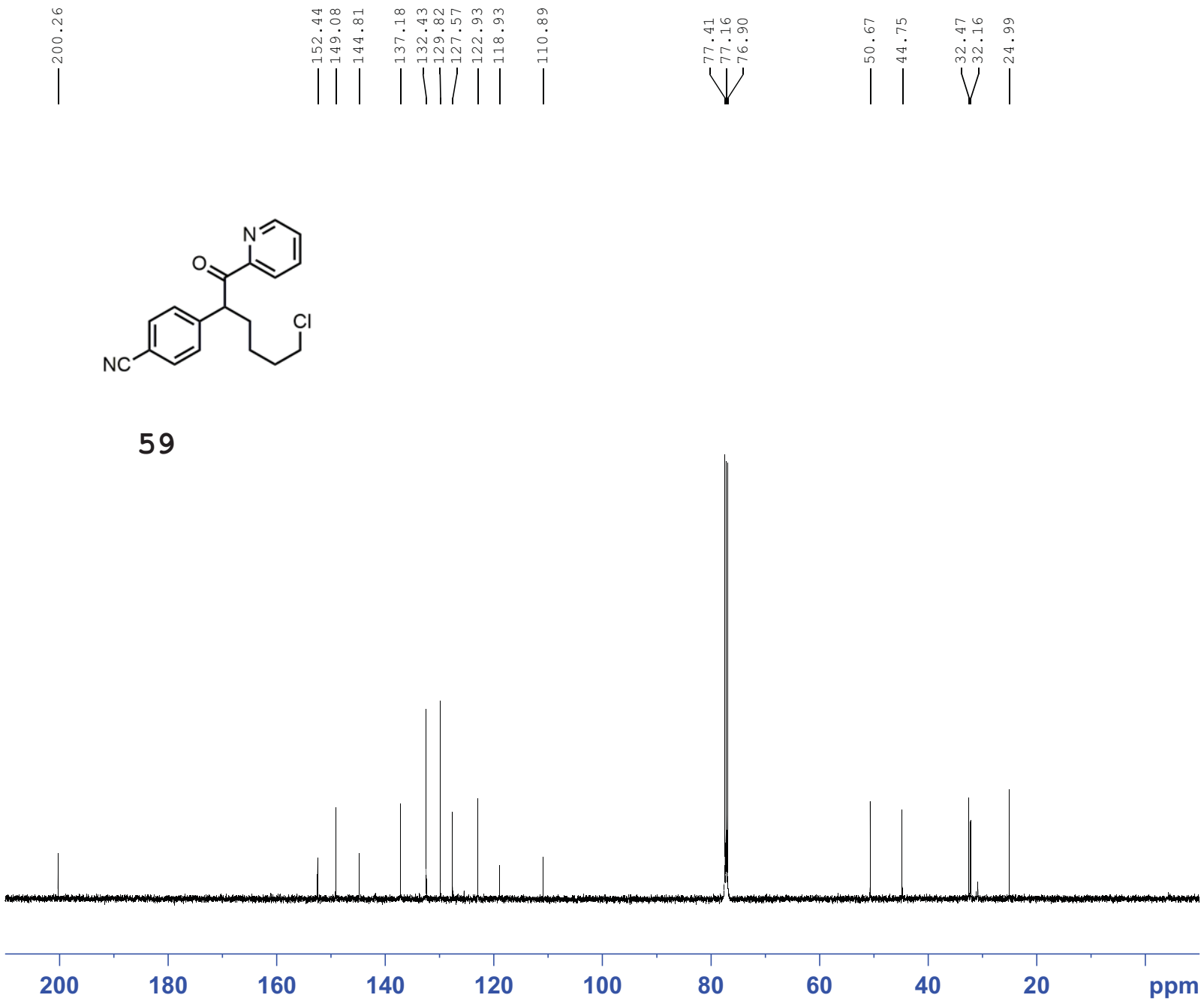


```
Current Data Parameters
NAME                 H-154J
EXPNO                     2
PROCNO                    1

F2 - Acquisition Parameters
Date_              20220301
Time                 20.12
INSTRUM              spect
PROBHD   5 mm CPPBBO BB
PULPROG             zgpg30
TD                   65536
SOLVENT              CDCl3
NS                      27
DS                       4
SWH              29761.904 Hz
FIDRES            0.454131 Hz
AQ                1.1010048 sec
RG                  192.89
DW                  16.800 usec
DE                  18.00 usec
TE                  298.2 K
D1             2.00000000 sec
D11            0.03000000 sec
TD0                      1

======== CHANNEL f1 ========
SFO1           125.7703637 MHz
NUC1                   13C
P1                  10.50 usec
PLW1           57.00000000 W

======== CHANNEL f2 ========
SFO2           500.1320005 MHz
NUC2                   1H
CPDPRG[2           waltz16
PCPD2               80.00 usec
PLW2           20.00000000 W
PLW12           0.39550999 W
PLW13           0.25312999 W

F2 - Processing parameters
SI                    32768
SF             125.7577744 MHz
WDW                      EM
SSB          0
LB                   1.00 Hz
GB           0
PC                   1.40
```

Supplementary Figure 114. ^{13}C-NMR of compound **59**, recorded at 126 MHz and 25 °C in $CDCl_3$.

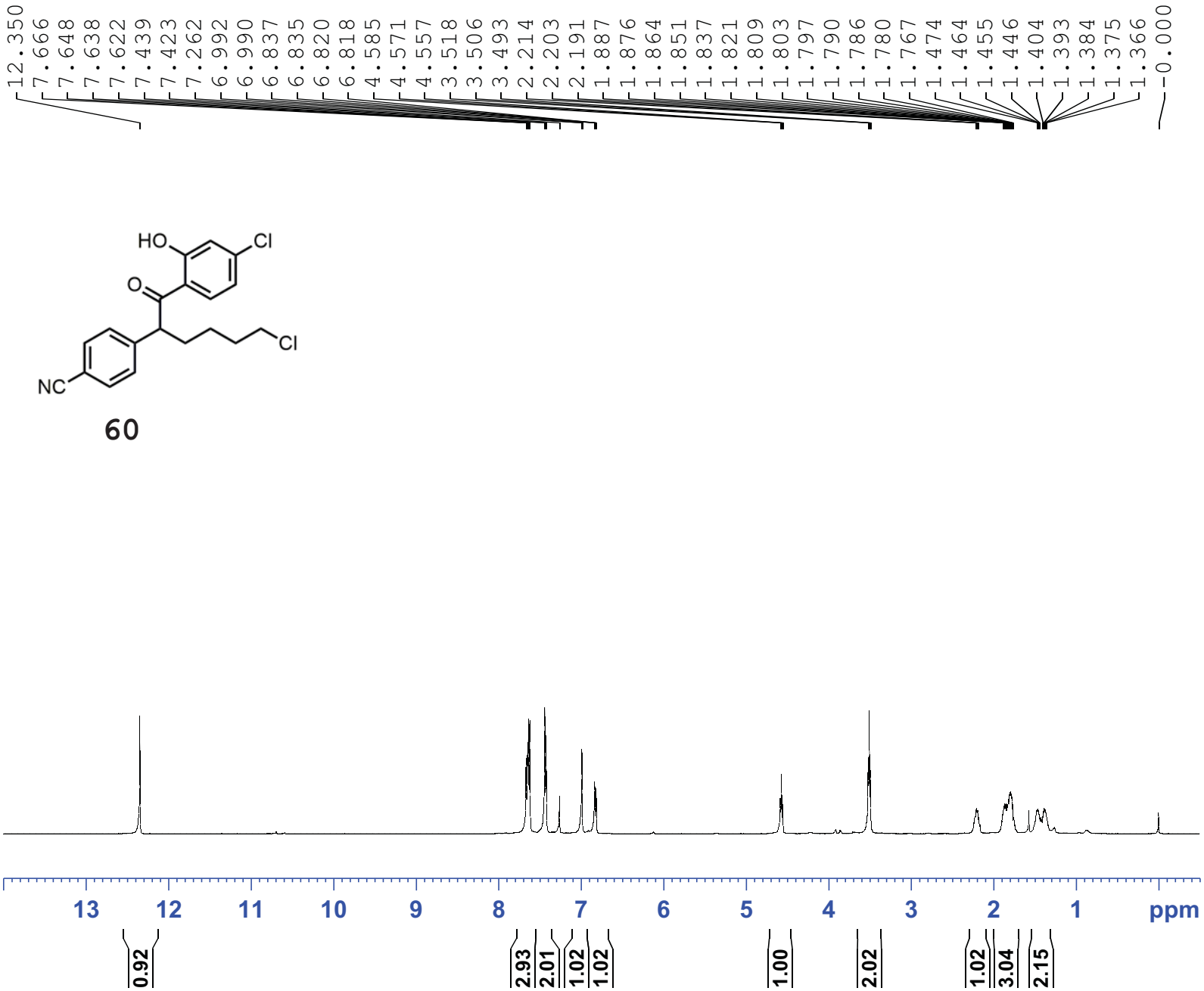


```
Current Data Parameters
NAME                  11189C
EXPNO                      1
PROCNO                     1

F2 - Acquisition Parameters
Date_              20220314
Time                  15.37
INSTRUM               spect
PROBHD    5 mm CPPBBO BB
PULPROG                zg30
TD                    65536
SOLVENT               CDCl3
NS                       16
DS                        2
SWH              10000.000 Hz
FIDRES            0.152588 Hz
AQ               3.2767999 sec
RG                    31.72
DW                   50.000 usec
DE                    6.50 usec
TE                   298.2 K
D1             1.00000000 sec
D11       0 sec
TD0                       1

======== CHANNEL f1 ========
SFO1           500.1330885 MHz
NUC1                      1H
P1                    11.25 usec
PLW1           20.00000000 W

======== CHANNEL f2 ========
SFO2           500.1330885 MHz
NUC2                     off
CPDPRG[2
PCPD2      0 usec
PLW2       0 W
PLW12      0 W
PLW13      0 W

F2 - Processing parameters
SI                    65536
SF               500.1300114 MHz
WDW                      EM
SSB        0
LB                    0.30 Hz
GB         0
PC                    1.00
```

Supplementary Figure 115. ^{1}H-NMR of compound **60**, recorded at 500 MHz and 25 °C in $CDCl_3$.

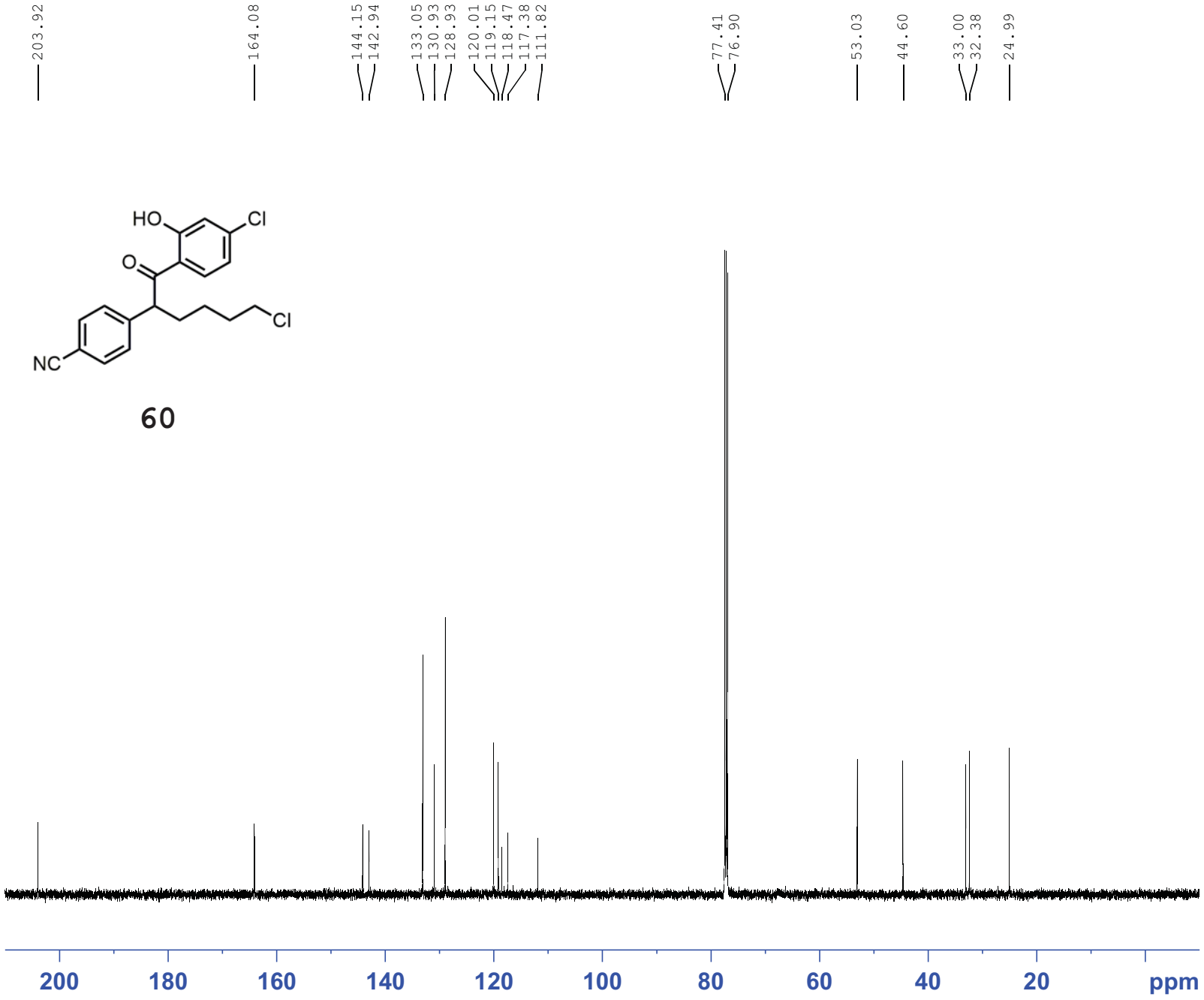


```
Current Data Parameters
NAME                 11189C
EXPNO                     2
PROCNO                    1

F2 - Acquisition Parameters
Date_              20220314
Time                 15.39
INSTRUM              spect
PROBHD   5 mm CPPBBO BB
PULPROG             zgpg30
TD                   65536
SOLVENT              CDCl3
NS                      40
DS                       4
SWH              29761.904 Hz
FIDRES            0.454131 Hz
AQ                1.1010048 sec
RG                  192.89
DW                  16.800 usec
DE                  18.00 usec
TE                  298.2 K
D1              2.00000000 sec
D11             0.03000000 sec
TD0                      1

======== CHANNEL f1 ========
SFO1           125.7703637 MHz
NUC1                   13C
P1                  10.50 usec
PLW1           57.00000000 W

======== CHANNEL f2 ========
SFO2           500.1320005 MHz
NUC2                   1H
CPDPRG[2           waltz16
PCPD2               80.00 usec
PLW2           20.00000000 W
PLW12           0.39550999 W
PLW13           0.25312999 W

F2 - Processing parameters
SI                    32768
SF             125.7577746 MHz
WDW                      EM
SSB          0
LB                   1.00 Hz
GB           0
PC                   1.40
```

Supplementary Figure 116. ^{13}C-NMR of compound **60**, recorded at 126 MHz and 25 °C in $CDCl_3$.

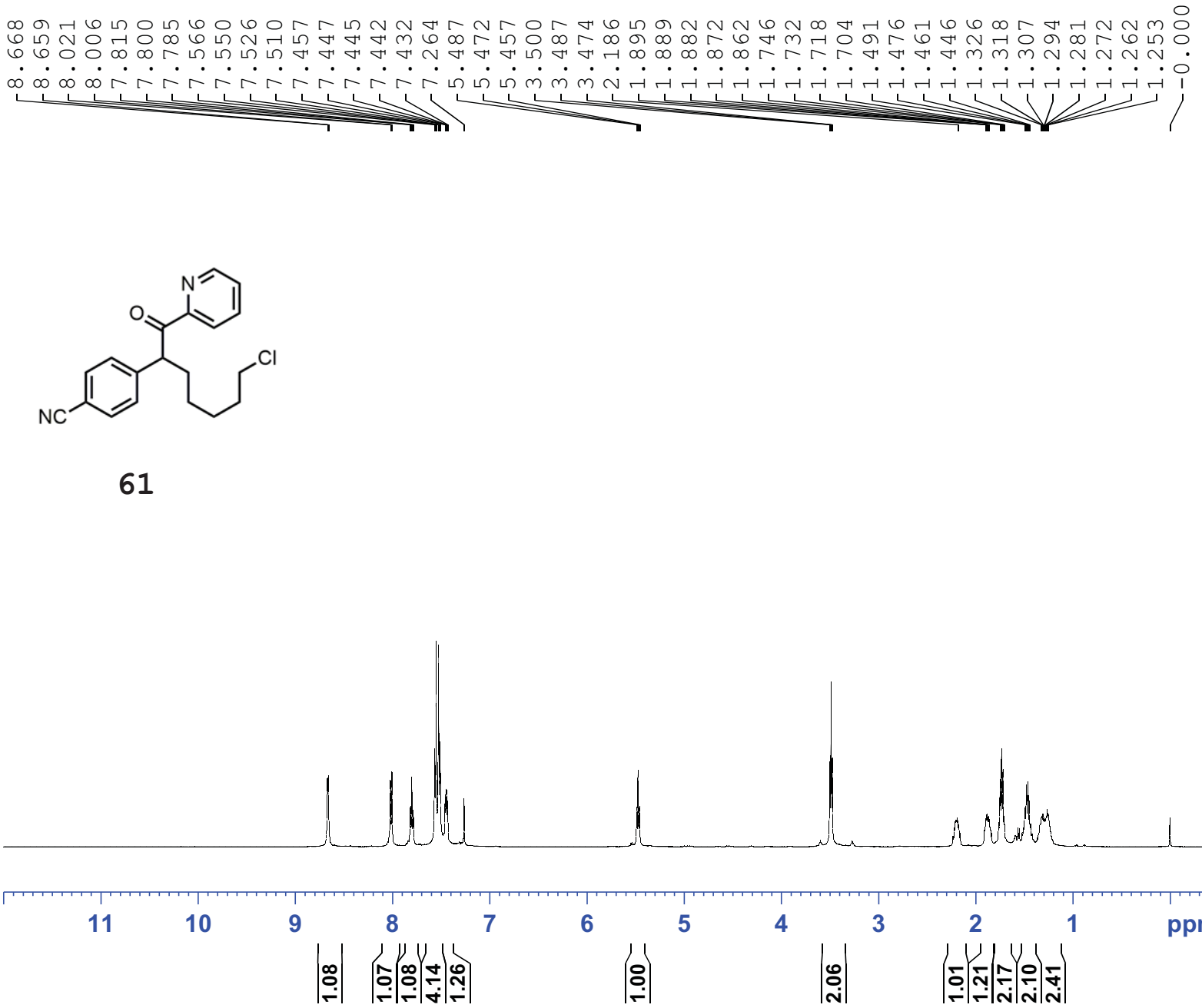


```
Current Data Parameters
NAME                  H-154F
EXPNO                      1
PROCNO                     1

F2 - Acquisition Parameters
Date_              20220301
Time                  20.15
INSTRUM               spect
PROBHD    5 mm CPPBBO BB
PULPROG                zg30
TD                    65536
SOLVENT               CDCl3
NS                       16
DS                        2
SWH              10000.000 Hz
FIDRES            0.152588 Hz
AQ                3.2767999 sec
RG                    55.37
DW                   50.000 usec
DE                    6.50 usec
TE                   298.2 K
D1              1.00000000 sec
D11       0 sec
TD0                       1

======== CHANNEL f1 ========
SFO1           500.1330885 MHz
NUC1                     1H
P1                   11.25 usec
PLW1           20.00000000 W

======== CHANNEL f2 ========
SFO2           500.1330885 MHz
NUC2                   off
CPDPRG[2
PCPD2      0 usec
PLW2       0 W
PLW12      0 W
PLW13      0 W

F2 - Processing parameters
SI                    65536
SF           500.1300109 MHz
WDW                      EM
SSB        0
LB                    0.30 Hz
GB         0
PC                    1.00
```

Supplementary Figure 117. ^{1}H-NMR of compound **61**, recorded at 500 MHz and 25 °C in $CDCl_3$.

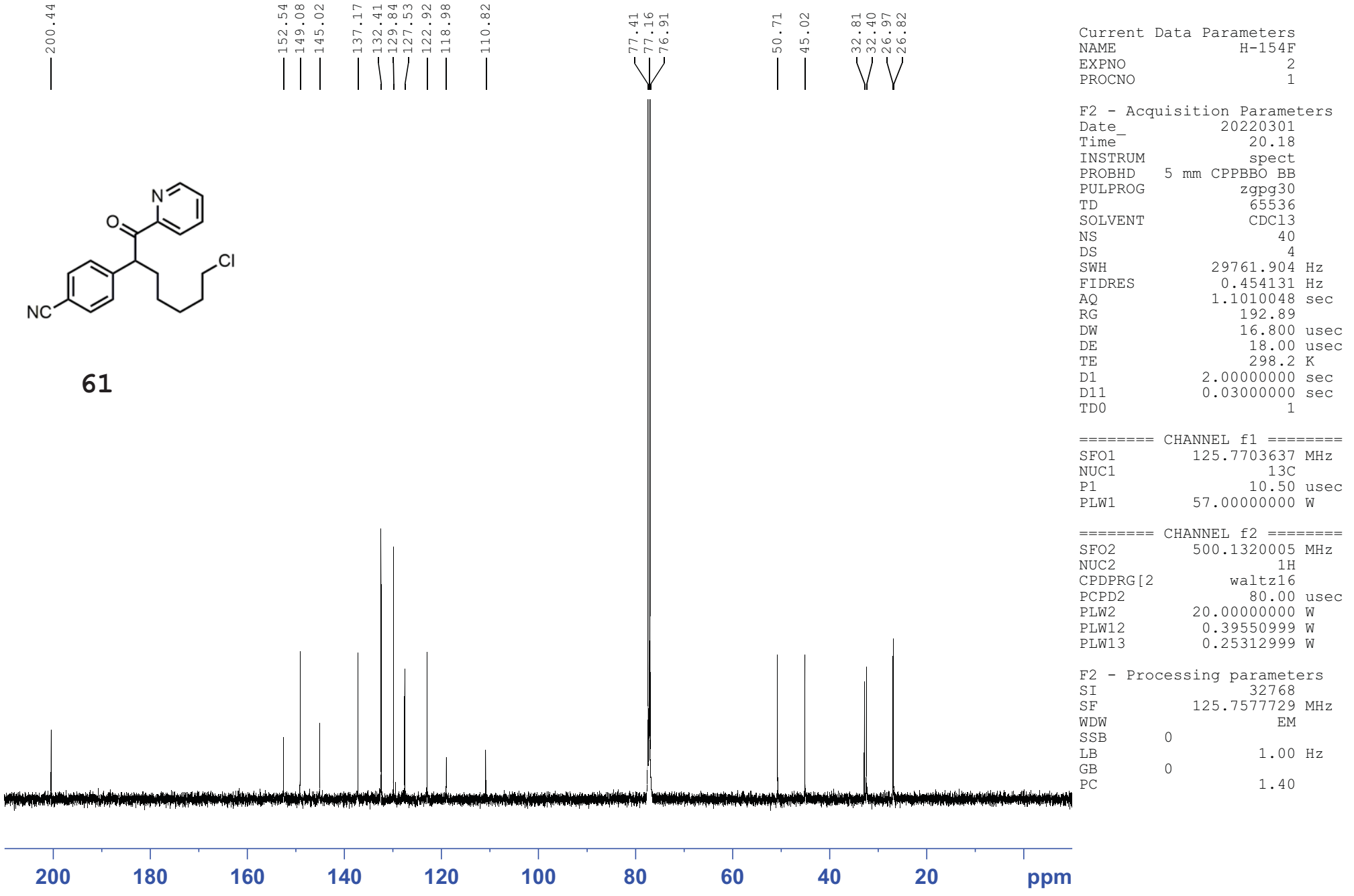


```
Current Data Parameters
NAME                 H-154F
EXPNO                     2
PROCNO                    1

F2 - Acquisition Parameters
Date_              20220301
Time                 20.18
INSTRUM              spect
PROBHD   5 mm CPPBBO BB
PULPROG             zgpg30
TD                   65536
SOLVENT              CDCl3
NS                      40
DS                       4
SWH              29761.904 Hz
FIDRES            0.454131 Hz
AQ                1.1010048 sec
RG                   192.89
DW                  16.800 usec
DE                  18.00 usec
TE                  298.2 K
D1              2.00000000 sec
D11             0.03000000 sec
TD0                       1

======== CHANNEL f1 ========
SFO1           125.7703637 MHz
NUC1                    13C
P1                   10.50 usec
PLW1           57.00000000 W

======== CHANNEL f2 ========
SFO2           500.1320005 MHz
NUC2                    1H
CPDPRG[2           waltz16
PCPD2                80.00 usec
PLW2           20.00000000 W
PLW12           0.39550999 W
PLW13           0.25312999 W

F2 - Processing parameters
SI                      32768
SF             125.7577729 MHz
WDW                        EM
SSB          0
LB                    1.00 Hz
GB           0
PC                    1.40
```

Supplementary Figure 118. ^{13}C-NMR of compound **61**, recorded at 126 MHz and 25 °C in $CDCl_3$.

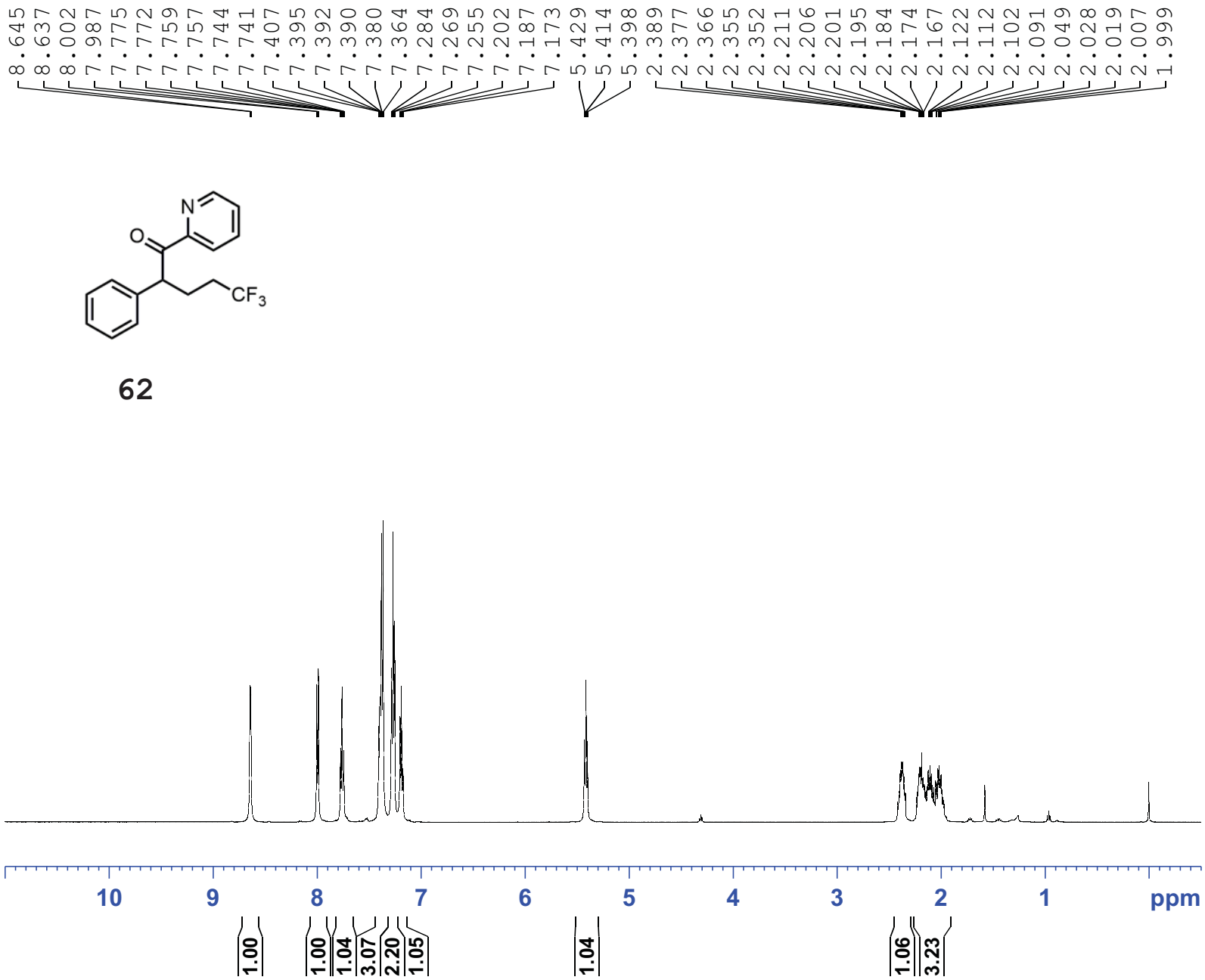


```
Current Data Parameters
NAME                  11136B
EXPNO                      1
PROCNO                     1

F2 - Acquisition Parameters
Date_              20220220
Time                  18.04
INSTRUM               spect
PROBHD   5 mm CPPBBO BB
PULPROG                zg30
TD                    65536
SOLVENT               CDCl3
NS                       16
DS                        2
SWH              10000.000 Hz
FIDRES            0.152588 Hz
AQ               3.2767999 sec
RG                    31.72
DW                  50.000 usec
DE                   6.50 usec
TE                   298.2 K
D1             1.00000000 sec
D11       0 sec
TD0                       1

======== CHANNEL f1 ========
SFO1           500.1330885 MHz
NUC1                     1H
P1                   11.25 usec
PLW1          20.00000000 W

======== CHANNEL f2 ========
SFO2           500.1330885 MHz
NUC2                   off
CPDPRG[2
PCPD2      0 usec
PLW2       0 W
PLW12      0 W
PLW13      0 W

F2 - Processing parameters
SI                    65536
SF           500.1300144 MHz
WDW                      EM
SSB        0
LB                  0.30 Hz
GB         0
PC                  1.00
```

Supplementary Figure 119. ^{1}H-NMR of compound **62**, recorded at 500 MHz and 25 °C in $CDCl_3$.

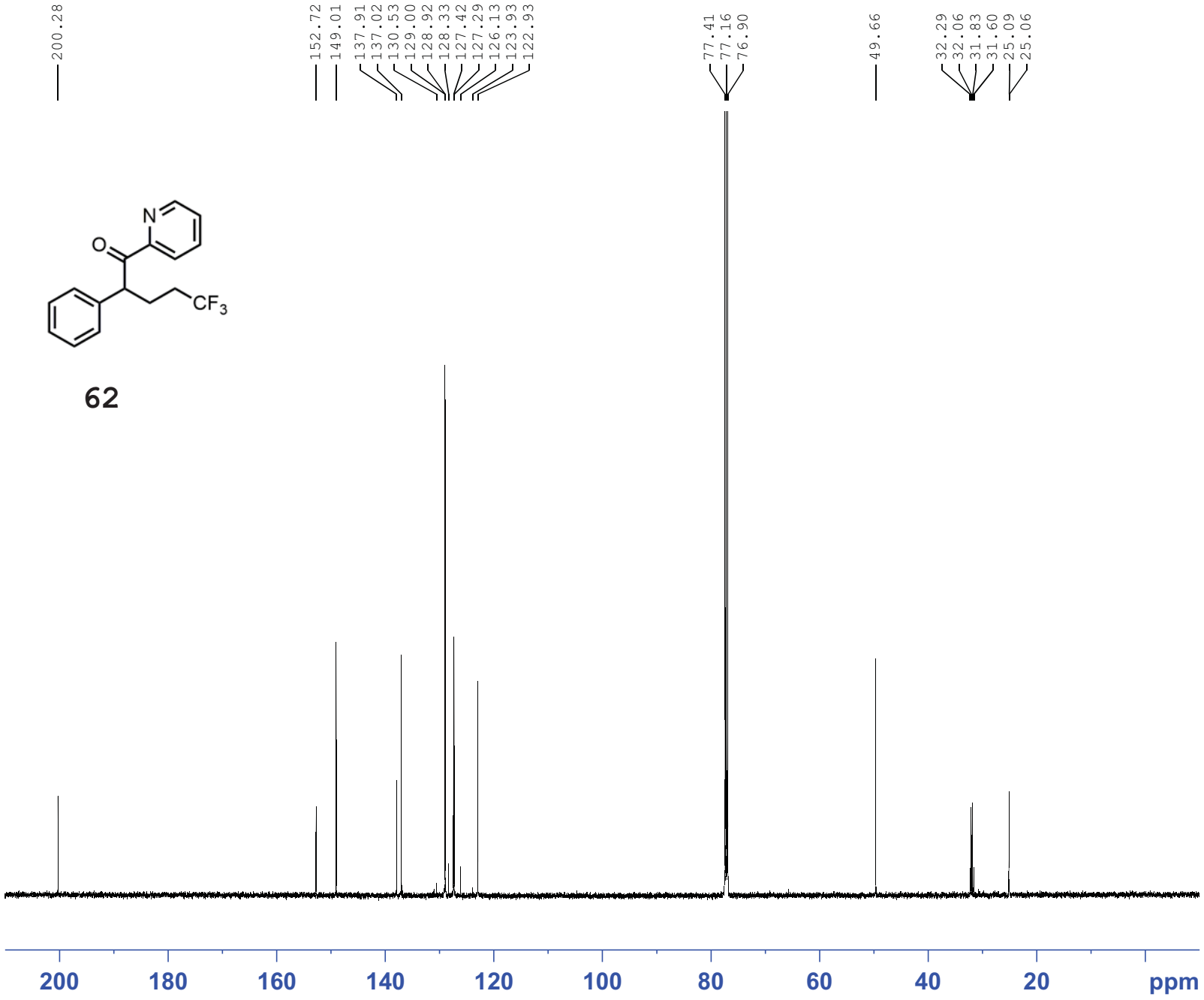


```
Current Data Parameters
NAME                 11136B
EXPNO                     2
PROCNO                    1

F2 - Acquisition Parameters
Date_              20220220
Time                 18.05
INSTRUM              spect
PROBHD   5 mm CPPBBO BB
PULPROG             zgpg30
TD                   65536
SOLVENT              CDCl3
NS                     160
DS                       4
SWH              29761.904 Hz
FIDRES            0.454131 Hz
AQ                1.1010048 sec
RG                   192.89
DW                  16.800 usec
DE                  18.00 usec
TE                  298.2 K
D1              2.00000000 sec
D11             0.03000000 sec
TD0                      1

======== CHANNEL f1 ========
SFO1           125.7703637 MHz
NUC1                   13C
P1                  10.50 usec
PLW1           57.00000000 W

======== CHANNEL f2 ========
SFO2           500.1320005 MHz
NUC2                   1H
CPDPRG[2           waltz16
PCPD2               80.00 usec
PLW2           20.00000000 W
PLW12           0.39550999 W
PLW13           0.25312999 W

F2 - Processing parameters
SI                    32768
SF             125.7577721 MHz
WDW                      EM
SSB          0
LB                   1.00 Hz
GB           0
PC                   1.40
```

Supplementary Figure 120. ^{13}C-NMR of compound **62**, recorded at 126 MHz and 25 °C in $CDCl_3$.

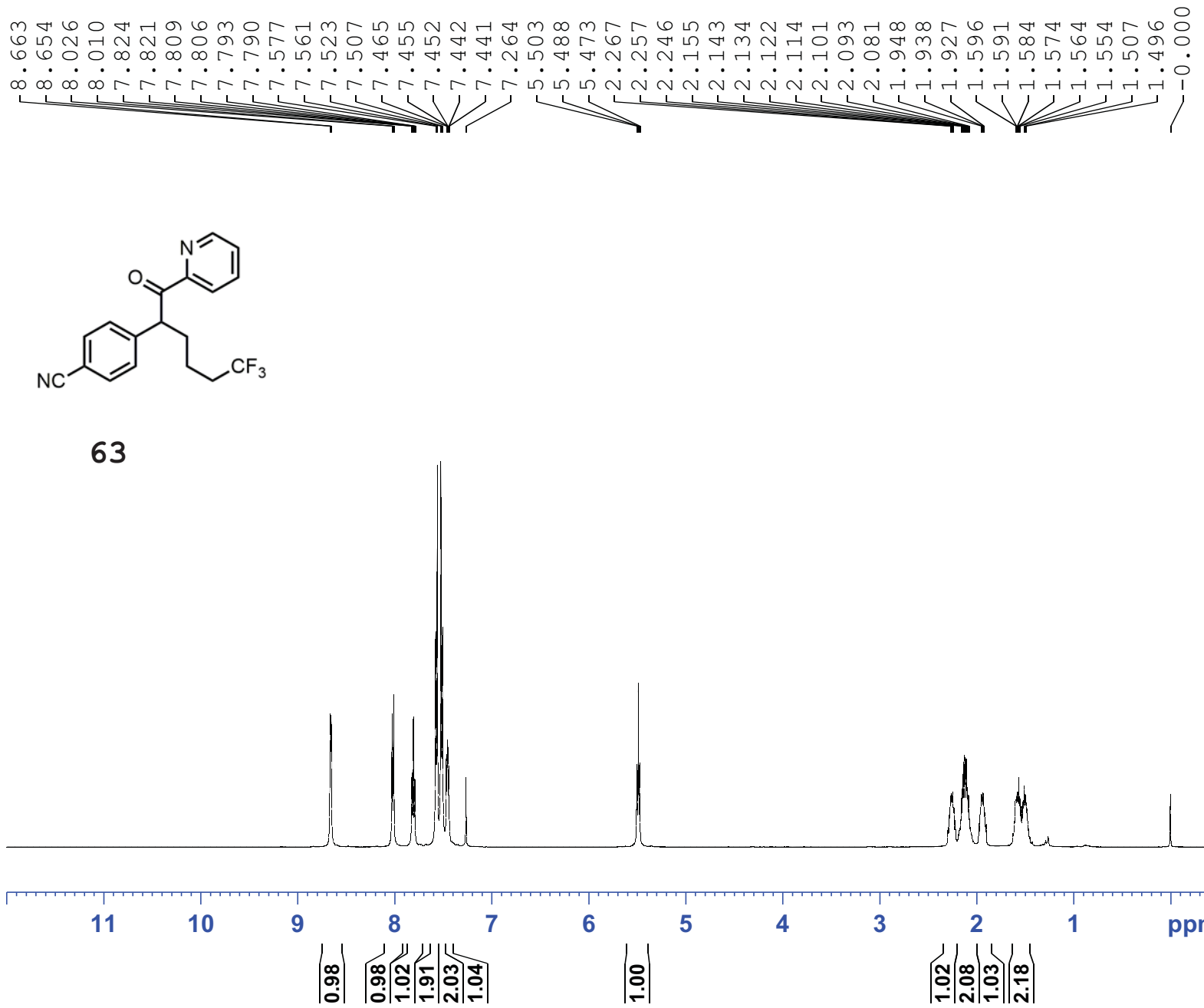


```
Current Data Parameters
NAME                  11154G
EXPNO                      1
PROCNO                     1

F2 - Acquisition Parameters
Date_              20220301
Time                  19.56
INSTRUM               spect
PROBHD    5 mm CPPBBO BB
PULPROG                zg30
TD                    65536
SOLVENT               CDCl3
NS                       16
DS                        2
SWH              10000.000 Hz
FIDRES            0.152588 Hz
AQ               3.2767999 sec
RG                    55.37
DW                  50.000 usec
DE                   6.50 usec
TE                   298.2 K
D1             1.00000000 sec
D11       0 sec
TD0                       1

======== CHANNEL f1 ========
SFO1           500.1330885 MHz
NUC1                     1H
P1                   11.25 usec
PLW1          20.00000000 W

======== CHANNEL f2 ========
SFO2           500.1330885 MHz
NUC2                   off
CPDPRG[2
PCPD2      0 usec
PLW2       0 W
PLW12      0 W
PLW13      0 W

F2 - Processing parameters
SI                    65536
SF               500.1300101 MHz
WDW                      EM
SSB        0
LB                    0.30 Hz
GB         0
PC                    1.00
```

Supplementary Figure 121. ^{1}H-NMR of compound **63**, recorded at 500 MHz and 25 °C in $CDCl_3$.

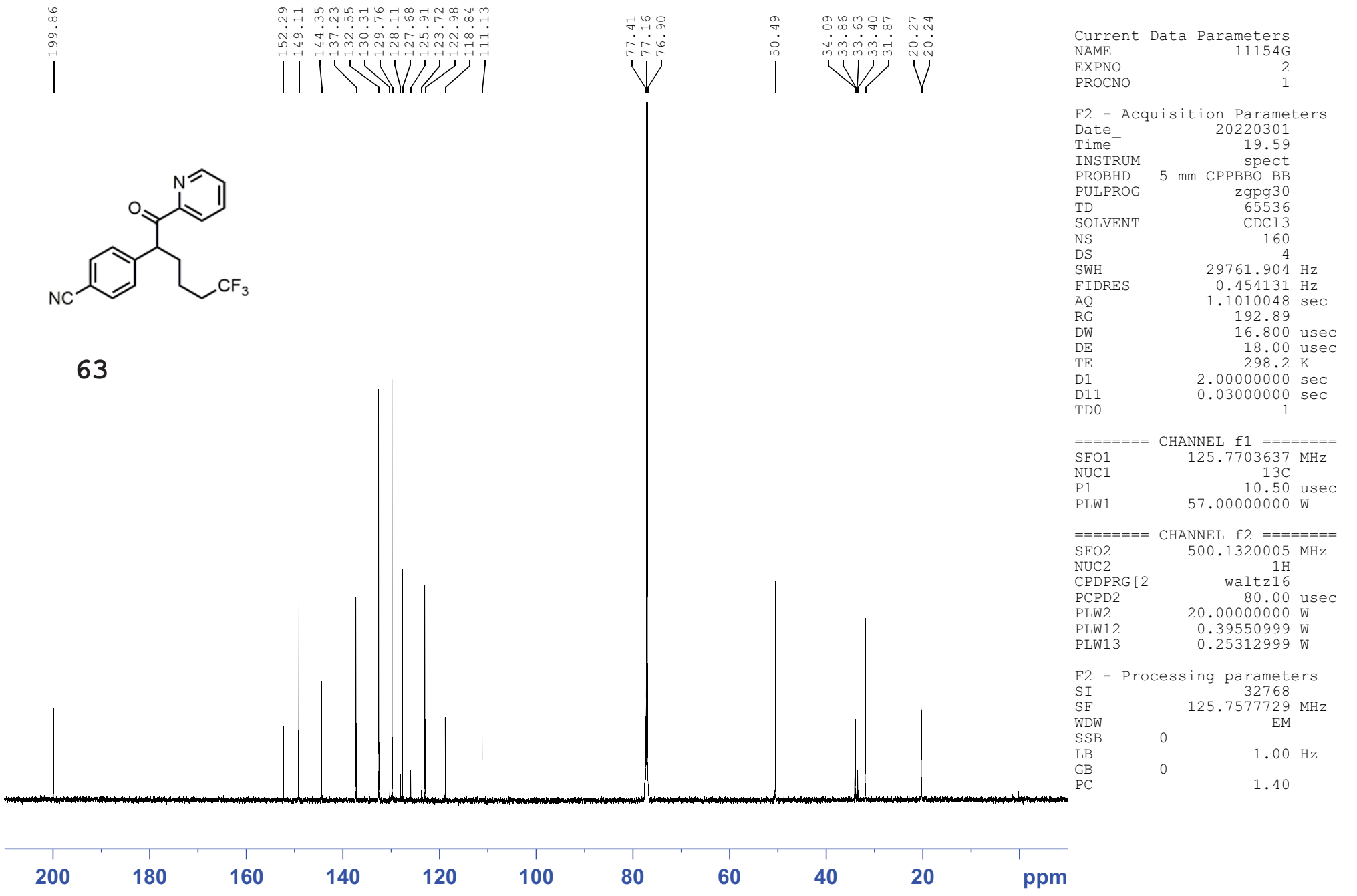


```
Current Data Parameters
NAME                 11154G
EXPNO                     2
PROCNO                    1

F2 - Acquisition Parameters
Date_              20220301
Time                 19.59
INSTRUM              spect
PROBHD   5 mm CPPBBO BB
PULPROG              zgpg30
TD                    65536
SOLVENT               CDCl3
NS                      160
DS                        4
SWH              29761.904 Hz
FIDRES            0.454131 Hz
AQ                1.1010048 sec
RG                   192.89
DW                   16.800 usec
DE                   18.00 usec
TE                   298.2 K
D1              2.00000000 sec
D11             0.03000000 sec
TD0                       1

======== CHANNEL f1 ========
SFO1           125.7703637 MHz
NUC1                    13C
P1                   10.50 usec
PLW1           57.00000000 W

======== CHANNEL f2 ========
SFO2           500.1320005 MHz
NUC2                    1H
CPDPRG[2            waltz16
PCPD2                80.00 usec
PLW2           20.00000000 W
PLW12           0.39550999 W
PLW13           0.25312999 W

F2 - Processing parameters
SI                    32768
SF             125.7577729 MHz
WDW                      EM
SSB          0
LB                    1.00 Hz
GB           0
PC                    1.40
```

Supplementary Figure 122. ^{13}C-NMR of compound **62**, recorded at 126 MHz and 25 °C in $CDCl_3$.

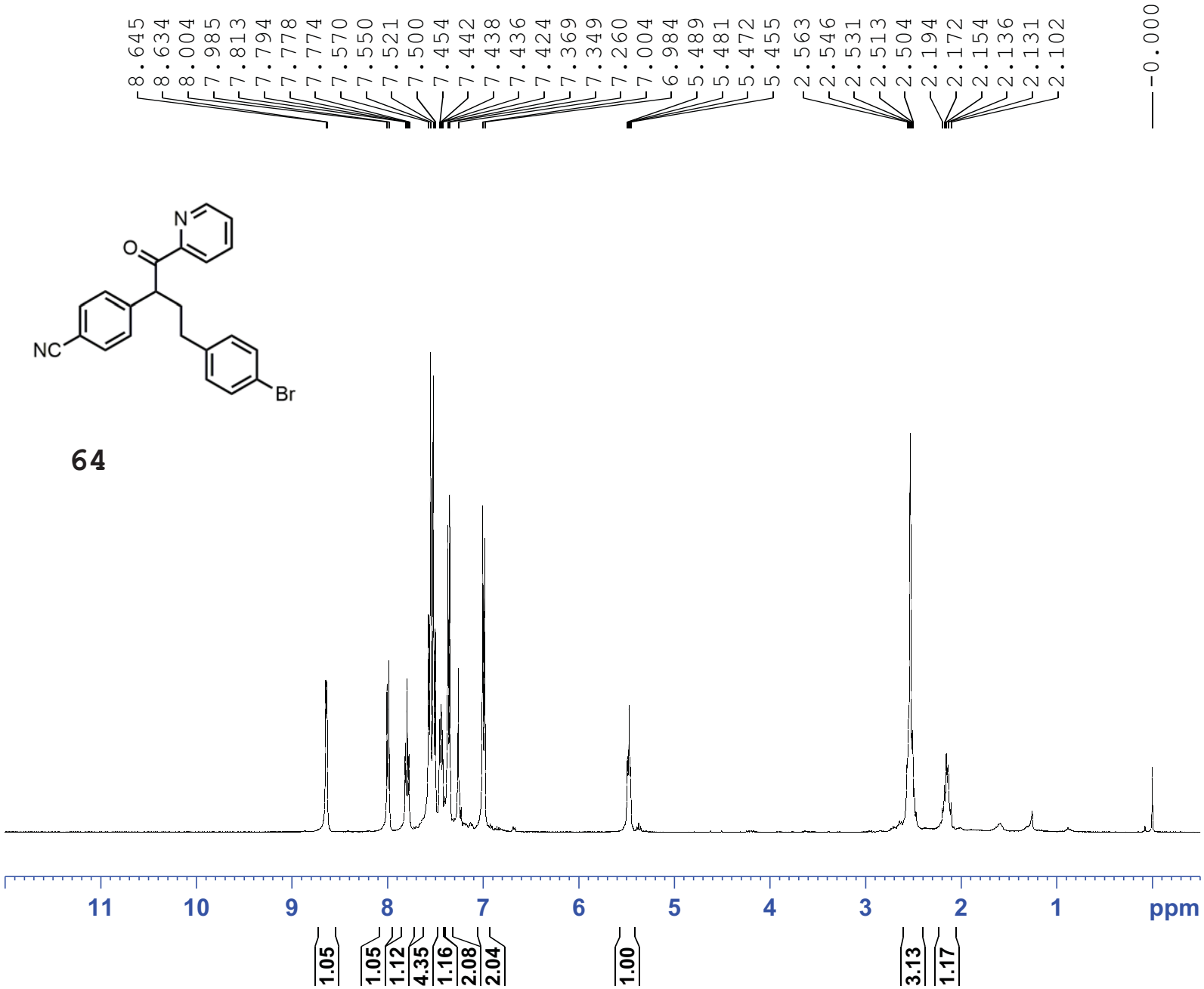


```
Current Data Parameters
NAME                  11158H
EXPNO                      1
PROCNO                     1

F2 - Acquisition Parameters
Date_              20220305
Time                  18.36
INSTRUM               spect
PROBHD    5 mm PABBO BB/
PULPROG                zg30
TD                    32768
SOLVENT               CDCl3
NS                       16
DS                        0
SWH               8012.820 Hz
FIDRES            0.244532 Hz
AQ               2.0447233 sec
RG                   206.33
DW                   62.400 usec
DE                    6.50 usec
TE                   299.3 K
D1             2.00000000 sec
D11       0 sec
TD0                       1

======== CHANNEL f1 ========
SFO1           400.2424716 MHz
NUC1                     1H
P1                   14.30 usec
PLW1           12.00000000 W

======== CHANNEL f2 ========
SFO2           400.2424716 MHz
NUC2                    off
CPDPRG[2
PCPD2      0 usec
PLW2       0 W
PLW12      0 W
PLW13      0 W

F2 - Processing parameters
SI                    65536
SF           400.2400103 MHz
WDW                      EM
SSB        0
LB                   0.30 Hz
GB         0
PC                   1.00
```

Supplementary Figure 123. ^{1}H-NMR of compound **64**, recorded at 400 MHz and 25 °C in $CDCl_3$.

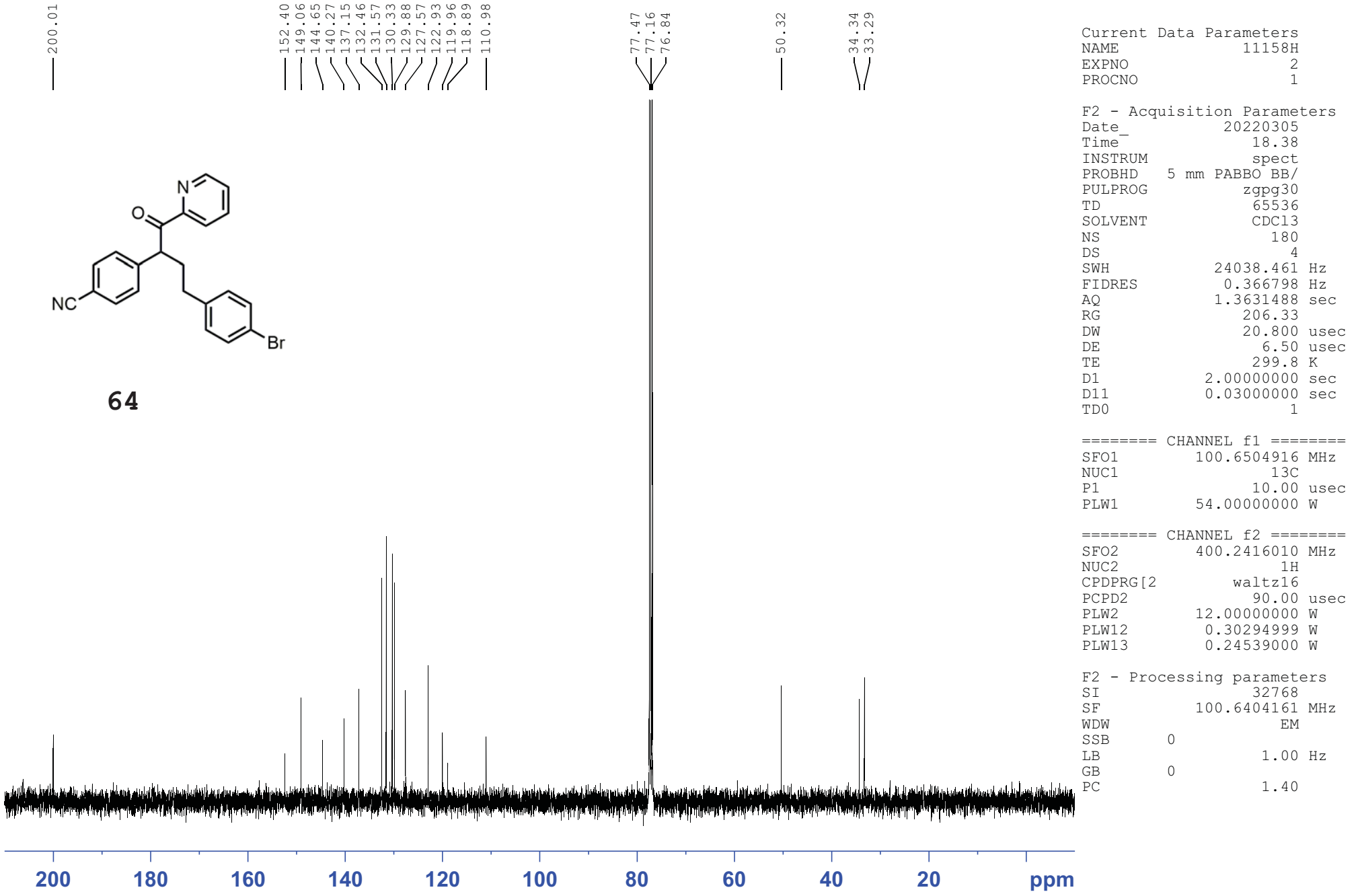


```
Current Data Parameters
NAME                 11158H
EXPNO                     2
PROCNO                    1

F2 - Acquisition Parameters
Date_              20220305
Time                 18.38
INSTRUM              spect
PROBHD    5 mm PABBO BB/
PULPROG             zgpg30
TD                   65536
SOLVENT              CDCl3
NS                     180
DS                       4
SWH             24038.461 Hz
FIDRES           0.366798 Hz
AQ               1.3631488 sec
RG                  206.33
DW                  20.800 usec
DE                   6.50 usec
TE                  299.8 K
D1              2.00000000 sec
D11             0.03000000 sec
TD0                      1

======== CHANNEL f1 ========
SFO1           100.6504916 MHz
NUC1                   13C
P1                  10.00 usec
PLW1           54.00000000 W

======== CHANNEL f2 ========
SFO2           400.2416010 MHz
NUC2                   1H
CPDPRG[2           waltz16
PCPD2               90.00 usec
PLW2           12.00000000 W
PLW12           0.30294999 W
PLW13           0.24539000 W

F2 - Processing parameters
SI                   32768
SF             100.6404161 MHz
WDW                     EM
SSB          0
LB                   1.00 Hz
GB           0
PC                   1.40
```

Supplementary Figure 124. ^{13}C-NMR of compound **64**, recorded at 101 MHz and 25 °C in $CDCl_3$.

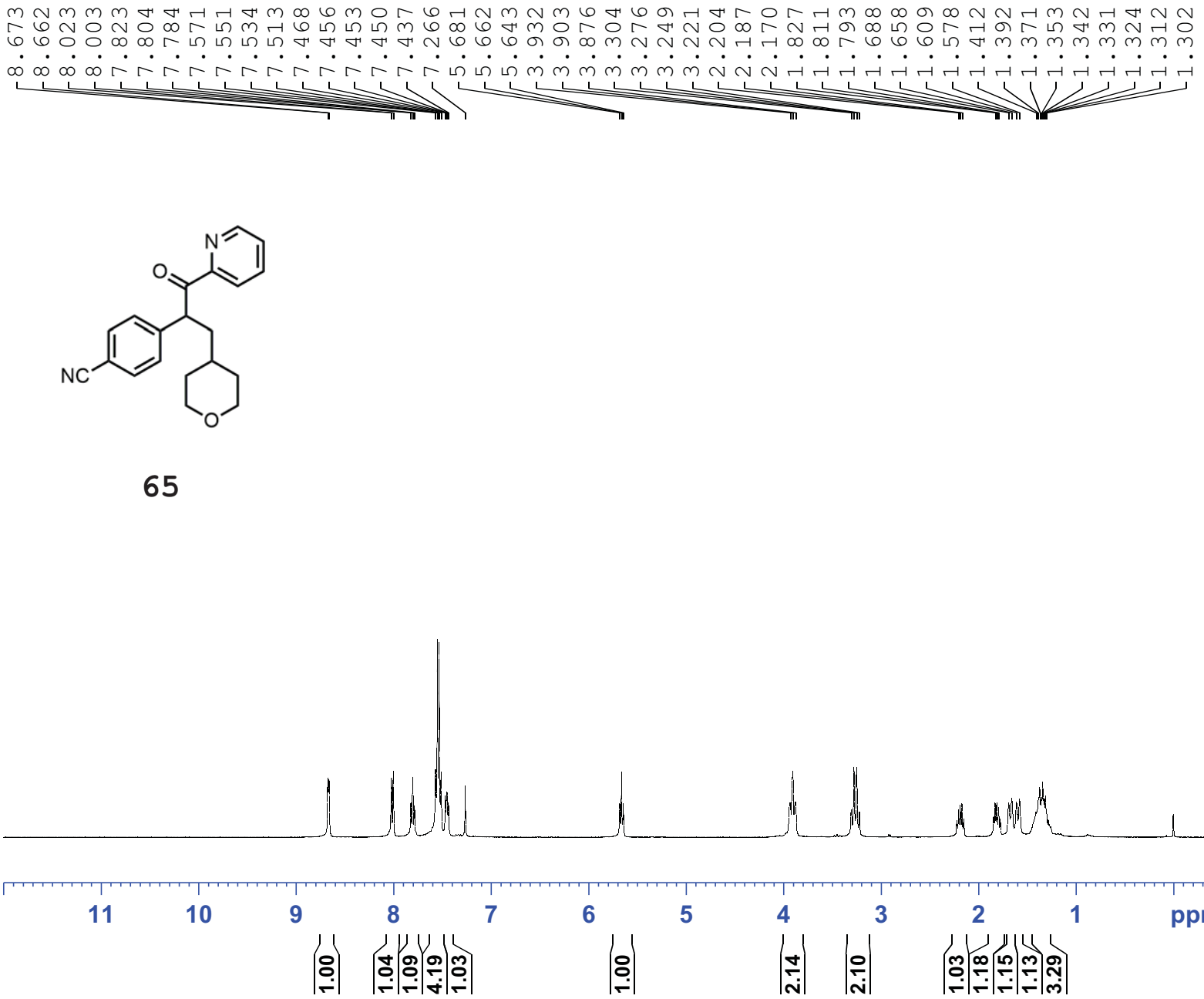


```
Current Data Parameters
NAME                  11158A
EXPNO                      1
PROCNO                     1

F2 - Acquisition Parameters
Date_              20220305
Time                  18.07
INSTRUM               spect
PROBHD    5 mm PABBO BB/
PULPROG                zg30
TD                    32768
SOLVENT               CDCl3
NS                       16
DS                        0
SWH               8012.820 Hz
FIDRES            0.244532 Hz
AQ               2.0447233 sec
RG                   206.33
DW                   62.400 usec
DE                    6.50 usec
TE                   299.2 K
D1             2.00000000 sec
D11       0 sec
TD0                       1

======== CHANNEL f1 ========
SFO1          400.2424716 MHz
NUC1                     1H
P1                   14.30 usec
PLW1          12.00000000 W

======== CHANNEL f2 ========
SFO2          400.2424716 MHz
NUC2                   off
CPDPRG[2
PCPD2      0 usec
PLW2       0 W
PLW12      0 W
PLW13      0 W

F2 - Processing parameters
SI                    65536
SF            400.2400078 MHz
WDW                      EM
SSB        0
LB                    0.30 Hz
GB         0
PC                    1.00
```

Supplementary Figure 125. ^{1}H-NMR of compound **65**, recorded at 400 MHz and 25 °C in $CDCl_3$.

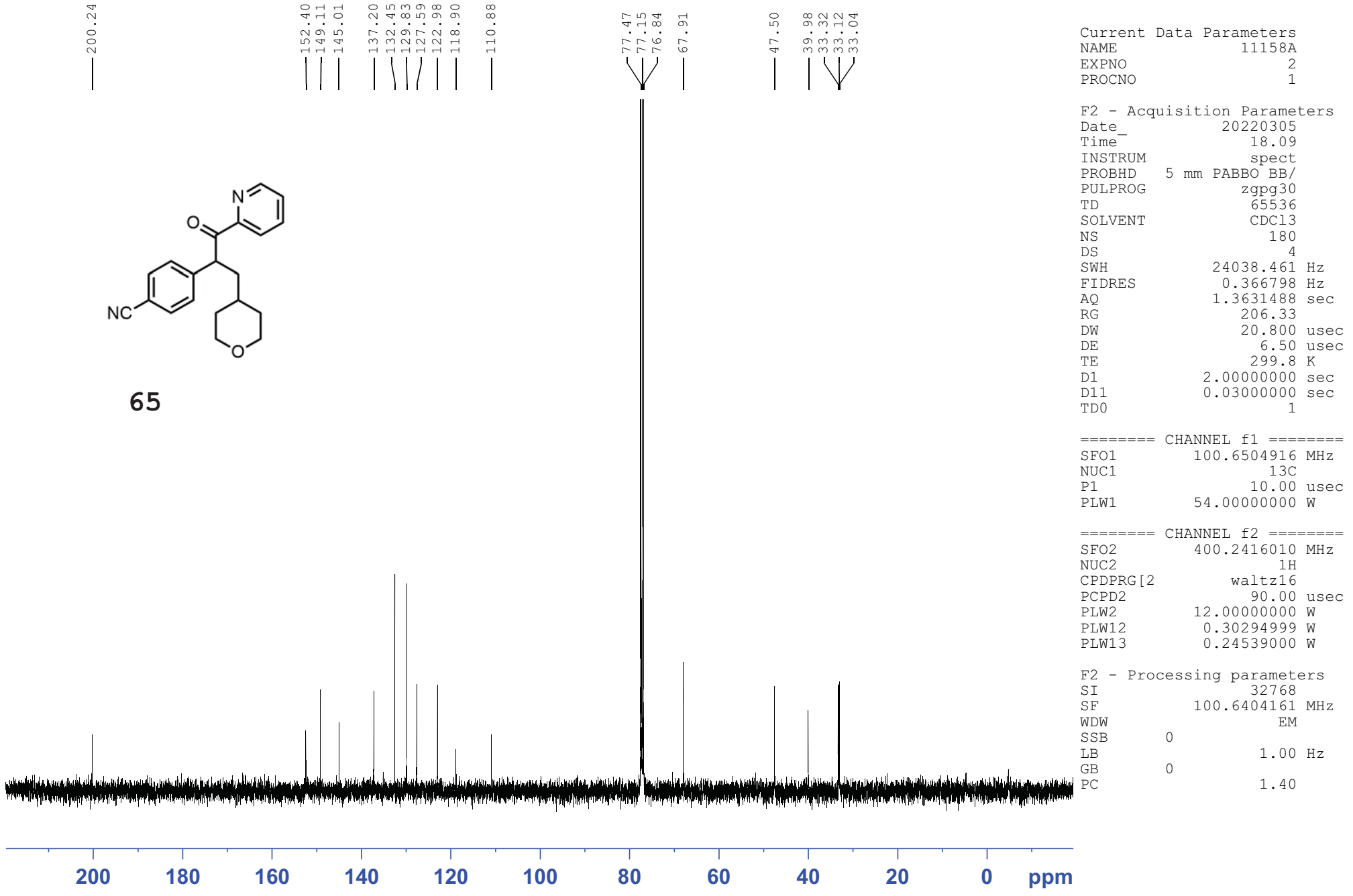


```
Current Data Parameters
NAME                 11158A
EXPNO                     2
PROCNO                    1

F2 - Acquisition Parameters
Date_              20220305
Time                 18.09
INSTRUM              spect
PROBHD    5 mm PABBO BB/
PULPROG             zgpg30
TD                   65536
SOLVENT              CDCl3
NS                     180
DS                       4
SWH             24038.461 Hz
FIDRES           0.366798 Hz
AQ               1.3631488 sec
RG                  206.33
DW                  20.800 usec
DE                   6.50 usec
TE                  299.8 K
D1             2.00000000 sec
D11            0.03000000 sec
TD0                      1

======== CHANNEL f1 ========
SFO1           100.6504916 MHz
NUC1                   13C
P1                  10.00 usec
PLW1           54.00000000 W

======== CHANNEL f2 ========
SFO2           400.2416010 MHz
NUC2                   1H
CPDPRG[2           waltz16
PCPD2               90.00 usec
PLW2           12.00000000 W
PLW12           0.30294999 W
PLW13           0.24539000 W

F2 - Processing parameters
SI                   32768
SF             100.6404161 MHz
WDW                     EM
SSB          0
LB                   1.00 Hz
GB           0
PC                   1.40
```

Supplementary Figure 126. ^{13}C-NMR of compound **65**, recorded at 101 MHz and 25 °C in $CDCl_3$.

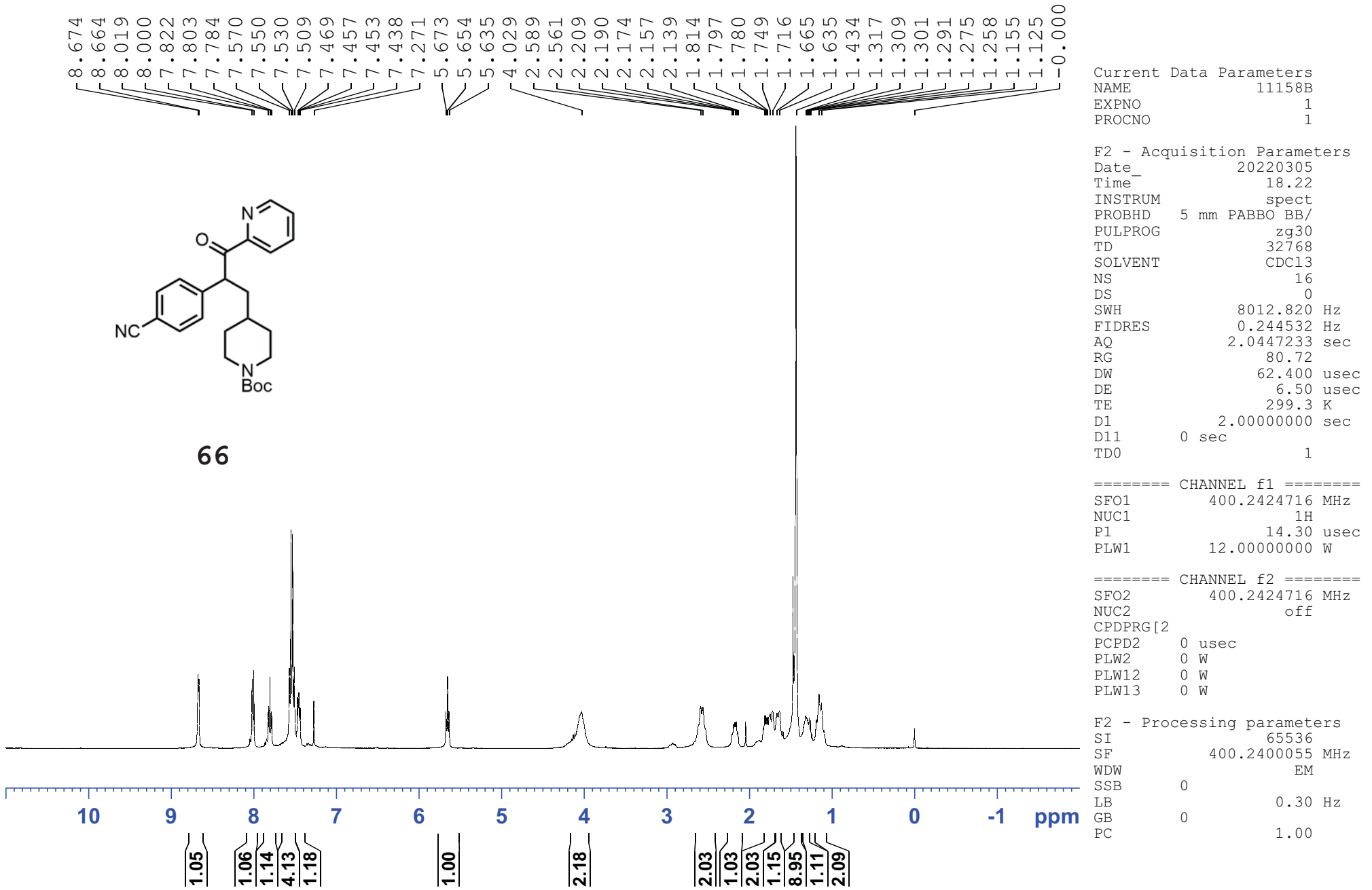


```
Current Data Parameters
NAME                  11158B
EXPNO                      1
PROCNO                     1

F2 - Acquisition Parameters
Date_              20220305
Time                  18.22
INSTRUM               spect
PROBHD    5 mm PABBO BB/
PULPROG                zg30
TD                    32768
SOLVENT               CDCl3
NS                       16
DS                        0
SWH               8012.820 Hz
FIDRES             0.244532 Hz
AQ                2.0447233 sec
RG                     80.72
DW                   62.400 usec
DE                    6.50 usec
TE                   299.3 K
D1              2.00000000 sec
D11       0 sec
TD0                        1

======== CHANNEL f1 ========
SFO1           400.2424716 MHz
NUC1                      1H
P1                    14.30 usec
PLW1           12.00000000 W

======== CHANNEL f2 ========
SFO2           400.2424716 MHz
NUC2                     off
CPDPRG[2
PCPD2      0 usec
PLW2       0 W
PLW12      0 W
PLW13      0 W

F2 - Processing parameters
SI                     65536
SF             400.2400055 MHz
WDW                       EM
SSB        0
LB                    0.30 Hz
GB         0
PC                    1.00
```

Supplementary Figure 127. ^{1}H-NMR of compound **66**, recorded at 400 MHz and 25 °C in $CDCl_3$.

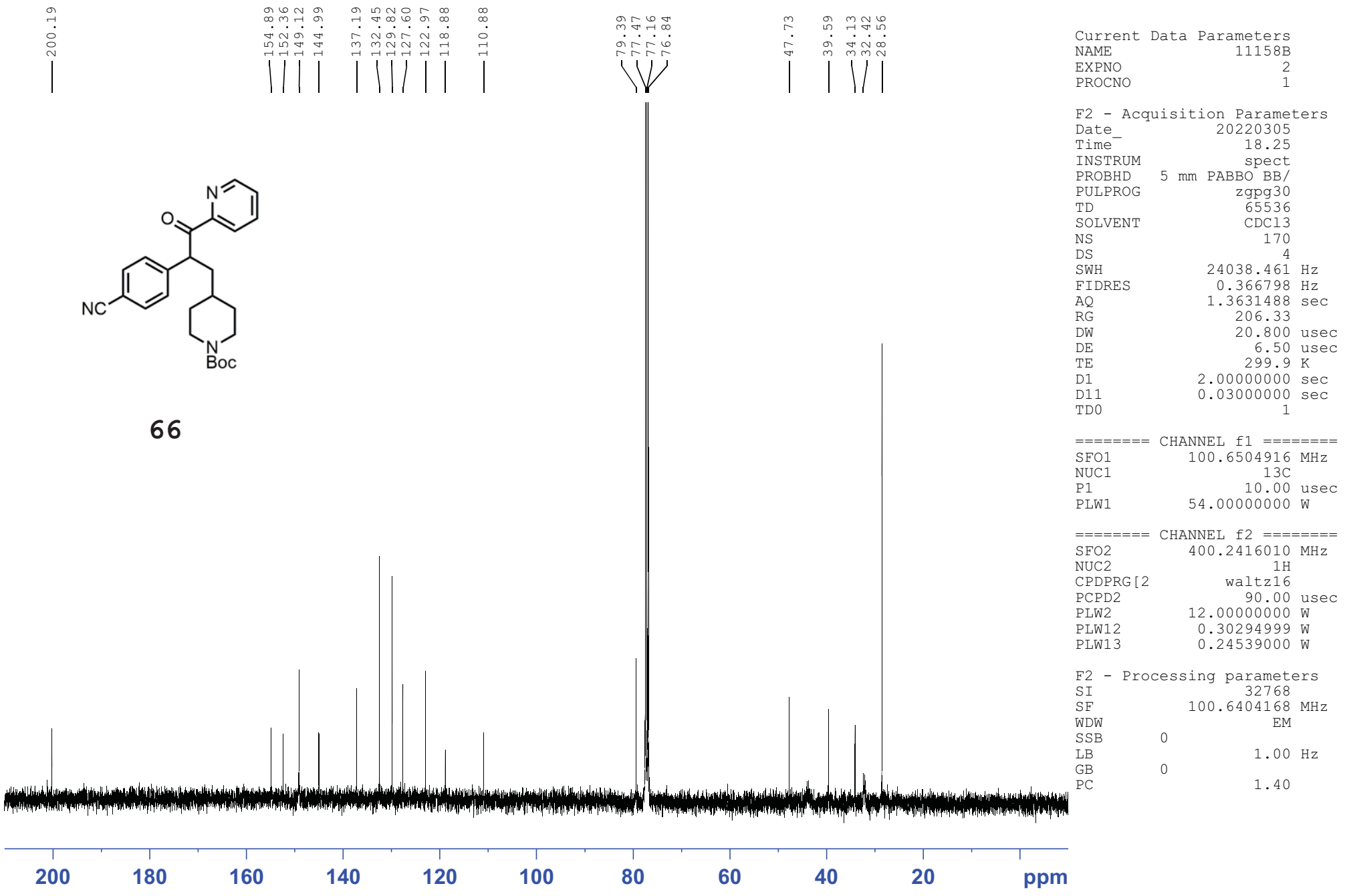


```
Current Data Parameters
NAME                 11158B
EXPNO                     2
PROCNO                    1

F2 - Acquisition Parameters
Date_              20220305
Time                 18.25
INSTRUM              spect
PROBHD    5 mm PABBO BB/
PULPROG             zgpg30
TD                   65536
SOLVENT              CDCl3
NS                     170
DS                       4
SWH             24038.461 Hz
FIDRES           0.366798 Hz
AQ               1.3631488 sec
RG                  206.33
DW                  20.800 usec
DE                   6.50 usec
TE                  299.9 K
D1              2.00000000 sec
D11             0.03000000 sec
TD0                      1

======== CHANNEL f1 ========
SFO1           100.6504916 MHz
NUC1                   13C
P1                  10.00 usec
PLW1           54.00000000 W

======== CHANNEL f2 ========
SFO2           400.2416010 MHz
NUC2                   1H
CPDPRG[2           waltz16
PCPD2               90.00 usec
PLW2           12.00000000 W
PLW12           0.30294999 W
PLW13           0.24539000 W

F2 - Processing parameters
SI                     32768
SF             100.6404168 MHz
WDW                      EM
SSB          0
LB                   1.00 Hz
GB           0
PC                   1.40
```

Supplementary Figure 128. ^{13}C-NMR of compound **66**, recorded at 101 MHz and 25 °C in $CDCl_3$.

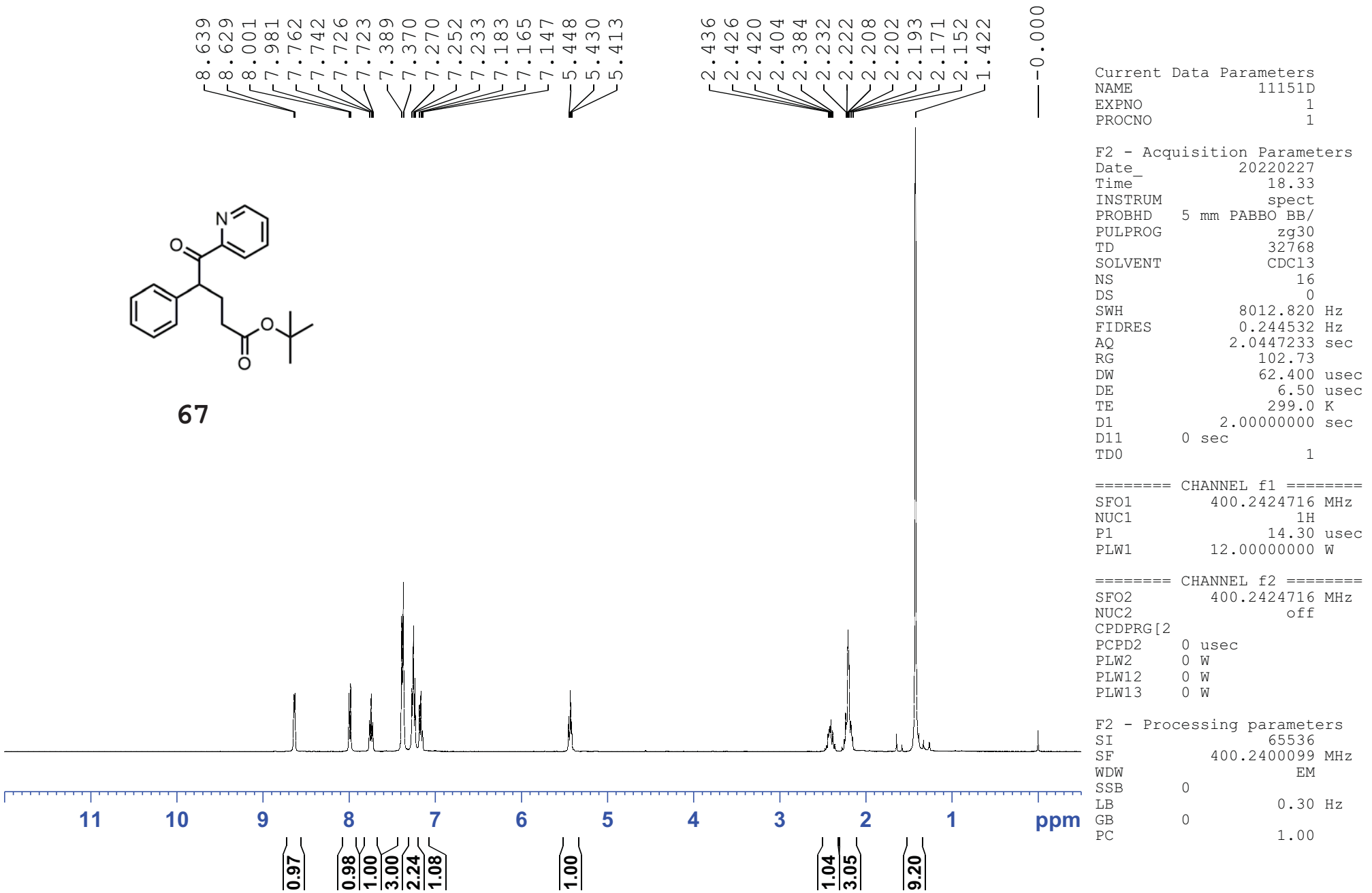

Supplementary Figure 129. ^{1}H-NMR of compound **67**, recorded at 400 MHz and 25 °C in $CDCl_3$.

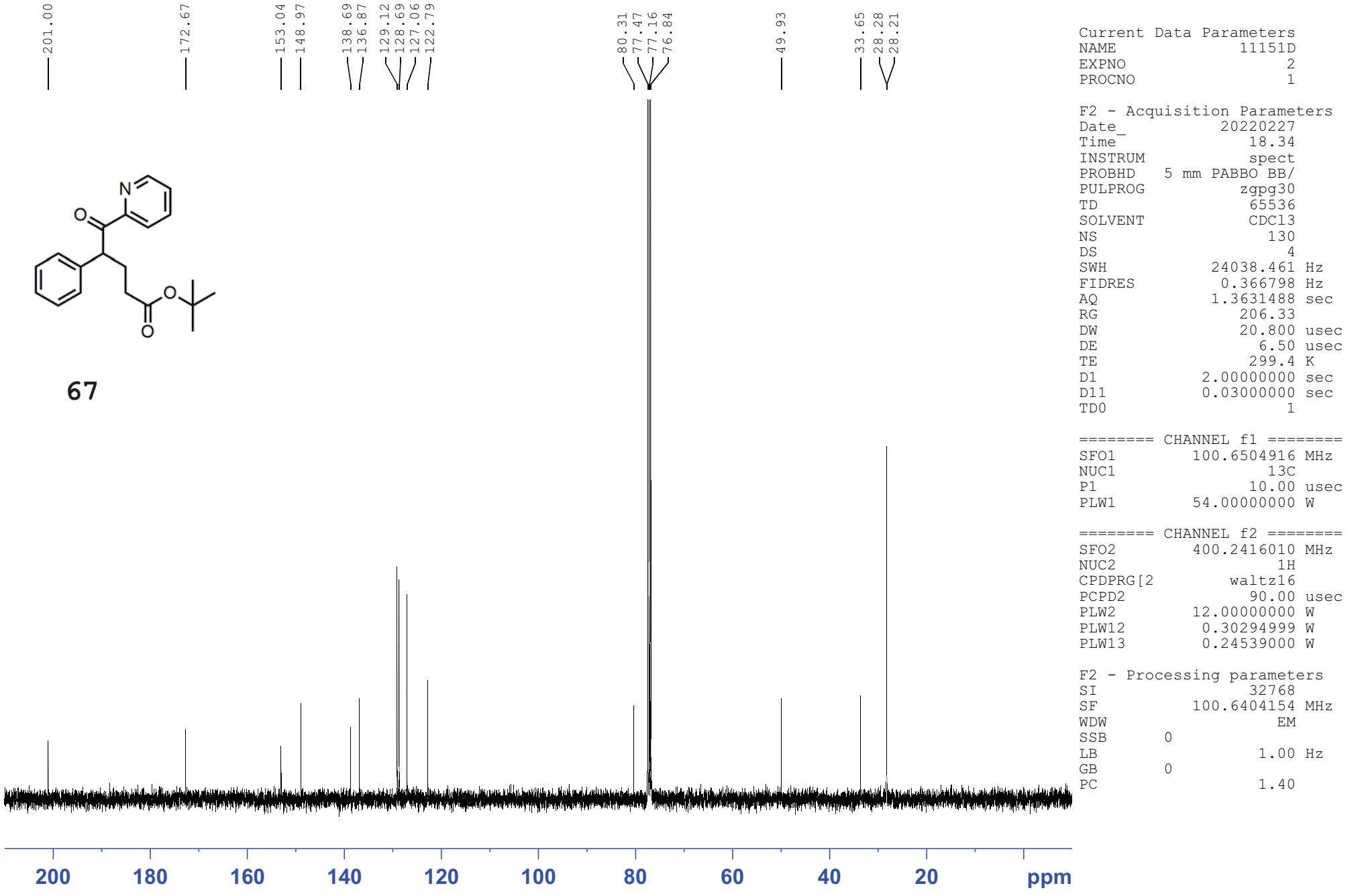


```
Current Data Parameters
NAME                 11151D
EXPNO                     2
PROCNO                    1

F2 - Acquisition Parameters
Date_              20220227
Time                 18.34
INSTRUM              spect
PROBHD    5 mm PABBO BB/
PULPROG             zgpg30
TD                   65536
SOLVENT              CDCl3
NS                     130
DS                       4
SWH             24038.461 Hz
FIDRES           0.366798 Hz
AQ               1.3631488 sec
RG                  206.33
DW                  20.800 usec
DE                   6.50 usec
TE                  299.4 K
D1              2.00000000 sec
D11             0.03000000 sec
TD0                      1

======== CHANNEL f1 ========
SFO1           100.6504916 MHz
NUC1                   13C
P1                  10.00 usec
PLW1           54.00000000 W

======== CHANNEL f2 ========
SFO2           400.2416010 MHz
NUC2                   1H
CPDPRG[2           waltz16
PCPD2               90.00 usec
PLW2           12.00000000 W
PLW12           0.30294999 W
PLW13           0.24539000 W

F2 - Processing parameters
SI                     32768
SF             100.6404154 MHz
WDW                       EM
SSB          0
LB                   1.00 Hz
GB          0
PC                   1.40
```

Supplementary Figure 130. ^{13}C-NMR of compound **67**, recorded at 101 MHz and 25 °C in $CDCl_3$.

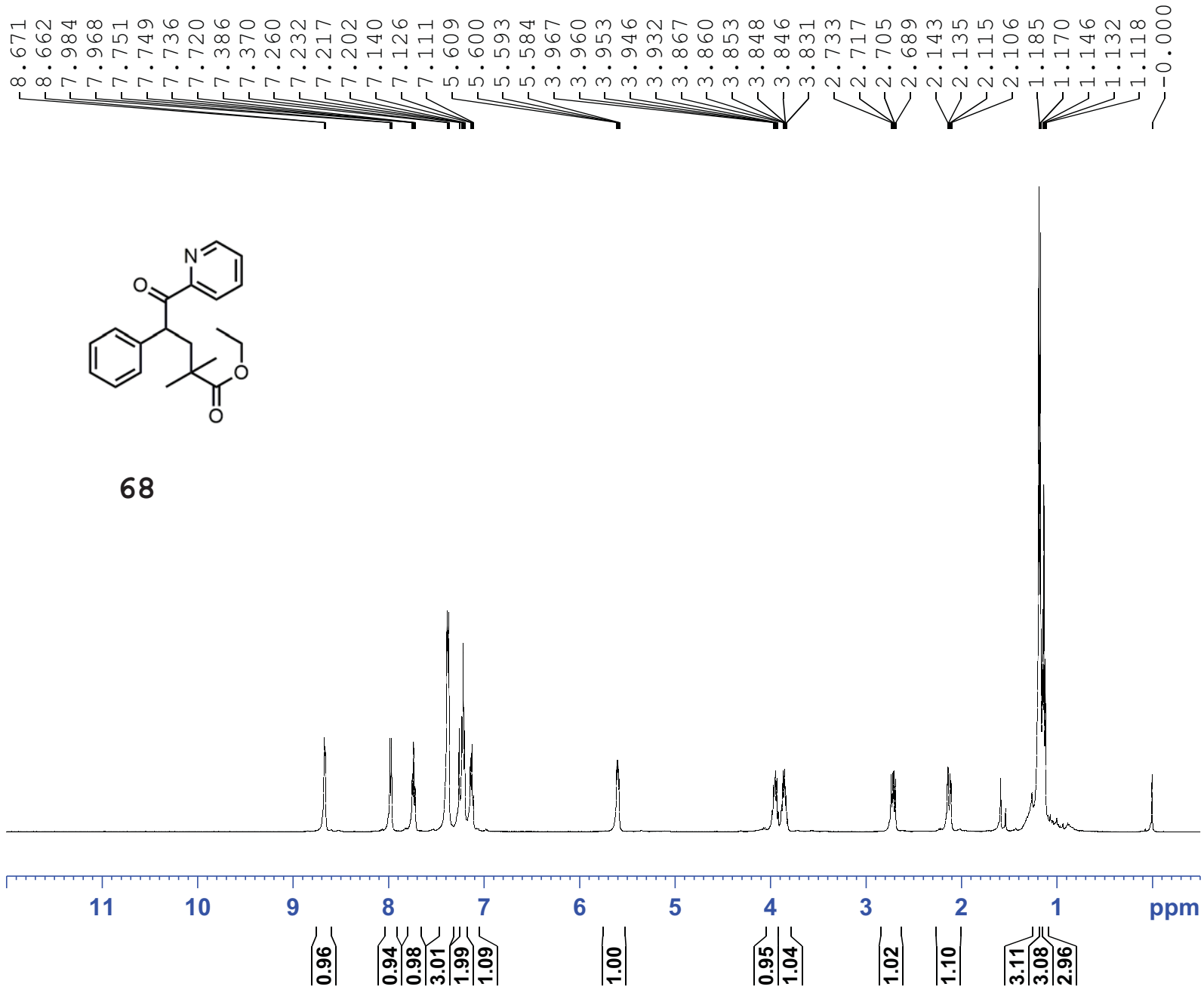


```
Current Data Parameters
NAME                  11156B
EXPNO                      1
PROCNO                     1

F2 - Acquisition Parameters
Date_              20220304
Time                  15.59
INSTRUM               spect
PROBHD    5 mm CPPBBO BB
PULPROG                zg30
TD                    65536
SOLVENT               CDCl3
NS                       16
DS                        2
SWH              10000.000 Hz
FIDRES            0.152588 Hz
AQ               3.2767999 sec
RG                    55.37
DW                  50.000 usec
DE                   6.50 usec
TE                   298.2 K
D1             1.00000000 sec
D11       0 sec
TD0                       1

======== CHANNEL f1 ========
SFO1           500.1330885 MHz
NUC1                     1H
P1                   11.25 usec
PLW1          20.00000000 W

======== CHANNEL f2 ========
SFO2           500.1330885 MHz
NUC2                    off
CPDPRG[2
PCPD2      0 usec
PLW2       0 W
PLW12      0 W
PLW13      0 W

F2 - Processing parameters
SI                    65536
SF           500.1300127 MHz
WDW                      EM
SSB        0
LB                  0.30 Hz
GB         0
PC                  1.00
```

Supplementary Figure 131. ^{1}H-NMR of compound **68**, recorded at 500 MHz and 25 °C in $CDCl_3$.

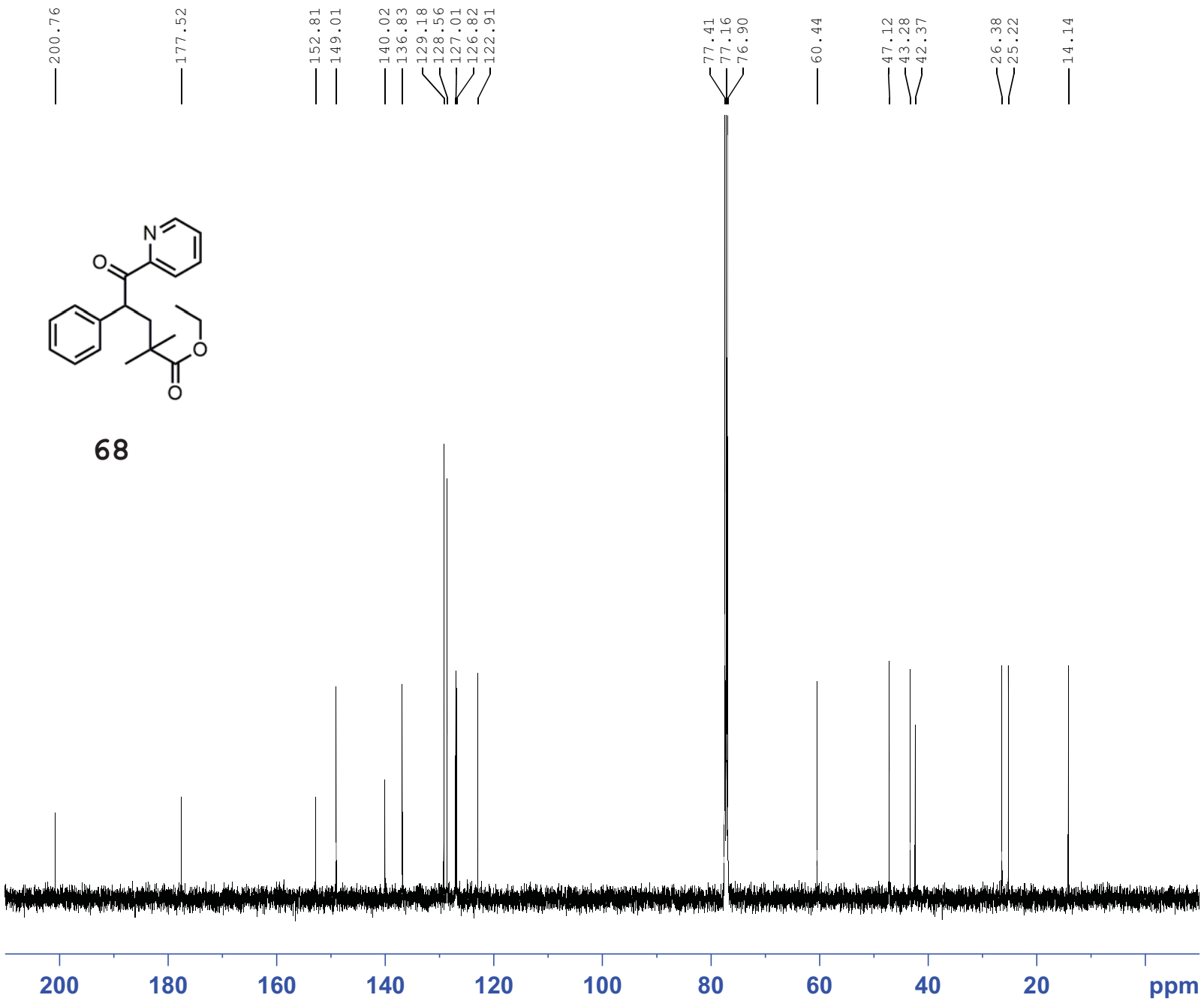


```
Current Data Parameters
NAME                  11156B
EXPNO                      2
PROCNO                     1

F2 - Acquisition Parameters
Date_              20220304
Time                 16.01
INSTRUM              spect
PROBHD    5 mm CPPBBO BB
PULPROG             zgpg30
TD                   65536
SOLVENT              CDCl3
NS                      70
DS                       4
SWH              29761.904 Hz
FIDRES            0.454131 Hz
AQ                1.1010048 sec
RG                  192.89
DW                  16.800 usec
DE                  18.00 usec
TE                  298.2 K
D1              2.00000000 sec
D11             0.03000000 sec
TD0                      1

======== CHANNEL f1 ========
SFO1           125.7703637 MHz
NUC1                    13C
P1                  10.50 usec
PLW1           57.00000000 W

======== CHANNEL f2 ========
SFO2           500.1320005 MHz
NUC2                    1H
CPDPRG[2           waltz16
PCPD2               80.00 usec
PLW2           20.00000000 W
PLW12           0.39550999 W
PLW13           0.25312999 W

F2 - Processing parameters
SI                   32768
SF             125.7577724 MHz
WDW                     EM
SSB          0
LB                   1.00 Hz
GB           0
PC                   1.40
```

Supplementary Figure 132. ^{13}C-NMR of compound **68**, recorded at 126 MHz and 25 °C in $CDCl_3$.

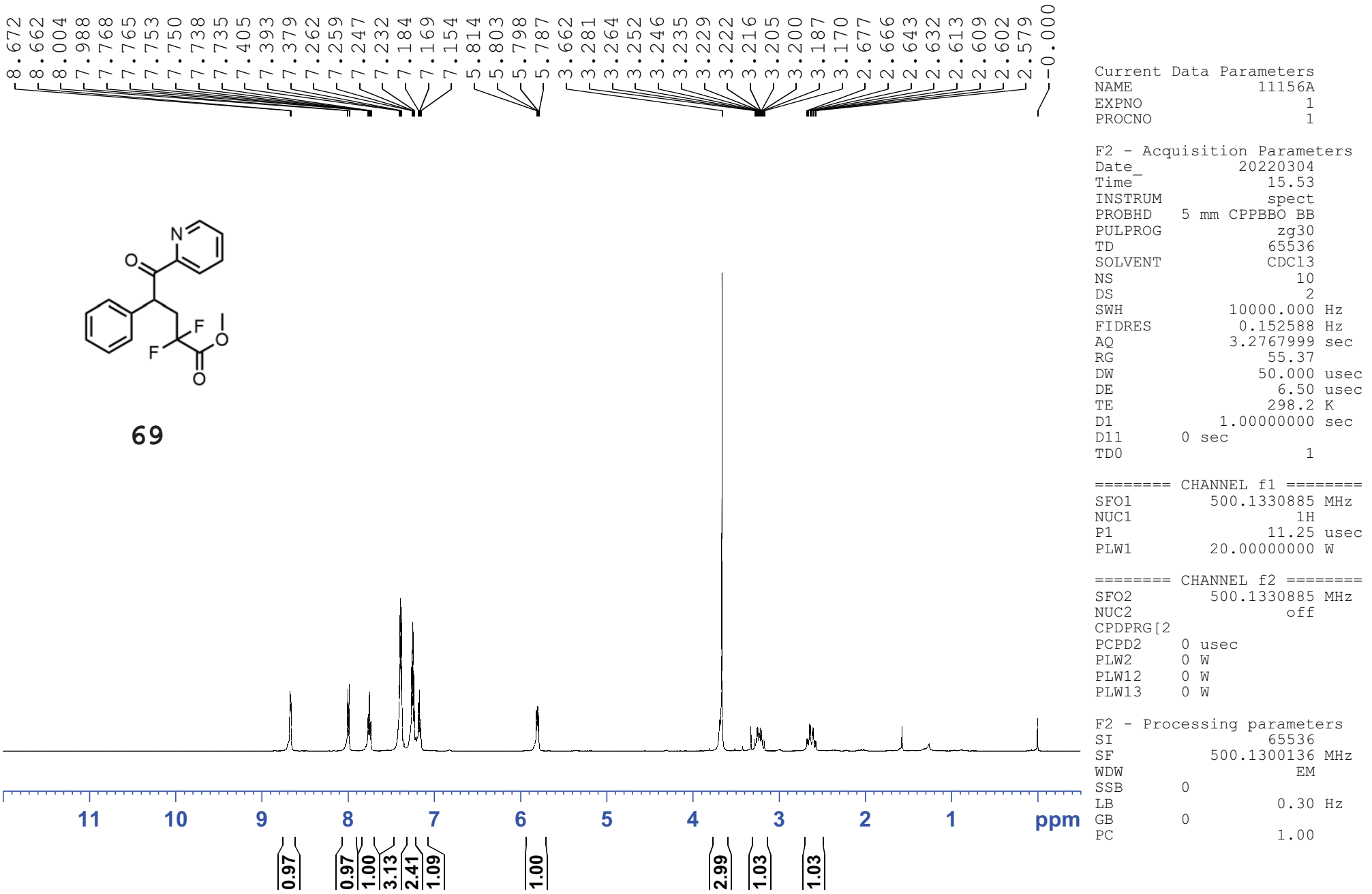


```
Current Data Parameters
NAME                  11156A
EXPNO                      1
PROCNO                     1

F2 - Acquisition Parameters
Date_              20220304
Time                  15.53
INSTRUM               spect
PROBHD    5 mm CPPBBO BB
PULPROG                zg30
TD                    65536
SOLVENT               CDCl3
NS                       10
DS                        2
SWH              10000.000 Hz
FIDRES            0.152588 Hz
AQ               3.2767999 sec
RG                    55.37
DW                   50.000 usec
DE                    6.50 usec
TE                   298.2 K
D1             1.00000000 sec
D11       0 sec
TD0                       1

======== CHANNEL f1 ========
SFO1           500.1330885 MHz
NUC1                      1H
P1                    11.25 usec
PLW1           20.00000000 W

======== CHANNEL f2 ========
SFO2           500.1330885 MHz
NUC2                    off
CPDPRG[2
PCPD2      0 usec
PLW2       0 W
PLW12      0 W
PLW13      0 W

F2 - Processing parameters
SI                    65536
SF           500.1300136 MHz
WDW                      EM
SSB        0
LB                    0.30 Hz
GB         0
PC                    1.00
```

Supplementary Figure 133. ^{1}H-NMR of compound **69**, recorded at 500 MHz and 25 °C in $CDCl_3$.

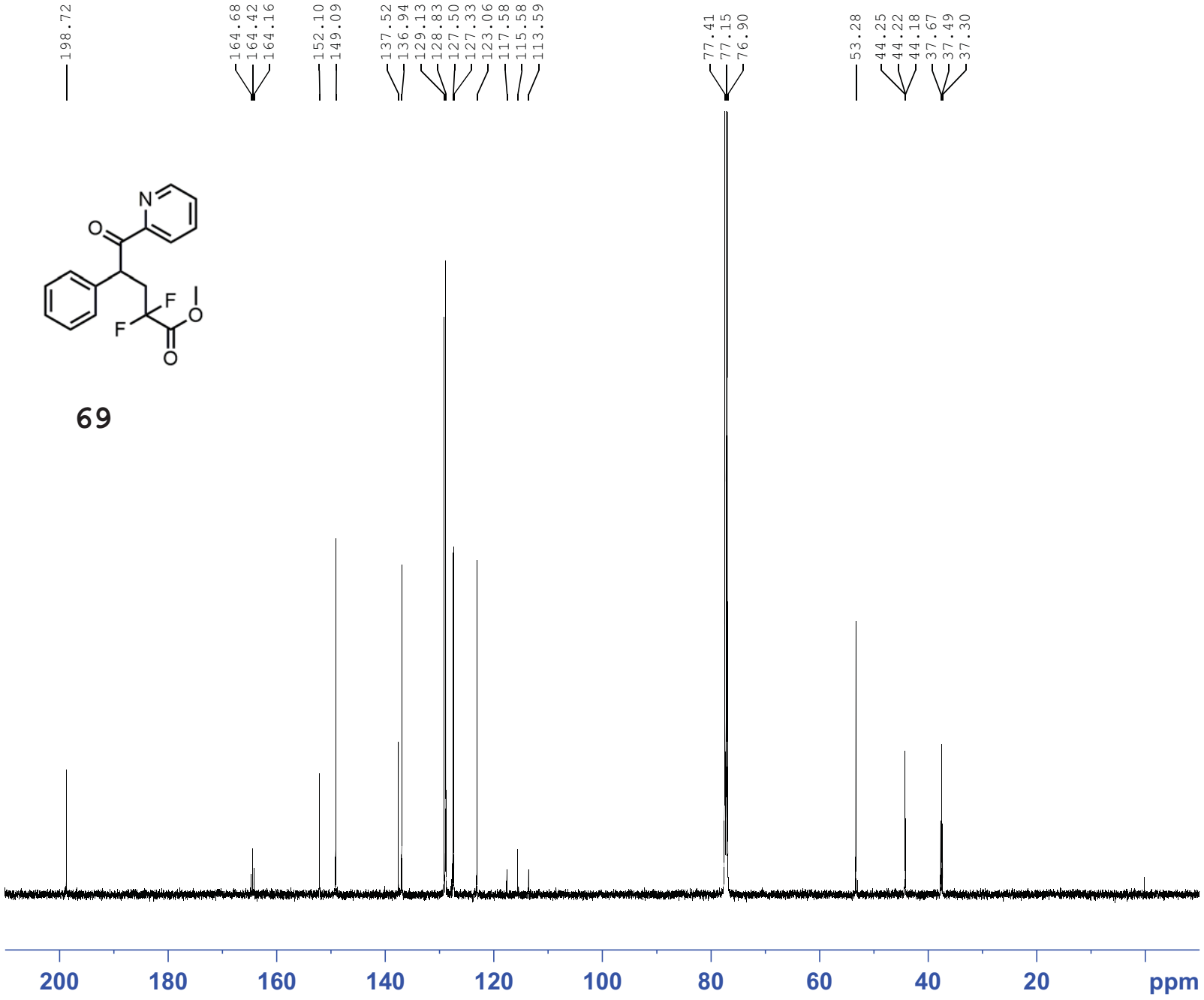


```
Current Data Parameters
NAME                 11156A
EXPNO                     3
PROCNO                    1

F2 - Acquisition Parameters
Date_              20220304
Time                 20.53
INSTRUM              spect
PROBHD    5 mm CPPBBO BB
PULPROG              zgpg30
TD                   65536
SOLVENT              CDCl3
NS                     400
DS                       4
SWH              29761.904 Hz
FIDRES            0.454131 Hz
AQ                1.1010048 sec
RG                  192.89
DW                  16.800 usec
DE                  18.00 usec
TE                  298.2 K
D1              2.00000000 sec
D11             0.03000000 sec
TD0                      1

======== CHANNEL f1 ========
SFO1           125.7703637 MHz
NUC1                   13C
P1                  10.50 usec
PLW1           57.00000000 W

======== CHANNEL f2 ========
SFO2           500.1320005 MHz
NUC2                   1H
CPDPRG[2           waltz16
PCPD2               80.00 usec
PLW2           20.00000000 W
PLW12           0.39550999 W
PLW13           0.25312999 W

F2 - Processing parameters
SI                    32768
SF             125.7577727 MHz
WDW                      EM
SSB          0
LB                   1.00 Hz
GB          0
PC                   1.40
```

Supplementary Figure 134. ^{13}C-NMR of compound **69**, recorded at 126 MHz and 25 °C in $CDCl_3$.

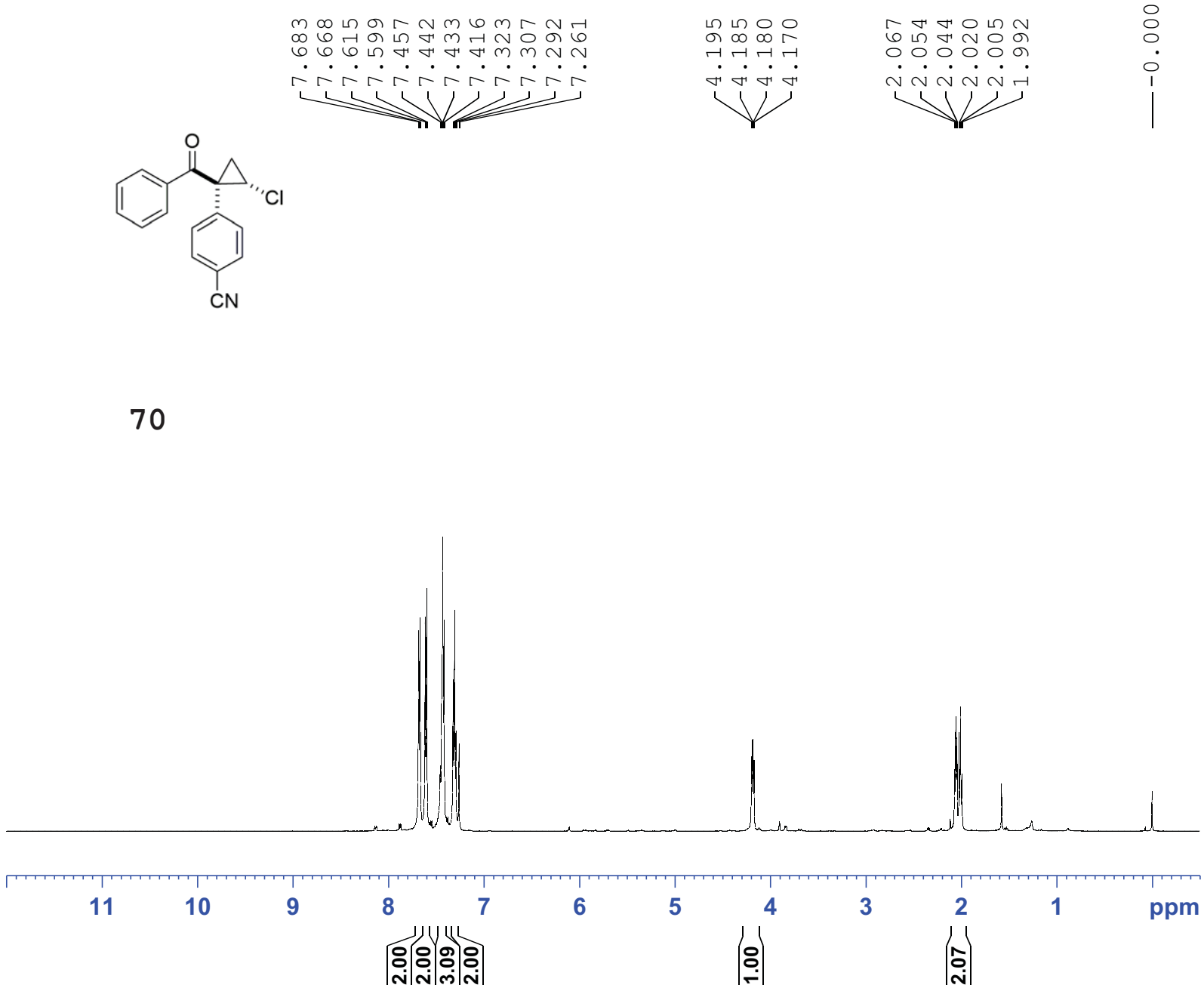

Supplementary Figure 135. ^{1}H-NMR of compound **70**, recorded at 500 MHz and 25 °C in $CDCl_3$.

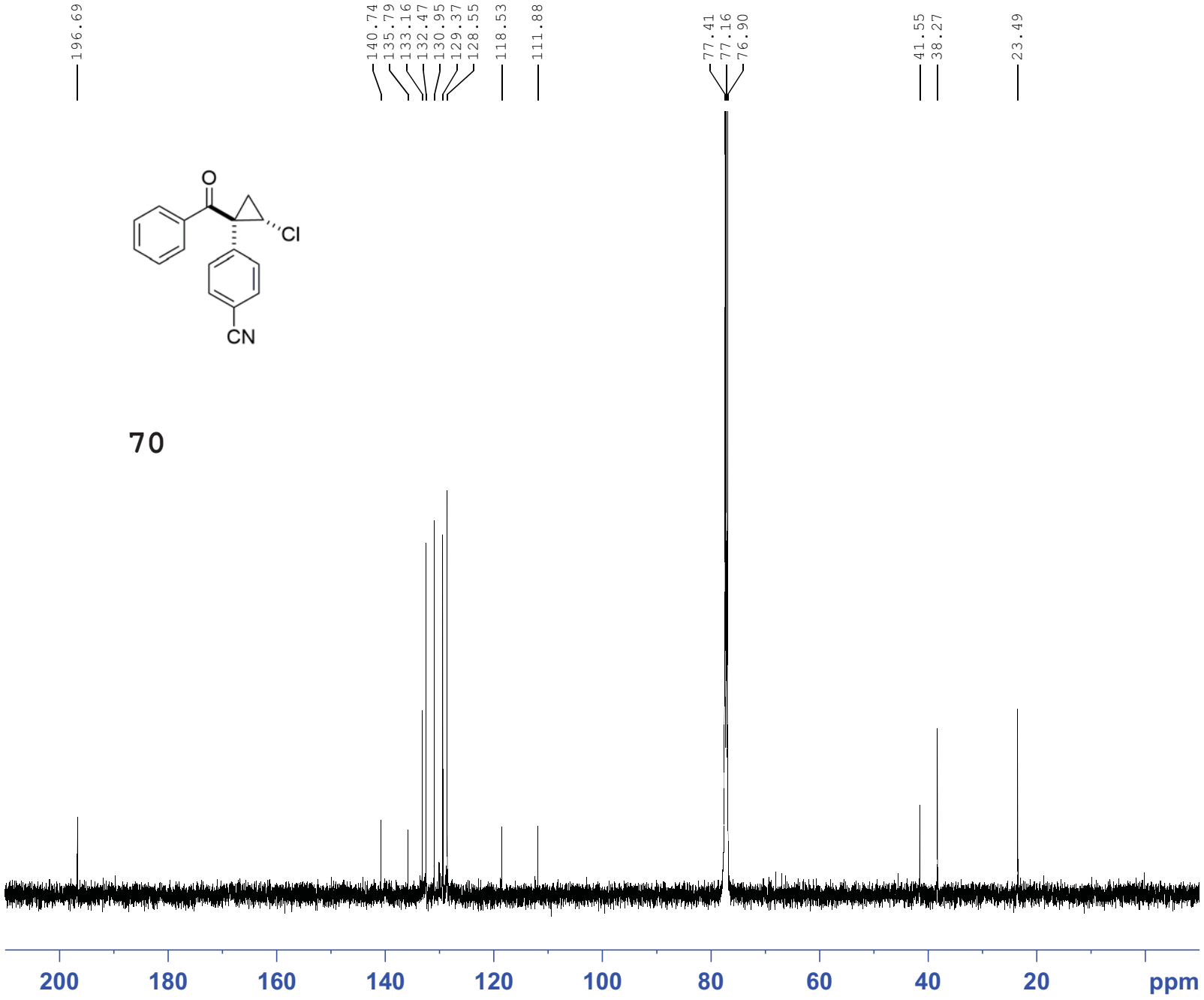


```
Current Data Parameters
NAME                 11199D
EXPNO                     2
PROCNO                    1

F2 - Acquisition Parameters
Date_              20220323
Time                  11.20
INSTRUM               spect
PROBHD   5 mm CPPBBO BB
PULPROG              zgpg30
TD                    65536
SOLVENT               CDCl3
NS                      160
DS                        4
SWH              29761.904 Hz
FIDRES            0.454131 Hz
AQ                1.1010048 sec
RG                   192.89
DW                   16.800 usec
DE                   18.00 usec
TE                   298.2 K
D1              2.00000000 sec
D11             0.03000000 sec
TD0                       1

======== CHANNEL f1 ========
SFO1           125.7703637 MHz
NUC1                    13C
P1                   10.50 usec
PLW1           57.00000000 W

======== CHANNEL f2 ========
SFO2           500.1320005 MHz
NUC2                    1H
CPDPRG[2            waltz16
PCPD2                80.00 usec
PLW2           20.00000000 W
PLW12           0.39550999 W
PLW13           0.25312999 W

F2 - Processing parameters
SI                    32768
SF             125.7577720 MHz
WDW                      EM
SSB          0
LB                    1.00 Hz
GB           0
PC                    1.40
```

Supplementary Figure 136. ^{13}C-NMR of compound **70**, recorded at 126 MHz and 25 °C in $CDCl_3$.

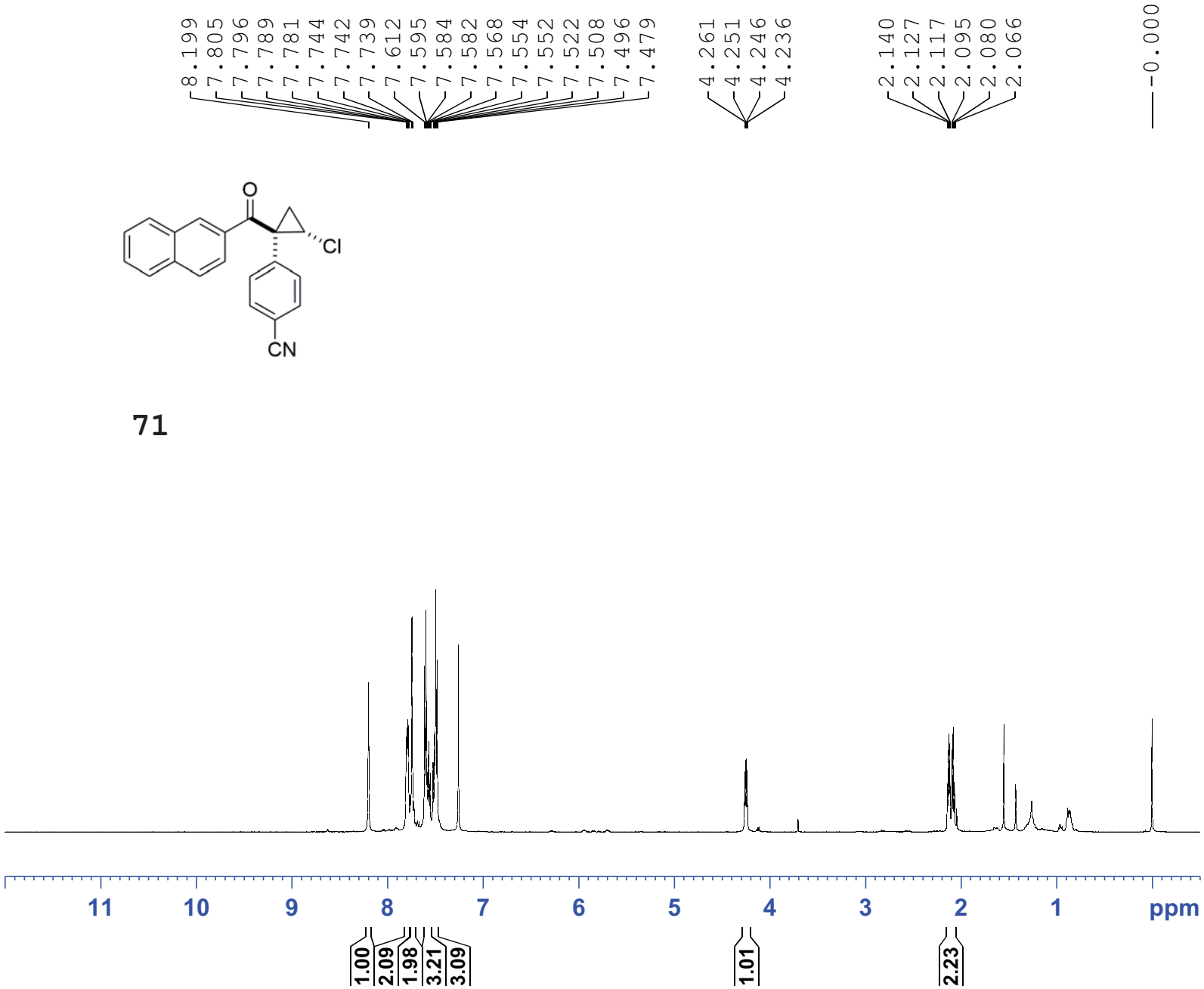


```
Current Data Parameters
NAME                  11193C-
EXPNO                       1
PROCNO                      1

F2 - Acquisition Parameters
Date_               20220320
Time                   11.53
INSTRUM                spect
PROBHD    5 mm CPPBBO BB
PULPROG                 zg30
TD                     65536
SOLVENT                CDCl3
NS                        16
DS                         2
SWH              10000.000 Hz
FIDRES            0.152588 Hz
AQ               3.2767999 sec
RG                     62.06
DW                    50.000 usec
DE                     6.50 usec
TE                    298.2 K
D1             1.00000000 sec
D11       0 sec
TD0                        1

======== CHANNEL f1 ========
SFO1           500.1330885 MHz
NUC1                     1H
P1                    11.25 usec
PLW1          20.00000000 W

======== CHANNEL f2 ========
SFO2           500.1330885 MHz
NUC2                    off
CPDPRG[2
PCPD2      0 usec
PLW2       0 W
PLW12      0 W
PLW13      0 W

F2 - Processing parameters
SI                     65536
SF             500.1300137 MHz
WDW                       EM
SSB        0
LB                    0.30 Hz
GB         0
PC                    1.00
```

Supplementary Figure 137. ^{1}H-NMR of compound **71**, recorded at 500 MHz and 25 °C in $CDCl_3$.

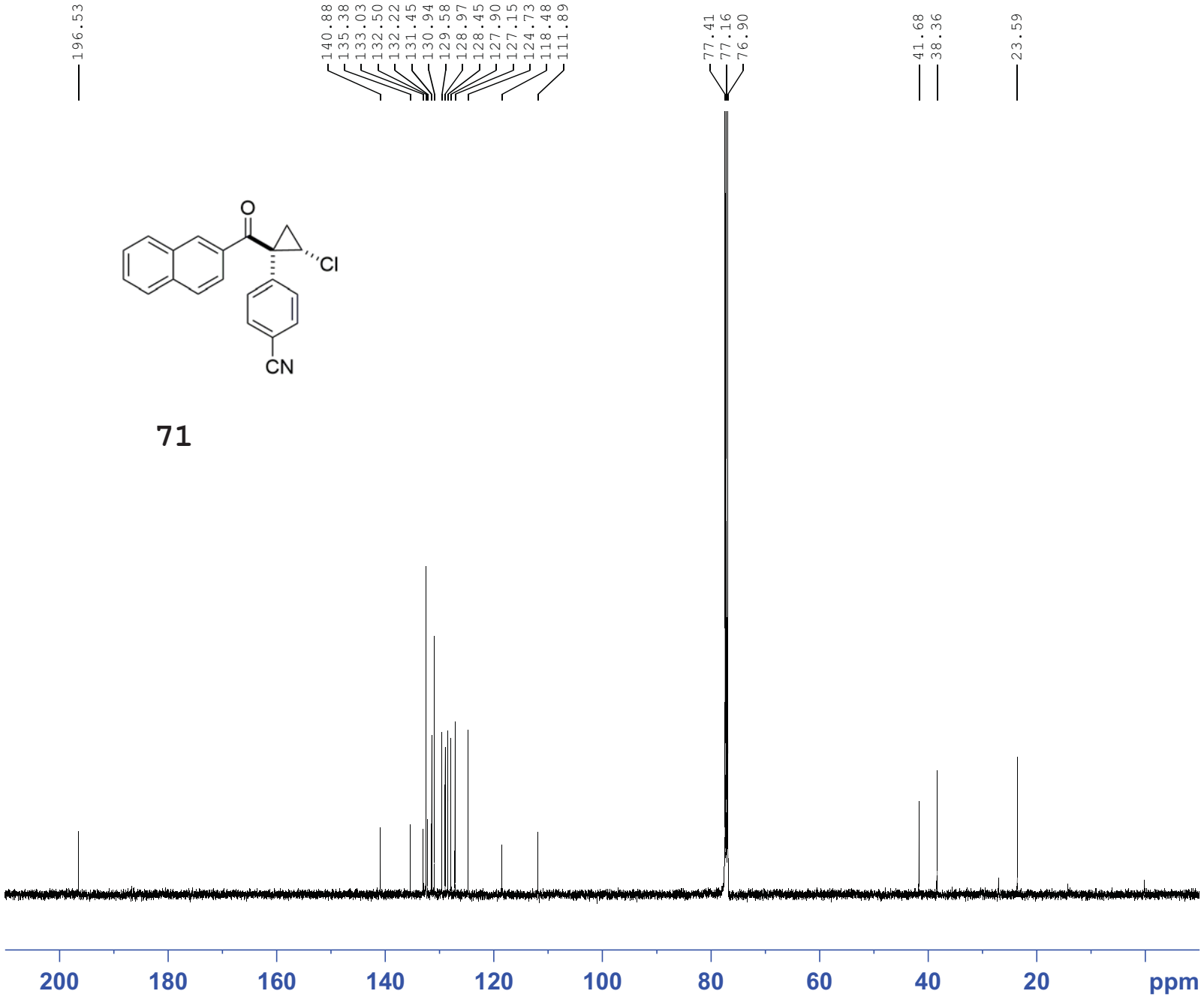


```
Current Data Parameters
NAME                 11193C-
EXPNO                      3
PROCNO                     1

F2 - Acquisition Parameters
Date_               20220320
Time                   12.10
INSTRUM                spect
PROBHD   5 mm CPPBBO BB
PULPROG              zgpg30
TD                     65536
SOLVENT                CDCl3
NS                       200
DS                         4
SWH                29761.904 Hz
FIDRES             0.454131 Hz
AQ                 1.1010048 sec
RG                    192.89
DW                    16.800 usec
DE                    18.00 usec
TE                    298.2 K
D1               2.00000000 sec
D11              0.03000000 sec
TD0                        1

======== CHANNEL f1 ========
SFO1            125.7703637 MHz
NUC1                    13C
P1                    10.50 usec
PLW1            57.00000000 W

======== CHANNEL f2 ========
SFO2            500.1320005 MHz
NUC2                    1H
CPDPRG[2             waltz16
PCPD2                 80.00 usec
PLW2            20.00000000 W
PLW12            0.39550999 W
PLW13            0.25312999 W

F2 - Processing parameters
SI                     32768
SF              125.7577729 MHz
WDW                       EM
SSB          0
LB                     1.00 Hz
GB           0
PC                     1.40
```

Supplementary Figure 138. ^{13}C-NMR of compound **71**, recorded at 126 MHz and 25 °C in $CDCl_3$.

11193C-2
1D Selective Gradient NOESY
freq: 4.274ppm

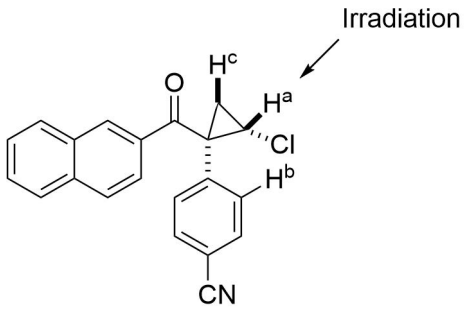

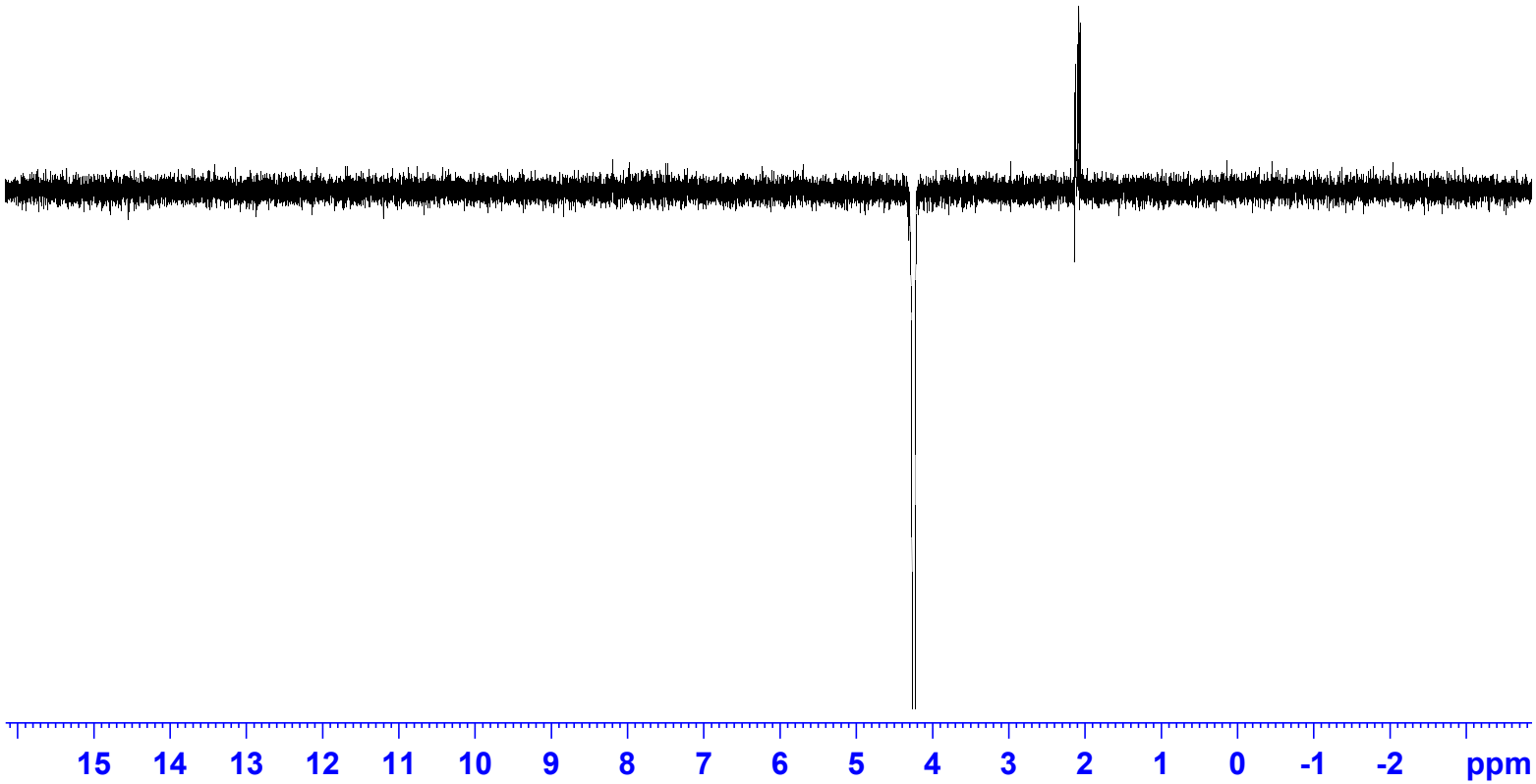


```
Current Data Parameters
NAME            11193C-2
EXPNO                  3
PROCNO                 1

F2 - Acquisition Parameters
Date_           20220407
Time              19.02
INSTRUM           spect
PROBHD   5 mm PABBO BB/
PULPROG         selnogp
TD                65536
SOLVENT           CDCl3
NS                   32
DS                    2
SWH            8012.820 Hz
FIDRES         0.122266 Hz
AQ             4.0894465 sec
RG                206.33
DW                62.400 usec
DE                 6.50 usec
TE                301.2 K
D1           2.00000000 sec
D11      0 sec
TD0                    1

======== CHANNEL f1 ========
SFO1         400.2424716 MHz
NUC1                   1H
P1                14.30 usec
PLW1         12.00000000 W

======== CHANNEL f2 ========
SFO2         400.2417780 MHz
NUC2                  off
CPDPRG[2
PCPD2     0 usec
PLW2      0 W
PLW12     0 W
PLW13     0 W

F2 - Processing parameters
SI                 32768
SF           400.2400112 MHz
WDW                   EM
SSB       0
LB                 0.10 Hz
GB        0
PC                 1.00
```

Supplementary Figure 139. NOESY of compound 71, recorded at 400 MHz and 25 °C in CDCl3

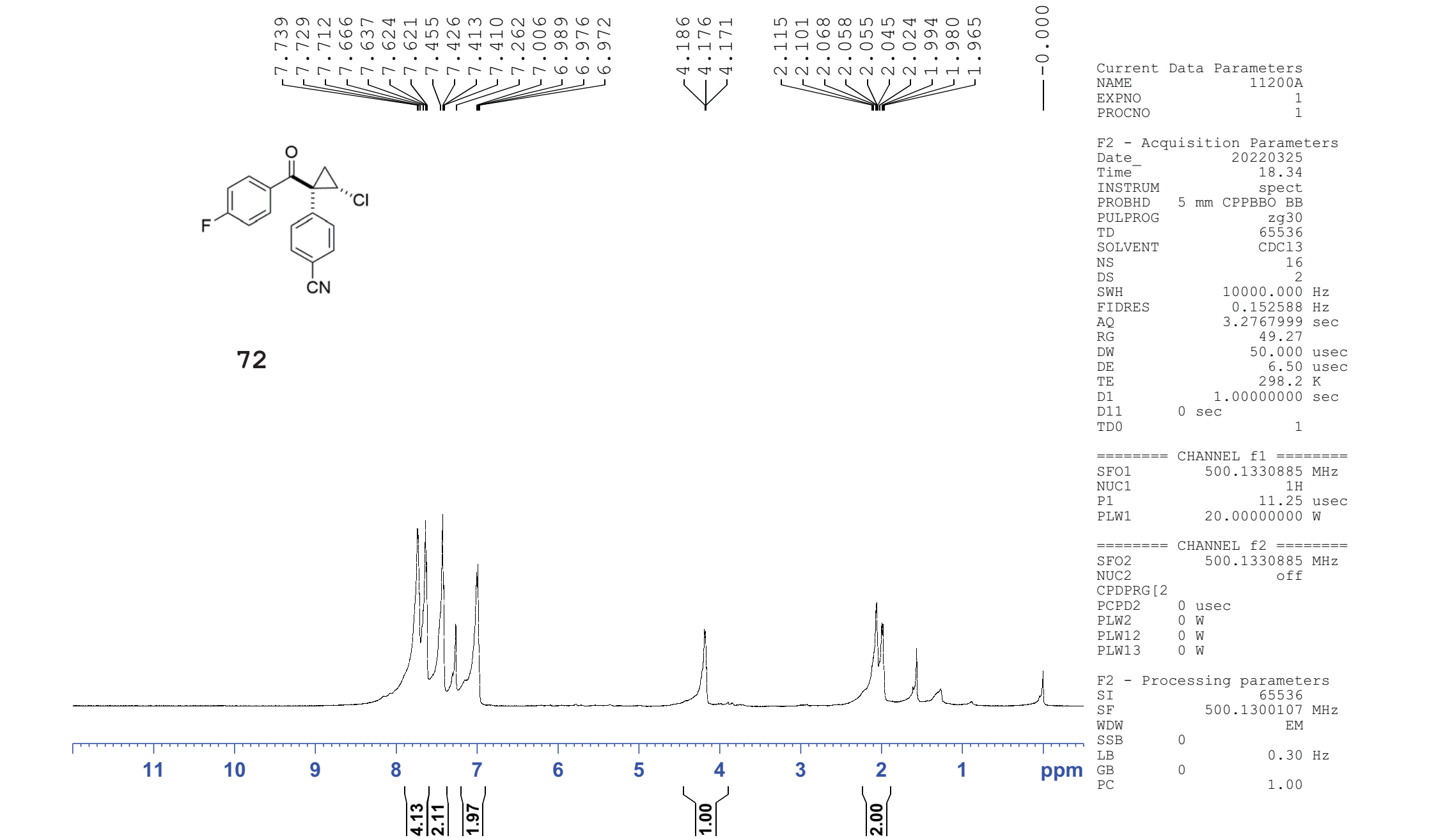

Supplementary Figure 140. ^{1}H-NMR of compound **72**, recorded at 500 MHz and 25 °C in $CDCl_3$.

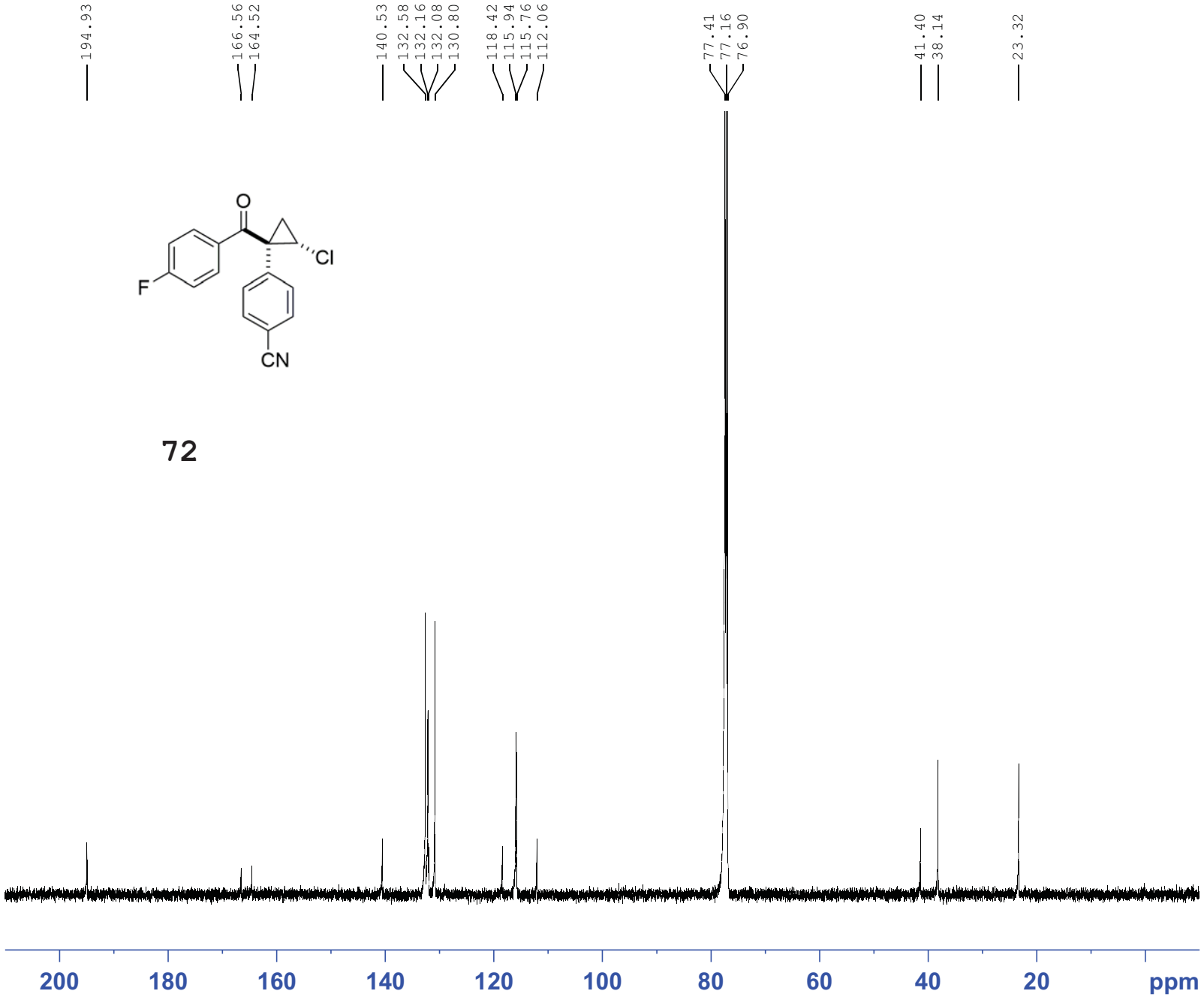


```
Current Data Parameters
NAME                 11200A
EXPNO                     2
PROCNO                    1

F2 - Acquisition Parameters
Date_              20220325
Time                 19.11
INSTRUM              spect
PROBHD   5 mm CPPBBO BB
PULPROG              zgpg30
TD                    65536
SOLVENT               CDCl3
NS                      700
DS                        4
SWH              29761.904 Hz
FIDRES            0.454131 Hz
AQ               1.1010048 sec
RG                   192.89
DW                  16.800 usec
DE                  18.00 usec
TE                  298.2 K
D1              2.00000000 sec
D11             0.03000000 sec
TD0                       1

======== CHANNEL f1 ========
SFO1           125.7703637 MHz
NUC1                    13C
P1                   10.50 usec
PLW1           57.00000000 W

======== CHANNEL f2 ========
SFO2           500.1320005 MHz
NUC2                    1H
CPDPRG[2            waltz16
PCPD2                80.00 usec
PLW2           20.00000000 W
PLW12           0.39550999 W
PLW13           0.25312999 W

F2 - Processing parameters
SI                    32768
SF             125.7577729 MHz
WDW                      EM
SSB          0
LB                    1.00 Hz
GB           0
PC                    1.40
```

Supplementary Figure 141. ^{13}C-NMR of compound **72**, recorded at 126 MHz and 25 °C in $CDCl_3$.

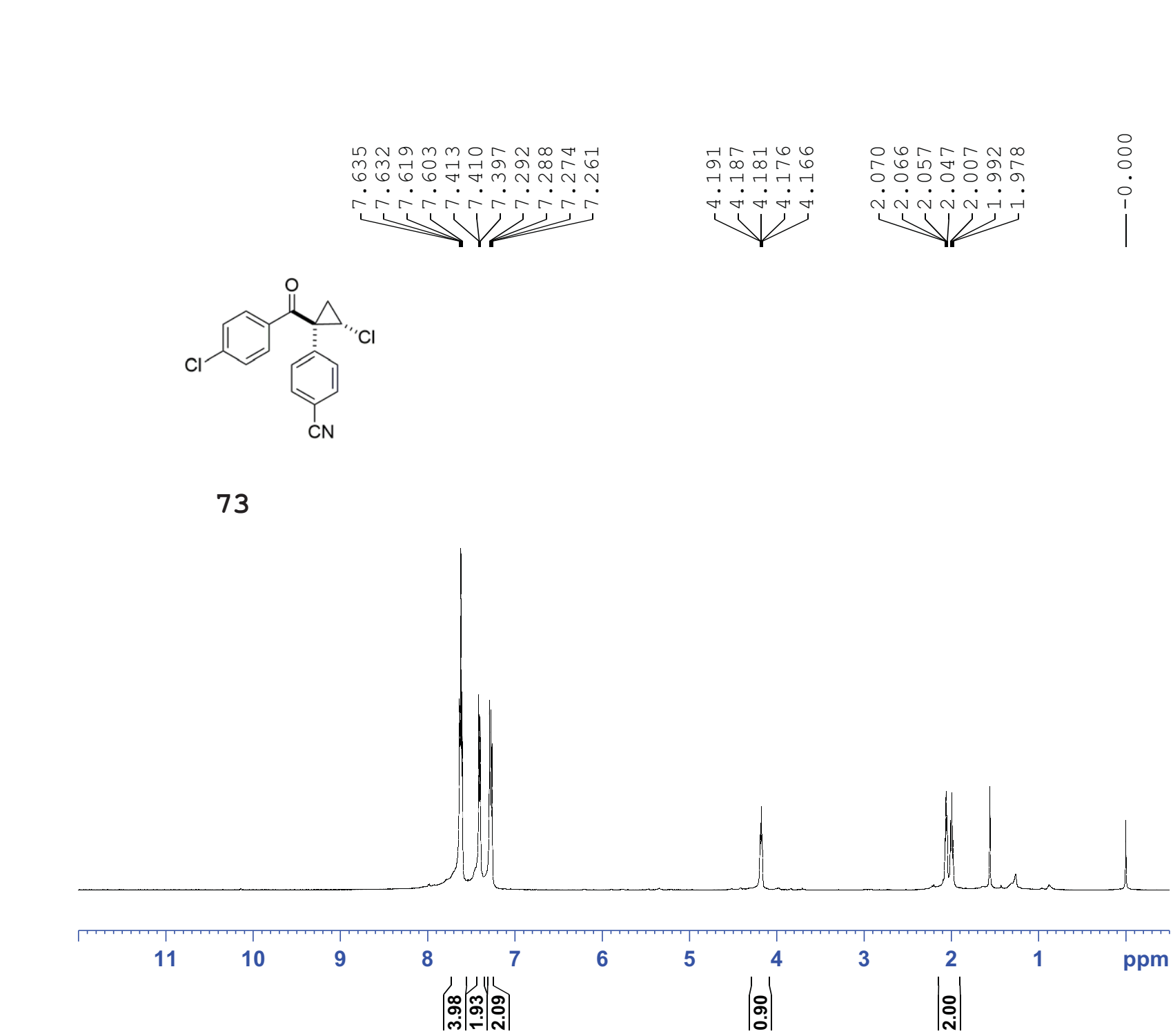


```
Current Data Parameters
NAME                  11199F
EXPNO                      1
PROCNO                     1

F2 - Acquisition Parameters
Date_              20220323
Time                  11.32
INSTRUM               spect
PROBHD    5 mm CPPBBO BB
PULPROG                zg30
TD                    65536
SOLVENT               CDCl3
NS                       12
DS                        2
SWH              10000.000 Hz
FIDRES               0.152588 Hz
AQ               3.2767999 sec
RG                    62.06
DW                   50.000 usec
DE                    6.50 usec
TE                   298.2 K
D1             1.00000000 sec
D11       0 sec
TD0                       1

======== CHANNEL f1 ========
SFO1           500.1330885 MHz
NUC1                     1H
P1                   11.25 usec
PLW1          20.00000000 W

======== CHANNEL f2 ========
SFO2           500.1330885 MHz
NUC2                   off
CPDPRG[2
PCPD2      0 usec
PLW2       0 W
PLW12      0 W
PLW13      0 W

F2 - Processing parameters
SI                    65536
SF           500.1300131 MHz
WDW                      EM
SSB        0
LB                    0.30 Hz
GB         0
PC                    1.00
```

Supplementary Figure 142. ^{1}H-NMR of compound **73**, recorded at 500 MHz and 25 °C in $CDCl_3$.

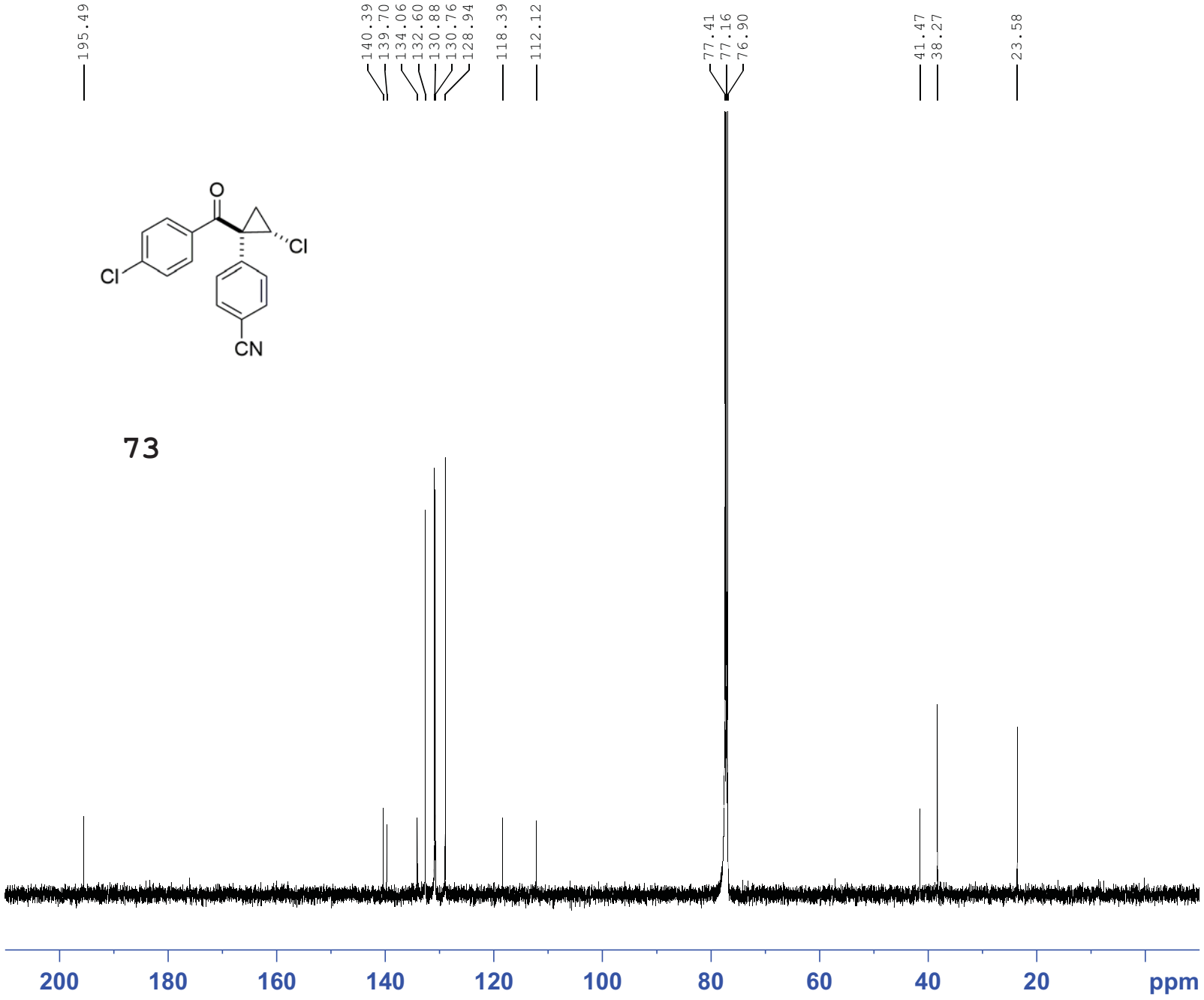


```
Current Data Parameters
NAME                 11199F
EXPNO                     2
PROCNO                    1

F2 - Acquisition Parameters
Date_              20220323
Time                  11.34
INSTRUM               spect
PROBHD   5 mm CPPBBO BB
PULPROG              zgpg30
TD                    65536
SOLVENT               CDCl3
NS                      160
DS                        4
SWH              29761.904 Hz
FIDRES            0.454131 Hz
AQ                1.1010048 sec
RG                   192.89
DW                   16.800 usec
DE                   18.00 usec
TE                   298.2 K
D1              2.00000000 sec
D11             0.03000000 sec
TD0                       1

======== CHANNEL f1 ========
SFO1           125.7703637 MHz
NUC1                    13C
P1                   10.50 usec
PLW1           57.00000000 W

======== CHANNEL f2 ========
SFO2           500.1320005 MHz
NUC2                    1H
CPDPRG[2            waltz16
PCPD2                80.00 usec
PLW2           20.00000000 W
PLW12           0.39550999 W
PLW13           0.25312999 W

F2 - Processing parameters
SI                      32768
SF             125.7577729 MHz
WDW                        EM
SSB          0
LB                    1.00 Hz
GB           0
PC                    1.40
```

Supplementary Figure 143. ^{13}C-NMR of compound **73**, recorded at 126 MHz and 25 °C in $CDCl_3$.

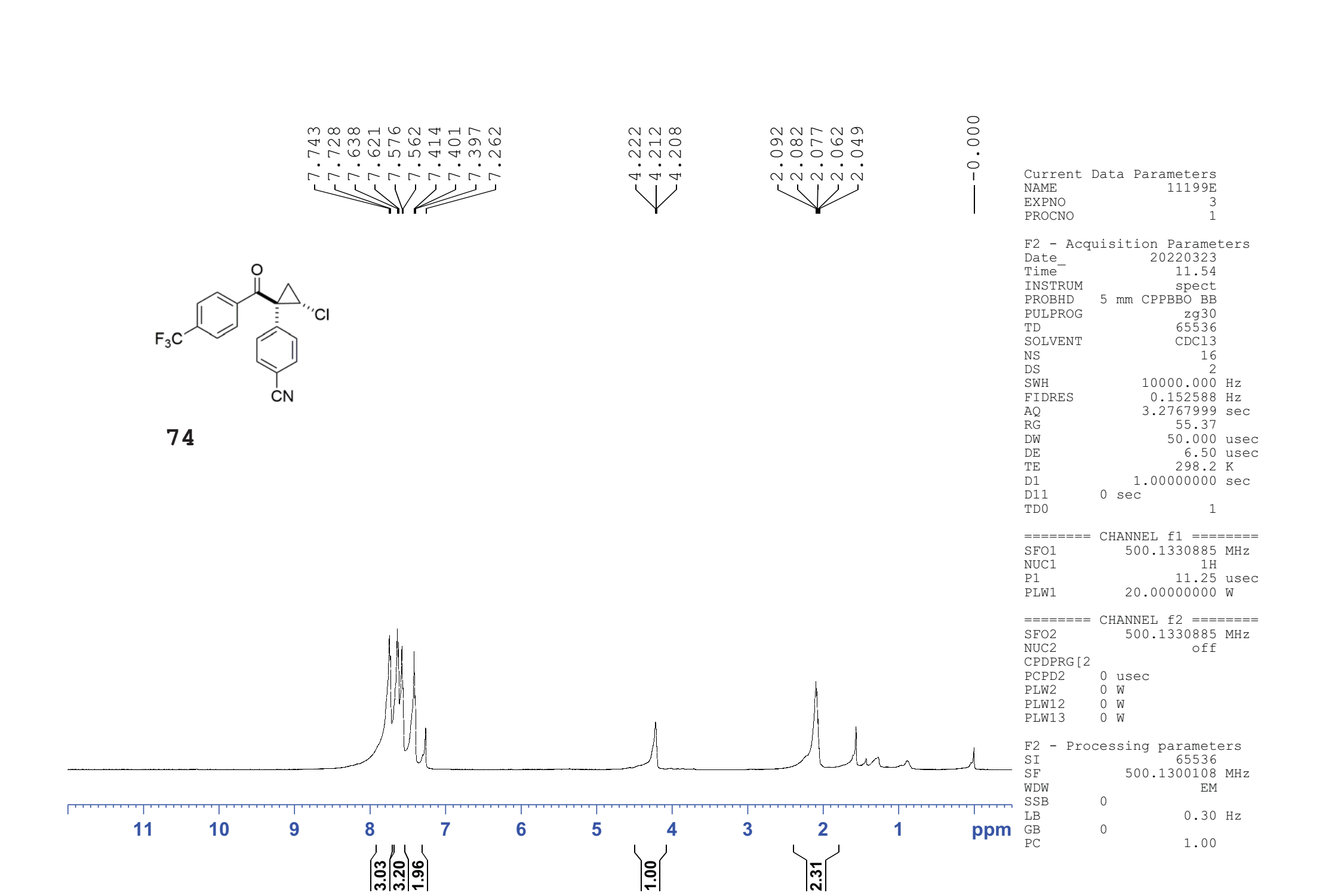

Supplementary Figure 144. ^{1}H-NMR of compound **74**, recorded at 500 MHz and 25 °C in $CDCl_3$.

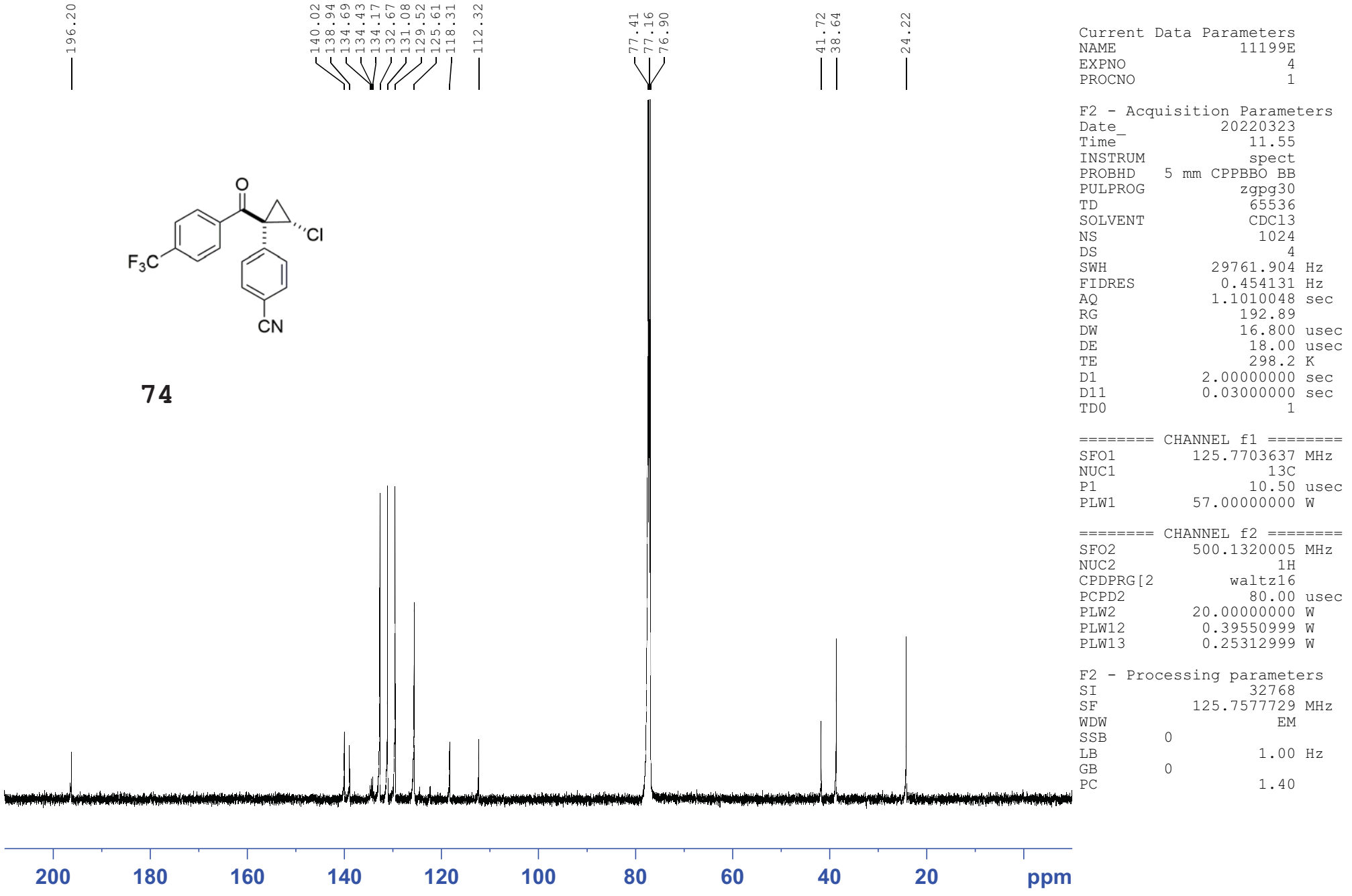


```
Current Data Parameters
NAME                 11199E
EXPNO                     4
PROCNO                    1

F2 - Acquisition Parameters
Date_              20220323
Time                  11.55
INSTRUM               spect
PROBHD   5 mm CPPBBO BB
PULPROG             zgpg30
TD                   65536
SOLVENT              CDCl3
NS                    1024
DS                       4
SWH              29761.904 Hz
FIDRES            0.454131 Hz
AQ                1.1010048 sec
RG                  192.89
DW                  16.800 usec
DE                  18.00 usec
TE                  298.2 K
D1              2.00000000 sec
D11             0.03000000 sec
TD0                       1

======== CHANNEL f1 ========
SFO1           125.7703637 MHz
NUC1                   13C
P1                  10.50 usec
PLW1           57.00000000 W

======== CHANNEL f2 ========
SFO2           500.1320005 MHz
NUC2                   1H
CPDPRG[2           waltz16
PCPD2               80.00 usec
PLW2           20.00000000 W
PLW12           0.39550999 W
PLW13           0.25312999 W

F2 - Processing parameters
SI                    32768
SF             125.7577729 MHz
WDW                      EM
SSB          0
LB                   1.00 Hz
GB          0
PC                   1.40
```

Supplementary Figure 145. ^{13}C-NMR of compound **74**, recorded at 126 MHz and 25 °C in $CDCl_3$.

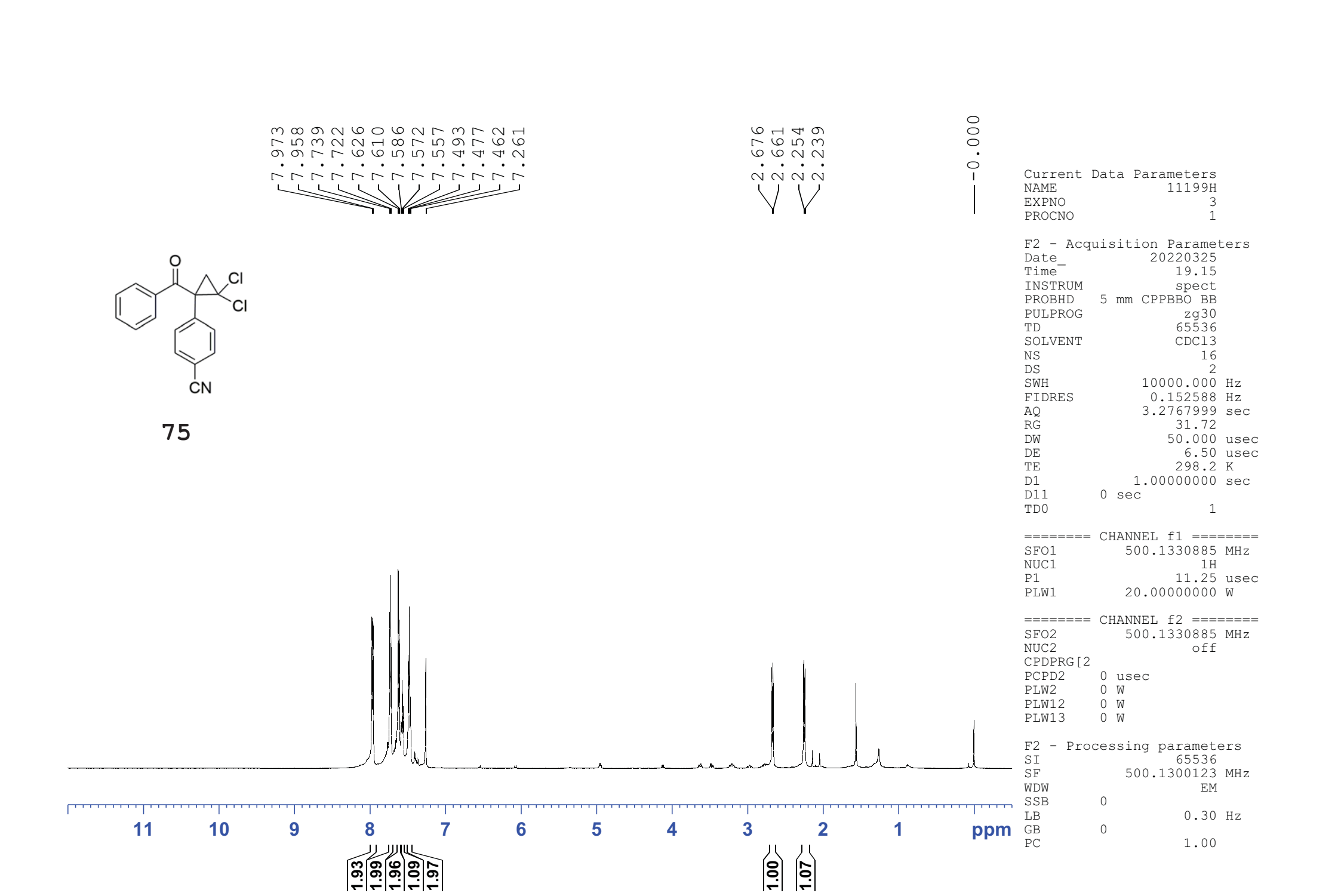

Supplementary Figure 146. ^{1}H-NMR of compound **75**, recorded at 500 MHz and 25 °C in $CDCl_3$.

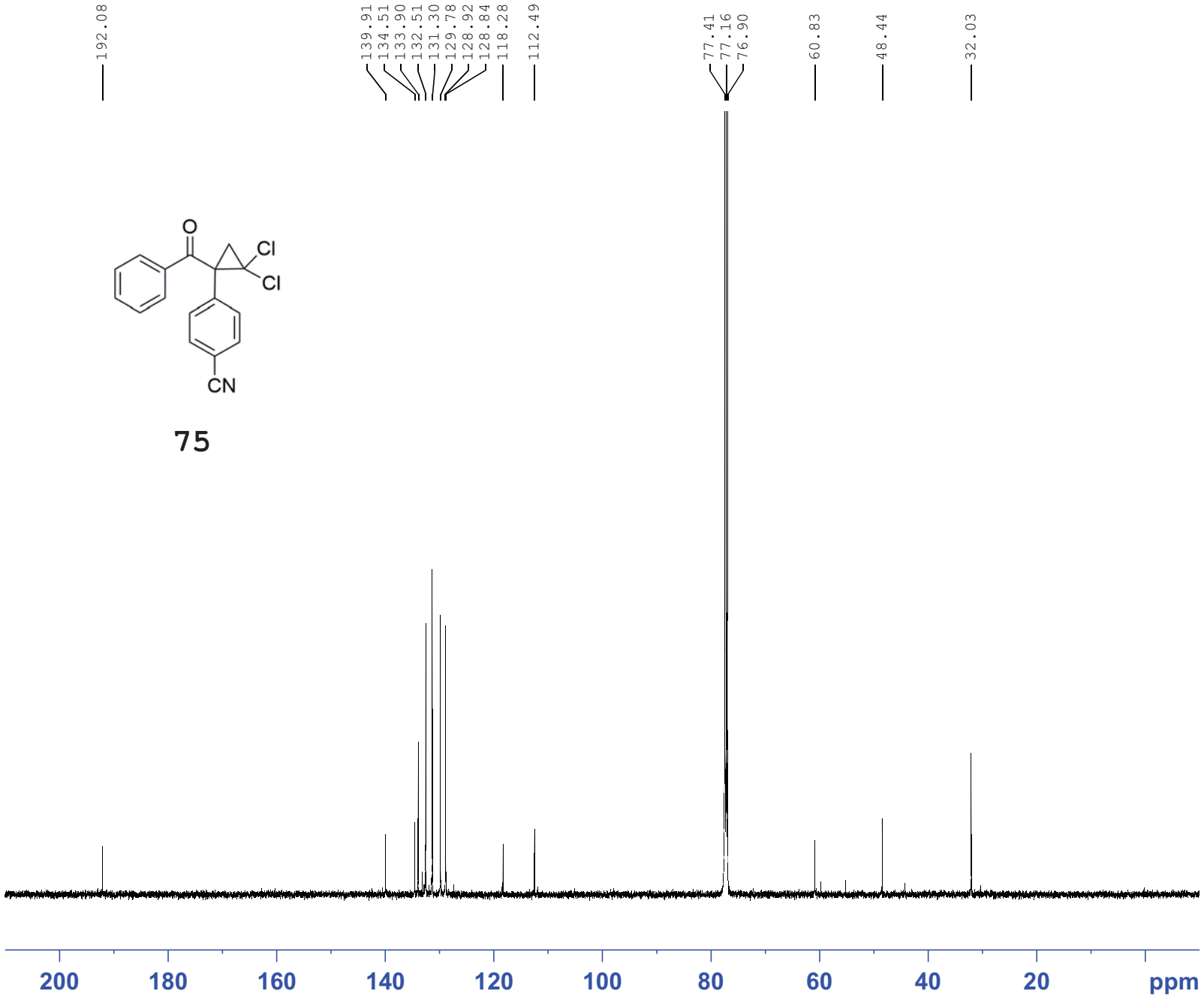


```
Current Data Parameters
NAME                 11199H
EXPNO                     4
PROCNO                    1

F2 - Acquisition Parameters
Date_              20220325
Time                  19.32
INSTRUM               spect
PROBHD   5 mm CPPBBO BB
PULPROG              zgpg30
TD                    65536
SOLVENT               CDCl3
NS                      300
DS                        4
SWH              29761.904 Hz
FIDRES            0.454131 Hz
AQ               1.1010048 sec
RG                   192.89
DW                   16.800 usec
DE                   18.00 usec
TE                   298.2 K
D1             2.00000000 sec
D11            0.03000000 sec
TD0                       1

======== CHANNEL f1 ========
SFO1           125.7703637 MHz
NUC1                    13C
P1                   10.50 usec
PLW1           57.00000000 W

======== CHANNEL f2 ========
SFO2           500.1320005 MHz
NUC2                    1H
CPDPRG[2           waltz16
PCPD2                80.00 usec
PLW2           20.00000000 W
PLW12           0.39550999 W
PLW13           0.25312999 W

F2 - Processing parameters
SI                    32768
SF             125.7577728 MHz
WDW                      EM
SSB       0
LB                    1.00 Hz
GB        0
PC                    1.40
```

Supplementary Figure 147. ^{13}C-NMR of compound **75**, recorded at 126 MHz and 25 °C in $CDCl_3$.

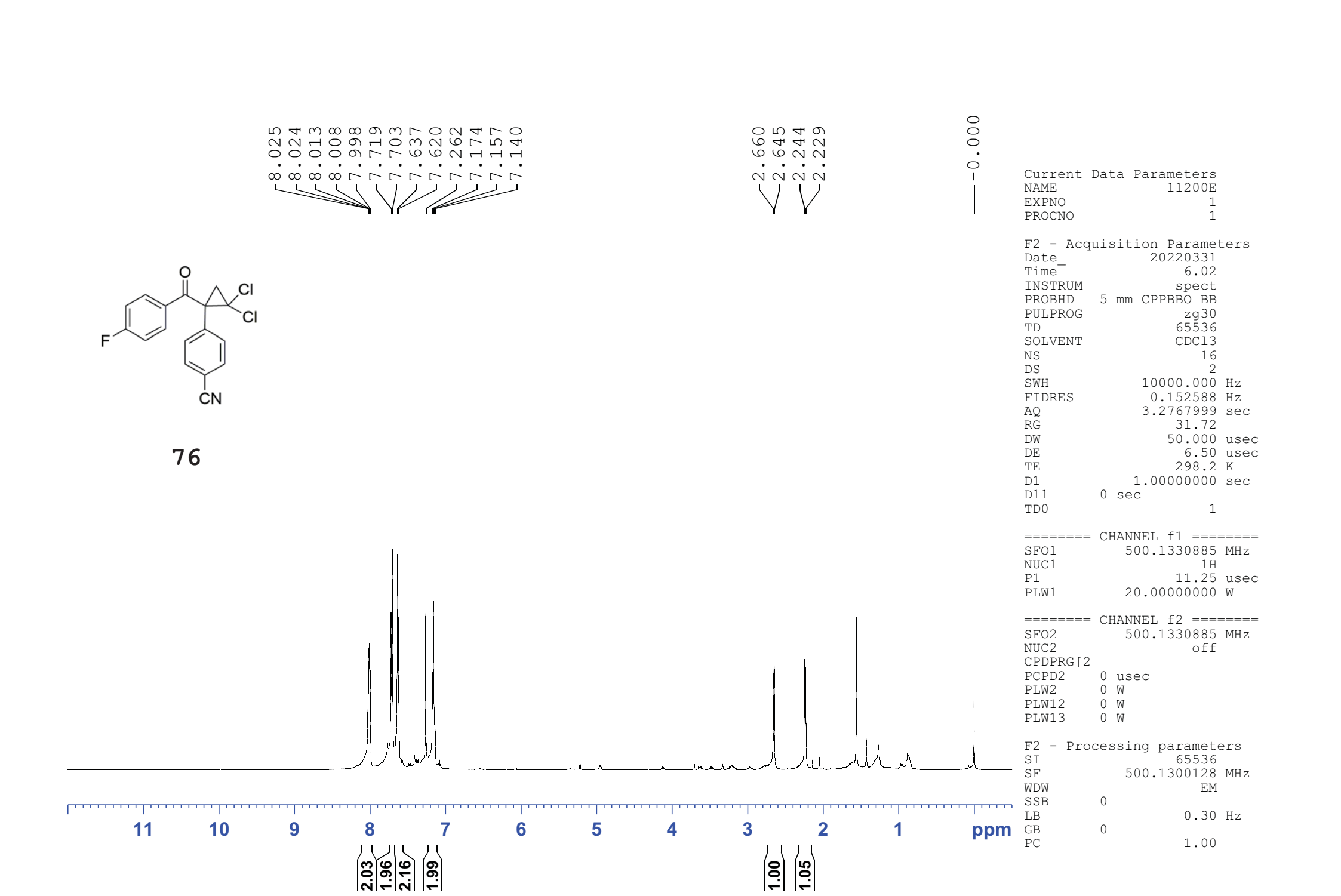

Supplementary Figure 148. ^{1}H-NMR of compound **76**, recorded at 500 MHz and 25 °C in $CDCl_3$.

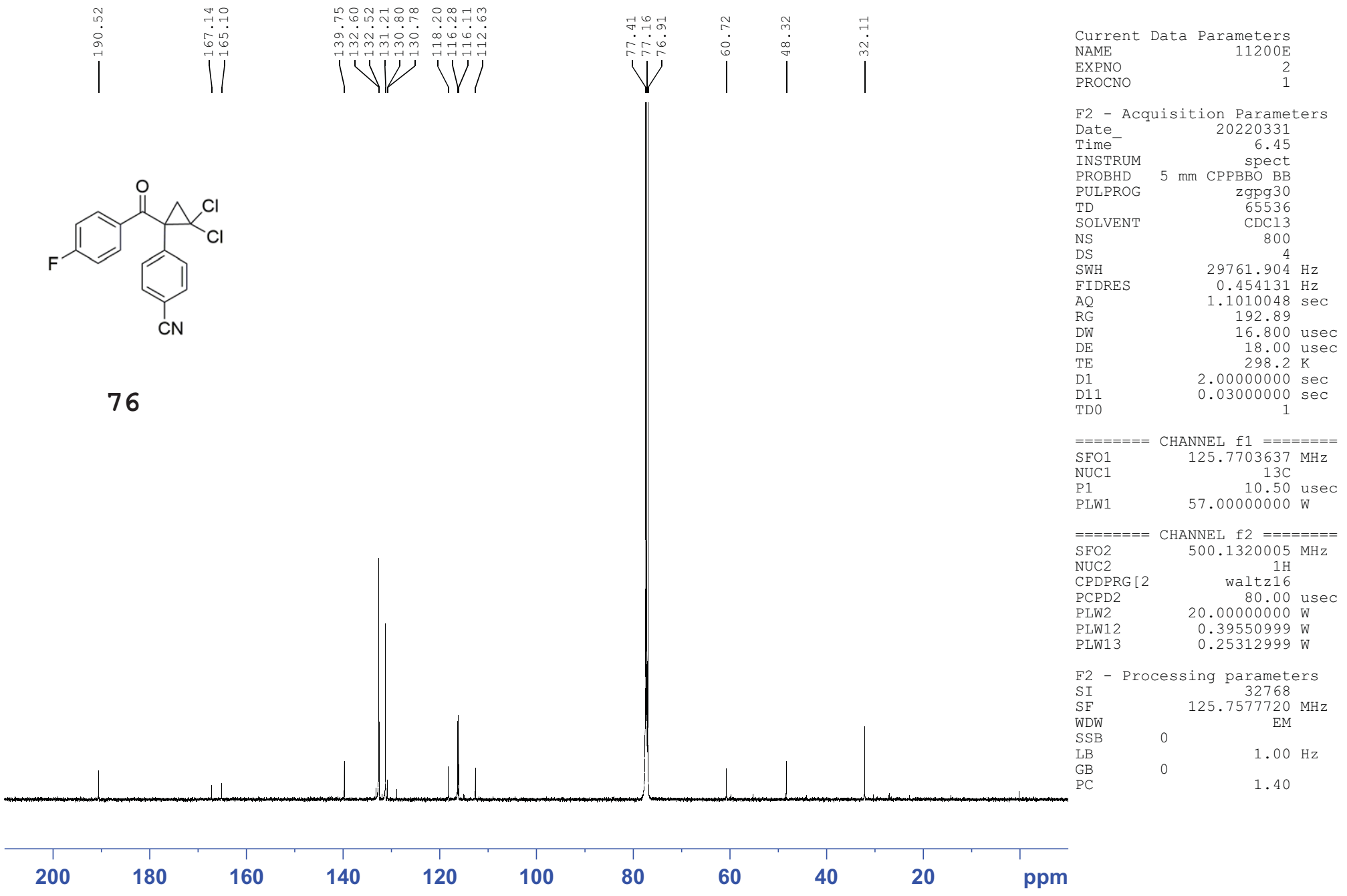


```
Current Data Parameters
NAME                 11200E
EXPNO                     2
PROCNO                    1

F2 - Acquisition Parameters
Date_              20220331
Time                   6.45
INSTRUM               spect
PROBHD   5 mm CPPBBO BB
PULPROG              zgpg30
TD                    65536
SOLVENT               CDCl3
NS                      800
DS                        4
SWH              29761.904 Hz
FIDRES            0.454131 Hz
AQ               1.1010048 sec
RG                   192.89
DW                   16.800 usec
DE                   18.00 usec
TE                   298.2 K
D1              2.00000000 sec
D11             0.03000000 sec
TD0                       1

======== CHANNEL f1 ========
SFO1           125.7703637 MHz
NUC1                    13C
P1                   10.50 usec
PLW1           57.00000000 W

======== CHANNEL f2 ========
SFO2           500.1320005 MHz
NUC2                    1H
CPDPRG[2            waltz16
PCPD2                80.00 usec
PLW2           20.00000000 W
PLW12           0.39550999 W
PLW13           0.25312999 W

F2 - Processing parameters
SI                    32768
SF             125.7577720 MHz
WDW                      EM
SSB          0
LB                    1.00 Hz
GB           0
PC                    1.40
```

Supplementary Figure 149. ^{13}C-NMR of compound **76**, recorded at 126 MHz and 25 °C in $CDCl_3$.

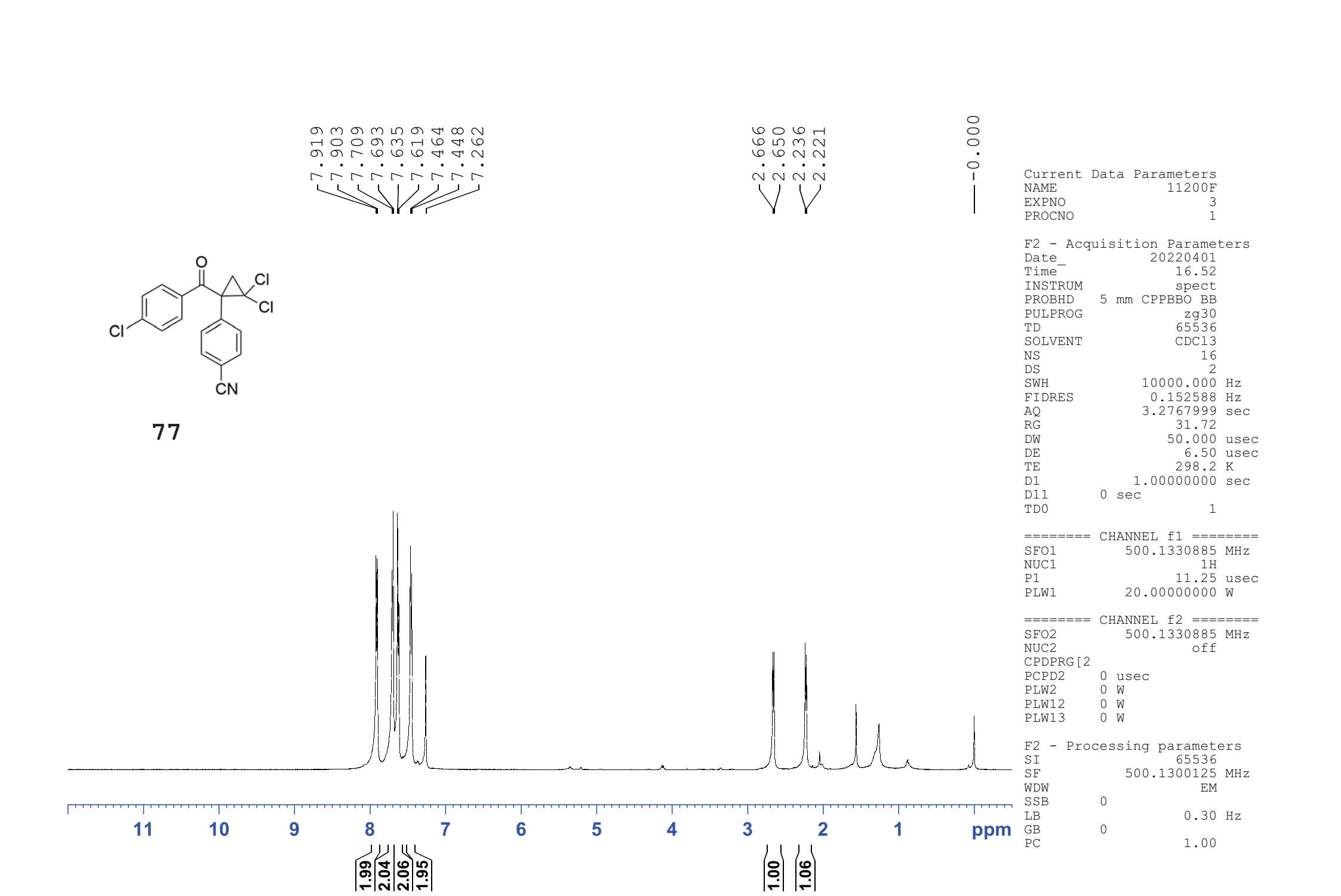

Supplementary Figure 150. ^{1}H-NMR of compound **77**, recorded at 500 MHz and 25 °C in $CDCl_3$.

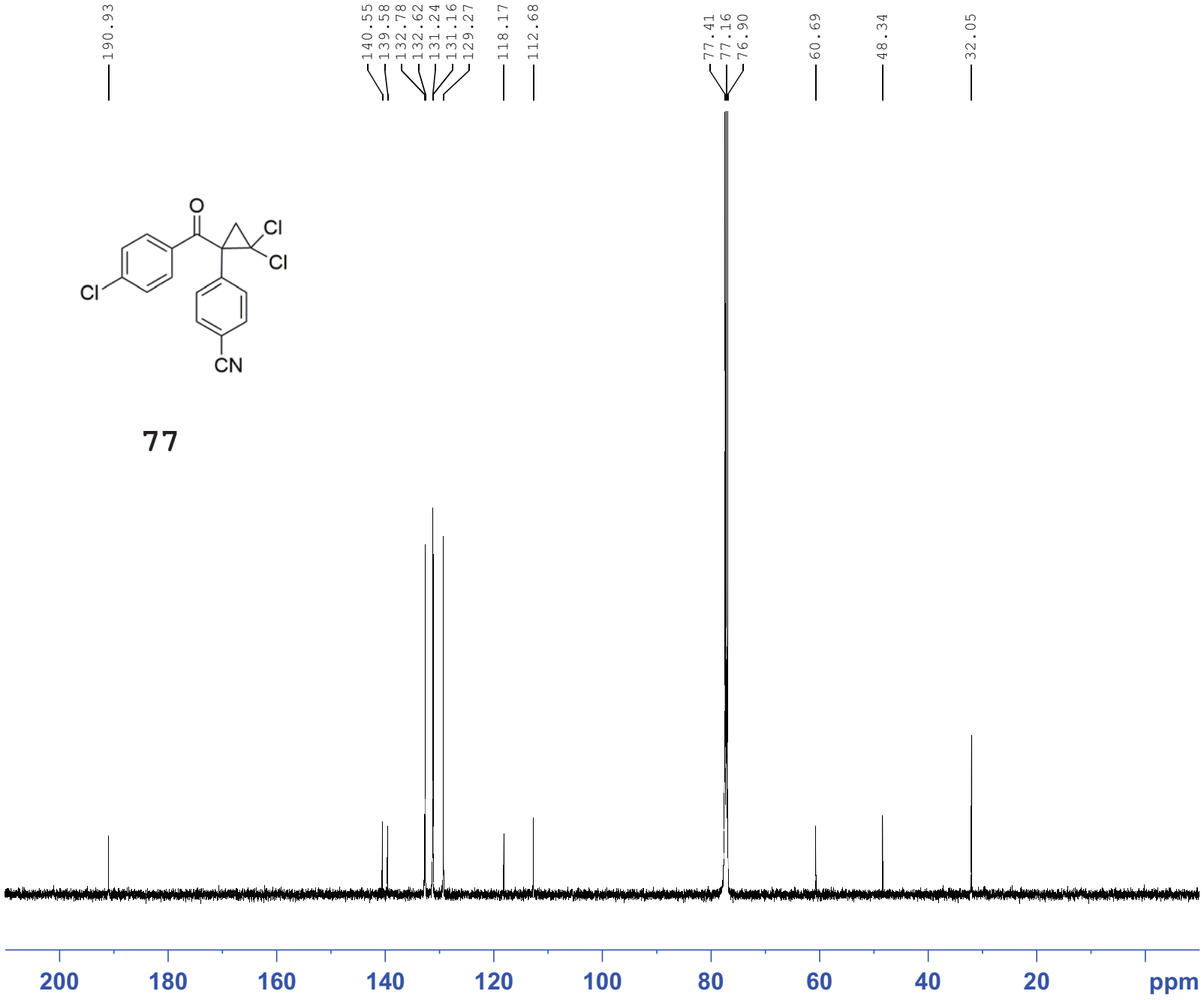


```
Current Data Parameters
NAME                 11200F
EXPNO                     4
PROCNO                    1

F2 - Acquisition Parameters
Date_              20220401
Time                 17.08
INSTRUM              spect
PROBHD   5 mm CPPBBO BB
PULPROG             zgpg30
TD                   65536
SOLVENT              CDCl3
NS                     300
DS                       4
SWH              29761.904 Hz
FIDRES            0.454131 Hz
AQ                1.1010048 sec
RG                   192.89
DW                  16.800 usec
DE                  18.00 usec
TE                  298.2 K
D1              2.00000000 sec
D11             0.03000000 sec
TD0                      1

======== CHANNEL f1 ========
SFO1           125.7703637 MHz
NUC1                    13C
P1                   10.50 usec
PLW1           57.00000000 W

======== CHANNEL f2 ========
SFO2           500.1320005 MHz
NUC2                    1H
CPDPRG[2           waltz16
PCPD2                80.00 usec
PLW2           20.00000000 W
PLW12           0.39550999 W
PLW13           0.25312999 W

F2 - Processing parameters
SI                   32768
SF             125.7577729 MHz
WDW                     EM
SSB       0
LB                    1.00 Hz
GB        0
PC                    1.40
```

Supplementary Figure 151. ^{13}C-NMR of compound **77**, recorded at 126 MHz and 25 °C in $CDCl_3$.

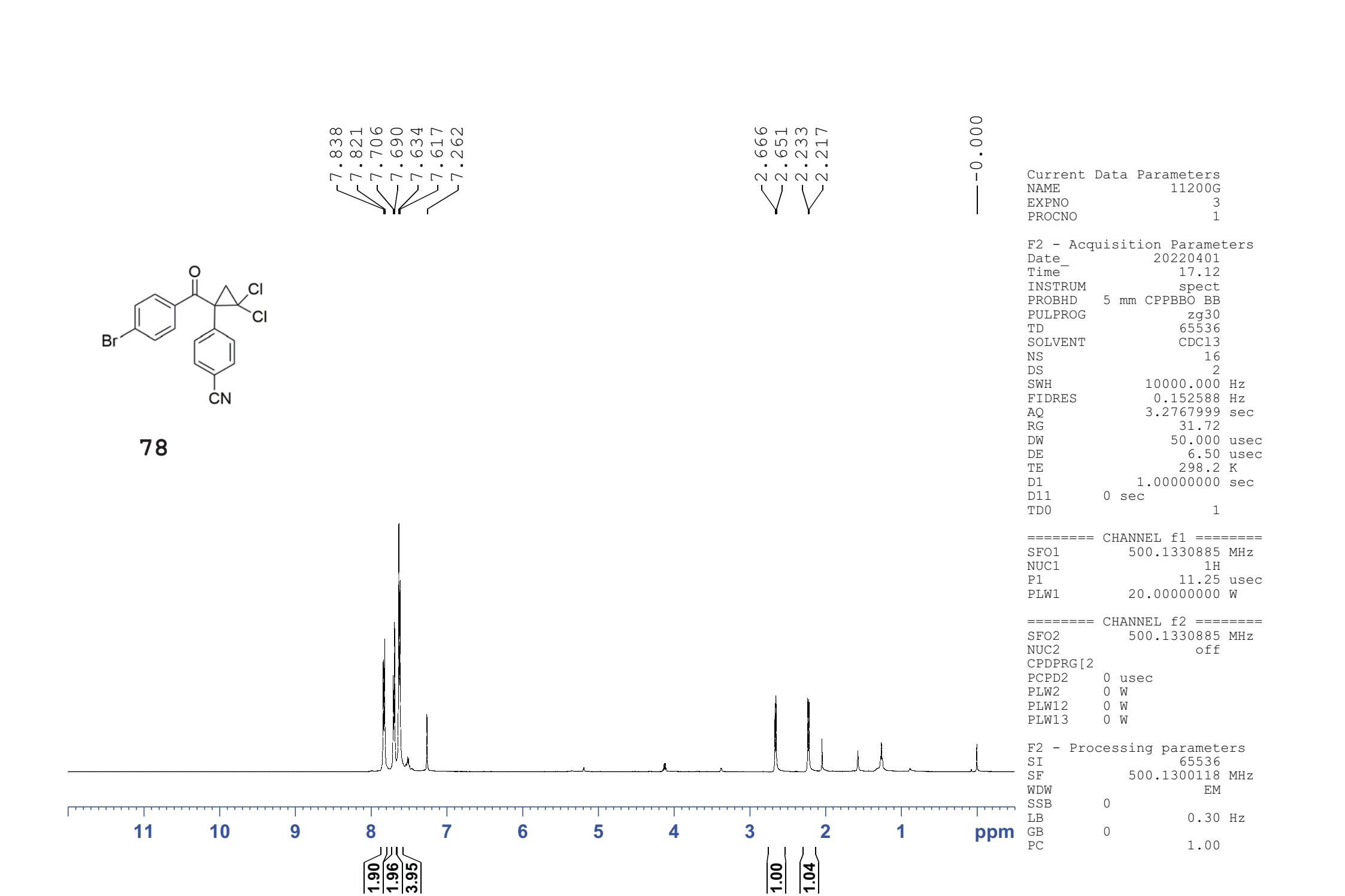

Supplementary Figure 152. ^{1}H-NMR of compound **78**, recorded at 500 MHz and 25 °C in $CDCl_3$.

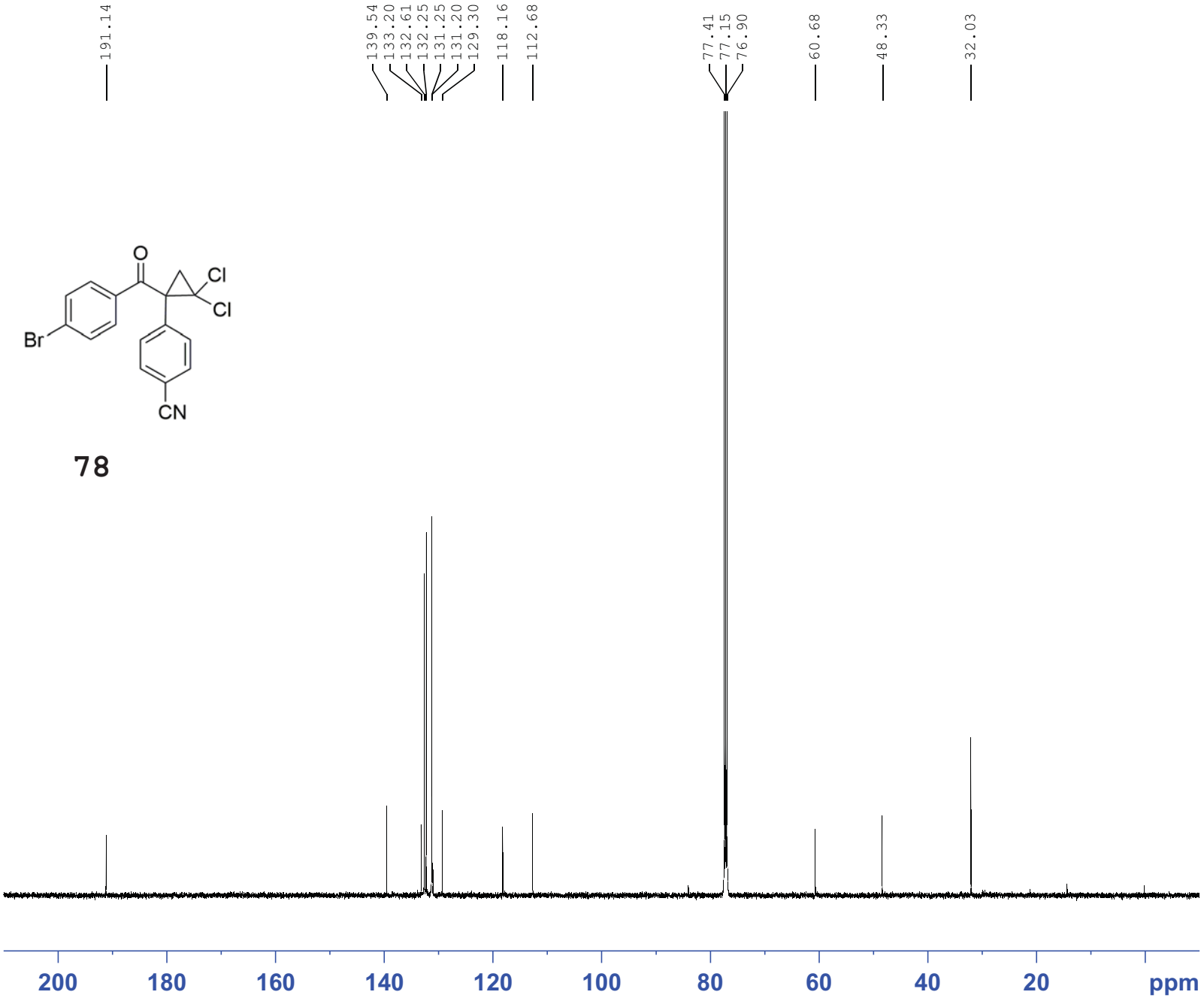


```
Current Data Parameters
NAME                 11200G
EXPNO                     4
PROCNO                    1

F2 - Acquisition Parameters
Date_              20220401
Time                 17.29
INSTRUM              spect
PROBHD   5 mm CPPBBO BB
PULPROG             zgpg30
TD                   65536
SOLVENT              CDCl3
NS                     300
DS                       4
SWH              29761.904 Hz
FIDRES            0.454131 Hz
AQ                1.1010048 sec
RG                   192.89
DW                  16.800 usec
DE                  18.00 usec
TE                  298.2 K
D1              2.00000000 sec
D11             0.03000000 sec
TD0                      1

======== CHANNEL f1 ========
SFO1           125.7703637 MHz
NUC1                    13C
P1                   10.50 usec
PLW1           57.00000000 W

======== CHANNEL f2 ========
SFO2           500.1320005 MHz
NUC2                    1H
CPDPRG[2           waltz16
PCPD2                80.00 usec
PLW2           20.00000000 W
PLW12           0.39550999 W
PLW13           0.25312999 W

F2 - Processing parameters
SI                   32768
SF             125.7577737 MHz
WDW                     EM
SSB          0
LB                    1.00 Hz
GB           0
PC                    1.40
```

Supplementary Figure 153. ^{13}C-NMR of compound **78**, recorded at 126 MHz and 25 °C in $CDCl_3$.

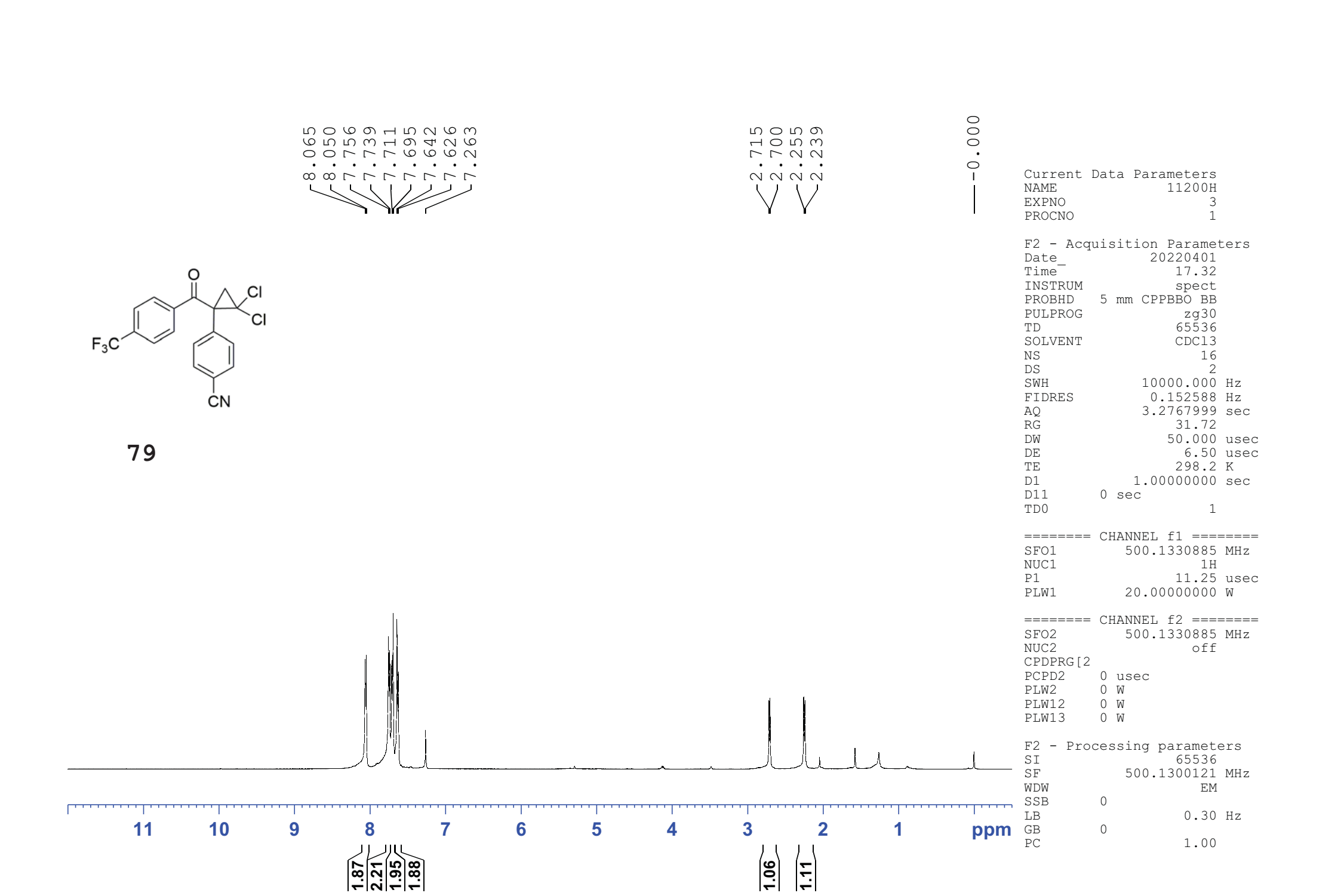

Supplementary Figure 154. ^{1}H-NMR of compound **79**, recorded at 500 MHz and 25 °C in $CDCl_3$.

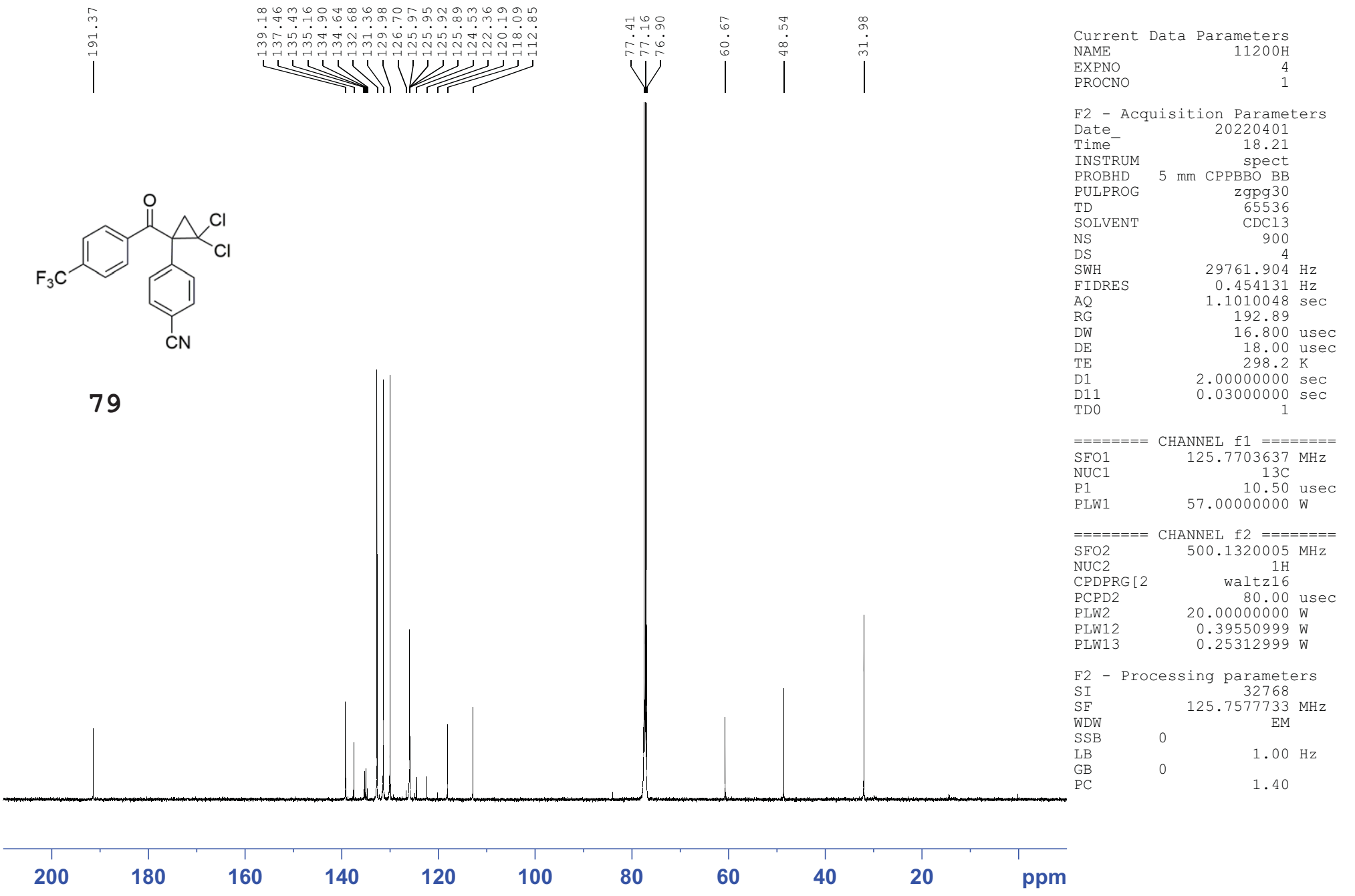


```
Current Data Parameters
NAME                 11200H
EXPNO                     4
PROCNO                    1

F2 - Acquisition Parameters
Date_              20220401
Time                 18.21
INSTRUM              spect
PROBHD    5 mm CPPBBO BB
PULPROG             zgpg30
TD                   65536
SOLVENT              CDCl3
NS                     900
DS                       4
SWH              29761.904 Hz
FIDRES            0.454131 Hz
AQ                1.1010048 sec
RG                   192.89
DW                   16.800 usec
DE                   18.00 usec
TE                   298.2 K
D1             2.00000000 sec
D11            0.03000000 sec
TD0                      1

======== CHANNEL f1 ========
SFO1           125.7703637 MHz
NUC1                   13C
P1                   10.50 usec
PLW1           57.00000000 W

======== CHANNEL f2 ========
SFO2           500.1320005 MHz
NUC2                   1H
CPDPRG[2           waltz16
PCPD2                80.00 usec
PLW2           20.00000000 W
PLW12           0.39550999 W
PLW13           0.25312999 W

F2 - Processing parameters
SI                   32768
SF             125.7577733 MHz
WDW                     EM
SSB          0
LB                    1.00 Hz
GB           0
PC                    1.40
```

Supplementary Figure 155. ^{13}C-NMR of compound **79**, recorded at 126 MHz and 25 °C in $CDCl_3$.

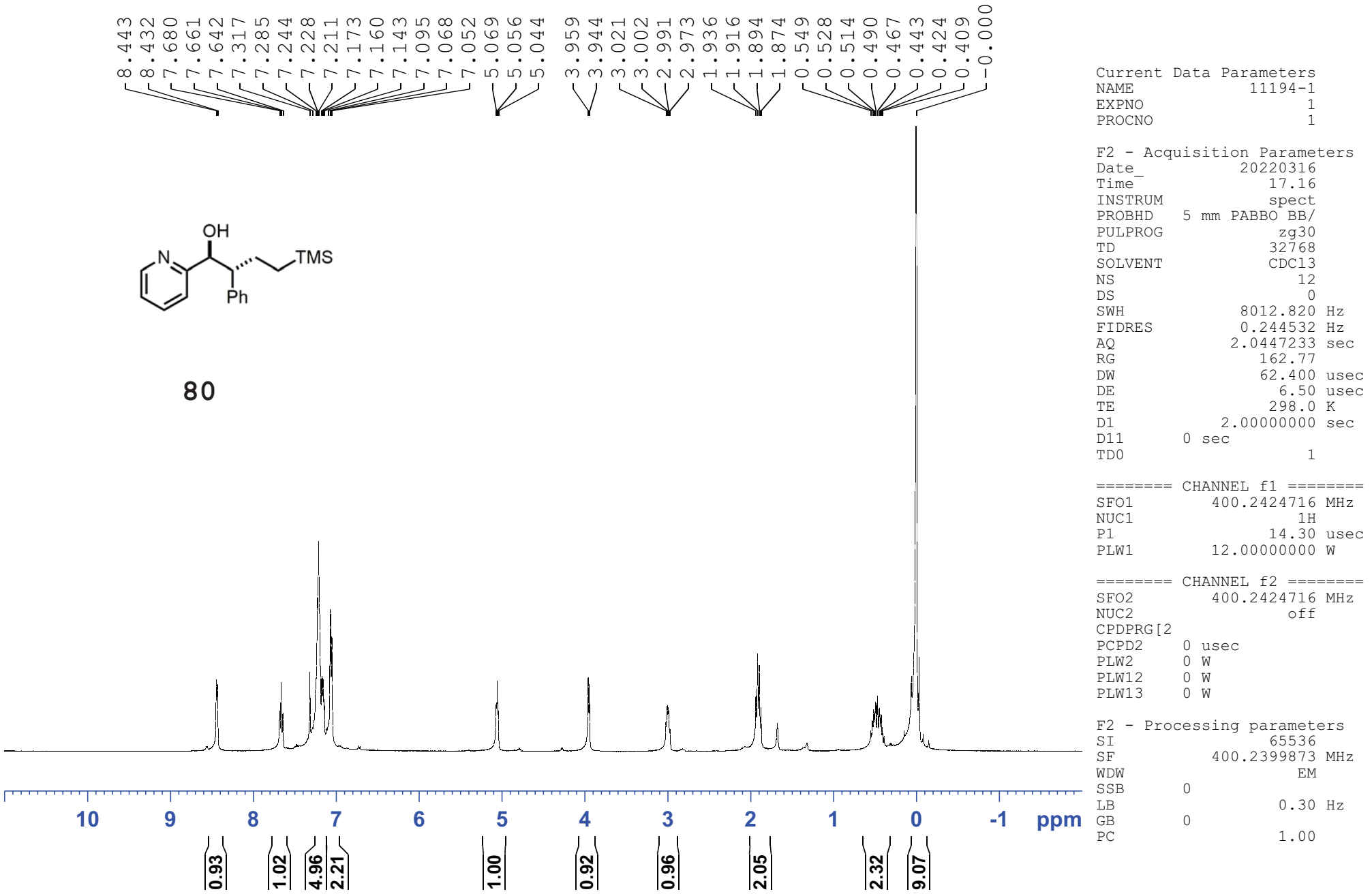


```
Current Data Parameters
NAME                  11194-1
EXPNO                       1
PROCNO                      1

F2 - Acquisition Parameters
Date_               20220316
Time                   17.16
INSTRUM                spect
PROBHD    5 mm PABBO BB/
PULPROG                 zg30
TD                     32768
SOLVENT                CDCl3
NS                        12
DS                         0
SWH                 8012.820 Hz
FIDRES              0.244532 Hz
AQ                2.0447233 sec
RG                    162.77
DW                    62.400 usec
DE                     6.50 usec
TE                    298.0 K
D1              2.00000000 sec
D11       0 sec
TD0                        1

======== CHANNEL f1 ========
SFO1            400.2424716 MHz
NUC1                       1H
P1                    14.30 usec
PLW1           12.00000000 W

======== CHANNEL f2 ========
SFO2            400.2424716 MHz
NUC2                     off
CPDPRG[2
PCPD2      0 usec
PLW2       0 W
PLW12      0 W
PLW13      0 W

F2 - Processing parameters
SI                      65536
SF              400.2399873 MHz
WDW                        EM
SSB        0
LB                    0.30 Hz
GB         0
PC                    1.00
```

Supplementary Figure 156. ^{1}H-NMR of compound **80**, recorded at 400 MHz and 25 °C in $CDCl_3$.

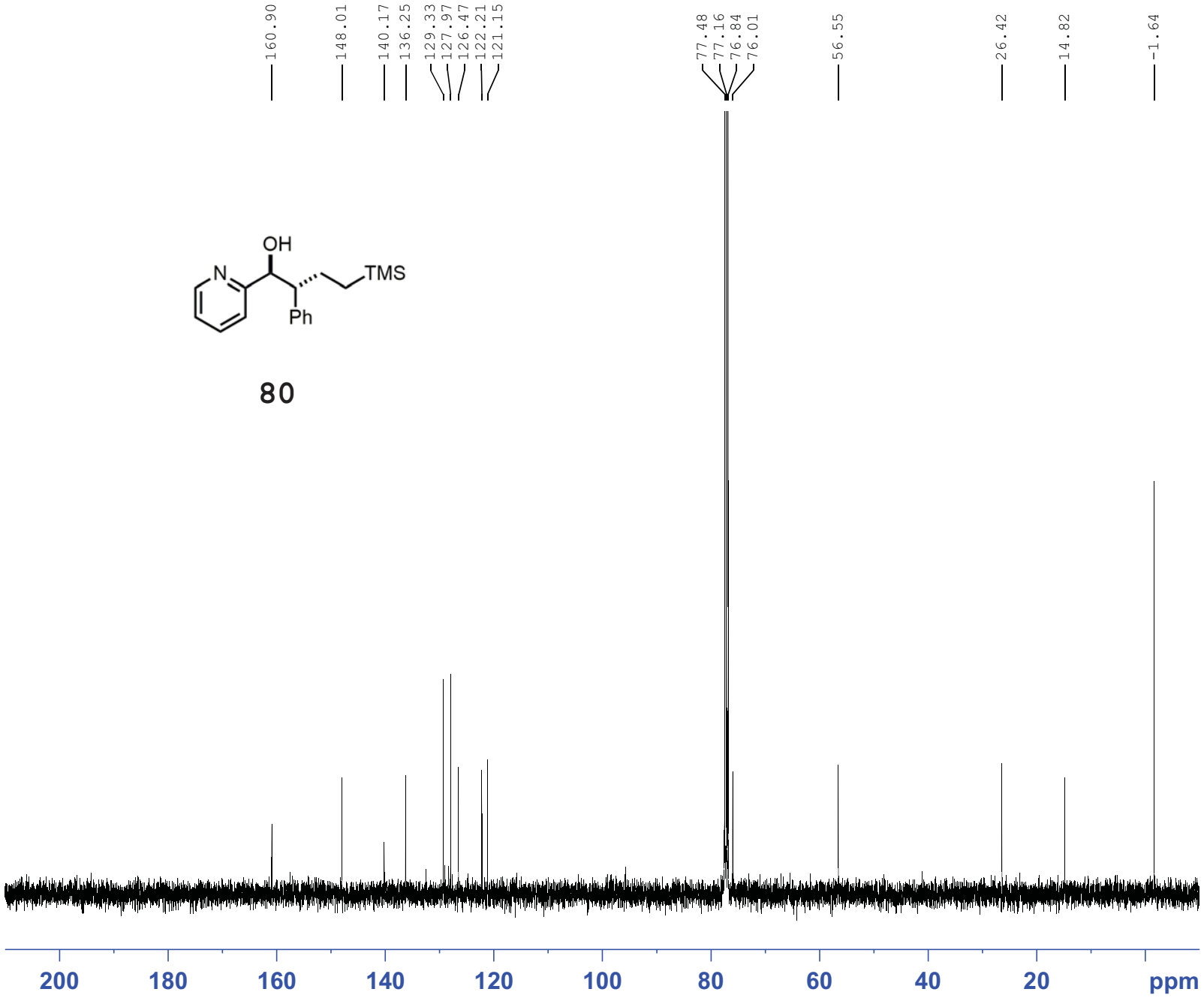


```
Current Data Parameters
NAME                  11194
EXPNO                     2
PROCNO                    1

F2 - Acquisition Parameters
Date_              20220316
Time                  16.55
INSTRUM               spect
PROBHD   5 mm PABBO BB/
PULPROG             zgpg30
TD                    65536
SOLVENT               CDCl3
NS                      150
DS                        4
SWH              24038.461 Hz
FIDRES            0.366798 Hz
AQ                1.3631488 sec
RG                   206.33
DW                   20.800 usec
DE                    6.50 usec
TE                   298.0 K
D1             2.00000000 sec
D11            0.03000000 sec
TD0                       1

======== CHANNEL f1 ========
SFO1           100.6504916 MHz
NUC1                    13C
P1                   10.00 usec
PLW1           54.00000000 W

======== CHANNEL f2 ========
SFO2           400.2416010 MHz
NUC2                    1H
CPDPRG[2           waltz16
PCPD2                90.00 usec
PLW2           12.00000000 W
PLW12           0.30294999 W
PLW13           0.24539000 W

F2 - Processing parameters
SI                    32768
SF             100.6404154 MHz
WDW                      EM
SSB       0
LB                    1.00 Hz
GB        0
PC                    1.40
```

Supplementary Figure 157. ^{13}C-NMR of compound **80**, recorded at 101 MHz and 25 °C in $CDCl_3$.

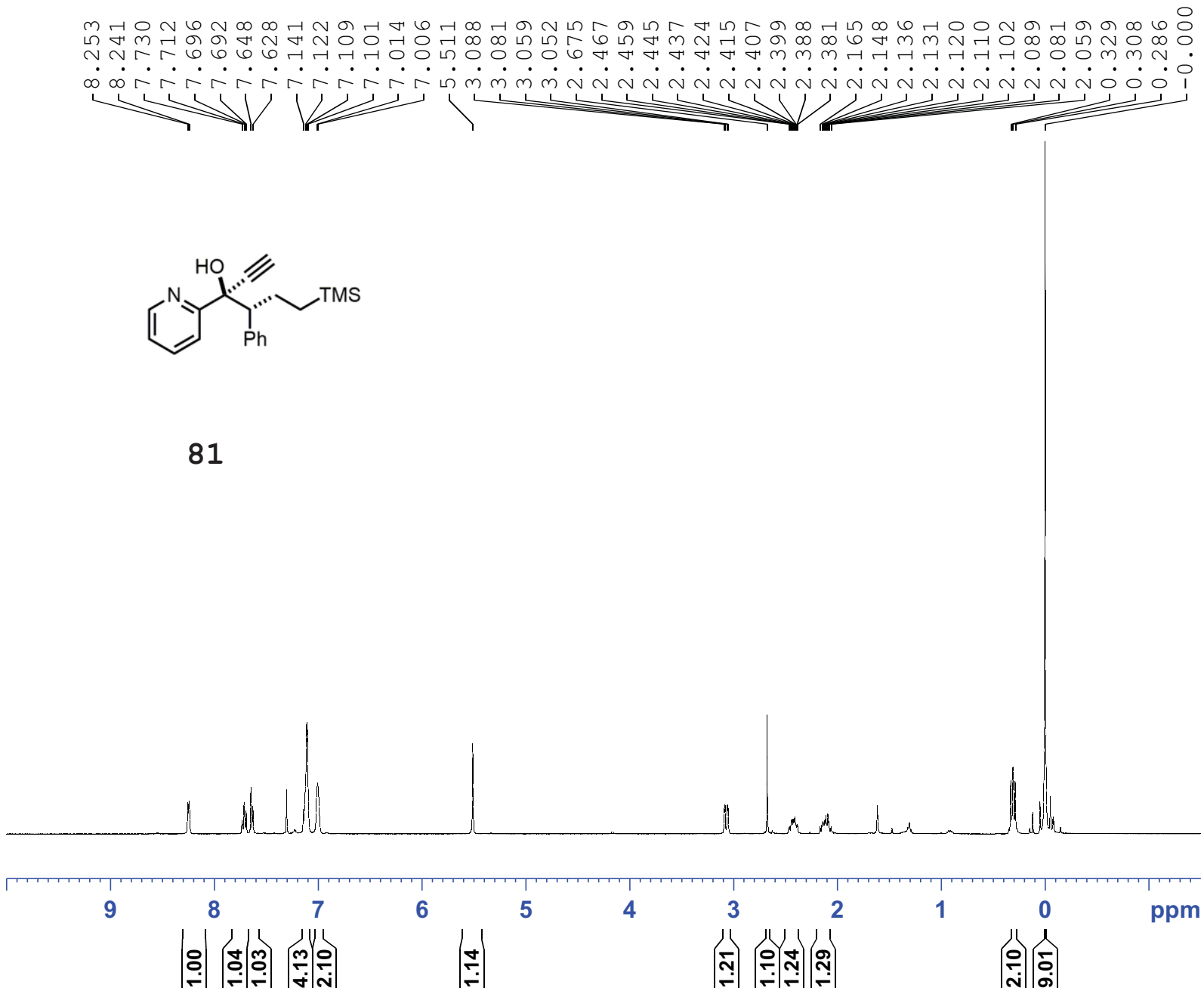


```
Current Data Parameters
NAME                  11195-1
EXPNO                       1
PROCNO                      1

F2 - Acquisition Parameters
Date_               20220316
Time                   17.20
INSTRUM                spect
PROBHD    5 mm PABBO BB/
PULPROG                 zg30
TD                     32768
SOLVENT                CDCl3
NS                        16
DS                         0
SWH                 8012.820 Hz
FIDRES              0.244532 Hz
AQ                2.0447233 sec
RG                    206.33
DW                    62.400 usec
DE                     6.50 usec
TE                    298.0 K
D1              2.00000000 sec
D11       0 sec
TD0                        1

======== CHANNEL f1 ========
SFO1            400.2424716 MHz
NUC1                       1H
P1                    14.30 usec
PLW1           12.00000000 W

======== CHANNEL f2 ========
SFO2            400.2424716 MHz
NUC2                     off
CPDPRG[2
PCPD2      0 usec
PLW2       0 W
PLW12      0 W
PLW13      0 W

F2 - Processing parameters
SI                      65536
SF              400.2399922 MHz
WDW                        EM
SSB        0
LB                    0.30 Hz
GB         0
PC                    1.00
```

Supplementary Figure 158. ^{1}H-NMR of compound **81**, recorded at 400 MHz and 25 °C in $CDCl_3$.

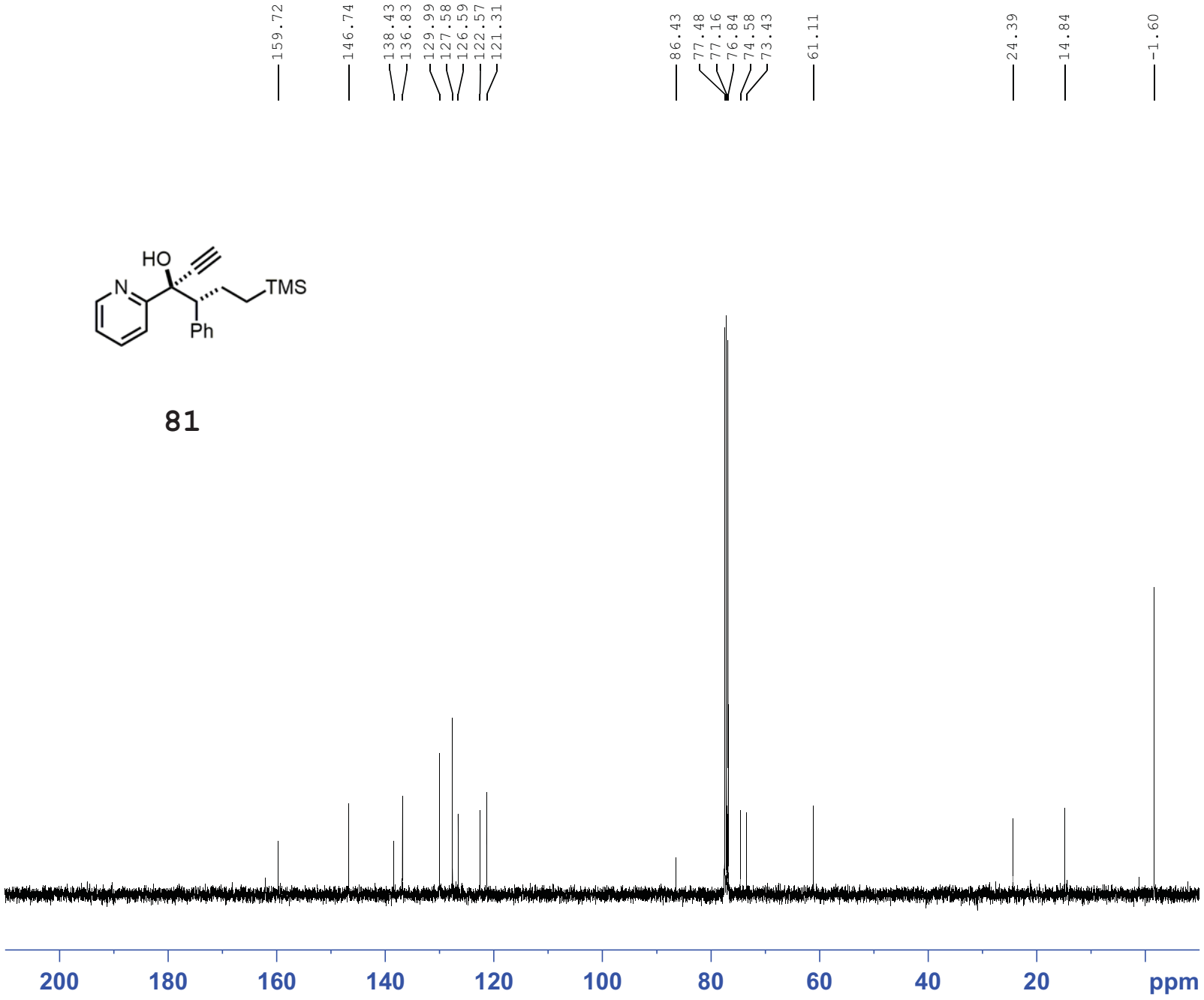


```
Current Data Parameters
NAME                 11195
EXPNO                    2
PROCNO                   1

F2 - Acquisition Parameters
Date_             20220316
Time                 17.08
INSTRUM              spect
PROBHD   5 mm PABBO BB/
PULPROG             zgpg30
TD                   65536
SOLVENT              CDCl3
NS                     110
DS                       4
SWH             24038.461 Hz
FIDRES           0.366798 Hz
AQ               1.3631488 sec
RG                  206.33
DW                  20.800 usec
DE                   6.50 usec
TE                  298.0 K
D1              2.00000000 sec
D11             0.03000000 sec
TD0                      1

======== CHANNEL f1 ========
SFO1           100.6504916 MHz
NUC1                   13C
P1                  10.00 usec
PLW1           54.00000000 W

======== CHANNEL f2 ========
SFO2           400.2416010 MHz
NUC2                   1H
CPDPRG[2           waltz16
PCPD2               90.00 usec
PLW2           12.00000000 W
PLW12           0.30294999 W
PLW13           0.24539000 W

F2 - Processing parameters
SI                   32768
SF             100.6404161 MHz
WDW                     EM
SSB          0
LB                   1.00 Hz
GB          0
PC                   1.40
```

Supplementary Figure 159. ^{13}C-NMR of compound **81**, recorded at 101 MHz and 25 °C in $CDCl_3$.

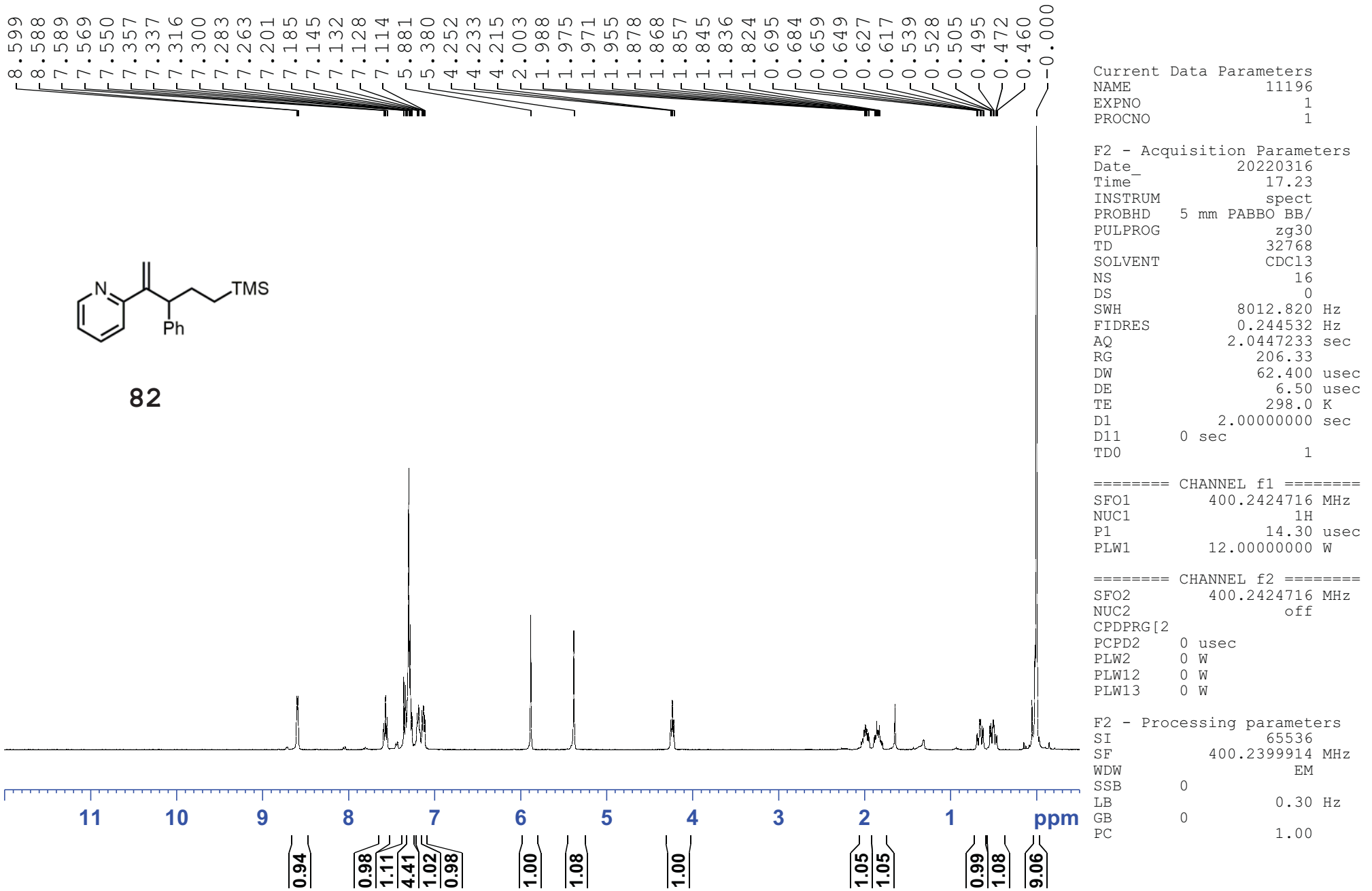


```
Current Data Parameters
NAME                  11196
EXPNO                     1
PROCNO                    1

F2 - Acquisition Parameters
Date_              20220316
Time                  17.23
INSTRUM               spect
PROBHD    5 mm PABBO BB/
PULPROG                zg30
TD                    32768
SOLVENT               CDCl3
NS                       16
DS                        0
SWH                8012.820 Hz
FIDRES             0.244532 Hz
AQ                2.0447233 sec
RG                   206.33
DW                   62.400 usec
DE                    6.50 usec
TE                   298.0 K
D1              2.00000000 sec
D11       0 sec
TD0                       1

======== CHANNEL f1 ========
SFO1           400.2424716 MHz
NUC1                      1H
P1                   14.30 usec
PLW1           12.00000000 W

======== CHANNEL f2 ========
SFO2           400.2424716 MHz
NUC2                    off
CPDPRG[2
PCPD2      0 usec
PLW2       0 W
PLW12      0 W
PLW13      0 W

F2 - Processing parameters
SI                     65536
SF             400.2399914 MHz
WDW                       EM
SSB        0
LB                   0.30 Hz
GB         0
PC                    1.00
```

Supplementary Figure 160. ^{1}H-NMR of compound **82**, recorded at 400 MHz and 25 °C in $CDCl_3$.

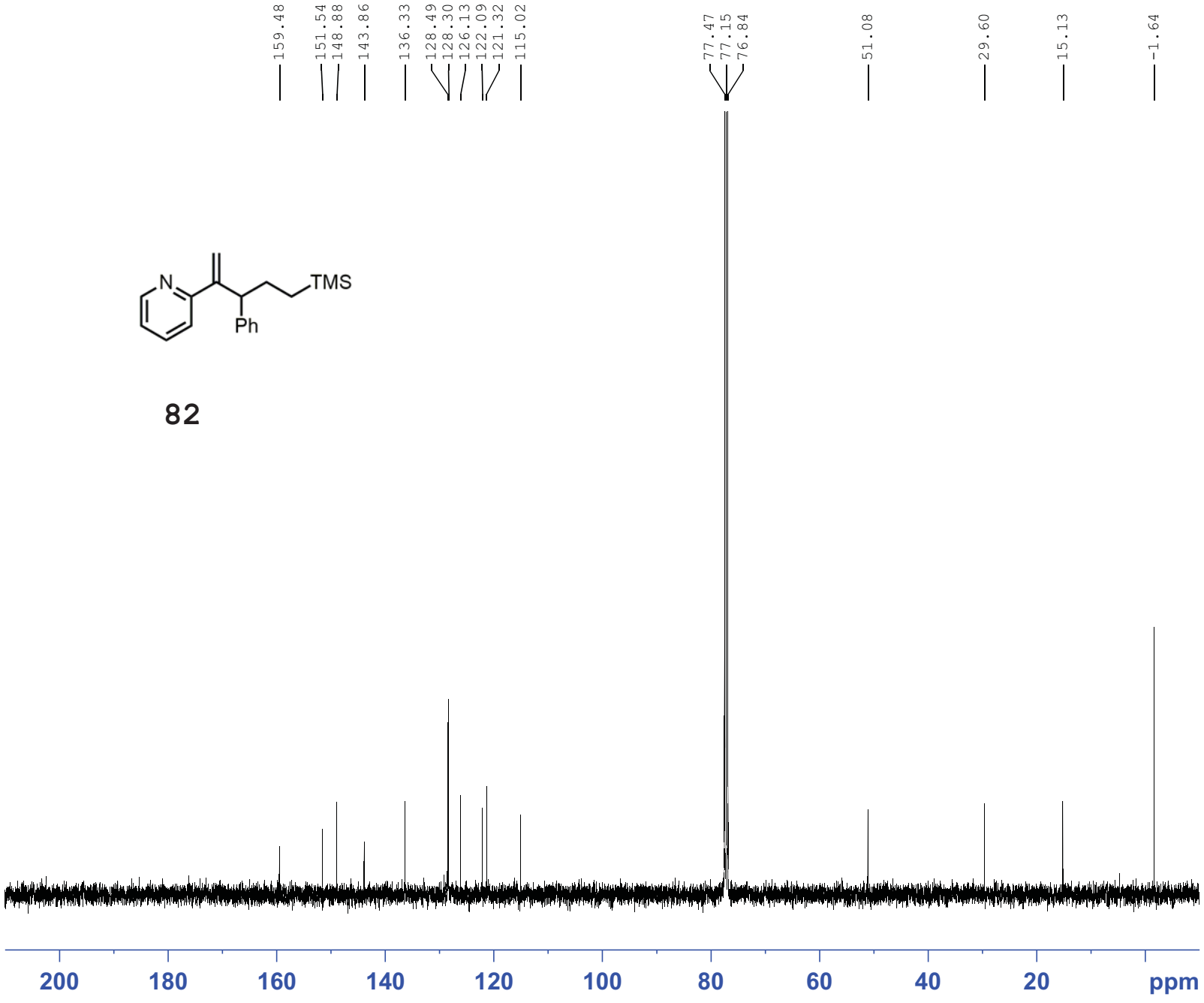


```
Current Data Parameters
NAME                 11196
EXPNO                    2
PROCNO                   1

F2 - Acquisition Parameters
Date_             20220316
Time                 17.25
INSTRUM              spect
PROBHD   5 mm PABBO BB/
PULPROG             zgpg30
TD                   65536
SOLVENT              CDCl3
NS                     200
DS                       4
SWH              24038.461 Hz
FIDRES            0.366798 Hz
AQ                1.3631488 sec
RG                   206.33
DW                  20.800 usec
DE                   6.50 usec
TE                  298.1 K
D1              2.00000000 sec
D11             0.03000000 sec
TD0                      1

======== CHANNEL f1 ========
SFO1           100.6504916 MHz
NUC1                    13C
P1                   10.00 usec
PLW1           54.00000000 W

======== CHANNEL f2 ========
SFO2           400.2416010 MHz
NUC2                    1H
CPDPRG[2           waltz16
PCPD2                90.00 usec
PLW2           12.00000000 W
PLW12           0.30294999 W
PLW13           0.24539000 W

F2 - Processing parameters
SI                   32768
SF             100.6404152 MHz
WDW                     EM
SSB          0
LB                    1.00 Hz
GB           0
PC                    1.40
```

Supplementary Figure 161. ^{13}C-NMR of compound **82**, recorded at 101 MHz and 25 °C in $CDCl_3$.

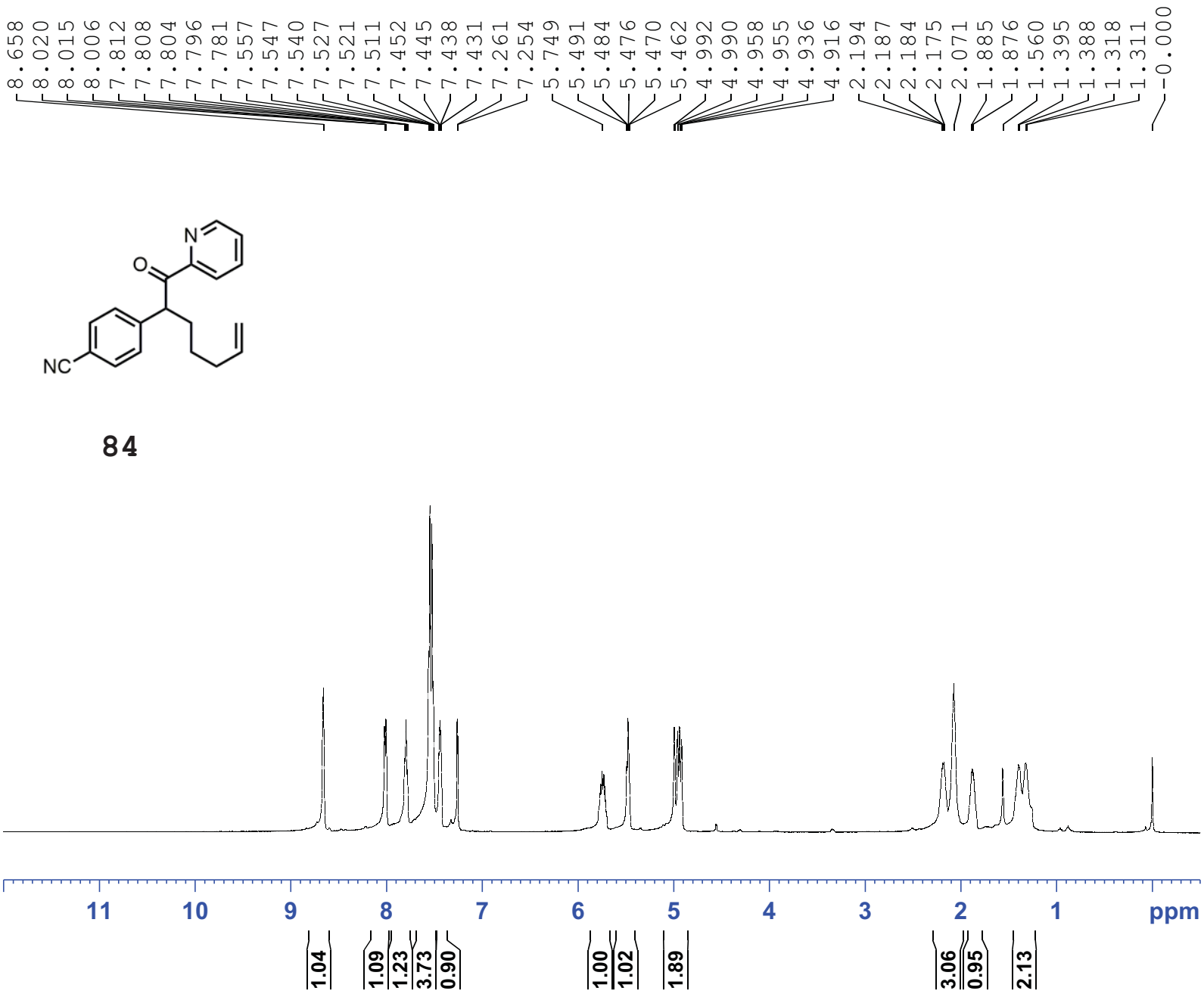


```
Current Data Parameters
NAME                  H-154H
EXPNO                      1
PROCNO                     1

F2 - Acquisition Parameters
Date_              20220301
Time                 20.39
INSTRUM              spect
PROBHD    5 mm CPPBBO BB
PULPROG               zg30
TD                   65536
SOLVENT              CDCl3
NS                      16
DS                       2
SWH             10000.000 Hz
FIDRES           0.152588 Hz
AQ               3.2767999 sec
RG                   62.06
DW                  50.000 usec
DE                   6.50 usec
TE                  298.2 K
D1              1.00000000 sec
D11       0 sec
TD0                      1

======== CHANNEL f1 ========
SFO1          500.1330885 MHz
NUC1                    1H
P1                  11.25 usec
PLW1          20.00000000 W

======== CHANNEL f2 ========
SFO2          500.1330885 MHz
NUC2                   off
CPDPRG[2
PCPD2      0 usec
PLW2       0 W
PLW12      0 W
PLW13      0 W

F2 - Processing parameters
SI                   65536
SF            500.1300143 MHz
WDW                     EM
SSB        0
LB                   0.30 Hz
GB         0
PC                   1.00
```

Supplementary Figure 162. ^{1}H-NMR of compound **84**, recorded at 500 MHz and 25 °C in $CDCl_3$.

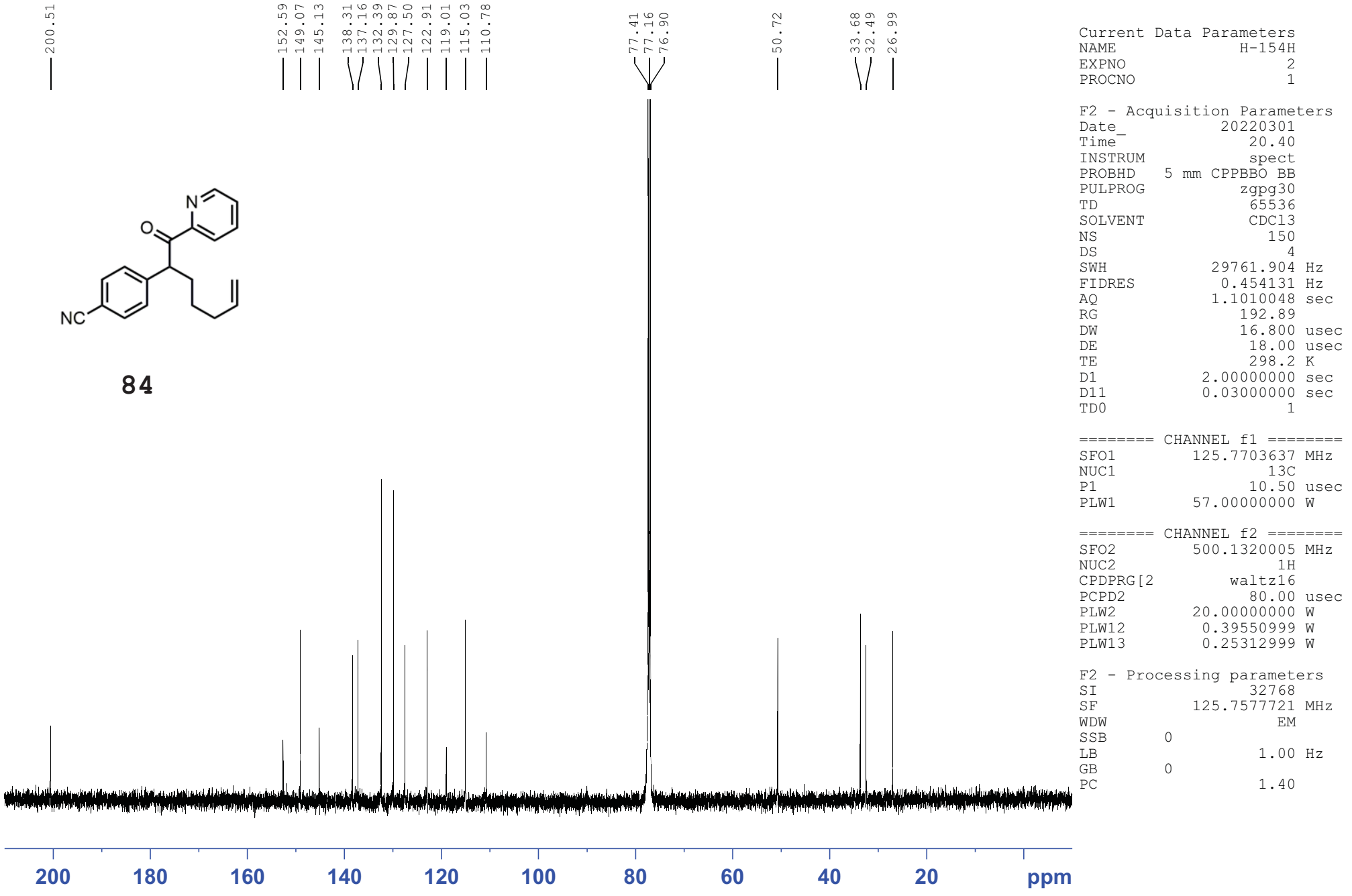


```
Current Data Parameters
NAME                 H-154H
EXPNO                     2
PROCNO                    1

F2 - Acquisition Parameters
Date_              20220301
Time                 20.40
INSTRUM              spect
PROBHD   5 mm CPPBBO BB
PULPROG             zgpg30
TD                   65536
SOLVENT              CDCl3
NS                     150
DS                       4
SWH              29761.904 Hz
FIDRES            0.454131 Hz
AQ                1.1010048 sec
RG                  192.89
DW                  16.800 usec
DE                  18.00 usec
TE                  298.2 K
D1              2.00000000 sec
D11             0.03000000 sec
TD0                      1

======== CHANNEL f1 ========
SFO1           125.7703637 MHz
NUC1                   13C
P1                  10.50 usec
PLW1           57.00000000 W

======== CHANNEL f2 ========
SFO2           500.1320005 MHz
NUC2                   1H
CPDPRG[2           waltz16
PCPD2               80.00 usec
PLW2           20.00000000 W
PLW12           0.39550999 W
PLW13           0.25312999 W

F2 - Processing parameters
SI                   32768
SF             125.7577721 MHz
WDW                     EM
SSB          0
LB                   1.00 Hz
GB           0
PC                   1.40
```

Supplementary Figure 163. ^{13}C-NMR of compound **84**, recorded at 126 MHz and 25 °C in $CDCl_3$.

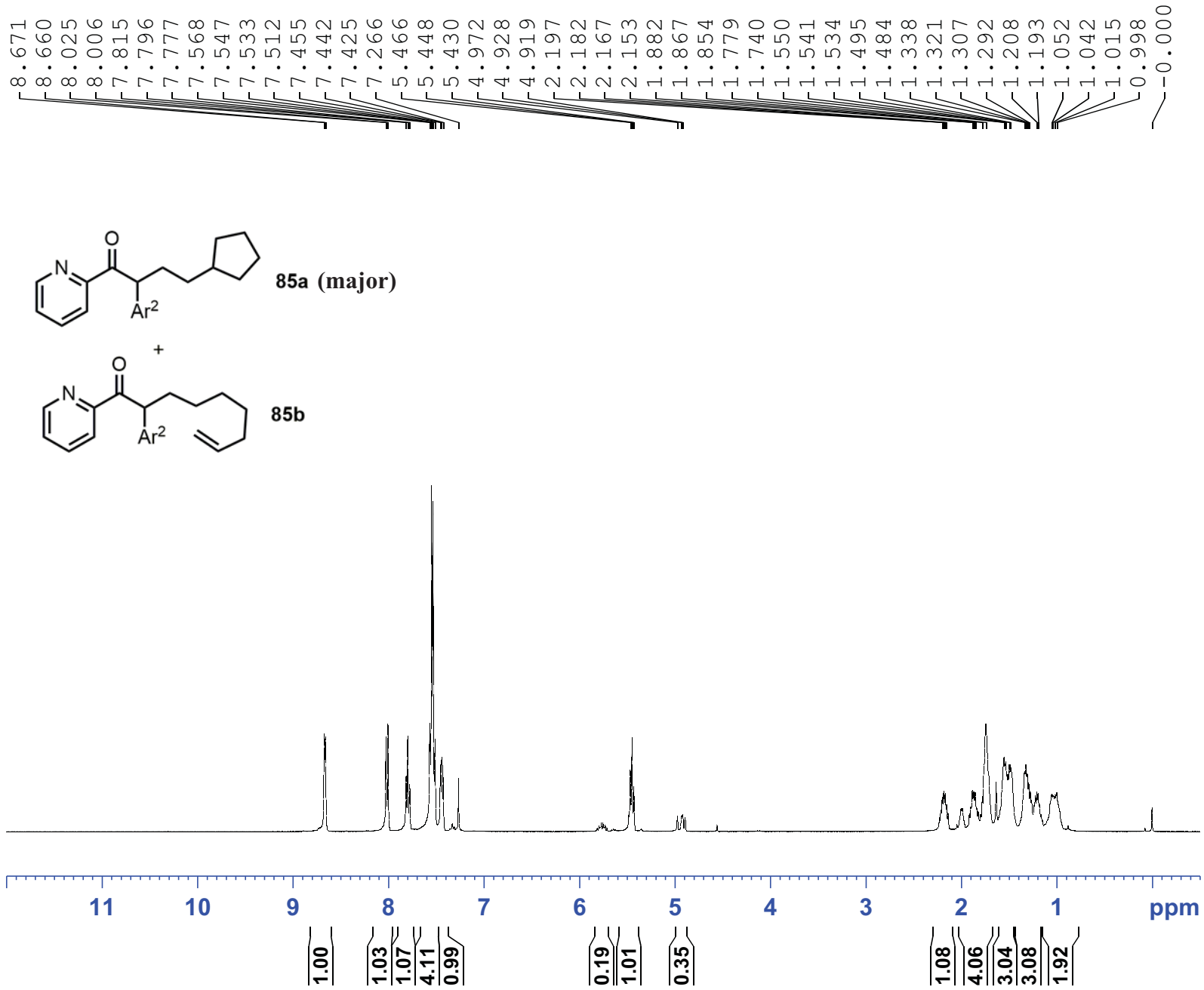


```
Current Data Parameters
NAME                  11178D
EXPNO                      1
PROCNO                     1

F2 - Acquisition Parameters
Date_              20220314
Time                  14.59
INSTRUM               spect
PROBHD    5 mm PABBO BB/
PULPROG                zg30
TD                    32768
SOLVENT               CDCl3
NS                       16
DS                        0
SWH               8012.820 Hz
FIDRES            0.244532 Hz
AQ                2.0447233 sec
RG                    92.09
DW                  62.400 usec
DE                   6.50 usec
TE                  298.0 K
D1              2.00000000 sec
D11       0 sec
TD0                       1

======== CHANNEL f1 ========
SFO1           400.2424716 MHz
NUC1                      1H
P1                    14.30 usec
PLW1           12.00000000 W

======== CHANNEL f2 ========
SFO2           400.2424716 MHz
NUC2                     off
CPDPRG[2
PCPD2      0 usec
PLW2       0 W
PLW12      0 W
PLW13      0 W

F2 - Processing parameters
SI                    65536
SF             400.2400077 MHz
WDW                      EM
SSB        0
LB                    0.30 Hz
GB         0
PC                    1.00
```

Supplementary Figure 164. ^{1}H-NMR of compound **85**, recorded at 400 MHz and 25 °C in $CDCl_3$.

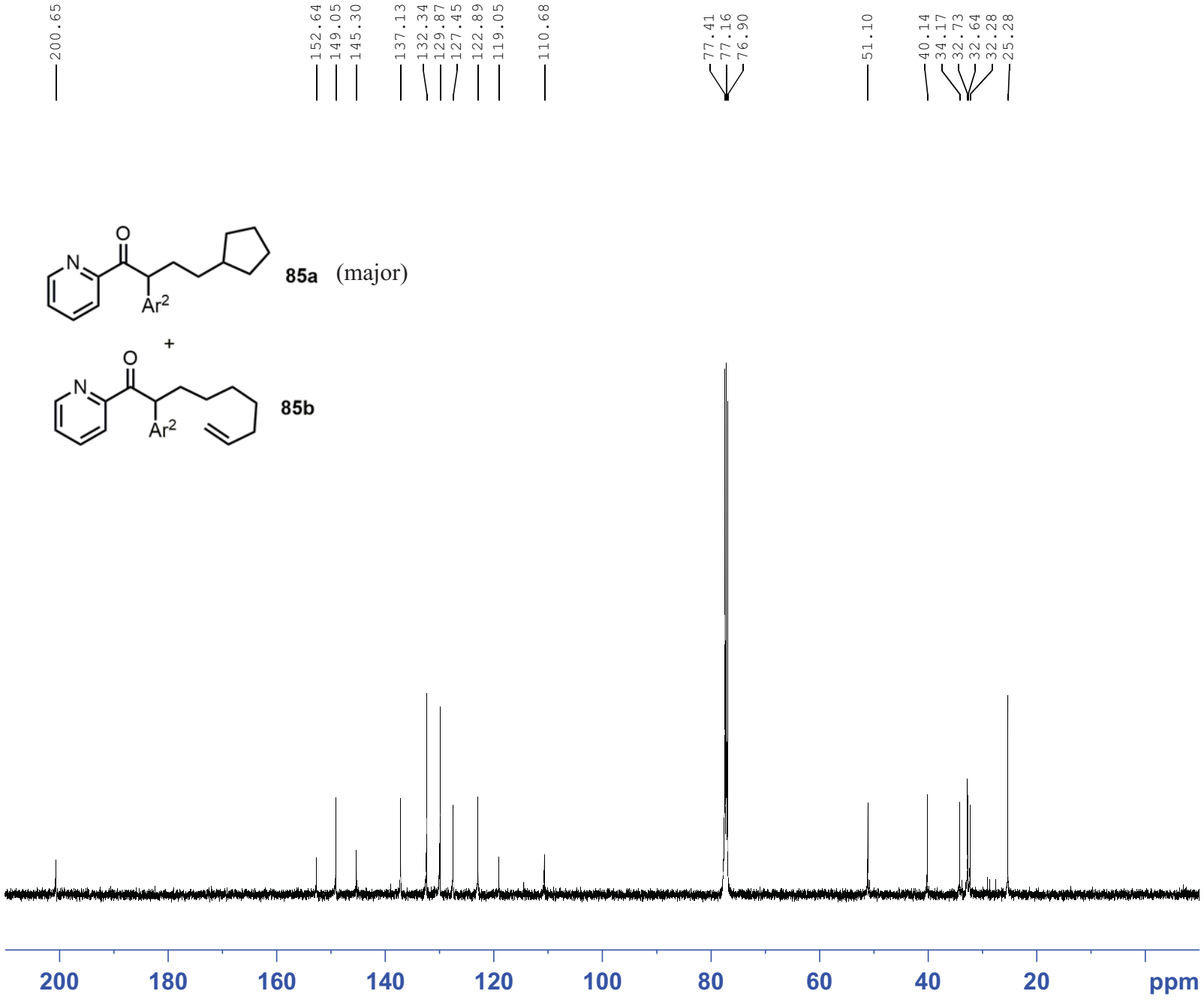


```
Current Data Parameters
NAME          11178D-500
EXPNO                  2
PROCNO                 1

F2 - Acquisition Parameters
Date_           20220311
Time              19.13
INSTRUM           spect
PROBHD   5 mm CPPBBO BB
PULPROG          zgpg30
TD                65536
SOLVENT           CDCl3
NS                  100
DS                    4
SWH           29761.904 Hz
FIDRES         0.454131 Hz
AQ             1.1010048 sec
RG                192.89
DW                16.800 usec
DE                18.00 usec
TE                298.2 K
D1           2.00000000 sec
D11          0.03000000 sec
TD0                   1

======== CHANNEL f1 ========
SFO1        125.7703637 MHz
NUC1                13C
P1                10.50 usec
PLW1        57.00000000 W

======== CHANNEL f2 ========
SFO2        500.1320005 MHz
NUC2                1H
CPDPRG[2         waltz16
PCPD2             80.00 usec
PLW2        20.00000000 W
PLW12        0.39550999 W
PLW13        0.25312999 W

F2 - Processing parameters
SI                 32768
SF          125.7577738 MHz
WDW                   EM
SSB       0
LB                 1.00 Hz
GB        0
PC                 1.40
```

Supplementary Figure 165. ^{13}C-NMR of compound **85**, recorded at 126 MHz and 25 °C in $CDCl_3$.

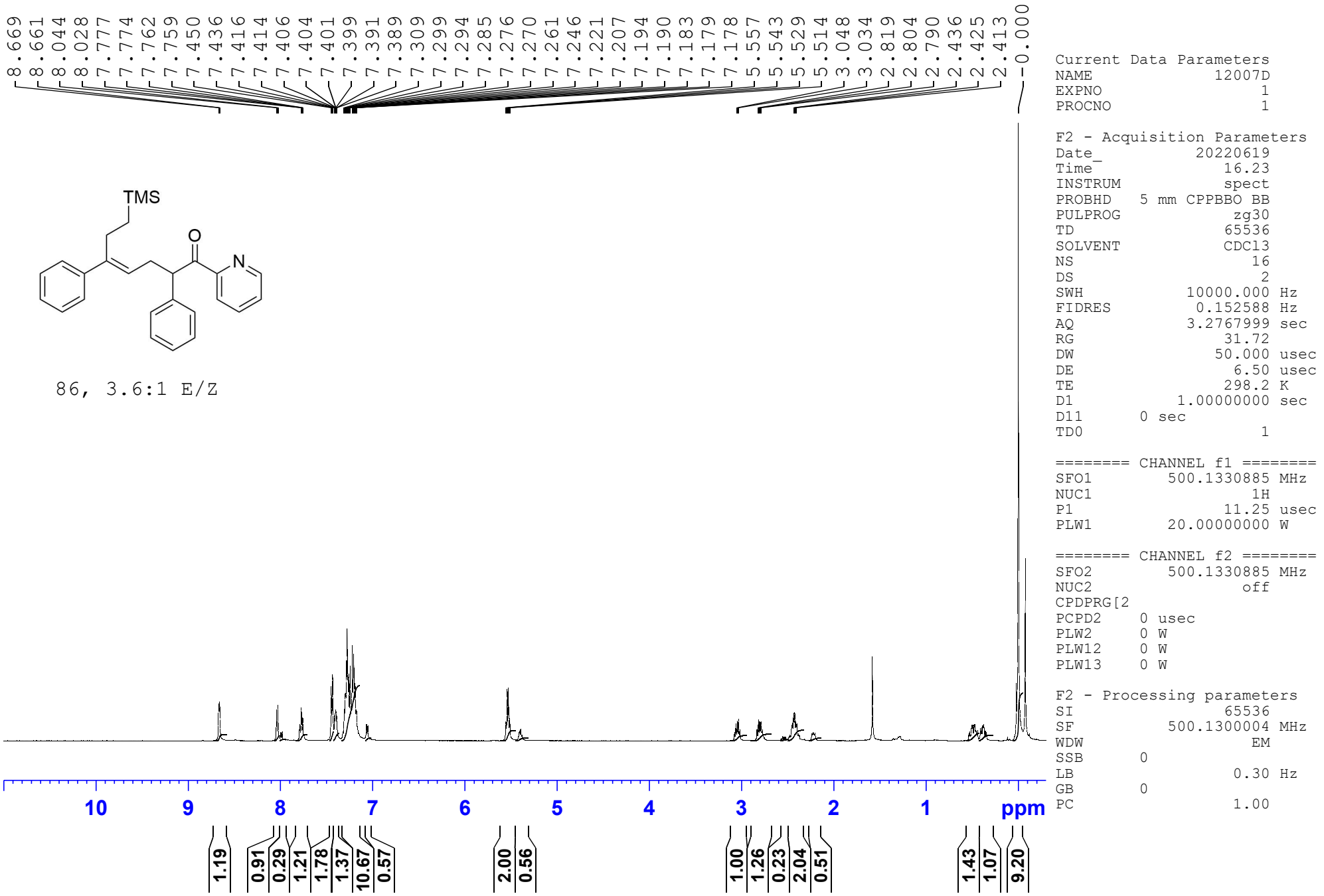


```
Current Data Parameters
NAME                  12007D
EXPNO                      1
PROCNO                     1

F2 - Acquisition Parameters
Date_              20220619
Time                  16.23
INSTRUM               spect
PROBHD    5 mm CPPBBO BB
PULPROG                zg30
TD                    65536
SOLVENT               CDCl3
NS                       16
DS                        2
SWH              10000.000 Hz
FIDRES            0.152588 Hz
AQ               3.2767999 sec
RG                    31.72
DW                   50.000 usec
DE                    6.50 usec
TE                   298.2 K
D1             1.00000000 sec
D11       0 sec
TD0                       1

======== CHANNEL f1 ========
SFO1           500.1330885 MHz
NUC1                     1H
P1                   11.25 usec
PLW1          20.00000000 W

======== CHANNEL f2 ========
SFO2           500.1330885 MHz
NUC2                   off
CPDPRG[2
PCPD2      0 usec
PLW2       0 W
PLW12      0 W
PLW13      0 W

F2 - Processing parameters
SI                    65536
SF           500.1300004 MHz
WDW                     EM
SSB        0
LB                   0.30 Hz
GB         0
PC                   1.00
```

Supplementary Figure 166. ^{1}H-NMR of compound **86**, recorded at 500 MHz and 25 °C in $CDCl_3$.

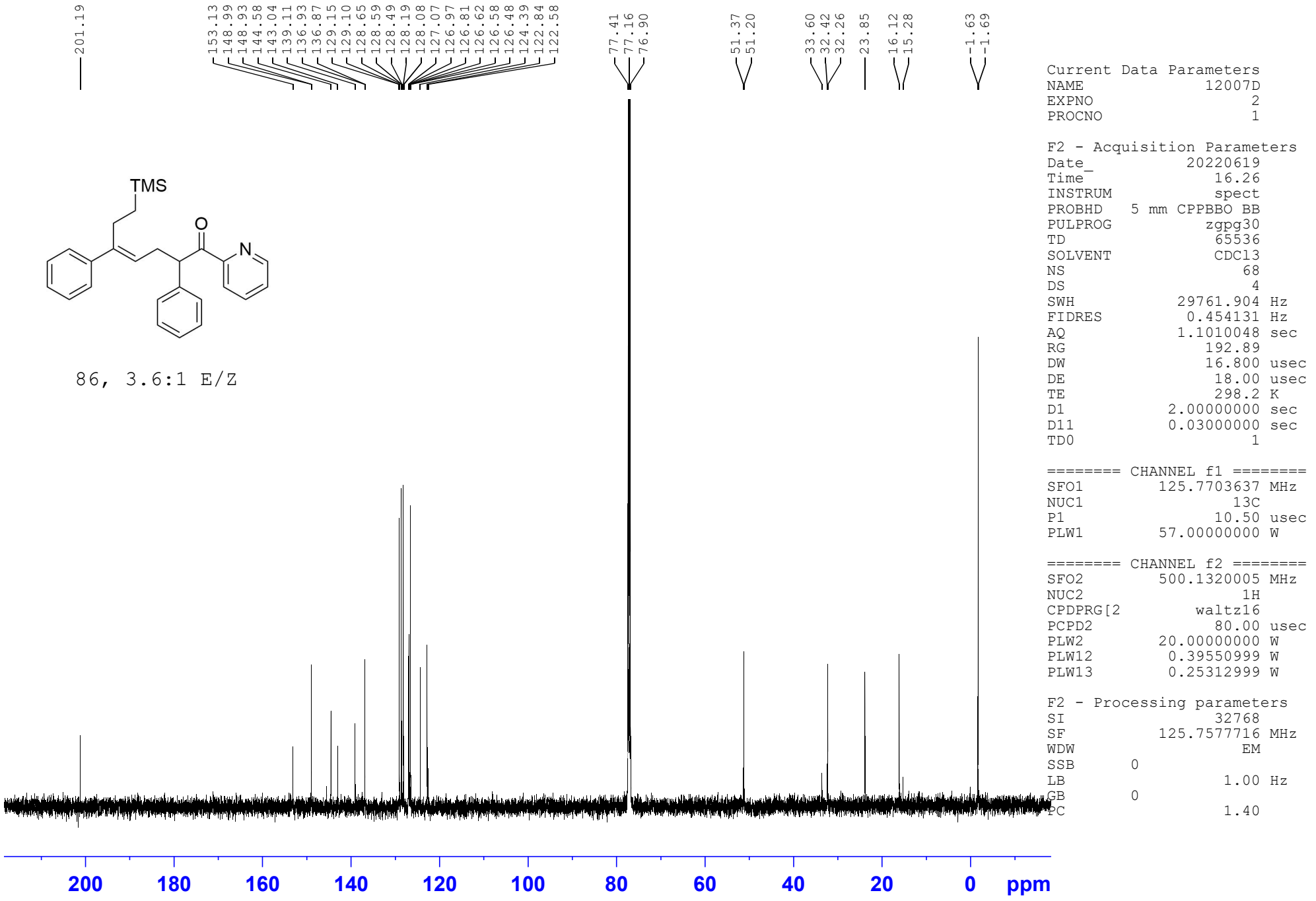


```
Current Data Parameters
NAME                  12007D
EXPNO                      2
PROCNO                     1

F2 - Acquisition Parameters
Date_              20220619
Time                  16.26
INSTRUM               spect
PROBHD   5 mm CPPBBO BB
PULPROG             zgpg30
TD                   65536
SOLVENT              CDCl3
NS                      68
DS                       4
SWH              29761.904 Hz
FIDRES            0.454131 Hz
AQ               1.1010048 sec
RG                  192.89
DW                  16.800 usec
DE                  18.00 usec
TE                  298.2 K
D1              2.00000000 sec
D11             0.03000000 sec
TD0                      1

======== CHANNEL f1 ========
SFO1           125.7703637 MHz
NUC1                    13C
P1                   10.50 usec
PLW1           57.00000000 W

======== CHANNEL f2 ========
SFO2           500.1320005 MHz
NUC2                    1H
CPDPRG[2             waltz16
PCPD2                80.00 usec
PLW2           20.00000000 W
PLW12           0.39550999 W
PLW13           0.25312999 W

F2 - Processing parameters
SI                      32768
SF             125.7577716 MHz
WDW                        EM
SSB       0
LB                    1.00 Hz
GB        0
PC                    1.40
```

Supplementary Figure 167. ^{13}C-NMR of compound **86**, recorded at 126 MHz and 25 °C in $CDCl_3$.

4. Supplementary References

1. Huang, H. M.; Bellotti, P.; Pfluger, P. M.; Schwarz, J. L.; Heidrich, B.; Glorius, F., Three-Component, Interrupted Radical Heck/Allylic Substitution Cascade Involving Unactivated Alkyl Bromides. *J. Am. Chem. Soc.* **2020,** *142* (22), 10173-10183.
