# Supplementary material for: Photoredox cooperative N-heterocyclic carbene/palladium-catalysed alkylacylation of alkenes
Source: Nat Commun. 2022 Sep 30;13:5754. doi: 10.1038/s41467-022-33444-0 (PMC9525644; doi:10.1038/s41467-022-33444-0)
Supplement: Supplementary file 3 — Description of Additional Supplementary Files [file 41467_2022_33444_MOESM3_ESM.pdf]

Supplementary Data 1: Source Data of Fig. 7 UV-Vis spectra and Fig. 8 Light on-off experiments. The UV-Vis absorption spectra of possible combinations of the substrates and reagents, and the change of yield of ketone 4 with light on-off.
